# The expression profiles of miRNA–mRNA of early response in genetically improved farmed tilapia (*Oreochromis niloticus*) liver by acute heat stress

**DOI:** 10.1038/s41598-017-09264-4

**Published:** 2017-08-18

**Authors:** Jun Qiang, Wen J. Bao, Fan Y. Tao, Jie He, Xia H. Li, Pao Xu, Lan Y. Sun

**Affiliations:** 1 Key Laboratory of Freshwater Fisheries and Germplasm Resources Utilization, Ministry of Agriculture, Freshwater Fisheries Research Centre, Chinese Academy of Fishery Sciences, 9 Shanshui East Road, Wuxi, Jiangsu 214081 China; 2 Wuxi Fisheries College, Nanjing Agricultural University, 9 Shanshui East Road, Wuxi, Jiangsu 214081 China

## Abstract

Genetically improved farmed **tilapia** (GIFT, *Oreochromis niloticus*) are commercially important fish that are cultured in China. GIFT are highly susceptible to diseases when exposed to high temperatures in summer. Better understanding the GIFT regulatory response to heat stress will not only help in determining the relationship between heat stress signalling pathways and adaption mechanisms, but will also contribute to breeding new high-temperature tolerant strains of GIFT. In this study, we built control (28 °C) and heat-treated (37.5 °C) groups, and extracted RNA from the liver tissues for high-throughput next-generation sequencing to study the miRNA and mRNA expression profiles. We identified 28 differentially expressed (DE) miRNAs and 744 DE mRNAs between the control and heat-treated groups and annotated them using the KEGG database. A total of 38 target genes were predicted for 21 of the DE miRNAs, including 64 negative miRNA–mRNA interactions. We verified 15 DE miRNA–mRNA pairs and 16 other DE mRNAs by quantitative real-time PCR. Important regulatory pathways involved in the early response of GIFT to heat stress included organism system, metabolism, and diseases. Our findings will facilitate the understanding of regulatory pathways affected by acute heat stress, which will help to better prevent heat damage to GIFT.

## Introduction

For cold-blooded animals such as fish, when the ambient temperature changes the new environmental conditions induce fish to undergo physiological adaptation changes, such as increased body temperature, reduced oxygen tolerance, and decreased metabolic rates^1, 2^. Increased water temperatures have been associated with increased fish feeding rates and growth^3^. However, when the water temperature exceeds a fish’s optimum temperature, its immune defence, digestion enzyme activity, protein synthesis, and growth processes are repressed^2–4^. Thus, a better understanding of the molecular regulatory response of fish to heat stress will not only provide crucial information about the relationship between heat stress signalling pathways and adaption mechanisms, but will also help in breeding new high-temperature tolerant strains of fish.

Genetically improved farmed tilapia (GIFT, *Oreochromis niloticus*) are an important source of affordable, high-yield protein. GIFT are cultured mainly in southern China, in places such as Hainan, Guangxi, Guangdong, and Fujian, because GIFT can tolerate relatively high temperatures but cannot survive in water below 11 °C^5^. The normal temperature range for tilapia growth is 20–33 °C, and at temperatures above 37 °C, their mortality suddenly increases^6, 7^. In recent years, the summer temperatures in southern China have been above 36 °C and water temperatures have reached 35 °C. High temperatures can result in liver damage, reduced lysozyme activity, reduced phagocytic function of white blood cells, and reduced immunoglobulin levels in blood, which eventually increase the susceptibly of tilapia to infectious diseases^8–11^. GIFT are not only an important cultured food fish, but are also an ideal model organism for the study of signal transduction and regulatory mechanisms under heat stress conditions.

MicroRNAs (miRNA) are a class of small non-coding RNAs (18–25 nucleotides) that function in RNA silencing and post-transcriptional regulation of gene expression by binding to the 3′-untranslated regions of target mRNAs^12^. MiRNA expression levels are regulated in response to temperature stresses. For example, 18 miRNAs were up-regulated and 11 were down-regulated in rat jejunum after heat treatment^13^, and 25 miRNAs were differentially expressed in zebrafish (*Danio rerio*) in response to cold stress^14^. The regulatory function of miRNAs is an important factor in the adaptive response of fish to heat stress.

Heat stress in fish has been shown to alter the expression profiles of genes involved in immune regulation and stress adaptation. For example, the expression levels of immune-related genes (e.g., genes coding immunoglobulin M, lysozyme, hepcidin, and transferrin) were significantly up-regulated in turbot (*Scophthalmus maximus*) 48 h post-heat stress^15^. Heat shock proteins (HSPs) play important roles in fish exposed to environmental stresses. For example, some highly conserved HSPs were synthesized in sculpin (*Oligocottus maculosus*)^16^ and turbot^17^ inresponse to heat stress. Fatty acid composition and utilization in fish were also shown to be affected by heat stress^18^. The gene coding ∆6-desaturase-α, which is involved in polyunsaturated fatty acid biosynthesis, was up-regulated in common carp (*Cyprinus carpio*) by heat stress, and its up-regulation helped to increase fatty acid metabolism and maintain the fluidity of cell membranes^19^. The proteins identified in these studies were generated from known and functional protein-coding genes; however, the significance of changes in the mRNA transcriptomes of fish in response to heat stress is far from clear.

Currently, limited information is available about the effects of acute heat stress on GIFT and the possible regulatory pathways involved. We hypothesized that various regulatory pathways will be affected by acute heat stress and aimed to find out why GIFT were highly susceptible to infectious disease when exposed to heat stress. The liver tissue of fish is extremely sensitive to changes in the external environment and is an important biological target organ that has been used in stress response and environmental monitoring^5^. We determined the expression profiles of miRNAs and mRNAs in liver of GIFT in control and heat stress-treated groups by high-throughput next-generation sequencing. Differently expressed (DE) miRNAs and DE mRNAs were jointly analysed in biological pathways related to heat stress. Our findings offer a deeper insight into the molecular mechanisms involved in the heat stress response in GIFT.

## Results

### Assessment of 48-h median lethal temperature (48-h LT_50_) in GIFT under heat stress

An increase of water temperature (from 35.5 °C to 39 °C) significantly increased the mortality of the GIFT after 48 h under heat stress (Table 1). Temperature and mortality were taken as independent and dependent variables respectively. The regression equation was Y = −1172.738 + 32.619X (R = 0.963, *P* < 0.001). The 48-h LT_50_ was determined as 37.49 °C by a linear interpolation method. Therefore, 37.5 °C was chosen as the heat stress temperature in this study.

**Table 1:** Comparison of the cumulative mortality of GIFT at high temperature stress for 48 h.

Temperature	Fish death in each tank at 48 h			Mortality
	Group 1	Group 2	Group 3	
35.5	0	0	0	0.00
36	0	0	0	0.00
36.5	1	1	0	6.67
37	5	3	5	43.33
37.5	5	7	6	60.00
38	7	7	6	66.67
38.5	8	8	9	83.33
39	9	10	9	96.67

**Figure 1:**
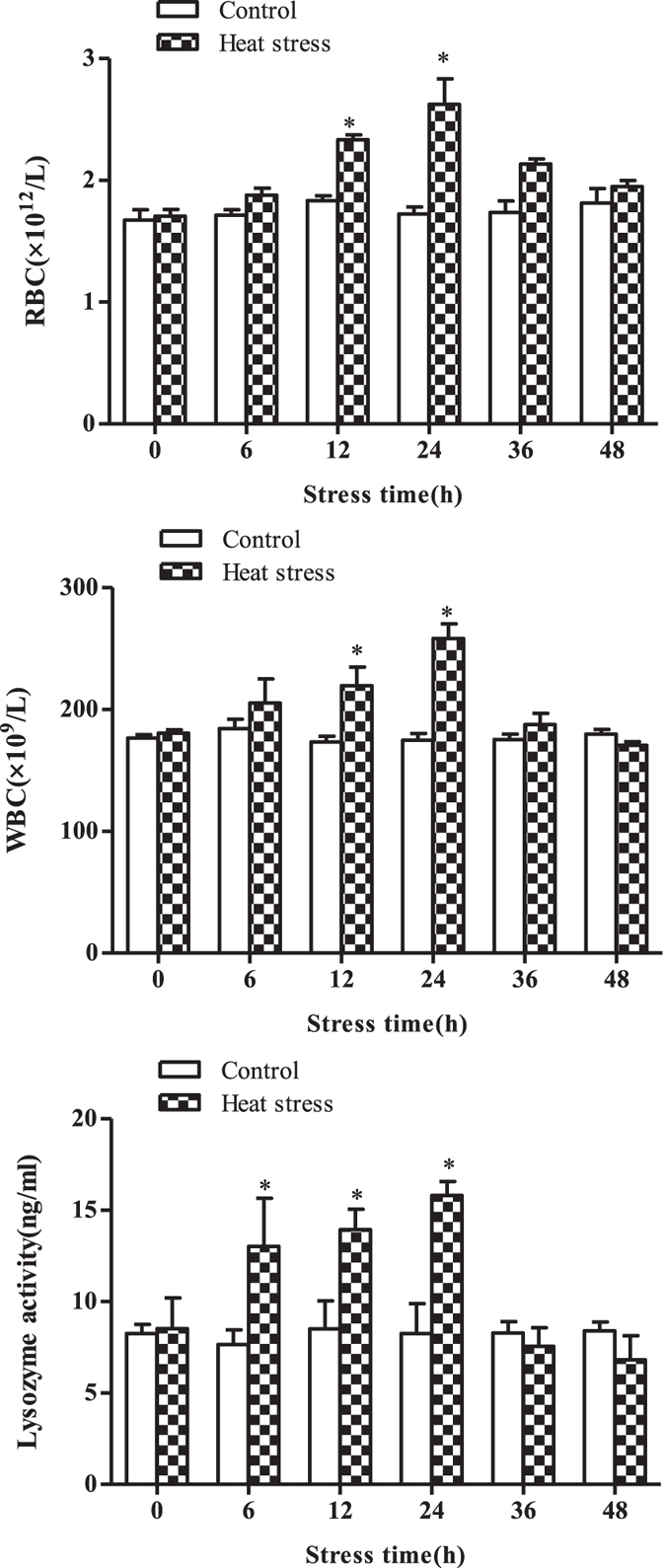
Red blood cell (RBC) and white blood cell (WBC) counts in blood samples, and serum lysozyme activity in GIFT between control (CO) and 37.5 °C heat-treated (HTS) groups for 48 h. Paired-samples t tests were used to compare different sampling times of the CO or HTS groups, which are indicated by asterisks.

At 12 h post-37.5 °C stress, a large number of fish were still on the bottom of the tank and exhibited reduced movement and, at 24 h post-heat stress, some fish began to show imbalance and lay on the tank bottom. When the abdomen of the fish was touched gently, the fish would swim quickly; such fish soon died. The red blood cell (RBC) and white blood cell (WBC) counts and serum lysozyme activity in the heat-stressed GIFT increased gradually from 0 h to 24 h, and reached peak values at 24 h; then significantly decreased at 36 h and 48 h (Fig. 1). Based on this result, we considered 24 h post-37.5 °C stress as an important threshold for GIFT. Therefore, we used liver from 24-h heat-stressed GIFT to investigate the regulatory mechanisms of miRNA–mRNA interactions.

### Expression profiling of miRNAs in heat-stressed GIFT liver

**Figure 2:**
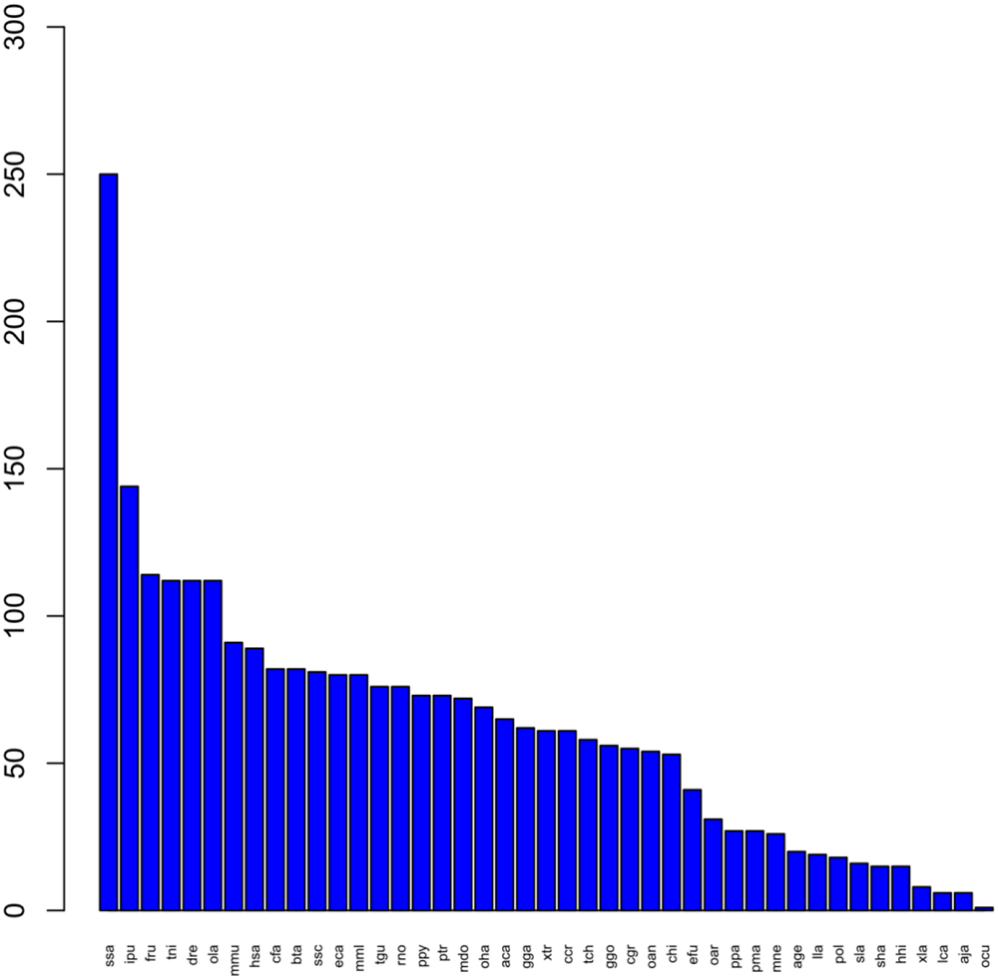
Conservation profiles of the identified GIFT liver miRNAs with miRNAs from other fish species, including *Salmo salar* (ssa), *Ictalurus punctatus* (ipu), *Fugu rubripes* (fru), *Tetraodon nigroviridis* (tni), Zebrafish (dre), and *Oryzias latipes* (ola).

We built and sequenced six miRNA libraries; three from 28°C control groups (CO-1, CO-2, and CO-3) and three from 37.5 °C-treated groups (HTS-1, HTS-2, and HTS-3). The biological replicates had good repeatability (Supplementary Fig. S1). A total of 13,602,611, 13,029,563, 14,426,681, 14,395,061, 11,506,732,and 11,963,811 raw reads were obtained from the CO-1, CO-2, CO-3, HTS-1, HTS-2, and HTS-3 libraries, respectively (Supplementary Table S1). After removing reads that contained adaptor sequences and low-quality reads, 7,336,868, 6,080,277, 5,233,861, 7,645,402, 6,614,342,and 6,033,205 clean reads remained in the CO-1, CO-2, CO-3, HTS-1, HTS-2, and HTS-3 libraries, respectively. The 22-nt long reads were the most abundant in the six libraries (Supplementary Fig. S2). Among the clean reads, we detected 46 predicted novel miRNAs (Supplementary Table S2) and 529 conserved miRNAs belonging to 96 miRNA families (Supplementary Table S3). The conserved miRNAs mapped to a large proportion of the miRNA precursors from other fish species listed in miRBase 21.0, including *Salmo salar* (ssa), *Ictalurus punctatus* (ipu), *Fugu rubripes* (fru), *Tetraodon nigroviridis* (tni), Zebrafish (dre), and *Oryzias latipes* (ola) (Fig. 2).

**Figure 3:**
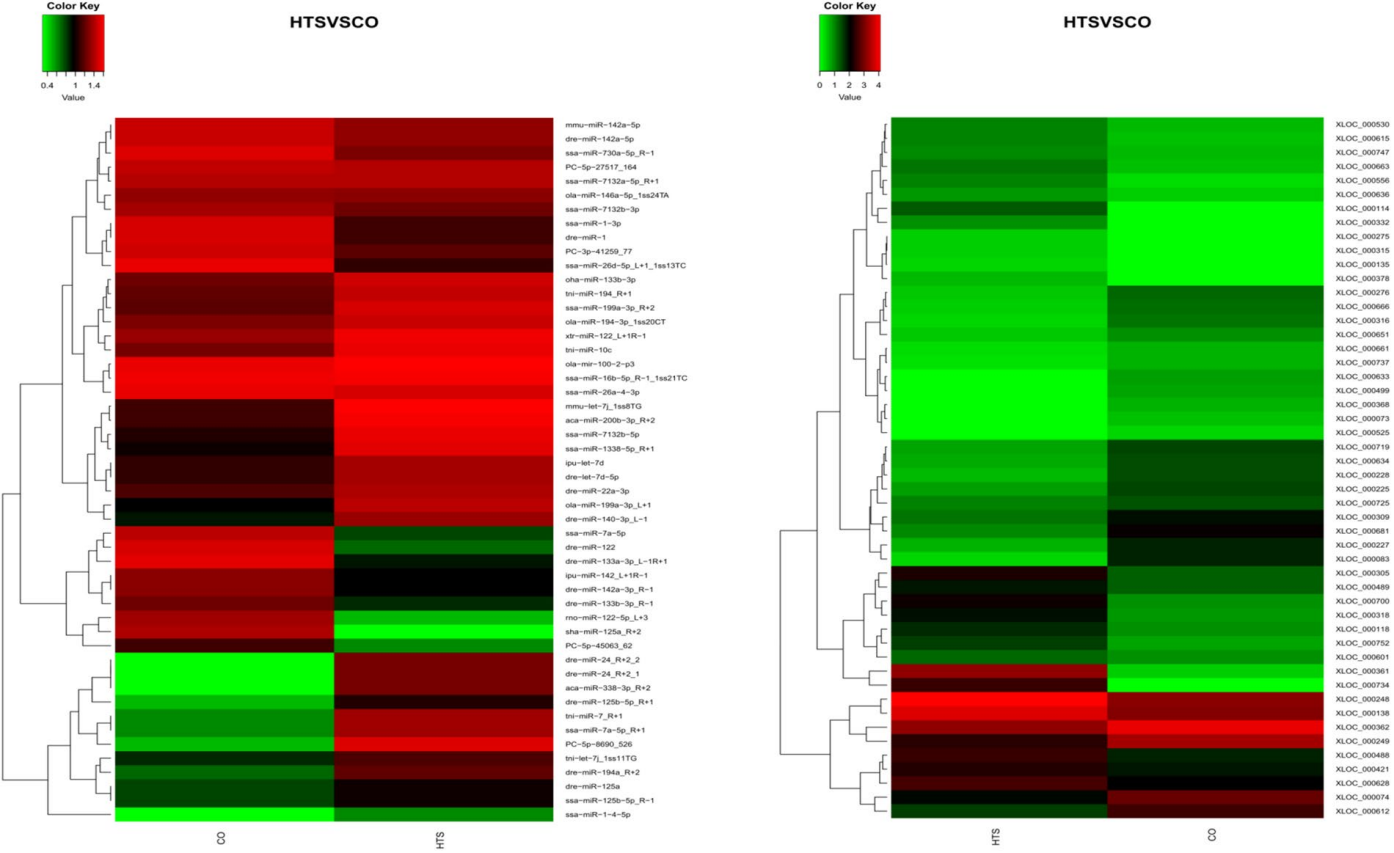
Hierarchical clustering of differentially expressed (DE) miRNAs and DE mRNAs between control (CO) and 37.5 °C heat-treated (HTS) groups. All the DE miRNAs are shown in the miRNA heat map, but only the top 60 DE mRNAs are shown in the mRNA heat map.

A total of 50 DE miRNAs were found between the CO and HTS libraries (Supplementary Table S4); among them, 28 DE miRNAs had *P* < 0.05, fold-change ≥ 1.5 or ≤0.67, and reads per million reads (RPM) ≥5. Among the 28 DE miRNAs, 11 were significantly down-regulated and 17 were significantly up-regulated, in the HTS libraries compared with the CO libraries. The DE miRNAs were divided into five biological processes, as shown in the heat map in Fig. 3.

### Expression profiling and annotation of mRNAs in heat-stressed GIFT liver

We built and sequenced six mRNA libraries, three from the 28 °C control groups (CO-1, CO-2, and CO-3) and three from the 37.5 °C-treated groups (HTS-1, HTS-2, and HTS-3). The biological replicates had good repeatability (Supplementary Fig. S3). An overview of the reads and quality filtering of the biological replicates are shown in Supplementary Table S5. After removing the low quality raw reads, 40,826,712, 48,116,524, 42,428,160, 40,083,434, 43,120,542, and 50,461,524 clean reads remained in the CO-1, CO-2, CO-3, HTS-1, HTS-2, and HTS-3 libraries respectively. Among them, the 25,941,213, 29,535,094, 26,308,819, 25,838,466, 26,844,109, and 33,704,213 reads that mapped to the Nile tilapia genome were analysed (Supplementary Table S6). Significantly more reads mapped to exon regions than to intron and intergenic regions of the genome (Supplementary Table S7).

**Figure 4:**
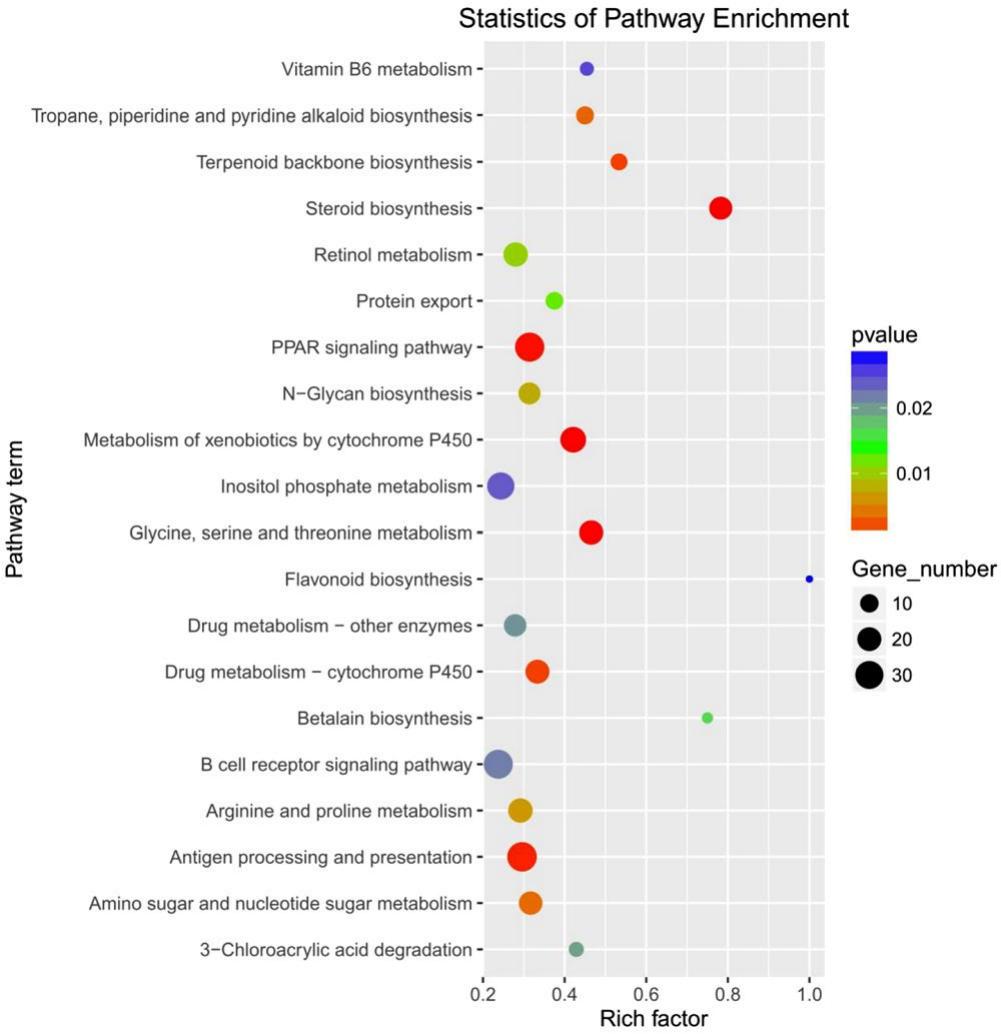
KEGG pathway enrichment analysis of the differentially expressed mRNAs from the liver of GIFT exposed to 37.5 °C heat-stress.

A total of 6685 DE mRNAs were detected between the CO and HTS libraries (Supplementary Table S8). These DE mRNAs were classified into five biological processes as shown in the heat map in Fig. 3. The KEGG pathway analysis of the DE mRNAs identified 20 pathways that were enriched by heat stress (*P* < 0.05) (Fig. 4). We identified 744 DE mRNAs that had *P* < 0.05, fold-change ≥2 or ≤0.5, and FPKM ≥10; among them, 202 and 240 known genes were up-regulated and down-regulated, respectively, in the HTS libraries compared with the CO libraries. Of the remaining mRNAs, 62 were unknown genes and 240 were novel genes. The significantly enriched pathways (with ≥5DE mRNAs) included organism system (steroid biosynthesis, cytochrome P450); metabolism (glycine, serine, and threonine metabolism, insulin signalling pathway, PPAR signalling pathway, retinol metabolism, fatty acid metabolism, glycolysis/gluconeogenesis, glutathione metabolism); and immune regulation (antigen processing and presentation, Toll-like receptor signalling pathway, complement and coagulation cascades, leukocyte transendothelial migration, and pathways in cancer) (Table 2). These results suggest that genes involved in organism system, metabolism, and immune regulation pathways may play important roles in the heat-stress response of GIFT.

**Table 2:** Significantly enriched pathways involving differentially expressed genes in heat-stressed GIFT liver.

Pathway name	Enriched genes
Steroid biosynthesis	*Cel.2, sigmar1, msmo1, tm7sf2, faxdc2, sc5d*
Peroxisome	*IDH2, agxtd, acox1, hacl1, ephx2, pipox*
Cytochrome P450	*mgst1.1,adh5, gstt1a,mgst3b, cyp3a65,gstt1b*,
Glycine, serine and threonine metabolism	*Agxtb, shmt1, alas2, gamt, bhmt, GLDC, tdh, cbsb, pipox, dmgdh, ENSONIG00000007379*
Insulin signaling pathway	*ACACA, PPP1R3b, FASN, PRKC2, PCK2*
PPAR signaling pathway	*LPL, FADS2, FABP1B.1, ACOX1, CYP4T8, SI:DKEY-9LI10.3, RBP2a,PCK2*
Retinol metabolism	*aldh8a1, retsat, zgc:64106, adh5, ENSONIG00000019488, dgat2, ENSONIG00000019489, cyp4t8, cyp3a65,ENSONIG00000015663*,
Fatty acid metabolism	*ECHS1, ADH5, ALDH9a1a.1, ENSONIG00000019488, ENSONIG00000019489,aldh3a2b, acox1, CYP4T8*
glycolysis/gluconeogenesis	*ADH5, ALDH9a1a.1, ENSONIG00000019488, ENSONIG00000019489,aldh3a2b,PCK2*
Glutathione metabolism	*IDH2, gpx4a, gpx1b, mgst1.1 ENSONIG00000009676, ENSONIG00000019188, gstt1a, mgst3b, G6PD, si:dkey-40m6.14, gstt2, gstt1b*
Antigen processing and presentation	*IFI30, CALR3b, CALR3a, CTSS, PSME1, ENSONIG00000013056*
Toll-like receptor signaling pathway	*TMED3, NFKBIAa, MAP2K4a, NFKBIAb, LRRC58b*
Complement and coagulation cascades	*dab1a, ENSONIG00000014877, f9a,ENSONIG00000012952, ENSONIG00000004728, si:ch1073-280e3.1, shbg, C3, ENSONIG00000014773,ENSONIG00000008983, masp1*
leukocyte transendothelial migration	*CYBA, ACTR3, CLDN2, RAC2, ENSONIG00000003145*
Pathways in cancer	*NFKBIAa, HSP90b1, NFKBIAb, EHLN2, FAM13a, RAC2, LAMC1*

### Integrated analysis of miRNA and mRNA expression profiles

**Figure 5:**
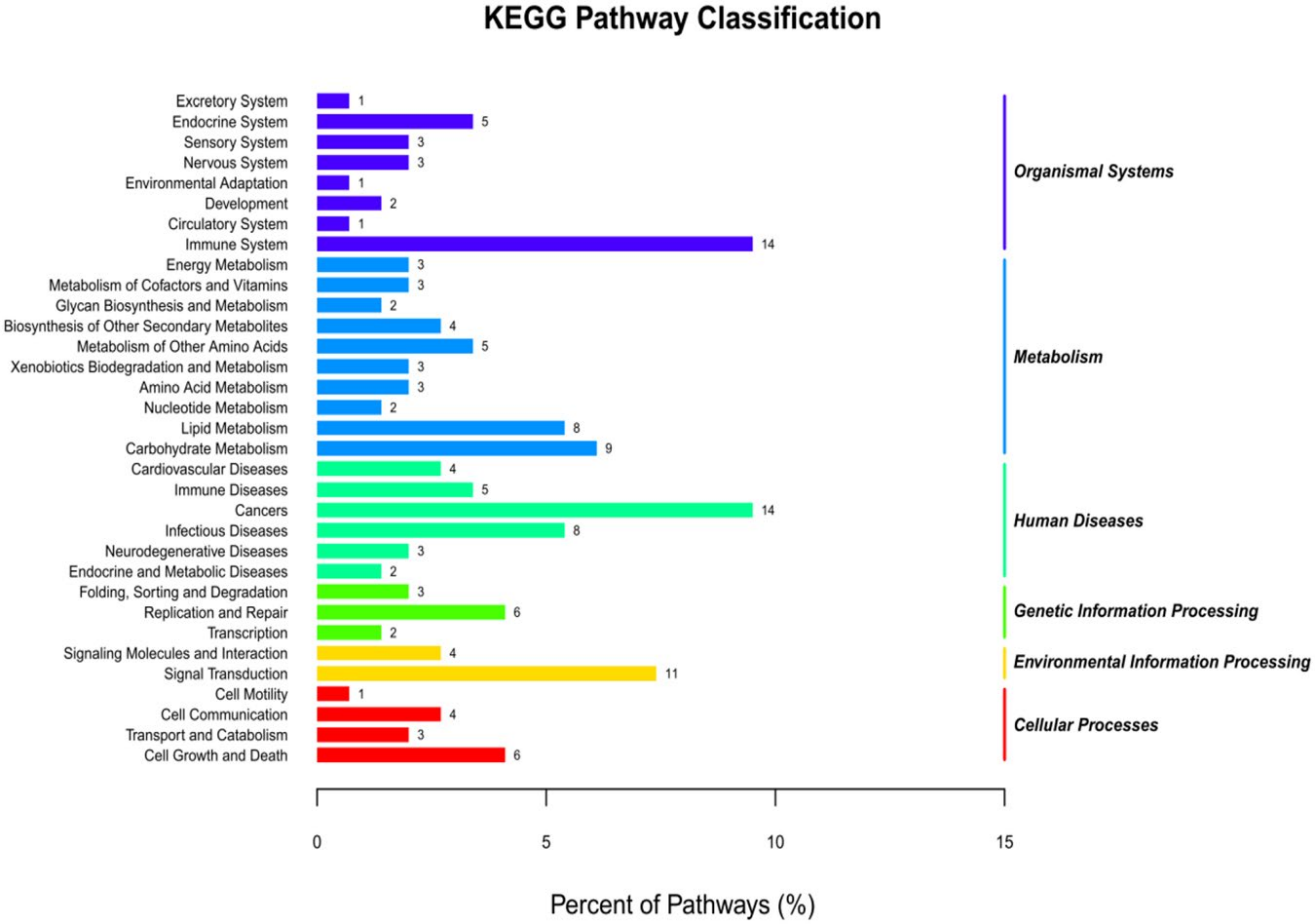
Classification of the enriched KEGG pathways for the negatively correlated miRNA–mRNA pairs of GIFT exposed to heat stress. Negative correlation is defined as having opposite expression patterns between miRNAs and their target mRNAs. The 1525 negatively correlated miRNA–mRNA pairs were associated with 33 KEGG pathways.

**Figure 6:**
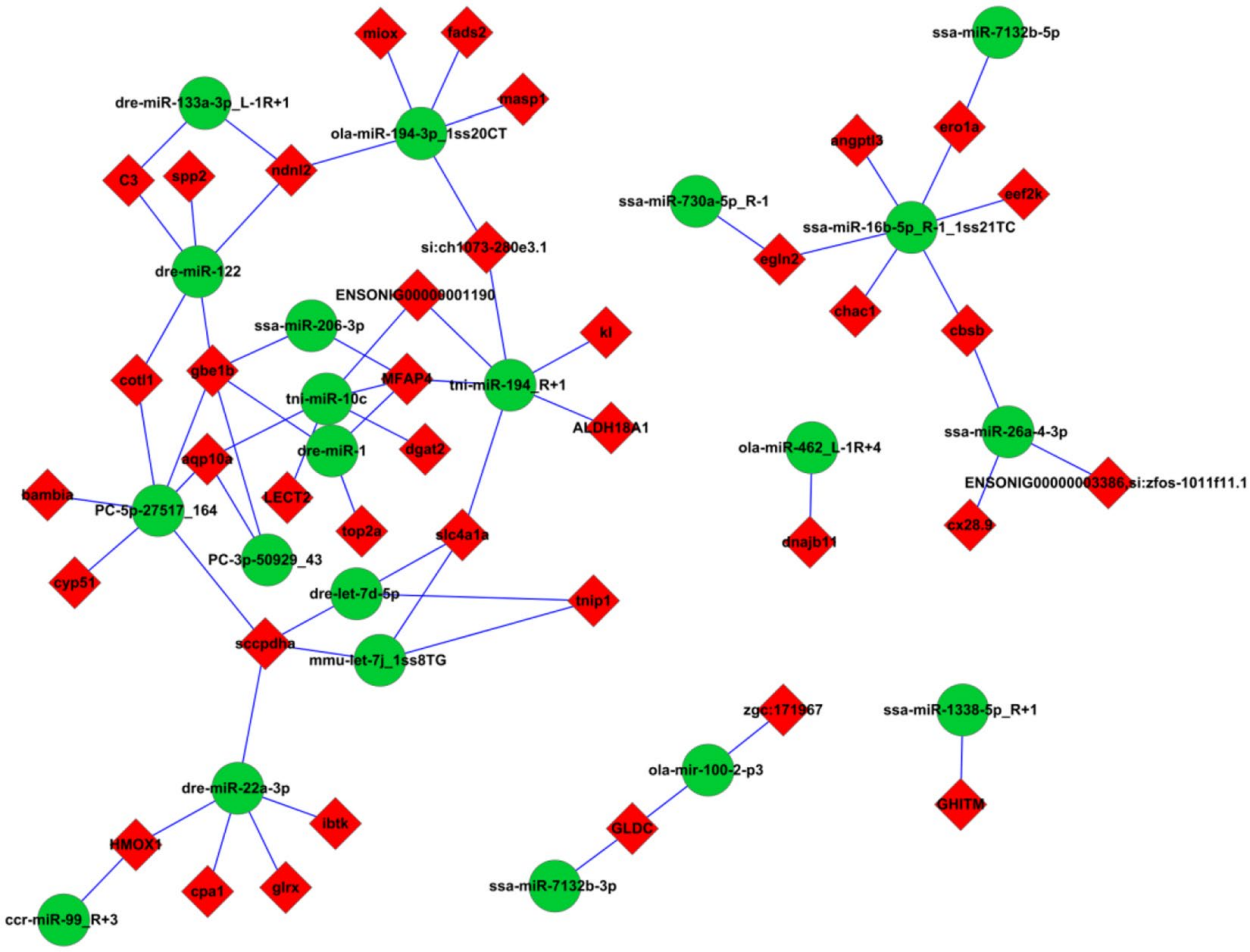
Analysis of the miRNA–mRNA negative correlation network. The network contains 64 negatively correlated miRNA–mRNA interactions (38 DE target mRNAs for 21 DE miRNAs) and was constructed by Cytoscape software.

A total of 2995 target genes were predicted for the 50 DE miRNAs using TargetScan and miRanda, and both positive and negative correlations were identified for the resultant miRNA–mRNA pairs (Supplementary Table S9). Negative correlations between the expression patterns of miRNAs and their target mRNAs are commonly found in animals^20^. In this study, 1525 of the miRNA–mRNA pairs (involving 50 DE miRNAs and 874 DE mRNAs) had a negative correlation of their expression patterns (Supplementary Table S10). The 1525 negatively correlated miRNA–mRNA pairs were associated with 33 KEGG pathways, as shown in Fig. 5. Among these pathways, organism system, metabolism, and diseases were the three main subclasses, which included sensory system, endocrine system, lipid metabolism, carbohydrate metabolism, immune system, cancers, and infectious diseases. We selected 64 negatively correlated miRNA–mRNA interactions (38 DE target mRNAs for 21 DE miRNAs) for screening based on the following identification: DE miRNAs with *P* < 0.05, fold-change ≥1.5 or ≤0.67, and RPM ≥ 5; and DE mRNAs with *P* < 0.05, fold-change ≥ 2 or ≤0.5 and FPKM ≥ 10.The correlation network for the 64 negatively correlated miRNA–mRNA interactions is showed in Fig. 6.

### Validation of selected DE miRNAs and DE mRNAs by qRT-PCR

**Figure 7:**
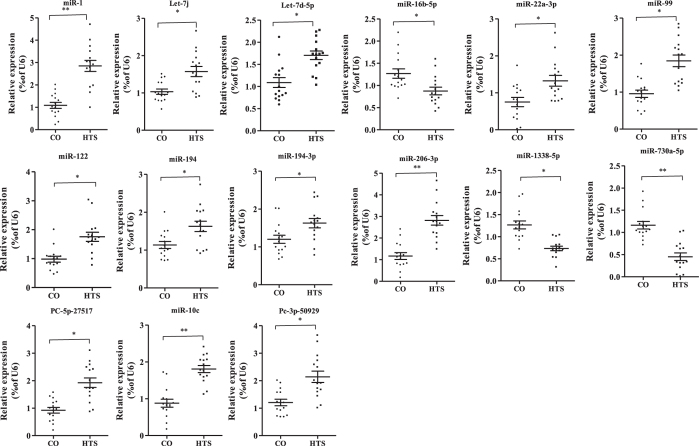
Validation of 15 differentially expressed (DE) miRNAs between control (CO) and 37.5 °C heat-treated (HTS) groups by qRT-PCR (n = 15 replicates per group). The values are expressed as the relative ratio with U6 as an internal control. *P < 0.05 and **P < 0.01, by unpaired Student’s T-tests.

**Figure 8:**
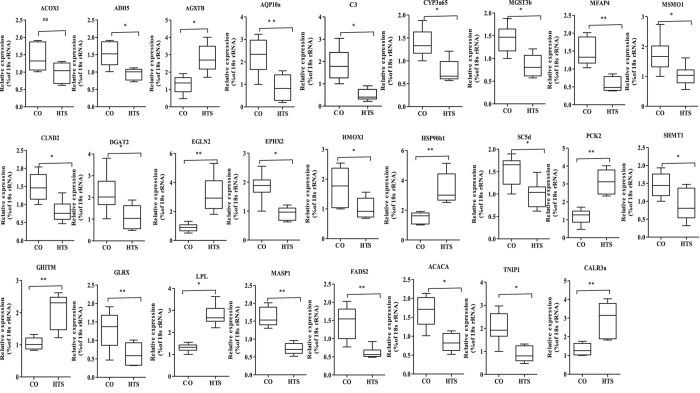
Validation of 26 differentially expressed (DE) mRNAs between control (CO) and 37.5 °C heat-treated (HTS) groups by qRT-PCR (n = 15 replicates per group). The expression levels of mRNA were normalized to that of 18 s rRNA as an internal control. *P < 0.05 and **P < 0.01, NS, no significant difference, by unpaired Student’s T-tests.

We selected 15 DE miRNAs (Table 3) and 26 DE mRNAs (Table 4) from the miRNA and mRNA libraries and verified them by qRT-PCR. They included the miRNAs and mRNAs in the following negative miRNA–mRNA pairs: miR-1/194/miR-206–3p–*MFAP4*, miR-1338-5p–*GHITM*, miR-16b-5p/730a-5p–*EGLN2*, miR-22a-3p–*GLRX*, miR-122–*C3*, let-7j/7d-5p–*TNIP1*, PC-5p-27517/3p-50929–*AQP10a*,miR-99–*HMOX1*, miR-194-3p–*FADS2*, and miR-10c–*DGAT2* (Fig. 7), together with 16 other DE mRNAs (*MSMO1*, *SC5d*,*EPHX2*, *MGST3b*, *CYP3a65*, *LPL*, *AGXTB*, *PCK2*, *SHMT1*, *ACOX1*, *ADH5*, *ACACA*, *HSP90b1*, *MASP1*, *CLDN2*, and *CALR3a*) (Fig. 8). We found that the expression patterns for all the selected miRNAs and mRNAs, except *ACOX1*, were consistent between the deep sequencing data and the qRT-PCR analysis.

**Table 3:** The 15 differentially expressed miRNAs in heat-stressed GIFT liver verified by miRNA-Seq.

MiR_name	MiR_seq	Log2 (fold_change)	Regulation (HTS vs CO)
dre-miR-1	TGGAATGTAAAGAAGTATGTAT	4.38	up
tni-miR-194_R + 1	TGTAACAGCAACTCCATGTGGA	0.35	up
ssa-miR-206-3p	TGGAATGTAAGGAAGTGTGTGG	4.89	up
ssa-miR-1338-5p_R + 1	AGGACTGTCCAACCTGAGAATG	−1.60	down
ssa-miR-16b-5p_R-1_1ss21TC	TAGCAGCACGTAAATATTGGC	−0.50	down
ssa-miR-730a-5p_R-1	TCCTCATTGTGCATGCTGTGT	−1.89	down
dre-miR-22a-3p	AAGCTGCCAGCTGAAGAACTGT	0.39	up
dre-miR-122	TGGAGTGTGACAATGGTGTTTG	0.48	up
tni-let-7j_1ss11TG	TGAGGTAGTTGTTTGTACAGTT	0.50	up
dre-let-7d-5p	TGAGGTAGTTGGTTGTATGGTT	0.57	up
PC-5p-27517_164	TACATGCAGAGGTGGAGCAAGA	2.31	up
PC-3p-50929_43	TGGAAGTGTCAGAAATTCTGAGT	1.80	up
ccr-miR-99_R + 3	AACCCGTAGATCCGATCTTGTGAA	2.28	up
ola-miR-194-3p_1ss20CT	CCAGTGGAGGTGCTGTTACTTG	0.19	up
tni-miR-10c	TACCCTGTAGATCCGGATTTGT	1.59	up

Fold change = HTS group (mean)/CO group (mean), where “mean” is the mean of three biological replicates.

**Table 4:** The 26 differentially expressed mRNAs in heat-stressed GIFT liver verified by mRNA-Seq.

Gene abbreviation	Gene description	Log2 (fold_change)	Regulation (HTS vs CO)
MFAP4	Microfibrillar associated protein 4	−1.83	down
GHITM	Growth hormone inducible transmembrane protein	2.41	up
EGLN2	Egl nine homolog 2	2.29	up
GLRX	Glutaredoxin	−1.84	down
C3	Complement C3	−1.39	down
TNIP1	TNFAIP3 interacting protein 1	−1.32	down
AQP10a	Aquaporin-10a	−2.17	down
HMOX1	Heme oxygenase 1	−1.72	down
FADS2	fatty acid desaturase-6	−3.42	down
DGAT2	Diacylglycerol O-Acyltransferase 2	−1.57	down
MSMO1	Methylsterol monooxygenase 1	−1.27	down
SC5d	Sterol-C5-desaturase	−2.56	down
EPHX2	Epoxide hydrolase 2	−1.08	down
MGST3b	Microsomal glutathione S-transferase 3b	−1.66	down
CYP3a65	Cytochrome P450, family 3, subfamily A, polypeptide 65	−1.02	down
LPL	Lipoprotein lipase	2.56	up
AGXTB	Alanine-glyoxylate aminotransferase b	2.01	up
PCK2	Phosphoenolpyruvate carboxykinase 2	2.52	up
SHMT1	Serine hydroxymethyltransferase 1	−1.19	down
ACOX1	Acyl-CoA oxidase 1	−1.03	down
ADH5	Alcohol dehydrogenase 5	−1.68	down
ACACA	Acetyl-CoA carboxylase	−2.28	down
HSP90b1	Heat shock protein 90 beta family member 1	2.86	up
MASP1	Mannan-binding lectin serine protease 1	−1.96	down
CLDN2	Claudin 2	−1.63	down
CALR3a	Calreticulin 3a	2.92	up

Fold change = HTS group (mean)/CO group (mean), where “mean” is the mean of three biological replicates.

## Discussion

We studied the early response in the liver of GIFT exposed to heat stress by deep sequencing CO and HTS libraries. A total of 63.34%, 61.38%, 62.01%, 64.46%, 62.25%, and 66.7% of the valid reads in the CO-1, CO-2, CO-3, HTS-1, HTS-2, and HTS-3 libraries, respectively, mapped to the reference Nile tilapia genome. The somewhat low percentage of mapped reads may be attributed to the specificity of gene expression in different tissues and/or some of the unmapped reads may represent incompletely sequenced regions of the reference Nile tilapia genome^21^. We screened 15 and 26 DE miRNAs and mRNAs, respectively, involved in the GIFT response to heat stress and verified them by qRT-PCR.

In the KEGG pathway analysis, organism system, metabolism, and immune regulation were highly enriched among the DE mRNAs. Steroid biosynthesis and cytochrome P450 can reduce endocrine-disrupting chemicals and increase oxygen-mediated sensitivity to protect respiratory epithelial cells against oxygen-induced toxicity^22, 23^. Disruption of steroid biosynthesis can result in impaired growth, development and reproduction, and the development of certain cancers. Steroid hormone biosynthesis is controlled by the activity of several highly substrate-selective cytochrome P450 enzymes and a number of steroid dehydrogenases and reductases^22^. In this study, we identified five DE genes (*MSMO1*, *SC5d*, *EPHX2*, *MGST3b*, and *CYP3a65*) between the CO and HTS libraries that were annotated as regulation of organism system. *SC5d* and *MSMO1* encode enzymes involved in cholesterol biosynthesis^24, 25^. *EPHX2* is usually involved in increasing susceptibility to ischemic stroke in patient, and may be associated with familial hypercholesterolaemia^26, 27^. Inhibition of *SC5d*, *MSMO1*, and *EPHX2* may reduce the synthesis of steroid hormones from cholesterol, and help GIFT cope with heat stress. *CYP3a* is the most abundant cytochrome P450 enzyme in the liver of animals, and is involved in the biotransformation of most drugs and environmental factors^28^. *CYP3a65* of zebrafish is an ortholog of *CYP3a* and is expressed mainly in the liver and intestine^29^. *MGST3b* also has an important role in the biotransformation of xenobiotics^30^. Down-regulation of *CYP3a65* and *MGST3b* in GIFT liver 24 h post-heat stress, suggests that the detoxification of xenobiotic substances may have been impaired^28^.

When fish are exposed to water temperatures higher than their thermoneutral temperature, internal body heat is dissipated by elevated metabolic rate and peripheral blood flow, which are known to increase the production and transportation of reactive oxygen species that react with lipids, proteins, and nucleic acids^13^. Some of the DE genes in GIFT liver in response to heat stress were predicted to be involved in glycine, serine, and threonine metabolism, PPAR signalling pathway, fatty acid metabolism, and glycolysis/gluconeogenesis, all of which are involved mainly in the decomposition and utilization of amino acids, fatty acids, and glycogen in liver. For instance, *PCK2* expression and activity were enhanced under low-glucose conditions^31^, and increased usage of hepatic glycogen occurred under stress. Elevated *LPL* expression contributed to the conversion of triglycerides to fatty acids in the liver to supply energy demand^32^. Up-regulation of *LPL*, *AGXTB*, and *PCK2* expression and down-regulation of *SHMT1* and *ADH5* expression were detected in this study. Therefore, heat stress may have stimulated an increase of glucose and lipid metabolism and repressed fatty acid *β*-oxidation, which may help to maintain homeostasis in GIFT liver exposed to heat stress through the regulation of various pathways^33^. The insulin signalling pathway was also enriched, and insulin is the major hormone that controls critical energy functions such as glucose and lipid metabolism. In many teleost fish, glucose stimulates insulin release either *in vitro* or *in vivo* after intraperitoneal administration^34^. The expression of *ACACA* was significantly down-regulated, suggesting that this enzyme may be involved in the response of lipid and glucose metabolism to heat stress^35^. Some DE genes were enriched in retinol metabolism and glutathione metabolism, implying that GIFT may use retinol and glutathione products to improve antioxidant and integrated detoxification^36^.

Immune regulation plays an important role in the stress response of fish, and involves mainly antigen processing and presentation, the Toll-like receptor signalling pathway, complement and coagulation cascades, leukocyte transendothelial migration, and pathways associated with cancer. *HSP90b1* plays an important role in tumour cell growth and promotes protein refolding^37^. Overexpression of *HSP90b1* was implicated in poor survival of patients with hepatocellular carcinoma^38^. Although *MASP1* is not directly involved in complement activation, it may play a role as an amplifier of complement activation^39^. Our results strongly suggest that *HSP90b1* was up-regulated, and *MASP1*were down-regulated in GIFT liver in response to heat stress. *CLDN2*, a “leak” protein that forms a tight junction with the vitamin D receptor, mediates paracellular water transport in epithelia. Down-regulation of *CLDN2* in GIFT liver may increase WBC numbers in blood, which would help to reduce stress injury^40^. Calcium-binding chaperone promotes folding, oligomeric assembly, and quality control in the endoplasmic reticulum via the calreticulin (CALR)/calnexin cycle, and it has been suggested that up-regulated *CALR3a* may participate in the stress response by regulating calcium homeostasis^41^.

In this study, we analysed miRNA profiles in control and heat-stressed GIFT liver samples at 24 h post-treatment by 48-h LT_50_. The 22-nt long miRNAs were the most abundant in the six libraries, which is in agreement with other aquatic animals, including *Megalobrama amblycephala* ^42^ *, Apostichopus japonicus* ^43^, and *Gobiocypris rarus* ^44^. We screened and verified 15 DE miRNAs and their negatively regulated target genes from the liver of GIFT under heat stress. Among these miRNAs, miR-1, miR-206-3p, miR-194, and miR-122 were predicted to be involved in immune response and disease pathways. Previous studies have found that miR-1 and miR-194 played vital roles in human cancers^45–47^. In this study, a target gene of miR-1/194/206-3p was *MFAP4*, which was recently identified as a biomarker for hepatic fibrosis and has been used to detect high-risk patients with severe fibrosis stages among hepatitis C patients^48^. Up-regulated miR-1, miR-206-3p, and miR-194 may regulate cell growth and stress response, and relieve cell damage in GIFT liver by inhibiting *MFAP4* expression levels. MiR-122 was enriched mainly in liver tissue, accounting for 70% and 52% of the miRNAs in adult mouse and human liver, respectively, and has been identified as a key factor and therapeutic target in liver disease^49^. In our study, miR-122 was predicted to target the gene coding complement *C3*, the central component of immune system. Up-regulation of miR-122 may inhibit complement *C3* expression, suggesting miR-122 may be involved in immune regulation in heat-stressed GIFT liver.

Under environmental stress, oxidative damage in fish can cause free radical metabolic disorders and lipid metabolic abnormalities. Therefore, the liver of GIFT may be involved in “lipid and carbohydrate metabolic process”, and up-regulated miR-194-3p, miR-22a-3p, and miR-10c may be involved in the adaptive regulation of metabolic levels post-heat stress. *FADS1* and *FADS2* play important roles in polyunsaturated fatty acid metabolism^50^. Suppression of *FADS2* gene expression inhibited the first step in the enzymatic cascade of polyunsaturated fatty acid synthesis in mouse^51^. Desaturation of fatty acids helps to maintain fluidity and lipid homeostasis in cell membranes, which can compensate for the rigidification of lipids in cells exposed to temperature stress^5, 52^. Significantly increased miR-194-3p levels were found in our study and its target, *FADS2*, was inhibited, suggesting an impaired cell membrane in response to heat stress in GIFT. Elevated hepatic miR-22-3p expression in mouse models of insulin resistance and type 2 diabetes impaired gluconeogenesis and reduced hepatic glucose output^53^. The target gene of miR-22a-3p, *GLRX*, codes a small thioltransferase that can remove protein glutathione adducts without having direct antioxidant properties. *GLRX*-deficient mice showed up-regulation of *SREBP-1* and key hepatic enzymes involved in lipid synthesis by inhibition of *SirT1* activity^54^. MiR-10c was implicated in the response to nutrient restriction and refeeding in skeletal muscle of Chinese perch, *Siniperca chuatsi* ^55^. MiR-10c expression significantly increased in fast muscle 1 h after refeeding, which may be related to amino acid and lipid utilization. Up-regulated miR-10c may regulate the target gene *DGAT2*, which catalyses the final step in triglyceride synthesis. In rats, treatment with *DGAT2* antisense oligonucleotides significantly reduced hepatic lipids and improved hepatic insulin sensitivity^56^. In our study, miR-22a-3p and miR-10c were significantly up-regulated and their target genes *GLRX* and *DGAT2* were significantly down-regulated, which suggested that the miR-22a-3p–*GLRX* and miR-10c–*DGAT2* pairs played vital roles in lipid metabolism, likely by increasing the transformation and utilization of energy substances to maintain energy homeostasis in heat-stressed GIFT.

The let-7 and miR-99 families are essential in regulating cell proliferation and development of organism systems. The sequence and functions of mature let-7 are highly conserved among animal species^57^. Let-7 was found to be widely involved in tissue development and metabolism during Japanese flounder (*Paralichthys olivaceus*) development, and mediated metamorphosis by cell proliferation and differentation^58^. Up-regulation of let-7 was reported to stimulate cell proliferation and reduce the development of cell-based disease^59^. Let-7j and let-7d-5p regulate the target gene *TNIP1*, which may be involved in NF-κB and nuclear receptor signalling and contribute to regulate key players of cell growth and differentiation in therapies^60^. Down-regulation of *TNIP1* by let-7j and let-7d-5p, which were up-regulation in GIFT under heat stress, may affect cell development and relieve the occurrence of cell disease. MiR-99a and miR-99b were highly expressed in mouse haematopoietic stem cells compared with their more differentiated progeny, and played important roles in the regulation of the haematopoietic system^61^. Therefore, up-regulation of miR-99 in response to heat stress may be related to adaptive regulation in GIFT liver by the repressed target gene *HMOX1*. In milk somatic cells of lactating yaks, miR-16b expression levels were found to be up-regulated and then decreased from early lactation to the colostrum period^62^. *EGLN2*, the predicted target gene of miR-16b-3p from our study, is involved in regulating cell growth, hypoxia tolerance, and the neuron apoptotic process^63^. Down-regulated miR-16b-3p may help to activate *EGLN2* expression in liver, indicating cell differentiation and carrying oxygen adaptation may have been regulated in the GIFT response to heat stress.

Two novel miRNAs, PC-5p-27517/3p-50929, detected in our study, may mediate *AQP10a* expression. AQP10 belongs to the aquaglyceroporin family of integral membrane proteins that function as water-permeable channels in the epithelia of organs that absorb and excrete water^64^. Up-regulation of PC-5p-27517/3p-50929 in response to heat stress in GIFT may have inhibited expression of their target gene *AQP10a*, indicating that transformation of water and glycerine may be reduced, and cell membrane fluidity may be impaired in heat-stressed GIFT liver. The functions of miR-1338-5p/730a-5p have not been reported so far. In our study, down-regulated miR-730a-5p was predicted to target *EGLN2* and may have a similar as function of miR-16b-5p. *GHITM* is a transmembrane protein that is a distributed widely in plants and animals, and is involved in regulation of growth, development processes, and antioxidation in animals^65, 66^. *GHITM* was first detected among differentially expressed genes of transgenic mice^67^. Down-regulated miR-1338-5p may up-regulate *GHITM* expression in response to heat stress, suggesting the involvement of liver in regulated cell growth and oxidative stress.

## Conclusions

**Figure 9:**
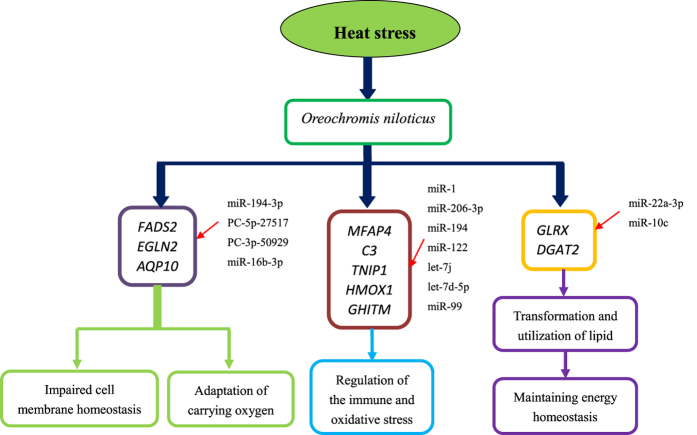
Diagram of regulated pathways in liver of GIFT exposed to 37.5 °C heat-stress for 24 h. The pathways involved mainly impaired cell membrane homeostasis, intervention in the immune response, stimulated lipid synthesis, decomposition, and utilization, and regulation of antioxidants and cell growth.

Deep sequencing-based expression profiling of miRNAs and mRNAs of liver isolated from GIFT exposed to heat stress for 24 h is reported in this study. A total of 28 DE miRNAs and 744 DE mRNAs were found and screened in the HTS libraries compared with the CO libraries. Fifteen negative miRNA–mRNA pairs involved in the heat-stress response were screened and verified, including miRNA–mRNA pairs related to organism system, metabolism, and disease pathways. In GIFT’s response to heat stress, we found various biological reactions of early response that may be impacted, including impaired cell membrane homeostasis, changes in the immune response, increased lipid synthesis, decomposition, and utilization, and regulation of antioxidants and cell growth (Fig. 9). Our findings build on and provide valuable network-based molecular regulation mechanisms for better functional characterization of miRNA–mRNA interactions in response to heat stress in GIFT. Further analysis of the miRNA–mRNA network is required in other tissues and at the cellular level under different stresses and at different time points, which will help to better understand molecular-regulated pathways that respond to heat stress, and to better prevent and treat fish damage caused by high-temperature stress.

## Materials and Methods

### Ethics approval

All the methods used in this study were performed in accordance with the Guidelines for Experimental Animals established by the Ministry of Science and Technology (Beijing, China). The study protocols were approved by the Freshwater Fisheries Research Centre of the Chinese Academy of Fishery Sciences (Wuxi, China). Fish were killed with an overdose of tricaine methanesulfonate (100 mg L^−1^ MS-222; Argent Chemical Laboratories, Redmond, WA, USA) within 1 min after capture and the liver tissue was extracted based on the Guide for the Care and Use of Laboratory Animals in China.

### GIFT materials and growth conditions

The GIFT used in this study were 16^th^ generation. The experimental fish all had the same genetic background and were offspring of the same family. They were obtained from the Yixing Tilapia Farm at the Freshwater Fisheries Research Centre of the Chinese Academy of Fishery Sciences (Wuxi, China). The same batch of breeding fry were selected and cultured in an outdoor cement pond for 50d, and then transported into an indoor water circulation system. The fish were acclimatized in indoor concrete tanks containing 28 °C water under a natural photoperiod with continuous aeration for 2 weeks prior to the experiment. The fish were conditioned to accept floating pellet feed (NingboTech-Bank Co., Ltd, Yuyao, China; crude protein 28%, crude lipid 6%). The average weight of the experimental GIFT was 105 ± 5 g. A total of 540 experimental fish were selected for the experiment, and the fish were fasted for 24 h before the experiment.

### Treatment and sampling

First, the 48-h LT_50_ of the GIFT was determined by observing the fish at eight temperatures: 35.5, 36, 36.5, 37, 37.5, 38, 38.5, or 39 °C. The experimental temperatures were reached in 30 min using heaters. Eight treatments with three replicates, each with 10 fish, were set up. The cumulative mortality of each treatment group within 48 h was counted and the 48-h LT_50_ of the GIFT was obtained by linear interpolation. The 48-h LT_50_ was determined as 37.49 °C; therefore, we chose 37.5 °C as the heat-stress temperature for the present study.

The 300 GIFT were stocked into six 900-L tanks (50 fish per tank) at an initial temperature of 28 °C. The control GIFT livers (CO-1, CO-2, and CO-3) were obtained from three tanks at 28 °C. Livers were immediately removed and stored in liquid nitrogen. The temperature of the three treatment tanks was rapidly increased to 37.5 °C using heaters. The liver tissues of the heat-stressed groups (HTS-1, HTS-2, and HTS-3) were sampled at 24 h of stress and stored in liquid nitrogen. Thus, three biological replicates of sampled livers were obtained for the CO and HTS groups. The liver tissues of three fish in each tank were mixed and pooled to construct one library, and six libraries (CO-1, CO-2, CO-3, HTS-1, HTS-2, and HTS-3) were built. Another five livers were obtained from fish selected randomly from each tank of the CO or HTS groups at 24 h. These livers were used in the qRT-PCR analysis to verify the miRNA and mRNA expression levels. Blood samples from three fish per tank were obtained at each of the following time periods post-heat stress: 0, 6, 12, 24, 36, and 48 h for WBC and RBC counts^68^, and determining lysozyme activity^9^.

### Small RNA library construction and sequencing

Total RNA was extracted from the liver samples using Trizol reagent (Invitrogen, CA,USA) according to the manufacturer’s instructions. ARNA 6000 Nano LabChip Kit and Bioanalyzer 2100 (Agilent, CA,USA) were used to detect the quantity and purity of the extracted RNA. If the RNA integrity number was >7.0, the samples were used for further processing. About 1 µg total RNA from each sample was employed to construct the small RNA libraries using TruSeq Small RNA Sample Prep Kits (Illumina, San Diego,USA) according to the manufacturer’s instructions. The small RNA libraries were sequenced on an Illumina HiSeq. 2500 platform by single-end sequencing (50 bp) and according to standard methods of the LC-BIO (Hangzhou, China)^20, 69^.

The basic sequencing data were analysed following the previous study by LC Sciences Service (Houston, TX, USA)^20, 69^. The known miRNAs and novel 3p- and 5p-derived miRNAs were identified by BLAST searches against specific species precursors in miRBase 21.0^20^. The mapping methods and identified miRNAs are listed in Supplementary Table S2. Modified RPM was employed to normalize the expression of the miRNAs.

### Analysis of DE miRNAs

Following normalized deep-sequencing counts, DE miRNAs were identified using Student’s T-tests. A threshold of <0.05 was considered significant in the analysis.

### Prediction of target genes and bioinformatic analysis

To predict the target genes of the DE miRNAs, we employed TargetScan 5.2 (http://www.targetscan.org/vert_50/) and the miRanda v3.3a toolbox (http://www.microrna.org/microrna/home.do) to analyse mRNAs for miRNA binding sites. The targets predicted by both algorithms were combined and overlaps were identified. The gene ontology (GO; http://www.geneontology.org) and KEGG pathway (http://www.genome.jp/kegg/pathway. html) databases were used to assign terms and pathways to the target genes to determine their potential downstream biological functions.

### mRNA library construction and sequencing

About 10 µg total RNA from each liver sample was used and poly (A) mRNA was isolated using poly(T) oligos attached to magnetic beads (Invitrogen, CA, USA). Six cDNA libraries were created by reverse-transcription (RT) based on the protocol for the mRNA-Seq Sample Preparation Kit (Illumina, San Diego, USA). We performed paired-end sequencing (300 ± 50 bp) on an Illumina HiSeq. 2500 according to standard methods (LC Sciences, Houston, USA). We aligned the reads obtained in the CO-1, CO-2, CO-3, HTS-1, HTS-2, and HTS-3 libraries to the *O.niloticus* (http://www.ncbi.nlm.nih.gov/genome/?term=Oreochromis%20niloticus) reference genome using the TopHat package. The alignment and annotation methods were based on those of the LC Sciences Service^20, 69^.

### Analysis of DE mRNAs

The aligned reads were analysed by Cufflinks, which uses normalized RNA-seq fragment counts to measure the relative abundances of transcripts^70^. The Fragment Per Kilobase of exon per Million fragments mapped (FPKM) was used as the unit of measurement. We used Student’s T-tests to analyse the DE mRNAs. The GO and KEGG pathway databases were used to assign terms and pathways to the DE genes to determine their potential downstream biological functions. Significantly enriched KEGG terms were determined using corrected *P*-values (*P* < 0.05)^71^.

### Integrated analysis of miRNA and mRNA expression profiles

We employed ACGT101-CORR1.1 to predict all possible positively and negatively correlated miRNA–mRNA pairs following the LC-BIO (Hangzhou, China) recommended protocol^72^. Based on the integrated analysis of DE miRNAs (*P* < 0.05, fold-change ≥ 1.5 or ≤0.67, and RPM ≥ 5) and DE mRNAs (*P* < 0.05, fold-change ≥ 2 or ≤0.5, and FPKM ≥ 10), and the principle of miRNA–mRNA pairs in animals, we selected the negatively correlated DE miRNA–mRNA pairs for screening. We used Cytoscape software (http://www.cytoscape.org/) to construct an interaction network of the screened pairs.

### Validation of miRNA and mRNA expression profiles

We employed qRT-PCR to validate 15 DE miRNAs (13 known and two novel miRNAs) and nine DE mRNAs from the screened miRNA–mRNA pairs, and 16 other DE mRNAs detected in the livers of heat-stressed GIFT.

Fifteen liver tissue samples from the CO or HTS groups were used to extract total RNA with Trizol reagent (Invitrogen, CA, USA). We used a Mir-X™ miRNA First-Strand Synthesis Kit (Takara, Dalian, China) to synthesize first-strand cDNA. The RT reaction was mixed and incubated according to Qiang *et al*.^68^. The qRT-PCRs were performed using a miRNA SYBR Green qRT-PCR Kit (Takara, Dalian, China) with the provided miRNA reference gene (U6). The 25 μL PCR mixture contained the RT product (template) 2.0 μL, 2× SYBR Advantage Premix 12.5 μL, ddH_2_O 9 μL, 50× ROX Dye 0.5 μL, miRNA-specific primer (10 μM) 0.5 μL, and mRQ 3′primer 0.5 μL. The default thermal profile used for the PCR amplifications was according to Qiang *et al*.^68^. Dissociation curve analysis of the amplified products was performed after each PCR reaction to confirm that only one PCR product was amplified and detected. For each cDNA, three-well replicates were used. The threshold cycle (Ct) value was determined using the automatic setting on the ABI 7900HT Fast Real-Time PCR system (Applied Biosystems, NY, USA). The Ct values determined for each sample were normalised against the values for U6. The relative fold changes in expression relative to U6 were calculated by the 2^−ΔΔCt^ method^72^. The miRNA specific primers (Supplementary Table S11) were synthesized by Genewiz, Inc. (Genewiz, Suzhou, China).

PrimeScript™ RT Master Mix and SYBR^®^ Premix Ex Taq kits (Takara, Dalian, China) were used for the RT reaction and qRT-PCRs of the mRNAs. The RT and PCR reaction methods followed those of our previous study^68^. The 18 S rRNA transcript level was taken as a reference. The primers (Supplementary Table S12) were synthesized by Shanghai GeneCore Bio Technologies Co., Ltd. (Shanghai, China). The mRNA expression levels were presented and analysed using the methods described above for the miRNAs. The mRNA expression levels were quantified using an ABI 7900HT Fast Real-Time PCR System (Applied Biosystems, NY, USA) and compared using Relative Quantification (RQ) manager software. We used Student’s T-tests to analyse the qRT-PCR expression results.

### Data Availability

The raw sequencing data generated and analysed in the current study areavailable in the Gene Expression Omnibus (GEO) repository under accession number (GSE94906) (https://www.ncbi.nlm.nih.gov/geo/query/acc.cgi?acc=GSE94906).

## Electronic supplementary material

Supplementary Information 
