# Supplementary material for: The expression profiles of miRNA–mRNA of early response in genetically improved farmed tilapia (Oreochromis niloticus) liver by acute heat stress
Source: Sci Rep. 2017 Aug 18;7:8705. doi: 10.1038/s41598-017-09264-4 (PMC5562739; doi:10.1038/s41598-017-09264-4)
Supplement: Supplementary file 1 — Supplementary Information [file 41598_2017_9264_MOESM1_ESM.pdf]

The expression profiles of miRNA-mRNA of early response in genetically improved farmed tilapia (*Oreochromis niloticus*) liver by acute heat stress

Jun Qiang[1*], Wen J. Bao[2], Fan Y. Tao[2], Jie He[1], Xia H. Li[1], Pao Xu[1*], Lan Y. Sun[1]

[1]Key Laboratory of Freshwater Fisheries and Germplasm Resources Utilization, Ministry of Agriculture, Freshwater Fisheries Research Centre, Chinese Academy of Fishery Sciences, 9 Shanshui East Road, Wuxi, Jiangsu 214081, China.

[2]Wuxi Fisheries College, Nanjing Agricultural University, 9 Shanshui East Road, Wuxi, Jiangsu 214081, China.[*]Correspondence and requests for materials should be addressed to P.X. (email: Xup@ffrc.cn)

Figure S1 The repeatability analysis of miRNA libraries between samples by using of pearson correlation

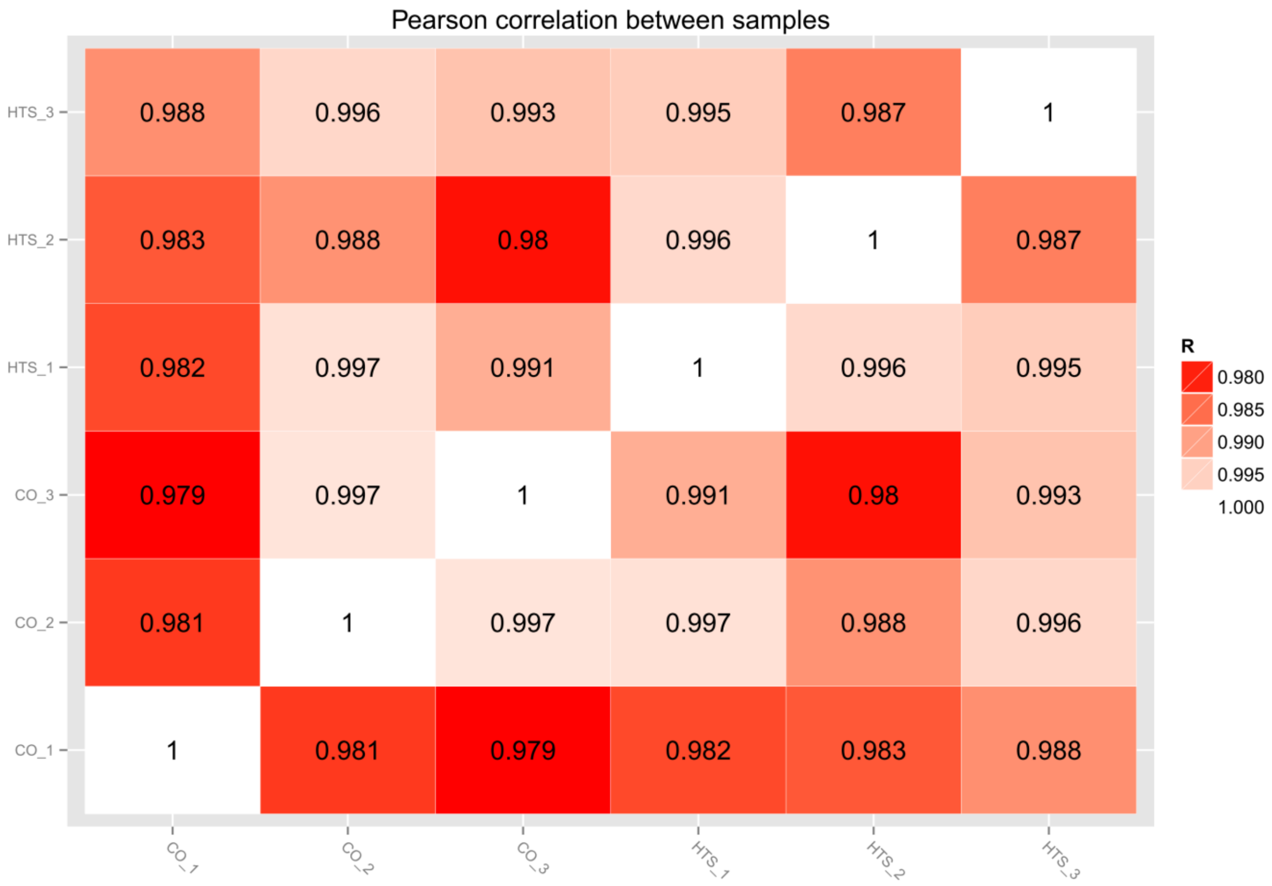

Figure S2 Distribution of the assembled genes and transcript length

.

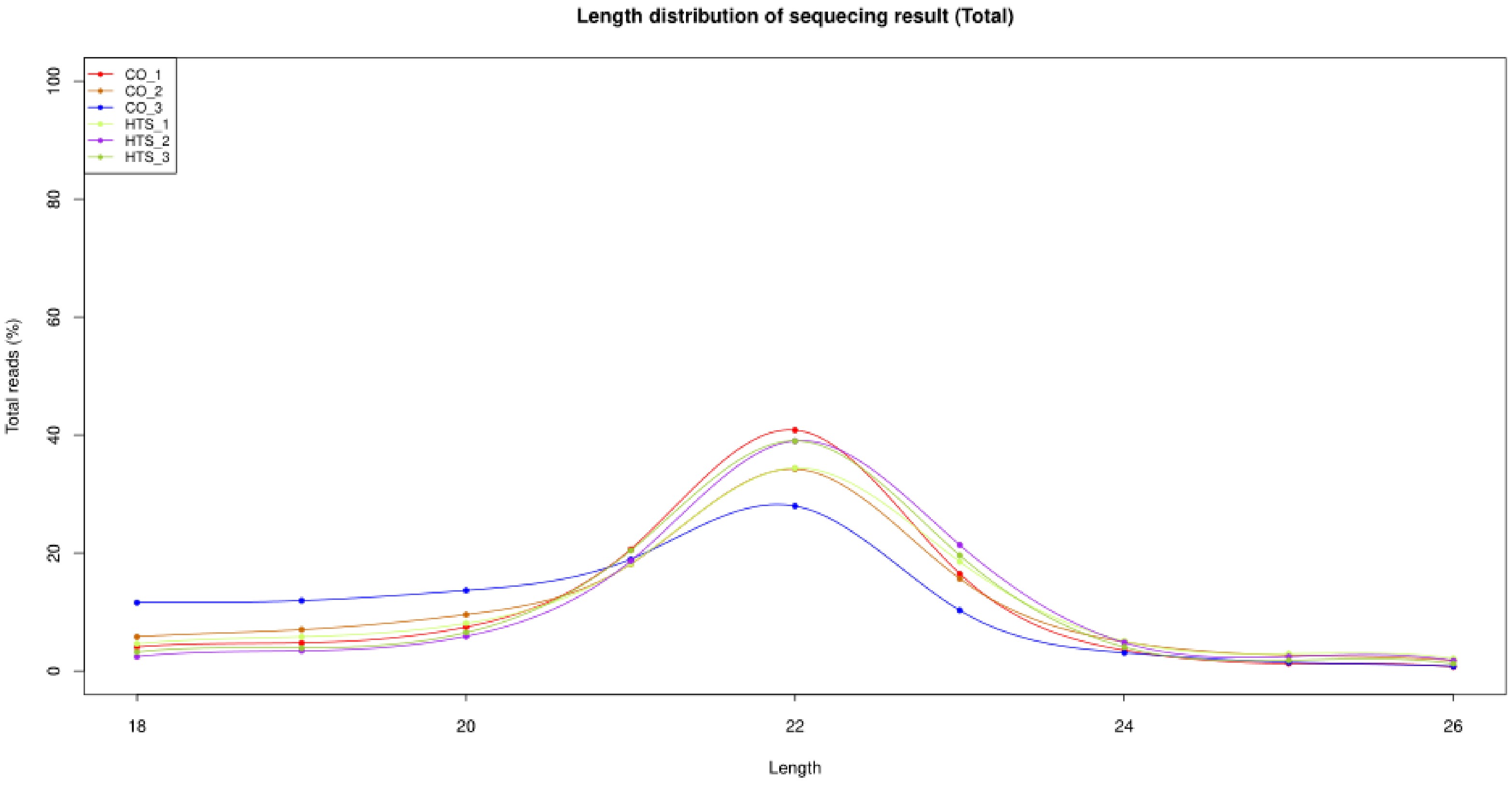

Table S1 Overview of reads for miRNA-seq of GIFT (*Oreochromis_niloticus*) from raw data to cleaned sequences

		CO_1				CO_2				CO_3			
lib	type	Total	% of Total	uniq	% of uniq	Total	% of Total	uniq	% of uniq	Total	% of Total	uniq	% of uniq
Raw reads	NA	13602611	100.00	906768	100.00	13029563	100.00	1057876	100.00	14426681	100.00	1276424	100.00
3ADT&length filter	Sequence type	4864085	35.76	604987	66.72	4733855	36.33	699545	66.13	6919560	47.96	938892	73.56
Junk reads	Sequence type	11607	0.09	2025	0.22	12299	0.09	2285	0.22	10197	0.07	1827	0.14
Rfam	RNA class	1282271	9.43	16104	1.78	2063742	15.84	18768	1.77	2102714	14.58	18004	1.41
mRNA	RNA class	877605	6.45	6957	0.77	1379764	10.59	6887	0.65	1356680	9.40	6718	0.53
Repeats	RNA class	345	0.00	24	0.00	647	0.00	16	0.00	1274	0.01	16	0.00
valid reads	Sequence type	7336868	53.94	279891	30.87	6080277	46.67	334166	31.59	5233861	36.28	314560	24.64
rRNA	RNA class	1078952	7.93	9336	0.07	1841155	14.13	10610	0.08	1866484	12.94	10398	0.07
tRNA	RNA class	123183	0.91	3123	0.02	91454	0.70	3394	0.03	88536	0.61	3137	0.02
snoRNA	RNA class	7374	0.05	462	0.00	7324	0.06	475	0.00	9501	0.07	463	0.00
snRNA	RNA class	5720	0.04	388	0.00	12645	0.10	1024	0.01	12194	0.08	935	0.01
other Rfam RNA	RNA class	67042	0.49	2795	0.02	111164	0.85	3265	0.03	125999	0.87	3071	0.02

		HTS_1				HTS_2				HTS_2			
lib	type	Total	% of Total	uniq	% of uniq	Total	% of Total	uniq	% of uniq	Total	% of Total	uniq	% of uniq
Raw reads	NA	14395061	100.00	1023917	100.00	11506732	100.00	795917	100.00	11963811	100.00	963554	100.00
3ADT&length filter	Sequence type	4167618	28.95	671207	65.55	3206612	27.87	537916	67.58	4493829	37.56	731099	75.88
Junk reads	Sequence type	12723	0.09	2408	0.24	9472	0.08	1685	0.21	11028	0.09	1689	0.18
Rfam	RNA class	2404413	16.70	18982	1.85	1576437	13.70	16139	2.03	1339663	11.20	15200	1.58
mRNA	RNA class	1534163	10.66	6580	0.64	904634	7.86	5601	0.70	966296	8.08	5480	0.57
Repeats	RNA class	521	0.00	13	0.00	206	0.00	12	0.00	229	0.00	13	0.00
valid reads	Sequence type	7645402	53.11	328500	32.08	6614342	57.48	237665	29.86	6033205	50.43	213039	22.11

rRNA	RNA class	2047992	14.23	10906	0.08	1136228	9.87	9666	0.08	1178432	9.85	9445	0.08
tRNA	RNA class	204532	1.42	3367	0.02	352332	3.06	2700	0.02	89903	0.75	2287	0.02
snoRNA	RNA class	7620	0.05	511	0.00	4386	0.04	395	0.00	3983	0.03	338	0.00
snRNA	RNA class	9130	0.06	778	0.01	4778	0.04	302	0.00	2893	0.02	274	0.00
other Rfam RNA	RNA class	135139	0.94	3420	0.02	78713	0.68	3076	0.03	64452	0.54	2856	0.02

Overview of reads from raw data to cleaned sequences.

3ADT&length filter: reads removed due to 3ADT not found and length with <18 nt and >25 nt were removed (for plants); length with<18 and >26 were remove(for animals)

Junk reads:Junk: >=2N, >=7A, >=8C, >=6G, >=7T, >=10Dimer, >=6Trimer, or >=5Tetramer

Rfam: Collection of many common non-coding RNA families except micro RNA; http://rfam.janelia.org

Repeats: Prototypic sequences representing repetitive DNA from different eukaryotic species; http://www.girinst.org/repbase.

Notes: valid reads may not be equal to raw reads - 3ADT&length filter - Junk reads ˙C mRNA ˙C Rfam - Repeats, because there are overlapped sequences between mRNA £¬Rfam and Repeats, details please refer to _comp_others.txt in fold 2_MappedData.

mRNA_Database:http://asia.ensembl.org/Oreochromis_niloticus/Info/Index/cdna/

Table S2 List of know-miRNA and novel-miRNA of GIFT (*Oreochromis_niloticus*)

miRNA_Index	miR_name	miR_seq	len	group
1	dre-miR-1	TGGAATGTAAAGAAGTATGTAT	22	gp1
2	ssa-miR-1-4-5p	ACATACTTCTTTATATGCCCATA	23	gp1
3	ssa-miR-1-3p	TGGAATGTAAAGAAGTATGTAT	22	gp1
4	dre-let-7a	TGAGGTAGTAGGTTGTATAGTT	22	gp1
5	ssa-let-7a-3-3p	CTATACAACTTACTGTCTTTCC	22	gp1
6	dre-let-7c-5p	TGAGGTAGTAGGTTGTATGGTT	22	gp1
7	dre-let-7c-3p	CTGTACAACCTTCTAGCTTTCC	22	gp1
8	ssa-let-7a-5p	TGAGGTAGTAGGTTGTATAGTT	22	gp1
9	ssa-let-7a-4-3p_2ss9TC15GA	CTATACAGCCTATTACCTTCCT	22	gp1
10	ipu-let-7a	TGAGGTAGTAGGTTGTATAGTT	22	gp1
11	ipu-let-7a-5-p3	CTATACAGTCTATTGCCTTCCT	22	gp1
12	dre-let-7a	TGAGGTAGTAGGTTGTATAGTT	22	gp1
13	ssa-let-7a-3p	CTGTACAGCCTCCTAGCTTTCC	22	gp1
14	ssa-miR-7a-5p_R+1	TGGAAGACTAGTGATTTTGTTGTT	24	gp1
15	ssa-miR-7a-3-3p_R-2	CAACAAATCACAGTCTGCCA	20	gp1
16	ipu-let-7b_R+1	TGAGGTAGTAGGTTGTGTGGTT	22	gp1
17	aca-let-7b-3p_R+1	CTATACAACCTACTGCCTTCCT	22	gp1
18	ssa-let-7g-5p	TGAGGTAGTAGTTTGTATAGTT	22	gp1
19	ssa-let-7g-3p_1ss22CT	CTATACAGTCTACTGTCTTTCT	22	gp1
20	ssa-let-7e-5p	TGAGGTAGTAGATTGAATAGTT	22	gp1
21	ssa-let-7e-3p_1ss22CT	CTATACAATCTACTGTCTTTCT	22	gp1
22	ola-let-7e_R+4	TGAGGTAGTAGATTGAATAGTT	22	gp1
23	ola-let-7e-p3	CTATACAATCTACTGTCTTTC	21	gp1

24	dre-let-7g	TGAGGTAGTAGTTTGTATAGTT	22	gp1
25	dre-let-7g-1-p3	CTATACAGCCTACTGTCTTTCT	22	gp1
26	tni-let-7j_1ss11TG	TGAGGTAGTTGTTTGTACAGTT	22	gp1
27	tni-let-7j-p3_1ss22CT	CTGTACAAGTGACTGCCTTGCT	22	gp1
28	dre-let-7i	TGAGGTAGTAGTTTGTGCTGTT	22	gp1
29	ssa-let-7i-2-3p_1ss22CT	CTGCGCAAGCTACTGCCTTGCT	22	gp1
30	ola-let-7a	TGAGGTAGTAGGTTGTATAGTT	22	gp1
31	ola-let-7a-3p_R+1	CTATACAACTTACTGTCTTTCC	22	gp1
32	ssa-let-7f-5p_1ss19TA	TGAGGTAGTAGATTGTATAGTT	22	gp1
33	ssa-let-7f-3p_1ss22CT	CTATACAATCTATTGCCTTCCT	22	gp1
34	dre-let-7b	TGAGGTAGTAGGTTGTGTGGTT	22	gp1
35	ssa-let-7b-3p_1ss3GA	CTATACAACCTACTGCCTTCCC	22	gp1
36	ssa-let-7a-5p	TGAGGTAGTAGGTTGTATAGTT	22	gp1
37	ssa-let-7a-3p	CTGTACAGCCTCCTAGCTTTCC	22	gp1
38	dre-let-7d-5p	TGAGGTAGTTGGTTGTATGGTT	22	gp1
39	dre-let-7c-3p	CTGTACAACCTTCTAGCTTTCC	22	gp1
40	dre-let-7a	TGAGGTAGTAGGTTGTATAGTT	22	gp1
41	dre-let-7a-1-p3_1ss5AT	CTATTCAACCTACTGTCTTTCT	22	gp1
42	ssa-let-7h-5p	TGAGGTAGTAAGTTGTGTTGTT	22	gp1
43	ssa-let-7h-3p	CTATACAACTTACTGCCTTCCT	22	gp1
44	ssa-miR-7a-5p	TGGAAGACTAGTGATTTTGTTGT	23	gp1
45	ssa-miR-7a-5p_R+1	TGGAAGACTAGTGATTTTGTTGTT	24	gp1
46	tni-miR-7_R+1	TGGAAGACTAGTGATTTTGTTGTT	24	gp1
47	ipu-let-7d	TGAGGTAGTTGGTTGTATGGTT	22	gp1
48	pol-let-7d-3p	CTGTACAACCTTCTAGCTTTCC	22	gp1
49	dre-miR-9-5p_R-2	TCTTTGGTTATCTAGCTGTAT	21	gp1

50	dre-miR-9-3p_R+1	TAAAGCTAGATAACCGAAAGTA	22	gp1
51	ssa-miR-9a-5p_R-2	TCTTTGGTTATCTAGCTGTAT	21	gp1
52	ssa-miR-9a-3-3p	TAAAGCTAGAGAACCGAAAGTA	22	gp1
53	ola-miR-9b-5p_R-2	TCTTTGGTTATCTAGCTGTAT	21	gp1
54	ola-miR-9a-3p_L-1R+2	TAAAGCTAGATAACCGAAAGTA	22	gp1
55	xtr-miR-9a-5p_R-2	TCTTTGGTTATCTAGCTGTAT	21	gp1
56	xtr-miR-9a-3p_R+1	TAAAGCTAGATAACCGAAAGTA	22	gp1
57	ssa-miR-9a-5p_R-2	TCTTTGGTTATCTAGCTGTAT	21	gp1
58	ssa-miR-9a-4-3p_L-1R+1	TAAAGCTAGATAACCGAAAGTA	22	gp1
59	ssa-miR-9a-5p_R-2	TCTTTGGTTATCTAGCTGTAT	21	gp1
60	ssa-miR-9a-2-3p	TAAAGCTAGATAACCGAAAGTA	22	gp1
61	dre-miR-9-5p_R-2	TCTTTGGTTATCTAGCTGTAT	21	gp1
62	dre-miR-9-4-3p_1ss19TA	TAAAGCTAGAGAACCGAAAGTA	22	gp1
63	dre-miR-10b-5p_R-1	TACCCTGTAGAACCGAATTTGT	22	gp1
64	ssa-miR-10b-5p	TACCCTGTAGAACCGAATTTGT	22	gp1
65	ola-miR-10d	TACCCTGTAGAACCGAATGTGT	22	gp1
66	tni-miR-10c	TACCCTGTAGATCCGGATTTGT	22	gp1
67	tni-mir-10c-p3	ACAAATTCGCTTCTAGGGGAGT	22	gp1
68	fru-miR-15a_R+1	TAGCAGCACGGAATGGTTTGTGT	23	gp1
69	fru-mir-15a-p3	CAGGCCATACTGTGCTGCCGCA	22	gp1
70	ssa-miR-15c-5p	TAGCAGCGCATCATGGTTTGA	21	gp1
71	ssa-mir-15c-2-p3_1ss11CA	TGCGAACCATAATTTGCTGCTT	22	gp1
72	ssa-miR-16b-5p_R-1_1ss21TC	TAGCAGCACGTAAATATTGGC	21	gp1
73	ssa-miR-16b-3p_R-1_1ss13TA	CCCAATATTAGCAGTGCTGCTT	22	gp1
74	ssa-miR-16a-5p	TAGCAGCACGTAAATATTGGAG	22	gp1
75	ssa-miR-16a-3p_R+1_1ss10TA	CCAGTATTGATCGTGCTGCTGAA	23	gp1

76	ola-miR-17_R+4	CAAAGTGCTTACAGTGCAGGTA	22	gp1
77	ola-mir-17-p3	ACTGCAGTGAAGGCACTTTCA	21	gp1
78	ssa-miR-17-5p_R-1	CAAAGTGCTTACAGTGCAGGTA	22	gp1
79	ssa-miR-17-3-3p_L-1_2ss19CA20TC	CTGCAGTGGAGGCACTTACAGC	22	gp1
80	dre-miR-18b-5p_1ss11TC	TAAGGTGCATCTAGTGCAGATA	22	gp1
81	dre-miR-18b-3p_L+1R-2_1ss15CT	ACTGCCCTAAGTGCTCCTTCT	21	gp1
82	dre-miR-18a	TAAGGTGCATCTAGTGCAGATA	22	gp1
83	oha-miR-18a-3p_R-1	ACTGCCCTAAGTGCTCCTTCT	21	gp1
84	ssa-miR-18a-5p	TAAGGTGCATCTAGTGTAGTTA	22	gp1
85	rno-miR-19a-3p_R-2	TGTGCAAATCTATGCAAAACT	21	gp1
86	dre-miR-19a-5p_L+1R-1	GCTAGTTTTGCATAGTTGCACT	22	gp1
87	dre-miR-19a-3p_2ss11TC23AT	TGTGCAAATCCATGCAAAACTGT	23	gp1
88	ola-mir-19d-p5	AGCTTTGCAGGGTGGGCAGTCAGC	24	gp1
89	ola-miR-19d_R+1	TGTGCAAACCCATGCAAAACTGA	23	gp1
90	ssa-miR-19c-4-5p_R+1_1ss17TA	AGTTTTGCTGGTTTGCATTCAGC	23	gp1
91	ssa-miR-19c-3p	TGTGCAAATCCATGCAAAACTG	22	gp1
92	ssa-miR-19c-3p	TGTGCAAATCCATGCAAAACTG	22	gp1
93	ola-miR-20a_R+2	TAAAGTGCTTATAGTGCAGGTAG	23	gp1
94	ola-mir-20a-1-p3	ACTGCAATGTAAGCACTTGAAG	22	gp1
95	ssa-miR-21b-5p	TAGCTTATCAGACTGGTGTTGGC	23	gp1
96	ssa-miR-21b-3p_R-1	CGACAACAGTCTGTAGGCTGT	21	gp1
97	dre-miR-22a-5p	AGTTCTTCACTGGCAAGCTTTA	22	gp1
98	dre-miR-22a-3p	AAGCTGCCAGCTGAAGAACTGT	22	gp1
99	dre-miR-22b-5p_R-1	CGTTCTTCACTGGCTAGCTTT	21	gp1
100	dre-miR-22b-3p	AAGCTGCCAGTTGAAGAGCTGT	22	gp1
101	ola-mir-22-2-p5	GTTCTTCACTGGCAAGCTTTA	21	gp1

102	ola-miR-22	AAGCTGCCAGCTGAAGAACTG	21	gp1
103	dre-miR-23a-3-5p	GGATTCCTGGCAGAGTGATTT	21	gp1
104	dre-miR-23a-3p_R-1	ATCACATTGCCAGGGATTTCC	21	gp1
105	dre-mir-23a-1-p5	GGGTTCCTGGCACCGTGATTT	21	gp1
106	dre-miR-23a-3p_R-1	ATCACATTGCCAGGGATTTCC	21	gp1
107	ipu-miR-23a	ATCACATTGCCAGGGATTTCC	21	gp1
108	ssa-miR-23b-5p_R+1	GGGTTCCTGGCGTGCTGATTT	21	gp1
109	ssa-miR-23b-3p_R-3	ATCACATTGCCAGGGATTACC	21	gp1
110	ola-mir-24a-3-p5_1ss19TA	TGTGCCTACTGAGCTGATAATC	22	gp1
111	ipu-miR-24_1ss23GT	TGGCTCAGTTCAGCAGGAACAGT	23	gp1
112	ssa-miR-24a-4-5p	TGCCTGCTGTGCTGATAATCAGT	23	gp1
113	ssa-miR-24a-3p_R+3	TGGCTCAGTTCAGCAGGAACAGT	23	gp1
114	cfa-miR-24_R-3	TGGCTCAGTTCAGCAGGAAC	20	gp1
115	fru-miR-24-5p_L-1	TGCCTACTGAACTGGTATCAGT	22	gp1
116	fru-miR-24-3p_R+1	TGGCTCAGTTCAGCAGGAACAGT	23	gp1
117	ola-miR-24b-5p	TGCCTACTGAGCTGATAACAGT	22	gp1
118	ipu-miR-24b	TGGCTCAGTTCAGCAGGAAC	20	gp1
119	dre-miR-25-5p_2ss18GA19CT	AGGCGGAGACTTGGGCAATTGCC	23	gp1
120	dre-miR-25-3p	CATTGCACTTGTCTCGGTCTGA	22	gp1
121	hhi-miR-26_R+1	TTCAAGTAATCCAGGATAGGCT	22	gp1
122	gga-miR-26a-3p_R-1_2ss6TG11GA	CCTATGCTTGATTACTTGCACT	22	gp1
123	ssa-miR-26a-5p	TTCAAGTAATCCAGGATAGGCT	22	gp1
124	ssa-miR-26a-6-3p_2ss8AG19TC	CCTATTCGTGATTACTTGCACT	22	gp1
125	ssa-miR-26a-5p	TTCAAGTAATCCAGGATAGGCT	22	gp1
126	ssa-miR-26a-4-3p	CCTATTCTTGATTACTTGTTTC	22	gp1
127	ipu-miR-26a	TTCAAGTAATCCAGGATAGGCT	22	gp1

128	ipu-mir-26a-1-p3_1ss8GC	CCTATTCCGGATGACTTGGTTC	22	gp1
129	ssa-miR-26b-5p	TTCAAGTAATCCAGGATAGGTT	22	gp1
130	ola-miR-27c-5p_L+1R+1_1ss10GA	CAGGACTTAACCCACATGTGAACA	24	gp1
131	ola-miR-27c-3p_R+1	TTCACAGTGGTTAAGTTCTGC	21	gp1
132	ssa-miR-27b-5p	AGAGCTTAGCTGATTGGTGAAC	22	gp1
133	ssa-miR-27b-3p	TTCACAGTGGCTAAGTTCTGC	21	gp1
134	ssa-miR-27c-5p_R+1	AGAGCTTAGCTAATTGGTGAGC	22	gp1
135	ssa-miR-27c-3p	TTCACAGTGGCTAAGTTCAGT	21	gp1
136	ola-miR-27a_R+2_1ss19CT	TTCACAGTGGCTAAGTTCTGCT	22	gp1
137	ssa-miR-29b-3p	TAGCACCATTTGAAATCGGTTA	22	gp1
138	ssa-miR-29a-5p	CTGGTTTCACATGGTGGTTTAGA	23	gp1
139	dre-miR-29b	TAGCACCATTTGAAATCAGTGT	22	gp1
140	ssa-miR-29b-1-5p_1ss18TC	ACTGATTTCTTCTGGTGCTTAGA	23	gp1
141	ssa-miR-29b-3p	TAGCACCATTTGAAATCGGTTA	22	gp1
142	tni-miR-29b	TAGCACCATTTGAAATCAGTGT	22	gp1
143	mmu-miR-30d-5p_R-2	TGTAAACATCCCCGACTGGA	20	gp1
144	mmu-miR-30d-3p_R-2_1ss10AG	CTTTCAGTCGGATGTTTGCT	20	gp1
145	fru-miR-30c	TGTAAACATCCTACACTCTCGG	22	gp1
146	fru-mir-30c-p3_1ss6GA	CCGGGAGTGGGACTGTTTGCACT	23	gp1
147	dre-miR-30e-5p_R+2	TGTAAACATCCTTGACTGGAAGCT	24	gp1
148	dre-miR-30e-3p	CTTTCAGTCGGATGTTTGCAGC	22	gp1
149	ssa-miR-30e-5p	TGTAAACATCCTACACTCAGCT	22	gp1
150	ssa-miR-30a-5p	TGTAAACATCCTACACTCTCAGC	23	gp1
151	ssa-miR-30a-4-3p	CTGGGAGAGGGGTGTTTACGCT	22	gp1
152	ssa-miR-30c-5p	TGTAAACATCCTTGACTGGAAGCT	24	gp1
153	ssa-miR-30c-3p	CTTTCAGTCGGATGTTTGCAGC	22	gp1

154	tgu-miR-33-5p	GTGCATTGTAGTTGCATTGC	20	gp1
155	ola-miR-33_R+2	CAATGTACCTGCAGTGCAACA	21	gp1
156	ssa-miR-33b-5p_R-1	GTGCATTGTAGTTGCATTGC	20	gp1
157	ssa-miR-33a-3p	CAATGTGTCTGCAGTGCAGTA	21	gp1
158	dre-miR-34a	TGGCAGTGTCTTAGCTGGTTGT	22	gp1
159	dre-mir-34a-p3	AATCAGCAAGTATACTGCCGCA	22	gp1
160	fru-mir-92-2-p5_1ss12AG	AGGTGGGGATCGGTAGCAATGCT	23	gp1
161	ipu-miR-92a	TATTGCACTTGTCCCGGCCTGT	22	gp1
162	ola-mir-92a-1-p5	AGGTTGGGAGAGGTGGCAATGCT	23	gp1
163	ssa-miR-92a-3p	TATTGCACTTGTCCCGGCCTGT	22	gp1
164	ssa-miR-92b-3p	TATTGCACTCGTCCCGGCCTCC	22	gp1
165	ssa-miR-96-5p	TTTGGCACTAGCACATTTTTGCT	23	gp1
166	ssa-miR-99-5p	AACCCGTAGATCCGATCTTGTG	22	gp1
167	ola-miR-100_R+3	AACCCGTAGATCCGAACTTGTG	22	gp1
168	ola-mir-100-1-p3_1ss9CT	CAAGCTTGTATCTACAGGTCTG	22	gp1
169	ipu-miR-100	AACCCGTAGATCCGAACTTGTG	22	gp1
170	ola-mir-100-2-p3	CAAGCTCGTATCTATAGGTATG	22	gp1
171	ssa-miR-101b-5p_L+1R-2	TCAGTTATCATGGTACCGGTGCT	23	gp1
172	ssa-miR-101b-3p_R+1_1ss10AG	TACAGTACTGTGATAACTGAAG	22	gp1
173	ssa-miR-101a-5p_R-1_1ss12TA	TCAGTTATCACAGTGCTGATGC	22	gp1
174	dre-miR-101a	TACAGTACTGTGATAACTGAAG	22	gp1
175	tni-mir-103-p5	AGCCTCTTTACAGTGCTGCCTTG	23	gp1
176	ipu-miR-103	AGCAGCATTGTACAGGGCTATGA	23	gp1
177	ssa-miR-103-5p_2ss11TC13AG	AGCCTCTTTACGGTGCTGCCTTGT	24	gp1
178	ssa-miR-103-3p	AGCAGCATTGTACAGGGCTATGA	23	gp1
179	ssa-miR-106b-5p_L-1R+1	AAAGTGCTTACAGTGCAGGTAG	22	gp1

180	ssa-miR-107-5p	AGCTTCTTTACAGTGTTGCCTTG	23	gp1
181	ssa-miR-107-3p	AGCAGCATTGTACAGGGCTATC	22	gp1
182	dre-miR-122	TGGAGTGTGACAATGGTGTTTG	22	gp1
183	ssa-miR-122-2-3p_R-1	AACGCCATTATCACACTAAAT	21	gp1
184	dre-miR-125b-5p	TCCCTGAGACCCTAACTTGTGA	22	gp1
185	dre-miR-125b-2-3p_L+1_1ss15CT	ACGGGTTGGGTTCTTGGGAGCT	22	gp1
186	ssa-miR-125b-5p_R-1	TCCCTGAGACCCTTAACCTGTG	22	gp1
187	ssa-miR-125b-2-3p	CAGGTGAGGTCCTTGGGAAC	20	gp1
188	ssa-miR-125a-5p	TCCCTGAGACCCTAACTTGTGA	22	gp1
189	ssa-miR-125a-2-3p	ACGGGTTAGGCTCTTGGGACGC	22	gp1
190	dre-miR-125a	TCCCTGAGACCCTTAACCTGTG	22	gp1
191	ssa-miR-125b-1-3p_R+1	ACAGGTGAGGTCCTCGGGAAC	21	gp1
192	ssa-miR-126-5p	CATTATTACTTTTGGTACGCG	21	gp1
193	ssa-miR-126-3p	TCGTACCGTGAGTAATAATGCA	22	gp1
194	tni-miR-128_R-1	TCACAGTGAACCGGTCTCTTT	21	gp1
195	ola-miR-128_R+1	TCACAGTGAACCGGTCTCTTT	21	gp1
196	ssa-miR-129-3p	AAGCCCTTACCCCAAAAAGCAT	22	gp1
197	dre-miR-129-3p	AAGCCCTTACCCCAAAAAGCAT	22	gp1
198	ssa-miR-130a-5p	ACTCTTTCCCTGTTGCACTACT	22	gp1
199	ssa-miR-130a-2-3p_R-1	CAGTGCAATAATGAAAGGGCAT	22	gp1
200	xtr-mir-130c-p5	GCCCTTTTTCTGTTGTACTACT	22	gp1
201	ssa-miR-130d-3p_R+1	CAGTGCAATATTAAAAGGGCAT	22	gp1
202	dre-miR-130c-5p	GCCCTTTTTCTGTTGTACTACT	22	gp1
203	dre-miR-130c-3p	CAGTGCAATATTAAAAGGGCAT	22	gp1
204	ssa-miR-132-5p	ACCGTGGCTTTAGATTGTTACT	22	gp1
205	ssa-miR-132-3p	TAACAGTCTACAGCCATGGTCG	22	gp1

206	dre-miR-132-3p	TAACAGTCTACAGCCATGGTCG	22	gp1
207	dre-miR-133a-5p	AGCTGGTAAAATGGAACCAAAT	22	gp1
208	dre-miR-133a-3p_L-1R+1	TTGGTCCCCTTCAACCAGCTGT	22	gp1
209	dre-miR-133b-3p_R-1	TTTGGTCCCCTTCAACCAGCT	21	gp1
210	dre-miR-133a-5p	AGCTGGTAAAATGGAACCAAAT	22	gp1
211	dre-miR-133a-3p_L-1R+1	TTGGTCCCCTTCAACCAGCTGT	22	gp1
212	ssa-miR-135b-5p_R-1	TATGGCTTTCTATTCCTATGTG	22	gp1
213	ssa-miR-135a-5p_R-1	TATGGCTTTTTATTCCTATCTG	22	gp1
214	ssa-miR-135a-3p_L+1R-1	ATATAGGGATGGAAGCCATGC	21	gp1
215	ola-miR-135b_R+4	TATGGCTTTTTATTCCTACGTGA	23	gp1
216	dre-miR-135c	TATGGCTTTCTATTCCTATGTG	22	gp1
217	ssa-miR-135b-3-3p_R+1_1ss11CT	ACATAGGGTCTAAAGCCATTGG	22	gp1
218	ssa-miR-137-3p_R-2	TTATTGCTTGAGAATACGCGT	21	gp1
219	ssa-miR-137-3p_R-2	TTATTGCTTGAGAATACGCGT	21	gp1
220	ssa-miR-138-5p	AGCTGGTGTTGTGAATCAGGCCG	23	gp1
221	dre-miR-138-5p_R+1	AGCTGGTGTTGTGAATCAGGCCG	23	gp1
222	ola-miR-139_R+2	TCTACAGTGCATGTGTCTCCAGT	23	gp1
223	dre-miR-140-5p	CAGTGGTTTTACCCTATGGTAG	22	gp1
224	dre-miR-140-3p_L-1	ACCACAGGGTAGAACCACGGAC	22	gp1
225	dre-miR-142a-5p	CATAAAGTAGAAAGCACTACT	21	gp1
226	dre-miR-142a-3p_R-1	TGTAGTGTTTCCTACTTTATGG	22	gp1
227	mmu-miR-142a-5p	CATAAAGTAGAAAGCACTACT	21	gp1
228	ipu-miR-142_L+1R-1	TGTAGTGTTTCCTACTTTATGG	22	gp1
229	ssa-miR-143-5p_R+1	GGTGCAGTGCTGCATCTCTGGTC	23	gp1
230	ssa-miR-143-3p_R+1	TGAGATGAAGCACTGTAGCTCT	22	gp1
231	ssa-miR-144-5p_R-1_1ss11AT	GGATATCATCTTATACTGTAAGT	23	gp1

232	ssa-miR-144-3p_R+1	CTACAGTATAGATGATGTACT	21	gp1
233	ssa-miR-145-5p	GTCCAGTTTTCCCAGGAATCCCT	23	gp1
234	ssa-miR-145-3p_L+2R-1	GGATTCCTGGAAATACTGTTCT	22	gp1
235	ssa-miR-146a-5p	TGAGAACTGAATTCCATAGATGG	23	gp1
236	ssa-miR-146a-3p_R-2	ATCTATGGGCTCAGTTCTTCT	21	gp1
237	ssa-miR-148a-5p_1ss19CA	AAGTTCTGTGATACACTTAGACT	23	gp1
238	dre-miR-148_R-1	TCAGTGCATTACAGAACTTTG	21	gp1
239	fru-mir-152-p5	AAGTTCTGTGATACACTCTGACT	23	gp1
240	ssa-miR-152-3p	TCAGTGCATAACAGAACTTTG	21	gp1
241	ssa-miR-153a-2-5p_3ss5CT6TC12AG	TCATTCTTGTGGTTTGCAGCT	21	gp1
242	ssa-miR-153a-3p_1ss21TG	TTGCATAGTCACAAAAATGAGC	22	gp1
243	ssa-miR-153a-3p	TTGCATAGTCACAAAAATGATC	22	gp1
244	tni-miR-153a	TTGCATAGTCACAAAAGTGATC	22	gp1
245	dre-miR-153a-3p	TTGCATAGTCACAAAAGTGATC	22	gp1
246	ssa-miR-181a-5p	AACATTCAACGCTGTCGGTGAGT	23	gp1
247	ssa-miR-181a-5-3p	ACCATCGACCGTTGACTGTGCC	22	gp1
248	ssa-miR-181b-5p_R-1_1ss18CG	AACATTCATTGCTGTCGGTGGGT	23	gp1
249	ola-miR-181b-5p_R-1	AACATTCATTGCTGTCGGTGGGT	23	gp1
250	ola-miR-181b-3p_R+1	CTCACTGAACGATGAATGCAA	21	gp1
251	ssa-miR-181a-5p	AACATTCAACGCTGTCGGTGAGT	23	gp1
252	ssa-miR-181a-2-3p	ACCATCGACCGTTGACTGTACC	22	gp1
253	dre-miR-181b-5p_R+1	AACATTCATTGCTGTCGGTGGGT	23	gp1
254	dre-miR-181b-3p_R-2	CTCACTGATCAATGAATGCA	20	gp1
255	ipu-miR-181a_R+1	AACATTCAACGCTGTCGGTGAGT	23	gp1
256	ipu-mir-181a-5-p3	ACCATCGAGTGTTGAGTGTACC	22	gp1
257	dre-miR-182-5p	TTTGGCAATGGTAGAACTCACA	22	gp1

258	ssa-miR-183-5p	TATGGCACTGGTAGAATTCACT	22	gp1
259	dre-miR-183-5p_R-1	TATGGCACTGGTAGAATTCACT	22	gp1
260	dre-miR-184_R-1	TGGACGGAGAACTGATAAGGG	21	gp1
261	ola-miR-184-3p	TGGACGGAGAACTGATAAGGG	21	gp1
262	dre-miR-187_R+3	TCGTGTCTTGTGTTGCAGCCAGT	23	gp1
263	dre-miR-190a_R+1	TGATATGTTTGATATATTAGGTT	23	gp1
264	ssa-miR-190a-3p	ACTATATATCAAACATATTCCT	22	gp1
265	ssa-miR-192a-5p	ATGACCTATGAATTGACAGCC	21	gp1
266	ssa-miR-192b-5p	CCTGTCAGTTCTGTAGGCCACT	22	gp1
267	dre-miR-193a-5p	TGGGTCTTTGCGGGCAAGGTGA	22	gp1
268	dre-miR-193a-3p	AACTGGCCTACAAAGTCCCAGT	22	gp1
269	tni-miR-194_R+1	TGTAACAGCAACTCCATGTGGA	22	gp1
270	dre-miR-196a-5p	TAGGTAGTTTCATGTTGTTGGG	22	gp1
271	dre-miR-196a-5p	TAGGTAGTTTCATGTTGTTGGG	22	gp1
272	dre-miR-199-5p	CCCAGTGTTCAGACTACCTGTTC	23	gp1
273	dre-miR-199-3-3p_1ss10CT	ACAGTAGTCTGCACATTGGTT	21	gp1
274	dre-miR-199-5p	CCCAGTGTTCAGACTACCTGTTC	23	gp1
275	dre-miR-199-3p_L-1	ACAGTAGTCTGCACATTGGTT	21	gp1
276	ipu-miR-199b_L-1R+1	AACCAATGTGCAGACTACTGTT	22	gp1
277	ssa-miR-199a-5p	CCCAGTGTTCAGACTACCTGTTC	23	gp1
278	ssa-miR-199a-3p	ACAGTAGTCTGCACATTGGTT	21	gp1
279	dre-miR-199-5p	CCCAGTGTTCAGACTACCTGTTC	23	gp1
280	dre-miR-199-3p_L-1	ACAGTAGTCTGCACATTGGTT	21	gp1
281	ssa-miR-200b-5p_1ss10TC	CATCTTACCCGACAGTGCTGGA	22	gp1
282	ssa-miR-200b-3p	TAACACTGTCTGGTAACGATGTT	23	gp1
283	ssa-miR-200a-2-5p	CATCTTACGAGGCAGCATTGGA	22	gp1

284	ssa-miR-200a-3p	TAATACTGCCTGGTAATGATGAT	23	gp1
285	ssa-miR-203a-2-5p_1ss10TC	AGTGGTTCTCAACAGTTCAACA	22	gp1
286	ssa-miR-203a-3p	GTGAAATGTTTAGGACCACTTG	22	gp1
287	xtr-miR-204	TTCCCTTTGTCATCCTATGCCT	22	gp1
288	ssa-miR-204-5p	TTCCCTTTGTCATCCTATGCCT	22	gp1
289	tni-miR-204a	TTCCCTTTGTCATCCTATGCCT	22	gp1
290	ipu-miR-205_R-1	TCCTTCATTCCACCGGAGTCTG	22	gp1
291	tni-mir-205-p3	TTTCAGTGGTGTGAAGTGTAAG	22	gp1
292	ssa-miR-205b-5p	TCCTTCATTCCACCGGAGTCTG	22	gp1
293	ssa-miR-206-3p	TGGAATGTAAGGAAGTGTGTGG	22	gp1
294	dre-miR-210-5p	AGCCACTGACTAACGCACATTG	22	gp1
295	dre-miR-210-3p_1ss22AT	CTGTGCGTGTGACAGCGGCTAT	22	gp1
296	ssa-miR-212a-5p_1ss10TC	ACCTTGGCTCTAGACTGCTTACT	23	gp1
297	ssa-miR-212a-3p	TAACAGTCTACAGTCATGGCT	21	gp1
298	ssa-miR-212b-5p	ACCTTGGCTCTAGACTGCTTACT	23	gp1
299	dre-miR-212	TAACAGTCTACAGTCATGGCT	21	gp1
300	ssa-miR-214-5p	TGCCTGTCTACACTTGCTGTGC	22	gp1
301	ssa-miR-214-3p_L-1_1ss23AT	ACAGCAGGCACAGACAGGCAGT	22	gp1
302	ssa-miR-214-5p	TGCCTGTCTACACTTGCTGTGC	22	gp1
303	ssa-miR-214-3p_L-1R-1	ACAGCAGGCACAGACAGGCAG	21	gp1
304	tni-miR-216b	TAATCTCTGCAGGCAACTGTGA	22	gp1
305	tni-mir-216b-p3	ACAATCACCTGGAGAGATTCT	21	gp1
306	tni-miR-216a	AAATCTCAGCTGGCAACTGTGA	22	gp1
307	tni-mir-216a-p3	CACAATGGCCTCTGGGATTATG	22	gp1
308	tni-miR-217_R+1	TACTGCATCAGGAACTGATTGGC	23	gp1
309	tni-mir-217-p3	CAACAGTACCTGATGCATTGCC	22	gp1

310	dre-miR-218b_R-1	TTGTGCTTGATCTAACCATGC	21	gp1
311	ola-miR-218b_R+2	TTGTGCTTGATCTAACCATGTG	22	gp1
312	dre-miR-218a	TTGTGCTTGATCTAACCATGTG	22	gp1
313	ssa-miR-221-3p_R-1_1ss5AG	ACCTGGCATACAATGTAGATTT	22	gp1
314	ssa-miR-221-5p	AGCTACATTGTCTGCTGGGTTT	22	gp1
315	pol-miR-221-5p_R-4	ACCTGGCATACAATGTAG	18	gp1
316	pol-miR-221-3p	AGCTACATTGTCTGCTGGGTTT	22	gp1
317	ssa-miR-222a-5p_R-2	TGCTCAGTAGGCAGTGTAGATC	22	gp1
318	ssa-miR-222a-3p_R-1	AGCTACATCTGGCTACTGGGTCT	23	gp1
319	ssa-miR-222b-5p	TGCTCAGTAGTCAGTGTAGATC	22	gp1
320	ssa-miR-222b-3p_R+1	AGCTACATCTGGCTACTGGGTCT	23	gp1
321	ola-mir-223-p5	TGTATTTGACAAGCTGAGTTGG	22	gp1
322	ola-miR-223_R+2	TGTCAGTTTGTCAAATACCCCA	22	gp1
323	ssa-miR-301d-5p_L+1	GCTCTGACTTCATTGCACTACT	22	gp1
324	ssa-miR-301d-3p_1ss1CT	TAGTGCAATAGTATTGTCAAAGC	23	gp1
325	dre-miR-301c-3p_R+1	CAGTGCAATAGTATTGTCATAGC	23	gp1
326	ssa-miR-338a-3-5p	AACAATATCCTGGTGCTGCCTGAGT	25	gp1
327	ssa-miR-338a-3p_R-1	TCCAGCATCAGTGATTTTGTT	21	gp1
328	ssa-miR-338a-3p_R-1	TCCAGCATCAGTGATTTTGTT	21	gp1
329	ssa-miR-338a-3p_R-1	TCCAGCATCAGTGATTTTGTT	21	gp1
330	ssa-miR-365-5p_R+3	AGGGACTTTTAGGGGCAGCTGTG	23	gp1
331	dre-miR-365	TAATGCCCCTAAAAATCCTTAT	22	gp1
332	ssa-miR-375-3p	TTTGTTCGTTCGGCTCGCGTTA	22	gp1
333	dre-miR-429a	TAATACTGTCTGGTAATGCCGT	22	gp1
334	hhi-miR-449_R-2	AGGCAGTGTCTTGTTAGCTGGT	22	gp1
335	ssa-miR-454-3p_R-2	TAGTGCAATATTGCTTATAGGGT	23	gp1

336	dre-miR-455-5p	TATGTGCCCTTGGACTACATCG	22	gp1
337	dre-miR-455-3p_L-1	TGCAGTCCATGGGCATATACAC	22	gp1
338	ssa-miR-455-5p	TATGTGCCCTTGGACTACATCG	22	gp1
339	ssa-miR-455-3p_L+1R-1	TGCAGTCCATGGGCATATACAC	22	gp1
340	ssa-miR-456-3p_1ss22AT	CAGGCTGGTTAGATGGTTGTCT	22	gp1
341	fru-mir-458-p5	AGCGCCATTTTCAGAGCTAT	20	gp1
342	fru-miR-458	ATAGCTCTTTAAATGGTACTGC	22	gp1
343	ssa-miR-460-5p	CCTGCATTGTACACACTGTGCG	22	gp1
344	ssa-miR-460-3p	CACAGCGCATACAATGTGGATG	22	gp1
345	ola-miR-462_L-1R+4	TAACGGAACCCATAATGCAGCT	22	gp1
346	ola-mir-462-p3	CTGGTTATGGGGTCCGTTTCC	21	gp1
347	ssa-miR-489-5p	TGGTCGTATGTATGACGTCATT	22	gp1
348	dre-miR-489_L-1	GTGACATCATATGTACGGCTGC	22	gp1
349	ssa-miR-499b-5p_R-1	TTAAGACTTGCAGTGATGTTT	21	gp1
350	ssa-miR-722-5p_L-1R+1	TTTGAAACGTTTTAGCCAAAA	21	gp1
351	dre-miR-722_L-2	TTTTGCAGAAACGTTTCAGATT	22	gp1
352	dre-miR-724	TTAAAGGGAATTTGCGACTGTT	22	gp1
353	ssa-miR-724-5p	TTAAAGGGAATTTGCGACTGTT	22	gp1
354	dre-miR-727-5p	TCAGTCTTCAATTCCTCCCAGC	22	gp1
355	dre-miR-727-3p_R-2	GTTGAGGCGAGTTGAAGACT	20	gp1
356	ssa-miR-730a-5p_R-1	TCCTCATTGTGCATGCTGTGT	21	gp1
357	ssa-miR-730a-3p	CACAGCGCCTGCAATGTGGAGG	22	gp1
358	ssa-miR-734-5p_L+1R-1	TGAACTATTCTGCAACATTTGT	22	gp1
359	ssa-miR-734-3p_R-1	TAAATGCTGCAGAATTGTGCT	21	gp1
360	dre-miR-1306_R-1	CCACCTCCCCTGCAAACGTCC	21	gp1
361	ssa-miR-1338-5p_R+1	AGGACTGTCCAACCTGAGAATG	22	gp1

362	ssa-miR-1338-3p	ATCTCAGGTTCGTCAGCCCATG	22	gp1
363	dre-miR-2187-5p_R-1	TTAATTAGTATAGCCTGTTTT	21	gp1
364	dre-miR-2187-3p_L+1R-1_1ss21AG	TTTACAGGCTATGCTAATCTGT	22	gp1
365	ssa-miR-2187-5p_L-1_1ss20AT	TTAATTAGTATAGCCTGTTTT	21	gp1
366	ssa-miR-2187-3p_L+1R-2_1ss21AG	TTTACAGGCTATGCTAATCTGT	22	gp1
367	dre-miR-2188-5p_R+1	AAGGTCCAACCTCACATGTCCT	22	gp1
368	dre-miR-2188-3p_L+1_2ss13TC14AG	GCTGTGTGAGGTCGGACCTATC	22	gp1
369	bta-mir-3596-p5_1ss4CT	AGGTAGTAGGTTGTATAGTTA	21	gp1
370	ipu-miR-3618_L+1	TGATTTCCAATAATTGAGACAGT	23	gp1
371	oha-mir-3618-p3	CAGTGATTCTGAAAGCTGTC	20	gp1
372	ssa-miR-7132b-5p	GACTTGGTCAAAGCTCCTCAGC	22	gp1
373	ssa-miR-7132b-3p	TGAGGCGTTTAGAACAAGTTCA	22	gp1
374	dre-miR-7147	TGTACCATGCTGGTAGCCAGT	21	gp1
375	dre-mir-7147-p3_2ss5AG20CT	TGGTGACCAGCGTTGTGCCT	20	gp1
1	oha-miR-1a-3p	TGGAATGTAAAGAAGTATGTAC	22	gp2
2	dre-miR-10a-5p	TACCCTGTAGATCCGAATTTGT	22	gp2
3	dre-miR-10a-5p_L-1	ACCCTGTAGATCCGAATTTGT	21	gp2
4	dre-miR-10b-2-3p	CAAATACGTCTCTACAGGAAT	21	gp2
5	aca-miR-18b-5p_R-3	TAAGGTGCATCTAGTGCAGT	20	gp2
6	dre-miR-21_1ss23CA	TAGCTTATCAGACTGGTGTTGGA	23	gp2
7	ola-mir-21-1-p3_1ss6AG	CAACAGCGGTCTGTAAGCTGGC	22	gp2
6	dre-miR-21_1ss23CA	TAGCTTATCAGACTGGTGTTGGA	23	gp2
8	oha-miR-22a	AAGCTGCCAGTTGAAGAACTGT	22	gp2
9	oha-miR-22a_R-2	AAGCTGCCAGTTGAAGAACT	20	gp2
10	dre-miR-24_R+2_1	TGGCTCAGTTCAGCAGGAACAGAA	24	gp2
11	pma-miR-24_R+1	TGGCTCAGTTCAGCAGGAACAGA	23	gp2

12	dre-miR-26a-5p_L+1R-1	CTTCAAGTAATCCAGGATAGGC	22	gp2
13	dre-miR-26a-3p_R+1	CCTATTCGGGATGACTTGGTTCT	23	gp2
14	dre-miR-26b_R+1	TTCAAGTAATCCAGGATAGGTTA	23	gp2
15	ssa-mir-26a-5-p3_1ss11GC	CAGCCTATTCCGGATGACTTGG	22	gp2
16	ssa-miR-26d-5p_L+1_1ss13TC	CTTCAAGTAATCCAGGATAGGCT	23	gp2
17	hsa-miR-26a-2-3p_R+1	CCTATTCTTGATTACTTGTTTCT	23	gp2
16	ssa-miR-26d-5p_L+1_1ss13TC	CTTCAAGTAATCCAGGATAGGCT	23	gp2
18	ssa-miR-26d-5p_2ss10TC12TC	TTCAAGTAACCCAGGATAGGCT	22	gp2
19	dre-miR-27d_R-1_1ss19TG	TTCACAGTGGCTAAGTTCGTC	21	gp2
20	dre-miR-27b-3p_R+1	TTCACAGTGGCTAAGTTCTGCAT	23	gp2
21	dre-miR-27e_1ss22GA	TTCACAGTGGCTAAGTTCAGTA	22	gp2
22	ccr-miR-27c-3p_R+1	TTCACAGTGGTTAAGTTCTGCCA	23	gp2
23	ola-mir-29b-1-p5_2ss1GA18GT	ACTGGTTTCAGATGGTGTCTTAGA	24	gp2
24	bta-miR-29d-3p_R-3	TAGCACCATTTGAAATCGA	19	gp2
25	aca-miR-29b_R+1_1ss10TA	TAGCACCATATGAAATCAGTGT	22	gp2
26	ssa-miR-30d-5p_2ss13TC19AG	TGTAAACATCCTCGACTGGAAGCT	24	gp2
27	ssa-miR-30d-2-3p_1ss1TC	CTTTCAGTCGGATGTTTGCAGCT	23	gp2
26	ssa-miR-30d-5p_2ss13TC19AG	TGTAAACATCCTCGACTGGAAGCT	24	gp2
27	ssa-miR-30d-2-3p_1ss1TC	CTTTCAGTCGGATGTTTGCAGCT	23	gp2
28	ola-miR-30c_L+1R-3	CTGTAAACATCCTACACTCTC	21	gp2
29	tni-mir-30c-p3_2ss5GA23GT	CGGGAGTGGGACTGTTTGCACTT	23	gp2
30	dre-miR-30c-5p_R+2	TGTAAACATCCTACACTCTCAGCG	24	gp2
31	aca-miR-31-5p_R+2_1ss11TC	AGGCAAGATGCTGGCATAGCTGT	23	gp2
32	pma-miR-33a_R-2	GTGCATTGTAGTTGCATTGT	20	gp2
32	pma-miR-33a_R-2	GTGCATTGTAGTTGCATTGT	20	gp2
33	ssa-miR-106b-5p_R+1_2ss1AC12CT	CAAAGTGCTTATAGTGCAGGTAG	23	gp2

34	dre-miR-107b_R-1	AGCAGCATTGTACAGGGCTT	20	gp2
35	xtr-miR-122_L+1R-1	CTGGAGTGTGACAATGGTGTTTG	23	gp2
36	rno-miR-122-3p_L+1R+1_1ss10CT	AAACGCCATTATCACACTAAT	21	gp2
37	dre-miR-125b-5p_R+1	TCCCTGAGACCCTAACTTGTGAT	23	gp2
38	dre-miR-130b_R+2	CAGTGCAATAATGAAAGGGCATTA	24	gp2
39	oha-miR-133b-3p	TTTGGTCCCCTTCAACCAGCTAT	23	gp2
40	dre-miR-139-5p_R+5	TCTACAGTGCATGTGTCTCCAGG	23	gp2
41	oan-miR-139-3p_R-1_1ss8AC	TGGAGACCCAGCTCTGTTGGA	21	gp2
42	pma-miR-140_R+1	CAGTGGTTTTACCCTATGGTAGC	23	gp2
43	xtr-miR-142-5p_R+3	CATAAAGTAGAAAGCACTACTAG	23	gp2
44	xtr-miR-142-3p_L+1R-1	CTGTAGTGTTTCCTACTTTATGG	23	gp2
43	xtr-miR-142-5p_R+3	CATAAAGTAGAAAGCACTACTAG	23	gp2
44	xtr-miR-142-3p_L+1R-1	CTGTAGTGTTTCCTACTTTATGG	23	gp2
45	dre-miR-150_R+1_1ss1TA	ACTCCCAATCCTTGTACCAGTGT	23	gp2
46	dre-miR-152_R-1_1ss10GC	TCAGTGCATCACAGAACTTTG	21	gp2
47	dre-miR-152_R-1_2ss10GT21GA	TCAGTGCATTACAGAACTTTA	21	gp2
48	dre-miR-153c-3p_R+1	TTGCATAGTCACAAAAATGATCA	23	gp2
49	dre-miR-181c-5p_R+1_1ss10TC	CACATTCATCGCTGTCGGTGGGT	23	gp2
50	ssa-miR-194b-5p	TGTAACAGCATCTCCATATGGA	22	gp2
51	ssa-miR-194c-3p_R-1_2ss20GC21GT	CCAGTGGAGCTGCTGTTATCT	21	gp2
52	dre-miR-194a_R+2	TGTAACAGCAACTCCATGTGGAT	23	gp2
53	ola-miR-194-3p_1ss20CT	CCAGTGGAGGTGCTGTTACTTG	22	gp2
54	ola-miR-199a-5p_R+3	CCCAGTGTTCAGACTACCTGTTCCT	25	gp2
55	ola-miR-199a-3p_L+1	AACAGTAGTCTGCACATTGGTTA	23	gp2
56	fru-mir-199-3-p3	ACAGTAGTCTGCACATTGGTTAAG	24	gp2
57	ssa-mir-203a-1-p5_1ss11AG	AGTGGTTCTAGATAGTTCAACA	22	gp2

58	ssa-miR-203a-3p_1ss21TA	GTGAAATGTTTAGGACCACTAG	22	gp2
59	hsa-miR-203a-3p	GTGAAATGTTTAGGACCACTAG	22	gp2
60	dre-miR-204-5p_L+1	TTTCCCTTTGTCATCCTATGCCT	23	gp2
60	dre-miR-204-5p_L+1	TTTCCCTTTGTCATCCTATGCCT	23	gp2
61	dre-miR-204-5p_L+1R-1	TTTCCCTTTGTCATCCTATGCC	22	gp2
62	ssc-miR-206	TGGAATGTAAGGAAGTGTGTGA	22	gp2
63	aca-miR-449a_R-1_2ss11TA21GA	TGGCAGTGTAATGTTAGCTGA	21	gp2
64	dre-miR-455-2-5p	GTATGTGCCCTTGGACTACATT	22	gp2
64	dre-miR-455-2-5p	GTATGTGCCCTTGGACTACATT	22	gp2
65	dre-miR-1788-5p	GGCTTGTTTTAAGTTGCCTGCG	22	gp2
66	dre-miR-1788-3p_2ss18TC21GT	CAGGCAGCTAAAGCAAGCCTT	21	gp2
67	hsa-mir-5100-p5_1ss18TC	GATCCCAGCGGTGCCTCCA	19	gp2
68	hsa-mir-7641-1-p3_1ss5TC	TGGTCAGTACTTGGATGG	18	gp2
69	pma-let-7c	TGAGGTAGTAGATTGTATGGTT	22	gp2
70	dre-let-7j_1ss10TA	TGAGGTAGTAGTTTGTACAGTT	22	gp2
71	oha-let-7c-5p_R+1	TGAGGTAGTAGGTTGTATGGTTG	23	gp2
1	aca-miR-10a-5p_1ss18TC	TACCCTGTAGATCCGAACTTGTG	23	gp3
2	bta-miR-15b	TAGCAGCACATCATGGTTTACA	22	gp3
3	oan-miR-16b-5p_L+1_1ss22TA	CTAGCAGCACGTAAATATTGGAG	23	gp3
4	mmu-miR-20b-5p_R-1_1ss12TC	CAAAGTGCTCACAGTGCAGGTA	22	gp3
5	dre-miR-21_R+2	TAGCTTATCAGACTGGTGTTGGCAT	25	gp3
6	hsa-miR-21-5p_R+1	TAGCTTATCAGACTGATGTTGAC	23	gp3
7	pma-miR-23b_R+1_1ss23CT	ATCACATTGCCAGGGATTACCATT	24	gp3
8	dre-miR-24_R+2_2	TGGCTCAGTTCAGCAGGAACAGTT	24	gp3
9	xtr-miR-25_L+4	GTGGCATTGCACTTGTCTCGGTCTGA	26	gp3
10	ssa-miR-26d-5p_1ss22TA	TTCAAGTAATCTAGGATAGGCA	22	gp3

11	dre-miR-27b-3p_R+1_1ss22AT	TTCACAGTGGCTAAGTTCTGCTT	23	gp3
12	aca-miR-30a-5p_R+4_1ss13CT	TGTAAACATCCTTGACTGGAAGCTTT	26	gp3
13	aca-miR-30a-3p_R+1_1ss22CT	CTTTCAGTCGGATGTTTGCAGTT	23	gp3
12	aca-miR-30a-5p_R+4_1ss13CT	TGTAAACATCCTTGACTGGAAGCTTT	26	gp3
13	aca-miR-30a-3p_R+1_1ss22CT	CTTTCAGTCGGATGTTTGCAGTT	23	gp3
14	gga-miR-30b-5p_R+1_1ss22TC	TGTAAACATCCTACACTCAGCCA	23	gp3
15	pma-miR-33a_R-1_1ss21AT	GTGCATTGTAGTTGCATTGTT	21	gp3
16	bta-miR-34c_R+1	AGGCAGTGTAGTTAGCTGATTGC	23	gp3
17	gga-miR-34b-5p_L-1R+1	AGGCAGTGTAGTTAGCTGATTGC	23	gp3
18	cgr-miR-34c-5p	AGGCAGTGTAGTTAGCTGATTGC	23	gp3
19	dre-miR-92a-3p_R+2	TATTGCACTTGTCCCGGCCTGTAA	24	gp3
20	dre-miR-93_R+2	AAAAGTGCTGTTTGTGCAGGTATT	24	gp3
21	ccr-miR-99_R+3	AACCCGTAGATCCGATCTTGTGAA	24	gp3
22	cfa-miR-99b	CACCCGTAGAACCGACCTTGCG	22	gp3
23	gga-miR-99a-5p_R+2	AACCCGTAGATCCGATCTTGTGAA	24	gp3
24	mmu-miR-101c_L+1R+2	TACAGTACTGTGATAACTGATC	22	gp3
25	ola-miR-106a_R+2	TAAAGTGCTTACAGTGCAGGTAT	23	gp3
26	bta-miR-106b_R-1_1ss10GT	TAAAGTGCTTACAGTGCAGA	20	gp3
27	cgr-miR-106b-5p_R-2_1ss10GT	TAAAGTGCTTACAGTGCAGA	20	gp3
28	rno-miR-122-5p_L+3	ATCTGGAGTGTGACAATGGTGTTTG	25	gp3
29	sha-miR-125a_R+2	TCCCTGAGACCCTAACTTGTGAAA	24	gp3
30	pma-miR-125-5p_R+1_1ss22AT	TCCCTGAGACCCTAACTTGTGTT	23	gp3
31	oan-miR-126-3p_R+1_1ss22GC	TCGTACCGTGAGTAATAATGCCT	23	gp3
31	oan-miR-126-3p_R+1_1ss22GC	TCGTACCGTGAGTAATAATGCCT	23	gp3
32	aca-miR-128-3p_R+2	TCACAGTGAACCGGTCTCTTTAT	23	gp3
33	oan-miR-139-5p_R+1_1ss22GA	TCTACAGTGCATGTGTCTCCAAG	23	gp3

34	pma-miR-140_R+1_1ss22GC	CAGTGGTTTTACCCTATGGTACC	23	gp3
35	dre-miR-141-3p	TAACACTGTCTGGTAACGATGC	22	gp3
36	mdo-miR-144-3p_L+1R-1	CTACAGTATAGATGATGTACTG	22	gp3
37	bta-miR-146b	TGAGAACTGAATTCCATAGGCTGT	24	gp3
38	ola-miR-146a-5p_1ss24TA	TGAGAACTGAATTCCATAGATGGAA	25	gp3
39	cgr-miR-146b-5p_R+1	TGAGAACTGAATTCCATAGGCTGT	24	gp3
40	ssa-miR-148b-3p_1ss22TA	TCAGTGCATTACAGAACTTTAA	22	gp3
41	bta-miR-151-5p	TCGAGGAGCTCACAGTCTAGT	21	gp3
41	bta-miR-151-5p	TCGAGGAGCTCACAGTCTAGT	21	gp3
42	hhi-miR-181b_R+2	AACATTCATTGCTGTCGGTGGGTTGT	26	gp3
43	gga-miR-181b-2-3p_L+1_1ss21AG	CTCACTGATCAATGAATGCAGA	22	gp3
44	bta-miR-186_R+1	CAAAGAATTCTCCTTTTGGGCTT	23	gp3
45	aca-miR-191-5p	CAACGGAATCCCAAAAGCAGCTG	23	gp3
45	aca-miR-191-5p	CAACGGAATCCCAAAAGCAGCTG	23	gp3
46	oan-miR-194-5p_R+2	TGTAACAGCAACTCCATGTGGATT	24	gp3
47	tni-miR-199_R+2	CCCAGTGTTCAGACTACCTGTTCCA	25	gp3
48	ssa-miR-199a-3p_R+2	ACAGTAGTCTGCACATTGGTTTT	23	gp3
49	bta-miR-199c_L-1R+2	ACAGTAGTCTGCACATTGGCA	21	gp3
50	aca-miR-200b-3p_R+2	TAATACTGCCTGGTAATGATGAAT	24	gp3
50	aca-miR-200b-3p_R+2	TAATACTGCCTGGTAATGATGAAT	24	gp3
51	cfa-miR-203_L-1R+1	TGAAATGTTTAGGACCACTAGT	22	gp3
52	mdo-miR-210-3p_L-1R+3	CTGTGCGTGTGACAGCGGCTACT	23	gp3
53	xtr-miR-215_R-1_2ss13AT20CA	ATGACCTATGAATTGACAGA	20	gp3
54	ssa-miR-192a-3p_R+2_1ss11AC	CCTGTCAGTTCTGTAGGCCACTGT	24	gp3
55	aca-miR-218-5p_R+2	TTGTGCTTGATCTAACCATGTGTT	24	gp3
56	ola-miR-222_R+7	AGCTACATCTGGCTACTGGGTCTCCT	26	gp3

57	aca-miR-338-3p_R+2	TCCAGCATCAGTGATTTTGTTAA	23	gp3
57	aca-miR-338-3p_R+2	TCCAGCATCAGTGATTTTGTTAA	23	gp3
58	cfa-miR-340	TTATAAAGCAATGAGACTGATT	22	gp3
59	hsa-miR-340-5p	TTATAAAGCAATGAGACTGATT	22	gp3
60	bta-miR-378	ACTGGACTTGGAGTCAGAAGGC	22	gp3
61	cfa-miR-429_R+2	TAATACTGTCTGGTAATGCCGTTT	24	gp3
62	aca-miR-456_R+2	CAGGCTGGTTAGATGGTTGTCTT	23	gp3
63	bta-miR-484	TCAGGCTCAGTCCCCTCCCGAT	22	gp3
64	dre-miR-737-5p_R+1_1ss2TC	GCTTTTTTAGGTTTTGATTTTT	22	gp3
65	bta-miR-1260b	ATCCCACCACTGCCACCA	18	gp3
66	oan-miR-1386_1ss18AT	CTCCTGGCTGGCTCGCCT	18	gp3
67	ola-miR-1388-5p_R+2	AGGACTGTCCAACCTGAGAATGTA	24	gp3
68	ola-miR-1388-3p_R+6	ATCTCAGGTTCGTCAGCCCATGTT	24	gp3
69	bta-miR-2478_L+2	TCGTATCCCACTTCTGACACCA	22	gp3
70	mmu-miR-3964_L+1_1ss6GA	CATAAAGTAGAAAGCACTAAA	21	gp3
71	hsa-miR-4286_L+2R+1	GAACCCCACTCCTGGTACCA	20	gp3
72	hsa-miR-4443_R+1	TTGGAGGCGTGGGTTTTT	18	gp3
73	hsa-miR-4448_1ss6CG	GGCTCGTTGGTCTAGGGGTA	20	gp3
74	hsa-miR-6087_R+1_1ss11GA	TGAGGCGGGGAGGCGAGCC	19	gp3
75	mmu-miR-6240_R-7_1ss1CA	ACAAAGCATCGCGAAGGCC	19	gp3
76	ssa-miR-7132a-5p_R+1	GACTTGGTCAAAGCTCCTCAGTT	23	gp3
77	ipu-miR-7550	ATCCGGCTCGAAGGACCA	18	gp3
78	hsa-miR-7641_L+1_1ss2TC	TCTGATCTCGGAAGCTAAGC	20	gp3
79	hsa-miR-7977_1ss6AG	TTCCCGGCCAACGCACCA	18	gp3
80	pol-let-7a-5p_R+3_1ss17AG	TGAGGTAGTAGGTTGTGTGGTTTGT	25	gp3
81	hsa-let-7d-5p	AGAGGTAGTAGGTTGCATAGTT	22	gp3

82	ccr-let-7j_R+2	TGAGGTAGTTGTTTGTACAGTTGT	24	gp3
83	mmu-let-7j_1ss8TG	TGAGGTAGTAGTTTGTGCTGTTAT	24	gp3
1	PC-5p-48743_50	AGACTATTTGGAAACCACTGGA	22	gp4
2	PC-3p-42938_70	AGTGGTTTCCAAATAGTCTGAT	22	gp4
1	PC-5p-48743_50	AGACTATTTGGAAACCACTGGA	22	gp4
2	PC-3p-42938_70	AGTGGTTTCCAAATAGTCTGAT	22	gp4
3	PC-5p-27517_164	TACATGCAGAGGTGGAGCAAGA	22	gp4
4	PC-3p-40786_80	TTGCTCCGCCTCTGCATGTACA	22	gp4
4	PC-3p-40786_80	TTGCTCCGCCTCTGCATGTACA	22	gp4
3	PC-5p-27517_164	TACATGCAGAGGTGGAGCAAGA	22	gp4
5	PC-5p-36247_102	GAGTTCATGTGAATCAGAATCA	22	gp4
6	PC-3p-678_2951	ATTCTGATTCACATGAACTCGT	22	gp4
6	PC-3p-678_2951	ATTCTGATTCACATGAACTCGT	22	gp4
7	PC-5p-27289_165	AACTCCAGTCCTCGAGGGCATGG	23	gp4
8	PC-3p-31363_133	CGGCCTTTGAGGAATGGAGTTT	22	gp4
9	PC-5p-57955_26	AAACTCCATTCCTCGAAGGCCG	22	gp4
10	PC-3p-27059_167	ATGCCCTCGAGGACTGGAGTTT	22	gp4
11	PC-5p-6223_665	CCTAGCAGCTGACTTAGAAC	20	gp4
11	PC-5p-6223_665	CCTAGCAGCTGACTTAGAAC	20	gp4
12	PC-5p-45063_62	AAGGATAACTACAACTGTACTT	22	gp4
12	PC-5p-45063_62	AAGGATAACTACAACTGTACTT	22	gp4
13	PC-5p-8690_526	GATGTTGAGTATCAAACTGTAT	22	gp4
13	PC-5p-8690_526	GATGTTGAGTATCAAACTGTAT	22	gp4
14	PC-3p-59008_24	CACCTAACATGTTAGCATTAGC	22	gp4
15	PC-3p-72473_9	TGAGAATGTGACTAACTGAAC	21	gp4
16	PC-5p-50252_45	ACGAGAATGATTGATTGATGTG	22	gp4

17	PC-3p-52484_39	TTCGACATCTCTGGTTTAGACT	22	gp4
18	PC-3p-31495_132	GCTCATCAGGACGTAGCGTTTT	22	gp4
19	PC-3p-38688_90	GCATACTGTAAGTGCTCTTTGT	22	gp4
20	PC-3p-41259_77	TGGCCATTAACTGCTAACCTTC	22	gp4
21	PC-5p-39669_85	GAAACAAAGGTGTGTACTCTC	21	gp4
22	PC-5p-44104_66	CGTGGTCTGTGGAGCCGTGCAGT	23	gp4
23	PC-5p-36298_102	AAACAAAGGTGTGTACTCTCC	21	gp4
24	PC-3p-40655_80	TAAGATTGATGAAACCTGCAGT	22	gp4
25	PC-3p-45713_60	CTGGGATGGTCCTGGATCAGGT	22	gp4
26	PC-5p-2385_1201	TCTTCCATGCACTTTGATGACT	22	gp4
27	PC-5p-33148_121	TGTAGAGCAGACTGGATTCTCT	22	gp4
28	PC-3p-54054_35	AAAGACACCTAGAATACAGTAT	22	gp4
29	PC-3p-31169_134	TGCCCCGCGGAGTGTGAGTGA	21	gp4
30	PC-3p-5333_737	TGTTGTTGCAATAGTAATCC	20	gp4
31	PC-5p-44577_64	CGTTCTCGGCAGGTACGTACTC	22	gp4
32	PC-5p-22578_213	AAAGCATTGCTCTACCTGCAC	21	gp4
33	PC-5p-71414_10	CTCCATCCAAGCTTTTGT	18	gp4
34	PC-3p-50929_43	TGGAAGTGTCAGAAATTCTGAGT	23	gp4
35	PC-5p-9153_506	AAGACGACTTCCTGTTCTGATC	22	gp4
36	PC-3p-60384_22	AAACACATGCAGCTGATCTGC	21	gp4
37	PC-5p-51716_41	TTGACTTTCTGCCCCGACGGC	21	gp4
38	PC-3p-11630_419	ATGAGGAAAAGAAGTTAGGAGA	22	gp4
39	PC-3p-50779_43	GGCCTTCGAGGACTGGAGTTTGT	23	gp4
40	PC-3p-67453_13	AGAAATGATGGTACAGAG	18	gp4
41	PC-5p-48963_49	CGTTCTCGGCAGGTACGTACT	21	gp4
42	PC-3p-54917_33	CATCATTCACTCTAAGCTGACT	22	gp4

43	PC-5p-7717_573	TTTTAAGCAGGAGGTGTC	18	gp4
44	PC-5p-20509_240	TTCCCTCCGAAGTTTCCC	18	gp4
45	PC-5p-48837_49	ATGACACCCAGTTTGTTCTCT	21	gp4
46	PC-5p-55836_31	TGTGTCTCTGGCAAGATGATTT	22	gp4

miRNA_Index mature miRNA

miR_name The miR_name is composed of the 1st known miR name in a cluster, a underscore, and a matching annotation: such as

L-n means the miRNA_seq (detected) is n base less than known rep_miRSeq in the left side;

R-n means the miRNA_seq (detected) is n base less than known rep_miRSeq in the right side;

L+n means the miRNA_seq (detected) is n base more than known rep_miRSeq in the left side;

R+n means the miRNA_seq (detected) is n base more than known rep_miRSeq in the right side;

2ss5TC13TA means 2 substitution (ss), which are T->C at position 5 and T->A at position 13

if there is no matching annotation, the miRNA_seq (detected) is exactly same as known rep_miRSeq.

New discovered 5p/3p sequence has been annotated as p3/p5: which is directly differentiate with the reported sequences,

miR_seq is a read at 5' or 3' of maximum mapped score in each cluster.

len miR_seq length

Sequence in miRbase New, miRNA identified in this study and not reported in miRBase,mainly for new reported 5p or 3p sequence; Diff, confirming miRNA sequences in miRBase, but different sequences are reported in our study; and Yes, confirming miRNA sequences in miRBase.

gp1:Reads were mapped to miRNAs/pre-miRNAs of specific species in miRbase and the pre-miRNAs were further mapped to genome & EST.

gp2:Reads were mapped to miRNAs/pre-miRNAs of selected species in miRbase and the mapped pre-miRNAs were not further mapped to genome, but the reads (and of course the miRNAs of the pre-miRNAs) were mapped to genome. The extended genome sequences from the genome loci may form hairpins.

gp3:Reads were mapped to miRNAs/pre-miRNAs of selected species in miRbase and the mapped pre-miRNAs were not further mapped to genome, and the reads were not mapped to genome, either. but the reads were mapped to the miRNAS(Matures)

gp4:Reads were not mapped to pre-miRNAs of selected species in miRbase. But the reads were mapped to genome & the extended genome sequences from genome may form hairpins.

Table S3 List of miRNA member in each family of GIFT (*Oreochromis_niloticus*)

AC	ID	MI
MIPF0000001	mir-17	dre-miR-18a,dre-miR-18b-5p_1ss11TC,dre-miR-18b-3p_L+1R-2_1ss15CT,dre-miR-93_R+2,mmu-miR-20b-5p_R-1_1ss12TC, bta-miR-106b_R-1_1ss10GT,aca-miR-18b-5p_R-3,ola-miR-20a_R+2,ola-mir-20a-1-p3,ola-miR-106a_R+2,ola-miR-17_R+4,ola-mir-17-p3, cgr-miR-106b-5p_R-2_1ss10GT,ssa-miR-106b-5p_L-1R+1,ssa-miR-106b-5p_R+1_2ss1AC12CT,ssa-miR-17-3-3p_L-1_2ss19CA20TC, ssa-miR-17-5p_R-1,ssa-miR-18a-5p,oha-miR-18a-3p_R-1
MIPF0000002	let-7	hsa-let-7d-5p,dre-let-7a,dre-let-7a-1-p3_1ss5AT,dre-let-7b,dre-let-7c-5p,dre-let-7c-3p,dre-let-7d-5p,dre-let-7g,dre-let-7g-1-p3,dre-let-7i, tni-let-7j_1ss11TG,tni-let-7j-p3_1ss22CT,dre-let-7j_1ss10TA,bta-mir-3596-p5_1ss4CT,pma-let-7c,aca-let-7b-3p_R+1,ola-let-7e_R+4, ola-let-7e-p3,ola-let-7a,ola-let-7a-3p_R+1,pol-let-7a-5p_R+3_1ss17AG,pol-let-7d-3p,ccr-let-7j_R+2,ipu-let-7a,ipu-let-7a-5-p3,ipu-let-7b_R+1, ipu-let-7d,ssa-let-7f-5p_1ss19TA,ssa-let-7f-3p_1ss22CT,ssa-let-7g-5p,ssa-let-7g-3p_1ss22CT,ssa-let-7h-5p,ssa-let-7h-3p, ssa-let-7i-2-3p_1ss22CT,ssa-let-7a-5p,ssa-let-7a-3p,ssa-let-7a-3-3p,ssa-let-7a-4-3p_2ss9TC15GA,ssa-let-7b-3p_1ss3GA,ssa-let-7e-5p, ssa-let-7e-3p_1ss22CT,oha-let-7c-5p_R+1
MIPF0000005	mir-30	mmu-miR-30d-5p_R-2,mmu-miR-30d-3p_R-2_1ss10AG,gga-miR-30b-5p_R+1_1ss22TC,dre-miR-30c-5p_R+2,dre-miR-30e-5p_R+2, dre-miR-30e-3p,fru-miR-30c,fru-mir-30c-p3_1ss6GA,tni-mir-30c-p3_2ss5GA23GT,aca-miR-30a-5p_R+4_1ss13CT, aca-miR-30a-3p_R+1_1ss22CT,ola-miR-30c_L+1R-3,ssa-miR-30a-5p,ssa-miR-30a-4-3p,ssa-miR-30c-5p,ssa-miR-30c-3p, ssa-miR-30d-5p_2ss13TC19AG,ssa-miR-30d-2-3p_1ss1TC,ssa-miR-30e-5p
MIPF0000006	mir-15	fru-miR-15a_R+1,fru-mir-15a-p3,bta-miR-15b,oan-miR-16b-5p_L+1_1ss22TA,ssa-miR-16a-5p,ssa-miR-16a-3p_R+1_1ss10TA, ssa-miR-16b-5p_R-1_1ss21TC,ssa-miR-16b-3p_R-1_1ss13TA,ssa-miR-15c-5p,ssa-mir-15c-2-p3_1ss11CA
MIPF0000007	mir-181	gga-miR-181b-2-3p_L+1_1ss21AG,dre-miR-181b-5p_R+1,dre-miR-181b-3p_R-2,dre-miR-181c-5p_R+1_1ss10TC,ola-miR-181b-5p_R-1, ola-miR-181b-3p_R+1,ipu-miR-181a_R+1,ipu-mir-181a-5-p3,hhi-miR-181b_R+2,ssa-miR-181a-2-3p,ssa-miR-181a-5p,ssa-miR-181a-5-3p, ssa-miR-181b-5p_R-1_1ss18CG
MIPF0000009	mir-29	dre-miR-29b,tni-miR-29b,bta-miR-29d-3p_R-3,aca-miR-29b_R+1_1ss10TA,ola-mir-29b-1-p5_2ss1GA18GT,ssa-miR-29a-5p, ssa-miR-29b-1-5p_1ss18TC,ssa-miR-29b-3p
MIPF0000011	mir-19	rno-miR-19a-3p_R-2,dre-miR-19a-5p_L+1R-1,dre-miR-19a-3p_2ss11TC23AT,ola-mir-19d-p5,ola-miR-19d_R+1,ssa-miR-19c-3p, ssa-miR-19c-4-5p_R+1_1ss17TA

MIPF0000013	mir-25	dre-miR-25-5p_2ss18GA19CT,dre-miR-25-3p,dre-miR-92a-3p_R+2,fru-mir-92-2-p5_1ss12AG,xtr-miR-25_L+4,ola-mir-92a-1-p5, ipu-miR-92a,ssa-miR-92a-3p,ssa-miR-92b-3p
MIPF0000014	mir-9	dre-miR-9-5p_R-2,dre-miR-9-3p_R+1,dre-miR-9-4-3p_1ss19TA,xtr-miR-9a-5p_R-2,xtr-miR-9a-3p_R+1,ola-miR-9b-5p_R-2, ola-miR-9a-3p_L-1R+2,ssa-miR-9a-2-3p,ssa-miR-9a-5p_R-2,ssa-miR-9a-3-3p,ssa-miR-9a-4-3p_L-1R+1
MIPF0000019	mir-8	cfa-miR-429_R+2,dre-miR-429a,dre-miR-141-3p,aca-miR-200b-3p_R+2,ssa-miR-200a-3p,ssa-miR-200a-2-5p,ssa-miR-200b-5p_1ss10TC, ssa-miR-200b-3p
MIPF0000022	mir-7	tni-miR-7_R+1,ssa-miR-7a-5p_R+1,ssa-miR-7a-3-3p_R-2,ssa-miR-7a-5p
MIPF0000024	mir-103	tni-mir-103-p5,dre-miR-107b_R-1,ipu-miR-103,ssa-miR-103-5p_2ss11TC13AG,ssa-miR-103-3p,ssa-miR-107-5p,ssa-miR-107-3p
MIPF0000026	mir-218	dre-miR-218a,dre-miR-218b_R-1,aca-miR-218-5p_R+2,ola-miR-218b_R+2
MIPF0000027	mir-23	dre-mir-23a-1-p5,dre-miR-23a-3p_R-1,dre-miR-23a-3-5p,pma-miR-23b_R+1_1ss23CT,ipu-miR-23a,ssa-miR-23b-5p_R+1, ssa-miR-23b-3p_R-3
MIPF0000028	mir-135	dre-miR-135c,ola-miR-135b_R+4,ssa-miR-135a-5p_R-1,ssa-miR-135a-3p_L+1R-1,ssa-miR-135b-5p_R-1,ssa-miR-135b-3-3p_R+1_1ss11CT
MIPF0000029	mir-133	dre-miR-133a-5p,dre-miR-133a-3p_L-1R+1,dre-miR-133b-3p_R-1,oha-miR-133b-3p
MIPF0000031	mir-196	dre-miR-196a-5p
MIPF0000033	mir-10	gga-miR-99a-5p_R+2,dre-miR-10a-5p,dre-miR-10a-5p_L-1,dre-miR-10b-5p_R-1,dre-miR-10b-2-3p,dre-miR-125a,dre-miR-125b-5p_R+1, dre-miR-125b-5p,dre-miR-125b-2-3p_L+1_1ss15CT,tni-miR-10c,tni-mir-10c-p3,cfa-miR-99b,pma-miR-125-5p_R+1_1ss22AT, aca-miR-10a-5p_1ss18TC,ola-miR-100_R+3,ola-mir-100-1-p3_1ss9CT,ola-mir-100-2-p3,ola-miR-10d,sha-miR-125a_R+2,ccr-miR-99_R+3, ipu-miR-100,ssa-miR-10b-5p,ssa-miR-125a-5p,ssa-miR-125a-2-3p,ssa-miR-125b-1-3p_R+1,ssa-miR-125b-5p_R-1,ssa-miR-125b-2-3p, ssa-miR-99-5p
MIPF0000034	mir-130	dre-miR-130b_R+2,dre-miR-130c-5p,dre-miR-130c-3p,dre-miR-301c-3p_R+1,xtr-mir-130c-p5,ssa-miR-130a-5p,ssa-miR-130a-2-3p_R-1, ssa-miR-130d-3p_R+1,ssa-miR-301d-5p_L+1,ssa-miR-301d-3p_1ss1CT
MIPF0000036	mir-27	dre-miR-27b-3p_R+1,dre-miR-27b-3p_R+1_1ss22AT,dre-miR-27d_R-1_1ss19TG,dre-miR-27e_1ss22GA,ola-miR-27c-5p_L+1R+1_1ss10GA, ola-miR-27c-3p_R+1,ola-miR-27a_R+2_1ss19CT,ccr-miR-27c-3p_R+1,ssa-miR-27b-5p,ssa-miR-27b-3p,ssa-miR-27c-5p_R+1, ssa-miR-27c-3p
MIPF0000038	mir-1	dre-miR-1,ssc-miR-206,ssa-miR-1-3p,ssa-miR-1-4-5p,ssa-miR-206-3p,oha-miR-1a-3p
MIPF0000039	mir-34	gga-miR-34b-5p_L-1R+1,dre-miR-34a,dre-mir-34a-p3,bta-miR-34c_R+1,cgr-miR-34c-5p

MIPF0000040	mir-199	dre-miR-199-5p,dre-miR-199-3p_L-1,dre-miR-199-3-3p_1ss10CT,fru-mir-199-3-p3,tni-miR-199_R+2,ola-miR-199a-5p_R+3, ola-miR-199a-3p_L+1,ssa-miR-199a-3p_R+2,ssa-miR-199a-5p,ssa-miR-199a-3p
MIPF0000041	mir-24	dre-miR-24_R+2_1,dre-miR-24_R+2_2,fru-miR-24-5p_L-1,fru-miR-24-3p_R+1,cfa-miR-24_R-3,pma-miR-24_R+1, ola-mir-24a-3-p5_1ss19TA, ola-miR-24b-5p,ipu-miR-24_1ss23GT,ipu-miR-24b,ssa-miR-24a-4-5p,ssa-miR-24a-3p_R+3
MIPF0000042	mir-204	dre-miR-204-5p_L+1,dre-miR-204-5p_L+1R-1,tni-miR-204a,xtr-miR-204,ssa-miR-204-5p
MIPF0000043	mir-26	hsa-miR-26a-2-3p_R+1,gga-miR-26a-3p_R-1_2ss6TG11GA,dre-miR-26a-5p_L+1R-1,dre-miR-26a-3p_R+1,dre-miR-26b_R+1,ipu-miR-26a, ipu-mir-26a-1-p3_1ss8GC,hhi-miR-26_R+1,ssa-miR-26a-5p,ssa-miR-26a-4-3p,ssa-mir-26a-5-p3_1ss11GC,ssa-miR-26a-6-3p_2ss8AG19TC, ssa-miR-26b-5p,ssa-miR-26d-5p_L+1_1ss13TC,ssa-miR-26d-5p_2ss10TC12TC,ssa-miR-26d-5p_1ss22TA
MIPF0000046	mir-101	dre-miR-101a,ssa-miR-101a-5p_R-1_1ss12TA,ssa-miR-101b-5p_L+1R-2,ssa-miR-101b-3p_R+1_1ss10AG
MIPF0000048	mir-128	tni-miR-128_R-1,aca-miR-128-3p_R+2,ola-miR-128_R+1
MIPF0000050	mir-153	dre-miR-153a-3p,dre-miR-153c-3p_R+1,tni-miR-153a,ssa-miR-153a-3p,ssa-miR-153a-2-5p_3ss5CT6TC12AG,ssa-miR-153a-3p_1ss21TG
MIPF0000051	mir-221	ola-miR-222_R+7,pol-miR-221-5p_R-4,pol-miR-221-3p,ssa-miR-221-3p_R-1_1ss5AG,ssa-miR-221-5p,ssa-miR-222a-5p_R-2, ssa-miR-222a-3p_R-1,ssa-miR-222b-5p,ssa-miR-222b-3p_R+1
MIPF0000053	mir-22	dre-miR-22a-5p,dre-miR-22a-3p,dre-miR-22b-5p_R-1,dre-miR-22b-3p,ola-mir-22-2-p5,ola-miR-22,oha-miR-22a,oha-miR-22a_R-2
MIPF0000054	mir-216	tni-miR-216a,tni-mir-216a-p3,tni-miR-216b,tni-mir-216b-p3
MIPF0000055	mir-194	dre-miR-194a_R+2,tni-miR-194_R+1,oan-miR-194-5p_R+2,ola-miR-194-3p_1ss20CT,ssa-miR-194b-5p, ssa-miR-194c-3p_R-1_2ss20GC21GT
MIPF0000056	mir-148	dre-miR-148_R-1,dre-miR-152_R-1_1ss10GC,dre-miR-152_R-1_2ss10GT21GA,fru-mir-152-p5,ssa-miR-148a-5p_1ss19CA, ssa-miR-148b-3p_1ss22TA,ssa-miR-152-3p
MIPF0000057	mir-28	bta-miR-151-5p
MIPF0000058	mir-205	tni-mir-205-p3,ipu-miR-205_R-1,ssa-miR-205b-5p
MIPF0000059	mir-184	dre-miR-184_R-1,ola-miR-184-3p
MIPF0000060	mir-21	hsa-miR-21-5p_R+1,dre-miR-21_1ss23CA,dre-miR-21_R+2,ola-mir-21-1-p3_1ss6AG,ssa-miR-21b-5p,ssa-miR-21b-3p_R-1
MIPF0000061	mir-365	dre-miR-365,ssa-miR-365-5p_R+3
MIPF0000062	mir-214	ssa-miR-214-5p,ssa-miR-214-3p_L-1R-1,ssa-miR-214-3p_L-1_1ss23AT
MIPF0000063	mir-192	xtr-miR-215_R-1_2ss13AT20CA,ssa-miR-192a-5p,ssa-miR-192a-3p_R+2_1ss11AC,ssa-miR-192b-5p

MIPF0000064	mir-31	aca-miR-31-5p_R+2_1ss11TC
MIPF0000065	mir-132	dre-miR-132-3p,dre-miR-212,ssa-miR-132-5p,ssa-miR-132-3p,ssa-miR-212a-5p_1ss10TC,ssa-miR-212a-3p,ssa-miR-212b-5p
MIPF0000066	mir-183	dre-miR-183-5p_R-1,ssa-miR-183-5p
MIPF0000067	mir-223	ola-mir-223-p5,ola-miR-223_R+2
MIPF0000070	mir-33	tgu-miR-33-5p,pma-miR-33a_R-2,pma-miR-33a_R-1_1ss21AT,ola-miR-33_R+2,ssa-miR-33b-5p_R-1,ssa-miR-33a-3p
MIPF0000072	mir-96	ssa-miR-96-5p
MIPF0000073	mir-129	dre-miR-129-3p,ssa-miR-129-3p
MIPF0000075	mir-138	dre-miR-138-5p_R+1,ssa-miR-138-5p
MIPF0000076	mir-190	dre-miR-190a_R+1,ssa-miR-190a-3p
MIPF0000077	mir-217	tni-miR-217_R+1,tni-mir-217-p3
MIPF0000078	mir-187	dre-miR-187_R+3
MIPF0000079	mir-145	ssa-miR-145-5p,ssa-miR-145-3p_L+2R-1
MIPF0000082	mir-193	dre-miR-193a-5p,dre-miR-193a-3p
MIPF0000084	mir-142	mmu-miR-142a-5p,dre-miR-142a-5p,dre-miR-142a-3p_R-1,xtr-miR-142-5p_R+3,xtr-miR-142-3p_L+1R-1,ipu-miR-142_L+1R-1
MIPF0000085	mir-140	dre-miR-140-5p,dre-miR-140-3p_L-1
MIPF0000086	mir-210	dre-miR-210-5p,dre-miR-210-3p_1ss22AT,mdo-miR-210-3p_L-1R+3
MIPF0000093	mir-144	mdo-miR-144-3p_L+1R-1,ssa-miR-144-5p_R-1_1ss11AT,ssa-miR-144-3p_R+1
MIPF0000094	mir-143	ssa-miR-143-5p_R+1,ssa-miR-143-3p_R+1
MIPF0000095	mir-122	rno-miR-122-3p_L+1R+1_1ss10CT,rno-miR-122-5p_L+3,dre-miR-122,xtr-miR-122_L+1R-1,ssa-miR-122-2-3p_R-1
MIPF0000097	mir-338	aca-miR-338-3p_R+2,ssa-miR-338a-3-5p,ssa-miR-338a-3p_R-1
MIPF0000103	mir-146	bta-miR-146b,ola-miR-146a-5p_1ss24TA,cgr-miR-146b-5p_R+1,ssa-miR-146a-5p,ssa-miR-146a-3p_R-2
MIPF0000106	mir-137	ssa-miR-137-3p_R-2
MIPF0000108	mir-203	hsa-miR-203a-3p,cfa-miR-203_L-1R+1,ssa-miR-203a-3p,ssa-mir-203a-1-p5_1ss11AG,ssa-miR-203a-3p_1ss21TA, ssa-miR-203a-2-5p_1ss10TC
MIPF0000109	mir-186	bta-miR-186_R+1
MIPF0000111	mir-489	dre-miR-489_L-1,ssa-miR-489-5p

MIPF0000114	mir-375	ssa-miR-375-3p
MIPF0000115	mir-126	oan-miR-126-3p_R+1_1ss22GC,ssa-miR-126-5p,ssa-miR-126-3p
MIPF0000116	mir-182	dre-miR-182-5p
MIPF0000117	mir-139	dre-miR-139-5p_R+5,oan-miR-139-3p_R-1_1ss8AC,oan-miR-139-5p_R+1_1ss22GA,ola-miR-139_R+2
MIPF0000129	mir-455	dre-miR-455-5p,dre-miR-455-3p_L-1,dre-miR-455-2-5p,ssa-miR-455-5p,ssa-miR-455-3p_L+1R-1
MIPF0000133	mir-449	aca-miR-449a_R-1_2ss11TA21GA,hhi-miR-449_R-2
MIPF0000134	mir-460	ssa-miR-460-5p,ssa-miR-460-3p,ssa-miR-730a-5p_R-1,ssa-miR-730a-3p
MIPF0000168	mir-378	bta-miR-378
MIPF0000173	mir-499	ssa-miR-499b-5p_R-1
MIPF0000174	mir-454	ssa-miR-454-3p_R-2
MIPF0000179	mir-458	fru-mir-458-p5,fru-miR-458
MIPF0000191	mir-340	hsa-miR-340-5p,cfa-miR-340
MIPF0000194	mir-191	aca-miR-191-5p
MIPF0000197	mir-150	dre-miR-150_R+1_1ss1TA
MIPF0000219	mir-484	bta-miR-484
MIPF0000341	mir-456	aca-miR-456_R+2,ssa-miR-456-3p_1ss22AT
MIPF0000531	mir-1306	dre-miR-1306_R-1
MIPF0000792	mir-1788	dre-miR-1788-5p,dre-miR-1788-3p_2ss18TC21GT
MIPF0000805	mir-1388	ola-miR-1388-5p_R+2,ola-miR-1388-3p_R+6,ssa-miR-1338-5p_R+1,ssa-miR-1338-3p
MIPF0000812	mir-2188	dre-miR-2188-5p_R+1,dre-miR-2188-3p_L+1_2ss13TC14AG
MIPF0001371	mir-727	dre-miR-727-5p,dre-miR-727-3p_R-2
MIPF0001381	mir-1260b	bta-miR-1260b
MIPF0001612	mir-722	dre-miR-722_L-2,ssa-miR-722-5p_L-1R+1
MIPF0001616	mir-724	dre-miR-724,ssa-miR-724-5p
MIPF0001689	mir-737	dre-miR-737-5p_R+1_1ss2TC
MIPF0001705	mir-7147	dre-miR-7147,dre-mir-7147-p3_2ss5AG20CT

MIPF0001710	mir-3618	ipu-miR-3618_L+1,oha-mir-3618-p3
MIPF0001770	mir-2187	dre-miR-2187-5p_R-1,dre-miR-2187-3p_L+1R-1_1ss21AG
MIPF0001825	mir-7641	hsa-mir-7641-1-p3_1ss5TC,hsa-miR-7641_L+1_1ss2TC
MIPF0002098	mir-7132	ssa-miR-7132a-5p_R+1

Table S4 List of DE miRNAs of GIFT (*Oreochromis_niloticus*) in response to high temperature stress

Index	miR_name	miR_seq	up/down	log2 (fold_change)	*P* value (t_test)	Group CO (mean)	Group HTS (mean)	Expression level
1	ssa-miR-1338-5p_R+1	AGGACTGTCCAACCTGAGAATG	down	-1.60	1.42E-03	1,604	529	middle
2	dre-miR-22a-3p	AAGCTGCCAGCTGAAGAACTGT	up	0.39	2.48E-03	223,565	293,692	high
3	ssa-miR-16b-5p_R-1_1ss21TC	TAGCAGCACGTAAATATTGGC	down	-0.50	2.76E-03	9,599	6,772	high
4	tni-let-7j_1ss11TG	TGAGGTAGTTGTTTGTACAGTT	up	0.50	3.37E-03	13,187	18,691	high
5	sha-miR-125a_R+2	TCCCTGAGACCCTAACTTGTGAAA	up	1.14	3.71E-03	47	103	middle
6	ssa-miR-7a-5p	TGGAAGACTAGTGATTTTGTTGT	up	1.06	4.29E-03	54	112	middle
7	dre-miR-24_R+2_1	TGGCTCAGTTCAGCAGGAACAGAA	up	inf	4.92E-03	0	2	low
8	dre-miR-24_R+2_2	TGGCTCAGTTCAGCAGGAACAGTT	up	inf	4.92E-03	0	2	low
9	PC-5p-45063_62	AAGGATAACTACAACTGTACTT	up	2.38	5.65E-03	2	11	middle
10	dre-miR-133b-3p_R-1	TTTGGTCCCCTTCAACCAGCT	up	5.13	5.71E-03	3	115	middle
11	ola-miR-146a-5p_1ss24TA	TGAGAACTGAATTCCATAGATGGAA	up	2.45	7.46E-03	4	24	middle
12	ssa-miR-199a-3p_R+2	ACAGTAGTCTGCACATTGGTTTT	up	0.83	7.71E-03	255	454	middle
13	dre-miR-133a-3p_L-1R+1	TTGGTCCCCTTCAACCAGCTGT	up	3.93	7.93E-03	84	1,280	middle
14	dre-miR-142a-3p_R-1	TGTAGTGTTTCCTACTTTATGG	down	-1.33	9.19E-03	3,584	1,429	middle
15	ipu-miR-142_L+1R-1	TGTAGTGTTTCCTACTTTATGG	down	-1.33	9.19E-03	3,584	1,429	middle
16	oha-miR-133b-3p	TTTGGTCCCCTTCAACCAGCTAT	up	3.91	1.08E-02	3	43	middle
17	ssa-miR-7a-5p_R+1	TGGAAGACTAGTGATTTTGTTGTT	up	1.27	1.09E-02	106	256	middle
18	tni-miR-7_R+1	TGGAAGACTAGTGATTTTGTTGTT	up	1.27	1.09E-02	106	256	middle
19	ssa-miR-7132b-3p	TGAGGCGTTTAGAACAAGTTCA	down	-1.11	1.24E-02	426	197	middle
20	dre-miR-125b-5p_R+1	TCCCTGAGACCCTAACTTGTGAT	up	0.59	1.26E-02	102	153	middle
21	ola-mir-100-2-p3	CAAGCTCGTATCTATAGGTATG	down	-0.44	1.29E-02	94	70	middle
22	aca-miR-200b-3p_R+2	TAATACTGCCTGGTAATGATGAAT	up	2.07	1.34E-02	2	7	low

23	ssa-miR-7132a-5p_R+1	GACTTGGTCAAAGCTCCTCAGTT	down	-0.80	1.36E-02	52	30	middle
24	ssa-miR-730a-5p_R-1	TCCTCATTGTGCATGCTGTGT	down	-1.89	1.36E-02	81	22	middle
25	ssa-miR-125b-5p_R-1	TCCCTGAGACCCTTAACCTGTG	up	0.26	1.39E-02	1,217	1,460	middle
26	dre-miR-125a	TCCCTGAGACCCTTAACCTGTG	up	0.26	1.39E-02	1,217	1,460	middle
27	ssa-miR-26a-4-3p	CCTATTCTTGATTACTTGTTTC	down	-0.94	1.59E-02	87	45	middle
28	dre-miR-140-3p_L-1	ACCACAGGGTAGAACCACGGAC	up	0.77	1.65E-02	1,493	2,545	middle
29	ola-miR-199a-3p_L+1	AACAGTAGTCTGCACATTGGTTA	up	0.99	1.67E-02	158	314	middle
30	xtr-miR-122_L+1R-1	CTGGAGTGTGACAATGGTGTTTG	up	0.69	1.96E-02	402	648	middle
31	dre-let-7d-5p	TGAGGTAGTTGGTTGTATGGTT	up	0.57	2.23E-02	1,947	2,897	middle
32	ipu-let-7d	TGAGGTAGTTGGTTGTATGGTT	up	0.57	2.23E-02	1,947	2,897	middle
33	ssa-miR-7132b-5p	GACTTGGTCAAAGCTCCTCAGC	down	-1.73	2.27E-02	1,783	539	middle
34	ssa-miR-1-4-5p	ACATACTTCTTTATATGCCCATA	up	inf	2.28E-02	0	11	middle
35	PC-3p-41259_77	TGGCCATTAACTGCTAACCTTC	up	1.55	2.37E-02	7	19	middle
36	dre-miR-142a-5p	CATAAAGTAGAAAGCACTACT	down	-1.37	3.01E-02	6,411	2,477	high
37	mmu-miR-142a-5p	CATAAAGTAGAAAGCACTACT	down	-1.37	3.01E-02	6,411	2,477	high
38	dre-miR-122	TGGAGTGTGACAATGGTGTTTG	up	0.48	3.01E-02	800,991	1,116,911	high
39	PC-5p-27517_164	TACATGCAGAGGTGGAGCAAGA	up	2.31	3.34E-02	6	30	middle
40	dre-miR-1	TGGAATGTAAAGAAGTATGTAT	up	4.38	3.36E-02	75	1,564	middle
41	ssa-miR-1-3p	TGGAATGTAAAGAAGTATGTAT	up	4.38	3.36E-02	75	1,564	middle
42	tni-miR-10c	TACCCTGTAGATCCGGATTTGT	up	1.59	3.37E-02	3,194	5,975	middle
43	mmu-let-7j_1ss8TG	TGAGGTAGTAGTTTGTGCTGTTAT	up	2.61	3.62E-02	2	9	middle
44	aca-miR-338-3p_R+2	TCCAGCATCAGTGATTTTGTTAA	up	2.34	3.63E-02	0	2	low
45	tni-miR-194_R+1	TGTAACAGCAACTCCATGTGGA	up	0.35	3.65E-02	28,518	36,302	high
46	PC-5p-8690_526	GATGTTGAGTATCAAACTGTAT	down	-0.99	3.74E-02	102	51	middle
47	dre-miR-194a_R+2	TGTAACAGCAACTCCATGTGGAT	up	0.74	3.81E-02	1,172	1,956	middle
48	ola-miR-194-3p_1ss20CT	CCAGTGGAGGTGCTGTTACTTG	up	0.19	3.95E-02	330	377	middle

49	ssa-miR-26d-5p_L+1_1ss13TC	CTTCAAGTAATCCAGGATAGGCT	up	0.73	4.14E-02	94	156	middle
50	rno-miR-122-5p_L+3	ATCTGGAGTGTGACAATGGTGTTTG	up	1.37	4.33E-02	42	108	middle
51	ccr-miR-99_R+3	AACCCGTAGATCCGATCTTGTGAA	up	2.28	4.43E-02	7	35	middle
52	gga-miR-99a-5p_R+2	AACCCGTAGATCCGATCTTGTGAA	up	2.28	4.43E-02	7	35	middle
53	ssa-mir-15c-2-p3_1ss11CA	TGCGAACCATAATTTGCTGCTT	down	-0.55	4.44E-02	457	312	middle
54	PC-3p-50929_43	TGGAAGTGTCAGAAATTCTGAGT	up	1.80	4.45E-02	7	23	middle
55	ssa-miR-206-3p	TGGAATGTAAGGAAGTGTGTGG	up	4.89	4.59E-02	23	693	middle
56	PC-3p-11630_419	ATGAGGAAAAGAAGTTAGGAGA	down	-0.89	4.59E-02	162	88	middle
57	ola-miR-462_L-1R+4	TAACGGAACCCATAATGCAGCT	down	-0.70	4.68E-02	16,178	9,974	high
58	ssc-miR-206	TGGAATGTAAGGAAGTGTGTGA	up	inf	4.70E-02	0	6	low
59	dre-miR-21_1ss23CA	TAGCTTATCAGACTGGTGTTGGA	up	1.25	4.92E-02	2,204	5,254	high

Note: red is *P* value<=0.001, orange is *P* value <=0.01, blue is *P* value<0.05,

Figure S3 The repeatability analysis of mRNA libraries between samples by using of pearson correlation

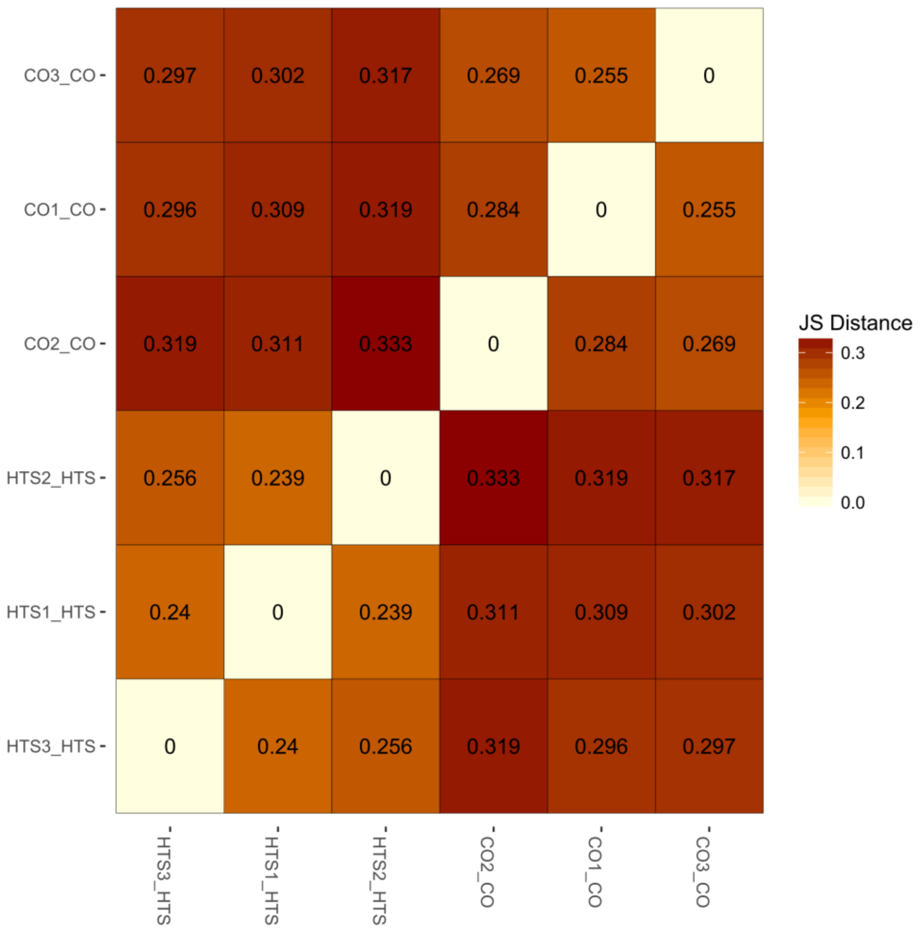

Table S5 Overview of reads for mRNA-seq of GIFT (*Oreochromis_niloticus*) and quality filtering

Sample	Raw Data Read	Base	Valid Data Read	Base	Valid% (reads)	Q20%	Q30%	GC content%
CO1_CO	42731386	6.41G	40826712	6.12G	95.54	99.48	95.36	48
CO2_CO	51604690	7.74G	48116524	7.22G	93.24	99.37	95.39	47.50
CO3_CO	43844692	6.58G	42428160	6.36G	96.77	99.42	94.34	48
HTS1_HTS	41627634	6.24G	40083434	6.01G	96.29	99.52	94.81	47
HTS2_HTS	44769570	6.72G	43120542	6.47G	96.32	99.59	95.64	47
HTS3_HTS	53006600	7.95G	50461524	7.57G	95.20	99.55	95.31	48

Table S6 Summary of read data aligned with *Oreochromis_niloticus* transcriptome

Sample	CO1_CO	CO2_CO	CO3_CO	HTS1_HTS	HTS2_HTS	HTS3_HTS
Valid reads	40826712	48116524	42428160	40083434	43120542	50461524
Mapped reads	25941213(63.54%)	29535094(61.38%)	26308819(62.01%)	25838466(64.46%)	26844109(62.25%)	33704213(66.79%)
Unique Mapped reads	25187538(61.69%)	28797265(59.85%)	25642608(60.44%)	25214535(62.91%)	26233302(60.84%)	32813187(65.03%)
Multi Mapped reads	753675(1.85%)	737829(1.53%)	666211(1.57%)	623931(1.56%)	610807(1.42%)	891026(1.77%)
PE Mapped reads	11347963(27.80%)	12920281(26.85%)	11265849(26.55%)	11043868(27.55%)	11781247(27.32%)	14641475(29.02%)
Mapped left reads	13697991(33.55%)	15557914(32.33%)	14117705(33.27%)	13752022(34.31%)	14108499(32.72%)	17866206(35.41%)
Mapped right reads	12243222(29.99%)	13977180(29.05%)	12191114(28.73%)	12086444(30.15%)	12735610(29.53%)	15838007(31.39%)
Reads map to sense strand	12642361(30.97%)	14463772(30.06%)	12871086(30.34%)	12654888(31.57%)	12700076(29.45%)	16018118(31.74%)
Reads map to antisense strand	12545177(30.73%)	14333493(29.79%)	12771522(30.10%)	12559647(31.33%)	12658774(29.36%)	15939967(31.59%)
Non-splice reads	11024654(27.00%)	14357418(29.84%)	11530634(27.18%)	12079323(30.14%)	13229822(30.68%)	14597325(28.93%)
Splice reads	14162884(34.69%)	14439847(30.01%)	14111974(33.26%)	13135212(32.77%)	12129028(28.13%)	17360760(34.40%)

Table S7 Exonic rates (%) of CO and HTS libraries regarding the mRNA-seq experiments

Samples	CO1_CO	CO2_CO	CO3_CO	HTS1_HTS	HTS2_HTS	HTS3_HTS
exon	89.29	87.78	89.22	88.30	87.84	90.11
intron	3.81	3.75	3.54	3.89	4.29	3.53
intergenic	6.90	8.47	7.24	7.80	7.88	6.36

Table S8 List of the differentially expressed genes in response to high temperature stress

Gene_id	Annotation	HTS_fpkm	CO_fpkm	log2(fold_change)	P_value
XLOC_000039	ENSONIG00000020575	65.64	22.38	1.55	0.00
XLOC_000073	-	0	2.26	-inf	0.00
XLOC_000074	-	101.58	725.20	-2.84	0.00
XLOC_000083	-	1.21	62.23	-5.68	0.00
XLOC_000114	-	20.12	0	inf	0.00
XLOC_000118	ENSONIG00000007929	50.77	7.38	2.78	0.00
XLOC_000135	-	1.41	0	inf	0.00
XLOC_000138	ENSONIG00000000439	6610.60	1227.16	2.43	0.00
XLOC_000225	-	5.56	29.72	-2.42	0.00
XLOC_000227	-	3.17	57.04	-4.17	0.00
XLOC_000228	-	2.94	27.09	-3.20	0.00
XLOC_000248	-	12592.30	1317.65	3.26	0.00
XLOC_000249	-	241.44	2279.30	-3.24	0.00
XLOC_000275	-	1.59	0	inf	0.00
XLOC_000276	-	2.07	15.39	-2.90	0.00
XLOC_000305	-	173.65	17.99	3.27	0.00
XLOC_000309	-	11.76	91.62	-2.96	0.00
XLOC_000315	-	1.57	0	inf	0.00
XLOC_000316	-	1.38	11.62	-3.08	0.00
XLOC_000318	-	89.11	5.73	3.96	0.00
XLOC_000332	-	7.03	0	inf	0.00
XLOC_000361	ENSONIG00000020545	1629.58	1.43	10.16	0.00
XLOC_000362	-	1500.68	7801.49	-2.38	0.00
XLOC_000368	-	0	3.09	-inf	0.00
XLOC_000378	-	2.98	0	inf	0.00
XLOC_000421	-	208.93	75.10	1.48	0.00
XLOC_000488	tmed3	307.18	59.70	2.36	0.00
XLOC_000489	IDH2 (1 of many)	71.96	17.63	2.03	0.00
XLOC_000499	-	0	4.17	-inf	0.00
XLOC_000525	-	0	1.29	-inf	0.00
XLOC_000530	sdr42e1	9.91	2.90	1.77	0.00
XLOC_000556	abcc12	9.01	0.96	3.23	0.00
XLOC_000601	nadsyn1	16.63	6.67	1.32	0.00
XLOC_000612	hmgcra	33.04	364.88	-3.46	0.00
XLOC_000615	gnpnat1	9.48	2.24	2.08	0.00
XLOC_000628	-	418.94	105.03	2.00	0.00
XLOC_000633	-	0	5.19	-inf	0.00
XLOC_000634	fam214a	3.91	25.86	-2.73	0.00
XLOC_000636	onecut1	6.39	1.59	2.01	0.00
XLOC_000651	mmp2	1.93	6.98	-1.86	0.00
XLOC_000661	adcy7	0.95	3.46	-1.86	0.00
XLOC_000663	heatr3	12.76	2.12	2.59	0.00
XLOC_000666	ENSONIG00000020603	1.68	14.47	-3.11	0.00
XLOC_000681	cyp1a	8.23	123.66	-3.91	0.00

XLOC_000700	ABCC11	149.34	7.32	4.35	0.00
XLOC_000719	dhcr7	4.31	29.56	-2.78	0.00
XLOC_000725	emilin1b	9.92	25.23	-1.35	0.00
XLOC_000734	-	311.94	0	inf	0.00
XLOC_000737	ENSONIG00000003297	0.78	3.25	-2.06	0.00
XLOC_000747	cog8	7.53	2.70	1.48	0.00
XLOC_000752	znf395a	34.43	4.33	2.99	0.00
XLOC_000791	mrc1b (1 of many)	14.67	2.62	2.49	0.00
XLOC_000793	mrc1b (1 of many)	4.86	0.33	3.89	0.00
XLOC_000794	mrc1b (1 of many)	0.30	2.15	-2.85	0.00
XLOC_000823	npc1	14.74	5.27	1.48	0.00
XLOC_000834	-	23.21	346.91	-3.90	0.00
XLOC_000836	-	2.09	0	inf	0.00
XLOC_000891	ENSONIG00000009803	128.95	16.84	2.94	0.00
XLOC_000897	-	0	1.65	-inf	0.00
XLOC_000903	-	10.57	84.78	-3.00	0.00
XLOC_000909	-	0	3.97	-inf	0.00
XLOC_000921	cacybp	57.57	7.32	2.98	0.00
XLOC_000974	SLC22A4	2.43	14.06	-2.54	0.00
XLOC_001000	slc25a12	9.80	44.68	-2.19	0.00
XLOC_001016	DPP9 (1 of many)	5.30	1.44	1.88	0.00
XLOC_001050	bcl6ab	7.09	1.92	1.89	0.00
XLOC_001055	ripk2	0.56	2.45	-2.13	0.00
XLOC_001071	-	61.34	21.64	1.50	0.00
XLOC_001072	ENSONIG00000010733	255.17	60.30	2.08	0.00
XLOC_001112	pter	2.61	9.74	-1.90	0.00
XLOC_001120	greb1l	1.20	5.44	-2.18	0.00
XLOC_001122	aqp1a.1,aqp1a.2	23.60	123.20	-2.38	0.00
XLOC_001134	wnt9a	1.23	4.06	-1.72	0.00
XLOC_001179	-	8.99	1.90	2.24	0.00
XLOC_001181	eif4g1a	84.47	25.68	1.72	0.00
XLOC_001190	agxtb,thap4	398.62	101.60	2.01	0.00
XLOC_001195	tal1	1.91	7.35	-1.95	0.00
XLOC_001231	eef2a.2	4.40	14.02	-1.67	0.00
XLOC_001257	spsb4b	4.84	0.90	2.42	0.00
XLOC_001258	slc25a36b (1 of many)	6.47	25.73	-1.99	0.00
XLOC_001265	ifi30	26.61	112.28	-2.08	0.00
XLOC_001360	pgbd5	0.36	4.25	-3.55	0.00
XLOC_001409	cd74b	170.77	1102.76	-2.69	0.00
XLOC_001447	wipf1b	1.54	5.13	-1.74	0.00
XLOC_001452	-	14.44	5.97	1.27	0.00
XLOC_001520	actn2b,p4ha1b	4.95	0.65	2.94	0.00
XLOC_001540	tnfaip3	2.72	15.78	-2.53	0.00
XLOC_001565	ENSONIG00000001070	2.25	27.52	-3.62	0.00
XLOC_001572	slc16a12a	2.12	8.94	-2.08	0.00
XLOC_001594	ugp2b	21.05	8.05	1.39	0.00
XLOC_001623	epas1b	8.90	3.27	1.45	0.00
XLOC_001631	si:ch1073-126c3.2	105.39	405.41	-1.94	0.00

XLOC_001645	KDELR2 (1 of many)	103.37	27.34	1.92	0.00
XLOC_001656	abcc3	1.64	4.41	-1.42	0.00
XLOC_001707	get4	13.64	3.39	2.01	0.00
XLOC_001716	rsl1d1	62.24	19.33	1.69	0.00
XLOC_001718	-	17.72	5.21	1.77	0.00
XLOC_001725	zgc:163057	3.96	25.37	-2.68	0.00
XLOC_001729	pgp	15.24	2.36	2.69	0.00
XLOC_001761	kat7b	7.69	2.21	1.80	0.00
XLOC_001771	slc6a16b	3.91	0.59	2.73	0.00
XLOC_001789	decr2	12.91	45.68	-1.82	0.00
XLOC_001829	vkorc1	99.72	6.40	3.96	0.00
XLOC_001835	foxk2	4.63	0.50	3.20	0.00
XLOC_001850	TOB1 (1 of many)	64.04	17.80	1.85	0.00
XLOC_001862	-	0.98	0	inf	0.00
XLOC_001886	sh3bp1	2.85	7.56	-1.40	0.00
XLOC_001890	aco2	11.36	3.57	1.67	0.00
XLOC_001916	si:dkey-121b10.7	13.60	1.33	3.36	0.00
XLOC_001920	-	0	2.95	-inf	0.00
XLOC_001931	bhlha15	21.07	1.75	3.59	0.00
XLOC_002011	ENSONIG00000020063	10.30	2.96	1.80	0.00
XLOC_002037	ENSONIG00000016564	8.93	22.91	-1.36	0.00
XLOC_002048	agmat	3.51	22.53	-2.68	0.00
XLOC_002052	-	11.36	0	inf	0.00
XLOC_002055	RSBN1	6.87	0.76	3.17	0.00
XLOC_002056	-	0	1.73	-inf	0.00
XLOC_002061	rapgef3	1.76	5.56	-1.66	0.00
XLOC_002062	dazap2	13.32	33.25	-1.32	0.00
XLOC_002063	si:ch211-210c8.6	1.62	8.16	-2.33	0.00
XLOC_002119	ENSONIG00000016898	0	1.08	-inf	0.00
XLOC_002159	-	118.89	19.87	2.58	0.00
XLOC_002160	-	71.37	7.74	3.21	0.00
XLOC_002184	FBLN2 (1 of many)	4.44	11.42	-1.36	0.00
XLOC_002201	ENSONIG00000017244	6.65	24.60	-1.89	0.00
XLOC_002223	slc6a6b	2.50	7.09	-1.51	0.00
XLOC_002231	slc17a9b	9.66	3.10	1.64	0.00
XLOC_002244	-	11.17	71.09	-2.67	0.00
XLOC_002264	ENSONIG00000021134	238.07	59.67	2.00	0.00
XLOC_002291	ptpn22	1.20	4.52	-1.91	0.00
XLOC_002315	ENSONIG00000016742	0.77	6.40	-3.05	0.00
XLOC_002318	slc13a3	6.41	25.67	-2.00	0.00
XLOC_002336	ppdpfa	4067.57	892.91	2.19	0.00
XLOC_002357	wasa	1.32	7.81	-2.56	0.00
XLOC_002364	-	11.00	0	inf	0.00
XLOC_002369	-	6.40	0	inf	0.00
XLOC_002407	rbm38	1.75	9.91	-2.50	0.00
XLOC_002416	irf10	1.52	12.88	-3.09	0.00
XLOC_002434	wdr46	3.86	1.27	1.60	0.00
XLOC_002443	SMIM4	72.47	11.93	2.60	0.00

XLOC_002467	uckl1a	2.67	8.26	-1.63	0.00
XLOC_002475	c23h20orf24	44.61	10.06	2.15	0.00
XLOC_002476	plcg1	2.48	5.96	-1.27	0.00
XLOC_002478	ncoa6	3.92	8.98	-1.20	0.00
XLOC_002479	GGT7	0.45	3.93	-3.13	0.00
XLOC_002480	TP53INP2	13.23	41.76	-1.66	0.00
XLOC_002481	samhd1	5.71	24.89	-2.12	0.00
XLOC_002504	zgc:153639	1.46	7.74	-2.40	0.00
XLOC_002506	-	8.88	34.21	-1.95	0.00
XLOC_002562	-	141.31	414.20	-1.55	0.00
XLOC_002589	ENSONIG00000006360	3.89	42.66	-3.46	0.00
XLOC_002595	ca2	2.47	14.95	-2.60	0.00
XLOC_002631	kpna1	9.38	3.79	1.31	0.00
XLOC_002642	ENSONIG00000005850	18.36	99.09	-2.43	0.00
XLOC_002645	cpb1	107.46	609.01	-2.50	0.00
XLOC_002702	CDH7 (1 of many)	3.19	1.03	1.64	0.00
XLOC_002722	tnk2a (1 of many)	2.35	0.57	2.06	0.00
XLOC_002730	-	45.21	259.34	-2.52	0.00
XLOC_002751	-	0	17.14	-inf	0.00
XLOC_002781	cotl1	12.52	51.24	-2.03	0.00
XLOC_002783	plcg2	1.17	3.30	-1.50	0.00
XLOC_002786	zfpm1	2.56	0.88	1.55	0.00
XLOC_002864	ca5a	378.77	6.22	5.93	0.00
XLOC_002882	si:ch211-122c9.5	0.12	3.87	-5.04	0.00
XLOC_002894	syk	2.50	9.11	-1.87	0.00
XLOC_002905	prrc1	18.45	6.43	1.52	0.00
XLOC_002927	zmiz2 (1 of many)	0.39	2.57	-2.72	0.00
XLOC_002968	herpud1,slc12a3	269.71	14.50	4.22	0.00
XLOC_003011	0000010996,ENSONIG0	3.59	10.48	-1.55	0.00
XLOC_003026	map1lc3b	15.46	46.49	-1.59	0.00
XLOC_003058	slc27a6	123.51	37.92	1.70	0.00
XLOC_003089	derl2	32.27	9.32	1.79	0.00
XLOC_003093	hmgcs1	6.42	58.35	-3.18	0.00
XLOC_003104	ENSONIG00000001709	6.72	0	inf	0.00
XLOC_003123	ncl1	11.73	3.81	1.62	0.00
XLOC_003128	fzr1a,haus5	11.48	31.28	-1.45	0.00
XLOC_003143	tor3a	110.19	17.19	2.68	0.00
XLOC_003175	lmx1a,rxrgb	20.19	7.37	1.45	0.00
XLOC_003194	lpl	266.59	45.15	2.56	0.00
XLOC_003195	ENSONIG00000002328	16.46	0.45	5.20	0.00
XLOC_003213	SLC25A42 (1 of many)	48.81	3.60	3.76	0.00
XLOC_003230	uap1	15.41	3.32	2.22	0.00
XLOC_003240	cyp2n13	301.27	53.01	2.51	0.00
XLOC_003257	ENSONIG00000002751	7.40	41.34	-2.48	0.00
XLOC_003267	suco	11.10	0.98	3.50	0.00
XLOC_003271	dab1a	12.63	68.12	-2.43	0.00
XLOC_003274	dhcr24	6.29	23.88	-1.93	0.00
XLOC_003276	aqp12	267.76	38.58	2.79	0.00

XLOC_003305	CELA3A	1.54	11.33	-2.87	0.00
XLOC_003312	CDKL5 (1 of many)	0.44	2.31	-2.40	0.00
XLOC_003332	-	17.51	49.86	-1.51	0.00
XLOC_003333	lrrc15	24.51	8.98	1.45	0.00
XLOC_003362	mibp	58.50	6.74	3.12	0.00
XLOC_003375	si:zfos-1404b8.2	47.42	139.07	-1.55	0.00
XLOC_003405	si:dkey-42p8.3	4.88	0.79	2.63	0.00
XLOC_003416	si:ch1073-396h14.1	78.99	1.69	5.54	0.00
XLOC_003421	-	83.46	333.17	-2.00	0.00
XLOC_003467	CYP2J2 (1 of many)	46.20	170.48	-1.88	0.00
XLOC_003483	CYP2J2 (1 of many)	41.61	310.44	-2.90	0.00
XLOC_003484	hook1	1.78	5.23	-1.55	0.00
XLOC_003494	JUN (1 of many)	32.76	3.56	3.20	0.00
XLOC_003502	tmed5	9.78	2.68	1.87	0.00
XLOC_003551	tmem45a	4.74	1.02	2.21	0.00
XLOC_003556	-	0	1.69	-inf	0.00
XLOC_003559	-	0	1.14	-inf	0.00
XLOC_003594	dnajc25	36.45	8.29	2.14	0.00
XLOC_003636	ENSONIG00000012766	22.14	3.35	2.72	0.00
XLOC_003683	GMFG	5.78	28.47	-2.30	0.00
XLOC_003684	tsr1	9.31	2.33	2.00	0.00
XLOC_003707	ENSONIG00000013185	86.65	16.85	2.36	0.00
XLOC_003708	-	539.05	0	inf	0.00
XLOC_003725	ENSONIG00000012561	0	1.61	-inf	0.00
XLOC_003732	-	98.65	0	inf	0.00
XLOC_003733	-	28.19	139.78	-2.31	0.00
XLOC_003735	-	44.40	16.96	1.39	0.00
XLOC_003750	-	0	1.96	-inf	0.00
XLOC_003795	slc30a5	38.87	10.85	1.84	0.00
XLOC_003808	si:dkey-11o18.5	0.67	3.09	-2.22	0.00
XLOC_003814	cel.2	43.69	271.38	-2.64	0.00
XLOC_003817	gfi1b	2.18	7.41	-1.77	0.00
XLOC_003820	gsna	0.50	3.39	-2.78	0.00
XLOC_003822	si:ch211-127i16.2	0.98	9.62	-3.29	0.00
XLOC_003824	ENSONIG00000013072	11.44	3.16	1.86	0.00
XLOC_003833	stoml2	16.78	6.57	1.35	0.00
XLOC_003836	-	24.62	75.31	-1.61	0.00
XLOC_003873	ENSONIG00000013184	33.74	4.28	2.98	0.00
XLOC_003879	ENSONIG00000022213	0	10.52	-inf	0.00
XLOC_003902	ldha	16.57	5.14	1.69	0.00
XLOC_003904	rassf7a	13.03	3.94	1.73	0.00
XLOC_003916	unc45a	9.10	2.87	1.67	0.00
XLOC_003921	ENSONIG00000014640	11.52	36.96	-1.68	0.00
XLOC_003960	ENSONIG00000014877	84.60	1065.75	-3.66	0.00
XLOC_003981	calub	173.87	46.74	1.90	0.00
XLOC_003987	miox	129.40	927.77	-2.84	0.00
XLOC_003997	-	3.26	0	inf	0.00
XLOC_004013	ctsh (1 of many)	6.33	28.59	-2.18	0.00

XLOC_004015	alpk3a	2.68	8.53	-1.67	0.00
XLOC_004080	spty2d1	7.16	1.39	2.37	0.00
XLOC_004113	ENSONIG00000014729	58.41	226.59	-1.96	0.00
XLOC_004136	mafb	11.74	2.09	2.49	0.00
XLOC_004139	chkb	32.56	260.73	-3.00	0.00
XLOC_004146	zgc:110286	156.85	31.92	2.30	0.00
XLOC_004165	fkbp4	36.95	4.87	2.92	0.00
XLOC_004192	zgc:171719	5.49	32.40	-2.56	0.00
XLOC_004203	DNAJA4	136.49	1.84	6.21	0.00
XLOC_004206	-	90.75	14.85	2.61	0.00
XLOC_004207	-	51.90	7.91	2.71	0.00
XLOC_004222	ppcdc	15.65	4.65	1.75	0.00
XLOC_004243	myrf	4.06	0.88	2.21	0.00
XLOC_004244	fads2 (1 of many)	22.81	244.41	-3.42	0.00
XLOC_004245	fads2 (1 of many)	13.59	507.21	-5.22	0.00
XLOC_004248	si:dkey-201c1.2	21.35	7.98	1.42	0.00
XLOC_004317	sel1l	77.66	7.04	3.46	0.00
XLOC_004335	ENSONIG00000000865	4.83	0.96	2.33	0.00
XLOC_004363	-	638.51	71.69	3.15	0.00
XLOC_004367	ccnk	6.41	2.03	1.66	0.00
XLOC_004379	-	0	2.23	-inf	0.00
XLOC_004396	si:ch73-91k6.2	43.81	14.23	1.62	0.00
XLOC_004408	aldh8a1	28.08	114.64	-2.03	0.00
XLOC_004423	-	67.40	17.24	1.97	0.00
XLOC_004424	-	30.95	7.64	2.02	0.00
XLOC_004432	pgm3	9.64	3.19	1.59	0.00
XLOC_004447	rhov	1.08	4.56	-2.08	0.00
XLOC_004448	chac1	167.83	45.35	1.89	0.00
XLOC_004453	)NIG00000000805,hsp9(	449.57	2.89	7.28	0.00
XLOC_004481	enpp5	1.55	6.92	-2.16	0.00
XLOC_004497	sptlc2b	12.26	4.02	1.61	0.00
XLOC_004507	-	4.24	17.21	-2.02	0.00
XLOC_004554	dzip1	1.18	4.27	-1.86	0.00
XLOC_004605	adamts1	4.65	0.52	3.16	0.00
XLOC_004616	fam134a	3.17	8.42	-1.41	0.00
XLOC_004639	-	0	1.42	-inf	0.00
XLOC_004671	zgc:92335	21.90	7.39	1.57	0.00
XLOC_004712	spp2	113.47	994.82	-3.13	0.00
XLOC_004723	pprc1	2.63	0.52	2.34	0.00
XLOC_004730	rrp12	4.46	1.33	1.75	0.00
XLOC_004733	wdfy4	0.69	3.43	-2.31	0.00
XLOC_004745	fuom	0.69	4.66	-2.76	0.00
XLOC_004782	itga9	2.45	11.65	-2.25	0.00
XLOC_004835	-	7.73	1.08	2.83	0.00
XLOC_004851	bag3	29.95	0.97	4.94	0.00
XLOC_004874	polr1b	2.56	0.62	2.04	0.00
XLOC_004902	ercc6	3.25	1.22	1.42	0.00
XLOC_004913	angpt2a	7.82	1.99	1.97	0.00

XLOC_004914	0000009676,ENSONIG0	10.65	88.73	-3.06	0.00
XLOC_004934	erlec1	35.08	10.85	1.69	0.00
XLOC_004951	NOLC1	4.45	1.48	1.59	0.00
XLOC_004995	-	0	0.95	-inf	0.00
XLOC_005015	pik3ap1	1.20	3.35	-1.48	0.00
XLOC_005024	ENSONIG00000010389	4.56	15.40	-1.75	0.00
XLOC_005031	cishb	1.01	6.61	-2.71	0.00
XLOC_005060	srgap3	0.67	2.21	-1.72	0.00
XLOC_005064	gpx1b	83.16	263.65	-1.66	0.00
XLOC_005065	RBM5 (1 of many)	38.55	8.49	2.18	0.00
XLOC_005074	SEC61A1 (1 of many)	54.41	17.41	1.64	0.00
XLOC_005125	suox	206.07	31.59	2.71	0.00
XLOC_005140	cnpy2	13.21	1.19	3.47	0.00
XLOC_005164	glt8d1	11.56	2.31	2.32	0.00
XLOC_005174	acap3b	4.50	0.76	2.57	0.00
XLOC_005187	zgc:123305	3.79	0.75	2.33	0.00
XLOC_005196	loxl2a	4.84	1.14	2.08	0.00
XLOC_005258	-	2.09	0	inf	0.00
XLOC_005304	cdk4	26.91	8.09	1.73	0.00
XLOC_005321	ENSONIG00000019270	190.90	72.23	1.40	0.00
XLOC_005342	gnl3	11.36	2.71	2.07	0.00
XLOC_005344	ENSONIG00000019342	29.73	7.96	1.90	0.00
XLOC_005395	tpgs2	44.31	16.26	1.45	0.00
XLOC_005431	hspa13	13.72	4.01	1.78	0.00
XLOC_005470	vmp1	24.67	9.76	1.34	0.00
XLOC_005472	rps6kb1a (1 of many)	10.53	2.85	1.89	0.00
XLOC_005477	-	21.58	7.83	1.46	0.00
XLOC_005493	zbtb38	3.51	0.79	2.14	0.00
XLOC_005515	ENSONIG00000005018	3.38	13.22	-1.97	0.00
XLOC_005543	ENSONIG00000005185	14.44	5.68	1.35	0.00
XLOC_005548	-	37.73	0	inf	0.00
XLOC_005551	gdpd5b	35.67	7.35	2.28	0.00
XLOC_005555	siae	25.80	7.40	1.80	0.00
XLOC_005565	ENSONIG00000005394	0.80	6.35	-2.98	0.00
XLOC_005587	BCHE	27.40	76.04	-1.47	0.00
XLOC_005593	-	21.35	2.89	2.89	0.00
XLOC_005609	eef2k	60.24	10.82	2.48	0.00
XLOC_005628	chac2	7.56	0.99	2.93	0.00
XLOC_005665	KCNMA1 (1 of many)	3.40	9.00	-1.40	0.00
XLOC_005667	COMTD1 (1 of many)	34.78	163.61	-2.23	0.00
XLOC_005688	-	293.86	30.08	3.29	0.00
XLOC_005691	DNAJC7 (1 of many)	53.37	7.89	2.76	0.00
XLOC_005697	cpn1	9.67	38.18	-1.98	0.00
XLOC_005701	nhlrc2	4.07	1.49	1.45	0.00
XLOC_005712	nmt1b	0.83	5.43	-2.71	0.00
XLOC_005716	-	33.32	8.19	2.02	0.00
XLOC_005727	ENSONIG00000020661	0.47	4.10	-3.12	0.00
XLOC_005729	sec24c	19.01	5.77	1.72	0.00

XLOC_005742	-	185.85	0	inf	0.00
XLOC_005763	RGR (1 of many)	3.01	10.62	-1.82	0.00
XLOC_005765	GHITM	170.40	32.14	2.41	0.00
XLOC_005768	-	0	39.87	-inf	0.00
XLOC_005770	DIT4 (1 of many),anapc1	445.45	37.28	3.58	0.00
XLOC_005829	slc35b1	203.79	24.26	3.07	0.00
XLOC_005833	slc4a1a	10.50	90.78	-3.11	0.00
XLOC_005845	st8sia6 (1 of many)	5.51	1.54	1.84	0.00
XLOC_005861	fam134c	6.49	20.58	-1.66	0.00
XLOC_005878	micall2a	2.22	6.98	-1.65	0.00
XLOC_005887	gpr146	3.53	0.90	1.97	0.00
XLOC_005897	hbae3 (1 of many)	181.55	3333.77	-4.20	0.00
XLOC_005916	nr5a5	2.41	7.46	-1.63	0.00
XLOC_005940	gcgra	12.30	4.22	1.54	0.00
XLOC_005952	-	1.74	8.20	-2.24	0.00
XLOC_005966	retsat	11.38	55.97	-2.30	0.00
XLOC_005968	plcd3a	1.00	8.44	-3.07	0.00
XLOC_005994	ENSONIG00000001066	2.55	48.96	-4.27	0.00
XLOC_006020	ENSONIG00000001286	38.70	16.67	1.22	0.00
XLOC_006051	ENSONIG00000001457	1375.37	21093.30	-3.94	0.00
XLOC_006055	-	7.31	30.91	-2.08	0.00
XLOC_006060	prkcsh	101.51	15.73	2.69	0.00
XLOC_006064	CDC37	34.39	9.88	1.80	0.00
XLOC_006078	ppan	10.10	3.19	1.66	0.00
XLOC_006089	atp13a1	7.94	3.25	1.29	0.00
XLOC_006091	gmip	0.92	3.15	-1.77	0.00
XLOC_006096	si:ch211-241f5.3	1.32	6.81	-2.37	0.00
XLOC_006111	si:ch1073-100f3.2	3.41	19.16	-2.49	0.00
XLOC_006120	-	11.93	4.09	1.55	0.00
XLOC_006128	-	2.92	0	inf	0.00
XLOC_006129	rnaset2	4.92	16.67	-1.76	0.00
XLOC_006134	prrg4	7.51	1.91	1.98	0.00
XLOC_006154	irf8	2.90	11.71	-2.02	0.00
XLOC_006194	cyb5b	12.55	4.32	1.54	0.00
XLOC_006199	mical2a	1.40	9.41	-2.75	0.00
XLOC_006207	tmem41b	8.03	1.72	2.22	0.00
XLOC_006211	scube2	4.30	21.24	-2.31	0.00
XLOC_006221	nucb2a	48.74	13.18	1.89	0.00
XLOC_006233	eif3jb	56.31	10.95	2.36	0.00
XLOC_006250	cyba,mvda	10.39	40.76	-1.97	0.00
XLOC_006319	copb1	35.59	11.99	1.57	0.00
XLOC_006357	-	0	8.77	-inf	0.00
XLOC_006380	jmjd6	18.65	2.43	2.94	0.00
XLOC_006393	-	1467.78	2.03	9.50	0.00
XLOC_006395	-	3.40	0	inf	0.00
XLOC_006396	-	611.21	33.50	4.19	0.00
XLOC_006397	-	26.41	0	inf	0.00
XLOC_006400	-	24.13	0	inf	0.00

XLOC_006401	-	14.36	0	inf	0.00
XLOC_006402	-	28.29	0	inf	0.00
XLOC_006404	-	221.73	0.33	9.39	0.00
XLOC_006405	-	314.22	0.41	9.58	0.00
XLOC_006406	-	9.10	0	inf	0.00
XLOC_006407	-	1.46	0	inf	0.00
XLOC_006408	-	2.18	0	inf	0.00
XLOC_006416	JMJD8	19.63	1.69	3.54	0.00
XLOC_006461	grna	6.05	16.71	-1.47	0.00
XLOC_006488	abi3a	2.61	11.75	-2.17	0.00
XLOC_006491	-	2.26	0	inf	0.00
XLOC_006492	-	1.34	0	inf	0.00
XLOC_006508	utp18	7.32	2.40	1.61	0.00
XLOC_006517	-	34.18	3.86	3.15	0.00
XLOC_006519	MBTD1	5.06	1.03	2.30	0.00
XLOC_006551	ENSONIG00000021447	1151.88	1.24	9.85	0.00
XLOC_006573	pigq	5.55	2.25	1.30	0.00
XLOC_006580	suz12a	2.33	9.50	-2.02	0.00
XLOC_006605	cyth4a	0.27	2.00	-2.88	0.00
XLOC_006610	-	0	3.43	-inf	0.00
XLOC_006642	coro1a	8.54	49.38	-2.53	0.00
XLOC_006665	CHST13 (1 of many)	3.10	0.75	2.06	0.00
XLOC_006694	bhlhe40	27.59	4.10	2.75	0.00
XLOC_006695	NAAA	16.67	44.53	-1.42	0.00
XLOC_006702	gata1a	6.14	28.48	-2.21	0.00
XLOC_006714	RBM38 (1 of many)	5.02	18.41	-1.88	0.00
XLOC_006751	glrx	16.74	59.97	-1.84	0.00
XLOC_006801	top1l	13.84	3.93	1.82	0.00
XLOC_006839	MUSTN1	6.20	1.11	2.48	0.00
XLOC_006842	prkcda	7.09	2.38	1.57	0.00
XLOC_006867	zgc:64106	188.29	772.44	-2.04	0.00
XLOC_006868	ENSONIG00000000375	1.64	16.07	-3.29	0.00
XLOC_006877	zgc:110783	37.40	102.32	-1.45	0.00
XLOC_006880	hhipl2	7.17	1.27	2.50	0.00
XLOC_006921	fndc3ba	5.65	2.18	1.37	0.00
XLOC_006939	-	15.21	4.44	1.77	0.00
XLOC_006943	slc25a21	30.64	9.70	1.66	0.00
XLOC_006959	scarb2 (1 of many)	0.48	15.03	-4.96	0.00
XLOC_006969	-	0	22.24	-inf	0.00
XLOC_007042	map4k5	8.80	24.25	-1.46	0.00
XLOC_007047	-	0.29	12.67	-5.46	0.00
XLOC_007076	nceh1a	6.93	75.84	-3.45	0.00
XLOC_007080	sptb	1.44	4.89	-1.76	0.00
XLOC_007167	-	0	1.74	-inf	0.00
XLOC_007169	nfil3-6	8.20	28.77	-1.81	0.00
XLOC_007201	hmox1a	0.77	7.74	-3.33	0.00
XLOC_007214	ENSONIG00000008347	7.47	43.34	-2.54	0.00
XLOC_007236	PMM1	32.35	6.47	2.32	0.00

XLOC_007252	mmd2a	73.17	27.94	1.39	0.00
XLOC_007253	card11	2.30	17.60	-2.93	0.00
XLOC_007274	rgl2	4.14	12.17	-1.55	0.00
XLOC_007278	tap1	1.68	10.88	-2.70	0.00
XLOC_007280	vmo1a	24.46	282.46	-3.53	0.00
XLOC_007282	PLCL2	5.09	0.62	3.05	0.00
XLOC_007306	pglyrp2	18.11	85.72	-2.24	0.00
XLOC_007319	gcat,tom1	79.51	25.93	1.62	0.00
XLOC_007326	DDX47	15.01	3.41	2.14	0.00
XLOC_007349	si:dkey-167k11.5	16.94	3.96	2.10	0.00
XLOC_007363	mgst1.1	53.04	250.77	-2.24	0.00
XLOC_007371	smcr8a	1.59	0.42	1.91	0.00
XLOC_007394	zgc:63831	7.75	19.55	-1.34	0.00
XLOC_007397	ENSONIG00000008571	1.50	5.68	-1.92	0.00
XLOC_007481	-	45.31	8.25	2.46	0.00
XLOC_007503	-	43.41	0	inf	0.00
XLOC_007526	fn1a	0.20	1.22	-2.60	0.00
XLOC_007574	ctso	8.18	27.21	-1.73	0.00
XLOC_007602	SPATA5	44.35	6.14	2.85	0.00
XLOC_007638	si:ch211-255i20.3	0	1.25	-inf	0.00
XLOC_007647	adh5	33.85	108.10	-1.68	0.00
XLOC_007655	tdo2b	48.45	700.96	-3.85	0.00
XLOC_007660	ppid	20.13	4.69	2.10	0.00
XLOC_007666	tmem33	47.33	7.24	2.71	0.00
XLOC_007725	xkr9	13.93	5.18	1.43	0.00
XLOC_007742	cct5	69.86	23.30	1.58	0.00
XLOC_007745	slc51a	42.40	213.67	-2.33	0.00
XLOC_007752	oplah	0.65	4.57	-2.82	0.00
XLOC_007760	msrb2	26.99	230.68	-3.10	0.00
XLOC_007780	eef1e1	14.05	4.82	1.54	0.00
XLOC_007806	ENSONIG00000014168	22.25	8.69	1.36	0.00
XLOC_007824	tram1	87.28	18.07	2.27	0.00
XLOC_007827	trpa1b	3.70	0.44	3.08	0.00
XLOC_007852	pdia4	1902.25	279.11	2.77	0.00
XLOC_007879	ipo4	7.72	1.92	2.01	0.00
XLOC_007884	mal2	4.04	21.37	-2.40	0.00
XLOC_007903	-	19.88	5.36	1.89	0.00
XLOC_007926	-	5.09	0	inf	0.00
XLOC_007988	ENSONIG00000007222	246.94	15.19	4.02	0.00
XLOC_007995	tmem147	10.44	3.78	1.46	0.00
XLOC_007999	hpn	138.73	34.27	2.02	0.00
XLOC_008014	-	105.24	785.25	-2.90	0.00
XLOC_008038	nudc	25.59	5.12	2.32	0.00
XLOC_008090	zgc:100906	9.50	2.40	1.98	0.00
XLOC_008091	ENSONIG00000007553	14.25	2.08	2.78	0.00
XLOC_008123	tmem54b	6.34	16.03	-1.34	0.00
XLOC_008131	ldlrap1a	9.76	28.09	-1.53	0.00
XLOC_008141	-	128.90	29.71	2.12	0.00

XLOC_008142	-	31.05	7.03	2.14	0.00
XLOC_008143	grb10a	7.35	1.16	2.67	0.00
XLOC_008157	GAS2 (1 of many)	0.76	9.20	-3.59	0.00
XLOC_008160	prc1a,spi1a	2.61	10.51	-2.01	0.00
XLOC_008183	csk	2.16	9.03	-2.07	0.00
XLOC_008186	lmo2	2.94	9.97	-1.76	0.00
XLOC_008191	AEBP2	0.79	7.50	-3.24	0.00
XLOC_008200	-	0.84	5.77	-2.77	0.00
XLOC_008213	PNPLA8 (1 of many)	0.77	3.55	-2.21	0.00
XLOC_008216	-	43.23	0	inf	0.00
XLOC_008238	-	2.15	0	inf	0.00
XLOC_008240	AKR1D1 (1 of many)	46.81	115.64	-1.30	0.00
XLOC_008247	cdkn1cb	17.76	0.86	4.36	0.00
XLOC_008256	-	36.83	91.49	-1.31	0.00
XLOC_008289	hsp90b1	3450.57	475.19	2.86	0.00
XLOC_008304	dhtkd1	49.17	8.26	2.57	0.00
XLOC_008332	plxnb2b	0.51	1.63	-1.67	0.00
XLOC_008354	-	0	1.51	-inf	0.00
XLOC_008389	-	0	6.29	-inf	0.00
XLOC_008400	tinagl1	3.79	1.12	1.76	0.00
XLOC_008401	ubqln4	39.15	15.16	1.37	0.00
XLOC_008412	stmnd1	0	0.76	-inf	0.00
XLOC_008419	tmem55a	0.80	10.86	-3.77	0.00
XLOC_008447	-	146.93	62.43	1.23	0.00
XLOC_008465	zgc:63863	4.86	1.59	1.61	0.00
XLOC_008521	mapk15	0.75	3.45	-2.21	0.00
XLOC_008527	-	0	1.78	-inf	0.00
XLOC_008542	hey1	4.06	0.54	2.91	0.00
XLOC_008552	jarid2b	35.89	5.63	2.67	0.00
XLOC_008555	hrsp12,stk3	26.47	77.63	-1.55	0.00
XLOC_008558	psmg2	133.87	31.72	2.08	0.00
XLOC_008599	-	68.72	24.16	1.51	0.00
XLOC_008601	aldh9a1a.1	20.88	397.55	-4.25	0.00
XLOC_008644	-	13.61	4.32	1.65	0.00
XLOC_008646	-	0	4.72	-inf	0.00
XLOC_008663	-	2.79	0	inf	0.00
XLOC_008674	-	0	1.12	-inf	0.00
XLOC_008708	-	0	109.19	-inf	0.00
XLOC_008726	bmp1b	0.21	7.77	-5.21	0.00
XLOC_008767	-	0	34.18	-inf	0.00
XLOC_008770	atp2a2b	42.77	5.08	3.07	0.00
XLOC_008797	antxr2b	4.98	28.13	-2.50	0.00
XLOC_008807	anxa4	9.78	32.12	-1.71	0.00
XLOC_008811	ENSONIG00000003557	23.22	60.36	-1.38	0.00
XLOC_008822	SCARB2	14.69	49.32	-1.75	0.00
XLOC_008832	ulk1b	1.16	4.98	-2.10	0.00
XLOC_008887	rasgef1bb	13.91	6.10	1.19	0.00
XLOC_008890	SLC46A2	1.57	6.92	-2.14	0.00

XLOC_008904	-	2.55	0	inf	0.00
XLOC_008944	ccl19a.1	56.46	375.96	-2.74	0.00
XLOC_008993	gstt1a	335.71	2021.92	-2.80	0.00
XLOC_009084	wfs1b	4.64	1.56	1.58	0.00
XLOC_009119	f8	1.75	4.37	-1.32	0.00
XLOC_009125	ENSONIG00000005270	2.03	8.71	-2.10	0.00
XLOC_009143	trmt112	33.34	5.61	2.57	0.00
XLOC_009175	-	0	1.18	-inf	0.00
XLOC_009187	ENSONIG00000004934	11.13	3.97	1.49	0.00
XLOC_009196	DOCK2	0.57	2.60	-2.19	0.00
XLOC_009222	-	91.14	32.55	1.49	0.00
XLOC_009223	SLC16A2	5.95	1.29	2.21	0.00
XLOC_009229	zgc:171967	109.08	26.70	2.03	0.00
XLOC_009231	scyl1	7.15	2.92	1.29	0.00
XLOC_009255	yif1a	42.72	16.24	1.40	0.00
XLOC_009259	RAD54L2 (1 of many)	1.73	0.09	4.26	0.00
XLOC_009274	wdr6	2.32	0.57	2.03	0.00
XLOC_009307	PRELID3B	24.24	4.50	2.43	0.00
XLOC_009346	exosc10	36.50	14.23	1.36	0.00
XLOC_009353	noc2l	13.56	5.51	1.30	0.00
XLOC_009381	-	1.26	14.06	-3.49	0.00
XLOC_009390	srsf6a	53.77	20.05	1.42	0.00
XLOC_009391	eif2s2	47.25	19.84	1.25	0.00
XLOC_009396	bpifcl	8.11	39.57	-2.29	0.00
XLOC_009398	SHMT2 (1 of many)	3.36	41.54	-3.63	0.00
XLOC_009406	-	0	3.85	-inf	0.00
XLOC_009415	si:ch211-263k4.2	1.30	0.23	2.51	0.00
XLOC_009416	-	0	2.65	-inf	0.00
XLOC_009449	pik3cd	0.42	3.18	-2.91	0.00
XLOC_009466	ENSONIG00000002460	2.24	11.92	-2.41	0.00
XLOC_009492	impdh1b	3.81	10.70	-1.49	0.00
XLOC_009508	chchd3a	8.74	2.61	1.75	0.00
XLOC_009549	si:dkey-159a18.1	8.47	0.21	5.30	0.00
XLOC_009551	-	135.07	5.08	4.73	0.00
XLOC_009553	-	4.13	0	inf	0.00
XLOC_009556	-	4.44	0	inf	0.00
XLOC_009615	-	0	2.76	-inf	0.00
XLOC_009630	-	3.68	0	inf	0.00
XLOC_009637	-	0	1.64	-inf	0.00
XLOC_009681	zap70	0.35	3.48	-3.30	0.00
XLOC_009683	mknk2a	30.61	3.27	3.23	0.00
XLOC_009697	creld2	351.50	39.50	3.15	0.00
XLOC_009720	FGD4 (1 of many)	0.36	6.64	-4.22	0.00
XLOC_009724	ETV6	18.16	6.73	1.43	0.00
XLOC_009752	ptprc	6.65	20.40	-1.62	0.00
XLOC_009762	mcm7	8.58	28.28	-1.72	0.00
XLOC_009776	ENSONIG00000002383	288.45	1201.09	-2.06	0.00
XLOC_009780	gsr	7.97	19.28	-1.27	0.00

XLOC_009786	sfxn5b	0.29	7.14	-4.64	0.00
XLOC_009814	sash3	1.77	6.49	-1.88	0.00
XLOC_009817	tspan7	29.44	7.00	2.07	0.00
XLOC_009915	mcf2a	3.60	15.91	-2.14	0.00
XLOC_009929	HSPA5 (1 of many)	4966.88	259.79	4.26	0.00
XLOC_009991	HNMT (1 of many)	20.92	137.29	-2.71	0.00
XLOC_009993	spopla	22.10	7.30	1.60	0.00
XLOC_010014	-	0.89	7.05	-2.98	0.00
XLOC_010027	zgc:152951	2.97	7.50	-1.34	0.00
XLOC_010040	gmppab	35.24	11.26	1.65	0.00
XLOC_010068	TUBB4A (1 of many)	11.35	4.32	1.39	0.00
XLOC_010124	fbln5	3.09	12.66	-2.03	0.00
XLOC_010131	SMOC1	2.87	18.37	-2.68	0.00
XLOC_010138	ENSONIG00000019487	5.66	41.09	-2.86	0.00
XLOC_010140	ENSONIG00000019489	402.65	1265.67	-1.65	0.00
XLOC_010162	wdr43	12.20	4.64	1.39	0.00
XLOC_010165	-	1.89	0	inf	0.00
XLOC_010173	odc1	16.48	2.71	2.61	0.00
XLOC_010186	rdh12	5.06	17.00	-1.75	0.00
XLOC_010218	NPC1L1	1.17	8.88	-2.92	0.00
XLOC_010241	ulk1a	12.04	2.02	2.58	0.00
XLOC_010248	klhl22	1.13	3.68	-1.70	0.00
XLOC_010309	NSONIG00000010777,ba	10.79	3.44	1.65	0.00
XLOC_010315	aplnra	1.99	12.37	-2.64	0.00
XLOC_010323	slc20a1a	4.59	20.44	-2.15	0.00
XLOC_010354	chfr	13.36	4.67	1.52	0.00
XLOC_010375	PLA2G1B (1 of many)	18.23	308.44	-4.08	0.00
XLOC_010387	spi1b	3.60	10.46	-1.54	0.00
XLOC_010398	dhodh	17.32	4.89	1.83	0.00
XLOC_010496	diaph3	0.09	2.48	-4.74	0.00
XLOC_010501	cyp27c1	4.45	15.46	-1.80	0.00
XLOC_010507	ly75	0.46	1.85	-2.01	0.00
XLOC_010519	tmem39a	6.78	2.11	1.68	0.00
XLOC_010523	G00000003386,si:zfos-1	344.88	58.70	2.55	0.00
XLOC_010557	clint1a	18.97	7.22	1.39	0.00
XLOC_010573	n4bp3	8.61	1.45	2.57	0.00
XLOC_010583	dusp1	58.66	19.92	1.56	0.00
XLOC_010628	ppp2r2ba	12.83	3.36	1.94	0.00
XLOC_010682	rlim	17.69	2.29	2.95	0.00
XLOC_010693	-	0	1.68	-inf	0.00
XLOC_010694	-	0	1.72	-inf	0.00
XLOC_010698	-	0	1.85	-inf	0.00
XLOC_010700	-	0	13.79	-inf	0.00
XLOC_010710	pim2	1.75	18.41	-3.40	0.00
XLOC_010717	-	32.00	0.87	5.21	0.00
XLOC_010727	-	0	1.66	-inf	0.00
XLOC_010733	PTPN7	2.68	8.52	-1.67	0.00
XLOC_010743	-	3.28	0	inf	0.00

XLOC_010744	-	7.15	0	inf	0.00
XLOC_010760	si:dkey-96f10.1	9.21	29.38	-1.67	0.00
XLOC_010783	wu:fb72h05	22.02	3.00	2.88	0.00
XLOC_010787	-	0	5.90	-inf	0.00
XLOC_010807	edem2	6.73	1.52	2.14	0.00
XLOC_010820	ndnl2	14.04	49.17	-1.81	0.00
XLOC_010840	cry1ba	41.86	13.11	1.68	0.00
XLOC_010936	slc1a3a	170.09	46.76	1.86	0.00
XLOC_010940	rmi1	10.08	2.49	2.02	0.00
XLOC_010948	si:ch211-103n10.5	79.21	337.96	-2.09	0.00
XLOC_010968	ENSONIG00000015186	473.73	1889.23	-2.00	0.00
XLOC_010979	PLPP1 (1 of many)	14.99	2.18	2.78	0.00
XLOC_010998	-	0.95	0	inf	0.00
XLOC_011017	gale	64.33	13.86	2.21	0.00
XLOC_011026	mgst3b	27.20	86.24	-1.66	0.00
XLOC_011038	-	64.30	12.69	2.34	0.00
XLOC_011039	ptp4a2a	82.20	23.35	1.82	0.00
XLOC_011068	tmem30aa	32.85	10.64	1.63	0.00
XLOC_011084	lck	0.43	4.43	-3.36	0.00
XLOC_011087	rsrp1	262.97	74.65	1.82	0.00
XLOC_011096	ENSONIG00000005558	1.89	11.38	-2.59	0.00
XLOC_011098	pcmt	3.46	16.50	-2.25	0.00
XLOC_011117	susd6	21.80	9.90	1.14	0.00
XLOC_011171	tmem214	73.59	22.99	1.68	0.00
XLOC_011174	-	3.11	13.25	-2.09	0.00
XLOC_011189	esr2a	7.44	1.82	2.03	0.00
XLOC_011228	nfkbie	6.04	24.06	-1.99	0.00
XLOC_011242	rhag	5.21	28.28	-2.44	0.00
XLOC_011267	copz2	126.66	26.51	2.26	0.00
XLOC_011302	-	931.22	61.64	3.92	0.00
XLOC_011317	cpt1cb	176.56	2.28	6.27	0.00
XLOC_011318	-	14.98	0	inf	0.00
XLOC_011376	col1a1	1.96	4.99	-1.35	0.00
XLOC_011378	-	0	48.10	-inf	0.00
XLOC_011382	-	4.85	79.46	-4.03	0.00
XLOC_011392	psmd11a	11.00	4.41	1.32	0.00
XLOC_011438	KLF15	23.94	8.12	1.56	0.00
XLOC_011443	aldh1l1	7.24	264.72	-5.19	0.00
XLOC_011460	si:dkey-166c18.1	0.91	5.74	-2.66	0.00
XLOC_011464	-	2.58	16.01	-2.63	0.00
XLOC_011467	zgc:112255	40.03	9.72	2.04	0.00
XLOC_011516	TUBA4A (1 of many)	62.42	19.48	1.68	0.00
XLOC_011534	kynu	14.75	47.79	-1.70	0.00
XLOC_011561	-	42.98	9.37	2.20	0.00
XLOC_011562	WBP4	35.19	107.15	-1.61	0.00
XLOC_011569	LECT2 (1 of many)	37.08	102.37	-1.46	0.00
XLOC_011589	-	3.91	21.85	-2.48	0.00
XLOC_011666	-	7.38	1.81	2.03	0.00

XLOC_011742	KLF11 (1 of many)	2.14	10.21	-2.25	0.00
XLOC_011800	cct8	33.08	9.94	1.73	0.00
XLOC_011840	lipia	1.20	8.25	-2.78	0.00
XLOC_011890	chordc1a (1 of many)	19.35	2.00	3.27	0.00
XLOC_011901	slc47a1	3.60	25.24	-2.81	0.00
XLOC_011924	SLC51A (1 of many)	0.58	75.77	-7.04	0.00
XLOC_011925	-	0	12.82	-inf	0.00
XLOC_011962	SIPA1	0.75	2.58	-1.77	0.00
XLOC_011990	hspa4a	29.75	1.11	4.74	0.00
XLOC_012011	slc7a3a	9.64	3.06	1.66	0.00
XLOC_012038	cct6a	56.83	21.46	1.41	0.00
XLOC_012071	larsb	9.67	3.51	1.46	0.00
XLOC_012089	sssca1	12.98	3.58	1.86	0.00
XLOC_012093	ablim3 (1 of many)	0.19	2.85	-3.90	0.00
XLOC_012138	-	1063.18	217.27	2.29	0.00
XLOC_012142	mmp15a	1.12	11.77	-3.40	0.00
XLOC_012196	-	8.75	25.13	-1.52	0.00
XLOC_012201	-	8.63	24.50	-1.50	0.00
XLOC_012214	ccnd2a	19.89	8.37	1.25	0.00
XLOC_012278	ENSONIG00000013586	6.22	17.02	-1.45	0.00
XLOC_012298	hivep1	0.91	2.80	-1.62	0.00
XLOC_012305	apoa4a	9.41	0.28	5.05	0.00
XLOC_012349	-	1.10	0	inf	0.00
XLOC_012379	si:dkeyp-92c9.3	0.63	2.42	-1.95	0.00
XLOC_012413	ZMA (1 of many),granzy	64.87	5.31	3.61	0.00
XLOC_012420	dnajc3a	324.43	53.18	2.61	0.00
XLOC_012430	casp8l2	1.67	7.00	-2.07	0.00
XLOC_012481	ENSONIG00000008973	1.56	10.18	-2.70	0.00
XLOC_012494	ehhadh	6.72	58.50	-3.12	0.00
XLOC_012572	-	2.02	13.04	-2.69	0.00
XLOC_012574	dap1b	11.82	45.95	-1.96	0.00
XLOC_012605	NCOA2 (1 of many)	6.99	2.35	1.57	0.00
XLOC_012654	pitrm1	11.05	2.68	2.05	0.00
XLOC_012686	-	7.42	29.85	-2.01	0.00
XLOC_012689	copa	52.90	18.69	1.50	0.00
XLOC_012728	gba2	3.99	10.30	-1.37	0.00
XLOC_012747	-	0	133.49	-inf	0.00
XLOC_012765	-	64.34	238.97	-1.89	0.00
XLOC_012769	ppib	372.43	104.01	1.84	0.00
XLOC_012820	-	23.71	0	inf	0.00
XLOC_012824	-	7.74	1.75	2.15	0.00
XLOC_012840	tmed9	127.23	49.68	1.36	0.00
XLOC_012853	-	2.43	17.97	-2.89	0.00
XLOC_012871	canx	100.59	29.12	1.79	0.00
XLOC_012886	-	2.62	15.61	-2.58	0.00
XLOC_012887	-	11.03	64.29	-2.54	0.00
XLOC_012890	fam65c	1.01	3.61	-1.84	0.00
XLOC_012898	wu:fb55g09	139.83	23.68	2.56	0.00

XLOC_012930	ENSONIG00000000681	0.28	2.40	-3.12	0.00
XLOC_012957	hgh1	5.07	1.61	1.66	0.00
XLOC_012958	zgc:153675	34.61	7.89	2.13	0.00
XLOC_012968	-	1.72	0	inf	0.00
XLOC_012989	CHAC1 (1 of many)	2.68	21.81	-3.03	0.00
XLOC_013010	PLD4	1.66	16.23	-3.29	0.00
XLOC_013011	srp14	30.18	6.76	2.16	0.00
XLOC_013033	sfxn5a	3.37	19.92	-2.56	0.00
XLOC_013060	exoc3l4	3.69	13.60	-1.88	0.00
XLOC_013062	;ONIG00000012403,tnfa	1.12	7.39	-2.73	0.00
XLOC_013063	-	0	7.02	-inf	0.00
XLOC_013076	SIPA1L1 (1 of many)	0.21	1.25	-2.57	0.00
XLOC_013112	-	1024.96	283.95	1.85	0.00
XLOC_013129	eif2ak3	6.98	1.36	2.36	0.00
XLOC_013135	cfd	8.79	23.77	-1.44	0.00
XLOC_013142	asmt2	0.91	4.70	-2.36	0.00
XLOC_013146	ahsa1b	66.09	6.36	3.38	0.00
XLOC_013147	-	0	41.04	-inf	0.00
XLOC_013160	Mb	154.87	3601.35	-4.54	0.00
XLOC_013175	HMOX1 (1 of many)	16.75	55.26	-1.72	0.00
XLOC_013185	-	33.20	336.98	-3.34	0.00
XLOC_013236	ERP27	5.67	38.02	-2.74	0.00
XLOC_013244	sept3	2.51	7.77	-1.63	0.00
XLOC_013245	-	0	1.50	-inf	0.00
XLOC_013246	serhl (1 of many)	9.41	24.40	-1.37	0.00
XLOC_013304	PGLYRP2 (1 of many)	72.27	11.78	2.62	0.00
XLOC_013314	srp68	20.55	7.73	1.41	0.00
XLOC_013332	rasal3	1.92	6.72	-1.80	0.00
XLOC_013340	nocta	7.80	2.12	1.88	0.00
XLOC_013353	unc93b1	4.41	11.27	-1.35	0.00
XLOC_013370	cpz	0.36	6.36	-4.13	0.00
XLOC_013416	PRF1 (1 of many)	27.88	0.92	4.93	0.00
XLOC_013434	-	0	1.30	-inf	0.00
XLOC_013450	creb3l2	9.98	2.25	2.15	0.00
XLOC_013468	-	9.24	2.02	2.20	0.00
XLOC_013469	kcnj8	6.33	1.85	1.77	0.00
XLOC_013508	-	0	4.01	-inf	0.00
XLOC_013512	itih5	0.95	2.79	-1.56	0.00
XLOC_013544	ENSONIG00000018051	0.89	26.69	-4.91	0.00
XLOC_013564	si:dkey-157g16.6	9.80	3.57	1.46	0.00
XLOC_013569	G6PD (1 of many)	23.65	66.29	-1.49	0.00
XLOC_013572	ssr4	342.32	114.11	1.58	0.00
XLOC_013665	def6a	0.87	3.54	-2.02	0.00
XLOC_013667	-	0	2.07	-inf	0.00
XLOC_013673	avpr2aa	0.50	3.47	-2.79	0.00
XLOC_013688	LRP1 (1 of many)	18.38	6.69	1.46	0.00
XLOC_013748	-	65.19	17.38	1.91	0.00
XLOC_013759	-	11.96	4.04	1.57	0.00

XLOC_013780	uba7	1.16	3.48	-1.59	0.00
XLOC_013810	ENSONIG00000004343	32.06	109.78	-1.78	0.00
XLOC_013824	-	32.79	1.73	4.24	0.00
XLOC_013830	-	49.70	2.21	4.49	0.00
XLOC_013831	-	103.85	2.21	5.55	0.00
XLOC_013834	-	1.82	0	inf	0.00
XLOC_013844	mon1bb	9.88	2.57	1.94	0.00
XLOC_013871	gpx1a	4.17	42.04	-3.33	0.00
XLOC_013891	gmppb	25.21	6.53	1.95	0.00
XLOC_013904	0000004340,ENSONIG0	421.15	2.68	7.30	0.00
XLOC_013944	rabggtb	16.07	6.27	1.36	0.00
XLOC_013948	fam73a	4.48	0.99	2.19	0.00
XLOC_013965	RAB31	85.28	28.82	1.56	0.00
XLOC_013966	vapal	43.08	13.19	1.71	0.00
XLOC_013987	klf2a	2.53	12.20	-2.27	0.00
XLOC_013988	DNAH8	1.77	0.08	4.42	0.00
XLOC_014060	sec62	30.53	12.74	1.26	0.00
XLOC_014081	tet3	3.04	0.32	3.24	0.00
XLOC_014085	add2	1.40	7.19	-2.36	0.00
XLOC_014106	atp2a2a	17.12	5.21	1.72	0.00
XLOC_014144	myl2b	0	0.97	-inf	0.00
XLOC_014201	si:dkey-57h18.2	1.44	4.47	-1.64	0.00
XLOC_014224	psmd11b	8.44	2.30	1.88	0.00
XLOC_014226	ormdl3	22.56	6.94	1.70	0.00
XLOC_014300	nfe2l1b	5.14	1.73	1.57	0.00
XLOC_014302	pnpo	11.80	32.82	-1.48	0.00
XLOC_014319	ANK3 (1 of many)	7.40	35.26	-2.25	0.00
XLOC_014329	-	29.57	7.95	1.89	0.00
XLOC_014353	parp12b	0.41	5.73	-3.79	0.00
XLOC_014373	calr3a	109.18	14.43	2.92	0.00
XLOC_014377	dohh	12.63	5.22	1.28	0.00
XLOC_014379	lonp1	18.21	2.98	2.61	0.00
XLOC_014382	sass6	42.50	137.32	-1.69	0.00
XLOC_014440	calua	149.16	5.22	4.84	0.00
XLOC_014447	ENSONIG00000007684	1.19	6.51	-2.45	0.00
XLOC_014458	dph5	21.79	7.04	1.63	0.00
XLOC_014510	ENSONIG00000004721	9.38	66.33	-2.82	0.00
XLOC_014519	vaspa	1.73	7.63	-2.14	0.00
XLOC_014523	-	0	8.13	-inf	0.00
XLOC_014526	npy	0.74	0	inf	0.00
XLOC_014560	-	11.93	1.65	2.85	0.00
XLOC_014563	ENSONIG00000004728	98.55	360.64	-1.87	0.00
XLOC_014572	hyou1	794.23	87.72	3.18	0.00
XLOC_014579	-	2.76	81.01	-4.88	0.00
XLOC_014637	eif4h	23.27	88.18	-1.92	0.00
XLOC_014661	-	1.54	0	inf	0.00
XLOC_014708	jak3	0.57	2.61	-2.20	0.00
XLOC_014741	RNF213 (1 of many)	0.10	1.51	-3.89	0.00

XLOC_014753	ENSONIG00000012486	0.58	33.01	-5.84	0.00
XLOC_014765	srek1ip1	12.67	3.96	1.68	0.00
XLOC_014773	TBXA2R	6.56	21.82	-1.73	0.00
XLOC_014792	tmem38a	1.72	8.37	-2.28	0.00
XLOC_014798	pde4cb	2.67	0.75	1.83	0.00
XLOC_014923	met	16.38	4.64	1.82	0.00
XLOC_014940	-	1.23	0	inf	0.00
XLOC_014997	cpa1	110.20	709.61	-2.69	0.00
XLOC_015001	ENSONIG00000003919	80.99	641.65	-2.99	0.00
XLOC_015002	ENSONIG00000003921	8.58	103.57	-3.59	0.00
XLOC_015003	-	0	1.12	-inf	0.00
XLOC_015054	TMEM25	11.95	1.05	3.51	0.00
XLOC_015086	psma6l	6.04	21.20	-1.81	0.00
XLOC_015092	-	0	86.41	-inf	0.00
XLOC_015097	si:ch1073-280e3.1	43.93	134.82	-1.62	0.00
XLOC_015140	naa38	14.65	5.59	1.39	0.00
XLOC_015142	-	37.75	3.95	3.26	0.00
XLOC_015143	-	50.26	4.50	3.48	0.00
XLOC_015160	shbg	247.60	766.80	-1.77	0.00
XLOC_015170	per1b	14.29	0.63	4.49	0.00
XLOC_015185	dnajc3b	11.11	4.05	1.46	0.00
XLOC_015190	-	1.32	49.96	-5.24	0.00
XLOC_015194	-	0	3.14	-inf	0.00
XLOC_015196	-	0	8.50	-inf	0.00
XLOC_015227	ENSONIG00000019072	1.24	0	inf	0.00
XLOC_015237	-	44.17	0	inf	0.00
XLOC_015295	heatr1	7.15	1.36	2.40	0.00
XLOC_015312	ankrd22	94.75	13.51	2.81	0.00
XLOC_015336	esd	5.39	21.00	-1.96	0.00
XLOC_015371	lacc1	2.01	5.94	-1.57	0.00
XLOC_015372	lcp1	10.72	34.10	-1.67	0.00
XLOC_015433	fnbp1b	3.04	14.68	-2.27	0.00
XLOC_015439	mrps2	22.39	6.60	1.76	0.00
XLOC_015479	dck,slc4a4b	7.67	1.61	2.26	0.00
XLOC_015503	hhip	7.51	24.84	-1.73	0.00
XLOC_015539	tnip2	0.46	5.54	-3.59	0.00
XLOC_015569	uchl1	59.75	8.40	2.83	0.00
XLOC_015570	prom1b	12.86	34.60	-1.43	0.00
XLOC_015616	-	69.57	25.04	1.47	0.00
XLOC_015634	net1	5.42	1.84	1.56	0.00
XLOC_015637	ENSONIG00000011980	3.90	10.75	-1.46	0.00
XLOC_015639	slc41a2b	14.12	6.05	1.22	0.00
XLOC_015662	-	2.38	11.03	-2.21	0.00
XLOC_015664	-	0	9.66	-inf	0.00
XLOC_015666	-	2.67	0	inf	0.00
XLOC_015697	mmp13a (1 of many)	13.58	2.53	2.42	0.00
XLOC_015701	tmprss2	8.96	40.69	-2.18	0.00
XLOC_015753	ust	4.41	0.99	2.15	0.00

XLOC_015781	daam1b	5.61	19.45	-1.79	0.00
XLOC_015819	acbd3	19.07	4.94	1.95	0.00
XLOC_015836	fdft1	3.26	33.64	-3.37	0.00
XLOC_015849	gne	2.05	9.68	-2.24	0.00
XLOC_015868	-	17.12	204.51	-3.58	0.00
XLOC_015874	-	0	21.07	-inf	0.00
XLOC_015879	hmox2a	52.00	14.24	1.87	0.00
XLOC_015881	-	27.92	7.44	1.91	0.00
XLOC_015887	slc27a1b	12.00	2.85	2.07	0.00
XLOC_015888	si:ch211-114l13.7	3.78	0.95	1.99	0.00
XLOC_015895	C3 (1 of many)	11.65	84.22	-2.85	0.00
XLOC_015947	-	0	1.75	-inf	0.00
XLOC_015949	dnajb1b	108.20	2.43	5.47	0.00
XLOC_015973	slc25a10	26.15	8.17	1.68	0.00
XLOC_015987	dnaja3a	18.81	4.65	2.02	0.00
XLOC_015995	-	194.85	61.91	1.65	0.00
XLOC_016002	-	1.32	11.47	-3.12	0.00
XLOC_016074	btbd17b,map2k6	1.76	6.24	-1.82	0.00
XLOC_016109	gbe1b	11.86	40.92	-1.79	0.00
XLOC_016118	FNDC3A	23.43	7.17	1.71	0.00
XLOC_016123	thrsp	362.59	3920.78	-3.43	0.00
XLOC_016152	usp16	7.48	1.38	2.43	0.00
XLOC_016163	treh	0.32	3.47	-3.42	0.00
XLOC_016193	idi1	5.81	25.33	-2.12	0.00
XLOC_016198	CCNY (1 of many)	0.83	3.80	-2.20	0.00
XLOC_016262	ankmy2a	5.50	1.49	1.89	0.00
XLOC_016265	-	2.64	0	inf	0.00
XLOC_016272	:h211-215k15.4 (1 of ma	14.03	4.92	1.51	0.00
XLOC_016279	tmod4	3.85	13.26	-1.79	0.00
XLOC_016308	sec24d	39.91	11.85	1.75	0.00
XLOC_016329	DHX15 (1 of many)	14.56	5.87	1.31	0.00
XLOC_016334	epdl1	6.30	70.64	-3.54	0.00
XLOC_016358	-	20.44	64.78	-1.66	0.00
XLOC_016403	-	0	2.69	-inf	0.00
XLOC_016416	zgc:85789	22.75	56.01	-1.30	0.00
XLOC_016418	stip1	21.14	5.10	2.05	0.00
XLOC_016425	tm7sf2	26.93	98.89	-1.88	0.00
XLOC_016436	pcolcea	3.59	31.88	-3.15	0.00
XLOC_016445	acaca	11.21	54.58	-2.28	0.00
XLOC_016447	ksr1a	0.93	3.27	-1.81	0.00
XLOC_016480	-	0	1.86	-inf	0.00
XLOC_016495	-	29.56	112.40	-1.93	0.00
XLOC_016505	cyp4t8	16.08	117.92	-2.87	0.00
XLOC_016514	stk40	2.14	7.39	-1.79	0.00
XLOC_016535	ENSONIG00000016150	84.45	370.95	-2.14	0.00
XLOC_016540	si:ch211-79k12.1	10.05	39.88	-1.99	0.00
XLOC_016570	srsf10a	9.72	2.73	1.83	0.00
XLOC_016573	-	1.38	0	inf	0.00

XLOC_016580	-	101.44	276.34	-1.45	0.00
XLOC_016581	dhdds	25.01	4.53	2.46	0.00
XLOC_016601	ntd5	10.57	2.31	2.19	0.00
XLOC_016667	vars	8.90	3.67	1.28	0.00
XLOC_016718	si:dkey-26c10.5	5.68	15.29	-1.43	0.00
XLOC_016719	-	420.83	117.08	1.85	0.00
XLOC_016734	0000012949,ENSONIG0	11.99	45.18	-1.91	0.00
XLOC_016748	-	0	3.04	-inf	0.00
XLOC_016756	pparaa	41.65	12.95	1.69	0.00
XLOC_016760	-	4.73	13.61	-1.53	0.00
XLOC_016803	cdkn1d	2.84	10.74	-1.92	0.00
XLOC_016817	dnajb9b	148.55	8.65	4.10	0.00
XLOC_016836	GRAMD2 (1 of many)	1.48	10.93	-2.88	0.00
XLOC_016870	coro2ba	0.50	3.14	-2.64	0.00
XLOC_016893	-	0	71.77	-inf	0.00
XLOC_016918	egln2	69.76	14.29	2.29	0.00
XLOC_016923	ENSONIG00000006056	0.81	3.70	-2.20	0.00
XLOC_016964	dock10	0.26	1.69	-2.68	0.00
XLOC_016982	glipr2l	2.06	13.69	-2.74	0.00
XLOC_016986	-	37.48	4.07	3.20	0.00
XLOC_016988	stx17	24.30	7.94	1.61	0.00
XLOC_016993	pdp1	400.12	32.19	3.64	0.00
XLOC_017010	ENSONIG00000006225	30.80	79.05	-1.36	0.00
XLOC_017030	fdps	2.40	18.13	-2.92	0.00
XLOC_017047	setdb1b	0.97	2.73	-1.49	0.00
XLOC_017067	ENSONIG00000005955	13.45	35.03	-1.38	0.00
XLOC_017068	steap4 (1 of many)	16.73	428.31	-4.68	0.00
XLOC_017079	alg2	20.28	5.40	1.91	0.00
XLOC_017099	gtpbp10	9.92	2.76	1.85	0.00
XLOC_017105	-	18.67	5.93	1.65	0.00
XLOC_017111	aqp10a	14.24	64.11	-2.17	0.00
XLOC_017145	GZMA (1 of many)	1.35	10.71	-2.99	0.00
XLOC_017147	slc20a2	11.68	3.51	1.73	0.00
XLOC_017148	lipg	2.81	102.87	-5.19	0.00
XLOC_017157	-	4.94	21.07	-2.09	0.00
XLOC_017169	dynll1	153.83	58.21	1.40	0.00
XLOC_017179	-	0	3.87	-inf	0.00
XLOC_017185	bhmt	170.59	1465.26	-3.10	0.00
XLOC_017213	ARHGEF28	7.85	29.32	-1.90	0.00
XLOC_017214	utp15	9.09	2.82	1.69	0.00
XLOC_017236	slc23a2	18.34	3.92	2.22	0.00
XLOC_017243	-	0	9.99	-inf	0.00
XLOC_017244	-	5.59	67.39	-3.59	0.00
XLOC_017247	ENSONIG00000014463	13.97	2.64	2.40	0.00
XLOC_017280	ethe1 (1 of many)	88.24	9.09	3.28	0.00
XLOC_017291	ENSONIG00000007420	0	2.80	-inf	0.00
XLOC_017326	ENSONIG00000007485	0.12	3.14	-4.76	0.00
XLOC_017328	-	135.15	0	inf	0.00

XLOC_017345	pfn1	23.10	82.02	-1.83	0.00
XLOC_017359	yipf6	24.72	5.83	2.08	0.00
XLOC_017360	ENSONIG00000001775	5.68	0.92	2.63	0.00
XLOC_017370	scfd1	47.33	18.82	1.33	0.00
XLOC_017376	ENSONIG00000001722	0.57	2.96	-2.37	0.00
XLOC_017385	egln3	100.36	6.48	3.95	0.00
XLOC_017447	fli1b	0.97	2.93	-1.60	0.00
XLOC_017451	mgat1a	61.66	20.20	1.61	0.00
XLOC_017539	zgc:77486	28.35	8.18	1.79	0.00
XLOC_017550	fkbp8	35.67	9.80	1.86	0.00
XLOC_017551	ell	4.62	1.09	2.09	0.00
XLOC_017608	rorca	25.25	5.36	2.24	0.00
XLOC_017660	faxdc2	88.15	438.89	-2.32	0.00
XLOC_017678	irf1b	2.70	77.15	-4.84	0.00
XLOC_017679	-	0	33.55	-inf	0.00
XLOC_017698	fgfr4	10.92	0.69	3.99	0.00
XLOC_017723	phyhd1	8.53	26.92	-1.66	0.00
XLOC_017728	SEC16A	8.23	3.35	1.30	0.00
XLOC_017737	skiv2l2	20.31	3.89	2.38	0.00
XLOC_017742	-	59.47	170.59	-1.52	0.00
XLOC_017751	lman1	89.18	28.77	1.63	0.00
XLOC_017760	C18orf32	47.46	16.85	1.49	0.00
XLOC_017761	dym	7.68	2.73	1.49	0.00
XLOC_017767	-	15.27	98.59	-2.69	0.00
XLOC_017803	amacr	3.59	13.23	-1.88	0.00
XLOC_017816	nedd4l	29.07	7.69	1.92	0.00
XLOC_017843	klf12b	6.60	1.21	2.45	0.00
XLOC_017875	ENSONIG00000006700	2.44	6.29	-1.37	0.00
XLOC_017881	mid1ip1a	118.20	2.96	5.32	0.00
XLOC_017893	gpr18	0.22	2.65	-3.56	0.00
XLOC_017929	mat2ab	16.59	130.20	-2.97	0.00
XLOC_017933	si:ch211-173b16.3	6.47	19.25	-1.57	0.00
XLOC_017943	pik3ip1	3.71	13.41	-1.86	0.00
XLOC_017979	erap2	1.34	5.73	-2.09	0.00
XLOC_017992	PCSK5	54.63	21.46	1.35	0.00
XLOC_018010	tcn2	97.20	27.38	1.83	0.00
XLOC_018046	acacb	2.32	5.97	-1.36	0.00
XLOC_018054	si:ch211-204d2.4	1.67	4.94	-1.56	0.00
XLOC_018076	-	4.79	29.26	-2.61	0.00
XLOC_018092	syvn1	99.23	19.19	2.37	0.00
XLOC_018095	LONRF3	2.69	0.74	1.86	0.00
XLOC_018108	-	0	25.58	-inf	0.00
XLOC_018110	ENSONIG00000015752	137.09	9.93	3.79	0.00
XLOC_018114	ENSONIG00000015762	1.18	5.87	-2.31	0.00
XLOC_018118	ctsf	67.26	206.62	-1.62	0.00
XLOC_018152	fam210b	5.48	13.77	-1.33	0.00
XLOC_018160	soat2	4.00	52.27	-3.71	0.00
XLOC_018172	PDE1B	1.33	30.97	-4.54	0.00

XLOC_018209	igsf8	1.61	6.57	-2.03	0.00
XLOC_018222	pigu	3.42	0.89	1.94	0.00
XLOC_018284	CNNM1 (1 of many)	5.68	0.11	5.66	0.00
XLOC_018298	zfand4	2.30	9.77	-2.09	0.00
XLOC_018331	ppifa	3.01	12.65	-2.07	0.00
XLOC_018336	comtd1	242.45	895.97	-1.89	0.00
XLOC_018344	hpse2	1.08	0	inf	0.00
XLOC_018346	got1	272.90	29.49	3.21	0.00
XLOC_018369	hif1an	8.67	2.09	2.05	0.00
XLOC_018372	scdb	416.75	1.50	8.12	0.00
XLOC_018375	tysnd1	12.94	3.90	1.73	0.00
XLOC_018418	-	7.27	32.65	-2.17	0.00
XLOC_018478	deptor	15.46	4.35	1.83	0.00
XLOC_018500	tcea3	3.93	11.07	-1.50	0.00
XLOC_018526	-	31.31	10.19	1.62	0.00
XLOC_018527	-	32.50	11.05	1.56	0.00
XLOC_018593	-	2363.50	137.93	4.10	0.00
XLOC_018643	fam13a	184.54	20.65	3.16	0.00
XLOC_018647	uso1	23.81	5.58	2.09	0.00
XLOC_018650	-	28.07	94.29	-1.75	0.00
XLOC_018663	papss1	12.58	5.12	1.30	0.00
XLOC_018676	mogs	23.98	7.49	1.68	0.00
XLOC_018725	si:ch211-217k17.7	8.45	3.02	1.48	0.00
XLOC_018730	ufsp2	20.65	6.67	1.63	0.00
XLOC_018735	vrk2	2.17	5.84	-1.43	0.00
XLOC_018745	haao	49.57	151.62	-1.61	0.00
XLOC_018757	rims1a	9.28	2.73	1.77	0.00
XLOC_018764	-	2.76	13.43	-2.28	0.00
XLOC_018765	RNF122 (1 of many)	1.54	7.96	-2.37	0.00
XLOC_018783	-	0	6.34	-inf	0.00
XLOC_018799	SYDE2	2.12	0.34	2.65	0.00
XLOC_018819	alg6	11.61	3.36	1.79	0.00
XLOC_018822	angptl3 (1 of many)	250.73	42.15	2.57	0.00
XLOC_018882	cct7	57.71	19.80	1.54	0.00
XLOC_018920	-	0.33	5.70	-4.13	0.00
XLOC_018933	clic1	1.29	3.93	-1.60	0.00
XLOC_018983	cyp51	30.11	178.63	-2.57	0.00
XLOC_019017	ENSONIG00000019706	41.44	3.38	3.62	0.00
XLOC_019043	si:dkey-266f7.9	2.87	17.67	-2.62	0.00
XLOC_019059	-	1.35	21.32	-3.98	0.00
XLOC_019070	MCOLN2	28.90	10.96	1.40	0.00
XLOC_019109	ENSONIG00000007355	0.82	60.65	-6.20	0.00
XLOC_019110	ENSONIG00000007369	1.20	227.55	-7.56	0.00
XLOC_019118	rhogb	11.02	32.31	-1.55	0.00
XLOC_019156	KIAA0895L	3.79	0.80	2.24	0.00
XLOC_019176	pdlim2	14.76	1.36	3.44	0.00
XLOC_019180	GFPT1	93.28	15.04	2.63	0.00
XLOC_019186	ido1	8.79	148.92	-4.08	0.00

XLOC_019232	gda	79.94	8.23	3.28	0.00
XLOC_019286	-	0	12.72	-inf	0.00
XLOC_019366	-	0	13.29	-inf	0.00
XLOC_019371	ENSONIG00000012337	7.27	1.86	1.97	0.00
XLOC_019375	ENSONIG00000021042	1.48	0	inf	0.00
XLOC_019379	si:ch211-235o23.1	2.93	8.21	-1.49	0.00
XLOC_019389	-	0	3.17	-inf	0.00
XLOC_019408	ENSONIG00000002020	3.62	116.07	-5.00	0.00
XLOC_019410	NACC1 (1 of many)	2.15	6.08	-1.50	0.00
XLOC_019416	LGALS1 (1 of many)	27.37	130.23	-2.25	0.00
XLOC_019422	ankrd40	5.87	1.27	2.21	0.00
XLOC_019423	wfikkn2a	1.26	0	inf	0.00
XLOC_019424	-	1.12	0	inf	0.00
XLOC_019428	-	0.63	7.09	-3.50	0.00
XLOC_019430	-	0	3.97	-inf	0.00
XLOC_019474	ENSONIG00000002150	3.36	21.65	-2.69	0.00
XLOC_019629	fynb	0.27	3.64	-3.74	0.00
XLOC_019659	marveld2a	9.31	25.60	-1.46	0.00
XLOC_019660	pi4kaa	4.83	1.71	1.50	0.00
XLOC_019675	marcksa	3.76	13.10	-1.80	0.00
XLOC_019679	slc16a10	16.44	49.58	-1.59	0.00
XLOC_019708	rtn1b	1.98	11.38	-2.52	0.00
XLOC_019710	tacc3	0.95	5.70	-2.58	0.00
XLOC_019714	hspa4l	2.13	0	inf	0.00
XLOC_019745	flvcr2a	1.54	4.39	-1.51	0.00
XLOC_019759	hif1aa	31.94	12.07	1.40	0.00
XLOC_019789	aspg	6.82	35.55	-2.38	0.00
XLOC_019820	c2h1orf27	57.31	14.99	1.93	0.00
XLOC_019822	ptbp2b	1.94	5.71	-1.56	0.00
XLOC_019827	tox	9.40	1.65	2.51	0.00
XLOC_019843	ENSONIG00000016571	0.79	3.47	-2.13	0.00
XLOC_019873	lancl1	1.18	4.03	-1.77	0.00
XLOC_019924	syncripl	11.79	3.89	1.60	0.00
XLOC_019931	rsad2	7.39	46.86	-2.67	0.00
XLOC_019949	ctsl.1 (1 of many)	5.04	18.05	-1.84	0.00
XLOC_019953	rps6ka1	1.00	5.04	-2.34	0.00
XLOC_019958	SLC25A29 (1 of many)	12.58	2.67	2.24	0.00
XLOC_019960	WARS	18.90	5.28	1.84	0.00
XLOC_019983	KLF11 (1 of many)	6.93	35.84	-2.37	0.00
XLOC_019994	hsd17b7 (1 of many)	5.19	17.06	-1.72	0.00
XLOC_020036	-	0	1.08	-inf	0.00
XLOC_020044	sgut1	49.61	19.13	1.37	0.00
XLOC_020046	ENSONIG00000018709	0	2.13	-inf	0.00
XLOC_020049	ENSONIG00000021257	0	1.16	-inf	0.00
XLOC_020088	gorasp2	14.69	3.56	2.04	0.00
XLOC_020094	-	3.00	17.97	-2.58	0.00
XLOC_020153	lrrc59	21.17	5.03	2.07	0.00
XLOC_020156	-	0	62.70	-inf	0.00

XLOC_020210	emsy	3.36	1.18	1.50	0.00
XLOC_020229	-	2.28	14.85	-2.70	0.00
XLOC_020232	cldn2	13.04	40.31	-1.63	0.00
XLOC_020247	-	1.96	37.98	-4.28	0.00
XLOC_020260	me1	1.52	88.04	-5.86	0.00
XLOC_020283	ibtk	10.04	27.60	-1.46	0.00
XLOC_020299	sqlea	1.57	42.82	-4.77	0.00
XLOC_020306	-	42.58	0	inf	0.00
XLOC_020325	-	3993.01	895.41	2.16	0.00
XLOC_020328	-	1.35	0	inf	0.00
XLOC_020333	si:ch211-284e20.8	65.18	213.17	-1.71	0.00
XLOC_020336	SERPINB1 (1 of many)	24.15	7.34	1.72	0.00
XLOC_020347	rgs4	11.49	1.04	3.46	0.00
XLOC_020377	ENSONIG00000010526	38.82	102.66	-1.40	0.00
XLOC_020406	ppp1r3b	20.82	124.80	-2.58	0.00
XLOC_020407	-	1.96	9.65	-2.30	0.00
XLOC_020423	-	2.87	0	inf	0.00
XLOC_020433	spra	120.78	47.54	1.35	0.00
XLOC_020443	pisd	43.64	118.12	-1.44	0.00
XLOC_020457	-	0	2.20	-inf	0.00
XLOC_020464	dmtn (1 of many)	2.48	10.53	-2.09	0.00
XLOC_020481	ufc1	75.58	14.91	2.34	0.00
XLOC_020493	-	116.54	654.67	-2.49	0.00
XLOC_020504	-	1.40	6.98	-2.31	0.00
XLOC_020521	glut1	24.22	3.23	2.91	0.00
XLOC_020525	-	2.59	10.14	-1.97	0.00
XLOC_020566	-	0	1.92	-inf	0.00
XLOC_020581	sc5d	22.38	131.79	-2.56	0.00
XLOC_020584	usp2b	16.62	3.37	2.30	0.00
XLOC_020604	-	32.73	0.64	5.68	0.00
XLOC_020605	-	54.44	0	inf	0.00
XLOC_020625	-	2.53	16.96	-2.75	0.00
XLOC_020627	-	21.05	60.02	-1.51	0.00
XLOC_020634	si:dkey-28e7.3	15.00	1.29	3.54	0.00
XLOC_020649	sec23b	49.98	8.10	2.63	0.00
XLOC_020653	XYLT1 (1 of many)	0.18	1.26	-2.84	0.00
XLOC_020658	KIAA0430	3.34	9.49	-1.51	0.00
XLOC_020662	klf1	1.49	16.10	-3.43	0.00
XLOC_020685	-	4.39	0.95	2.21	0.00
XLOC_020718	slc44a2	2.34	10.74	-2.20	0.00
XLOC_020719	acp5a	1.88	12.25	-2.71	0.00
XLOC_020728	lrpap1	32.87	8.43	1.96	0.00
XLOC_020758	SUSD2 (1 of many)	1.75	7.90	-2.17	0.00
XLOC_020762	-	359.38	147.70	1.28	0.00
XLOC_020775	UHRF2	9.01	2.56	1.81	0.00
XLOC_020794	tgfbr2	17.25	6.31	1.45	0.00
XLOC_020796	brf2	6.88	1.57	2.13	0.00
XLOC_020822	si:ch73-209e20.3	223.79	1.90	6.88	0.00

XLOC_020851	-	0	2.75	-inf	0.00
XLOC_020909	-	0	2.57	-inf	0.00
XLOC_020921	tufm	34.57	14.50	1.25	0.00
XLOC_020931	recql5,si:ch211-120g10.1	1.71	5.00	-1.55	0.00
XLOC_020973	sec14l1	4.99	21.00	-2.07	0.00
XLOC_020998	-	1.84	0	inf	0.00
XLOC_020999	-	3.52	0	inf	0.00
XLOC_021002	-	10.90	0	inf	0.00
XLOC_021017	-	0	29.74	-inf	0.00
XLOC_021019	mtr	16.87	6.51	1.37	0.00
XLOC_021024	cyp26a1	1.51	19.73	-3.70	0.00
XLOC_021031	ipmkb	10.27	1.57	2.71	0.00
XLOC_021078	-	15.77	2.98	2.41	0.00
XLOC_021087	XPO1 (1 of many)	17.13	2.51	2.77	0.00
XLOC_021136	SUSD2 (1 of many)	3.25	9.56	-1.56	0.00
XLOC_021146	wsb2	72.06	19.68	1.87	0.00
XLOC_021173	hspb8	256.13	99.85	1.36	0.00
XLOC_021176	pebp1	29.29	101.04	-1.79	0.00
XLOC_021181	-	2.73	27.33	-3.32	0.00
XLOC_021218	-	0	1.00	-inf	0.00
XLOC_021223	lonrf1l	11.01	2.88	1.94	0.00
XLOC_021240	cisd2	9.13	2.82	1.70	0.00
XLOC_021256	dnase1l3 (1 of many)	58.81	13.41	2.13	0.00
XLOC_021288	ONIG00000017947,denn	3.67	15.95	-2.12	0.00
XLOC_021302	si:ch73-302a13.2	9.28	3.40	1.45	0.00
XLOC_021305	plod1a	17.45	3.22	2.44	0.00
XLOC_021312	tbc1d25	2.77	7.11	-1.36	0.00
XLOC_021319	timm17a	30.01	7.87	1.93	0.00
XLOC_021334	zmp:0000000758	17.63	52.48	-1.57	0.00
XLOC_021339	-	240.56	52.43	2.20	0.00
XLOC_021347	si:ch211-195b11.7	23.94	2.45	3.29	0.00
XLOC_021352	tubb1	15.03	37.89	-1.33	0.00
XLOC_021368	-	1.08	9.86	-3.19	0.00
XLOC_021403	ero1a	86.37	20.25	2.09	0.00
XLOC_021426	zgc:171592	15.98	169.68	-3.41	0.00
XLOC_021443	0000002804,ENSONIG0	18.80	77.18	-2.04	0.00
XLOC_021444	EPB41 (1 of many)	10.07	51.70	-2.36	0.00
XLOC_021456	ankrd28b,hacl1	14.33	46.29	-1.69	0.00
XLOC_021470	dfna5b	0.57	6.50	-3.52	0.00
XLOC_021498	igflr1	2.80	13.63	-2.28	0.00
XLOC_021508	AIF1	14.67	50.97	-1.80	0.00
XLOC_021518	chn2	1.00	5.29	-2.40	0.00
XLOC_021524	mpp6a	4.68	12.28	-1.39	0.00
XLOC_021532	ptdss1a	1.59	12.84	-3.01	0.00
XLOC_021563	galnt2	6.55	2.02	1.70	0.00
XLOC_021567	efcab2	0.97	0	inf	0.00
XLOC_021578	ENSONIG00000020553	3.92	16.46	-2.07	0.00
XLOC_021603	adpgk2	33.86	12.64	1.42	0.00

XLOC_021636	-	0	2.53	-inf	0.00
XLOC_021672	-	4.67	1.10	2.08	0.00
XLOC_021675	-	1.80	0	inf	0.00
XLOC_021679	-	0	3.12	-inf	0.00
XLOC_021699	TUBB (1 of many)	63.26	2.66	4.57	0.00
XLOC_021741	ENSONIG00000021373	8.18	85.31	-3.38	0.00
XLOC_021780	ccr7	0.97	6.35	-2.72	0.00
XLOC_021785	-	3.04	0	inf	0.00
XLOC_021820	-	51.19	8.06	2.67	0.00
XLOC_021827	-	5.00	0.82	2.60	0.00
XLOC_021828	-	11.86	0	inf	0.00
XLOC_021837	-	0	26.77	-inf	0.00
XLOC_021858	ENSONIG00000013714	9.11	54.05	-2.57	0.00
XLOC_021912	-	1.11	5.92	-2.41	0.00
XLOC_021957	sts	3.51	10.26	-1.55	0.00
XLOC_021960	ENSONIG00000003713	15.15	62.33	-2.04	0.00
XLOC_021985	ENSONIG00000007831	0.73	112.45	-7.26	0.00
XLOC_021990	-	1.39	0	inf	0.00
XLOC_021994	-	95.18	0	inf	0.00
XLOC_022021	-	75.16	0	inf	0.00
XLOC_022027	ENSONIG00000007859	6.47	1.28	2.33	0.00
XLOC_022094	ENSONIG00000003662	0.68	9.87	-3.87	0.00
XLOC_022098	ENSONIG00000003675	1.48	5.27	-1.84	0.00
XLOC_022122	tcaim	12.83	4.47	1.52	0.00
XLOC_022198	ENSONIG00000016996	0.24	10.19	-5.44	0.00
XLOC_022200	ENSONIG00000016999	90.48	13.98	2.69	0.00
XLOC_022214	si:dkey-91i10.3	14.13	56.42	-2.00	0.00
XLOC_022238	slc2a6	0	1.17	-inf	0.00
XLOC_022254	desi1a	8.03	28.55	-1.83	0.00
XLOC_022255	llgl1	27.20	9.01	1.59	0.00
XLOC_022272	-	42.52	9.72	2.13	0.00
XLOC_022273	ENSONIG00000015704	16.59	3.98	2.06	0.00
XLOC_022277	PRKCB (1 of many)	0.14	1.15	-3.03	0.00
XLOC_022279	-	0.80	4.41	-2.46	0.00
XLOC_022308	TMEM38B	95.07	26.68	1.83	0.00
XLOC_022320	-	14.41	0	inf	0.00
XLOC_022321	-	8.45	0	inf	0.00
XLOC_022322	-	3.28	0	inf	0.00
XLOC_022324	znf618	5.68	1.89	1.59	0.00
XLOC_022349	COL13A1	0.74	7.81	-3.40	0.00
XLOC_022351	gbf1	32.91	13.01	1.34	0.00
XLOC_022365	-	0	16.63	-inf	0.00
XLOC_022403	-	0	18.54	-inf	0.00
XLOC_022404	-	0.81	6.96	-3.11	0.00
XLOC_022417	tnrc5	43.37	8.01	2.44	0.00
XLOC_022419	-	0	2.76	-inf	0.00
XLOC_022459	acad8	7.88	2.47	1.67	0.00
XLOC_022491	med13b	20.97	3.85	2.45	0.00

XLOC_022533	ENSONIG00000015298	0.44	21.35	-5.59	0.00
XLOC_022602	-	377.85	142.68	1.41	0.00
XLOC_022693	arl4ab	0.90	5.77	-2.67	0.00
XLOC_022706	GLDC	129.36	13.90	3.22	0.00
XLOC_022711	gas1a	1.43	11.87	-3.06	0.00
XLOC_022722	CCNG2 (1 of many)	5.94	18.22	-1.62	0.00
XLOC_022726	ENSONIG00000016924	33.96	7.47	2.19	0.00
XLOC_022738	aacs	1.12	4.13	-1.88	0.00
XLOC_022740	erap1b	3.04	7.21	-1.25	0.00
XLOC_022743	tars	29.10	11.15	1.38	0.00
XLOC_022796	ddost	381.63	103.98	1.88	0.00
XLOC_022862	vtg3	6.46	182.26	-4.82	0.00
XLOC_022868	kif21a	0.69	7.73	-3.48	0.00
XLOC_022871	cpa4	106.78	551.27	-2.37	0.00
XLOC_022882	-	2.44	8.20	-1.75	0.00
XLOC_022903	mpi	3.12	1.02	1.61	0.00
XLOC_022905	ampd3b	0.98	7.78	-2.99	0.00
XLOC_022917	-	144.39	31.98	2.17	0.00
XLOC_022920	pik3c2a	2.18	4.92	-1.18	0.00
XLOC_022924	abhd2b	9.27	2.20	2.08	0.00
XLOC_022980	-	44.70	211.97	-2.25	0.00
XLOC_023015	brd3b	11.37	4.49	1.34	0.00
XLOC_023026	-	3.38	0	inf	0.00
XLOC_023038	ENSONIG00000000873	1.50	4.16	-1.47	0.00
XLOC_023058	chek2	22.50	5.99	1.91	0.00
XLOC_023097	-	0	1.85	-inf	0.00
XLOC_023221	tdh	268.31	40.73	2.72	0.00
XLOC_023237	ENSONIG00000014146	9.39	28.09	-1.58	0.00
XLOC_023273	rint1	17.97	5.48	1.71	0.00
XLOC_023290	slc22a15	10.12	27.72	-1.45	0.00
XLOC_023312	-	18.81	0	inf	0.00
XLOC_023427	sh3bgrl	8.91	28.10	-1.66	0.00
XLOC_023428	ENSONIG00000017648	1.00	7.29	-2.87	0.00
XLOC_023438	crtac1a	4.77	1.03	2.22	0.00
XLOC_023553	-	102.17	312.90	-1.61	0.00
XLOC_023573	ENSONIG00000009615	1001.29	18.30	5.77	0.00
XLOC_023575	-	4.52	0	inf	0.00
XLOC_023602	si:ch211-132g1.7	5.97	1.47	2.03	0.00
XLOC_023617	-	0	19.87	-inf	0.00
XLOC_023619	arhgef7b	0.76	2.41	-1.67	0.00
XLOC_023717	ern2	7.12	0.91	2.97	0.00
XLOC_023727	socs1b	1.03	7.21	-2.81	0.00
XLOC_023732	pdia2	2.43	26.99	-3.48	0.00
XLOC_023735	farp1	5.64	1.80	1.65	0.00
XLOC_023753	-	2.25	0	inf	0.00
XLOC_023754	-	5.47	0	inf	0.00
XLOC_023764	cbsb	1477.04	180.92	3.03	0.00
XLOC_023765	ENSONIG00000001903	74.99	27.40	1.45	0.00

XLOC_023796	nus1	22.02	5.74	1.94	0.00
XLOC_023804	-	0	1.63	-inf	0.00
XLOC_023874	znfx1	0.67	2.69	-2.02	0.00
XLOC_023875	-	0	17.14	-inf	0.00
XLOC_023882	gls2b	1.81	6.05	-1.74	0.00
XLOC_023891	-	0	13.63	-inf	0.00
XLOC_023895	-	0	23.22	-inf	0.00
XLOC_023915	NFATC2 (1 of many)	0.38	2.53	-2.75	0.00
XLOC_023916	cyp24a1	12.20	3.15	1.95	0.00
XLOC_023929	zgc:63645	30.19	6.92	2.13	0.00
XLOC_023942	ncor1	8.31	27.39	-1.72	0.00
XLOC_023981	msna	5.75	15.00	-1.38	0.00
XLOC_024048	ENSONIG00000020507	187.72	21.60	3.12	0.00
XLOC_024055	ENSONIG00000017404	5.60	26.40	-2.24	0.00
XLOC_024085	-	3.24	0	inf	0.00
XLOC_024099	manf	411.46	72.44	2.51	0.00
XLOC_024109	ENSONIG00000008188	6.98	1.02	2.77	0.00
XLOC_024116	sat1b	20.48	55.01	-1.43	0.00
XLOC_024179	hspe1,mob4	294.38	53.89	2.45	0.00
XLOC_024204	hspd1	84.89	14.94	2.51	0.00
XLOC_024207	dnajb11	141.17	19.96	2.82	0.00
XLOC_024226	xaf1	0.24	2.61	-3.45	0.00
XLOC_024241	slc43a2a	5.01	55.28	-3.46	0.00
XLOC_024250	smtnl	4.96	0.37	3.73	0.00
XLOC_024268	ern1	22.59	9.14	1.31	0.00
XLOC_024281	GCNT1 (1 of many)	3.60	14.73	-2.03	0.00
XLOC_024290	KDELR2 (1 of many)	11.15	3.20	1.80	0.00
XLOC_024325	pld3	2.70	6.62	-1.30	0.00
XLOC_024349	blvrb	31.33	126.59	-2.01	0.00
XLOC_024360	MAP4K1	0.94	4.01	-2.10	0.00
XLOC_024363	capn5a	2.68	6.86	-1.36	0.00
XLOC_024369	rhbdd1 (1 of many)	2.19	6.81	-1.64	0.00
XLOC_024374	-	4.91	0	inf	0.00
XLOC_024383	0000001531,ENSONIG0	2.10	51.16	-4.61	0.00
XLOC_024407	ENSONIG00000001512	0.30	8.02	-4.73	0.00
XLOC_024459	tec	0.67	2.93	-2.12	0.00
XLOC_024469	rnf150b	2.25	6.10	-1.44	0.00
XLOC_024477	ENSONIG00000011706	4.04	11.85	-1.55	0.00
XLOC_024480	-	13.47	0	inf	0.00
XLOC_024481	-	5.53	0	inf	0.00
XLOC_024483	slc10a7	11.05	2.64	2.06	0.00
XLOC_024492	hmgb2a	33.34	92.40	-1.47	0.00
XLOC_024518	-	3.58	0.62	2.53	0.00
XLOC_024520	-	13.65	2.51	2.44	0.00
XLOC_024548	fgf19	4.45	0.75	2.57	0.00
XLOC_024577	oraov1	25.71	7.96	1.69	0.00
XLOC_024587	ero1b	54.12	16.41	1.72	0.00
XLOC_024588	gstt1b	46.59	214.02	-2.20	0.00

XLOC_024625	wdr3	6.15	1.96	1.65	0.00
XLOC_024627	hao2,si:rp71-68n21.9	4.33	29.27	-2.76	0.00
XLOC_024638	cx30.3	2.44	9.70	-1.99	0.00
XLOC_024660	-	0	4.37	-inf	0.00
XLOC_024669	-	0	8.30	-inf	0.00
XLOC_024680	si:ch73-111k22.2	16.22	4.20	1.95	0.00
XLOC_024681	cx28.9	81.31	14.00	2.54	0.00
XLOC_024682	cx32.3	93.88	11.73	3.00	0.00
XLOC_024703	ENSONIG00000004731	24.20	91.88	-1.92	0.00
XLOC_024708	abca4a	1.87	0.51	1.88	0.00
XLOC_024711	dph2	7.25	1.53	2.24	0.00
XLOC_024762	-	22.33	3.71	2.59	0.00
XLOC_024764	-	52.36	3.83	3.77	0.00
XLOC_024767	-	1.93	0	inf	0.00
XLOC_024771	-	3.22	0	inf	0.00
XLOC_024773	-	3.28	17.64	-2.43	0.00
XLOC_024777	-	0	15.72	-inf	0.00
XLOC_024782	-	1.08	0	inf	0.00
XLOC_024783	-	2.62	0	inf	0.00
XLOC_024818	ptrhd1	53.74	14.37	1.90	0.00
XLOC_024842	cad	35.01	3.74	3.23	0.00
XLOC_024844	unc93a	2.68	8.41	-1.65	0.00
XLOC_024860	cyp7a1	1.20	51.71	-5.43	0.00
XLOC_024886	ENSONIG00000006347	0.77	2.71	-1.82	0.00
XLOC_024890	cahz	25.71	173.15	-2.84	0.00
XLOC_024903	mibp2	238.97	1254.70	-2.39	0.00
XLOC_024923	abhd2a	9.59	2.46	1.96	0.00
XLOC_024935	h211-137i24.10 (1 of ma	23.93	66.33	-1.47	0.00
XLOC_025002	tagln2	7.63	18.59	-1.28	0.00
XLOC_025013	-	1.75	0	inf	0.00
XLOC_025037	rogdi	21.00	4.74	2.15	0.00
XLOC_025081	txndc11	17.42	5.22	1.74	0.00
XLOC_025118	rbp2a	38.34	144.77	-1.92	0.00
XLOC_025119	spsb4a	42.81	10.11	2.08	0.00
XLOC_025171	arfgef2	7.56	2.88	1.39	0.00
XLOC_025178	fkbp1ab	2.34	10.80	-2.21	0.00
XLOC_025189	cox4i2	25.41	194.92	-2.94	0.00
XLOC_025197	oatx	29.07	7.88	1.88	0.00
XLOC_025199	slc23a1	2.57	10.46	-2.03	0.00
XLOC_025208	adamts2	8.18	2.67	1.61	0.00
XLOC_025219	-	0	1.65	-inf	0.00
XLOC_025248	sra1	31.99	12.49	1.36	0.00
XLOC_025254	lcp2a	2.61	10.52	-2.01	0.00
XLOC_025302	slc16a1b	25.64	2.48	3.37	0.00
XLOC_025345	si:ch73-106k19.5	0	3.70	-inf	0.00
XLOC_025384	-	0	5.19	-inf	0.00
XLOC_025440	EGFR (1 of many)	27.13	8.78	1.63	0.00
XLOC_025444	-	0	11.42	-inf	0.00

XLOC_025467	lgals3b	5.35	16.24	-1.60	0.00
XLOC_025490	slirp	18.85	7.00	1.43	0.00
XLOC_025510	-	84.66	12.55	2.75	0.00
XLOC_025518	-	0.87	0	inf	0.00
XLOC_025522	ENSONIG00000003942	414.95	3555.59	-3.10	0.00
XLOC_025525	BNIPL (1 of many)	16.46	3.75	2.13	0.00
XLOC_025547	-	14.22	4.64	1.62	0.00
XLOC_025566	ENSONIG00000010845	5.42	70.26	-3.70	0.00
XLOC_025591	btd (1 of many)	215.43	591.70	-1.46	0.00
XLOC_025600,	ENSONIG00000010885	15.63	274.42	-4.13	0.00
XLOC_025640	-	275.35	45.14	2.61	0.00
XLOC_025648	-	0	6.66	-inf	0.00
XLOC_025649	-	0	2.02	-inf	0.00
XLOC_025663	-	4.15	55.29	-3.74	0.00
XLOC_025685	ssr1	67.64	27.15	1.32	0.00
XLOC_025839	-	42.02	0	inf	0.00
XLOC_025841	prmt5	7.06	2.73	1.37	0.00
XLOC_025882	mmp14b	0.66	3.61	-2.45	0.00
XLOC_025907	nfkb2	12.54	33.00	-1.40	0.00
XLOC_025919	ENSONIG00000007088	0.92	0	inf	0.00
XLOC_025969	-	0	5.94	-inf	0.00
XLOC_026009	-	5.78	0	inf	0.00
XLOC_026040	-	12.41	0.30	5.39	0.00
XLOC_026045	sdf2	12.15	4.80	1.34	0.00
XLOC_026083	SLC39A1	18.28	6.56	1.48	0.00
XLOC_026093	mhc1laa	0.21	3.86	-4.17	0.00
XLOC_026119	ENSONIG00000014773	25.05	111.65	-2.16	0.00
XLOC_026130	arcn1b	59.13	13.14	2.17	0.00
XLOC_026152	abt1	6.40	1.24	2.37	0.00
XLOC_026185	epoa	21.48	6.10	1.82	0.00
XLOC_026245	mkrn2	33.37	10.25	1.70	0.00
XLOC_026249	-	18.41	7.54	1.29	0.00
XLOC_026371	-	37.61	0	inf	0.00
XLOC_026375	si:ch211-149p5.1	6.44	0	inf	0.00
XLOC_026448	-	15.66	3.26	2.26	0.00
XLOC_026465	-	0	8.26	-inf	0.00
XLOC_026500	DGKD	7.80	3.04	1.36	0.00
XLOC_026501	-	23.53	7.69	1.61	0.00
XLOC_026503	masp1	17.19	66.68	-1.96	0.00
XLOC_026540	hck	5.37	15.85	-1.56	0.00
XLOC_026541	tm9sf4	12.94	5.07	1.35	0.00
XLOC_026586	-	3.17	0	inf	0.00
XLOC_026594	-	0	1.40	-inf	0.00
XLOC_026661	-	87.81	10.80	3.02	0.00
XLOC_026669	bco1l	44.13	9.97	2.15	0.00
XLOC_026689	ENSONIG00000018736	1.82	0.13	3.76	0.00
XLOC_026716	-	0	1.63	-inf	0.00
XLOC_026717	ENSONIG00000009414	0.50	4.51	-3.17	0.00

XLOC_026724	ftcd	11.21	92.72	-3.05	0.00
XLOC_026753	blnk	1.29	5.66	-2.14	0.00
XLOC_026826	spns2	2.30	6.16	-1.42	0.00
XLOC_026860	zmynd11	1.66	6.44	-1.96	0.00
XLOC_026861	-	22.34	60.62	-1.44	0.00
XLOC_026867	-	2.96	15.57	-2.40	0.00
XLOC_026901	-	34.27	1.72	4.31	0.00
XLOC_026924	-	0	5.97	-inf	0.00
XLOC_026952	magt1	88.53	26.62	1.73	0.00
XLOC_026980	ENSONIG00000014065	1.12	0	inf	0.00
XLOC_026985	-	54.52	0	inf	0.00
XLOC_027012	-	1.08	0	inf	0.00
XLOC_027018	-	6.25	0	inf	0.00
XLOC_027043	-	1.25	0	inf	0.00
XLOC_027061	grpel1	24.92	9.16	1.44	0.00
XLOC_027063	SLC29A2 (1 of many)	17.63	2.56	2.78	0.00
XLOC_027072	-	4.82	36.05	-2.90	0.00
XLOC_027152	-	0	6.93	-inf	0.00
XLOC_027163	pdia6	2108.88	114.41	4.20	0.00
XLOC_027164	si:dkey-90m5.4	210.54	58.76	1.84	0.00
XLOC_027166	rock2b	4.02	1.30	1.63	0.00
XLOC_027179	paplnb	5.69	27.83	-2.29	0.00
XLOC_027182	alg5	86.05	29.11	1.56	0.00
XLOC_027189	hmgb1a	37.15	139.60	-1.91	0.00
XLOC_027218	rbl2	15.87	6.82	1.22	0.00
XLOC_027253	tbc1d15	51.96	19.86	1.39	0.00
XLOC_027280	pipox	45.39	324.67	-2.84	0.00
XLOC_027331	lss	6.17	33.77	-2.45	0.00
XLOC_027414	-	3.45	0	inf	0.00
XLOC_027435	ENSONIG00000009935	123.62	41.53	1.57	0.00
XLOC_027489	mesdc2	62.16	24.80	1.33	0.00
XLOC_027491	c6ast1	40.07	7.24	2.47	0.00
XLOC_027508	-	0.80	14.24	-4.15	0.00
XLOC_027525	txnl4a	11.34	2.73	2.06	0.00
XLOC_027530	-	16.37	3.11	2.40	0.00
XLOC_027534	ENSONIG00000015439	0.23	1.83	-3.00	0.00
XLOC_027546	mgat1b	3.77	0.74	2.35	0.00
XLOC_027552	-	0	1.65	-inf	0.00
XLOC_027576	-	0	4.40	-inf	0.00
XLOC_027577	-	0	2.44	-inf	0.00
XLOC_027606	-	0	39.63	-inf	0.00
XLOC_027625	slc25a11	114.03	48.85	1.22	0.00
XLOC_027660	dmgdh	19.56	63.56	-1.70	0.00
XLOC_027671	ENSONIG00000007379	92.16	531.56	-2.53	0.00
XLOC_027721	chchd2	165.87	41.83	1.99	0.00
XLOC_027786	mmp13a (1 of many)	9.25	2.23	2.05	0.00
XLOC_027821	-	0	3.20	-inf	0.00
XLOC_027846	ENSONIG00000000342	6.02	49.61	-3.04	0.00

XLOC_027848	-	0	1.85	-inf	0.00
XLOC_027856	-	3.78	24.07	-2.67	0.00
XLOC_027875	-	0	2.94	-inf	0.00
XLOC_027914	si:dkey-33c9.6	0.57	3.41	-2.58	0.00
XLOC_027915	ponzr1 (1 of many)	3.19	21.56	-2.76	0.00
XLOC_027943	-	284.28	10.50	4.76	0.00
XLOC_027953	ATXN2L (1 of many)	11.85	3.71	1.67	0.00
XLOC_027992	zgc:92590	20.32	159.43	-2.97	0.00
XLOC_027998	tomm40	19.91	7.48	1.41	0.00
XLOC_028070	-	11.91	165.34	-3.79	0.00
XLOC_028101	-	115.82	35.89	1.69	0.00
XLOC_028107	-	0	6.77	-inf	0.00
XLOC_028255	lamc1	24.45	112.84	-2.21	0.00
XLOC_028287	slc25a24	3.39	17.36	-2.36	0.00
XLOC_028291	-	12.79	2.35	2.44	0.00
XLOC_028310	SLC37A4 (1 of many)	1.32	6.87	-2.38	0.00
XLOC_028334	ENSONIG00000011941	13.98	4.70	1.57	0.00
XLOC_028358	sdf2l1	222.83	42.68	2.38	0.00
XLOC_028382	ENSONIG00000010955	4.63	124.36	-4.75	0.00
XLOC_028384	ENSONIG00000010953	5.24	60.17	-3.52	0.00
XLOC_028389	-	0	119.01	-inf	0.00
XLOC_028403	-	1.21	24.61	-4.35	0.00
XLOC_028413	chia.1 (1 of many)	4.29	0	inf	0.00
XLOC_028441	-	6.67	0	inf	0.00
XLOC_028514	epdr1	14.92	63.56	-2.09	0.00
XLOC_028538	nfxl1	5.69	1.40	2.02	0.00
XLOC_028555	-	31.23	167.65	-2.42	0.00
XLOC_028578	ENSONIG00000009197	4.52	31.38	-2.80	0.00
XLOC_028590	-	1.63	16.37	-3.32	0.00
XLOC_028645	cnpy1	336.22	56.66	2.61	0.00
XLOC_028738	dhrs13b	1.30	18.04	-3.80	0.00
XLOC_028764	-	15.85	4.89	1.70	0.00
XLOC_028767	-	159.78	0	inf	0.00
XLOC_028830	ENSONIG00000019943	0	6.14	-inf	0.00
XLOC_028832	ENSONIG00000019932	0.92	30.84	-5.07	0.00
XLOC_028834	-	597.77	46.21	3.69	0.00
XLOC_028838	ENSONIG00000019937	1.71	9.14	-2.42	0.00
XLOC_028868	eif4e2rs1	33.95	11.96	1.51	0.00
XLOC_028889	-	0	2.96	-inf	0.00
XLOC_028938	-	0	35.20	-inf	0.00
XLOC_028952	-	0	11.28	-inf	0.00
XLOC_028967	-	66.54	6.14	3.44	0.00
XLOC_028971	-	0	4.44	-inf	0.00
XLOC_028974	-	0	1.45	-inf	0.00
XLOC_029009	-	0	33.86	-inf	0.00
XLOC_029012	-	0	1.52	-inf	0.00
XLOC_029109	abat	18.18	59.59	-1.71	0.00
XLOC_029131	ENSONIG00000007518	0.14	4.51	-5.01	0.00

XLOC_029215	atad2 (1 of many)	2.30	10.87	-2.24	0.00
XLOC_029222	hspa9	60.25	24.42	1.30	0.00
XLOC_029237	-	1623.69	302.33	2.43	0.00
XLOC_029244	zbtb14	38.26	13.10	1.55	0.00
XLOC_029258	rhbdd1 (1 of many)	66.01	8.15	3.02	0.00
XLOC_029274	ppp5c	12.64	3.30	1.94	0.00
XLOC_029276	ENSONIG00000005899	0.80	6.90	-3.11	0.00
XLOC_029277	ENSONIG00000005902	16.13	107.21	-2.73	0.00
XLOC_029332	-	9.55	0	inf	0.00
XLOC_029338	pck2	619.12	108.03	2.52	0.00
XLOC_029424	fam49ba	2.19	10.08	-2.20	0.00
XLOC_029459	-	0	1.28	-inf	0.00
XLOC_029470	ENSONIG00000004048	7.47	22.26	-1.58	0.00
XLOC_029504	-	2.83	0	inf	0.00
XLOC_029509	-	11.31	0	inf	0.00
XLOC_029513	camk2d1	2.90	8.76	-1.59	0.00
XLOC_029556	ENSONIG00000008413	1.29	8.94	-2.80	0.00
XLOC_029561	tapbp.2	3.49	14.51	-2.06	0.00
XLOC_029654	-	1.06	0	inf	0.00
XLOC_029656	-	4.09	0	inf	0.00
XLOC_029695	-	17.89	1.61	3.47	0.00
XLOC_029700	-	0	1.65	-inf	0.00
XLOC_029726	-	43.48	11.39	1.93	0.00
XLOC_029754	-	0	41.86	-inf	0.00
XLOC_029786	rab1ba	32.88	8.37	1.97	0.00
XLOC_029797	-	1.27	0	inf	0.00
XLOC_029808	ENSONIG00000004507	2.03	7.28	-1.84	0.00
XLOC_029828	-	4.02	26.67	-2.73	0.00
XLOC_029831	-	1.71	0	inf	0.00
XLOC_029851	-	0	1.54	-inf	0.00
XLOC_029891	-	0	2.61	-inf	0.00
XLOC_029944	-	17.57	0	inf	0.00
XLOC_030014	-	0	49.99	-inf	0.00
XLOC_030039	amph	16.99	2.27	2.91	0.00
XLOC_030040	dnajc1	27.39	8.82	1.63	0.00
XLOC_030042	inhbab	36.15	9.16	1.98	0.00
XLOC_030060	polk	5.04	1.45	1.80	0.00
XLOC_030090	-	246.20	0	inf	0.00
XLOC_030095	-	1.77	30.72	-4.11	0.00
XLOC_030123	-	97.31	8.54	3.51	0.00
XLOC_030133	-	155.22	0	inf	0.00
XLOC_030163	-	3.04	0	inf	0.00
XLOC_030202	-	1279.27	443.97	1.53	0.00
XLOC_030204	-	2.50	0	inf	0.00
XLOC_030251	-	1.47	0	inf	0.00
XLOC_030259	-	0	2.78	-inf	0.00
XLOC_030296	-	0	21.91	-inf	0.00
XLOC_030348	nop58	13.11	4.89	1.42	0.00

XLOC_030385	-	0	72.34	-inf	0.00
XLOC_030390	-	5.87	53.92	-3.20	0.00
XLOC_030424	ENSONIG00000003902	6.46	26.97	-2.06	0.00
XLOC_030427	-	21.77	0	inf	0.00
XLOC_030433	-	2.68	0	inf	0.00
XLOC_030443	-	48.27	0	inf	0.00
XLOC_030467	-	9.17	0	inf	0.00
XLOC_030468	-	6.85	0	inf	0.00
XLOC_030471	-	13.96	3.09	2.17	0.00
XLOC_030517	-	0	4.56	-inf	0.00
XLOC_030526	-	0	17.78	-inf	0.00
XLOC_030536	-	0	12.27	-inf	0.00
XLOC_030554	pacsin3	9.63	31.48	-1.71	0.00
XLOC_030582	ANKS6	2.70	0.87	1.63	0.00
XLOC_030586	-	2.87	0	inf	0.00
XLOC_030599	-	29.62	0	inf	0.00
XLOC_030600	-	35.31	2.22	3.99	0.00
XLOC_030611	-	62.81	0	inf	0.00
XLOC_030615	-	1.33	0	inf	0.00
XLOC_030728	minal	8.51	1.97	2.11	0.00
XLOC_030734	-	0	7.33	-inf	0.00
XLOC_030748	-	0	57.93	-inf	0.00
XLOC_030750	ENSONIG00000013907	83.05	16.13	2.36	0.00
XLOC_030796	-	0	4.02	-inf	0.00
XLOC_030807	EML4	23.85	7.81	1.61	0.00
XLOC_030821	-	6.31	0	inf	0.00
XLOC_030837	ENSONIG00000009177	6.97	37.03	-2.41	0.00
XLOC_030867	dkc1	35.83	14.03	1.35	0.00
XLOC_030879	-	83.83	10.54	2.99	0.00
XLOC_030958	-	10.77	0	inf	0.00
XLOC_030964	-	587.43	0	inf	0.00
XLOC_031028	-	3.68	0	inf	0.00
XLOC_031074	ccdc25	5.97	1.57	1.93	0.00
XLOC_031080	-	0	1.50	-inf	0.00
XLOC_031192	-	207.15	57.25	1.86	0.00
XLOC_031238	-	449.48	137.47	1.71	0.00
XLOC_031272	-	15.48	48.31	-1.64	0.00
XLOC_031273	-	10.45	34.86	-1.74	0.00
XLOC_031315	-	2.12	0	inf	0.00
XLOC_031318	ENSONIG00000010124	1.19	14.36	-3.59	0.00
XLOC_031320	-	10.72	0	inf	0.00
XLOC_031322	-	1.18	7.12	-2.59	0.00
XLOC_031334	ENSONIG00000009100	7.94	111.03	-3.81	0.00
XLOC_031359	-	0	66.46	-inf	0.00
XLOC_031373	-	0	2.60	-inf	0.00
XLOC_031396	-	63.95	0	inf	0.00
XLOC_000350	-	0	4.25	-inf	0.00
XLOC_000388	-	8.17	0	inf	0.00

XLOC_001171	prkag2b	0.57	3.10	-2.44	0.00
XLOC_001259	rab11bb (1 of many)	1.24	5.22	-2.08	0.00
XLOC_001336	minpp1a,papss2a	62.26	162.54	-1.38	0.00
XLOC_001755	ENSONIG00000019863	33.09	13.92	1.25	0.00
XLOC_001787	h1f0	12.31	36.82	-1.58	0.00
XLOC_001887	pdap1a	15.00	6.50	1.21	0.00
XLOC_002188	FGD3	0.66	2.72	-2.03	0.00
XLOC_002385	ENSONIG00000016959	3.29	15.83	-2.27	0.00
XLOC_002468	si:dkey-256k13.2	0.30	1.23	-2.04	0.00
XLOC_002848	cyb5r2	148.98	423.21	-1.51	0.00
XLOC_003018	tcp11l1	4.65	1.61	1.53	0.00
XLOC_003150	-	1.04	5.68	-2.45	0.00
XLOC_003323	ftsj3	10.41	4.39	1.25	0.00
XLOC_003460	-	7.38	34.44	-2.22	0.00
XLOC_003470	CYP2J2 (1 of many)	42.94	131.88	-1.62	0.00
XLOC_003721	ENSONIG00000012547	0.34	109.54	-8.34	0.00
XLOC_003895	igf2	16.51	6.55	1.33	0.00
XLOC_003926	-	7.56	2.16	1.81	0.00
XLOC_003967	pdp2	6.91	2.77	1.32	0.00
XLOC_004365	-	641.29	272.32	1.24	0.00
XLOC_004460	yy1b	13.33	33.30	-1.32	0.00
XLOC_004496	ism2b	2.58	0.46	2.48	0.00
XLOC_004628	mcf2la	0.67	2.13	-1.67	0.00
XLOC_004744	echs1	15.57	35.74	-1.20	0.00
XLOC_004814	ENSONIG00000010088	3.10	0.32	3.30	0.00
XLOC_004896	arhgap22	0.40	2.34	-2.55	0.00
XLOC_005067	-	10.56	0	inf	0.00
XLOC_005284	endou	140.51	657.30	-2.23	0.00
XLOC_005312	ctsz	26.24	61.02	-1.22	0.00
XLOC_005463	-	24.34	55.16	-1.18	0.00
XLOC_005528	aasdhppt	6.03	1.25	2.27	0.00
XLOC_005741	gch2	2.05	14.86	-2.86	0.00
XLOC_005749	ptprea	1.74	5.63	-1.70	0.00
XLOC_005863	fmnl1a	2.74	6.89	-1.33	0.00
XLOC_006222	CAPRIN1 (1 of many)	78.38	24.60	1.67	0.00
XLOC_006294	ENSONIG00000005911	7.30	26.32	-1.85	0.00
XLOC_006384	CASKIN2 (1 of many)	5.87	2.53	1.22	0.00
XLOC_006392	-	0.28	5.87	-4.40	0.00
XLOC_006803	-	3.13	0.45	2.81	0.00
XLOC_006913	hltf	1.47	4.07	-1.47	0.00
XLOC_006974	elavl1 (1 of many)	13.85	4.71	1.56	0.00
XLOC_007008	usp40	3.84	1.58	1.28	0.00
XLOC_007046	zgc:175176	0.18	6.95	-5.31	0.00
XLOC_007085	CRIP2	30.51	92.51	-1.60	0.00
XLOC_007171	nfil3-2	1.01	4.39	-2.12	0.00
XLOC_007180	pkn1b	1.55	4.43	-1.51	0.00
XLOC_007210	ddx5	124.92	43.32	1.53	0.00
XLOC_007346	srebf2	4.69	11.81	-1.33	0.00

XLOC_007431	ube2g2	76.61	26.90	1.51	0.00
XLOC_007433	rrp1	5.66	2.04	1.47	0.00
XLOC_007762	ptgdsb.1	24.62	6.05	2.02	0.00
XLOC_008078	ndufs6	124.25	49.75	1.32	0.00
XLOC_008426	mrpl53	97.66	31.80	1.62	0.00
XLOC_008517	ENSONIG00000002494	1.79	15.27	-3.09	0.00
XLOC_008641	inpp5d	2.39	6.93	-1.53	0.00
XLOC_008711	zgc:103559	69.31	28.70	1.27	0.00
XLOC_008812	ggcx	84.16	30.09	1.48	0.00
XLOC_008825	si:ch211-243g18.2	1.08	4.75	-2.14	0.00
XLOC_008901	hs3st1l2	3.66	9.34	-1.35	0.00
XLOC_009331	ela2l	153.75	1251.83	-3.03	0.00
XLOC_009429	alas2	39.40	125.51	-1.67	0.00
XLOC_009522	napepld	3.25	8.49	-1.38	0.00
XLOC_009948	-	6.61	1.36	2.28	0.00
XLOC_010179	-	0.26	10.09	-5.26	0.00
XLOC_010338	fgfr1a	2.49	0.65	1.94	0.00
XLOC_010460	-	1.42	0	inf	0.00
XLOC_010663	-	12.34	30.77	-1.32	0.00
XLOC_011175	-	0.76	4.66	-2.61	0.00
XLOC_011192	-	1.34	0	inf	0.00
XLOC_011769	synj1	1.12	3.00	-1.42	0.00
XLOC_011922	gabrr3a	0.31	7.88	-4.67	0.00
XLOC_012488	TRA2B	23.77	8.50	1.48	0.00
XLOC_012513	uggt2	2.66	1.02	1.38	0.00
XLOC_012797	ENSONIG00000012952	98.76	23.72	2.06	0.00
XLOC_013316	DDX5 (1 of many)	11.90	27.66	-1.22	0.00
XLOC_013522	ENSONIG00000017979	49.10	17.27	1.51	0.00
XLOC_013589	si:dkey-166n8.9	15.37	6.82	1.17	0.00
XLOC_013613	copz1	32.70	11.63	1.49	0.00
XLOC_013657	mapk14a	8.81	3.10	1.51	0.00
XLOC_014053	calr3b	993.32	244.69	2.02	0.00
XLOC_014114	-	10.04	28.28	-1.49	0.00
XLOC_014217	mrpl10	10.11	3.63	1.48	0.00
XLOC_014278	-	0.17	19.41	-6.87	0.00
XLOC_014288	SRCAP	4.07	1.70	1.26	0.00
XLOC_014584	-	40.41	116.11	-1.52	0.00
XLOC_015546	usp38	4.33	1.78	1.28	0.00
XLOC_015766	fbxo25	1.60	4.11	-1.36	0.00
XLOC_016494	-	1.48	15.11	-3.36	0.00
XLOC_016556	sh3d21	1.18	3.96	-1.74	0.00
XLOC_016692	-	0.79	15.82	-4.32	0.00
XLOC_016975	mcamb	1.61	4.09	-1.35	0.00
XLOC_017023	-	249.63	103.34	1.27	0.00
XLOC_017601	ENSONIG00000004649	1.02	0	inf	0.00
XLOC_017918	hip1rb	3.28	1.08	1.61	0.00
XLOC_018117	eif1ad	9.80	3.91	1.32	0.00
XLOC_018229	zgc:77816 (1 of many)	1.49	8.50	-2.51	0.00

XLOC_018270	-	2.85	0	inf	0.00
XLOC_018295	pgam1a	44.57	17.32	1.36	0.00
XLOC_018377	ppa1b	266.90	95.92	1.48	0.00
XLOC_018399	ptn	1.02	13.60	-3.74	0.00
XLOC_018904	pcxb	84.22	33.89	1.31	0.00
XLOC_019090	ddx10	2.88	0.88	1.71	0.00
XLOC_019813	prox2	3.10	0.59	2.40	0.00
XLOC_019902	id3	28.79	67.39	-1.23	0.00
XLOC_020207	gatsl2	3.88	9.33	-1.27	0.00
XLOC_021034	-	8.78	0	inf	0.00
XLOC_021873	-	0	3.55	-inf	0.00
XLOC_021959	-	0	2.05	-inf	0.00
XLOC_022204	si:ch1073-440b2.1	4.84	1.89	1.36	0.00
XLOC_022503	tmed2	119.33	48.36	1.30	0.00
XLOC_023028	endog	15.32	6.15	1.32	0.00
XLOC_023366	-	2.84	8.60	-1.60	0.00
XLOC_023395	-	1.39	0	inf	0.00
XLOC_023595	ENSONIG00000015285	1.29	13.39	-3.37	0.00
XLOC_023694	arpc1a	46.10	20.21	1.19	0.00
XLOC_024276	ARHGAP17 (1 of many)	2.38	6.75	-1.50	0.00
XLOC_024468	-	1.05	10.53	-3.33	0.00
XLOC_026460	-	0	5.74	-inf	0.00
XLOC_026638	-	0	11.42	-inf	0.00
XLOC_026649	gspt1l (1 of many)	48.02	20.79	1.21	0.00
XLOC_026850	pdzd3b	3.70	8.98	-1.28	0.00
XLOC_026882	stard3nl	7.94	20.09	-1.34	0.00
XLOC_027151	kcnf1a	3.54	0.55	2.69	0.00
XLOC_027174	prep	2.67	0.36	2.88	0.00
XLOC_027596	-	15.40	0	inf	0.00
XLOC_027824	igfbp7	7.00	26.36	-1.91	0.00
XLOC_028314	dgat2	44.34	131.83	-1.57	0.00
XLOC_028365	zgc:64051	16.37	37.44	-1.19	0.00
XLOC_028651	ACOT11 (1 of many)	3.50	9.26	-1.40	0.00
XLOC_029013	-	0	5.02	-inf	0.00
XLOC_029074	mtdhb	97.26	31.28	1.64	0.00
XLOC_029108	rcn3	193.83	61.14	1.66	0.00
XLOC_029350	psme1	52.78	127.47	-1.27	0.00
XLOC_029503	-	1.98	0	inf	0.00
XLOC_029525	cdc42bpb	6.46	2.53	1.35	0.00
XLOC_030091	-	0	3.82	-inf	0.00
XLOC_030094	-	1.85	21.01	-3.50	0.00
XLOC_030096	-	0	1.64	-inf	0.00
XLOC_030112	-	6.63	0	inf	0.00
XLOC_030187	gemin4	4.03	1.60	1.33	0.00
XLOC_030395	-	192.46	1013.88	-2.40	0.00
XLOC_030507	ENSONIG00000016974	3.99	0.74	2.43	0.00
XLOC_031298	-	1.69	0	inf	0.00
XLOC_031340	-	3.66	30.34	-3.05	0.00

XLOC_000040	-	80.70	30.71	1.39	0.00
XLOC_001188	elovl1a	8.79	0.33	4.74	0.00
XLOC_001501	edrf1	3.31	1.21	1.45	0.00
XLOC_002328	-	17.48	7.87	1.15	0.00
XLOC_002599	ABI1 (1 of many)	7.46	16.86	-1.18	0.00
XLOC_002625	ENSONIG00000006617	3.08	9.21	-1.58	0.00
XLOC_002963	MLKL (1 of many)	0.55	2.83	-2.37	0.00
XLOC_003098	zgc:92791	13.42	33.64	-1.33	0.00
XLOC_003115	timm44	7.83	2.75	1.51	0.00
XLOC_003439	fstl3	2.88	0.55	2.39	0.00
XLOC_003497	VAMP4	13.01	5.54	1.23	0.00
XLOC_003579	ndor1	4.94	1.38	1.84	0.00
XLOC_004374	nfkbiaa	26.32	103.55	-1.98	0.00
XLOC_004916	elovl5	43.15	112.89	-1.39	0.00
XLOC_005014	sorbs1	11.37	4.97	1.19	0.00
XLOC_005330	srgap2	6.98	2.98	1.23	0.00
XLOC_005580	ssr3	955.20	340.80	1.49	0.00
XLOC_005923	brd4 (1 of many)	4.50	2.02	1.16	0.00
XLOC_006130	rps6ka2	1.75	0.34	2.38	0.00
XLOC_006132	rcn1	6.84	1.37	2.32	0.00
XLOC_006668	srsf6b	23.76	9.65	1.30	0.00
XLOC_007002	uggt1	17.79	7.38	1.27	0.00
XLOC_007155	BDKRB2 (1 of many)	2.27	6.99	-1.62	0.00
XLOC_007370	polr3h	12.50	3.50	1.84	0.00
XLOC_007418	-	2.48	7.36	-1.57	0.00
XLOC_007525	umps (1 of many)	9.17	3.75	1.29	0.00
XLOC_007681	hopx	2.91	9.78	-1.75	0.00
XLOC_007872	tmem56a	0.41	1.74	-2.07	0.00
XLOC_008040	paqr7a	5.43	0.58	3.22	0.00
XLOC_008342	pparab	6.96	2.31	1.59	0.00
XLOC_008518	ENSONIG00000002495	25.13	61.87	-1.30	0.00
XLOC_008530	-	0	1.19	-inf	0.00
XLOC_008709	hdlbpa	124.82	46.06	1.44	0.00
XLOC_008784	-	0	1.74	-inf	0.00
XLOC_008814	slc20a1b	75.45	24.42	1.63	0.00
XLOC_009296	zgc:136971	0.60	3.07	-2.35	0.00
XLOC_009463	-	0.60	3.78	-2.65	0.00
XLOC_009535	ENSONIG00000011415	3.43	0.69	2.30	0.00
XLOC_009537	tcp11l2	4.69	11.91	-1.35	0.00
XLOC_009554	-	8.70	0	inf	0.00
XLOC_009627	-	0.55	7.36	-3.73	0.00
XLOC_009657	utp20	4.81	1.98	1.28	0.00
XLOC_009833	f9a	12.41	70.62	-2.51	0.00
XLOC_010107	-	2.40	11.12	-2.21	0.00
XLOC_010139	ENSONIG00000019488	173.26	472.87	-1.45	0.00
XLOC_010158	pqlc3	3.10	9.36	-1.60	0.00
XLOC_010429	gas6	5.99	13.60	-1.18	0.00
XLOC_010541	AR (1 of many)	6.87	2.84	1.27	0.00

XLOC_010666	ENSONIG00000017836	5.29	2.10	1.34	0.00
XLOC_010667	nsdhl	5.72	18.42	-1.69	0.00
XLOC_010716	-	10.08	0.30	5.09	0.00
XLOC_011116	thbs1a	0.92	2.49	-1.44	0.00
XLOC_011374	-	1.05	9.45	-3.17	0.00
XLOC_011760	-	21.94	3.42	2.68	0.00
XLOC_011765	-	97.06	43.07	1.17	0.00
XLOC_012117	unc119b	0.53	2.30	-2.11	0.00
XLOC_012136	pllp	10.28	31.54	-1.62	0.00
XLOC_012897	ENSONIG00000000455	0.71	2.87	-2.02	0.00
XLOC_013381	-	5.64	22.04	-1.97	0.00
XLOC_013703	ebp	8.16	21.09	-1.37	0.00
XLOC_013878	ENSONIG00000004173	2.54	15.92	-2.65	0.00
XLOC_014082	-	10.63	4.04	1.39	0.00
XLOC_014135	tia1	12.50	4.79	1.39	0.00
XLOC_014389	slc30a7	54.20	21.63	1.33	0.00
XLOC_014411	-	11.67	4.53	1.37	0.00
XLOC_014412	ENSONIG00000007461	0.16	1.99	-3.64	0.00
XLOC_014436	gcc1	2.70	1.01	1.42	0.00
XLOC_014797	-	20.65	4.28	2.27	0.00
XLOC_014897	mapk13	1.89	5.93	-1.65	0.00
XLOC_015430	hexb	9.79	22.37	-1.19	0.00
XLOC_015810	slc22a16	1.43	5.39	-1.91	0.00
XLOC_016015	zgc:112148	49.34	19.27	1.36	0.00
XLOC_016214	si:dkey-256h2.1	8.09	18.54	-1.20	0.00
XLOC_016506	-	0	11.60	-inf	0.00
XLOC_016537	bcam	1.53	4.01	-1.39	0.00
XLOC_016625	nop2	7.74	2.90	1.42	0.00
XLOC_017001	crot	4.63	13.16	-1.51	0.00
XLOC_017576	tmem259	6.26	2.31	1.44	0.00
XLOC_017828	rnaseh2b	2.56	7.34	-1.52	0.00
XLOC_018063	riok2	5.92	1.77	1.74	0.00
XLOC_018178	ENSONIG00000005280	11.96	31.34	-1.39	0.00
XLOC_018467	hivep3b	1.05	4.38	-2.05	0.00
XLOC_018488	SPAG1	9.15	4.12	1.15	0.00
XLOC_018498	STMN1 (1 of many)	0.40	3.10	-2.96	0.00
XLOC_018678	fbxo41	0.15	1.20	-3.00	0.00
XLOC_018891	-	8.19	2.49	1.72	0.00
XLOC_019130	drd4b	1.81	0.25	2.85	0.00
XLOC_019219	adrb3a	2.27	7.58	-1.74	0.00
XLOC_019228	-	1.80	9.11	-2.34	0.00
XLOC_019786	-	8.35	27.83	-1.74	0.00
XLOC_020725	asna1	26.06	10.61	1.30	0.00
XLOC_021608	sccpdha	23.70	60.68	-1.36	0.00
XLOC_021671	-	7.95	2.83	1.49	0.00
XLOC_022031	ENSONIG00000007870	0.58	2.31	-2.00	0.00
XLOC_022285	ENSONIG00000014952	90.87	487.40	-2.42	0.00
XLOC_022824	ppp1r2	18.63	4.81	1.95	0.00

XLOC_022993	dmtn (1 of many)	1.76	4.94	-1.49	0.00
XLOC_023667	-	2.18	0	inf	0.00
XLOC_024433	-	0	1.53	-inf	0.00
XLOC_024744	-	0	1.62	-inf	0.00
XLOC_024852	msra	15.15	3.17	2.26	0.00
XLOC_026107	CREB3L4	14.47	5.94	1.28	0.00
XLOC_026561	pcmtd2	2.40	5.81	-1.28	0.00
XLOC_026585	-	1.34	0	inf	0.00
XLOC_026810	-	0	1.94	-inf	0.00
XLOC_026946	-	13.91	0	inf	0.00
XLOC_027592	-	83.82	9.43	3.15	0.00
XLOC_027771	dytn	4.88	1.46	1.74	0.00
XLOC_028150	-	6.90	17.42	-1.34	0.00
XLOC_028688	-	26.46	75.44	-1.51	0.00
XLOC_028699	slc6a4a	1.70	4.86	-1.51	0.00
XLOC_029092	ENSONIG00000017501	5.68	30.30	-2.42	0.00
XLOC_029495	-	23.91	0	inf	0.00
XLOC_029558	-	17.66	61.41	-1.80	0.00
XLOC_029635	ENSONIG00000000241	2.76	9.72	-1.81	0.00
XLOC_029998	ssr2	698.67	250.07	1.48	0.00
XLOC_030560	-	4.21	16.74	-1.99	0.00
XLOC_031029	-	7.80	0	inf	0.00
XLOC_031411	-	0	3.64	-inf	0.00
XLOC_031444	-	1.84	31.20	-4.08	0.00
XLOC_000409	-	1.21	0	inf	0.00
XLOC_001025	st3gal3b	5.11	12.71	-1.31	0.00
XLOC_001058	eif4a2	76.05	33.79	1.17	0.00
XLOC_001821	TRPM4	0.09	0.76	-3.04	0.00
XLOC_002189	0000017197,ENSONIG0	0.66	7.15	-3.44	0.00
XLOC_003079	pargl	2.51	8.96	-1.84	0.00
XLOC_003220	hmha1b	6.55	14.45	-1.14	0.00
XLOC_003331	-	11.85	29.12	-1.30	0.00
XLOC_003827	-	12.83	4.15	1.63	0.00
XLOC_003903	ENSONIG00000014563	2.56	0.11	4.50	0.00
XLOC_004043	sema7a	0.50	2.29	-2.20	0.00
XLOC_004614	cnppd1	8.42	3.87	1.12	0.00
XLOC_005041	ATAD3A	6.44	2.33	1.47	0.00
XLOC_005077	pfkfb4b	8.98	3.55	1.34	0.00
XLOC_005089	os9	31.21	13.58	1.20	0.00
XLOC_005271	-	0	4.05	-inf	0.00
XLOC_005332	tmem183a	9.13	3.56	1.36	0.00
XLOC_005351	tmem167b	9.36	3.88	1.27	0.00
XLOC_005859	ezh1	2.28	5.38	-1.24	0.00
XLOC_006737	lyar	9.15	3.02	1.60	0.00
XLOC_007843	mtrr	6.03	2.39	1.33	0.00
XLOC_007974	ENSONIG00000007156	0.85	4.03	-2.24	0.00
XLOC_008155	ano5a	8.11	3.51	1.21	0.00
XLOC_008388	-	1.78	5.18	-1.54	0.00

XLOC_008788	jak2a	7.54	17.52	-1.22	0.00
XLOC_008792	-	39.58	95.55	-1.27	0.00
XLOC_008829	ENSONIG00000003728	0.57	2.55	-2.17	0.00
XLOC_009111	ENSONIG00000005201	3.73	8.99	-1.27	0.00
XLOC_009301	selk	56.66	23.68	1.26	0.00
XLOC_009475	slc16a4	0.54	2.83	-2.40	0.00
XLOC_009934	-	3.02	1.11	1.45	0.00
XLOC_011232	hlx1	6.63	2.33	1.51	0.00
XLOC_011588	ahr2 (1 of many)	5.32	11.90	-1.16	0.00
XLOC_011746	kidins220b	5.66	2.50	1.18	0.00
XLOC_011963	kat5b	15.07	6.68	1.17	0.00
XLOC_012064	fgf1b	10.03	3.34	1.59	0.00
XLOC_012506	cflara	1.74	5.42	-1.64	0.00
XLOC_013048	ENSONIG00000012393	10.04	1.77	2.50	0.00
XLOC_013557	asb8	4.25	11.23	-1.40	0.00
XLOC_013584	b4galnt1a	0.70	2.29	-1.71	0.00
XLOC_014000	prkci	4.19	1.59	1.40	0.00
XLOC_014066	ackr4a	46.97	103.99	-1.15	0.00
XLOC_014345	cradd	18.10	7.61	1.25	0.00
XLOC_015177	-	49.64	12.60	1.98	0.00
XLOC_015461	pik3r1 (1 of many)	207.26	76.21	1.44	0.00
XLOC_015485	tor1 (1 of many)	14.74	5.82	1.34	0.00
XLOC_016055	rnf213a	3.93	12.19	-1.63	0.00
XLOC_016135	-	2.04	8.45	-2.05	0.00
XLOC_016183	rab18a	6.62	2.79	1.25	0.00
XLOC_016282	CTSS (1 of many)	14.92	38.95	-1.38	0.00
XLOC_016491	ENSONIG00000016151	111.13	317.00	-1.51	0.00
XLOC_016638	ENSONIG00000012947	0.93	4.41	-2.25	0.00
XLOC_016811	-	0	16.65	-inf	0.00
XLOC_016822	pus7l	1.82	5.00	-1.46	0.00
XLOC_016863	-	15.86	0	inf	0.00
XLOC_016921	PRKD2	1.00	2.75	-1.45	0.00
XLOC_017060	si:dkey-283b15.4	0.95	2.81	-1.57	0.00
XLOC_017986	dimt1l	8.56	2.38	1.85	0.00
XLOC_018157	mars	15.19	4.40	1.79	0.00
XLOC_019334	-	0	19.76	-inf	0.00
XLOC_020058	rgs13	4.29	12.36	-1.53	0.00
XLOC_020420	hpse	0.97	5.48	-2.50	0.00
XLOC_020928	si:ch211-11k18.4	2.93	0.87	1.75	0.00
XLOC_021722	-	0	3.93	-inf	0.00
XLOC_022118	caspa (1 of many)	0.65	3.14	-2.27	0.00
XLOC_022484	ENSONIG00000011116	6.67	2.87	1.22	0.00
XLOC_022608	sfxn1	27.15	8.91	1.61	0.00
XLOC_022935	casq2	0.10	2.39	-4.64	0.00
XLOC_023101	SNORD14	72.72	12.34	2.56	0.00
XLOC_023195	ENSONIG00000015358	1.18	4.85	-2.04	0.00
XLOC_023251	-	11.75	4.94	1.25	0.00
XLOC_023800	ube2j1	11.85	4.69	1.34	0.00

XLOC_023936	flot2a	6.86	14.59	-1.09	0.00
XLOC_024503	esf1	5.48	2.03	1.44	0.00
XLOC_024766	-	1.29	0	inf	0.00
XLOC_025311	hsd17b10	6.61	16.22	-1.30	0.00
XLOC_025616	SERPINB1 (1 of many)	19.73	7.64	1.37	0.00
XLOC_025656	-	27.13	2.31	3.56	0.00
XLOC_025786	-	17.90	1.50	3.57	0.00
XLOC_026395	MFAP4 (1 of many)	604.16	166.04	1.86	0.00
XLOC_026529	-	3069.38	927.54	1.73	0.00
XLOC_027078	ppp1cbl	95.59	39.10	1.29	0.00
XLOC_028093	ENSONIG00000013997	12.88	321.09	-4.64	0.00
XLOC_028179	-	1.24	0	inf	0.00
XLOC_028774	ENSONIG00000013211	4.37	1.40	1.64	0.00
XLOC_029067	ENSONIG00000013857	0.67	3.29	-2.29	0.00
XLOC_029852	-	0	2.85	-inf	0.00
XLOC_030247	-	3.05	24.33	-3.00	0.00
XLOC_030332	-	609.87	226.47	1.43	0.00
XLOC_031176	-	0	1.37	-inf	0.00
XLOC_031408	-	0	1.49	-inf	0.00
XLOC_000191	-	0	6.07	-inf	0.00
XLOC_000520	aktip	18.31	8.37	1.13	0.00
XLOC_000904	-	2.11	22.08	-3.39	0.00
XLOC_000943	mbnl1	14.47	32.86	-1.18	0.00
XLOC_001399	-	19.97	5.82	1.78	0.00
XLOC_001665	mrpl38	14.88	5.36	1.47	0.00
XLOC_001860	fdxr	4.29	10.79	-1.33	0.00
XLOC_002441	ywhaba	78.89	33.98	1.22	0.00
XLOC_002970	tat	329.43	122.80	1.42	0.00
XLOC_003461	rab11bb (1 of many)	11.08	4.65	1.25	0.00
XLOC_003523	tmem165	13.97	5.20	1.42	0.00
XLOC_004452	PPP2R5C (1 of many)	1.90	4.51	-1.25	0.00
XLOC_004791	mrps6	60.75	25.02	1.28	0.00
XLOC_006315	swap70a	0.28	1.99	-2.82	0.00
XLOC_007391	ENSONIG00000008536	0.57	2.26	-1.99	0.00
XLOC_007545	slc39a10	1.38	3.40	-1.30	0.00
XLOC_007802	-	0.94	4.25	-2.18	0.00
XLOC_008196	metap2a	0.53	2.41	-2.20	0.00
XLOC_008258	clpxa	13.85	29.27	-1.08	0.00
XLOC_008381	elmo1	3.28	10.07	-1.62	0.00
XLOC_008750	nt5c2l1	17.99	7.05	1.35	0.00
XLOC_009024	adamts3	2.09	0.80	1.39	0.00
XLOC_009105	RNF38 (1 of many)	0.73	2.18	-1.58	0.00
XLOC_009435	pigt	13.35	5.86	1.19	0.00
XLOC_009458	slc2a5	3.89	10.90	-1.49	0.00
XLOC_009825	fhl1a	1.07	6.94	-2.69	0.00
XLOC_009854	surf4	105.32	43.34	1.28	0.00
XLOC_010688	-	6.45	2.18	1.56	0.00
XLOC_011010	foxa2	10.96	24.96	-1.19	0.00

XLOC_011625	-	1.42	5.28	-1.89	0.00
XLOC_011882	-	24.01	62.14	-1.37	0.00
XLOC_011883	i:dkey-5g14.1 (1 of many	5.79	16.34	-1.50	0.00
XLOC_012204	ENSONIG00000010213	6.36	16.49	-1.37	0.00
XLOC_012266	ENSONIG00000013498	0.26	2.43	-3.23	0.00
XLOC_012510	dct	2.96	0.11	4.71	0.00
XLOC_013954	pak1ip1	8.75	2.44	1.84	0.00
XLOC_013995	hiat1a	11.22	4.83	1.22	0.00
XLOC_014001	ENSONIG00000018627	2.07	0.48	2.10	0.00
XLOC_014360	zgc:77650	40.79	18.41	1.15	0.00
XLOC_014717	-	40.02	15.76	1.34	0.00
XLOC_015376	-	8.29	0	inf	0.00
XLOC_016166	cdon	1.15	3.03	-1.40	0.00
XLOC_016810	dock4b	0.24	0.97	-2.00	0.00
XLOC_018028	ssh1a	2.70	1.07	1.34	0.00
XLOC_019112	rpf1	13.72	4.73	1.54	0.00
XLOC_019221	antxr1a	0.47	2.81	-2.59	0.00
XLOC_019554	arhgef7a	1.09	3.11	-1.52	0.00
XLOC_019760	prkcha	0.93	3.50	-1.92	0.00
XLOC_020008	-	5.93	17.39	-1.55	0.00
XLOC_022521	hmgcrb	11.75	5.39	1.12	0.00
XLOC_022572	ccng1	90.76	236.62	-1.38	0.00
XLOC_022601	hspa4b	30.77	13.30	1.21	0.00
XLOC_023630	-	4.42	14.25	-1.69	0.00
XLOC_023913	SRC (1 of many)	0.31	1.38	-2.15	0.00
XLOC_024912	tle3b	18.19	8.12	1.16	0.00
XLOC_025298	agap2	0.53	1.95	-1.88	0.00
XLOC_025977	-	2.91	0	inf	0.00
XLOC_026127	ENSONIG00000014771	34.95	16.20	1.11	0.00
XLOC_026171	-	1.68	0	inf	0.00
XLOC_026414	UBL5	153.62	70.98	1.11	0.00
XLOC_026858	-	10.31	4.08	1.34	0.00
XLOC_026862	gtpbp4	198.10	79.73	1.31	0.00
XLOC_027427	-	0	2.16	-inf	0.00
XLOC_027526	znrf2a	7.22	16.10	-1.16	0.00
XLOC_027947	zgc:92040	6.82	16.19	-1.25	0.00
XLOC_028004	cd79a	0.64	7.12	-3.48	0.00
XLOC_028035	-	0	3.51	-inf	0.00
XLOC_028284	tm4sf18	8.80	21.79	-1.31	0.00
XLOC_028496	ENSONIG00000001492	0.48	5.23	-3.44	0.00
XLOC_029160	agla	4.17	9.09	-1.12	0.00
XLOC_029431	ptmaa	257.41	655.75	-1.35	0.00
XLOC_029849	-	1.96	8.88	-2.18	0.00
XLOC_030482	lamb1a	1.02	2.63	-1.37	0.00
XLOC_030527	-	1.32	0	inf	0.00
XLOC_030594	tmem70	17.23	3.78	2.19	0.00
XLOC_031153	-	112.24	17.67	2.67	0.00
XLOC_002152	gpd1a	76.80	33.46	1.20	0.00

XLOC_002667	-	14.99	4.96	1.60	0.00
XLOC_002697	cdv3	9.55	2.65	1.85	0.00
XLOC_004402	ENSONIG00000000431	414.03	146.89	1.49	0.00
XLOC_004701	CASK (1 of many)	1.02	2.93	-1.53	0.00
XLOC_005360	hdac7b	1.29	3.62	-1.49	0.00
XLOC_005494	RASA2	2.43	0.88	1.46	0.00
XLOC_005858	si:ch73-141c7.1	14.88	33.36	-1.17	0.00
XLOC_006786	comtb	60.46	132.94	-1.14	0.00
XLOC_007351	kdelr3	14.10	3.29	2.10	0.00
XLOC_008072	SRFBP1	3.91	1.10	1.83	0.00
XLOC_008548	pag1	2.69	6.65	-1.31	0.00
XLOC_009010	rasgef1ba	4.70	1.83	1.36	0.00
XLOC_011350	praf2	6.77	2.81	1.27	0.00
XLOC_011991	rnf14	19.54	9.12	1.10	0.00
XLOC_013704	ENSONIG00000012291	4.94	13.92	-1.49	0.00
XLOC_014095	arhgap25	1.10	4.38	-1.99	0.00
XLOC_014101	si:ch73-247j11.2	2.80	7.53	-1.43	0.00
XLOC_014419	nup50	7.51	3.21	1.22	0.00
XLOC_014695	ONIG00000012487,tcte	2.79	32.52	-3.54	0.00
XLOC_016078	-	0	2.80	-inf	0.00
XLOC_016633	-	120.65	279.46	-1.21	0.00
XLOC_016826	gsap	0.96	2.57	-1.42	0.00
XLOC_017174	fahd2a	7.01	14.65	-1.06	0.00
XLOC_017823	-	0	2.15	-inf	0.00
XLOC_018340	-	1.38	3.71	-1.43	0.00
XLOC_018407	kdm7ab	3.82	1.34	1.51	0.00
XLOC_018723	mxd4	3.74	13.14	-1.81	0.00
XLOC_019317	-	0	1.54	-inf	0.00
XLOC_020110	col1a1b	2.19	5.35	-1.29	0.00
XLOC_020680	tmc6b	3.00	7.13	-1.25	0.00
XLOC_021020	bms1	3.99	1.65	1.27	0.00
XLOC_022286	NOP14	10.97	4.27	1.36	0.00
XLOC_022556	si:dkeyp-87d8.8	0.63	2.48	-1.98	0.00
XLOC_023217	blk	0.29	1.87	-2.67	0.00
XLOC_023458	cnot7	4.32	1.85	1.22	0.00
XLOC_025946	-	6.03	2.50	1.27	0.00
XLOC_026019	-	19.18	0	inf	0.00
XLOC_026035	ABHD15	0.36	4.98	-3.77	0.00
XLOC_026090	pmvk	11.63	26.63	-1.20	0.00
XLOC_026607	tm9sf1	14.90	6.21	1.26	0.00
XLOC_028149	ENSONIG00000004827	29.35	89.29	-1.61	0.00
XLOC_028356	sh3glb2a	2.59	6.88	-1.41	0.00
XLOC_028461	-	0	1.14	-inf	0.00
XLOC_028829	hmgb2b	2.70	6.50	-1.27	0.00
XLOC_030178	pwp2h	2.53	0.85	1.58	0.00
XLOC_031015	-	0	1.62	-inf	0.00
XLOC_000246	-	3.98	0	inf	0.00
XLOC_000777	pkp3b	28.34	12.29	1.21	0.00

XLOC_001307	zgc:153913	76.91	34.08	1.17	0.00
XLOC_001546	hint1	56.99	121.89	-1.10	0.00
XLOC_001548	lrpprc	7.92	3.37	1.23	0.00
XLOC_001557	PPP4R3CP	20.74	9.39	1.14	0.00
XLOC_002524	rbm33a	1.76	0.69	1.34	0.00
XLOC_002967	cetp	43.62	18.36	1.25	0.00
XLOC_002988	-	72.66	33.46	1.12	0.00
XLOC_004269	sec63,si:ch211-286f9.2	8.17	18.95	-1.21	0.00
XLOC_004773	si:dkey-85a20.4	1.56	0.40	1.97	0.00
XLOC_005127	cdk2	2.93	8.08	-1.47	0.00
XLOC_005432	serpinh1b	17.72	7.47	1.25	0.00
XLOC_006079	olfm2a	5.19	12.59	-1.28	0.00
XLOC_006883	SONIG00000000454,ma	76.76	33.50	1.20	0.00
XLOC_007166	-	0.27	4.66	-4.12	0.00
XLOC_007197	kri1	4.17	1.51	1.46	0.00
XLOC_007540	-	11.32	45.33	-2.00	0.00
XLOC_009311	-	13.17	2.92	2.17	0.00
XLOC_010036	pdxka	7.98	3.20	1.32	0.00
XLOC_010195	-	48.06	22.30	1.11	0.00
XLOC_010513	atic	22.87	65.38	-1.52	0.00
XLOC_010561	bnip1a	23.15	10.82	1.10	0.00
XLOC_011291	ENSONIG00000006326	0.40	2.77	-2.78	0.00
XLOC_011423	-	34.82	0	inf	0.00
XLOC_012198	SONIG00000010203,gna	7.94	1.91	2.06	0.00
XLOC_012740	aadat	1.76	6.65	-1.92	0.00
XLOC_013100	zfyve19	6.71	2.71	1.31	0.00
XLOC_013180	-	39.25	0	inf	0.00
XLOC_014068	-	0	83.24	-inf	0.00
XLOC_014079	kat6a	1.16	2.74	-1.23	0.00
XLOC_014301	cdk5rap3	38.78	17.41	1.16	0.00
XLOC_015804	armt1	2.12	5.85	-1.46	0.00
XLOC_016046	tfap4 (1 of many)	2.62	8.30	-1.66	0.00
XLOC_016187	svila	1.90	4.72	-1.31	0.00
XLOC_016673	atp1a3b	0.76	2.08	-1.45	0.00
XLOC_018567	fam114a1	6.34	2.81	1.18	0.00
XLOC_018672	ENSONIG00000017547	14.63	5.71	1.36	0.00
XLOC_019099	CARTPT (1 of many)	0.95	4.97	-2.38	0.00
XLOC_019103	si:ch73-238c9.1	20.87	8.90	1.23	0.00
XLOC_019238	ela3l	115.10	454.99	-1.98	0.00
XLOC_019942	-	1.25	0	inf	0.00
XLOC_020266	ENSONIG00000020708	1.39	8.01	-2.53	0.00
XLOC_023822	-	0	8.37	-inf	0.00
XLOC_024473	ENSONIG00000011693	17.39	7.51	1.21	0.00
XLOC_024879	blvra	6.50	17.59	-1.44	0.00
XLOC_025511	traf3	2.43	6.35	-1.39	0.00
XLOC_026126	POU2AF1	0.21	2.26	-3.40	0.00
XLOC_026326	-	14.85	0	inf	0.00
XLOC_027193	ugt5d1 (1 of many)	13.17	4.12	1.68	0.00

XLOC_027555	ENSONIG00000004680	9.92	4.29	1.21	0.00
XLOC_027785	-	1.65	0	inf	0.00
XLOC_027859	ncf1	29.86	9.98	1.58	0.00
XLOC_028223	-	0	1.10	-inf	0.00
XLOC_028374	marveld2b	2.97	0.75	1.98	0.00
XLOC_030258	-	0	2.68	-inf	0.00
XLOC_030548	-	0	2.00	-inf	0.00
XLOC_031332	-	8.74	0	inf	0.00
XLOC_000582	nob1	14.19	6.41	1.15	0.00
XLOC_000620	sec11a	169.86	73.74	1.20	0.00
XLOC_001033	gadd45aa	2.11	7.50	-1.83	0.00
XLOC_002378	-	6.54	1.23	2.41	0.00
XLOC_002382	frmd4bb	0.41	1.71	-2.08	0.00
XLOC_003263	prrc2c	4.76	2.17	1.14	0.00
XLOC_004220	-	3.11	12.05	-1.95	0.00
XLOC_005260	PRKCD (1 of many)	0.58	2.45	-2.07	0.00
XLOC_007369	CSDC2	12.36	5.35	1.21	0.00
XLOC_008135	mrpl36	32.99	12.84	1.36	0.00
XLOC_008533	-	23.23	7.52	1.63	0.00
XLOC_008831	crkl	4.04	11.87	-1.56	0.00
XLOC_008836	-	14.69	5.11	1.52	0.00
XLOC_009159	fosl1a	4.68	1.35	1.79	0.00
XLOC_009183	-	51.60	20.05	1.36	0.00
XLOC_010881	CCDC112,pggt1b	3.83	1.61	1.25	0.00
XLOC_011016	-	5.89	14.71	-1.32	0.00
XLOC_012473	CD302	177.01	75.52	1.23	0.00
XLOC_014406	mast2	2.68	1.22	1.13	0.00
XLOC_014601	ENSONIG00000001239	4.84	15.98	-1.72	0.00
XLOC_014893	si:dkey-31b16.7	24.91	9.63	1.37	0.00
XLOC_015175	stim2b	1.90	0.54	1.81	0.00
XLOC_015180	rbpjb	3.03	0.58	2.40	0.00
XLOC_016042	trap1	7.35	3.28	1.17	0.00
XLOC_016075	ccdc137	3.25	0.82	1.98	0.00
XLOC_016280	ENSONIG00000006658	112.08	48.09	1.22	0.00
XLOC_016503	cited4b	1.43	5.87	-2.04	0.00
XLOC_016985	-	1123.76	422.17	1.41	0.00
XLOC_017581	map1sa	6.37	3.02	1.08	0.00
XLOC_017962	gal3st1a,pes	32.20	12.29	1.39	0.00
XLOC_018775	thada	1.56	0.44	1.81	0.00
XLOC_019354	-	26.07	11.01	1.24	0.00
XLOC_019664	naa25	2.87	1.16	1.30	0.00
XLOC_020727	TOM1 (1 of many)	1.87	5.31	-1.50	0.00
XLOC_022596	ENSONIG00000013056	343.31	1134.32	-1.72	0.00
XLOC_022941	ASB9	0.72	3.56	-2.31	0.00
XLOC_023836	-	3.05	0	inf	0.00
XLOC_024111	-	0	2.74	-inf	0.00
XLOC_024403	nagpa	9.69	4.65	1.06	0.00
XLOC_024543	dpy30	94.02	41.17	1.19	0.00

XLOC_026420	ENSONIG00000008983	33.16	85.37	-1.36	0.00
XLOC_026866	-	2.43	13.96	-2.52	0.00
XLOC_027553	-	0	2.19	-inf	0.00
XLOC_027747	si:dkey-95o3.4	2.70	1.05	1.36	0.00
XLOC_027763	-	2.01	0	inf	0.00
XLOC_027929	sult1st6	15.98	40.01	-1.32	0.00
XLOC_029653	-	4.86	0	inf	0.00
XLOC_000408	-	10.60	2.07	2.35	0.00
XLOC_000418	-	7.05	52.54	-2.90	0.00
XLOC_000757	-	2.98	0	inf	0.00
XLOC_000977	-	88.61	41.26	1.10	0.00
XLOC_001488	prkg1a	4.55	1.96	1.21	0.00
XLOC_001879	-	0	2.71	-inf	0.00
XLOC_002257	-	0	1.43	-inf	0.00
XLOC_002505	csrnp1b	1.84	5.50	-1.58	0.00
XLOC_002507	gorasp1b	7.34	2.85	1.36	0.00
XLOC_003783	sh3bp2	0.71	2.09	-1.56	0.00
XLOC_004707	mbnl2	1.99	4.34	-1.13	0.00
XLOC_005612	-	0	14.26	-inf	0.00
XLOC_006006	stat3	53.21	22.80	1.22	0.00
XLOC_006345	gbgt1l2	1.67	5.91	-1.82	0.00
XLOC_006620	ENSONIG00000009728	1.38	4.69	-1.77	0.00
XLOC_007103	-	0	1.38	-inf	0.00
XLOC_007143	lbh	0.61	3.18	-2.38	0.00
XLOC_008093	-	0	5.22	-inf	0.00
XLOC_008809	trim69	0.35	2.44	-2.80	0.00
XLOC_008903	atp8b5a	4.49	9.35	-1.06	0.00
XLOC_009297	sema3b	0.56	1.63	-1.54	0.00
XLOC_009774	-	4.47	15.42	-1.79	0.00
XLOC_009805	ndufa1	69.02	29.90	1.21	0.00
XLOC_009889	sept6	4.72	10.32	-1.13	0.00
XLOC_009996	ENSONIG00000004445	0.66	7.44	-3.49	0.00
XLOC_010178	slc39a8	2.89	6.78	-1.23	0.00
XLOC_010697	-	0	1.67	-inf	0.00
XLOC_010796	ptpn18	3.13	9.17	-1.55	0.00
XLOC_010914	cdc14b	1.36	4.18	-1.63	0.00
XLOC_012670	ENSONIG00000015077	2.17	5.50	-1.34	0.00
XLOC_012841	b4galt7	7.70	2.96	1.38	0.00
XLOC_013090	-	2.77	8.69	-1.65	0.00
XLOC_013204	pik3r5	0.88	2.66	-1.59	0.00
XLOC_013504	si:ch73-71d17.1	30.11	64.94	-1.11	0.00
XLOC_013595	esyt1a	1.99	4.59	-1.21	0.00
XLOC_013634	arhgef3l	4.22	9.75	-1.21	0.00
XLOC_014143	PPP1CC	118.01	46.79	1.33	0.00
XLOC_014328	xpot	2.37	0.71	1.74	0.00
XLOC_014884	ENSONIG00000009203	2.72	5.88	-1.11	0.00
XLOC_015880	gde1	57.42	24.20	1.25	0.00
XLOC_017107	-	22.16	9.20	1.27	0.00

XLOC_017264	ENSONIG00000007433	57.83	149.53	-1.37	0.00
XLOC_017373	zgc:110091	7.70	2.01	1.94	0.00
XLOC_017506	-	24.85	10.98	1.18	0.00
XLOC_017536	LGALS3BP	0.43	3.33	-2.95	0.00
XLOC_017851	p2ry8	1.62	6.18	-1.93	0.00
XLOC_018797	cyr61	7.16	1.69	2.08	0.00
XLOC_019044	AKAP9	9.27	4.26	1.12	0.00
XLOC_019149	arfgap2	17.08	7.75	1.14	0.00
XLOC_021395	prmt3	12.23	5.48	1.16	0.00
XLOC_022173	-	0.76	2.88	-1.93	0.00
XLOC_023122	-	0	7.44	-inf	0.00
XLOC_023383	ENSONIG00000011912	8.91	2.68	1.73	0.00
XLOC_023622	atg13	6.89	15.67	-1.19	0.00
XLOC_023785	mdn1	2.07	0.93	1.16	0.00
XLOC_025107	lpar5b (1 of many)	0.67	7.56	-3.50	0.00
XLOC_025715	-	4.99	14.20	-1.51	0.00
XLOC_025945	-	21.21	4.98	2.09	0.00
XLOC_026195	ENSONIG00000004111	5.41	1.78	1.60	0.00
XLOC_026419	-	207.68	70.06	1.57	0.00
XLOC_028669	ENSONIG00000007800	2.86	6.69	-1.23	0.00
XLOC_028922	-	0.38	1.73	-2.20	0.00
XLOC_029364	-	0	1.46	-inf	0.00
XLOC_029798	vimp	125.20	55.77	1.17	0.00
XLOC_031271	-	1065.72	2368.68	-1.15	0.00
XLOC_031430	-	0	1.13	-inf	0.00
XLOC_000023	ENSONIG00000010957	12.50	4.13	1.60	0.00
XLOC_002995	-	2.96	0	inf	0.00
XLOC_004017	kti12	6.89	1.94	1.83	0.00
XLOC_004307	ehd3	30.22	14.33	1.08	0.00
XLOC_006476	cox6a2	299.09	732.45	-1.29	0.00
XLOC_006596	map2k4a	11.25	33.09	-1.56	0.00
XLOC_007234	-	10.41	2.49	2.06	0.00
XLOC_007247	shmt1	38.98	88.87	-1.19	0.00
XLOC_007617	nipsnap3a	37.86	95.72	-1.34	0.00
XLOC_008574	dgat1a	5.58	2.23	1.32	0.00
XLOC_008847	-	16.77	6.24	1.43	0.00
XLOC_009126	ttc9c	10.06	3.59	1.49	0.00
XLOC_009173	-	4.56	0	inf	0.00
XLOC_009467	gstm.1	4.24	13.49	-1.67	0.00
XLOC_009509	plxna4	0.06	1.06	-4.09	0.00
XLOC_009548	fam3c	54.22	24.56	1.14	0.00
XLOC_009577	copg2	35.90	16.93	1.08	0.00
XLOC_009694	-	3.11	395.28	-6.99	0.00
XLOC_010517	-	1.73	7.50	-2.11	0.00
XLOC_010718	-	8.12	0.96	3.08	0.00
XLOC_010890	mapk4	0.22	1.16	-2.42	0.00
XLOC_011845	zgc:153704 (1 of many)	0	0.77	-inf	0.00
XLOC_013267	ENSONIG00000018079	9.95	22.46	-1.17	0.00

XLOC_013367	pi4k2b	11.83	4.61	1.36	0.00
XLOC_014255	-	16.44	4.14	1.99	0.00
XLOC_016415	-	0	20.17	-inf	0.00
XLOC_017114	tuft1a	13.55	6.10	1.15	0.00
XLOC_017242	dusp2	1.67	4.83	-1.53	0.00
XLOC_017493	si:dkey-199f5.8	5.99	2.67	1.17	0.00
XLOC_018267	ACTR3 (1 of many)	18.46	39.27	-1.09	0.00
XLOC_018470	rcc1	3.72	1.19	1.64	0.00
XLOC_019121	dub	2.41	6.94	-1.53	0.00
XLOC_019351	saraf	30.62	12.56	1.29	0.00
XLOC_019785	-	15.27	47.23	-1.63	0.00
XLOC_020001	uck2b	21.27	8.44	1.33	0.00
XLOC_020270	dcaf13	9.68	4.32	1.17	0.00
XLOC_022720	zgc:152968	0.29	2.33	-3.00	0.00
XLOC_024192	etv5a	3.39	1.28	1.40	0.00
XLOC_024512	-	16.51	7.29	1.18	0.00
XLOC_024517	-	2.62	0.38	2.79	0.00
XLOC_024986	mtmr2	3.45	1.30	1.41	0.00
XLOC_025230	nhp2	66.36	29.00	1.19	0.00
XLOC_025838	ENSONIG00000009132	10.46	31.30	-1.58	0.00
XLOC_025992	-	6.82	0	inf	0.00
XLOC_026961	ccr9a	5.00	11.99	-1.26	0.00
XLOC_027387	alox12	10.96	4.33	1.34	0.00
XLOC_027462	ENSONIG00000018192	4.06	1.19	1.77	0.00
XLOC_027633	-	2.31	57.42	-4.63	0.00
XLOC_027898	tp53	4.13	9.30	-1.17	0.00
XLOC_028068	ENSONIG00000007160	3.88	145.84	-5.23	0.00
XLOC_028584	-	0	35.70	-inf	0.00
XLOC_028685	-	0	6.72	-inf	0.00
XLOC_028873	-	4.87	0	inf	0.00
XLOC_029494	-	12.58	0	inf	0.00
XLOC_031215	-	1279.43	544.92	1.23	0.00
XLOC_000185	ponzr1 (1 of many)	7.72	26.96	-1.80	0.00
XLOC_000676	ENSONIG00000002904	4.34	1.69	1.36	0.00
XLOC_001881	zgc:195081	30.35	7.99	1.93	0.00
XLOC_001938	snu13a	55.28	25.97	1.09	0.00
XLOC_002423	iqsec1b	9.17	3.86	1.25	0.00
XLOC_003021	fa2h	11.60	5.53	1.07	0.00
XLOC_003632	pum3	3.56	1.59	1.16	0.00
XLOC_004819	-	9.90	2.62	1.92	0.00
XLOC_006991	nrd1a	3.08	1.32	1.22	0.00
XLOC_007237	top3a	2.90	1.18	1.30	0.00
XLOC_007378	rangap1a	6.67	2.65	1.33	0.00
XLOC_008835	wu:fc66h01	0.12	2.32	-4.32	0.00
XLOC_009295	ifrd2	7.24	2.86	1.34	0.00
XLOC_010924	-	6.03	0	inf	0.00
XLOC_011277	ppp4cb	37.02	17.56	1.08	0.00
XLOC_011623	filip1l	4.01	1.76	1.19	0.00

XLOC_011770	c10h21orf59	9.36	2.80	1.74	0.00
XLOC_012474	-	282.99	129.10	1.13	0.00
XLOC_013773	-	1175	240.40	2.29	0.00
XLOC_014035	ENSONIG00000018506	6.58	19.70	-1.58	0.00
XLOC_016196	-	0	17.72	-inf	0.00
XLOC_017411	col6a4a	0.28	0.85	-1.60	0.00
XLOC_017820	CTIF	3.33	1.34	1.31	0.00
XLOC_020060	rgs18	3.71	8.41	-1.18	0.00
XLOC_020834	oaz1b	45.12	93.08	-1.04	0.00
XLOC_021349	-	0	7.95	-inf	0.00
XLOC_021480	rbm12b	11.67	5.06	1.20	0.00
XLOC_022350	-	1.37	11.67	-3.09	0.00
XLOC_024271	gaa	1.71	4.74	-1.47	0.00
XLOC_024422	-	1.31	0	inf	0.00
XLOC_026160	zgc:113363	0.59	2.67	-2.18	0.00
XLOC_026333	-	18.91	44.95	-1.25	0.00
XLOC_027864	ENSONIG00000000340	22.71	52.38	-1.21	0.00
XLOC_029329	-	0	2.22	-inf	0.00
XLOC_030306	aoc2	9.62	20.77	-1.11	0.00
XLOC_031008	-	8.39	0	inf	0.00
XLOC_031328	ENSONIG00000010887	1.31	4.06	-1.64	0.00
XLOC_001909	rhbdf1b	0.23	1.38	-2.57	0.00
XLOC_002637	gars (1 of many)	23.51	11.25	1.06	0.00
XLOC_003531	dcun1d4	1.77	5.90	-1.74	0.00
XLOC_003700	-	8.57	3.24	1.41	0.00
XLOC_003982	KCP	6.68	20.38	-1.61	0.00
XLOC_006104	dnajb1a	4.92	0.39	3.66	0.00
XLOC_007246	pdap1b	14.55	6.93	1.07	0.00
XLOC_008034	-	10.88	3.33	1.71	0.00
XLOC_008168	cep152	0.67	7.41	-3.47	0.00
XLOC_009011	-	2.16	7.18	-1.73	0.00
XLOC_009253	cfl1	4.07	10.26	-1.33	0.00
XLOC_009539	si:ch211-244b2.3	0.53	2.21	-2.07	0.00
XLOC_010148	psma6a	10.59	23.60	-1.16	0.00
XLOC_010399	hp	5214.12	887.29	2.55	0.00
XLOC_010615	xiap	23.44	11.18	1.07	0.00
XLOC_011421	-	18.56	0	inf	0.00
XLOC_012081	-	48.02	13.68	1.81	0.00
XLOC_012125	abhd11	3.05	1.02	1.57	0.00
XLOC_014231	psme3	14.12	6.08	1.22	0.00
XLOC_014795	gamt	28.68	64.57	-1.17	0.00
XLOC_014994	-	0	3.50	-inf	0.00
XLOC_015536	plrg1	9.27	4.44	1.06	0.00
XLOC_016203	-	2.05	5.18	-1.34	0.00
XLOC_016357	-	32.78	74.26	-1.18	0.00
XLOC_016698	grwd1	12.43	5.67	1.13	0.00
XLOC_017109	-	13.60	5.67	1.26	0.00
XLOC_017212	osmr	20.77	9.11	1.19	0.00

XLOC_017428	zgc:171704	5.28	0.32	4.06	0.00
XLOC_017573	herc2	2.66	5.87	-1.14	0.00
XLOC_017810	sdad1	14.43	6.76	1.09	0.00
XLOC_017948	ficd	22.64	2.99	2.92	0.00
XLOC_018422	dram1	0.29	2.81	-3.30	0.00
XLOC_020311	-	5.00	0.39	3.68	0.00
XLOC_020698	zgc:56719	26.20	11.63	1.17	0.00
XLOC_024421	-	0	15.06	-inf	0.00
XLOC_026397	VAV1	3.78	8.71	-1.21	0.00
XLOC_026801	psmb12	0.68	4.90	-2.84	0.00
XLOC_026915	PRPS2	76.70	35.88	1.10	0.00
XLOC_027781	ENSONIG00000020591	9.93	1.75	2.51	0.00
XLOC_028663	mfsd12a	1.48	4.05	-1.45	0.00
XLOC_030936	-	8.86	2.04	2.12	0.00
XLOC_000164	-	1225.73	503.04	1.28	0.00
XLOC_000243	ENSONIG00000008330	10.89	3.16	1.78	0.00
XLOC_001571	-	2.72	6.60	-1.28	0.00
XLOC_003192	malt2	0.17	1.84	-3.41	0.00
XLOC_003359	cope	179.47	87.98	1.03	0.00
XLOC_005372	quo	0.11	0.90	-3.06	0.00
XLOC_005383	-	35.17	16.81	1.07	0.00
XLOC_006638	si:ch211-51c14.1	4.37	1.95	1.16	0.00
XLOC_007955	ARMIL1 (1 of many),scg	0.42	3.52	-3.08	0.00
XLOC_010334	stx2b	2.00	0.40	2.33	0.00
XLOC_013081	THBS1 (1 of many)	0.34	1.17	-1.77	0.00
XLOC_015203	-	0	1.82	-inf	0.00
XLOC_015665	-	0	24.13	-inf	0.00
XLOC_017221	vdac3	11.93	25.86	-1.12	0.00
XLOC_018751	mcfd2	180.74	80.66	1.16	0.00
XLOC_019898	ythdf2	7.48	3.35	1.16	0.00
XLOC_020324	-	6536.77	2020.16	1.69	0.00
XLOC_020474	slc31a1	158.40	66.24	1.26	0.00
XLOC_021065	CNNM1 (1 of many)	1.22	3.27	-1.43	0.00
XLOC_021840	wdr55	3.67	1.03	1.84	0.00
XLOC_023266	il17ra1b	5.86	12.22	-1.06	0.00
XLOC_024142	apoob,klhl15	9.53	3.96	1.27	0.00
XLOC_024313	MSRB1 (1 of many)	17.48	6.66	1.39	0.00
XLOC_025168	dpm1	48.00	23.02	1.06	0.00
XLOC_025542	pi4kb (1 of many)	1.73	0.65	1.41	0.00
XLOC_025603	TNS3 (1 of many)	8.03	3.85	1.06	0.00
XLOC_026429	CD247	0.33	3.27	-3.33	0.00
XLOC_026599	-	0	30.63	-inf	0.00
XLOC_027434	-	0	2.05	-inf	0.00
XLOC_028423	ATF6B	4.75	2.12	1.17	0.00
XLOC_028660	cdh6	2.23	4.90	-1.13	0.00
XLOC_028863	-	17.22	5.96	1.53	0.00
XLOC_031182	ENSONIG00000000374	206.66	85.99	1.26	0.00
XLOC_001355	FAM53B (1 of many)	0.70	3.15	-2.18	0.00

XLOC_002442	zgc:193598	1.17	0	inf	0.00
XLOC_004090	ENSONIG00000014587	1.48	3.82	-1.37	0.00
XLOC_004854	sec23ip	19.36	8.53	1.18	0.00
XLOC_004972	sptlc3	1.71	4.63	-1.44	0.00
XLOC_005075	chchd4b	5.32	1.36	1.97	0.00
XLOC_005579	slc33a1	2.93	0.64	2.21	0.00
XLOC_006942	-	20.23	5.53	1.87	0.00
XLOC_007230	-	3.95	13.37	-1.76	0.00
XLOC_007375	xpnpep3	7.53	3.10	1.28	0.00
XLOC_007928	ENSONIG00000007267	3.91	0.55	2.83	0.00
XLOC_008461	SLC45A4 (1 of many)	0.91	2.49	-1.46	0.00
XLOC_009330	-	18.90	48.32	-1.35	0.00
XLOC_009640	-	37.12	17.63	1.07	0.00
XLOC_010762	taz	23.81	11.35	1.07	0.00
XLOC_013545	cct2	67.83	32.19	1.08	0.00
XLOC_013674	idh3g	18.70	7.62	1.30	0.00
XLOC_013910	ENSONIG00000004380	1.15	4.58	-1.99	0.00
XLOC_015507	mmaa	1.24	0.18	2.79	0.00
XLOC_016531	etfb	87.47	42.39	1.05	0.00
XLOC_016599	si:dkeyp-69b9.6	1.91	3.92	-1.04	0.00
XLOC_018191	rbpjl	0.39	2.92	-2.91	0.00
XLOC_018845	cyb5a	100.75	282.54	-1.49	0.00
XLOC_019018	mrps21	168.39	62.91	1.42	0.00
XLOC_019161	-	8.05	2.86	1.49	0.00
XLOC_021069	ENSONIG00000000297	2.84	0.40	2.84	0.00
XLOC_022421	-	1.07	8.77	-3.03	0.00
XLOC_022709	si:ch211-214j8.1	17.68	8.58	1.04	0.00
XLOC_023376	med21	11.66	4.94	1.24	0.00
XLOC_024061	insig2	11.57	5.09	1.18	0.00
XLOC_027560	sacm1la	26.01	11.75	1.15	0.00
XLOC_027962	taco1	6.05	2.19	1.47	0.00
XLOC_028290	pik3ca (1 of many)	1.08	2.43	-1.17	0.00
XLOC_012810	-	72.28	0	inf	0.00
XLOC_001354	METTL10	15.76	5.55	1.50	0.00
XLOC_002535	zgc:112356	8.74	23.37	-1.42	0.00
XLOC_003823	vav2	1.14	3.21	-1.49	0.00
XLOC_004858	-	2.57	13.10	-2.35	0.00
XLOC_005469	npat	2.27	0.88	1.36	0.00
XLOC_006178	-	13.22	4.54	1.54	0.00
XLOC_007291	epor	4.12	8.97	-1.12	0.00
XLOC_008130	marcksl1b	0.30	3.57	-3.56	0.00
XLOC_008288	hbp1	7.17	17.32	-1.27	0.00
XLOC_008924	ptrh1	6.53	2.47	1.40	0.00
XLOC_008969	-	0	1.50	-inf	0.00
XLOC_009393	vapb (1 of many)	20.95	8.92	1.23	0.00
XLOC_009743	myo1f	1.83	3.92	-1.10	0.00
XLOC_010407	-	2.20	5.45	-1.31	0.00
XLOC_010446	-	117.94	0	inf	0.00

XLOC_011551	-	54.25	0	inf	0.00
XLOC_011927	pcyt1bb	0.68	3.05	-2.17	0.00
XLOC_012606	pfkpb	2.36	6.77	-1.52	0.00
XLOC_015747	rab32a	5.90	21.66	-1.88	0.00
XLOC_016421	zfpl1	20.90	10.29	1.02	0.00
XLOC_017000	-	559.54	1415.62	-1.34	0.00
XLOC_017504	-	1.23	0	inf	0.00
XLOC_019868	si:ch211-93g23.2	5.67	14.63	-1.37	0.00
XLOC_020456	-	0.73	6.03	-3.05	0.00
XLOC_022082	p2ry1	2.14	5.00	-1.23	0.00
XLOC_023092	ENSONIG00000014110	38.10	90.50	-1.25	0.00
XLOC_023160	zmynd19	2.93	1.04	1.49	0.00
XLOC_023203	tmed10	315.28	124.63	1.34	0.00
XLOC_023466	ITM2A	29.00	59.97	-1.05	0.00
XLOC_023965	eral1	5.58	12.31	-1.14	0.00
XLOC_024607	rac2	22.14	61.23	-1.47	0.00
XLOC_025234	ENSONIG00000017881	36.01	17.23	1.06	0.00
XLOC_026668	-	12.48	5.48	1.19	0.00
XLOC_027475	-	0	4.30	-inf	0.00
XLOC_028766	-	0	2.40	-inf	0.00
XLOC_030620	-	1.59	0	inf	0.00
XLOC_031011	-	0	8.38	-inf	0.00
XLOC_001289	scinlb	8.61	17.32	-1.01	0.00
XLOC_001923	-	0.81	58.19	-6.16	0.00
XLOC_002186	si:ch73-142c19.1	1.30	3.95	-1.60	0.00
XLOC_002262	gdi1	2.63	1.05	1.32	0.00
XLOC_002283	hipk1a	1.25	0.36	1.78	0.00
XLOC_003272	plpp3	4.53	9.56	-1.08	0.00
XLOC_003391	atf6	15.15	7.34	1.05	0.00
XLOC_005610	polr3e	4.81	2.14	1.17	0.00
XLOC_006107	-	31.02	13.89	1.16	0.00
XLOC_006340	-	3.32	0	inf	0.00
XLOC_006539	-	16.08	6.82	1.24	0.00
XLOC_006808	ndrg3a	5.28	1.94	1.45	0.00
XLOC_007907	AZI2	4.90	10.74	-1.13	0.00
XLOC_008897	sigmar1	18.04	42.52	-1.24	0.00
XLOC_010409	ENSONIG00000020615	17.14	34.70	-1.02	0.00
XLOC_011011	-	7.51	17.46	-1.22	0.00
XLOC_012115	aldh3a2b	31.98	71.09	-1.15	0.00
XLOC_012234	ttc38	23.35	48.53	-1.06	0.00
XLOC_012714	-	53.98	110.02	-1.03	0.00
XLOC_012949	mcm2	0.72	2.41	-1.74	0.00
XLOC_013082	papl	9.19	20.76	-1.18	0.00
XLOC_013571	arhgap4a	1.53	3.55	-1.21	0.00
XLOC_013669	ptges3a	44.85	20.48	1.13	0.00
XLOC_017274	-	0	8.48	-inf	0.00
XLOC_018481	mrs2	5.88	13.78	-1.23	0.00
XLOC_018523	rnf19a (1 of many)	9.96	20.96	-1.07	0.00

XLOC_020011	fzr1b	11.09	26.24	-1.24	0.00
XLOC_020484	-	12.75	30.98	-1.28	0.00
XLOC_021925	ENSONIG00000003653	1.77	0.46	1.94	0.00
XLOC_023538	-	0	11.51	-inf	0.00
XLOC_023931	gusb (1 of many)	6.31	2.45	1.37	0.00
XLOC_025571	-	0	4.04	-inf	0.00
XLOC_026893	cnot1	27.65	11.90	1.22	0.00
XLOC_027874	-	0	5.94	-inf	0.00
XLOC_028859	-	7.28	32.83	-2.17	0.00
XLOC_028925	-	0	5.49	-inf	0.00
XLOC_002827	ENSONIG00000010929	8.88	3.88	1.20	0.00
XLOC_003691	si:dkey-175a17.3	2.75	0.76	1.86	0.00
XLOC_003706	-	2.23	0	inf	0.00
XLOC_004032	-	13.59	2.52	2.43	0.00
XLOC_004418	si:ch73-21k16.1	1.02	2.63	-1.37	0.00
XLOC_004487	agbl5	0.55	1.71	-1.64	0.00
XLOC_005908	-	2.39	8.84	-1.89	0.00
XLOC_006457	-	1.22	0	inf	0.00
XLOC_006831	cidec	53.62	26.34	1.03	0.00
XLOC_008236	-	2.07	0	inf	0.00
XLOC_013074	RASGRP1	1.01	2.75	-1.45	0.00
XLOC_013205	PIK3R6	0.34	1.24	-1.85	0.00
XLOC_013854	rbp7b	1.92	26.82	-3.80	0.00
XLOC_014039	vav3b	0.14	0.99	-2.81	0.00
XLOC_015078	-	0.57	2.62	-2.20	0.00
XLOC_015103	sidt2	40.25	18.95	1.09	0.00
XLOC_015730	-	21.98	10.24	1.10	0.00
XLOC_017220	plat	1.18	3.55	-1.59	0.00
XLOC_019055	SZT2	1.83	4.01	-1.13	0.00
XLOC_019670	ndufaf4	3.05	0.89	1.78	0.00
XLOC_019677	-	0	1.46	-inf	0.00
XLOC_020364	-	4.21	0.90	2.23	0.00
XLOC_023287	-	1.17	0	inf	0.00
XLOC_024289	proza	1.11	2.77	-1.32	0.00
XLOC_024437	TXK	0.11	1.82	-4.01	0.00
XLOC_024653	ttf2	2.09	0.70	1.58	0.00
XLOC_027292	akap1b	1.36	0.24	2.51	0.00
XLOC_027394	-	0	1.20	-inf	0.00
XLOC_027954	ENSONIG00000011489	0.91	3.80	-2.07	0.00
XLOC_028478	-	2.28	0	inf	0.00
XLOC_029442	ENSONIG00000007400	0.52	4.60	-3.13	0.00
XLOC_029474	znf362a	0.32	2.00	-2.63	0.00
XLOC_030107	camk2b2	0.17	0.92	-2.42	0.00
XLOC_031433	-	30.63	4.14	2.89	0.00
XLOC_000099	-	0	38.54	-inf	0.00
XLOC_001006	TIMM44 (1 of many)	1.12	0.17	2.73	0.00
XLOC_002074	dgkaa	0.26	1.64	-2.65	0.00
XLOC_003017	hipk3a	3.05	1.32	1.21	0.00

XLOC_003412	cdc34a	8.29	3.76	1.14	0.00
XLOC_004007	pex11a	2.60	7.28	-1.49	0.00
XLOC_005533	NOX4	2.93	0.24	3.62	0.00
XLOC_005842	hexim1	6.88	15.78	-1.20	0.00
XLOC_006108	prkacaa	5.47	2.27	1.27	0.00
XLOC_006147	atmin	1.51	0.40	1.91	0.00
XLOC_007794	atp8a2	14.20	35.37	-1.32	0.00
XLOC_010945	-	0	46.07	-inf	0.00
XLOC_011910	alg9	13.83	6.17	1.17	0.00
XLOC_012223	ENSONIG00000010321	3.35	1.56	1.10	0.00
XLOC_014268	ENSONIG00000018100	1.05	3.63	-1.78	0.00
XLOC_015896	-	3.16	9.59	-1.60	0.00
XLOC_018396	:ch73-352p4.8 (1 of many	23.40	11.49	1.03	0.00
XLOC_021521	snx10b	3.79	13.64	-1.85	0.00
XLOC_021658	pparg	4.97	10.62	-1.09	0.00
XLOC_022215	eaf2	11.67	5.50	1.09	0.00
XLOC_022802	tmem82	44.29	91.96	-1.05	0.00
XLOC_023786	-	22.58	7.18	1.65	0.00
XLOC_024977	rnf167,srrt	31.90	14.44	1.14	0.00
XLOC_026003	alg14	50.14	21.02	1.25	0.00
XLOC_027531	-	3.89	1.08	1.85	0.00
XLOC_030737	-	0	1.62	-inf	0.00
XLOC_031099	ENSONIG00000014656	1.43	9.90	-2.79	0.00
XLOC_000103	ENSONIG00000007342	2.38	5.71	-1.26	0.00
XLOC_000302	-	45.76	13.01	1.81	0.00
XLOC_000641	SHF	3.79	1.60	1.24	0.00
XLOC_002242	-	10.54	67.20	-2.67	0.00
XLOC_002253	rnf114	7.96	20.50	-1.36	0.00
XLOC_003928	itpr2	0.75	1.75	-1.23	0.00
XLOC_004219	hexa	32.91	15.62	1.08	0.00
XLOC_004807	tasp1	0.95	2.97	-1.64	0.00
XLOC_007400	rpz4,rpz5	24.24	10.15	1.26	0.00
XLOC_007650	egf	0.34	1.19	-1.80	0.00
XLOC_008353	ptdss2	24.81	11.09	1.16	0.00
XLOC_009099	rad9a	1.10	3.87	-1.81	0.00
XLOC_009277	-	15.37	3.27	2.23	0.00
XLOC_009424	-	5.30	13.63	-1.36	0.00
XLOC_010751	-	1.86	0	inf	0.00
XLOC_011550	CTDSP1 (1 of many)	5.61	1.98	1.50	0.00
XLOC_012380	-	0.70	2.46	-1.81	0.00
XLOC_012393	lipea	2.63	6.07	-1.21	0.00
XLOC_014137	upb1	16.27	34.22	-1.07	0.00
XLOC_015582	lrata	6.06	0.82	2.89	0.00
XLOC_016093	frya	4.29	10.00	-1.22	0.00
XLOC_017031	dap3	16.22	7.68	1.08	0.00
XLOC_017302	-	0	17.31	-inf	0.00
XLOC_018466	-	0	1.71	-inf	0.00
XLOC_018657	dnajb14	13.48	5.90	1.19	0.00

XLOC_018744	xpo1a	16.32	7.66	1.09	0.00
XLOC_019407	znf622	11.76	5.69	1.05	0.00
XLOC_022005	-	0	1.48	-inf	0.00
XLOC_025740	fnbp1l	4.91	1.93	1.35	0.00
XLOC_025861	csrnp1a	8.24	17.73	-1.11	0.00
XLOC_025866	col6a2	2.02	4.56	-1.18	0.00
XLOC_027991	cblc	0.26	1.40	-2.44	0.00
XLOC_029677	-	0	3.07	-inf	0.00
XLOC_031243	-	0	8.09	-inf	0.00
XLOC_001194	ppm1la	4.55	1.88	1.28	0.00
XLOC_001713	tomm22	8.48	3.22	1.39	0.00
XLOC_001991	metrn	8.00	2.70	1.57	0.00
XLOC_002240	-	0.70	7.42	-3.41	0.00
XLOC_002896	nadk2	4.34	1.81	1.26	0.00
XLOC_004721	cox15	11.35	4.83	1.23	0.00
XLOC_005715	-	6.06	2.07	1.55	0.00
XLOC_006179	-	14.84	5.19	1.51	0.00
XLOC_006704	ENSONIG00000020302	1.37	3.38	-1.30	0.00
XLOC_006814	-	0	1.49	-inf	0.00
XLOC_009555	-	10.25	0	inf	0.00
XLOC_010447	PRSS23	1.47	4.45	-1.60	0.00
XLOC_010596	-	0.78	3.33	-2.10	0.00
XLOC_010648	cltb	81.19	36.12	1.17	0.00
XLOC_011644	PRKD3 (1 of many)	1.78	4.25	-1.25	0.00
XLOC_012065	-	15.63	32.73	-1.07	0.00
XLOC_014376	-	1.96	4.96	-1.34	0.00
XLOC_016147	trim3a	0.40	1.37	-1.76	0.00
XLOC_018204	R3HDM2	6.90	3.27	1.08	0.00
XLOC_019061	-	0.74	4.46	-2.59	0.00
XLOC_019071	lpar3	2.28	0.70	1.69	0.00
XLOC_022566	-	1.87	11.37	-2.60	0.00
XLOC_023418	pdgfrl	0.54	3.77	-2.81	0.00
XLOC_025247	camk2a	1.40	0.22	2.67	0.00
XLOC_026520	lipt2	4.49	1.26	1.83	0.00
XLOC_026692	eif5b	10.26	5.25	0.97	0.00
XLOC_027484	ENSONIG00000015044	1.28	3.84	-1.59	0.00
XLOC_030738	-	0	1.70	-inf	0.00
XLOC_031405	-	10.84	1.87	2.53	0.00
XLOC_000018	-	24.43	6.56	1.90	0.00
XLOC_000875	-	0	1.80	-inf	0.00
XLOC_001820	ENSONIG00000020073	5.59	1.54	1.86	0.00
XLOC_004184	malt3	9.73	4.67	1.06	0.00
XLOC_004657	-	0.69	3.20	-2.22	0.00
XLOC_005088	arhgef25b	2.62	0.93	1.49	0.00
XLOC_005415	-	2.93	10.05	-1.78	0.00
XLOC_005745	-	0	38.11	-inf	0.00
XLOC_007696	noa1	7.63	3.39	1.17	0.00
XLOC_009142	si:ch73-45o6.2	2.96	5.93	-1.00	0.00

XLOC_009387	-	4.24	1.74	1.29	0.00
XLOC_012988	DLL4 (1 of many)	5.87	2.73	1.10	0.00
XLOC_013187	-	3.57	26.66	-2.90	0.00
XLOC_013290	-	24.59	53.46	-1.12	0.00
XLOC_017024	ENSONIG00000006321	12.74	34.23	-1.43	0.00
XLOC_018921	SNORD22	33.32	13.51	1.30	0.00
XLOC_019312	-	8.70	1.74	2.32	0.00
XLOC_023647	TGDS	5.66	2.64	1.10	0.00
XLOC_025353	-	22.79	55.01	-1.27	0.00
XLOC_025692	-	0	1.99	-inf	0.00
XLOC_025875	-	0	22.36	-inf	0.00
XLOC_027363	arhgap15	2.37	6.79	-1.52	0.00
XLOC_027728	limk1a	1.59	0.56	1.50	0.00
XLOC_029845	-	50.69	11.11	2.19	0.00
XLOC_031321	-	1.36	0	inf	0.00
XLOC_000624	ctsh (1 of many)	0.27	3.02	-3.47	0.00
XLOC_000898	-	0	2.24	-inf	0.00
XLOC_001463	cox20	6.08	1.71	1.83	0.00
XLOC_001641	mrpl27	10.55	4.99	1.08	0.00
XLOC_001861	-	0.84	5.69	-2.76	0.00
XLOC_002558	si:ch211-198h5.3	7.01	3.40	1.05	0.00
XLOC_002752	-	1.03	4.61	-2.16	0.00
XLOC_003309	REPS2	0.42	1.65	-1.97	0.00
XLOC_004241	-	6.96	0.78	3.15	0.00
XLOC_006292	-	0	2.84	-inf	0.00
XLOC_008031	-	0	4.12	-inf	0.00
XLOC_008176	ugt5a1	22.32	10.10	1.14	0.00
XLOC_008378	elp2	2.95	1.17	1.33	0.00
XLOC_008771	-	11.29	1.04	3.44	0.00
XLOC_009684	-	205.72	95.00	1.11	0.00
XLOC_011088	rhd	10.62	40.28	-1.92	0.00
XLOC_011681	allc	11.41	23.39	-1.04	0.00
XLOC_012010	SNX12	51.45	20.51	1.33	0.00
XLOC_014188	-	62.95	30.80	1.03	0.00
XLOC_014507	-	12.82	4.75	1.43	0.00
XLOC_015285	-	3.23	0.52	2.64	0.00
XLOC_016706	si:dkey-17m8.1	1.29	2.96	-1.20	0.00
XLOC_020249	ENSONIG00000000045	10.40	33.33	-1.68	0.00
XLOC_020500	slc25a22	5.02	11.81	-1.23	0.00
XLOC_020776	-	155.28	36.68	2.08	0.00
XLOC_021826	-	8.77	1.24	2.82	0.00
XLOC_022551	sec31a	51.27	22.59	1.18	0.00
XLOC_023108	-	5.17	16.59	-1.68	0.00
XLOC_024447	abce1	49.83	25.05	0.99	0.00
XLOC_025319	:dkey-78k11.9 (1 of man	1.14	6.92	-2.60	0.00
XLOC_025349	-	0	2.69	-inf	0.00
XLOC_030253	ENSONIG00000011731	1.86	0.77	1.27	0.00
XLOC_000291	-	1.46	0	inf	0.00

XLOC_002438	magi1a	3.46	6.80	-0.98	0.00
XLOC_003020	ano10b	0.67	1.67	-1.32	0.00
XLOC_003611	-	4.34	0.96	2.18	0.00
XLOC_003739	lmo4a	2.49	0.94	1.40	0.00
XLOC_004763	znf451	6.07	2.72	1.16	0.00
XLOC_004996	-	0	4.02	-inf	0.00
XLOC_006017	-	10.16	21.52	-1.08	0.00
XLOC_007687	ikbkap	3.49	1.55	1.17	0.00
XLOC_007904	-	25.47	9.63	1.40	0.00
XLOC_008816	drg1	27.94	13.96	1.00	0.00
XLOC_009620	ano6	0.94	2.41	-1.35	0.00
XLOC_009788	smyd5	7.65	3.02	1.34	0.00
XLOC_009899	c1galt1c1	16.82	7.99	1.07	0.00
XLOC_013029	mrps26	6.43	2.26	1.51	0.00
XLOC_014262	tvp23b	40.19	19.82	1.02	0.00
XLOC_014344	-	1.83	5.05	-1.47	0.00
XLOC_014607	wu:fd14a06	0.34	1.66	-2.30	0.00
XLOC_015696	acer3	3.78	7.91	-1.06	0.00
XLOC_016185	bambia	11.15	22.32	-1.00	0.00
XLOC_019123	pcf11	18.09	8.68	1.06	0.00
XLOC_019583	xk	0.79	3.21	-2.03	0.00
XLOC_020248	ENSONIG00000000044	0	5.54	-inf	0.00
XLOC_020992	naprt	2.30	0.53	2.12	0.00
XLOC_021934	-	13.75	0	inf	0.00
XLOC_022626	-	48.45	238.95	-2.30	0.00
XLOC_026011	hccsb	22.97	10.67	1.11	0.00
XLOC_026939	aamp	13.05	5.81	1.17	0.00
XLOC_029826	-	1.44	0	inf	0.00
XLOC_030121	-	3.43	0	inf	0.00
XLOC_002405	rnd1a	12.72	6.18	1.04	0.00
XLOC_002557	smarcd3b	3.81	0.72	2.40	0.00
XLOC_005151	prelp	2.79	1.02	1.45	0.00
XLOC_007552	col5a2a	1.63	3.67	-1.17	0.00
XLOC_010219	rnf34a	3.88	8.47	-1.13	0.00
XLOC_011148	aida	3.30	7.73	-1.23	0.00
XLOC_011865	si:ch211-117m20.5	41.82	115.23	-1.46	0.00
XLOC_012719	-	0.59	2.68	-2.18	0.00
XLOC_013407	clgn	2.92	1.02	1.51	0.00
XLOC_013574	WNK3	0.85	0.26	1.72	0.00
XLOC_016242	nt5c3a	2.42	5.56	-1.20	0.00
XLOC_018230	sec13	69.92	34.79	1.01	0.00
XLOC_020246	foxred1	6.83	2.49	1.46	0.00
XLOC_021445	EPB41 (1 of many)	1.73	11.13	-2.69	0.00
XLOC_022119	-	2.95	14.23	-2.27	0.00
XLOC_022182	si:dkey-145p14.5	6.44	2.59	1.31	0.00
XLOC_022210	sec22a	3.89	1.20	1.70	0.00
XLOC_026077	npr1b	3.09	1.30	1.26	0.00
XLOC_027561	-	8.62	3.35	1.36	0.00

XLOC_028315	mogat2	0.33	3.21	-3.27	0.00
XLOC_029250	-	9.47	90.36	-3.25	0.00
XLOC_004041	ca12	0.27	2.63	-3.30	0.00
XLOC_004334	ARF6 (1 of many)	26.68	13.47	0.99	0.00
XLOC_005194	stx18	14.97	7.28	1.04	0.00
XLOC_007040	l2hgdh	81.50	35.80	1.19	0.00
XLOC_007243	-	9.45	2.86	1.72	0.00
XLOC_010155	diexf	2.70	0.83	1.70	0.00
XLOC_010202	IGSF9	0.30	0.96	-1.67	0.00
XLOC_012130	bbs2	0.91	2.61	-1.51	0.00
XLOC_012583	tg1ipb,sumo3b (1 of man	12.41	5.70	1.12	0.00
XLOC_014086	snrnp27	13.72	5.79	1.25	0.00
XLOC_015374	itm2bb	172.08	376.40	-1.13	0.00
XLOC_016259	agmo	3.87	9.01	-1.22	0.00
XLOC_017718	ISONIG00000017379,zw	13.49	6.46	1.06	0.00
XLOC_019872	-	6.05	13.74	-1.18	0.00
XLOC_020942	-	2.54	5.47	-1.11	0.00
XLOC_023379	slc38a4	460.83	166.31	1.47	0.00
XLOC_024598	-	1.07	4.72	-2.14	0.00
XLOC_027702	-	0	1.86	-inf	0.00
XLOC_029830	-	8.52	0	inf	0.00
XLOC_000290	-	2.74	0	inf	0.00
XLOC_001327	-	10.37	2.85	1.86	0.00
XLOC_002086	kdm5bb	8.41	4.24	0.99	0.00
XLOC_003966	ENSONIG00000020963	2.10	7.08	-1.76	0.00
XLOC_004369	nrxn3b	0.50	1.47	-1.54	0.00
XLOC_006359	si:ch73-237c6.1	277.66	1026.97	-1.89	0.00
XLOC_008932	-	8.82	2.71	1.70	0.00
XLOC_008937	bmp2k	1.83	0.68	1.43	0.00
XLOC_009033	ctsla	297.30	138.91	1.10	0.00
XLOC_011019	hmgcl	22.88	11.49	0.99	0.00
XLOC_014570	hspa8	1260.19	445.65	1.50	0.00
XLOC_015224	ENSONIG00000019068	2.43	0.67	1.86	0.00
XLOC_016244	bmper	3.34	1.11	1.59	0.00
XLOC_016542	ctps1b	2.58	0.88	1.54	0.00
XLOC_021324	-	72.43	34.63	1.06	0.00
XLOC_021823	-	4.04	0.81	2.32	0.00
XLOC_022152	-	1.91	0	inf	0.00
XLOC_029829	-	0.74	4.74	-2.69	0.00
XLOC_031131	gstr (1 of many)	243.46	525.52	-1.11	0.00
XLOC_002715	anxa13	12.90	25.05	-0.96	0.00
XLOC_003469	-	4.34	17.68	-2.03	0.00
XLOC_005459	sgcg	0.17	1.80	-3.44	0.00
XLOC_007301	farsa	65.00	27.37	1.25	0.00
XLOC_007386	rnf216	4.18	8.46	-1.02	0.00
XLOC_007803	flt1	6.05	2.64	1.20	0.00
XLOC_008914	GRAMD3 (1 of many)	36.46	87.52	-1.26	0.00
XLOC_010976	fabp1b.1	450.48	1128.53	-1.32	0.00

XLOC_011073	qpct	38.61	78.76	-1.03	0.00
XLOC_011665	adgrg6	0.89	0.25	1.81	0.00
XLOC_011993	ARHGAP26	0.21	0.83	-2.02	0.00
XLOC_013252	keap1b	52.44	25.64	1.03	0.00
XLOC_016724	si:ch1073-90m23.1	0.11	2.78	-4.61	0.00
XLOC_017741	ENSONIG00000007616	5.50	12.59	-1.20	0.00
XLOC_019962	ENSONIG00000020627	1.63	0	inf	0.00
XLOC_021777	rims1b	0.32	1.03	-1.66	0.00
XLOC_023254	nek7	8.43	4.10	1.04	0.00
XLOC_023940	emc6	24.52	8.70	1.49	0.00
XLOC_023966	trpv1	0.52	2.05	-1.98	0.00
XLOC_024324	foxa3	42.51	20.64	1.04	0.00
XLOC_000454	-	26.26	67.67	-1.37	0.00
XLOC_003560	-	0	2.28	-inf	0.00
XLOC_004532	mcm3	0.70	2.41	-1.79	0.00
XLOC_004559	zgc:56576	35.25	17.32	1.03	0.00
XLOC_004775	map3k4	1.27	2.67	-1.07	0.00
XLOC_005040	mrpl20	7.40	2.90	1.35	0.00
XLOC_008333	parvg	1.18	3.98	-1.76	0.00
XLOC_010695	-	0	2.43	-inf	0.00
XLOC_011129	si:ch211-59d15.9	25.25	9.64	1.39	0.00
XLOC_011879	arfip2b	7.30	3.60	1.02	0.00
XLOC_012760	-	0	1.75	-inf	0.00
XLOC_013486	-	0	1.77	-inf	0.00
XLOC_013770	epha2a	3.87	1.80	1.11	0.00
XLOC_017735	si:dkey-27b3.2	25.97	13.04	0.99	0.00
XLOC_017878	bcor (1 of many)	2.53	1.17	1.11	0.00
XLOC_017937	si:ch211-102c2.7	3.91	1.78	1.13	0.00
XLOC_018964	hax1	21.60	10.18	1.09	0.00
XLOC_019580	si:ch211-237c6.4	2.00	0.28	2.84	0.00
XLOC_019604	lrrc58b	19.97	51.46	-1.37	0.00
XLOC_021468	skap2	3.09	7.14	-1.21	0.00
XLOC_023932	znhit3	27.18	56.02	-1.04	0.00
XLOC_024341	zgc:152863	0.32	1.59	-2.30	0.00
XLOC_025387	ENSONIG00000004547	0	1.20	-inf	0.00
XLOC_025802	-	20.17	1.66	3.60	0.00
XLOC_028482	plin2	42.67	116.07	-1.44	0.00
XLOC_030944	-	0	1.35	-inf	0.00
XLOC_000844	abcb8	3.61	1.49	1.28	0.00
XLOC_003465	ENSONIG00000002601	0.21	0.79	-1.94	0.00
XLOC_005061	-	3.12	12.34	-1.98	0.00
XLOC_006584	0000009540,ENSONIG0	97.34	45.78	1.09	0.00
XLOC_007006	sumo3b (1 of many)	46.62	17.16	1.44	0.00
XLOC_007038	cpxm1a	0.56	1.50	-1.42	0.00
XLOC_007450	myo1b	56.02	26.74	1.07	0.00
XLOC_011255	ENSONIG00000006133	295.07	906.71	-1.62	0.00
XLOC_014990	-	0	2.67	-inf	0.00
XLOC_015186	CLDN15 (1 of many)	1.35	4.90	-1.86	0.00

XLOC_015415	TRAF2 (1 of many)	0.18	0.97	-2.44	0.00
XLOC_015791	sesn1	6.00	12.79	-1.09	0.00
XLOC_015893	tmed1b	11.46	4.06	1.50	0.00
XLOC_016777	ifrd1	25.75	53.59	-1.06	0.00
XLOC_017583	-	27.31	10.84	1.33	0.00
XLOC_018232	-	0	2.29	-inf	0.00
XLOC_018683	-	12.19	3.81	1.68	0.00
XLOC_018769	rel	2.58	5.49	-1.09	0.00
XLOC_019928	colec11	11.64	24.11	-1.05	0.00
XLOC_020492	ADORA3 (1 of many)	752.88	2180.71	-1.53	0.00
XLOC_020556	TCP11X2	0.97	3.30	-1.76	0.00
XLOC_023075	-	319.05	815.95	-1.35	0.00
XLOC_023712	foxk1	10.16	5.08	1.00	0.00
XLOC_027935	ENSONIG00000011530	5.16	12.96	-1.33	0.00
XLOC_029348	-	4.37	1.77	1.30	0.00
XLOC_030669	-	0	40.76	-inf	0.00
XLOC_002243	-	4.13	27.88	-2.75	0.00
XLOC_006399	-	68.45	0	inf	0.00
XLOC_006688	trnt1	4.11	1.33	1.63	0.00
XLOC_007482	-	9.48	0	inf	0.00
XLOC_007743	march6 (1 of many)	12.98	6.35	1.03	0.00
XLOC_008470	-	86.02	42.47	1.02	0.00
XLOC_009408	zgc:154075	67.06	32.20	1.06	0.00
XLOC_010064	nfkbiab	14.64	31.03	-1.08	0.00
XLOC_011547	-	8.36	0	inf	0.00
XLOC_011688	ENSONIG00000018840	1.97	6.54	-1.73	0.00
XLOC_012771	-	0	1.40	-inf	0.00
XLOC_013259	-	12.81	6.47	0.99	0.00
XLOC_013843	si:dkey-183j2.10	0.20	4.77	-4.56	0.00
XLOC_014592	zgc:162872	8.02	4.03	0.99	0.00
XLOC_016239	s100a1	35.20	4.06	3.12	0.00
XLOC_016552	yrdc	7.11	1.77	2.00	0.00
XLOC_016723	si:dkey-40m6.14	47.08	100.78	-1.10	0.00
XLOC_019757	tpp1	42.13	90.27	-1.10	0.00
XLOC_020326	-	2.83	0	inf	0.00
XLOC_020652	nomo	6.56	3.34	0.97	0.00
XLOC_022897	pdia3	1470.79	535.48	1.46	0.00
XLOC_023507	pcyt2 (1 of many)	80.44	37.27	1.11	0.00
XLOC_028404	-	0.21	33.26	-7.33	0.00
XLOC_028433	-	0	7.52	-inf	0.00
XLOC_029402	-	11.22	0	inf	0.00
XLOC_029675	tmx2b	14.39	7.30	0.98	0.00
XLOC_029948	-	0	6.67	-inf	0.00
XLOC_031116	-	7.63	0	inf	0.00
XLOC_031152	-	0	3.55	-inf	0.00
XLOC_008221	IRAK4	6.14	13.82	-1.17	0.00
XLOC_009619	pwp1	4.12	1.70	1.28	0.00
XLOC_010137	eif4eb	37.49	13.94	1.43	0.00

XLOC_010259	-	12.40	2.42	2.36	0.00
XLOC_010281	polr3d	2.02	0.40	2.34	0.00
XLOC_010763	atp6ap1a	19.47	9.99	0.96	0.00
XLOC_013312	acox1	16.54	33.81	-1.03	0.00
XLOC_014826	klf15	13.14	28.86	-1.13	0.00
XLOC_015626	-	2.84	0	inf	0.00
XLOC_016225	herpud2	8.35	16.49	-0.98	0.00
XLOC_018779	-	13.08	6.03	1.12	0.00
XLOC_021340	rab33ba	3.12	1.42	1.13	0.00
XLOC_021607	itsn2b	3.52	1.76	1.00	0.00
XLOC_022232	-	0	13.87	-inf	0.00
XLOC_022542	denr	49.09	25.05	0.97	0.00
XLOC_022915	sox6 (1 of many)	0.42	1.64	-1.96	0.00
XLOC_024596	-	4.59	12.60	-1.46	0.00
XLOC_027944	-	16.78	1.64	3.36	0.00
XLOC_028109	si:dkeyp-67a8.2	1.14	4.82	-2.08	0.00
XLOC_028299	stt3a	255.63	117.43	1.12	0.00
XLOC_029681	si:ch211-113e8.3	2.21	0.61	1.85	0.00
XLOC_030127	-	16.92	0	inf	0.00
XLOC_000458	-	8.75	2.88	1.60	0.00
XLOC_001255	copb2	29.41	14.65	1.01	0.00
XLOC_002131	slc16a7	3.52	8.98	-1.35	0.00
XLOC_002626	irf9	9.29	21.90	-1.24	0.00
XLOC_004105	ppfibp1b	14.52	7.41	0.97	0.00
XLOC_004474	-	12.59	4.59	1.46	0.00
XLOC_004716	cdadc1	1.99	0.48	2.04	0.00
XLOC_006450	grap2a (1 of many)	1.90	5.16	-1.44	0.00
XLOC_008405	otud6b	13.38	6.17	1.12	0.00
XLOC_010972	slc35d2	10.89	5.43	1.00	0.00
XLOC_011176	dnmt3aa	0.41	2.15	-2.38	0.00
XLOC_013473	TMEM19	2.50	5.46	-1.13	0.00
XLOC_013685	dhh	0.32	2.72	-3.10	0.00
XLOC_013760	-	23.79	8.30	1.52	0.00
XLOC_022400	-	2.27	25.32	-3.48	0.00
XLOC_025524	fam63a	12.23	6.22	0.98	0.00
XLOC_030356	BMPR2 (1 of many)	19.97	9.71	1.04	0.00
XLOC_002008	rexo4	4.36	1.79	1.28	0.00
XLOC_002009	ptprh	1.86	5.71	-1.62	0.00
XLOC_005417	-	0	17.46	-inf	0.00
XLOC_006303	-	7.82	17.89	-1.19	0.00
XLOC_006481	-	0	11.67	-inf	0.00
XLOC_006577	-	3.60	1.03	1.80	0.00
XLOC_007144	bmp4	0.47	2.25	-2.26	0.00
XLOC_009155	zgc:92907	71.43	34.25	1.06	0.00
XLOC_009900	clic2	20.76	42.28	-1.03	0.00
XLOC_010092	ENSONIG00000019558	7.87	3.70	1.09	0.00
XLOC_010791	bcap31	59.66	29.42	1.02	0.00
XLOC_011195	heca	0.41	2.09	-2.35	0.00

XLOC_012134	tgm2l (1 of many)	95.51	42.77	1.16	0.00
XLOC_013783	-	1.06	3.77	-1.83	0.00
XLOC_014627	snoU2-30	1.77	4.60	-1.38	0.00
XLOC_017499	pycard (1 of many)	3.02	9.38	-1.64	0.00
XLOC_017507	si:ch73-95l15.3	0.95	3.48	-1.87	0.00
XLOC_020051	-	0	29.70	-inf	0.00
XLOC_022599	-	0.59	6.51	-3.45	0.00
XLOC_023893	-	0	4.23	-inf	0.00
XLOC_029065	-	0	6.26	-inf	0.00
XLOC_029231	ENSONIG00000008168	622.15	139.52	2.16	0.00
XLOC_030236	rpa3	12.68	5.14	1.30	0.00
XLOC_000789	-	0	4.76	-inf	0.00
XLOC_001818	prr12a	0.93	2.28	-1.30	0.00
XLOC_004271	ENSONIG00000020743	44.80	9.91	2.18	0.00
XLOC_006009	ENSONIG00000001203	2.56	1.09	1.23	0.00
XLOC_009080	pcdh1g29	17.01	8.48	1.00	0.00
XLOC_009547	btg1	61.89	31.04	1.00	0.00
XLOC_013130	rpia	19.39	8.82	1.14	0.00
XLOC_016188	zeb1b	2.01	0.87	1.20	0.00
XLOC_017703	-	1.40	7.30	-2.38	0.00
XLOC_023018	gstt2	12.78	27.17	-1.09	0.00
XLOC_023191	-	33.13	0.63	5.71	0.00
XLOC_023344	-	11.85	4.78	1.31	0.00
XLOC_028813	-	9.92	22.38	-1.17	0.00
XLOC_028844	ENSONIG00000017556	0	1.57	-inf	0.00
XLOC_030075	lgalsla	3.00	1.09	1.46	0.00
XLOC_002289	tuba2	11.21	5.50	1.03	0.00
XLOC_002820	exoc3l1	4.24	2.10	1.01	0.00
XLOC_005848	ENSONIG00000000924	0.66	1.89	-1.53	0.00
XLOC_006449	-	0	2.27	-inf	0.00
XLOC_009935	-	10.68	3.95	1.43	0.00
XLOC_010533	agtr2	11.09	21.10	-0.93	0.00
XLOC_011697	-	0	14.52	-inf	0.00
XLOC_012829	-	0	29.12	-inf	0.00
XLOC_020092	-	0	1.69	-inf	0.00
XLOC_020104	arhgap27	1.12	2.72	-1.29	0.00
XLOC_024354	-	1.47	5.83	-1.99	0.00
XLOC_027084	-	56.24	28.57	0.98	0.00
XLOC_027591	-	36.15	5.68	2.67	0.00
XLOC_027687	-	3.13	8.79	-1.49	0.00
XLOC_028166	si:ch73-335l21.4	16.34	6.63	1.30	0.00
XLOC_031254	-	0	22.12	-inf	0.00
XLOC_001927	GID4	8.86	4.13	1.10	0.00
XLOC_010123	trip11	10.17	5.27	0.95	0.00
XLOC_010809	rfc2	1.55	4.79	-1.63	0.00
XLOC_015208	-	0.29	2.49	-3.12	0.00
XLOC_017308	-	1.01	6.14	-2.61	0.00
XLOC_017599	-	4.09	0	inf	0.00

XLOC_019442	ADAP2 (1 of many)	0.43	3.95	-3.20	0.00
XLOC_019804	-	1.73	0	inf	0.00
XLOC_024052	-	0	6.23	-inf	0.00
XLOC_024479	-	13.77	0.67	4.36	0.00
XLOC_001513	ENSONIG00000000618	0.46	1.82	-1.97	0.00
XLOC_003360	mydgf	142.87	68.34	1.06	0.00
XLOC_010475	ENSONIG00000003102	0.21	1.42	-2.74	0.00
XLOC_013174	ENSONIG00000018071	5.44	11.26	-1.05	0.00
XLOC_014002	-	10.02	3.54	1.50	0.00
XLOC_014879	-	19.19	7.97	1.27	0.00
XLOC_016838	scamp2l	6.85	3.25	1.07	0.00
XLOC_018354	mat1a	111.10	50.02	1.15	0.00
XLOC_019782	ddx24	3.99	1.03	1.95	0.00
XLOC_025245	-	2.17	0.30	2.84	0.00
XLOC_026010	ptbp2a	11.17	5.56	1.01	0.00
XLOC_026925	ENSONIG00000001006	0.26	4.25	-4.02	0.00
XLOC_031110	-	6.72	1.72	1.97	0.00
XLOC_005640	fam53b	2.74	11.96	-2.13	0.00
XLOC_006776	gxylt2	0.31	1.90	-2.62	0.00
XLOC_008515	ENSONIG00000002492	1.51	7.02	-2.21	0.00
XLOC_008586	-	0	11.59	-inf	0.00
XLOC_010035	agpat3	7.30	14.34	-0.97	0.00
XLOC_010912	gadd45ga	49.06	126.05	-1.36	0.00
XLOC_012232	tmtc2b	0.33	1.16	-1.80	0.00
XLOC_012317	nfatc1	0.29	0.98	-1.77	0.00
XLOC_013555	pfkma	9.20	18.57	-1.01	0.00
XLOC_014758	polr2h	4.49	1.46	1.62	0.00
XLOC_017252	cdo1	801.59	314.37	1.35	0.00
XLOC_018646	g3bp2	32.33	17.00	0.93	0.00
XLOC_023844	-	0	74.67	-inf	0.00
XLOC_000338	-	0	9.26	-inf	0.00
XLOC_002136	-	0.33	4.90	-3.89	0.00
XLOC_002816	ccl35.1	0.45	9.31	-4.38	0.00
XLOC_003549	chd1l	4.14	1.79	1.21	0.00
XLOC_004358	fkbp1b	27.26	59.16	-1.12	0.00
XLOC_004944	-	14.00	47.52	-1.76	0.00
XLOC_005109	ESYT1 (1 of many)	0.81	1.79	-1.15	0.00
XLOC_005806	afap1l2	0.85	1.99	-1.22	0.00
XLOC_008277	nat10	4.59	1.95	1.23	0.00
XLOC_009153	-	7.93	17.85	-1.17	0.00
XLOC_009328	wrap73	1.07	2.65	-1.31	0.00
XLOC_009669	ENSONIG00000009979	0.33	1.27	-1.94	0.00
XLOC_011774	tiam1a	0.18	1.17	-2.67	0.00
XLOC_021735	rnf19b	0.61	1.47	-1.27	0.00
XLOC_022686	arhgef1a	0.38	1.18	-1.64	0.00
XLOC_023277	-	0	5.13	-inf	0.00
XLOC_023478	-	6.36	1.20	2.40	0.00
XLOC_024539	ENSONIG00000006819	126.10	61.30	1.04	0.00

XLOC_024613	cyth4b	2.35	5.47	-1.22	0.00
XLOC_025287	ada	0.89	3.66	-2.04	0.00
XLOC_030809	-	1.87	0	inf	0.00
XLOC_001629	-	2.66	11.10	-2.06	0.00
XLOC_002846	far1	0.66	2.48	-1.91	0.00
XLOC_004319	zgc:172246	676.57	1877.16	-1.47	0.00
XLOC_006749	-	0.45	2.53	-2.50	0.00
XLOC_007512	ENSONIG00000004273	4.63	11.26	-1.28	0.00
XLOC_011931	-	1.45	3.97	-1.45	0.00
XLOC_012683	-	1.34	2.95	-1.14	0.00
XLOC_017238	pcna	5.04	10.32	-1.03	0.00
XLOC_025215	chm	16.11	8.27	0.96	0.00
XLOC_027510	-	0	3.63	-inf	0.00
XLOC_027524	fam49a	0.86	3.89	-2.18	0.00
XLOC_029773	-	0	6.04	-inf	0.00
XLOC_002867	klhdc4	2.92	1.26	1.21	0.00
XLOC_003697	-	3.47	0.90	1.95	0.00
XLOC_004530	-	71.83	0	inf	0.00
XLOC_005791	PPP1R1B (1 of many)	51.64	103.02	-1.00	0.00
XLOC_005799	VAT1 (1 of many)	1.42	5.12	-1.85	0.00
XLOC_008633	slco2a1	2.40	5.23	-1.12	0.00
XLOC_010187	arg2	3.25	9.13	-1.49	0.00
XLOC_010714	-	4.48	0	inf	0.00
XLOC_011576	map3k2	3.26	1.63	1.00	0.00
XLOC_014951	ANO1 (1 of many)	0.34	1.39	-2.05	0.00
XLOC_016158	ndufc2	111.90	54.52	1.04	0.00
XLOC_017080	erp44	44.14	13.05	1.76	0.00
XLOC_018867	-	8.94	19.70	-1.14	0.00
XLOC_024987	cwc15	53.71	23.78	1.18	0.00
XLOC_026204	ENSONIG00000004146	0.16	6.61	-5.41	0.00
XLOC_028203	-	432.75	1040.05	-1.27	0.00
XLOC_002885	aars	25.49	12.64	1.01	0.00
XLOC_004109	plekhg7	4.09	11.14	-1.45	0.00
XLOC_006189	usp10	6.19	3.07	1.01	0.00
XLOC_009156	si:zfos-80g12.1	7.22	3.50	1.04	0.00
XLOC_010643	tnip1	32.22	80.33	-1.32	0.00
XLOC_010872	zgc:136564	21.34	10.82	0.98	0.00
XLOC_011325	-	0.79	5.64	-2.84	0.00
XLOC_015545	gab1	3.73	7.35	-0.98	0.00
XLOC_017163	-	6.41	2.66	1.27	0.00
XLOC_019476	KHSRP (1 of many)	0.74	2.12	-1.52	0.00
XLOC_022638	slc35a4	7.50	2.18	1.78	0.00
XLOC_022727	-	21.39	42.29	-0.98	0.00
XLOC_028897	shcbp1	3.44	7.13	-1.05	0.00
XLOC_029294	-	5.85	0	inf	0.00
XLOC_001052	imp3	8.40	4.10	1.03	0.00
XLOC_001880	-	0	9.99	-inf	0.00
XLOC_009533	ENSONIG00000011390	1.97	9.85	-2.32	0.00

XLOC_014582	-	3.53	25.63	-2.86	0.00
XLOC_014849	-	83.10	33.46	1.31	0.00
XLOC_016361	-	12.63	26.90	-1.09	0.00
XLOC_020274	oxr1b	18.10	36.84	-1.03	0.00
XLOC_024062	-	6.76	14.77	-1.13	0.00
XLOC_027572	-	110.51	9.46	3.55	0.00
XLOC_030199	-	4023.12	2003.43	1.01	0.00
XLOC_001144	mcm4	0.65	1.76	-1.44	0.00
XLOC_004180	IDH2 (1 of many)	2.42	5.75	-1.25	0.00
XLOC_007953	sntb1	1.32	0.40	1.73	0.00
XLOC_013701	mov10b.1	21.98	10.68	1.04	0.00
XLOC_019985	-	3.27	0.36	3.19	0.00
XLOC_025449	actr3b	1.17	2.78	-1.25	0.00
XLOC_025655	-	23.02	5.80	1.99	0.00
XLOC_001104	si:ch73-52e5.2	2.74	0.24	3.54	0.00
XLOC_001292	RPL5 (1 of many)	73.31	34.72	1.08	0.00
XLOC_004800	300000010021,EPHX1 (1	30.64	71.68	-1.23	0.00
XLOC_007286	UBE2E1 (1 of many)	120.87	62.28	0.96	0.00
XLOC_014257	gprc5c	44.32	22.68	0.97	0.00
XLOC_014269	ENSONIG00000018101	1.01	3.55	-1.81	0.00
XLOC_014713	klhl26	1.82	0.34	2.41	0.00
XLOC_015515	-	0	1.86	-inf	0.00
XLOC_017817	dnajc21	5.60	2.41	1.22	0.00
XLOC_019899	-	34.17	13.08	1.39	0.00
XLOC_020080	rgs11	1.35	3.39	-1.33	0.00
XLOC_020353	tsta3	9.92	4.80	1.05	0.00
XLOC_021193	-	0	2.95	-inf	0.00
XLOC_023719	eif3bb	72.57	36.01	1.01	0.00
XLOC_024854	-	0	25.09	-inf	0.00
XLOC_028184	-	0	39.33	-inf	0.00
XLOC_029960	-	0	1.71	-inf	0.00
XLOC_030685	-	12.61	0	inf	0.00
XLOC_001643	slc9a3r1	27.83	14.43	0.95	0.00
XLOC_002190	WNK2 (1 of many)	2.08	6.05	-1.54	0.00
XLOC_002956	fam96a	36.97	13.75	1.43	0.00
XLOC_004131	ENSONIG00000014819	1.92	3.68	-0.94	0.00
XLOC_005900	si:xx-by187g17.1	2064.17	8938.71	-2.11	0.00
XLOC_005954	ddx42	4.80	9.15	-0.93	0.00
XLOC_020444	fer	4.39	2.18	1.01	0.00
XLOC_022211	pdia5	11.97	6.13	0.97	0.00
XLOC_023682	0000003145,ENSONIG0	15.35	36.43	-1.25	0.00
XLOC_023869	-	0.40	2.50	-2.66	0.00
XLOC_024493	wdr17	4.90	1.56	1.65	0.00
XLOC_024674	gnpat	1.56	0.59	1.39	0.00
XLOC_025391	ENSONIG00000004552	0.22	1.14	-2.39	0.00
XLOC_028079	pms1	0.45	1.60	-1.81	0.00
XLOC_029915	-	5.91	1.91	1.63	0.00
XLOC_001574	-	14.17	27.60	-0.96	0.00

XLOC_006170	cgnl1	2.34	4.56	-0.96	0.00
XLOC_009059	mrpl18	24.28	10.53	1.21	0.00
XLOC_011590	-	5.39	30.57	-2.50	0.00
XLOC_011803	aldocb	62.06	30.30	1.03	0.00
XLOC_012770	adpgk	1.61	0.55	1.55	0.00
XLOC_016850	nr1h3	8.86	17.83	-1.01	0.00
XLOC_018210	ENSONIG00000005120	0.25	1.74	-2.83	0.00
XLOC_018356	sfxn3	5.33	1.72	1.63	0.00
XLOC_021441	hcst	4.48	10.64	-1.25	0.00
XLOC_022398	-	1.77	18.18	-3.36	0.00
XLOC_024922	zgc:77041	38.20	20.04	0.93	0.00
XLOC_003437	bsg	379.98	175.45	1.11	0.00
XLOC_008618	-	11.38	25.76	-1.18	0.00
XLOC_010719	-	17.44	2.86	2.61	0.00
XLOC_011980	dpysl3	0.23	0.99	-2.11	0.00
XLOC_014350	cped1	0.16	1.08	-2.71	0.00
XLOC_018091	smad5	8.94	4.55	0.98	0.00
XLOC_020615	slc35f2	2.01	0.34	2.56	0.00
XLOC_021221	spock3	1.79	5.16	-1.53	0.00
XLOC_023046	tti2	7.19	17.09	-1.25	0.00
XLOC_029665	-	0	14.28	-inf	0.00
XLOC_031119	ENSONIG00000007394	0.33	2.49	-2.92	0.00
XLOC_000255	-	2.32	19.35	-3.06	0.00
XLOC_002629	-	23.69	0	inf	0.00
XLOC_004171	pgpep1l	1.21	10.18	-3.07	0.00
XLOC_009557	-	3.08	0	inf	0.00
XLOC_012095	-	38.98	0	inf	0.00
XLOC_012632	cadm3	0.36	1.13	-1.64	0.00
XLOC_013976	dcaf8	4.76	8.86	-0.90	0.00
XLOC_015731	armc2	1.28	3.45	-1.43	0.00
XLOC_018649	-	0	8.10	-inf	0.00
XLOC_019198	gfra2a	0.93	0.23	2.03	0.00
XLOC_020127	timm23a	17.94	8.17	1.14	0.00
XLOC_020413	mfhas1	2.64	5.09	-0.95	0.00
XLOC_020677	pycr1b	40.15	20.21	0.99	0.00
XLOC_022480	zbtb16b	1.96	0.78	1.33	0.00
XLOC_022751	-	1.36	0	inf	0.00
XLOC_024763	-	23.97	4.81	2.32	0.00
XLOC_024792	-	5.79	0	inf	0.00
XLOC_026512	eif2a	44.87	23.34	0.94	0.00
XLOC_029043	-	1.64	6.38	-1.96	0.00
XLOC_031147	-	1.92	0	inf	0.00
XLOC_009781	slc30a9	5.11	2.56	1.00	0.00
XLOC_010009	dnajb2	5.99	2.43	1.30	0.00
XLOC_011866	c2cd2l	0.97	2.38	-1.29	0.00
XLOC_012588	smarcal1	0.91	2.48	-1.45	0.00
XLOC_013154	-	0.34	2.73	-3.01	0.00
XLOC_017462	ENSONIG00000002289	3.26	9.10	-1.48	0.00

XLOC_019158	gyltl1b	0.77	0.27	1.54	0.00
XLOC_021832	-	0	2.91	-inf	0.00
XLOC_027726	-	1.33	5.08	-1.93	0.00
XLOC_028465	-	0	2.75	-inf	0.00
XLOC_028912	-	0	2.76	-inf	0.00
XLOC_029134	-	0	2.54	-inf	0.00
XLOC_030416	-	0	1.75	-inf	0.00
XLOC_000346	-	109.81	268.25	-1.29	0.00
XLOC_001348	bnip3	47.66	133.47	-1.49	0.00
XLOC_004907	erlin1	12.99	24.81	-0.93	0.00
XLOC_005478	ccnl1a	184.08	78.41	1.23	0.00
XLOC_005879	zfand2a	19.01	9.50	1.00	0.00
XLOC_007764	pign	1.38	0.44	1.63	0.00
XLOC_011862	LTBP3	1.42	3.00	-1.08	0.00
XLOC_013839	pck1	90.08	31.59	1.51	0.00
XLOC_014905	tmco4	11.43	3.43	1.74	0.00
XLOC_016780	si:dkey-180p18.9	6.65	16.08	-1.27	0.00
XLOC_019372	dusp28	1.48	0.38	1.95	0.00
XLOC_021400	-	0	33.85	-inf	0.00
XLOC_023009	prdm12b (1 of many)	19.90	8.17	1.28	0.00
XLOC_029035	atf4a	66.92	31.60	1.08	0.00
XLOC_002729	ggh	6.06	12.66	-1.06	0.00
XLOC_005662	ppifb	2.98	5.84	-0.97	0.00
XLOC_010621	itk	0.32	1.31	-2.05	0.00
XLOC_014092	cds2	6.08	2.09	1.54	0.00
XLOC_024060	si:dkey-251i10.3	2.94	1.16	1.34	0.00
XLOC_024206	satb2 (1 of many)	2.51	1.12	1.16	0.00
XLOC_024436	ANP32B (1 of many)	6.16	12.90	-1.07	0.00
XLOC_026081	anp32e (1 of many)	5.12	9.75	-0.93	0.00
XLOC_001509	cog2	5.43	12.07	-1.15	0.00
XLOC_001690	hexdc	29.99	12.79	1.23	0.00
XLOC_002202	ENSONIG00000017246	5.92	16.34	-1.46	0.00
XLOC_004909	ap3m1	5.58	2.71	1.04	0.00
XLOC_005557	srpr	92.02	31.33	1.55	0.00
XLOC_006503	kpnb1	19.14	10.15	0.92	0.00
XLOC_016365	dock11	0.73	1.56	-1.09	0.00
XLOC_017950	dao.2	26.95	51.41	-0.93	0.00
XLOC_020111	ppp1r9ba	0.27	1.18	-2.11	0.00
XLOC_021039	rab3gap1	13.38	7.14	0.91	0.00
XLOC_023664	-	0	3.72	-inf	0.00
XLOC_027245	rpgrip1l	1.74	0.72	1.26	0.00
XLOC_028295	-	87.70	42.85	1.03	0.00
XLOC_030982	-	0	1.45	-inf	0.00
XLOC_009809	urp1	1.32	0	inf	0.00
XLOC_011237	tagapb	3.24	6.44	-0.99	0.00
XLOC_018701	herc3	2.66	5.25	-0.98	0.00
XLOC_020321	rgs5a	18.35	9.47	0.95	0.00
XLOC_022996	-	11.08	4.09	1.44	0.00

XLOC_024750	-	64.42	33.61	0.94	0.00
XLOC_025727	dnajc19	33.32	17.46	0.93	0.00
XLOC_025789	-	1.86	8.12	-2.13	0.00
XLOC_030011	-	0	9.60	-inf	0.00
XLOC_030557	LRRK1	3.38	7.93	-1.23	0.00
XLOC_000754	-	4.50	9.77	-1.12	0.00
XLOC_004112	banp	3.53	1.66	1.09	0.00
XLOC_006601	bsk146	3.00	1.36	1.14	0.00
XLOC_007499	igfbp2b	45.00	88.68	-0.98	0.00
XLOC_008667	ENSONIG00000019188	0.28	1.20	-2.12	0.00
XLOC_014820	krt5	1.56	0.29	2.43	0.00
XLOC_015678	kptn	2.91	6.33	-1.12	0.00
XLOC_019643	lbr	1.64	3.97	-1.28	0.00
XLOC_020811	dgcr14	4.65	2.15	1.11	0.00
XLOC_022278	clec16a	2.47	4.84	-0.97	0.00
XLOC_022881	zgc:113516	91.08	191.08	-1.07	0.00
XLOC_030126	-	11.18	0.58	4.26	0.00
XLOC_001676	rasd1	2.16	0.13	4.02	0.00
XLOC_001807	ENSONIG00000020037	2.80	7.89	-1.50	0.00
XLOC_002294	-	0.79	3.76	-2.26	0.00
XLOC_003358	cers1	1.14	4.02	-1.82	0.00
XLOC_003395	ENSONIG00000002172	18.53	39.52	-1.09	0.00
XLOC_004426	-	27.74	5.28	2.39	0.00
XLOC_005815	smarcd2	2.45	5.41	-1.14	0.00
XLOC_006649	zgc:162613	25.64	13.65	0.91	0.00
XLOC_007691	-	44.92	20.04	1.16	0.00
XLOC_011853	ephb4a	0.66	1.97	-1.58	0.00
XLOC_012855	-	139.36	72.57	0.94	0.00
XLOC_015046	si:dkey-243k1.3	4.01	11.01	-1.46	0.00
XLOC_015409	-	8.96	18.59	-1.05	0.00
XLOC_017951	usp30	4.99	9.73	-0.96	0.00
XLOC_019470	ENSONIG00000002135	3.20	7.19	-1.17	0.00
XLOC_019598	ENSONIG00000014381	0.20	1.12	-2.46	0.00
XLOC_020496	mov10a	7.50	3.91	0.94	0.00
XLOC_027428	tbk1	3.79	10.27	-1.44	0.00
XLOC_028305	zbtb21	1.31	0.47	1.48	0.00
XLOC_002319	-	0.71	6.91	-3.27	0.00
XLOC_010892	elac1	1.95	0.46	2.07	0.00
XLOC_013089	-	0	1.80	-inf	0.00
XLOC_015244	SONIG00000019086,co:	10.52	5.43	0.95	0.00
XLOC_023142	brf1b (1 of many)	7.61	3.32	1.20	0.00
XLOC_000181	-	1.93	8.76	-2.18	0.00
XLOC_000659	nod2	0.31	1.18	-1.94	0.00
XLOC_000937	gpc1b	2.43	0.57	2.10	0.00
XLOC_001977	ube2ib	28.87	15.09	0.94	0.00
XLOC_002276	ENSONIG00000016568	9.94	2.72	1.87	0.00
XLOC_007081	ENSONIG00000000930	0.82	2.38	-1.54	0.00
XLOC_013628	etv7	2.02	4.68	-1.21	0.00

XLOC_015236	-	34.63	0	inf	0.00
XLOC_020007	pde4bb	11.99	6.15	0.96	0.00
XLOC_022898	isg20	6.46	2.55	1.34	0.00
XLOC_024063	dbi	207.03	99.94	1.05	0.00
XLOC_026528	ABCF3	8.24	4.26	0.95	0.00
XLOC_028010	-	0	2.40	-inf	0.00
XLOC_028198	-	11.69	0	inf	0.00
XLOC_030261	-	0	1.44	-inf	0.00
XLOC_030420	-	0	5.03	-inf	0.00
XLOC_002541	smchd1	2.40	13.82	-2.53	0.00
XLOC_003774	-	9.00	2.47	1.87	0.00
XLOC_003874	-	0	10.51	-inf	0.00
XLOC_009304	TKT (1 of many)	7.09	13.77	-0.96	0.00
XLOC_012639	-	7.84	0	inf	0.00
XLOC_012791	faah2b	5.00	9.66	-0.95	0.00
XLOC_014425	-	0	1.64	-inf	0.00
XLOC_021015	fasn	12.52	36.35	-1.54	0.00
XLOC_021070	ENSONIG00000000299	1.44	3.69	-1.36	0.00
XLOC_023999	lyn	4.52	9.25	-1.03	0.00
XLOC_024187	itgb2	7.51	16.46	-1.13	0.00
XLOC_026409	ttyh3b	0.63	2.20	-1.79	0.00
XLOC_026436	-	0	2.84	-inf	0.00
XLOC_027397	-	0	3.38	-inf	0.00
XLOC_028909	-	240.91	13.06	4.21	0.00
XLOC_030887	-	0	24.74	-inf	0.00
XLOC_031276	-	0	1.18	-inf	0.00
XLOC_003050	uap1l1	16.15	8.40	0.94	0.00
XLOC_004140	ENSONIG00000014876	0.21	1.50	-2.87	0.00
XLOC_007232	ENSONIG00000021079	0.45	1.93	-2.10	0.00
XLOC_014235	MFAP4 (1 of many)	25.12	89.12	-1.83	0.00
XLOC_021670	-	17.44	4.72	1.89	0.00
XLOC_026248	-	8.23	3.94	1.06	0.00
XLOC_002563	ENSONIG00000006237	0.90	5.77	-2.69	0.00
XLOC_003247	mmachc	3.51	1.36	1.37	0.00
XLOC_005831	-	21.91	2.27	3.27	0.00
XLOC_008738	slc35e4	1.28	0.16	3.03	0.00
XLOC_008984	slc2a8	4.40	8.65	-0.98	0.00
XLOC_009233	-	9351.12	2224.42	2.07	0.00
XLOC_010798	-	0.39	3.93	-3.33	0.00
XLOC_012302	eya3	1.83	3.73	-1.03	0.00
XLOC_016971	-	1.41	11.62	-3.04	0.00
XLOC_020143	-	1.30	3.88	-1.57	0.00
XLOC_023319	-	2.19	8.58	-1.97	0.00
XLOC_023884	smarcd1	6.75	3.31	1.03	0.00
XLOC_027964	ripk3	0.42	1.40	-1.73	0.00
XLOC_001858	unc13d	0.25	0.98	-1.97	0.00
XLOC_002296	plxna2	0.80	2.53	-1.66	0.00
XLOC_003480	-	81.70	202.48	-1.31	0.00

XLOC_003828	rxraa	2.22	0.57	1.96	0.00
XLOC_006163	cog4	8.13	4.14	0.97	0.00
XLOC_006326	plekha7b	0.53	1.34	-1.34	0.00
XLOC_013758	HYAL2 (1 of many)	4.17	1.42	1.55	0.00
XLOC_020442	-	2.14	7.45	-1.80	0.00
XLOC_004242	-	462.79	211.33	1.13	0.00
XLOC_005736	ppm1bb	12.69	6.46	0.97	0.00
XLOC_010122	golga5	6.76	3.45	0.97	0.00
XLOC_010677	prps1b	10.30	5.25	0.97	0.00
XLOC_016350	RASGRP2	1.43	3.46	-1.27	0.00
XLOC_017404	ENSONIG00000002024	5.72	2.61	1.13	0.00
XLOC_018268	ENSONIG00000007888	96.66	40.78	1.25	0.00
XLOC_023419	-	11.88	4.58	1.37	0.00
XLOC_026981	-	2.61	0	inf	0.00
XLOC_006255	fes	1.05	3.82	-1.86	0.00
XLOC_008094	cited4a	9.64	18.45	-0.94	0.00
XLOC_010680	si:ch211-153b23.5	1.09	3.49	-1.68	0.00
XLOC_011580	GLS (1 of many)	3.07	1.46	1.07	0.00
XLOC_012091	f9b	676.51	296.38	1.19	0.00
XLOC_014096	bmp10	1.56	3.35	-1.10	0.00
XLOC_014663	IL1RAP	6.76	3.58	0.92	0.00
XLOC_023552	-	207.56	762.42	-1.88	0.00
XLOC_024023	dad1	360.72	188.49	0.94	0.00
XLOC_028001	prss59.1	973.22	4642.79	-2.25	0.00
XLOC_029605	trmt10a	1.53	0.55	1.48	0.00
XLOC_003178	si:rp71-15d4.1	0.53	1.70	-1.68	0.00
XLOC_005126	mmp19	2.04	0.50	2.03	0.00
XLOC_010003	prmt2	2.90	1.28	1.18	0.00
XLOC_011249	ttc32	49.12	21.30	1.21	0.00
XLOC_013152	ckba	1.67	15.00	-3.16	0.00
XLOC_016269	ENSONIG00000006599	231.83	43.77	2.41	0.00
XLOC_018264	-	8.15	0	inf	0.00
XLOC_020366	tmem97	56.59	108.55	-0.94	0.00
XLOC_022532	ENSONIG00000015481	9.10	4.73	0.94	0.00
XLOC_028512	ENSONIG00000001462	0.51	2.54	-2.30	0.00
XLOC_001393	atl2	19.13	40.76	-1.09	0.00
XLOC_005752	bccip	19.17	9.56	1.00	0.00
XLOC_006056	ENSONIG00000001489	7673.46	30318.90	-1.98	0.00
XLOC_008787	strbp	0.38	1.46	-1.94	0.00
XLOC_009220	-	1.46	19.03	-3.70	0.00
XLOC_011454	IQSEC1 (1 of many)	1.58	0.70	1.17	0.00
XLOC_013695	cbx5	13.91	26.57	-0.93	0.00
XLOC_014238	LASP1 (1 of many)	0.57	4.37	-2.94	0.00
XLOC_015728	foxo3b	3.03	1.38	1.13	0.00
XLOC_015757	ccdc167	15.08	6.98	1.11	0.00
XLOC_016963	mre11a	6.07	2.97	1.03	0.00
XLOC_021894	cybb	4.59	11.03	-1.27	0.00
XLOC_025586	col1a2	2.20	4.55	-1.04	0.00

XLOC_025704	-	0.34	3.20	-3.25	0.00
XLOC_026691	iqcb1	2.04	0.61	1.74	0.00
XLOC_031236	-	35.64	17.62	1.02	0.00
XLOC_000002	-	28.22	0	inf	0.00
XLOC_008180	cox5aa	79.51	42.11	0.92	0.00
XLOC_009057	prp19	12.22	6.42	0.93	0.00
XLOC_017040	TARS2	13.63	7.12	0.94	0.00
XLOC_020631	LDB1 (1 of many)	8.50	16.45	-0.95	0.00
XLOC_023146	-	3.58	0	inf	0.00
XLOC_025072	dnaja3b	3.62	1.04	1.80	0.00
XLOC_027585	-	3.62	0	inf	0.00
XLOC_027593	-	1.61	0	inf	0.00
XLOC_000251	-	18.65	6.77	1.46	0.00
XLOC_000523	-	0.48	3.14	-2.71	0.00
XLOC_003151	acot11b	3.37	8.88	-1.40	0.00
XLOC_006238	wt1b	1.10	2.55	-1.22	0.00
XLOC_006671	rpn2	74.60	35.94	1.05	0.00
XLOC_007240	st13	58.30	31.26	0.90	0.00
XLOC_007976	rbms3	3.74	0.61	2.63	0.00
XLOC_011672	ENSONIG00000018790	0.16	2.74	-4.07	0.00
XLOC_013869	rbm5	46.23	94.78	-1.04	0.00
XLOC_014949	KLHL25	2.60	1.19	1.12	0.00
XLOC_015167	-	433.99	222.95	0.96	0.00
XLOC_022747	psat1	1.00	3.60	-1.85	0.00
XLOC_023873	rbck1	3.61	7.15	-0.98	0.00
XLOC_027338	adat3,alkbh6	9.96	4.17	1.26	0.00
XLOC_006828	sec61a1	425.07	195.18	1.12	0.00
XLOC_007771	-	0	1.72	-inf	0.00
XLOC_009715	casc1	0.25	2.07	-3.07	0.00
XLOC_011153	enpp1	9.93	18.78	-0.92	0.00
XLOC_011973	ubash3ba	12.66	6.53	0.95	0.00
XLOC_012068	yipf5	28.46	14.91	0.93	0.00
XLOC_014501	col16a1	0.23	0.78	-1.73	0.00
XLOC_018245	COL6A3	2.98	9.58	-1.68	0.00
XLOC_020383	-	0	5.13	-inf	0.00
XLOC_021946	-	5.94	2.73	1.12	0.00
XLOC_023944	TIMM22	3.59	1.17	1.62	0.00
XLOC_025894	ENSONIG00000015663	15.75	50.36	-1.68	0.00
XLOC_026123	-	0	2.52	-inf	0.00
XLOC_029834	-	0	4.70	-inf	0.00
XLOC_001158	irf4a	0.51	1.49	-1.54	0.00
XLOC_008917	nudt12	1.63	4.09	-1.32	0.00
XLOC_010010	si:ch73-199e17.1	3.80	8.37	-1.14	0.00
XLOC_010343	vsig8b	2.96	6.58	-1.15	0.00
XLOC_012828	g3bp1	12.14	6.36	0.93	0.00
XLOC_014299	-	30.84	5.67	2.44	0.00
XLOC_015645	krr1	7.80	3.54	1.14	0.00
XLOC_015806	mthfd1l	0.99	0.22	2.14	0.00

XLOC_015955	C3 (1 of many)	307.24	807.92	-1.39	0.00
XLOC_017511	glrx3	24.97	13.09	0.93	0.00
XLOC_006004	si:ch211-210g13.5	0.22	1.02	-2.24	0.00
XLOC_006372	prf1.3	0.11	1.16	-3.43	0.00
XLOC_007104	ENSONIG00000001094	0.60	14.81	-4.64	0.00
XLOC_007184	txn2	60.23	32.01	0.91	0.00
XLOC_008509	fam91a1	4.11	2.12	0.96	0.00
XLOC_009944	tuba8l2	7.04	14.17	-1.01	0.00
XLOC_012346	RNF5	8.87	3.00	1.56	0.00
XLOC_012822	-	2.51	0.55	2.18	0.00
XLOC_018083	ENSONIG00000020654	4.86	11.07	-1.19	0.00
XLOC_019935	-	2.26	8.22	-1.86	0.00
XLOC_020027	tfr1a	307.94	132.76	1.21	0.00
XLOC_026998	puf60a	11.38	5.88	0.95	0.00
XLOC_027268	-	8.62	0	inf	0.00
XLOC_002529	ube3c	6.85	3.47	0.98	0.00
XLOC_009413	rer1,tp73	13.40	6.17	1.12	0.00
XLOC_011839	ENSONIG00000005110	0.14	0.84	-2.59	0.00
XLOC_015752	sash1a	0.33	1.03	-1.63	0.00
XLOC_016340	rbm4.3 (1 of many)	7.84	4.01	0.97	0.00
XLOC_020716	rgl3a	0.25	1.02	-2.01	0.00
XLOC_024685	-	0.65	1.95	-1.58	0.00
XLOC_028723	dhrs4	6.69	12.50	-0.90	0.00
XLOC_028946	tp53bp2b	0.64	1.76	-1.45	0.00
XLOC_000327	-	5.95	14.84	-1.32	0.00
XLOC_001131	cyp8b2	1304.07	459.04	1.51	0.00
XLOC_001311	-	2.87	0.66	2.12	0.00
XLOC_003221	gpx4a	698.00	1599.63	-1.20	0.00
XLOC_009151	-	3.09	10.48	-1.76	0.00
XLOC_011521	ENSONIG00000012166	227.85	116.58	0.97	0.00
XLOC_012219	-	1.54	11.60	-2.91	0.00
XLOC_017292	ENSONIG00000007421	1.20	3.38	-1.49	0.00
XLOC_018330	zmiz1b	1.84	0.90	1.03	0.00
XLOC_018840	-	12.30	5.40	1.19	0.00
XLOC_020782	adamts17	3.09	8.43	-1.45	0.00
XLOC_021461	hibadhb	18.23	9.69	0.91	0.00
XLOC_024656	zgc:194224	2.85	0.47	2.60	0.00
XLOC_024790	-	47.45	16.41	1.53	0.00
XLOC_024910	FBN1	6.96	13.39	-0.94	0.00
XLOC_000245	-	18.43	40.13	-1.12	0.00
XLOC_001410	-	0	1.90	-inf	0.00
XLOC_002690	ENSONIG00000006163	3.21	7.41	-1.21	0.00
XLOC_004512	ENSONIG00000001190	2684.35	10214.50	-1.93	0.00
XLOC_005013	pdlim1	22.66	12.34	0.88	0.00
XLOC_010584	neurl1b	0.37	1.47	-1.99	0.00
XLOC_013866	fam212ab	1.71	5.02	-1.55	0.00
XLOC_016557	thrap3a	1.34	3.18	-1.25	0.00
XLOC_018086	aup1	224.16	64.01	1.81	0.00

XLOC_022291	fam78ab	1.02	3.56	-1.80	0.00
XLOC_026673	-	100.59	49.73	1.02	0.00
XLOC_005981	MRC2	3.87	1.89	1.03	0.00
XLOC_006696	ARL8B	15.24	8.11	0.91	0.00
XLOC_009497	ENSONIG00000020520	8.79	4.39	1.00	0.00
XLOC_012279	c19h1orf109	4.03	1.37	1.55	0.00
XLOC_013506	vetz	0.87	1.85	-1.08	0.00
XLOC_014789	ndufa7	19.27	10.10	0.93	0.00
XLOC_018423	si: zfos-932h1.3	2.26	0.94	1.26	0.00
XLOC_021375	-	18.98	36.21	-0.93	0.00
XLOC_028701	rap1gap2a	0.13	1.22	-3.23	0.00
XLOC_030124	-	112.31	3.05	5.20	0.00
XLOC_000425	-	52.61	24.15	1.12	0.00
XLOC_012833	ENSONIG00000004284	0.67	2.82	-2.08	0.00
XLOC_013480	-	49.85	25.30	0.98	0.00
XLOC_017684	PPP2CA (1 of many)	120.75	56.97	1.08	0.00
XLOC_018940	eomesa	0.17	1.96	-3.51	0.00
XLOC_020830	zdhhc8a	0.45	1.50	-1.72	0.00
XLOC_021694	rad54l2	2.75	1.29	1.09	0.00
XLOC_029912	-	11.24	5.42	1.05	0.00
XLOC_000919	ENSONIG00000021433	0.96	3.82	-1.99	0.00
XLOC_001921	-	0.46	12.66	-4.78	0.00
XLOC_004693	-	7.97	0	inf	0.00
XLOC_004737	si:dkey-275b16.2	5.70	14.26	-1.32	0.00
XLOC_005607	socs3b	16.86	5.19	1.70	0.00
XLOC_008572	-	2.62	0	inf	0.00
XLOC_014525	mpp6b	1.02	0.29	1.83	0.00
XLOC_019241	lysmd3	4.81	2.42	0.99	0.00
XLOC_019361	-	3.42	0	inf	0.00
XLOC_020520	-	34.06	1.47	4.54	0.00
XLOC_027215	dgkzb	1.06	3.10	-1.55	0.00
XLOC_027719	-	44.45	0	inf	0.00
XLOC_029330	-	7.83	0	inf	0.00
XLOC_030477	capn2b	5.62	10.72	-0.93	0.00
XLOC_031031	-	12.46	0	inf	0.00
XLOC_002678	yes1	0.19	1.20	-2.64	0.00
XLOC_003498	itpa	13.78	6.33	1.12	0.00
XLOC_003830	prkab1a	1.45	3.14	-1.12	0.00
XLOC_009418	ela2	882.39	4533.20	-2.36	0.00
XLOC_016530	ENSONIG00000016353	1.69	4.83	-1.52	0.00
XLOC_022470	c18h3orf33	12.99	5.46	1.25	0.00
XLOC_022523	ENSONIG00000015441	7.51	15.42	-1.04	0.00
XLOC_027840	pmpcb	10.95	5.55	0.98	0.00
XLOC_011743	taf1b	3.14	1.07	1.56	0.00
XLOC_012388	apoea	1.64	4.49	-1.45	0.00
XLOC_017412	atp2c1	11.14	6.04	0.88	0.00
XLOC_018312	si:ch211-233m11.2	0.87	0.21	2.03	0.00
XLOC_021945	-	111.68	29.37	1.93	0.00

XLOC_003685	hic1	1.20	0.30	1.99	0.00
XLOC_004344	-	3.64	1.53	1.25	0.00
XLOC_009828	ENSONIG00000002689	0.55	2.52	-2.20	0.00
XLOC_011383	B4GALNT2 (1 of many)	1.14	0.15	2.89	0.00
XLOC_018483	ENSONIG00000006837	5.25	12.35	-1.23	0.00
XLOC_020343	si:ch211-117l17.5	4.00	8.76	-1.13	0.00
XLOC_023417	slc7a2	33.12	16.17	1.03	0.00
XLOC_025624	BPHL	16.49	30.64	-0.89	0.00
XLOC_030196	ENSONIG00000015215	173.65	77.21	1.17	0.00
XLOC_002501	map3k20	6.47	3.49	0.89	0.00
XLOC_002881	si:dkey-12e7.4	3.61	0.50	2.86	0.00
XLOC_004799	mgme1	1.69	5.07	-1.59	0.00
XLOC_005938	-	9.68	4.66	1.05	0.00
XLOC_007313	s1pr5a	5.21	2.42	1.11	0.00
XLOC_011613	cyp20a1	18.00	9.63	0.90	0.00
XLOC_011941	-	6.51	2.24	1.54	0.00
XLOC_013537	TYMP	1.94	5.21	-1.43	0.00
XLOC_015390	-	1.07	2.81	-1.40	0.00
XLOC_015544	si:ch211-208h7.4	2.57	5.71	-1.15	0.00
XLOC_019594	tnfsf13b	0.76	2.85	-1.90	0.00
XLOC_020137	sema4gb	0.98	0.32	1.60	0.00
XLOC_021558	psmc3	48.10	26.39	0.87	0.00
XLOC_023219	-	11.07	3.35	1.72	0.00
XLOC_024238	tekt1	0.29	1.74	-2.59	0.00
XLOC_028406	-	54.70	266.94	-2.29	0.00
XLOC_029628	-	1.00	4.43	-2.14	0.00
XLOC_031122	ENSONIG00000017776	0.61	2.67	-2.13	0.00
XLOC_006239	cstf3	9.93	5.17	0.94	0.00
XLOC_007133	atg9a	2.00	4.09	-1.03	0.00
XLOC_008580	dph3	3.31	0.53	2.65	0.00
XLOC_013835	-	0	6.83	-inf	0.00
XLOC_018410	golgb1	4.43	2.28	0.95	0.00
XLOC_019589	ing1	9.58	4.61	1.06	0.00
XLOC_027473	-	79.48	40.34	0.98	0.00
XLOC_027993	mrpl17	10.20	4.44	1.20	0.00
XLOC_003702	synrg	5.83	3.15	0.89	0.00
XLOC_010947	-	1.57	8.58	-2.45	0.00
XLOC_011669	-	2.51	0.64	1.96	0.00
XLOC_011759	-	199.26	10.34	4.27	0.00
XLOC_013583	znf385a	0.16	0.75	-2.27	0.00
XLOC_014318	-	11.69	32.44	-1.47	0.00
XLOC_014853	prex1	0.72	1.52	-1.07	0.00
XLOC_016978	-	17.39	7.97	1.13	0.00
XLOC_019062	CARTPT (1 of many)	1.29	7.14	-2.47	0.00
XLOC_019092	-	6.00	0	inf	0.00
XLOC_022011	-	0.14	2.93	-4.37	0.00
XLOC_023045	nudt18	3.89	1.59	1.29	0.00
XLOC_023087	-	0.45	2.59	-2.53	0.00

XLOC_005700	casp7	0.41	2.09	-2.35	0.00
XLOC_006832	uqcrc1	62.72	33.31	0.91	0.00
XLOC_021755	dtd1	6.77	2.93	1.21	0.00
XLOC_023130	LMAN2L (1 of many)	1.41	0.11	3.69	0.00
XLOC_024643	gdap2	6.47	3.45	0.91	0.00
XLOC_028862	-	61.61	15.20	2.02	0.00
XLOC_029913	-	15.14	4.99	1.60	0.00
XLOC_003806	arrdc1b	0.50	1.35	-1.44	0.00
XLOC_010946	-	2.99	11.17	-1.90	0.00
XLOC_012534	cdca7a	0.66	2.45	-1.89	0.00
XLOC_013228	-	6.03	0.46	3.72	0.00
XLOC_016482	myo18aa	1.21	0.55	1.14	0.00
XLOC_017834	tfg	36.08	19.37	0.90	0.00
XLOC_024980	rnf121	36.00	19.10	0.91	0.00
XLOC_025880	dhrs1	32.86	60.80	-0.89	0.00
XLOC_000428	-	0	2.20	-inf	0.00
XLOC_001143	ube2v2	54.03	29.39	0.88	0.00
XLOC_008516	ENSONIG00000002493	0.27	4.33	-4.02	0.00
XLOC_008720	tmem131	18.29	9.82	0.90	0.00
XLOC_014506	pef1	3.25	6.70	-1.04	0.00
XLOC_016349	-	0	2.05	-inf	0.00
XLOC_021905	-	0	9.91	-inf	0.00
XLOC_023663	-	0	4.43	-inf	0.00
XLOC_023771	-	0	1.73	-inf	0.00
XLOC_029939	-	0	3.31	-inf	0.00
XLOC_004540	thbd	2.86	0.80	1.84	0.00
XLOC_005147	ARFGAP1	5.55	2.89	0.94	0.00
XLOC_005298	nr1d4b	0.87	2.19	-1.33	0.00
XLOC_008563	ctss1	1.28	3.74	-1.54	0.00
XLOC_010879	tmed7	24.04	12.86	0.90	0.00
XLOC_013413	PRF1 (1 of many)	0.18	2.75	-3.91	0.00
XLOC_014895	bcas2	12.26	6.24	0.97	0.00
XLOC_020781	-	6.16	2.22	1.48	0.00
XLOC_028281	-	6.50	0.60	3.44	0.00
XLOC_007757	bmi1a	5.36	2.18	1.30	0.00
XLOC_012835	pcdh12	2.79	5.25	-0.91	0.00
XLOC_019934	-	2.25	9.49	-2.08	0.00
XLOC_022858	ptger3	0.91	0.15	2.57	0.00
XLOC_022961	bmpr1aa	26.35	14.22	0.89	0.00
XLOC_026432	xdh	38.11	19.07	1.00	0.00
XLOC_026546	tbc1d22b	0.26	1.10	-2.07	0.00
XLOC_027211	-	5.59	46.31	-3.05	0.00
XLOC_027520	yars	12.97	6.99	0.89	0.00
XLOC_029864	-	4.87	44.82	-3.20	0.00
XLOC_030184	-	1.10	3.87	-1.82	0.00
XLOC_030521	-	50.00	11.47	2.12	0.00
XLOC_000865	ENSONIG00000009630	20.88	43.45	-1.06	0.00
XLOC_005373	myl9a	10.61	19.61	-0.89	0.00

XLOC_006850	ENSONIG00000020363	0.21	1.35	-2.71	0.00
XLOC_007951	-	0	37.68	-inf	0.00
XLOC_008199	-	0.40	5.18	-3.68	0.00
XLOC_017998	clip1a	6.93	3.69	0.91	0.00
XLOC_021669	-	17.40	6.87	1.34	0.00
XLOC_021835	-	0	2.11	-inf	0.00
XLOC_026268	-	8.72	4.55	0.94	0.00
XLOC_026314	-	0	6.04	-inf	0.00
XLOC_028128	-	0	5.84	-inf	0.00
XLOC_028395	-	0	20.22	-inf	0.00
XLOC_000343	-	8.52	2.86	1.57	0.00
XLOC_001324	-	303.79	1605.74	-2.40	0.00
XLOC_005636	-	3.67	8.42	-1.20	0.00
XLOC_007259	adsl (1 of many)	3.93	1.75	1.17	0.00
XLOC_008487	yrk	8.86	18.91	-1.09	0.00
XLOC_011204	ENSONIG00000005757	0.72	2.63	-1.87	0.00
XLOC_015036	-	79.94	145.91	-0.87	0.00
XLOC_015139	tnfsf12	0.89	2.54	-1.52	0.00
XLOC_025066	iqgap1	2.68	4.86	-0.86	0.00
XLOC_025501	mapkbp1	2.64	1.33	0.99	0.00
XLOC_026144	cxxc1a	1.38	3.25	-1.23	0.00
XLOC_028990	ENSONIG00000017891	9.00	3.96	1.18	0.00
XLOC_000168	ENSONIG00000009426	1.71	7.10	-2.05	0.00
XLOC_001805	cog7	6.49	3.51	0.89	0.00
XLOC_013002	cyp1c2	3.64	1.15	1.67	0.00
XLOC_018381	-	7.16	3.60	0.99	0.00
XLOC_024869	ccr12a	1.97	5.47	-1.48	0.00
XLOC_006549	cdipt	6.94	2.92	1.25	0.00
XLOC_013809	ENSONIG00000004342	174.25	0.70	7.96	0.00
XLOC_015350	ddx3a	43.09	22.10	0.96	0.00
XLOC_017682	vdac1	6.40	13.33	-1.06	0.00
XLOC_018697	ENSONIG00000017414	0.30	3.15	-3.41	0.00
XLOC_021144	taok3a	3.26	6.31	-0.95	0.00
XLOC_000087	-	7.55	15.35	-1.02	0.00
XLOC_002600	ENSONIG00000006407	9.47	3.28	1.53	0.00
XLOC_007480	sdpra	0.72	1.68	-1.21	0.00
XLOC_015599	pnpla3	16.01	29.57	-0.89	0.00
XLOC_019599	-	19.70	9.52	1.05	0.00
XLOC_020351	EEF1D	15.31	8.24	0.89	0.00
XLOC_021483	upp1	6.44	2.72	1.24	0.00
XLOC_021707	-	0	1.73	-inf	0.00
XLOC_024397	-	0	2.79	-inf	0.00
XLOC_025541	-	10.41	4.82	1.11	0.00
XLOC_027551	r3hdm4 (1 of many)	10.79	4.41	1.29	0.00
XLOC_001664	wbp2	20.99	10.32	1.02	0.00
XLOC_001958	top2a	0.25	0.79	-1.63	0.00
XLOC_002762	ENSONIG00000010602	0.46	1.41	-1.61	0.00
XLOC_003736	fam102ab	0.09	1.34	-3.93	0.00

XLOC_008023	ctps1a	9.92	23.59	-1.25	0.00
XLOC_009234	-	431.59	209.55	1.04	0.00
XLOC_010768	commd7	10.16	4.00	1.34	0.00
XLOC_010995	ENSONIG00000007996	4.76	9.34	-0.97	0.00
XLOC_013470	ENSONIG00000021220	0.57	2.39	-2.06	0.00
XLOC_014747	ENSONIG00000012749	32.29	15.47	1.06	0.00
XLOC_016084	baiap2a	0.84	2.52	-1.58	0.00
XLOC_019848	rab2a	24.14	13.15	0.88	0.00
XLOC_026816	ENSONIG00000004480	0.91	2.33	-1.36	0.00
XLOC_001201	ap1s3b	12.05	5.58	1.11	0.00
XLOC_002023	ENSONIG00000020089	0.20	0.84	-2.04	0.00
XLOC_010449	il1fma	0.37	1.38	-1.90	0.00
XLOC_013749	-	7.37	2.15	1.78	0.00
XLOC_019750	dlst (1 of many)	134.16	70.04	0.94	0.00
XLOC_021550	hk1 (1 of many)	2.78	5.60	-1.01	0.00
XLOC_026835	c1qbp	44.10	24.42	0.85	0.00
XLOC_030097	ENSONIG00000007649	2.37	7.22	-1.61	0.00
XLOC_002886	sf3b3	5.18	2.81	0.88	0.00
XLOC_003935	rfx4	1.02	0.24	2.10	0.00
XLOC_009880	gla	1.03	3.78	-1.88	0.00
XLOC_018826	ebna1bp2	6.36	3.31	0.94	0.00
XLOC_020392	ppm1db	3.31	1.26	1.39	0.00
XLOC_025279	ydjc	4.95	2.17	1.19	0.00
XLOC_027341	stat4	0.59	1.67	-1.49	0.00
XLOC_027518	abhd5b	2.96	6.17	-1.06	0.00
XLOC_005403	zgc:114130	4.43	1.77	1.33	0.00
XLOC_007902	grb10b	8.37	3.80	1.14	0.00
XLOC_007914	fbxo32	0.76	2.26	-1.56	0.00
XLOC_009314	-	15.94	7.45	1.10	0.00
XLOC_022887	ENSONIG00000015580	0.77	3.49	-2.17	0.00
XLOC_024456	ENSONIG00000011619	22.98	43.11	-0.91	0.00
XLOC_029393	)000020408,HIST2H2AE	37.49	9.84	1.93	0.00
XLOC_002981	zgc:91860	2.27	0.76	1.58	0.00
XLOC_009879	btk	2.21	8.18	-1.89	0.00
XLOC_011749	-	4.73	0.70	2.75	0.00
XLOC_011757	zbtb1	3.78	2.03	0.90	0.00
XLOC_015313	ACTA1	9.36	4.70	0.99	0.00
XLOC_017567	-	26.81	12.34	1.12	0.00
XLOC_017716	tbl2	12.66	6.10	1.05	0.00
XLOC_022189	ccnyl1 (1 of many)	1.64	0.57	1.53	0.00
XLOC_023439	exosc1,pgam1b	30.90	14.01	1.14	0.00
XLOC_028678	-	7.39	0	inf	0.00
XLOC_004449	dll4	8.50	3.71	1.19	0.00
XLOC_006259	ENSONIG00000005710	0.30	1.46	-2.27	0.00
XLOC_009985	-	1.32	10.99	-3.05	0.00
XLOC_011462	-	0.84	6.11	-2.86	0.00
XLOC_012371	ncdn	1.65	0.62	1.42	0.00
XLOC_020953	-	8.35	1.73	2.27	0.00

XLOC_022584	-	1.75	5.24	-1.58	0.00
XLOC_025239	pfdn1	196.19	105.47	0.90	0.00
XLOC_001700	rangap1b	3.36	1.23	1.44	0.01
XLOC_003198	-	17.62	9.21	0.94	0.01
XLOC_009089	slc43a3b	2.34	4.58	-0.97	0.01
XLOC_015448	ptcd2	3.38	1.38	1.29	0.01
XLOC_029018	TMPRSS6	22.27	41.46	-0.90	0.01
XLOC_000234	-	61.58	121.36	-0.98	0.01
XLOC_000258	-	0	2.04	-inf	0.01
XLOC_002333	-	0	2.67	-inf	0.01
XLOC_011492	slc41a1	2.62	1.00	1.39	0.01
XLOC_015698	-	0	11.67	-inf	0.01
XLOC_016543	-	0	3.60	-inf	0.01
XLOC_018379	si:ch211-262i1.5	1.59	0.20	3.02	0.01
XLOC_019606	-	44.90	0	inf	0.01
XLOC_021071	-	0	48.83	-inf	0.01
XLOC_024720	degs1	48.83	26.47	0.88	0.01
XLOC_031163	ENSONIG00000021066	3.44	0.57	2.59	0.01
XLOC_031277	-	0	2.05	-inf	0.01
XLOC_003776	txnl1	20.62	11.15	0.89	0.01
XLOC_007406	bola1	9.23	4.22	1.13	0.01
XLOC_007885	MTSS1 (1 of many)	20.03	10.79	0.89	0.01
XLOC_015996	-	6.37	2.35	1.44	0.01
XLOC_016647	lpcat3	7.05	3.27	1.11	0.01
XLOC_017327	ENSONIG00000007487	0.06	1.70	-4.74	0.01
XLOC_002358	slc38a5a	21.02	11.66	0.85	0.01
XLOC_002753	-	8.08	18.43	-1.19	0.01
XLOC_006214	trim66	0.90	2.16	-1.26	0.01
XLOC_017513	gpatch4	2.17	0.93	1.22	0.01
XLOC_018288	zgc:123010	3.68	1.89	0.96	0.01
XLOC_019122	stim1b	1.74	4.32	-1.31	0.01
XLOC_026332	-	11.08	32.46	-1.55	0.01
XLOC_027204	kl	21.05	47.21	-1.17	0.01
XLOC_027505	slc6a18	0.22	1.59	-2.87	0.01
XLOC_030319	foxp3b	0.05	0.89	-4.10	0.01
XLOC_001090	chmp5a	4.36	1.72	1.34	0.01
XLOC_006000	kat2a	0.86	0.27	1.65	0.01
XLOC_007702	naa50	10.76	5.77	0.90	0.01
XLOC_009999	ENSONIG00000004471	0.40	1.24	-1.65	0.01
XLOC_013886	fkbp5	132.42	53.45	1.31	0.01
XLOC_019253	-	0	50.30	-inf	0.01
XLOC_029857	IL4I1 (1 of many)	0.64	2.26	-1.82	0.01
XLOC_013565	stat6	2.20	4.30	-0.97	0.01
XLOC_023974	zswim7	2.52	0.72	1.80	0.01
XLOC_024239	inpp5ka	1.61	3.68	-1.19	0.01
XLOC_026603	-	36.31	0	inf	0.01
XLOC_027463	-	15.77	0	inf	0.01
XLOC_000213	-	21.69	1.87	3.54	0.01

XLOC_008494	fabp3	127.69	254.37	-0.99	0.01
XLOC_011235	ezrb	3.18	6.96	-1.13	0.01
XLOC_020006	LEPROT	9.19	16.65	-0.86	0.01
XLOC_020010	-	0.65	3.73	-2.53	0.01
XLOC_028338	zgc:113162	0.40	2.42	-2.58	0.01
XLOC_006412	-	1.94	8.22	-2.08	0.01
XLOC_008002	sdha	23.01	12.61	0.87	0.01
XLOC_012756	-	6.56	12.46	-0.93	0.01
XLOC_017544	sec22bb	79.36	43.88	0.85	0.01
XLOC_026856	-	0.80	3.60	-2.18	0.01
XLOC_027071	-	1.83	15.61	-3.09	0.01
XLOC_029349	-	1.17	0.19	2.62	0.01
XLOC_001383	-	0.48	2.22	-2.20	0.01
XLOC_008282	tp53i11b	3.19	6.08	-0.93	0.01
XLOC_012290	map7d1b	4.41	8.05	-0.87	0.01
XLOC_012786	ENSONIG00000012900	199.12	445.47	-1.16	0.01
XLOC_015064	porb	67.88	35.61	0.93	0.01
XLOC_017211	gck	0.11	156.15	-10.45	0.01
XLOC_020323	fetub (1 of many)	1445.65	4813.73	-1.74	0.01
XLOC_023220	pinx1	4.61	1.44	1.68	0.01
XLOC_029238	-	26.69	12.83	1.06	0.01
XLOC_001106	-	0.65	2.10	-1.70	0.01
XLOC_007054	TP53I3	0.21	5.60	-4.75	0.01
XLOC_012374	eva1bb	3.12	0.55	2.50	0.01
XLOC_016162	ttc36	451.03	240.50	0.91	0.01
XLOC_019733	gstz1	96.86	179.82	-0.89	0.01
XLOC_025596	twistnb	10.19	5.19	0.97	0.01
XLOC_027723	psph	19.64	4.33	2.18	0.01
XLOC_031174	-	10.68	2.13	2.33	0.01
XLOC_004859	-	2.47	12.20	-2.31	0.01
XLOC_020009	-	0.43	3.27	-2.93	0.01
XLOC_020739	zgc:77056	2.86	7.46	-1.39	0.01
XLOC_024262	-	4.30	1.79	1.26	0.01
XLOC_026552	sps2	6.54	11.86	-0.86	0.01
XLOC_001758	arsg	16.43	30.49	-0.89	0.01
XLOC_005122	mettl1	10.35	3.98	1.38	0.01
XLOC_005995	ENSONIG00000001073	41.96	79.13	-0.92	0.01
XLOC_008121	-	0	5.07	-inf	0.01
XLOC_008457	clptm1l	15.99	8.88	0.85	0.01
XLOC_011048	-	0	3.77	-inf	0.01
XLOC_013488	-	0	14.73	-inf	0.01
XLOC_017375	-	0	4.44	-inf	0.01
XLOC_017709	-	0	2.87	-inf	0.01
XLOC_018368	-	0	3.44	-inf	0.01
XLOC_018411	hcls1,pex26	3.08	10.78	-1.81	0.01
XLOC_021235	dnaja1	7.83	4.12	0.92	0.01
XLOC_021637	-	0	2.22	-inf	0.01
XLOC_022823	pfn2l	33.02	13.45	1.30	0.01

XLOC_023911	-	0	2.25	-inf	0.01
XLOC_025316	-	0	2.04	-inf	0.01
XLOC_025347	-	0	2.78	-inf	0.01
XLOC_026324	-	0	1.60	-inf	0.01
XLOC_028045	-	0	2.68	-inf	0.01
XLOC_028407	-	0	615.51	-inf	0.01
XLOC_028927	-	45.45	98.79	-1.12	0.01
XLOC_028945	-	0	14.73	-inf	0.01
XLOC_029190	-	0	5.73	-inf	0.01
XLOC_029336	-	12.72	6.25	1.03	0.01
XLOC_006021	gpatch8	16.40	30.22	-0.88	0.01
XLOC_008427	fabp11a	39.22	71.09	-0.86	0.01
XLOC_010696	-	1.48	570.26	-8.59	0.01
XLOC_016839	bbs4	3.38	1.45	1.22	0.01
XLOC_020966	RAB37	1.29	4.24	-1.72	0.01
XLOC_022975	mospd2	31.55	17.30	0.87	0.01
XLOC_027830	arap3	2.50	1.33	0.91	0.01
XLOC_027931	pelo	3.84	1.89	1.02	0.01
XLOC_028540	ENSONIG00000005475	0.15	1.19	-3.03	0.01
XLOC_030963	-	0	13.88	-inf	0.01
XLOC_029049	-	0.47	2.19	-2.21	0.01
XLOC_006932	efna2a	1.72	0.39	2.15	0.01
XLOC_008821	-	12.92	25.18	-0.96	0.01
XLOC_008840	ube2g1b	6.08	1.96	1.63	0.01
XLOC_015721	tcp1	35.79	17.82	1.01	0.01
XLOC_020357	bcas3	2.22	0.88	1.34	0.01
XLOC_023324	pcgf5a	2.13	4.72	-1.15	0.01
XLOC_029572	dpp4	7.11	13.55	-0.93	0.01
XLOC_001421	-	1.16	3.35	-1.53	0.01
XLOC_006125	ddx21	179.33	88.47	1.02	0.01
XLOC_010412	nsun3	3.05	1.06	1.52	0.01
XLOC_015968	-	2.16	5.04	-1.22	0.01
XLOC_016049	coro7	0.92	2.30	-1.32	0.01
XLOC_022202	ENSONIG00000017002	8.47	3.94	1.10	0.01
XLOC_025439	-	28.99	15.12	0.94	0.01
XLOC_027752	-	2.15	0.65	1.73	0.01
XLOC_000726	vps13c	1.43	2.55	-0.84	0.01
XLOC_006439	mmd	22.68	12.55	0.85	0.01
XLOC_016270	ch211-215k15.4 (1 of ma	6.94	2.84	1.29	0.01
XLOC_016845	slc25a44b	2.03	0.29	2.82	0.01
XLOC_017287	-	2.32	0	inf	0.01
XLOC_017301	-	15.93	0	inf	0.01
XLOC_017321	-	3.03	0	inf	0.01
XLOC_018168	ENSONIG00000005135	28.33	15.85	0.84	0.01
XLOC_020177	-	7.41	0	inf	0.01
XLOC_020371	srfa	2.27	5.05	-1.15	0.01
XLOC_021650	cyb561d2	22.86	4.01	2.51	0.01
XLOC_023443	-	5.19	2.82	0.88	0.01

XLOC_023738	-	5.99	0	inf	0.01
XLOC_023971	-	4566.20	1862.62	1.29	0.01
XLOC_026519	pgm2l1	6.55	3.32	0.98	0.01
XLOC_028847	-	10.99	0	inf	0.01
XLOC_001783	-	4.82	1.02	2.24	0.01
XLOC_011966	dpagt1	83.46	45.38	0.88	0.01
XLOC_017059	sphk2	3.17	6.59	-1.05	0.01
XLOC_021716	-	0.31	2.24	-2.86	0.01
XLOC_030185	-	2.23	5.65	-1.34	0.01
XLOC_000363	-	0.41	13.07	-4.98	0.01
XLOC_002929	ddx56	8.17	4.27	0.94	0.01
XLOC_018971	ENSONIG00000019763	18.70	68.06	-1.86	0.01
XLOC_021572	smyd3	1.68	4.47	-1.41	0.01
XLOC_025334	ctdsp2	9.44	17.83	-0.92	0.01
XLOC_003980	gtf3c6	19.03	8.48	1.17	0.01
XLOC_004265	trim35-30	0.11	0.94	-3.14	0.01
XLOC_013994	myo9b	1.02	0.44	1.20	0.01
XLOC_014119	zgc:114173	2.93	7.75	-1.40	0.01
XLOC_019936	id2b	9.85	21.59	-1.13	0.01
XLOC_020863	-	0.49	2.37	-2.28	0.01
XLOC_022281	-	9.80	35.09	-1.84	0.01
XLOC_024853	ift172	4.84	0.12	5.40	0.01
XLOC_025862	gorasp1a	9.87	5.45	0.86	0.01
XLOC_001186	ubxn7	4.70	8.73	-0.89	0.01
XLOC_007589	ldb2a	2.41	4.71	-0.97	0.01
XLOC_008710	agxta,dtymk	364.37	151.19	1.27	0.01
XLOC_010321	-	0.30	1.83	-2.62	0.01
XLOC_012005	ENSONIG00000008531	6.08	14.35	-1.24	0.01
XLOC_012205	ENSONIG00000010218	39.10	69.77	-0.84	0.01
XLOC_012825	slc26a2	1.31	0.35	1.90	0.01
XLOC_015315	noc3l	8.46	4.64	0.87	0.01
XLOC_017757	agxt2	2274.61	789.51	1.37	0.01
XLOC_018112	-	1.43	10.27	-2.85	0.01
XLOC_027663	cd99	2.79	5.60	-1.00	0.01
XLOC_000699	mef2ab	0.78	1.95	-1.32	0.01
XLOC_003929	bhlhe41	1.87	0.67	1.49	0.01
XLOC_007716	mtfr1	8.01	3.97	1.01	0.01
XLOC_009505	-	0.67	3.82	-2.51	0.01
XLOC_011360	yipf2	13.21	7.30	0.86	0.01
XLOC_019756	degs2	1.67	4.48	-1.42	0.01
XLOC_024007	abhd4	6.34	3.00	1.08	0.01
XLOC_027327	wdr75	5.80	3.11	0.90	0.01
XLOC_000474	styx	12.68	5.27	1.27	0.01
XLOC_004459	dh6a1 (1 of many),entpd5	9.95	20.29	-1.03	0.01
XLOC_007722	prex2	1.51	0.75	1.02	0.01
XLOC_008848	-	10.25	2.82	1.86	0.01
XLOC_009280	-	20.40	3.56	2.52	0.01
XLOC_014546	hibadha	13.39	7.23	0.89	0.01

XLOC_018306	ccar1	7.57	13.80	-0.87	0.01
XLOC_020907	si:dkeyp-23e4.3	1.53	0.66	1.20	0.01
XLOC_028187	-	1.00	5.48	-2.46	0.01
XLOC_000254	-	0	2.21	-inf	0.01
XLOC_000528	sall1a	9.25	19.26	-1.06	0.01
XLOC_010735	-	8.54	15.29	-0.84	0.01
XLOC_018011	si:ch211-106a19.1	0.44	1.37	-1.63	0.01
XLOC_020204	tmem248	0.85	2.93	-1.79	0.01
XLOC_027464	-	1.48	4.39	-1.57	0.01
XLOC_028126	-	0	5.71	-inf	0.01
XLOC_002776	zgc:162634	27.09	14.89	0.86	0.01
XLOC_007168	-	0.14	1.80	-3.65	0.01
XLOC_009327	:ch73-21k16.5 (1 of many	0.14	2.29	-4.05	0.01
XLOC_003228	si:ch211-129c21.1	1.65	3.40	-1.04	0.01
XLOC_003525	-	15.21	3.16	2.27	0.01
XLOC_004071	INCENP	0.20	0.72	-1.83	0.01
XLOC_000622	cry5	2.08	0.57	1.87	0.01
XLOC_004422	-	6.85	3.26	1.07	0.01
XLOC_005604	sfxn2	7.02	3.47	1.02	0.01
XLOC_029036	-	9.34	4.91	0.93	0.01
XLOC_001441	peli1b	0.31	0.87	-1.50	0.01
XLOC_014880	-	6.78	3.05	1.15	0.01
XLOC_015360	akap11	0.96	1.82	-0.92	0.01
XLOC_030589	-	19.42	0	inf	0.01
XLOC_003170	xcr1a.1	0.68	2.73	-2.01	0.01
XLOC_012400	zgc:109744	4.80	2.32	1.05	0.01
XLOC_012877	tomm34 (1 of many)	9.62	5.04	0.93	0.01
XLOC_024093	ssuh2.1	0.29	1.16	-1.99	0.01
XLOC_013188	-	0.95	2.38	-1.33	0.01
XLOC_014709	-	12.24	60.24	-2.30	0.01
XLOC_014728	arid3a	0.68	0.14	2.31	0.01
XLOC_017476	rpp25l	10.74	5.03	1.09	0.01
XLOC_018413	ccnd2b	1.56	3.19	-1.03	0.01
XLOC_020483	-	51.16	90.82	-0.83	0.01
XLOC_023713	cyp3a65 (1 of many)	15.52	31.44	-1.02	0.01
XLOC_028401	-	0	32.54	-inf	0.01
XLOC_029710	BOLA2	6.33	3.32	0.93	0.01
XLOC_003092	tln1	1.15	2.11	-0.88	0.01
XLOC_008778	ak3	4.22	9.79	-1.21	0.01
XLOC_017494	psmc4	12.92	7.34	0.82	0.01
XLOC_024105	-	50.78	28.22	0.85	0.01
XLOC_026863	WDR37 (1 of many)	1.66	0.51	1.69	0.01
XLOC_027900	cnpy4	3.09	0.70	2.13	0.01
XLOC_030281	ENSONIG00000006679	3.78	1.17	1.70	0.01
XLOC_000514	zgc:101723	12.56	26.57	-1.08	0.01
XLOC_000718	-	0.94	2.89	-1.63	0.01
XLOC_003001	ciapin1	10.57	5.73	0.88	0.01
XLOC_003913	-	0.72	5.89	-3.03	0.01

XLOC_004401	-	104.23	413.10	-1.99	0.01
XLOC_004792	psap	106.92	203.14	-0.93	0.01
XLOC_007905	ddc	0.44	1.37	-1.63	0.01
XLOC_009290	PRKAR2A (1 of many)	6.72	12.57	-0.90	0.01
XLOC_015030	cirh1a	4.19	2.01	1.06	0.01
XLOC_021573	ENSONIG00000017137	3.93	7.16	-0.86	0.01
XLOC_022481	-	2.11	8.00	-1.92	0.01
XLOC_023463	sybl1	21.68	11.34	0.93	0.01
XLOC_028270	ncbp2	21.18	10.47	1.02	0.01
XLOC_029398	-	40.97	73.68	-0.85	0.01
XLOC_000920	mrps14	49.37	24.44	1.01	0.01
XLOC_002222	abtb1	1.71	4.57	-1.42	0.01
XLOC_003190	fh	72.11	38.51	0.90	0.01
XLOC_005175	ube2j2	19.48	11.06	0.82	0.01
XLOC_008854	si:ch211-282j22.3	2.63	1.38	0.93	0.01
XLOC_009464	dnase1l3l	0.74	3.99	-2.43	0.01
XLOC_011446	cass4	0.19	0.81	-2.08	0.01
XLOC_011455	-	13.93	7.40	0.91	0.01
XLOC_012202	-	150.79	295.02	-0.97	0.01
XLOC_016136	CXCR5	0.39	4.93	-3.66	0.01
XLOC_028074	plcd4b	0.90	2.40	-1.42	0.01
XLOC_001153	DHX30	2.43	0.53	2.20	0.01
XLOC_003918	zgc:153343	3.38	0.95	1.84	0.01
XLOC_004406	slc2a12	0.25	1.54	-2.64	0.01
XLOC_006269	ogfod1	1.70	0.74	1.21	0.01
XLOC_007186	slc25a38a	62.05	30.96	1.00	0.01
XLOC_012098	-	13.13	23.62	-0.85	0.01
XLOC_015531	fat1a	4.47	2.53	0.82	0.01
XLOC_016025	cdc42ep4a	4.71	8.76	-0.90	0.01
XLOC_018682	-	9.29	4.62	1.01	0.01
XLOC_021781	SMARCE1 (1 of many)	3.62	9.14	-1.34	0.01
XLOC_022839	TSC22D2	4.60	1.95	1.24	0.01
XLOC_030200	ENSONIG00000015217	820.63	364.09	1.17	0.01
XLOC_000295	-	7.56	2.83	1.42	0.01
XLOC_028760	-	79.76	11.12	2.84	0.01
XLOC_000768	eftud1	2.19	0.92	1.25	0.01
XLOC_004798	cfl1l	141.89	281.84	-0.99	0.01
XLOC_006719	rpn1	156.83	82.04	0.93	0.01
XLOC_010921	atp5a1 (1 of many)	119.01	65.19	0.87	0.01
XLOC_016731	tapbpl	0.17	1.56	-3.19	0.01
XLOC_017077	tmem245	1.33	2.56	-0.95	0.01
XLOC_019700	eml1	1.76	3.32	-0.91	0.01
XLOC_006929	palm1b	1.66	6.02	-1.86	0.01
XLOC_020437	ythdc1	19.99	10.98	0.86	0.01
XLOC_028434	-	22.66	7.82	1.53	0.01
XLOC_000172	ENSONIG00000000238	7.90	1.36	2.53	0.01
XLOC_001976	-	0	2.24	-inf	0.01
XLOC_005564	-	0	2.74	-inf	0.01

XLOC_006293	-	0.74	4.18	-2.49	0.01
XLOC_009268	impdh2	5.28	2.51	1.07	0.01
XLOC_011059	fbl	37.85	21.37	0.82	0.01
XLOC_012735	-	0	3.45	-inf	0.01
XLOC_017188	AP3S1	7.00	3.31	1.08	0.01
XLOC_018492	-	0	26.41	-inf	0.01
XLOC_019829	impad1	1.77	0.47	1.90	0.01
XLOC_022436	entpd2a.2	0.39	2.30	-2.55	0.01
XLOC_026334	-	0	5.25	-inf	0.01
XLOC_026481	-	0	15.41	-inf	0.01
XLOC_027647	-	0	5.66	-inf	0.01
XLOC_028923	ENSONIG00000013631	0.25	1.41	-2.49	0.01
XLOC_029659	-	0	6.66	-inf	0.01
XLOC_030640	-	0	26.41	-inf	0.01
XLOC_008625	ttc39c	0.47	1.30	-1.48	0.01
XLOC_010228	-	12.98	4.78	1.44	0.01
XLOC_021391	-	2.54	7.95	-1.65	0.01
XLOC_025788	-	1.11	3.93	-1.82	0.01
XLOC_031335	ENSONIG00000009100	1.53	38.79	-4.67	0.01
XLOC_003938	-	19.10	8.51	1.17	0.01
XLOC_010284	ENSONIG00000010644	0.39	1.38	-1.82	0.01
XLOC_011332	kat7a	1.51	2.81	-0.90	0.01
XLOC_016435	mpp1	8.97	15.96	-0.83	0.01
XLOC_017431	mtx1b	20.16	9.98	1.01	0.01
XLOC_020427	-	4.53	2.14	1.08	0.01
XLOC_021222	DLC1	6.34	3.48	0.86	0.01
XLOC_023378	slc38a2	25.31	12.96	0.97	0.01
XLOC_001709	ubtd1b	4.35	1.43	1.61	0.01
XLOC_001800	nubp2	18.53	9.50	0.96	0.01
XLOC_008287	gpr22b	1.30	0.09	3.80	0.01
XLOC_009319	emc1	10.80	6.03	0.84	0.01
XLOC_011270	scpep1	9.06	19.03	-1.07	0.01
XLOC_012275	-	0	1.97	-inf	0.01
XLOC_016271	-	10.61	3.30	1.69	0.01
XLOC_017797	-	4.70	11.20	-1.25	0.01
XLOC_021077	-	0.57	2.05	-1.84	0.01
XLOC_024658	fam89a	3.78	0.86	2.14	0.01
XLOC_028498	-	1.40	6.32	-2.18	0.01
XLOC_001321	zgc:66475	2.09	0.61	1.78	0.01
XLOC_013692	mfsd5	12.00	6.26	0.94	0.01
XLOC_018253	arglu1a	11.12	19.98	-0.85	0.01
XLOC_021257	prkcz	1338.76	2362.17	-1.51	0.01
XLOC_021429	ENSONIG00000002743	0.31	1.59	-2.36	0.01
XLOC_021807	-	177.31	4.79	5.21	0.01
XLOC_029707	rwdd	7.33	3.14	1.22	0.01
XLOC_030188	-	55.65	14.00	1.99	0.01
XLOC_001622	SLC22A7 (1 of many)	7.80	3.16	1.30	0.01
XLOC_002430	PLXND1	3.47	6.55	-0.92	0.01

XLOC_002582	ccr9b	0.19	1.58	-3.03	0.01
XLOC_016655	ENSONIG00000013019	0.09	2.17	-4.62	0.01
XLOC_017393	ehd4	1.28	3.00	-1.23	0.01
XLOC_022228	-	2.24	1.07	1.06	0.01
XLOC_027788	arl11	0.69	5.71	-3.04	0.01
XLOC_028943	atat1	0.97	2.69	-1.47	0.01
XLOC_003513	VPS4B (1 of many)	6.84	3.14	1.12	0.01
XLOC_016211	slc12a7b	1.23	2.49	-1.02	0.01
XLOC_025556	-	393.72	216.70	0.86	0.01
XLOC_029024	-	3.66	10.77	-1.56	0.01
XLOC_006204	mrvi1	0.42	1.15	-1.46	0.01
XLOC_012016	fgf13b	1.01	0.17	2.53	0.01
XLOC_015686	ckma	0.76	3.39	-2.16	0.01
XLOC_019313	zgc:194578	1.40	0.50	1.49	0.01
XLOC_019508	tbl3	4.83	2.54	0.93	0.01
XLOC_020919	gys1	7.25	2.25	1.69	0.01
XLOC_000397	-	2.49	14.33	-2.53	0.01
XLOC_001945	WIPI1	0.63	2.30	-1.88	0.01
XLOC_002036	pfdn4	627.67	305.47	1.04	0.01
XLOC_007439	-	0.44	5.62	-3.68	0.01
XLOC_016319	-	0.45	2.41	-2.43	0.01
XLOC_022339	cdk1	0.92	2.91	-1.65	0.01
XLOC_026841	atp2a3	0.14	0.69	-2.33	0.01
XLOC_006529	ENSONIG00000009340	0.21	0.99	-2.21	0.01
XLOC_009407	-	3.10	0.54	2.51	0.01
XLOC_011832	zgc:100829	0.52	1.30	-1.32	0.01
XLOC_020957	-	4.54	10.10	-1.16	0.01
XLOC_025100	-	3.28	0.57	2.52	0.01
XLOC_025732	aspm	1.67	3.03	-0.86	0.01
XLOC_001331	polh	1.61	3.36	-1.06	0.01
XLOC_006565	fus	0.87	1.93	-1.14	0.01
XLOC_015526	msmo1	747.85	1802.74	-1.27	0.01
XLOC_003202	ENSONIG00000002356	4.95	2.41	1.04	0.01
XLOC_010327	ncs1a	3.74	1.61	1.21	0.01
XLOC_012601	esyt2a	6.81	3.85	0.82	0.01
XLOC_028023	-	5.52	11.40	-1.05	0.01
XLOC_029806	-	4.55	0	inf	0.01
XLOC_009020	ypel1	1.40	3.14	-1.16	0.01
XLOC_012406	-	0.11	1.78	-4.06	0.01
XLOC_018171	zgc:172352	2.63	4.75	-0.85	0.01
XLOC_021961	ENSONIG00000003715	3255.92	1342.82	1.28	0.01
XLOC_023153	ENSONIG00000000208	1.58	3.85	-1.29	0.01
XLOC_024536	-	1.66	0.28	2.56	0.01
XLOC_025564	cdk6	0.65	1.56	-1.25	0.01
XLOC_029311	cul1b	11.14	20.18	-0.86	0.01
XLOC_004808	jag1b	4.65	2.52	0.88	0.01
XLOC_006715	ARPC4 (1 of many)	24.90	45.43	-0.87	0.01
XLOC_024747	chchd1	5.85	3.03	0.95	0.01

XLOC_025865	col6a1	4.88	8.90	-0.87	0.01
XLOC_029911	-	6.11	0.40	3.95	0.01
XLOC_002924	nudcd3	10.20	4.92	1.05	0.01
XLOC_002951	-	5.83	1.18	2.31	0.01
XLOC_005000	zgc:153073	5.95	12.27	-1.05	0.01
XLOC_007982	ext1a	2.48	4.47	-0.85	0.01
XLOC_012245	mtss1la	2.45	4.68	-0.93	0.01
XLOC_013282	utp6	3.42	1.45	1.24	0.01
XLOC_017225	ufd1l	45.26	25.46	0.83	0.01
XLOC_024364	dcun1d5	7.08	3.41	1.06	0.01
XLOC_006578	nde1	0.80	0.19	2.07	0.01
XLOC_016567	rhbdl2	0.57	2.12	-1.89	0.01
XLOC_025113	acsl3a	0.22	0.82	-1.87	0.01
XLOC_025670	-	16.46	2.51	2.72	0.01
XLOC_009152	-	1.82	5.95	-1.70	0.01
XLOC_010847	ENSONIG00000014904	0.10	1.40	-3.87	0.01
XLOC_012036	mrps17	24.79	11.27	1.14	0.01
XLOC_012663	kif5bb	3.70	2.05	0.86	0.01
XLOC_019456	-	4.48	1.85	1.27	0.01
XLOC_031012	ENSONIG00000008308	117.18	62.95	0.90	0.01
XLOC_031265	ENSONIG00000011900	0.56	2.66	-2.24	0.01
XLOC_010213	grk5l	3.07	5.91	-0.94	0.01
XLOC_012823	-	5.31	1.47	1.85	0.01
XLOC_018559	si:dkey-82j4.2	1.86	0.81	1.20	0.01
XLOC_009791	ENSONIG00000002431	0.33	1.56	-2.25	0.01
XLOC_015561	lrba	2.25	4.00	-0.83	0.01
XLOC_017876	-	57.99	104.77	-0.85	0.01
XLOC_019390	-	0.44	3.87	-3.12	0.01
XLOC_028493	nol7	21.86	6.69	1.71	0.01
XLOC_002440	pabpc1l	2.52	0.09	4.79	0.01
XLOC_004836	)H18A1 (1 of many),HA	200.80	463.60	-1.21	0.01
XLOC_016288	ENSONIG00000006720	0.76	1.84	-1.28	0.01
XLOC_018332	anxa11a	4.34	7.94	-0.87	0.01
XLOC_022578	si:ch1073-44g3.1	0.95	3.20	-1.75	0.01
XLOC_028282	-	1.90	0.51	1.90	0.01
XLOC_008844	noc4l	4.82	2.11	1.19	0.01
XLOC_015969	-	0.27	1.83	-2.74	0.01
XLOC_019734	gskip	28.60	9.14	1.65	0.01
XLOC_021036	-	7.89	36.51	-2.21	0.01
XLOC_026275	arhgap6	0.29	0.91	-1.62	0.01
XLOC_027343	asnsd1	14.11	3.98	1.82	0.01
XLOC_003813	zgc:92275	8.80	15.47	-0.81	0.01
XLOC_004871	ikzf1	2.43	5.26	-1.11	0.01
XLOC_012301	si:ch211-195b13.1	1.25	2.62	-1.07	0.01
XLOC_014485	cbx3a	18.76	32.81	-0.81	0.01
XLOC_018758	ogfrl1	0.05	1.05	-4.28	0.01
XLOC_023109	aoc1	0.37	1.22	-1.74	0.01
XLOC_024865	-	2.08	7.87	-1.92	0.01

XLOC_003826	-	28.79	7.92	1.86	0.01
XLOC_012099	-	2.72	6.61	-1.28	0.01
XLOC_012624	eif3f	261.52	144.42	0.86	0.01
XLOC_013144	-	35.19	19.78	0.83	0.01
XLOC_015155	-	1.68	8.04	-2.26	0.01
XLOC_021765	MLLT6	1.26	0.63	1.00	0.01
XLOC_023077	ENSONIG00000014100	1.36	0.25	2.44	0.01
XLOC_029048	-	2.41	8.71	-1.85	0.01
XLOC_011136	zgc:162967	6.58	3.32	0.99	0.01
XLOC_019297	cox6b2 (1 of many)	4.48	1.94	1.21	0.01
XLOC_022465	dyrk1ab	5.97	3.36	0.83	0.01
XLOC_024342	RSF1	1.40	2.67	-0.93	0.01
XLOC_014330	fbxo7	2.93	6.38	-1.13	0.01
XLOC_025834	ENSONIG00000009129	1.68	13.33	-2.98	0.01
XLOC_026086	-	4.66	0.22	4.39	0.01
XLOC_030568	-	4.40	28.03	-2.67	0.01
XLOC_016555	ZC3H12A (1 of many)	0.43	1.19	-1.47	0.01
XLOC_016571	fabp10a	5658.07	18023.10	-1.67	0.01
XLOC_021893	ephx2	60.69	128.11	-1.08	0.01
XLOC_023455	mmgt1	27.13	15.27	0.83	0.01
XLOC_025873	mcm6	0.76	2.12	-1.48	0.01
XLOC_028367	pnpla7b	0.46	1.06	-1.20	0.01
XLOC_005320	-	33.98	0.37	6.54	0.01
XLOC_012742	mfap3l	7.72	4.23	0.87	0.01
XLOC_018681	glb1	2.34	4.57	-0.97	0.01
XLOC_019448	nup85	3.34	1.42	1.23	0.01
XLOC_021729	znf384l (1 of many)	1.43	2.64	-0.89	0.01
XLOC_031401	-	15.75	3.57	2.14	0.01
XLOC_003555	hsdl2	14.07	24.74	-0.81	0.01
XLOC_006930	ptbp1a	5.73	3.19	0.84	0.01
XLOC_015449	si:ch73-209e20.5	5.27	2.98	0.83	0.01
XLOC_016674	ENSONIG00000013103	9.46	4.75	0.99	0.01
XLOC_020601	orai2	0.52	1.34	-1.37	0.01
XLOC_027097	ENSONIG00000000206	11.21	5.81	0.95	0.01
XLOC_005002	mlh1	4.29	2.06	1.06	0.01
XLOC_010846	-	77.44	44.45	0.80	0.01
XLOC_015150	ENSONIG00000009357	2.33	8.66	-1.90	0.01
XLOC_015248	SUN1 (1 of many)	1.63	3.29	-1.01	0.01
XLOC_016701	josd2	7.47	3.97	0.91	0.01
XLOC_019451	zgc:161969	1.35	0.41	1.71	0.01
XLOC_020960	ypel3	26.73	72.69	-1.44	0.01
XLOC_023247	-	143.14	311.40	-1.12	0.01
XLOC_024937	h211-137i24.10 (1 of ma	31.91	70.47	-1.14	0.01
XLOC_001128	higd1a	81.42	24.17	1.75	0.01
XLOC_006716	si:dkey-202e22.2	1.40	0.59	1.26	0.01
XLOC_012252	mterf2	1.83	0.40	2.20	0.01
XLOC_013405	NAA15 (1 of many)	7.81	4.38	0.83	0.01
XLOC_015118	inppl1a	1.39	2.66	-0.94	0.01

XLOC_022947	cog3	4.87	2.47	0.98	0.01
XLOC_027117	ap1s2	2.20	4.87	-1.15	0.01
XLOC_002020	-	0.15	1.63	-3.41	0.01
XLOC_003349	-	20.99	11.31	0.89	0.01
XLOC_007242	tefb	3.25	1.58	1.04	0.01
XLOC_007421	-	1.96	5.19	-1.40	0.01
XLOC_007437	-	0.24	1.75	-2.84	0.01
XLOC_000003	-	3.84	0	inf	0.01
XLOC_016060	pycr1a	6.60	3.05	1.11	0.01
XLOC_016130	ENSONIG00000005727	1.50	2.83	-0.92	0.01
XLOC_017379	wdr89	2.36	0.65	1.85	0.01
XLOC_017503	-	1.75	0	inf	0.01
XLOC_017578	-	41.14	0	inf	0.01
XLOC_024382	ENSONIG00000001529	0.50	1.35	-1.44	0.01
XLOC_025975	-	5.17	0	inf	0.01
XLOC_027852	-	1.21	4.06	-1.75	0.01
XLOC_028040	-	13.21	0	inf	0.01
XLOC_029780	-	2.79	0	inf	0.01
XLOC_030198	-	2.37	0	inf	0.01
XLOC_030547	-	26.48	0	inf	0.01
XLOC_006343	itga2b	2.81	5.27	-0.91	0.01
XLOC_014611	SNORD15	0	6.22	-inf	0.01
XLOC_015178	-	16.34	5.31	1.62	0.01
XLOC_021402	-	3.93	11.73	-1.58	0.01
XLOC_024575	si:dkey-102c8.2	0.97	3.61	-1.89	0.01
XLOC_027240	phf21ab	0.60	1.66	-1.46	0.01
XLOC_031266	ENSONIG00000002864	0.54	4.39	-3.01	0.01
XLOC_005870	lfng	0.18	0.96	-2.40	0.01
XLOC_007587	qdpra	83.33	154.48	-0.89	0.01
XLOC_014827	SLC41A3 (1 of many)	2.76	1.08	1.35	0.01
XLOC_017519	stx6	8.08	4.09	0.98	0.01
XLOC_023005	ogdha	12.94	7.29	0.83	0.01
XLOC_026502	tsen34	7.39	3.67	1.01	0.01
XLOC_006540	-	29.62	15.93	0.89	0.01
XLOC_011943	gfra3	5.40	9.49	-0.81	0.01
XLOC_019284	-	0.55	8.58	-3.95	0.01
XLOC_020903	-	16.28	3.40	2.26	0.01
XLOC_022825	acap2	2.79	4.89	-0.81	0.01
XLOC_026823	p2rx1	2.16	0.70	1.62	0.01
XLOC_000922	plk3	3.48	1.66	1.07	0.01
XLOC_010471	nae1	5.96	3.17	0.91	0.01
XLOC_021229	-	1.64	0.57	1.52	0.01
XLOC_023840	nol6	3.05	1.47	1.05	0.01
XLOC_029667	ddx27	7.75	4.30	0.85	0.01
XLOC_001969	-	3.69	10.52	-1.51	0.01
XLOC_011645	cebpz	2.68	1.25	1.10	0.01
XLOC_013696	nfe2	0.61	2.14	-1.82	0.01
XLOC_014863	tns2a	9.13	5.23	0.80	0.01

XLOC_020173	-	3.01	0	inf	0.01
XLOC_024409	snrnp48	3.65	7.18	-0.98	0.01
XLOC_012507	-	3.67	8.27	-1.17	0.01
XLOC_014152	ogfod2	5.88	2.72	1.11	0.01
XLOC_025737	ttc14	5.65	10.42	-0.88	0.01
XLOC_000094	-	1.49	28.54	-4.26	0.01
XLOC_005219	25A34,TMEM82 (1 of m	12.97	7.34	0.82	0.01
XLOC_013072	fcf1	15.61	6.45	1.28	0.01
XLOC_018806	fkbp2	39.66	22.55	0.81	0.01
XLOC_022306	-	2.95	5.62	-0.93	0.01
XLOC_024761	-	7.04	0.50	3.83	0.01
XLOC_002115	nt5dc2	35.05	65.17	-0.89	0.01
XLOC_003016	-	21.03	11.64	0.85	0.01
XLOC_012754	ccs	8.01	15.93	-0.99	0.01
XLOC_025791	-	1.30	5.88	-2.18	0.01
XLOC_028728	mrpl52	13.63	6.30	1.11	0.01
XLOC_001712	-	54.36	25.91	1.07	0.01
XLOC_002570	scrib	4.80	9.10	-0.92	0.01
XLOC_013974	ENSONIG00000018526	311.80	1281.20	-2.04	0.01
XLOC_016173	cpne3 (1 of many)	2.58	4.83	-0.91	0.01
XLOC_023085	-	0.69	7.19	-3.37	0.01
XLOC_008754	ENSONIG00000003647	0.80	2.27	-1.50	0.01
XLOC_013045	-	0.56	3.67	-2.71	0.01
XLOC_017907	dnajc15	9.65	1.66	2.54	0.01
XLOC_030266	-	5.78	1.03	2.48	0.01
XLOC_005698	dnmbp	3.00	1.61	0.90	0.01
XLOC_008375	ndrg1a	26.09	70.41	-1.43	0.01
XLOC_010559	adam19b	0.65	1.65	-1.35	0.01
XLOC_010638	TSPAN17	12.70	5.58	1.19	0.01
XLOC_011796	SLC47A1 (1 of many)	0.06	1.27	-4.36	0.01
XLOC_012884	-	1.08	9.48	-3.13	0.01
XLOC_023918	-	6.42	14.10	-1.14	0.01
XLOC_025206	-	0.55	1.67	-1.61	0.01
XLOC_025217	-	1.42	0.17	3.10	0.01
XLOC_026085	dennd4b	2.99	5.17	-0.79	0.01
XLOC_006479	zgc:153733	0.20	2.29	-3.49	0.01
XLOC_007985	rspo3	0.21	1.95	-3.19	0.01
XLOC_015158	arhgef15	1.68	0.82	1.04	0.01
XLOC_017384	dtd2	3.04	1.31	1.21	0.01
XLOC_019353	gb:eh507706	1.30	2.65	-1.03	0.01
XLOC_022243	socs1a	3.75	7.37	-0.97	0.01
XLOC_027287	wsb1	59.18	32.83	0.85	0.01
XLOC_006530	ENSONIG00000009341	0.40	2.04	-2.35	0.01
XLOC_006618	-	0.57	2.01	-1.81	0.01
XLOC_019086	nup98	5.49	9.70	-0.82	0.01
XLOC_021231	mtp	43.12	23.60	0.87	0.01
XLOC_003590	-	0	2.21	-inf	0.01
XLOC_007053	dnajc5gb	9.88	5.49	0.85	0.01

XLOC_008410	adnp2b	6.85	3.00	1.19	0.01
XLOC_018493	ldlrad4b	1.81	0.83	1.13	0.01
XLOC_024989	-	1.41	0.29	2.29	0.01
XLOC_027705	-	0	5.37	-inf	0.01
XLOC_000907	-	0.28	3.93	-3.83	0.01
XLOC_002829	zgc:194879 (1 of many)	0.76	2.24	-1.56	0.01
XLOC_003290	clocka	0.21	0.83	-1.98	0.01
XLOC_003941	ENSONIG00000014774	1.62	2.99	-0.89	0.01
XLOC_005434	tsku	44.73	25.18	0.83	0.01
XLOC_009592	tspan33a	5.37	2.54	1.08	0.01
XLOC_010194	-	79.48	27.52	1.53	0.01
XLOC_013003	gchfr	331.63	175.04	0.92	0.01
XLOC_018611	ENSONIG00000021112	1.23	17.62	-3.84	0.01
XLOC_008299	ntn4	0.31	1.21	-1.98	0.01
XLOC_010981	SONIG00000015265,nm	18.32	34.83	-0.93	0.01
XLOC_011341	rad23aa	19.33	11.27	0.78	0.01
XLOC_030108	si:dkey-13n15.2	5.01	2.30	1.12	0.01
XLOC_030902	ENSONIG00000015362	0.37	6.03	-4.03	0.01
XLOC_000284	-	10.24	19.08	-0.90	0.01
XLOC_004511	ENSONIG00000001186	106.50	198.52	-0.90	0.01
XLOC_005054	RBM15 (1 of many)	1.31	0.62	1.08	0.01
XLOC_006124	supv3l1	2.37	0.84	1.50	0.01
XLOC_008000	-	13.47	3.93	1.78	0.01
XLOC_027753	zgc:153372	2.30	5.28	-1.20	0.01
XLOC_030522	-	7.89	1.55	2.35	0.01
XLOC_008026	-	1.35	5.28	-1.97	0.01
XLOC_014214	skap1	0.23	1.84	-3.02	0.01
XLOC_015373	NEK5	6.04	3.38	0.84	0.01
XLOC_001843	hs3st3l	2.09	0.82	1.35	0.01
XLOC_010383	ano5b	1.38	2.60	-0.91	0.01
XLOC_013596	fmnl3	3.55	7.11	-1.00	0.01
XLOC_015596	si:dkey-48p11.3	6.25	2.64	1.24	0.01
XLOC_023919	-	23.51	42.22	-0.84	0.01
XLOC_030736	nlrx1	10.51	5.77	0.87	0.01
XLOC_011272	-	22.30	12.62	0.82	0.01
XLOC_011290	rarab	1.66	3.18	-0.94	0.01
XLOC_012838	pcdh1b	20.76	11.91	0.80	0.01
XLOC_017543	npl	2.52	5.75	-1.19	0.01
XLOC_025858	OXSR1 (1 of many)	3.62	1.95	0.89	0.01
XLOC_001041	-	82.70	47.57	0.80	0.01
XLOC_002651	acad11	130.27	62.24	1.07	0.01
XLOC_015944	tnfsf14 (1 of many)	0.68	2.34	-1.79	0.01
XLOC_023042	mak16	9.64	5.23	0.88	0.01
XLOC_025036	cog1	7.13	4.04	0.82	0.01
XLOC_025561	tfpi2	4.58	8.73	-0.93	0.01
XLOC_026660	-	9.91	24.22	-1.29	0.01
XLOC_004651	poglut1	3.47	1.78	0.96	0.01
XLOC_014243	-	0.74	3.31	-2.16	0.01

XLOC_016769	SLC37A3	4.52	7.98	-0.82	0.01
XLOC_024347	-	2.70	0.65	2.05	0.01
XLOC_025723	-	0.19	1.39	-2.88	0.01
XLOC_007978	-	28.55	15.84	0.85	0.01
XLOC_008396	nfyc	11.05	19.43	-0.81	0.01
XLOC_009719	arl1	43.83	13.41	1.71	0.01
XLOC_009921	inppl1b	0.22	0.99	-2.18	0.01
XLOC_016796	-	3.01	5.98	-0.99	0.01
XLOC_018628	pds5a	5.23	2.92	0.84	0.01
XLOC_001967	-	4.26	23.46	-2.46	0.01
XLOC_002800	-	7.10	2.75	1.37	0.01
XLOC_004950	mcu	1.82	3.32	-0.87	0.01
XLOC_010502	ercc3	5.05	2.72	0.89	0.01
XLOC_014429	dusp16	11.25	6.45	0.80	0.01
XLOC_024458	ENSONIG00000011630	0.21	0.89	-2.07	0.01
XLOC_025779	-	0.67	5.31	-2.98	0.01
XLOC_002035	TSHZ2	0.84	0.22	1.91	0.01
XLOC_005780	ENSONIG00000016181	45.45	26.27	0.79	0.01
XLOC_016596	ttyh1	3.10	1.25	1.31	0.01
XLOC_018358	sema4ga	17.27	9.65	0.84	0.01
XLOC_021442	csnk2a1	31.38	18.19	0.79	0.01
XLOC_001568	mtrf1l	1.25	0.46	1.45	0.01
XLOC_009420	kazna (1 of many)	13.24	23.20	-0.81	0.01
XLOC_014531	oxnad1	3.77	1.90	0.99	0.01
XLOC_019314	si:ch211-171h4.3	2.42	4.70	-0.96	0.01
XLOC_008763	plgrkt	8.60	4.68	0.88	0.01
XLOC_010209	rorb	1.74	0.53	1.71	0.01
XLOC_016914	rhoub	17.33	9.90	0.81	0.01
XLOC_017083	cdh17	1.29	2.45	-0.93	0.01
XLOC_002241	-	6.09	24.79	-2.02	0.01
XLOC_008837	-	9.47	3.08	1.62	0.01
XLOC_009149	hs6st2	1.74	0.70	1.31	0.01
XLOC_012103	crebrf	0.18	0.87	-2.24	0.01
XLOC_016182	itgb1a	2.20	4.01	-0.87	0.01
XLOC_018437	-	2.56	0	inf	0.01
XLOC_020102	mfsd13a	7.24	3.73	0.96	0.01
XLOC_022097	ENSONIG00000003663	0.54	8.28	-3.95	0.01
XLOC_022362	nt5c2a	4.17	8.25	-0.98	0.01
XLOC_023309	-	8.93	0	inf	0.01
XLOC_024622	fam46c	2.36	0.39	2.60	0.01
XLOC_028292	-	10.91	1.38	2.99	0.01
XLOC_005057	ENSONIG00000018963	1.02	2.04	-1.00	0.01
XLOC_007968	ccr8.1	0.45	1.33	-1.56	0.01
XLOC_009525	si:dkey-222l13.1	2.92	1.60	0.87	0.01
XLOC_024100	oser1	14.38	7.74	0.89	0.01
XLOC_005114	-	7.66	14.39	-0.91	0.01
XLOC_002116	rap1gap	0.63	1.76	-1.49	0.01
XLOC_012072	-	5.38	0	inf	0.01

XLOC_021724	-	2.75	0	inf	0.01
XLOC_027516	zgc:110239	8.43	14.61	-0.79	0.01
XLOC_005639	hpdl	0.84	0.25	1.77	0.01
XLOC_013570	naa10	67.71	38.64	0.81	0.01
XLOC_020227	zgc:123105	43.01	76.55	-0.83	0.01
XLOC_030699	-	2.51	0.21	3.59	0.01
XLOC_002721	-	7.55	2.37	1.67	0.01
XLOC_020509	pkp1b	8.22	4.40	0.90	0.01
XLOC_030775	-	0.87	3.95	-2.18	0.01
XLOC_006417	ubald2	2.90	5.71	-0.98	0.01
XLOC_019487	-	1.47	4.89	-1.73	0.01
XLOC_004359	mut	44.88	23.38	0.94	0.01
XLOC_011447	fam120a	6.32	3.58	0.82	0.01
XLOC_016324	ENSONIG00000007617	1.38	0.61	1.17	0.01
XLOC_028402	-	0	13.01	-inf	0.01
XLOC_004014	blm	0.45	1.06	-1.24	0.01
XLOC_019022	icn	8.53	20.16	-1.24	0.01
XLOC_026625	MYO5B	0.78	0.34	1.17	0.01
XLOC_028024	-	1.27	3.91	-1.62	0.01
XLOC_009260	tmem115	3.25	1.84	0.82	0.01
XLOC_010971	zgc:103482	0.40	1.50	-1.90	0.01
XLOC_014811	ARHGAP4 (1 of many)	0.13	0.63	-2.24	0.01
XLOC_029564	ENSONIG00000007955	0.17	0.88	-2.40	0.01
XLOC_006018	-	6.89	12.52	-0.86	0.01
XLOC_007517	rabl3	17.06	8.77	0.96	0.01
XLOC_011953	CAPN6	1.62	0.53	1.62	0.01
XLOC_023071	-	6.15	3.28	0.91	0.01
XLOC_023961	vkorc1l1	12.36	4.59	1.43	0.01
XLOC_000613	-	8.53	3.93	1.12	0.01
XLOC_001984	-	2.87	16.33	-2.51	0.01
XLOC_002714	-	0.13	2.71	-4.40	0.01
XLOC_006059	dock6	6.49	12.66	-0.96	0.01
XLOC_026063	decr1	52.69	92.18	-0.81	0.01
XLOC_028347	lman2lb	18.66	10.93	0.77	0.01
XLOC_002380	UBA3 (1 of many)	12.47	7.05	0.82	0.01
XLOC_003920	polg	2.81	1.42	0.98	0.01
XLOC_009860	coq4	10.39	4.42	1.23	0.01
XLOC_012309	-	6.72	1.98	1.76	0.01
XLOC_020099	fam213ab	18.05	9.93	0.86	0.01
XLOC_020330	-	2517.34	626.50	2.01	0.01
XLOC_006084	xab2	4.34	2.39	0.86	0.01
XLOC_014643	ece2b	1.21	0.55	1.14	0.01
XLOC_021474	brd9	2.67	5.06	-0.92	0.01
XLOC_000321	ENSONIG00000014848	1.80	5.72	-1.67	0.01
XLOC_009056	-	0.84	2.66	-1.66	0.01
XLOC_012713	-	271.92	145.41	0.90	0.01
XLOC_015299	-	2.07	0.66	1.64	0.01
XLOC_026067	-	14.43	0	inf	0.01

XLOC_026105	-	3.20	25.24	-2.98	0.01
XLOC_006675	-	0.29	2.71	-3.24	0.01
XLOC_009230	ints5	2.66	1.33	1.00	0.01
XLOC_022610	ABP6 (1 of many),adra1b	4.85	23.23	-2.26	0.01
XLOC_023412	ddx26b	2.06	0.95	1.11	0.01
XLOC_011030	ldlrap1b	1.77	4.68	-1.40	0.01
XLOC_018106	-	0.57	2.42	-2.10	0.01
XLOC_028436	-	9.25	2.83	1.71	0.01
XLOC_001222	ARMC6	1.07	0.32	1.73	0.01
XLOC_003081	aebp1	0.88	1.88	-1.09	0.01
XLOC_007217	-	20.34	11.06	0.88	0.01
XLOC_016857	hsd17b12b	79.04	42.13	0.91	0.01
XLOC_019680	rpf2	3.51	1.94	0.86	0.01
XLOC_003659	ppp2r2ab	1.72	0.79	1.13	0.01
XLOC_004316	ARHGAP18	3.03	5.46	-0.85	0.01
XLOC_005810	fra10ac1	7.12	13.82	-0.96	0.01
XLOC_009040	GZMA (1 of many)	2.89	0.42	2.78	0.01
XLOC_022837	ENSONIG00000002698	1.36	4.91	-1.85	0.01
XLOC_012384	-	5.47	0.71	2.94	0.01
XLOC_012456	fastkd1	1.00	0.29	1.77	0.01
XLOC_014069	arvcfb	0.20	0.64	-1.72	0.01
XLOC_020592	-	7.64	3.17	1.27	0.01
XLOC_021626	-	0.38	2.02	-2.39	0.01
XLOC_027523	ENSONIG00000021006	540.33	198.16	1.45	0.01
XLOC_029968	ENSONIG00000008913	0.94	6.50	-2.79	0.01
XLOC_013481	-	27.17	8.02	1.76	0.01
XLOC_013536	cand1	6.01	3.43	0.81	0.01
XLOC_029452	-	13.19	0	inf	0.01
XLOC_006538	nupr1	504.77	251.29	1.01	0.01
XLOC_008417	uqcrb	181.69	96.65	0.91	0.01
XLOC_025890	plcd1b	2.08	0.68	1.61	0.01
XLOC_029419	ENSONIG00000004890	2.51	1.08	1.22	0.01
XLOC_010556	ebf1b	0.21	1.13	-2.44	0.01
XLOC_017654	ddx46	7.17	12.31	-0.78	0.01
XLOC_023027	-	0.96	0	inf	0.01
XLOC_022267	-	7.61	13.75	-0.85	0.01
XLOC_005269	ENSONIG00000019062	1.24	2.48	-1.00	0.01
XLOC_007991	mroh1	1.20	2.15	-0.84	0.01
XLOC_008696	scinla	0.47	1.48	-1.65	0.01
XLOC_011134	rdh14b	2.11	0.93	1.18	0.01
XLOC_013766	-	5.14	1.63	1.66	0.01
XLOC_005224	ssu72	21.22	12.34	0.78	0.01
XLOC_007241	zgc:162198	4.34	7.57	-0.80	0.01
XLOC_007498	-	1.16	3.43	-1.56	0.01
XLOC_010094	-	6.49	2.59	1.32	0.01
XLOC_010665	rab9b	13.89	5.64	1.30	0.01
XLOC_021374	-	4.54	2.09	1.12	0.01
XLOC_008492	nkain1	0.39	1.21	-1.63	0.01

XLOC_012464	ENSONIG00000008881	61.73	35.18	0.81	0.01
XLOC_014395	stil	0.76	2.76	-1.86	0.01
XLOC_020873	-	42.25	24.13	0.81	0.01
XLOC_025949	si:ch211-150i13.1	2.20	0.95	1.21	0.01
XLOC_029041	pla2g6	0.56	1.86	-1.74	0.01
XLOC_012912	dram2b	3.88	7.25	-0.90	0.01
XLOC_018892	ch211-152c8.5 (1 of man	11.81	5.52	1.10	0.01
XLOC_027920	eif3c	33.77	19.18	0.82	0.01
XLOC_031177	ENSONIG00000011081	0.79	4.62	-2.55	0.01
XLOC_010965	c7a	0.11	0.89	-3.05	0.01
XLOC_017890	lmo7a	9.96	5.69	0.81	0.01
XLOC_026967	-	7.63	3.19	1.26	0.01
XLOC_002042	cptp	1.63	0.46	1.82	0.01
XLOC_021097	npepps	5.71	3.21	0.83	0.01
XLOC_023871	-	0.68	4.82	-2.83	0.01
XLOC_024420	-	0.38	4.13	-3.46	0.01
XLOC_002094	gid8b	8.05	4.54	0.83	0.01
XLOC_007258	atf4b	32.31	18.37	0.81	0.01
XLOC_020815	-	1.50	2.94	-0.98	0.01
XLOC_025739	-	4.77	2.47	0.95	0.01
XLOC_000838	cx47.1	2.24	0.16	3.83	0.01
XLOC_001281	UQCRHL	114.53	47.47	1.27	0.01
XLOC_001283	-	0	10.53	-inf	0.01
XLOC_001673	-	0	2.18	-inf	0.01
XLOC_002022	-	0	2.29	-inf	0.01
XLOC_002387	-	0	3.96	-inf	0.01
XLOC_005467	-	0	3.90	-inf	0.01
XLOC_006458	-	0	25.08	-inf	0.01
XLOC_007150	-	0	12.28	-inf	0.01
XLOC_013874	-	0	6.92	-inf	0.01
XLOC_014147	gcn1	9.03	5.30	0.77	0.01
XLOC_014438	tnpo3	4.56	2.57	0.83	0.01
XLOC_014814	-	0	9.14	-inf	0.01
XLOC_016500	-	0	6.05	-inf	0.01
XLOC_017349	-	0	16.73	-inf	0.01
XLOC_018859	-	0	27.42	-inf	0.01
XLOC_022259	-	0	5.68	-inf	0.01
XLOC_024227	-	0	2.41	-inf	0.01
XLOC_024470	ENSONIG00000011683	1.96	3.79	-0.95	0.01
XLOC_024556	-	0	17.37	-inf	0.01
XLOC_025787	-	1.92	9.86	-2.36	0.01
XLOC_025885	-	1.67	3.77	-1.18	0.01
XLOC_026358	-	0	2.34	-inf	0.01
XLOC_026975	ARMC1 (1 of many)	4.54	2.51	0.86	0.01
XLOC_027148	-	0	5.57	-inf	0.01
XLOC_028039	-	0	11.89	-inf	0.01
XLOC_028269	-	0	4.69	-inf	0.01
XLOC_028543	-	0	40.39	-inf	0.01

XLOC_028614	-	0	13.10	-inf	0.01
XLOC_029643	-	0	3.51	-inf	0.01
XLOC_029759	-	0	18.04	-inf	0.01
XLOC_030208	-	0	25.08	-inf	0.01
XLOC_005118	LIMA1 (1 of many)	8.35	4.86	0.78	0.01
XLOC_007515	-	3.45	6.31	-0.87	0.01
XLOC_008043	si:dkey-218f9.10	5.25	2.79	0.91	0.01
XLOC_017337	-	5.17	13.58	-1.39	0.01
XLOC_017613	edem3	6.35	3.66	0.79	0.01
XLOC_002302	pou6f1	0.37	1.00	-1.43	0.01
XLOC_029463	-	17.39	9.97	0.80	0.01
XLOC_010353	golga3	3.57	2.07	0.79	0.01
XLOC_027090	mapk6	16.15	31.80	-0.98	0.01
XLOC_029563	-	168.59	13.12	3.68	0.01
XLOC_030355	nufip1	4.76	2.28	1.06	0.01
XLOC_012628	lig1	0.78	1.78	-1.19	0.01
XLOC_012984	pomt2	5.29	2.87	0.88	0.01
XLOC_015498	-	9.14	4.59	0.99	0.01
XLOC_006809	phf20a	3.03	1.64	0.89	0.01
XLOC_030150	-	51.37	1.96	4.71	0.01
XLOC_002591	-	0.99	2.59	-1.39	0.01
XLOC_022723	-	1.10	3.09	-1.50	0.01
XLOC_028915	ENSONIG00000013639	1.66	6.46	-1.96	0.01
XLOC_005976	eftud2,phospho1	10.39	18.41	-0.83	0.01
XLOC_007546	-	38.26	2.95	3.70	0.01
XLOC_009212	-	0.51	1.97	-1.94	0.01
XLOC_015399	-	11.10	22.02	-0.99	0.01
XLOC_026765	-	0.42	2.71	-2.69	0.01
XLOC_007866	-	283.47	152.06	0.90	0.01
XLOC_010047	inhbb	1.15	0.05	4.40	0.01
XLOC_013538	-	3.57	6.50	-0.86	0.01
XLOC_025064	crtc3	3.09	5.44	-0.81	0.01
XLOC_025264	-	0	5.95	-inf	0.01
XLOC_019636	-	2.38	4.86	-1.03	0.01
XLOC_023084	-	2.07	15.71	-2.92	0.01
XLOC_025714	-	6.77	20.20	-1.58	0.01
XLOC_003492	faslg	0.37	1.54	-2.07	0.01
XLOC_006313	adma	23.19	12.55	0.89	0.01
XLOC_008495	arpp21	0.27	0.94	-1.78	0.01
XLOC_015711	ckma	0.17	1.53	-3.16	0.01
XLOC_015749	-	0.37	2.72	-2.86	0.01
XLOC_002477	top1	0.38	1.24	-1.69	0.01
XLOC_005397	gosr1	10.45	6.07	0.78	0.01
XLOC_015862	vps37a	8.16	14.14	-0.79	0.01
XLOC_023381	-	2.55	0.83	1.61	0.01
XLOC_029354	-	36.41	5.01	2.86	0.01
XLOC_003443	ENSONIG00000002439	0.67	2.34	-1.81	0.01
XLOC_004597	-	2.55	0.16	3.98	0.01

XLOC_008691	sec16b	4.90	8.46	-0.79	0.01
XLOC_009070	etf1b	53.65	31.46	0.77	0.01
XLOC_009732	pde4ba	1.00	2.21	-1.15	0.01
XLOC_014566	gstr (1 of many)	70.14	125.51	-0.84	0.01
XLOC_028739	dhrs13a.3	4.02	7.48	-0.90	0.01
XLOC_008315	slc35b4	1.66	0.60	1.46	0.01
XLOC_010563	cpeb4 (1 of many)	1.17	0.53	1.15	0.01
XLOC_013137	ENSONIG00000012347	61.37	110.71	-0.85	0.01
XLOC_014558	FUCA1	12.67	21.62	-0.77	0.01
XLOC_019540	ankrd10b	3.80	7.95	-1.07	0.01
XLOC_020066	NASP	0.18	0.79	-2.17	0.01
XLOC_022583	-	1.40	3.79	-1.43	0.01
XLOC_004337	-	5.95	2.77	1.10	0.01
XLOC_013423	zgc:77739	5.02	8.83	-0.81	0.01
XLOC_015929	-	3.74	10.09	-1.43	0.01
XLOC_020582	tbcelb	7.22	12.34	-0.77	0.01
XLOC_024550	fadd	2.24	6.42	-1.52	0.01
XLOC_003583	-	4.23	1.41	1.59	0.01
XLOC_006052	hbae3 (1 of many)	0.19	2.91	-3.94	0.01
XLOC_011041	sdsl	21.68	12.31	0.82	0.01
XLOC_014707	-	15.92	8.00	0.99	0.01
XLOC_005818	SONIG00000016468,slc4	1.70	0.54	1.66	0.01
XLOC_009104	-	1.41	0.35	2.01	0.01
XLOC_011809	heatr6	3.67	2.01	0.87	0.01
XLOC_018380	-	31.33	16.93	0.89	0.01
XLOC_022765	-	6.37	2.82	1.17	0.01
XLOC_026987	si:ch1073-184j22.1	3.59	1.63	1.14	0.01
XLOC_007084	ptgr2	4.71	13.41	-1.51	0.01
XLOC_004107	arntl2 (1 of many)	1.06	2.10	-0.99	0.01
XLOC_004405	tcf21	0.73	2.27	-1.64	0.01
XLOC_012116	vtnb	110.33	221.64	-1.01	0.01
XLOC_007458	mras	1.35	2.66	-0.98	0.01
XLOC_005453	snrpd2	4.55	2.55	0.84	0.01
XLOC_001484	GTPBP2 (1 of many)	3.02	5.24	-0.80	0.01
XLOC_012457	atp5b (1 of many)	19.90	11.48	0.79	0.01
XLOC_013311	RHBDF2	0.36	1.01	-1.48	0.01
XLOC_018164	shmt2 (1 of many)	8.54	14.61	-0.77	0.01
XLOC_025778	-	4.34	14.70	-1.76	0.01
XLOC_007930	-	5.07	1.25	2.02	0.01
XLOC_009648	slco1d1	143.53	297.95	-1.05	0.01
XLOC_010331	slc15a4	1.13	2.35	-1.06	0.01
XLOC_010606	-	7.02	14.09	-1.01	0.01
XLOC_012991	ZFYVE1 (1 of many)	1.01	2.03	-1.01	0.01
XLOC_019545	-	3.91	14.82	-1.92	0.01
XLOC_003602	-	0.98	3.88	-1.99	0.01
XLOC_009600	phtf2	0.15	0.77	-2.37	0.01
XLOC_019004	TGFBR2 (1 of many)	4.26	7.87	-0.89	0.01
XLOC_008249	-	0.68	2.78	-2.03	0.01

XLOC_009471	ENSONIG00000002509	0.47	1.38	-1.54	0.01
XLOC_012137	anapc13	83.11	32.67	1.35	0.01
XLOC_017743	NUP54	5.73	3.14	0.87	0.01
XLOC_021233	ACER2	6.24	11.38	-0.87	0.01
XLOC_021267	si:dkey-6n6.2	0.37	1.96	-2.42	0.01
XLOC_000382	-	5.08	0.26	4.28	0.01
XLOC_005084	fkbp11	3.78	7.39	-0.97	0.01
XLOC_008036	-	3.34	1.53	1.12	0.01
XLOC_009574	-	3.34	9.43	-1.50	0.01
XLOC_016922	gpr184	0.38	2.06	-2.42	0.01
XLOC_031148	-	14.51	5.35	1.44	0.01
XLOC_000660	nkd1	0.37	0.88	-1.25	0.01
XLOC_008232	srpk2	0.66	2.67	-2.02	0.01
XLOC_011728	cox7a2a	155.44	92.25	0.75	0.01
XLOC_019097	-	42.68	25.17	0.76	0.01
XLOC_020384	-	2.90	7.49	-1.37	0.01
XLOC_021936	pnpla4	2.64	6.93	-1.39	0.01
XLOC_024441	-	28.16	1.34	4.40	0.01
XLOC_026241	ENSONIG00000000250	1.57	3.73	-1.25	0.01
XLOC_006516	-	163.87	32.80	2.32	0.01
XLOC_014654	-	0.65	1.80	-1.47	0.01
XLOC_025030	-	7.21	12.15	-0.75	0.01
XLOC_026775	psmb8a	0.43	4.10	-3.24	0.01
XLOC_028796	-	5.94	0	inf	0.01
XLOC_028836	-	9.70	0	inf	0.01
XLOC_030343	-	23.79	0	inf	0.01
XLOC_030512	-	8.32	0	inf	0.01
XLOC_003891	-	7.37	2.19	1.75	0.01
XLOC_015116	si:ch211-135f11.1	2.22	3.90	-0.81	0.01
XLOC_021478	nbeal2	0.52	0.98	-0.90	0.01
XLOC_025933	-	8.76	5.10	0.78	0.01
XLOC_007782	txndc5	40.03	22.59	0.83	0.01
XLOC_008774	snrpd3l	27.37	15.59	0.81	0.01
XLOC_019601	-	4.22	0.77	2.46	0.01
XLOC_029400	ap4m1	3.00	5.80	-0.95	0.01
XLOC_004993	fam160b1	5.95	3.51	0.76	0.01
XLOC_023246	b2m	684.30	1474.79	-1.11	0.01
XLOC_030523	-	30.07	17.67	0.77	0.01
XLOC_004586	ndfip2	5.91	10.27	-0.80	0.01
XLOC_013194	rhot1b	0.40	1.05	-1.38	0.01
XLOC_023126	-	14.54	41.30	-1.51	0.01
XLOC_030648	mtfmt	5.12	2.73	0.91	0.01
XLOC_004130	prr5a	5.43	9.44	-0.80	0.01
XLOC_009129	zgc:92249	9.75	18.49	-0.92	0.01
XLOC_021329	-	158.49	282.00	-0.83	0.01
XLOC_016005	cant1a	7.28	12.48	-0.78	0.01
XLOC_006203	eif4g2a	28.74	16.65	0.79	0.01
XLOC_007260	-	7.46	2.50	1.57	0.01

XLOC_007814	MCMDC2	12.99	5.10	1.35	0.01
XLOC_017293	-	0.21	1.39	-2.75	0.01
XLOC_022125	cnot10	7.72	4.29	0.85	0.01
XLOC_024104	-	47.62	21.85	1.12	0.01
XLOC_026847	ruvbl2	4.67	2.57	0.86	0.01
XLOC_002196	IFT122	1.26	2.83	-1.17	0.01
XLOC_008224	nuak1b	15.37	4.23	1.86	0.01
XLOC_015684	exoc3l2a	1.16	0.49	1.26	0.01
XLOC_016740	p3h3	4.89	8.53	-0.80	0.01
XLOC_006841	sfmbt1	12.86	7.65	0.75	0.01
XLOC_008859	-	47.24	27.42	0.78	0.01
XLOC_009208	tmx2a	9.92	4.64	1.10	0.01
XLOC_013276	-	2.21	4.89	-1.15	0.01
XLOC_024245	crk	6.60	3.76	0.81	0.01
XLOC_025450	-	1.63	5.80	-1.83	0.01
XLOC_027975	tox4b	3.54	1.82	0.96	0.01
XLOC_029078	ENSONIG00000016415	3.22	6.54	-1.02	0.01
XLOC_001757	AMZ2	3.52	6.64	-0.92	0.01
XLOC_006847	slc25a26	3.44	1.24	1.47	0.01
XLOC_018310	-	72.99	39.24	0.90	0.01
XLOC_022250	ENSONIG00000015736	0.74	2.21	-1.59	0.01
XLOC_025068	cdkn2aip	3.17	5.66	-0.84	0.01
XLOC_027408	ENSONIG00000010564	0.42	12.76	-4.94	0.01
XLOC_001774	zgc:91999	0.09	5.58	-5.93	0.01
XLOC_008349	ppm1h	1.11	2.24	-1.01	0.01
XLOC_002081	pym1	9.60	5.48	0.81	0.01
XLOC_011810	ddx52	3.92	1.93	1.02	0.01
XLOC_018984	MTERF1	1.85	0.42	2.12	0.01
XLOC_026142	tfe3a	7.96	13.40	-0.75	0.01
XLOC_004397	brox	12.59	7.39	0.77	0.01
XLOC_007385	ENSONIG00000008505	329.62	687.38	-1.06	0.01
XLOC_010125	TC2N	0.18	0.75	-2.08	0.01
XLOC_017309	-	0.58	4.28	-2.88	0.01
XLOC_015519	tma16	3.19	1.03	1.62	0.01
XLOC_015760	-	7.20	3.41	1.08	0.01
XLOC_017865	kpna3	1.14	0.28	2.04	0.01
XLOC_023906	zgc:92511,zgc:92745	1246.83	5366.43	-2.11	0.01
XLOC_030288	-	1.14	3.78	-1.73	0.01
XLOC_010746	ndrg3b	1.59	0.70	1.18	0.01
XLOC_013588	acvr1bb	2.93	1.16	1.33	0.01
XLOC_023903	-	7.89	14.64	-0.89	0.01
XLOC_025940	-	3.52	1.89	0.90	0.01
XLOC_028869	GUCY2D (1 of many)	0.72	0.27	1.43	0.01
XLOC_011049	-	51.08	8.87	2.53	0.01
XLOC_022146	preb	24.24	14.46	0.75	0.01
XLOC_022592	adra2da	0.42	2.00	-2.25	0.01
XLOC_000851	pcmtd1	1.62	3.10	-0.94	0.01
XLOC_004495	cyp46a1.3	8.53	14.87	-0.80	0.01

XLOC_015840	ENSONIG00000013592	12.05	32.32	-1.42	0.01
XLOC_003343	fbn2b	0.32	0.65	-1.00	0.01
XLOC_012431	prkra	3.96	1.77	1.16	0.01
XLOC_014073	si:ch1073-179p4.3	4.22	2.40	0.81	0.01
XLOC_021029	ENSONIG00000007251	23.78	13.13	0.86	0.01
XLOC_000803	-	0	16.24	-inf	0.01
XLOC_002889	-	0	19.04	-inf	0.01
XLOC_010459	mcee	15.76	35.38	-1.17	0.01
XLOC_014047	-	0	3.35	-inf	0.01
XLOC_014580	-	0	3.16	-inf	0.01
XLOC_021389	mpped2a	5.32	1.53	1.80	0.01
XLOC_025482	-	0	2.58	-inf	0.01
XLOC_029696	-	0	17.56	-inf	0.01
XLOC_001013	MED26	2.61	1.42	0.88	0.01
XLOC_002307	si:dkey-190g6.2	2.46	1.18	1.07	0.01
XLOC_019063	GADD45B (1 of many)	56.93	31.19	0.87	0.01
XLOC_022246	-	1.25	3.03	-1.28	0.01
XLOC_028241	lrch4	1.90	3.41	-0.85	0.01
XLOC_031115	-	0.14	3.65	-4.74	0.01
XLOC_007089	-	0.50	3.36	-2.75	0.01
XLOC_007119	ISYNA1	0.81	1.92	-1.24	0.01
XLOC_013391	p4hb	913.29	420.12	1.12	0.01
XLOC_016298	mfsd8	7.71	13.14	-0.77	0.01
XLOC_018082	-	62.84	106.79	-0.77	0.01
XLOC_026104	-	0.45	7.18	-4.01	0.01
XLOC_004781	golga4	4.12	2.43	0.76	0.01
XLOC_003937	-	31.63	16.81	0.91	0.01
XLOC_005005	dnajc12	5.68	2.91	0.97	0.01
XLOC_005621	-	5.34	1.86	1.52	0.01
XLOC_012861	thoc3	5.13	2.39	1.10	0.01
XLOC_015051	tagln	8.91	15.85	-0.83	0.01
XLOC_030177	eed	5.00	2.68	0.90	0.01
XLOC_001715	gspt1l (1 of many)	63.07	36.49	0.79	0.01
XLOC_018773	-	5.16	1.96	1.40	0.01
XLOC_006890	dusp23a	15.42	1.88	3.04	0.01
XLOC_011145	hsp90ab1	980.97	403.08	1.28	0.01
XLOC_013050	-	1.29	3.39	-1.39	0.01
XLOC_015851	nansa (1 of many)	20.46	11.43	0.84	0.01
XLOC_024523	pmm2	66.79	37.57	0.83	0.01
XLOC_001710	-	3.51	1.52	1.21	0.01
XLOC_004930	kcnk1b	1.80	3.59	-1.00	0.01
XLOC_007799	urad	21.16	48.22	-1.19	0.01
XLOC_015795	-	17.02	9.74	0.81	0.01
XLOC_016497	-	1211.47	2741.65	-1.18	0.01
XLOC_017906	znf385b	0.87	3.40	-1.96	0.01
XLOC_019155	sirt3	1.14	2.66	-1.22	0.01
XLOC_029875	-	53.57	7.44	2.85	0.01
XLOC_002053	si:dkey-205h13.1	23.48	5.44	2.11	0.01

XLOC_004643	abcb11b	14.07	25.69	-0.87	0.01
XLOC_008581	tmtops2a	32.42	17.88	0.86	0.01
XLOC_029197	-	2.41	4.99	-1.05	0.01
XLOC_007942	derl1	139.54	63.82	1.13	0.01
XLOC_026993	-	7.73	20.84	-1.43	0.01
XLOC_009266	xpc	1.39	0.69	1.01	0.01
XLOC_014381	ENSONIG00000007712	3250.91	8579.19	-1.40	0.01
XLOC_018719	-	1.69	0.71	1.26	0.01
XLOC_004212	neil1	3.15	7.34	-1.22	0.01
XLOC_012066	-	16.26	32.72	-1.01	0.01
XLOC_021201	aptx	10.99	1.75	2.65	0.01
XLOC_024439	-	4.32	10.58	-1.29	0.01
XLOC_004685	C1QL2 (1 of many)	0.39	1.14	-1.54	0.01
XLOC_029435	mycb	4.26	8.57	-1.01	0.01
XLOC_005794	fkbp10b	0.15	2.97	-4.33	0.01
XLOC_022078	stx1a	6.98	12.01	-0.78	0.01
XLOC_011169	RHOB	120.82	53.33	1.18	0.01
XLOC_011271	CSNK1D (1 of many)	5.41	2.78	0.96	0.01
XLOC_013710	tead3a	3.31	1.54	1.11	0.01
XLOC_015417	-	0.48	1.83	-1.94	0.01
XLOC_018476	lars2	3.31	1.82	0.87	0.01
XLOC_020441	-	5.11	10.51	-1.04	0.01
XLOC_027549	gtf2h4	4.18	7.37	-0.82	0.01
XLOC_006050	nprl3	6.77	11.74	-0.79	0.01
XLOC_011494	-	1.50	0.49	1.63	0.01
XLOC_013912	ccdc51	1.61	0.74	1.12	0.01
XLOC_016609	isoc2	29.03	53.63	-0.89	0.01
XLOC_003468	-	6.18	21.08	-1.77	0.02
XLOC_007001	hs6st1b	2.22	0.66	1.75	0.02
XLOC_008323	yaf2 (1 of many)	5.91	2.94	1.01	0.02
XLOC_008463	-	3.79	9.82	-1.37	0.02
XLOC_014272	arf2b	39.46	23.20	0.77	0.02
XLOC_018827	ap1m3	0.53	2.04	-1.95	0.02
XLOC_002987	si:ch1073-174d20.2	12.90	7.65	0.75	0.02
XLOC_026763	-	4.43	27.15	-2.62	0.02
XLOC_003216	rfxank	3.10	6.30	-1.02	0.02
XLOC_007233	pick1	3.43	1.69	1.02	0.02
XLOC_009634	USP15	6.60	3.64	0.86	0.02
XLOC_012848	PAIP2	9.60	16.47	-0.78	0.02
XLOC_014284	armc7	1.97	0.41	2.27	0.02
XLOC_023816	hsf2	6.21	3.56	0.80	0.02
XLOC_028351	imp4	9.29	4.99	0.90	0.02
XLOC_002907	zcchc9	8.17	4.62	0.82	0.02
XLOC_008218	-	4.00	1.92	1.06	0.02
XLOC_009281	-	5.20	0.84	2.63	0.02
XLOC_009813	xpnpep2	1.01	0.41	1.32	0.02
XLOC_025932	-	3.48	1.05	1.73	0.02
XLOC_028742	-	6.50	0.05	6.96	0.02

XLOC_005328	si:dkey-85p17.3	0.80	2.31	-1.52	0.02
XLOC_012814	actn3a	0.22	2.06	-3.21	0.02
XLOC_016360	kirrela	0.26	0.94	-1.87	0.02
XLOC_030711	-	12.90	6.00	1.11	0.02
XLOC_004003	fam174b	2.12	4.23	-1.00	0.02
XLOC_004754	agpat5	12.80	7.69	0.73	0.02
XLOC_005280	gpr84 (1 of many)	1.95	4.59	-1.24	0.02
XLOC_015477	fbp1b	554.75	293.59	0.92	0.02
XLOC_026766	-	0.37	4.83	-3.71	0.02
XLOC_000217	-	2.28	33.58	-3.88	0.02
XLOC_005455	ENSONIG00000005419	3.80	1.00	1.93	0.02
XLOC_008185	-	6.14	20.55	-1.74	0.02
XLOC_013664	ppard	4.63	7.72	-0.74	0.02
XLOC_014176	-	0.67	4.74	-2.82	0.02
XLOC_018435	-	3.11	0.84	1.89	0.02
XLOC_003353	use1	5.52	3.02	0.87	0.02
XLOC_005508	dus4l	3.59	1.62	1.15	0.02
XLOC_005517	-	0.81	3.45	-2.08	0.02
XLOC_013414	PRF1 (1 of many)	0.18	1.68	-3.18	0.02
XLOC_024054	vrk1	1.69	3.33	-0.97	0.02
XLOC_001067	PFN2 (1 of many)	12.17	4.93	1.30	0.02
XLOC_006206	sbf2	1.58	2.72	-0.79	0.02
XLOC_001492	SLK (1 of many)	1.19	2.15	-0.86	0.02
XLOC_016424	vps51	4.56	2.61	0.81	0.02
XLOC_022989	srp19	260.97	146.18	0.84	0.02
XLOC_004981	tomm20a	23.98	11.29	1.09	0.02
XLOC_013955	-	17.43	9.05	0.94	0.02
XLOC_016367	ch211-15b10.6 (1 of mar	8.14	16.16	-0.99	0.02
XLOC_010917	znf366	1.26	2.50	-0.98	0.02
XLOC_019285	ENSONIG00000006971	0	1.16	-inf	0.02
XLOC_027153	ENSONIG00000014688	3.50	6.47	-0.89	0.02
XLOC_014878	HDAC7 (1 of many)	1.08	1.94	-0.84	0.02
XLOC_015106	LSAMP	0.56	1.29	-1.19	0.02
XLOC_022026	ndrg2	0.78	2.22	-1.51	0.02
XLOC_029346	-	19.03	9.97	0.93	0.02
XLOC_000444	-	8.13	46.40	-2.51	0.02
XLOC_009453	plekhn1	1.73	0.79	1.13	0.02
XLOC_010934	ndc80	0.18	0.83	-2.25	0.02
XLOC_017470	-	5.28	2.53	1.06	0.02
XLOC_006026	-	5.15	0.90	2.51	0.02
XLOC_026260	TSEN2	1.98	4.43	-1.16	0.02
XLOC_009357	slc25a33	71.79	37.28	0.95	0.02
XLOC_008931	ptgesl	5.23	2.92	0.84	0.02
XLOC_014568	gstr (1 of many)	2.35	6.62	-1.49	0.02
XLOC_003277	cep19	3.33	0.48	2.81	0.02
XLOC_003281	impact	9.45	5.24	0.85	0.02
XLOC_017888	cln5	7.03	3.63	0.95	0.02
XLOC_001105	si:ch73-52e5.1	12.23	6.98	0.81	0.02

XLOC_012873	ENSONIG00000004373	7.93	21.34	-1.43	0.02
XLOC_013820	c1qa	23.40	39.36	-0.75	0.02
XLOC_025545	-	0.90	3.83	-2.09	0.02
XLOC_015255	LECT2 (1 of many)	5745.55	13424.50	-1.22	0.02
XLOC_005728	camk2g1	6.63	11.12	-0.75	0.02
XLOC_006366	-	3.91	2.02	0.95	0.02
XLOC_010397	ist1	4.23	7.27	-0.78	0.02
XLOC_001543	tmem26a (1 of many)	2.39	4.55	-0.93	0.02
XLOC_023817	cx43	2.90	1.39	1.06	0.02
XLOC_025289	fitm2	4.31	8.14	-0.92	0.02
XLOC_001966	-	6.09	13.82	-1.18	0.02
XLOC_000693	mrpl46	2.31	1.04	1.15	0.02
XLOC_001586	pno1	281.49	160.84	0.81	0.02
XLOC_017658	TXNDC15	33.70	18.67	0.85	0.02
XLOC_017813	esm1	6.13	2.30	1.42	0.02
XLOC_001954	ENSONIG00000019914	0.42	0.89	-1.08	0.02
XLOC_023654	-	0.19	1.57	-3.08	0.02
XLOC_029164	arpc5a	14.85	24.73	-0.74	0.02
XLOC_030331	-	34.82	13.09	1.41	0.02
XLOC_015053	SIK3	19.46	11.42	0.77	0.02
XLOC_020660	micall1a	0.64	1.39	-1.11	0.02
XLOC_023317	SLC30A6	8.72	4.90	0.83	0.02
XLOC_003970	ENSONIG00000014914	7.37	15.35	-1.06	0.02
XLOC_005743	ENSONIG00000015909	5.28	8.79	-0.74	0.02
XLOC_022113	neu4	0.79	0.18	2.10	0.02
XLOC_026320	-	107.19	210.55	-0.97	0.02
XLOC_002309	cd63	11.25	19.01	-0.76	0.02
XLOC_007728	rdh10a	0.37	1.59	-2.10	0.02
XLOC_016544	scmh1	13.31	8.00	0.73	0.02
XLOC_022600	-	1.37	25.19	-4.20	0.02
XLOC_025560	zgc:85777	56.16	15.68	1.84	0.02
XLOC_013566	zgc:66440	2.56	4.46	-0.80	0.02
XLOC_023380	-	7.27	2.56	1.51	0.02
XLOC_025928	-	0.49	1.87	-1.94	0.02
XLOC_004121	ergic2	26.79	15.61	0.78	0.02
XLOC_007920	-	0.38	1.76	-2.20	0.02
XLOC_029709	si:ch211-212k18.9	3.78	8.44	-1.16	0.02
XLOC_020802	morn3	0.24	2.39	-3.31	0.02
XLOC_004414	si:ch73-217b7.1	0.96	0.22	2.10	0.02
XLOC_004620	cpb2	180.07	343.55	-0.93	0.02
XLOC_005440	fam118b	52.45	22.79	1.20	0.02
XLOC_008037	-	9.03	4.00	1.18	0.02
XLOC_008202	golt1ba	4.58	1.32	1.79	0.02
XLOC_014727	r3hdm4 (1 of many)	11.67	6.84	0.77	0.02
XLOC_002709	acbd5a	21.36	35.44	-0.73	0.02
XLOC_008064	zgc:91944	0.88	2.24	-1.35	0.02
XLOC_013617	ccdc115	3.89	1.97	0.98	0.02
XLOC_028176	-	1.18	29.62	-4.65	0.02

XLOC_002470	b4galt5	0.87	1.70	-0.97	0.02
XLOC_018351	slc25a16	2.03	0.97	1.06	0.02
XLOC_029327	-	3.14	1.12	1.49	0.02
XLOC_001613	cycsb (1 of many)	24.77	13.51	0.87	0.02
XLOC_003256	ENSONIG00000002748	240.18	481.77	-1.00	0.02
XLOC_022147	cnih4	11.72	6.94	0.76	0.02
XLOC_029493	ENSONIG00000019954	0.30	1.03	-1.80	0.02
XLOC_030419	ENSONIG00000009121	4.66	9.01	-0.95	0.02
XLOC_010849	ENSONIG00000014908	0.16	1.01	-2.66	0.02
XLOC_004726	npm3	6.23	2.69	1.21	0.02
XLOC_017472	-	0.87	4.00	-2.20	0.02
XLOC_022009	-	0.31	1.35	-2.12	0.02
XLOC_000745	-	3.21	0.85	1.92	0.02
XLOC_002135	-	1.76	5.04	-1.52	0.02
XLOC_003401	-	7.34	0.81	3.17	0.02
XLOC_007284	rab5ab	9.18	4.59	1.00	0.02
XLOC_013735	utp3	1.98	0.73	1.44	0.02
XLOC_014723	-	1.15	4.83	-2.07	0.02
XLOC_002494	nktr	25.00	60.27	-1.27	0.02
XLOC_012490	igf2bp2a	0.79	0.23	1.79	0.02
XLOC_014004	-	9.79	2.92	1.75	0.02
XLOC_014944	wasla	0.78	1.62	-1.06	0.02
XLOC_017432	thbs3a	0.63	1.38	-1.13	0.02
XLOC_025221	-	0.56	1.93	-1.77	0.02
XLOC_025963	dnph1	3.74	8.09	-1.11	0.02
XLOC_028753	-	1.99	6.32	-1.67	0.02
XLOC_005099	larp4ab	2.07	0.91	1.19	0.02
XLOC_012263	DUSP8	5.27	2.97	0.83	0.02
XLOC_023175	rpl7l1	9.11	5.25	0.80	0.02
XLOC_029559	-	2.01	6.59	-1.71	0.02
XLOC_004068	PNPLA2	37.34	20.65	0.85	0.02
XLOC_013919	cyc1	19.62	11.89	0.72	0.02
XLOC_015782	si:ch211-212g7.6	4.50	7.85	-0.80	0.02
XLOC_017021	ENSONIG00000006319	2.59	0.57	2.18	0.02
XLOC_023201	ENSONIG00000015366	0.10	0.82	-2.96	0.02
XLOC_000391	-	15.98	0	inf	0.02
XLOC_005685	ghdc	3.95	2.21	0.84	0.02
XLOC_008283	si:dkey-29p10.4	6.67	11.37	-0.77	0.02
XLOC_014889	-	0.43	2.88	-2.75	0.02
XLOC_014995	dusp6	3.05	1.59	0.94	0.02
XLOC_018149	ptcd3	7.11	2.96	1.26	0.02
XLOC_021081	gtf3aa	3.22	0.84	1.94	0.02
XLOC_022986	FASTKD5	1.03	0.34	1.60	0.02
XLOC_002304	-	7.94	4.07	0.96	0.02
XLOC_007434	TMEM50B	3.90	14.15	-1.86	0.02
XLOC_017355	brf1a	1.18	0.26	2.18	0.02
XLOC_029088	RIDA (1 of many)	45.45	78.42	-0.79	0.02
XLOC_002073	rnf41	7.39	4.32	0.77	0.02

XLOC_006234	mpc1	84.47	49.55	0.77	0.02
XLOC_011491	-	12.03	6.49	0.89	0.02
XLOC_012018	ubtd2	1.74	4.60	-1.40	0.02
XLOC_012803	ENSONIG00000012993	0.42	1.59	-1.91	0.02
XLOC_016815	tfec	1.28	2.72	-1.08	0.02
XLOC_019014	cers2a	69.79	122.22	-0.81	0.02
XLOC_020422	pbdc1	40.56	22.83	0.83	0.02
XLOC_023447	sgsh	10.68	18.40	-0.79	0.02
XLOC_002397	DLGAP4 (1 of many)	1.21	0.41	1.58	0.02
XLOC_004876	cdc42ep3	4.01	1.73	1.21	0.02
XLOC_015689	hnrpl	6.72	3.86	0.80	0.02
XLOC_016884	bbox1	1.73	0.65	1.41	0.02
XLOC_003454	si:ch211-125m10.6	9.27	17.34	-0.90	0.02
XLOC_016969	laynb	0.51	1.79	-1.81	0.02
XLOC_000599	CCDC102A	0.42	1.23	-1.56	0.02
XLOC_002978	-	1.86	0.28	2.71	0.02
XLOC_005682	med1	3.34	1.96	0.77	0.02
XLOC_010788	-	0	3.30	-inf	0.02
XLOC_018684	ENSONIG00000017573	0.25	1.82	-2.85	0.02
XLOC_031409	-	5.14	2.27	1.18	0.02
XLOC_020664	nfil3-5	2.07	4.02	-0.96	0.02
XLOC_007767	pex2	4.44	1.71	1.37	0.02
XLOC_012614	-	2.77	0.55	2.32	0.02
XLOC_020913	fam173a	2.02	0.90	1.16	0.02
XLOC_023555	ENSONIG00000009619	1.20	2.62	-1.13	0.02
XLOC_026440	si:zfos-1962a1.5	623.62	231.91	1.43	0.02
XLOC_003829	-	0	28.82	-inf	0.02
XLOC_018445	-	101.22	61.58	0.72	0.02
XLOC_022292	-	0	658.36	-inf	0.02
XLOC_022640	csf1ra	4.25	7.09	-0.74	0.02
XLOC_027511	-	1.54	6.24	-2.02	0.02
XLOC_002377	ENSONIG00000016933	116.75	62.92	0.89	0.02
XLOC_002528	nom1	3.72	1.97	0.91	0.02
XLOC_020129	nat9	2.57	0.74	1.80	0.02
XLOC_026581	-	9.59	35.48	-1.89	0.02
XLOC_008734	aplnrb	5.41	10.08	-0.90	0.02
XLOC_015790	armc2	9.98	26.35	-1.40	0.02
XLOC_028440	-	11.26	1.39	3.02	0.02
XLOC_024145	MYNN	3.40	1.95	0.80	0.02
XLOC_030244	-	114.64	63.90	0.84	0.02
XLOC_004574	fdx1	36.46	21.67	0.75	0.02
XLOC_007850	NDUFB4	96.99	58.74	0.72	0.02
XLOC_014861	-	9.08	3.72	1.29	0.02
XLOC_017478	pik3r4	4.99	2.99	0.74	0.02
XLOC_018640	ucp1	245.55	118.27	1.05	0.02
XLOC_026734	-	0.49	3.43	-2.82	0.02
XLOC_002548	sox17	1.25	2.84	-1.19	0.02
XLOC_004463	-	9.69	2.54	1.93	0.02

XLOC_028454	-	32.40	8.48	1.93	0.02
XLOC_031079	-	0	14.89	-inf	0.02
XLOC_000478	ddx28	1.86	0.62	1.59	0.02
XLOC_020199	clip2	5.19	2.95	0.82	0.02
XLOC_026917	-	659.58	1100.02	-0.74	0.02
XLOC_000308	ENSONIG00000006695	1.19	8.49	-2.84	0.02
XLOC_022271	-	53.57	15.79	1.76	0.02
XLOC_002339	ENSONIG00000021138	9.75	5.57	0.81	0.02
XLOC_012049	pdlim4	4.42	7.45	-0.75	0.02
XLOC_016528	-	12.27	6.90	0.83	0.02
XLOC_022225	-	0.98	0.15	2.67	0.02
XLOC_011344	gadd45gip1	13.56	6.86	0.98	0.02
XLOC_026667	-	12.40	1.49	3.05	0.02
XLOC_003211	midn	3.19	1.84	0.79	0.02
XLOC_005420	naalad2	1.18	0.37	1.67	0.02
XLOC_007064	stx11a (1 of many)	0.47	1.80	-1.94	0.02
XLOC_013636	rbm10	6.94	11.51	-0.73	0.02
XLOC_003464	ubxn6	3.01	5.11	-0.76	0.02
XLOC_003934	polr3b	5.54	3.23	0.78	0.02
XLOC_011021	zgc:154055	0.51	1.33	-1.40	0.02
XLOC_015597	scube1	0.84	0.41	1.03	0.02
XLOC_022609	ppp2ca	1.27	3.22	-1.34	0.02
XLOC_031281	-	0.51	7.61	-3.91	0.02
XLOC_006172	si:ch211-94n14.3	3.03	1.34	1.18	0.02
XLOC_010545	-	51.04	30.81	0.73	0.02
XLOC_013797	ENSONIG00000004228	0.85	1.90	-1.15	0.02
XLOC_014865	krt18	204.96	112.64	0.86	0.02
XLOC_015991	-	0.48	1.35	-1.50	0.02
XLOC_021767	-	31.82	7.13	2.16	0.02
XLOC_000642	ube2q1	6.06	3.65	0.73	0.02
XLOC_020043	ENSONIG00000018703	2.25	4.07	-0.86	0.02
XLOC_029848	-	2.95	7.01	-1.25	0.02
XLOC_030210	ENSONIG00000004512	0.79	2.66	-1.75	0.02
XLOC_001391	-	2.14	0.41	2.38	0.02
XLOC_003300	fryl	9.13	16.04	-0.81	0.02
XLOC_007029	exd2	2.19	0.99	1.14	0.02
XLOC_008303	camk1db	0.35	1.51	-2.12	0.02
XLOC_019687	srp9	63.64	29.66	1.10	0.02
XLOC_021562	urb2	0.89	0.36	1.31	0.02
XLOC_000548	-	12.27	20.13	-0.71	0.02
XLOC_014097	rchy1	5.74	3.26	0.82	0.02
XLOC_016600	dbpb	5.15	3.00	0.78	0.02
XLOC_012077	fhl1b	2.81	5.55	-0.98	0.02
XLOC_013333	trim25 (1 of many)	5.46	9.11	-0.74	0.02
XLOC_022059	smc4	0.86	1.69	-0.97	0.02
XLOC_025898	casp8,catip	26.01	14.88	0.81	0.02
XLOC_026330	-	0	9.26	-inf	0.02
XLOC_029032	ENSONIG00000013088	4.84	9.59	-0.98	0.02

XLOC_001042	sept2	6.07	10.87	-0.84	0.02
XLOC_005473	gmps	9.01	5.43	0.73	0.02
XLOC_012931	ece1	9.25	15.58	-0.75	0.02
XLOC_015006	deaf1	1.68	0.77	1.12	0.02
XLOC_020757	myo1hb	10.25	6.06	0.76	0.02
XLOC_008159	fibina	0.79	3.96	-2.33	0.02
XLOC_000304	-	0	8.33	-inf	0.02
XLOC_016100	urb1	1.50	0.45	1.73	0.02
XLOC_025635	-	2.01	5.01	-1.32	0.02
XLOC_026945	ENSONIG00000001011	0.30	2.65	-3.17	0.02
XLOC_013113	-	0.87	26.01	-4.91	0.02
XLOC_027029	-	13.18	7.89	0.74	0.02
XLOC_002617	phex	0.88	0.27	1.70	0.02
XLOC_007055	cenpo	0.31	1.24	-2.01	0.02
XLOC_012985	-	3.70	8.81	-1.25	0.02
XLOC_013366	-	0.36	7.20	-4.33	0.02
XLOC_016155	cog6	11.81	6.53	0.86	0.02
XLOC_024451	ednrab	1.20	0.47	1.37	0.02
XLOC_025918	acss1	0.31	1.40	-2.20	0.02
XLOC_026078	-	1.93	0.59	1.72	0.02
XLOC_026214	-	109.06	430.19	-1.98	0.02
XLOC_005466	-	4.33	8.53	-0.98	0.02
XLOC_017509	tnfb	0.18	1.66	-3.19	0.02
XLOC_021935	-	115.06	25.82	2.16	0.02
XLOC_030626	-	0.36	1.57	-2.14	0.02
XLOC_010442	psmd14	20.44	12.42	0.72	0.02
XLOC_011417	nfixb	3.69	1.98	0.90	0.02
XLOC_016657	:ch73-86n18.1 (1 of man	459.01	195.20	1.23	0.02
XLOC_019300	-	1.81	0.21	3.10	0.02
XLOC_031317	-	0	26.73	-inf	0.02
XLOC_011335	carhsp1	11.46	19.55	-0.77	0.02
XLOC_018005	mthfd2	3.82	7.98	-1.06	0.02
XLOC_019672	nt5dc1	1.29	0.50	1.36	0.02
XLOC_001279	mrpl34	4.57	2.31	0.98	0.02
XLOC_024768	-	4.18	0.07	5.99	0.02
XLOC_024780	-	5.71	0.77	2.89	0.02
XLOC_001502	zranb1b	1.02	1.94	-0.93	0.02
XLOC_001950	ace	1.19	0.56	1.08	0.02
XLOC_009313	-	5.98	3.01	0.99	0.02
XLOC_019497	aimp2	96.43	58.04	0.73	0.02
XLOC_000210	-	15.85	29.06	-0.87	0.02
XLOC_002992	MLKL (1 of many)	0.32	1.00	-1.64	0.02
XLOC_006176	-	4.63	1.50	1.63	0.02
XLOC_016157	rcbtb2	3.32	5.75	-0.79	0.02
XLOC_022270	-	28.78	8.16	1.82	0.02
XLOC_002973	ENSONIG00000010702	10.28	5.53	0.89	0.02
XLOC_003024	pla2g15	48.98	21.49	1.19	0.02
XLOC_010736	itpr3	0.92	0.51	0.85	0.02

XLOC_011808	-	21.28	12.41	0.78	0.02
XLOC_019567	si:dkeyp-118h3.6	1.97	0.77	1.35	0.02
XLOC_004612	abcb6a (1 of many)	3.76	2.13	0.82	0.02
XLOC_011118	srsf5b	26.03	44.76	-0.78	0.02
XLOC_026703	-	3.55	0.29	3.61	0.02
XLOC_019272	necap1	22.92	13.82	0.73	0.02
XLOC_022325	RGS3	25.45	13.22	0.94	0.02
XLOC_030228	-	6.96	23.17	-1.74	0.02
XLOC_030559	-	0.50	2.30	-2.19	0.02
XLOC_030987	-	0.47	13.26	-4.81	0.02
XLOC_005245	thumpd3	7.01	3.93	0.83	0.02
XLOC_022401	-	0.31	5.18	-4.06	0.02
XLOC_003912	-	0	5.17	-inf	0.02
XLOC_010626	-	0	10.83	-inf	0.02
XLOC_014982	-	2.20	7.46	-1.76	0.02
XLOC_026916	-	407.63	718.18	-0.82	0.02
XLOC_027595	-	6.41	15.73	-1.30	0.02
XLOC_013111	mfsd2aa	0.68	1.70	-1.32	0.02
XLOC_021310	rtel1	1.16	2.15	-0.89	0.02
XLOC_025654	-	7.29	2.56	1.51	0.02
XLOC_001558	PNPT1	2.51	1.32	0.93	0.02
XLOC_005989	cdk12	8.17	4.92	0.73	0.02
XLOC_012476	WDSUB1 (1 of many)	0.46	1.63	-1.83	0.02
XLOC_026072	-	10.33	2.73	1.92	0.02
XLOC_001638	cryzl1	4.33	7.79	-0.85	0.02
XLOC_020168	-	34.91	8.17	2.10	0.02
XLOC_021195	sod3b	63.64	29.00	1.13	0.02
XLOC_028228	mpdu1b	29.79	18.20	0.71	0.02
XLOC_031285	-	1.09	4.18	-1.94	0.02
XLOC_019066	SLC39A3,sgta	15.14	9.09	0.74	0.02
XLOC_025623	-	20.15	10.76	0.90	0.02
XLOC_001786	zgc:112496	9.87	4.80	1.04	0.02
XLOC_003956	nudt7	4.36	1.23	1.82	0.02
XLOC_004636	-	0.75	5.34	-2.83	0.02
XLOC_012082	rbm14a	24.87	12.09	1.04	0.02
XLOC_025708	ahr2 (1 of many)	8.46	14.57	-0.79	0.02
XLOC_014809	MRPL49	46.70	27.41	0.77	0.02
XLOC_020819	tescb	0.30	1.60	-2.39	0.02
XLOC_026451	-	0.27	1.56	-2.51	0.02
XLOC_016727	emg1	12.66	6.55	0.95	0.02
XLOC_001792	telo2	1.86	0.90	1.04	0.02
XLOC_004403	-	85.13	3.86	4.46	0.02
XLOC_015590	asb13a.2	2.41	0.64	1.92	0.02
XLOC_024016	ENSONIG00000009050	671.83	1357.44	-1.01	0.02
XLOC_024776	ENSONIG00000006635	286.21	145.74	0.97	0.02
XLOC_013922	-	3.83	1.42	1.43	0.02
XLOC_018034	si:rp71-1c23.3	8.66	15.55	-0.84	0.02
XLOC_009014	wdfy3	0.69	1.17	-0.76	0.02

XLOC_015168	ponzr1 (1 of many)	0.79	3.06	-1.95	0.02
XLOC_023697	cyp3a65 (1 of many)	0.66	3.00	-2.18	0.02
XLOC_025074	akap8l	4.56	9.33	-1.03	0.02
XLOC_004650	timmdc1	4.89	2.53	0.95	0.02
XLOC_014362	-	0.58	2.49	-2.09	0.02
XLOC_023182	TGFB3 (1 of many)	0.97	1.82	-0.91	0.02
XLOC_020431	fgfr1b	3.02	5.12	-0.76	0.02
XLOC_022890	api5	10.58	6.47	0.71	0.02
XLOC_002139	-	5.03	10.48	-1.06	0.02
XLOC_002639	plcd1a	2.72	0.96	1.51	0.02
XLOC_018833	COL24A1	0.30	0.64	-1.11	0.02
XLOC_017994	ubap2b	9.72	5.86	0.73	0.02
XLOC_020391	brip1	0.61	1.29	-1.08	0.02
XLOC_004213	commd4	17.15	29.26	-0.77	0.02
XLOC_004757	fbxo9	17.19	28.05	-0.71	0.02
XLOC_022007	-	0.13	1.20	-3.20	0.02
XLOC_009473	ngrn	4.12	1.29	1.68	0.02
XLOC_015640	aldh1l2	1.38	2.67	-0.95	0.02
XLOC_022123	-	24.56	8.11	1.60	0.02
XLOC_004764	bag2	4.09	0.87	2.23	0.02
XLOC_005907	angptl6	6.55	11.65	-0.83	0.02
XLOC_009026	-	9.13	15.07	-0.72	0.02
XLOC_028866	-	8.19	1.34	2.61	0.02
XLOC_007173	TRAPPC5 (1 of many)	5.74	3.33	0.78	0.02
XLOC_011928	si:ch211-114c12.2	4.22	7.08	-0.75	0.02
XLOC_007082	cdan1	0.84	1.55	-0.88	0.02
XLOC_008439	rrm2b	12.85	7.43	0.79	0.02
XLOC_011807	-	4.67	0.96	2.28	0.02
XLOC_011955	il13ra2	1.45	2.93	-1.01	0.02
XLOC_026627	-	0.52	2.35	-2.18	0.02
XLOC_004259	-	4.67	0.21	4.48	0.02
XLOC_005257	eefsec	3.67	1.56	1.23	0.02
XLOC_020779	exosc2	5.95	3.17	0.91	0.02
XLOC_022739	-	4.25	7.70	-0.86	0.02
XLOC_022894	-	2.12	0.17	3.67	0.02
XLOC_029050	fam20cl	1.38	5.74	-2.05	0.02
XLOC_029740	sox18	0.35	1.13	-1.68	0.02
XLOC_005951	map3k3	1.29	2.46	-0.93	0.02
XLOC_009671	ENSONIG00000009982	0.90	2.51	-1.49	0.02
XLOC_009804	sowahd	0.51	1.75	-1.76	0.02
XLOC_014005	nadkb	21.09	12.94	0.70	0.02
XLOC_025955	ENSONIG00000007060	0.33	0.71	-1.09	0.02
XLOC_001872	-	0.16	1.28	-3.04	0.02
XLOC_004524	hnrnpua	17.76	10.81	0.72	0.02
XLOC_005890	-	8.55	14.25	-0.74	0.02
XLOC_007148	aqr	2.88	1.69	0.77	0.02
XLOC_023934	ggnbp2	8.75	5.19	0.75	0.02
XLOC_003121	enc3	4.75	2.50	0.93	0.02

XLOC_016594	ENSONIG00000012760	8.74	4.92	0.83	0.02
XLOC_023154	-	1.10	5.29	-2.26	0.02
XLOC_007917	-	0.07	2.44	-5.05	0.02
XLOC_014163	ENSONIG00000018036	1.22	0.41	1.56	0.02
XLOC_031303	-	2.17	6.03	-1.47	0.02
XLOC_004579	enpp4	0.54	1.66	-1.62	0.02
XLOC_018175	psmf1	10.39	17.18	-0.73	0.02
XLOC_022768	-	0.28	2.07	-2.87	0.02
XLOC_015931	-	3.76	10.13	-1.43	0.02
XLOC_020358	-	4.97	2.41	1.04	0.02
XLOC_021542	fam188a	3.00	1.58	0.93	0.02
XLOC_030953	-	3.10	5.97	-0.95	0.02
XLOC_001254	dvl3a	4.91	2.91	0.75	0.02
XLOC_005182	krt4	0.96	0.21	2.17	0.02
XLOC_012085	dpp3	9.75	5.87	0.73	0.02
XLOC_014099	-	17.30	10.59	0.71	0.02
XLOC_024135	eif1axb	141.39	79.77	0.83	0.02
XLOC_002974	vps9d1	0.58	1.14	-0.97	0.02
XLOC_021271	tnnc1b	0.08	1.57	-4.31	0.02
XLOC_030168	ENSONIG00000015455	2.90	6.05	-1.06	0.02
XLOC_004759	LRRC1	3.25	1.83	0.83	0.02
XLOC_014636	rffl	2.96	1.70	0.80	0.02
XLOC_019435	SLC9A3R2 (1 of many)	1.31	2.40	-0.87	0.02
XLOC_002830	zgc:194879 (1 of many)	1.46	3.03	-1.06	0.02
XLOC_014668	timm50	6.10	3.05	1.00	0.02
XLOC_012925	arhgef10la	3.88	2.30	0.76	0.02
XLOC_016622	zyx	13.52	7.64	0.82	0.02
XLOC_024318	eml2	1.81	3.24	-0.84	0.02
XLOC_006664	txnrd1	14.82	9.11	0.70	0.02
XLOC_007393	zgc:162544	71.61	118.75	-0.73	0.02
XLOC_011200	aven	8.97	4.33	1.05	0.02
XLOC_011703	atf3	22.14	12.00	0.88	0.02
XLOC_025498	mpp5a	4.99	2.74	0.86	0.02
XLOC_030655	-	0.65	6.36	-3.30	0.02
XLOC_016855	TTC17	13.21	6.50	1.02	0.02
XLOC_022995	si:ch211-87m7.2	2.85	5.04	-0.82	0.02
XLOC_000706	itfg1	12.92	7.91	0.71	0.02
XLOC_010832	dnmt3bb.1	1.64	2.92	-0.83	0.02
XLOC_010829	acss2	4.36	2.57	0.76	0.02
XLOC_009257	mrpl11	24.57	14.37	0.77	0.02
XLOC_027902	EM256-PLSCR3 (1 of m	2.07	6.52	-1.65	0.02
XLOC_010279	hps4	5.93	3.39	0.80	0.02
XLOC_017805	-	1.07	6.81	-2.68	0.02
XLOC_024259	cdr2a	2.22	0.74	1.58	0.02
XLOC_006048	cdip1	19.17	11.29	0.76	0.02
XLOC_027608	-	0.42	1.36	-1.69	0.02
XLOC_006823	tada3l	2.72	1.39	0.96	0.02
XLOC_025939	ncoa4	62.70	110.59	-0.82	0.02

XLOC_004260	-	3.65	0.56	2.71	0.02
XLOC_013345	ENSONIG00000020622	0.36	3.30	-3.19	0.02
XLOC_017771	dolk	2.28	1.12	1.02	0.02
XLOC_022184	trappc10	4.31	2.57	0.74	0.02
XLOC_025599	cdca7b	0.29	0.94	-1.72	0.02
XLOC_001680	-	30.48	18.59	0.71	0.02
XLOC_003184	shc2	12.38	7.58	0.71	0.02
XLOC_023750	-	1.52	0.44	1.80	0.02
XLOC_028551	ehd2b	1.03	2.05	-0.98	0.02
XLOC_030183	usp2a	4.29	1.84	1.22	0.02
XLOC_012731	si:ch211-1o7.3	0.88	2.39	-1.44	0.02
XLOC_015818	nhsl1b	4.15	2.52	0.72	0.02
XLOC_023577	-	4.02	0.87	2.22	0.02
XLOC_027177	smek1	7.45	4.41	0.75	0.02
XLOC_002503	oxsr1b	6.59	4.02	0.71	0.02
XLOC_005953	-	1.36	4.00	-1.56	0.02
XLOC_012203	0000010207,ENSONIG0	130.99	74.58	0.81	0.02
XLOC_014275	-	0.32	2.99	-3.22	0.02
XLOC_017186	mrps36	56.26	22.70	1.31	0.02
XLOC_017307	-	0.62	7.37	-3.57	0.02
XLOC_020842	-	208.87	71.67	1.54	0.02
XLOC_023180	lrrc74a	1.00	0.29	1.77	0.02
XLOC_009205	CTNND1 (1 of many)	3.97	6.78	-0.77	0.02
XLOC_011243	CRISP3	1.63	0.11	3.84	0.02
XLOC_011965	hinfp	3.54	1.93	0.88	0.02
XLOC_021316	ipo9	3.62	2.11	0.78	0.02
XLOC_027509	-	0.58	8.91	-3.94	0.02
XLOC_006511	-	2.97	0.88	1.75	0.02
XLOC_000417	-	0.37	7.37	-4.30	0.02
XLOC_011905	arhgap42a	0.95	1.85	-0.96	0.02
XLOC_013794	slc6a13	0.53	1.37	-1.36	0.02
XLOC_027053	ENSONIG00000009715	4.34	8.60	-0.99	0.02
XLOC_016816	akr1b1	0.99	0.15	2.72	0.02
XLOC_018875	plcb3	1.41	0.70	1.00	0.02
XLOC_001737	-	0.43	16.21	-5.24	0.02
XLOC_004330	wdr20b	3.53	2.07	0.77	0.02
XLOC_004529	emilin1a	1.89	3.32	-0.81	0.02
XLOC_007908	eomesb	0.06	1.75	-4.77	0.02
XLOC_011897	si:dkey-175g6.5	5.73	3.23	0.83	0.02
XLOC_009477	mcat	6.06	3.07	0.98	0.02
XLOC_000748	kifc3	2.07	1.09	0.92	0.02
XLOC_008253	sigirr	6.06	12.88	-1.09	0.02
XLOC_009102	kdm2ab	1.92	3.26	-0.76	0.02
XLOC_016457	-	4.92	0.50	3.30	0.02
XLOC_021300	-	2.16	0.97	1.15	0.02
XLOC_010433	GRTP1 (1 of many)	4.16	1.97	1.08	0.02
XLOC_015948	ENSONIG00000016393	0.99	2.66	-1.43	0.02
XLOC_022051	dhx36	3.78	2.22	0.76	0.02

XLOC_006462	si:dkey-151m15.5	1.52	0.66	1.21	0.02
XLOC_015202	ENSONIG00000009356	0.44	2.64	-2.60	0.02
XLOC_018958	s100u	2.25	3.84	-0.77	0.02
XLOC_005454	vaspb	4.18	2.36	0.83	0.02
XLOC_016218	tmem106ba	9.42	15.38	-0.71	0.02
XLOC_016613	-	1.14	3.96	-1.79	0.02
XLOC_027000	ubtfl	19.91	11.20	0.83	0.02
XLOC_003099	MFN1 (1 of many)	13.33	7.95	0.75	0.02
XLOC_003495	prdx6	134.42	243.34	-0.86	0.02
XLOC_009232	mascRNA-menRNA	431.28	189.27	1.19	0.02
XLOC_011166	elmsan1a	1.60	2.75	-0.78	0.02
XLOC_019849	cryz	26.87	43.43	-0.69	0.02
XLOC_031197	ENSONIG00000001770	4747.62	2559.30	0.89	0.02
XLOC_000627	znf710b	0.90	0.38	1.25	0.02
XLOC_001449	lnpb	1.24	2.45	-0.98	0.02
XLOC_021022	NHLRC3	4.13	6.97	-0.76	0.02
XLOC_025553	ENSONIG00000003974	2.93	6.82	-1.22	0.02
XLOC_005070	fam212aa	0.19	0.94	-2.33	0.02
XLOC_006709	zmynd8	3.72	6.32	-0.76	0.02
XLOC_015066	CLPB	9.63	5.12	0.91	0.02
XLOC_017106	-	24.90	6.39	1.96	0.02
XLOC_022514	-	0.19	0.94	-2.33	0.02
XLOC_009457	eno1a	72.39	133.02	-0.88	0.02
XLOC_009728	ttc4	9.46	5.33	0.83	0.02
XLOC_024943	-	3.52	1.94	0.86	0.02
XLOC_025102	-	10.27	17.33	-0.75	0.02
XLOC_003615	-	3.03	1.19	1.35	0.02
XLOC_017981	cdk7	6.40	3.37	0.92	0.02
XLOC_000536	larp6	1.12	0.47	1.26	0.02
XLOC_010958	pgm5	3.33	6.23	-0.90	0.02
XLOC_013868	slc38a3b	47.68	27.60	0.79	0.02
XLOC_014777	-	1.90	11.41	-2.59	0.02
XLOC_005735	-	14.03	5.15	1.45	0.02
XLOC_015930	-	6.88	22.78	-1.73	0.02
XLOC_024822	mllt4a	16.13	9.78	0.72	0.02
XLOC_029072	try	2683.56	10566	-1.98	0.02
XLOC_023186	eif2b4	12.93	7.74	0.74	0.02
XLOC_027328	asnsd1	17.02	9.12	0.90	0.02
XLOC_010180	cnih1	5.25	3.10	0.76	0.02
XLOC_008513	snrpd1	9.43	5.60	0.75	0.02
XLOC_020640	as3mt	26.69	15.95	0.74	0.02
XLOC_001486	plek	1.61	3.01	-0.90	0.02
XLOC_004329	-	1.15	3.98	-1.80	0.02
XLOC_014021	cc2d1b	3.50	2.11	0.73	0.02
XLOC_022088	-	0.25	1.51	-2.58	0.02
XLOC_024624	-	4.43	1.94	1.19	0.02
XLOC_009144	tgfb2l	1.29	0.47	1.46	0.02
XLOC_020002	scp2a	79.65	140.49	-0.82	0.02

XLOC_023798	ENSONIG00000020813	0.65	2.20	-1.75	0.02
XLOC_007613	tgfbi	3.95	6.71	-0.76	0.02
XLOC_000196	-	1.24	3.49	-1.49	0.02
XLOC_008666	-	22.31	159.31	-2.84	0.02
XLOC_012726	si:dkeyp-75h12.2	6.28	3.65	0.79	0.02
XLOC_014015	usp33	4.41	2.62	0.75	0.02
XLOC_021906	-	0.39	1.85	-2.26	0.02
XLOC_030201	-	7143.47	3515.44	1.02	0.02
XLOC_021543	-	0.65	2.40	-1.89	0.02
XLOC_022107	ENSONIG00000003754	0.27	0.87	-1.70	0.02
XLOC_027123	-	0.60	3.22	-2.41	0.02
XLOC_005261	FGD5 (1 of many)	0.51	1.03	-1.02	0.02
XLOC_018061	epg5	8.90	5.08	0.81	0.02
XLOC_019743	fosaa,mlh3	2.22	0.80	1.48	0.02
XLOC_005663	anxa11b	13.17	21.25	-0.69	0.02
XLOC_005865	gosr2	24.86	15.35	0.70	0.02
XLOC_010773	-	4.56	8.73	-0.94	0.02
XLOC_010926	gnaq	3.24	1.30	1.32	0.02
XLOC_021865	ENSONIG00000003580	0.21	0.72	-1.80	0.02
XLOC_024244	ENSONIG00000010863	20.75	11.35	0.87	0.02
XLOC_025092	-	4.63	13.50	-1.55	0.02
XLOC_002872	nutf2	52.00	32.36	0.68	0.02
XLOC_007616	rest	3.72	2.27	0.71	0.02
XLOC_016979	-	12.18	5.07	1.26	0.02
XLOC_017629	creb3l3a	28.36	49.89	-0.81	0.02
XLOC_020879	ENSONIG00000016400	3.47	6.07	-0.81	0.02
XLOC_008363	-	2.18	0.34	2.66	0.02
XLOC_014317	-	2.75	14.73	-2.42	0.02
XLOC_019803	ENSONIG00000020177	518.60	266.73	0.96	0.02
XLOC_001985	rhot2	5.65	3.18	0.83	0.02
XLOC_006660	PBLD (1 of many)	2.40	4.89	-1.03	0.02
XLOC_017864	-	9.28	3.20	1.54	0.02
XLOC_018477	LIMD1	2.27	1.18	0.95	0.02
XLOC_019566	nck2a	2.25	0.96	1.23	0.02
XLOC_021252	-	27.29	14.72	0.89	0.02
XLOC_026857	-	3.94	0.61	2.68	0.02
XLOC_007979	zgc:110410	7.90	13.15	-0.73	0.02
XLOC_021533	si:ch211-269k10.5	0.47	1.49	-1.67	0.02
XLOC_011103	mtif3	3.62	1.94	0.90	0.02
XLOC_015179	-	82.08	29.30	1.49	0.02
XLOC_023938	0000021788,ENSONIG0	2.62	16.32	-2.64	0.02
XLOC_030072	ENSONIG00000015994	2.57	1.37	0.91	0.02
XLOC_006008	zgc:153952	2.15	3.97	-0.88	0.02
XLOC_007287	nr1d2a	10.45	6.32	0.72	0.02
XLOC_009490	si:dkeyp-38g8.5	2.44	0.79	1.63	0.02
XLOC_010804	hm13,mrgbp	49.55	27.99	0.82	0.02
XLOC_030129	-	1.09	3.99	-1.87	0.02
XLOC_023128	-	21.41	5.20	2.04	0.02

XLOC_005363	-	14.39	7.53	0.93	0.02
XLOC_010645	rmnd5b	9.43	5.81	0.70	0.02
XLOC_020024	ENSONIG00000018657	0.62	1.72	-1.47	0.02
XLOC_012145	zgc:77158	5.74	3.35	0.78	0.02
XLOC_020004	angptl3 (1 of many)	0.23	1.07	-2.23	0.02
XLOC_001093	tcea1	15.62	9.53	0.71	0.02
XLOC_004922	DST	2.70	6.65	-1.30	0.02
XLOC_013624	tnnt2a	1.45	0.50	1.55	0.02
XLOC_000156	-	21.03	34.04	-0.69	0.02
XLOC_004091	pdhx	21.46	13.41	0.68	0.02
XLOC_005154	zgc:101564	3.53	1.87	0.91	0.02
XLOC_015578	cyp4v8	35.61	58.54	-0.72	0.02
XLOC_016566	RRAGC (1 of many)	5.26	3.19	0.72	0.02
XLOC_009855	ASS1	13.40	7.99	0.75	0.02
XLOC_013872	rrp9	3.30	1.53	1.11	0.02
XLOC_014116	zgc:110329	2.78	1.32	1.08	0.02
XLOC_028549	bckdha	15.20	24.97	-0.72	0.02
XLOC_012389	tomm40l	3.91	1.55	1.34	0.02
XLOC_014907	lrrk2	0.75	2.16	-1.53	0.02
XLOC_022691	-	3.18	5.44	-0.77	0.02
XLOC_003336	si:ch211-232d19.4	3.06	0.99	1.63	0.02
XLOC_006845	ENSONIG00000020351	2.83	4.74	-0.74	0.02
XLOC_016454	-	2.23	0.69	1.68	0.02
XLOC_019703	si:dkey-33c12.12	8.05	14.06	-0.80	0.02
XLOC_002446	ENSONIG00000017328	3.00	5.68	-0.92	0.02
XLOC_011791	hephl1b	6.18	3.76	0.72	0.02
XLOC_020790	-	1.13	0.17	2.74	0.02
XLOC_022895	TICRR	0.39	0.87	-1.17	0.02
XLOC_027067	-	8.49	5.13	0.73	0.02
XLOC_000564	adgrg1	2.53	4.42	-0.80	0.02
XLOC_001338	ENSONIG00000000388	4.11	7.90	-0.94	0.02
XLOC_004435	tpd52l1	3.16	5.53	-0.81	0.02
XLOC_019129	ctsd	142.75	85.10	0.75	0.02
XLOC_029033	-	1.06	2.71	-1.35	0.02
XLOC_014304	tbx21	0.13	0.83	-2.66	0.02
XLOC_024038	-	1.91	0.08	4.53	0.02
XLOC_027521	inip	14.02	5.81	1.27	0.02
XLOC_006448	ENSONIG00000009690	2.16	1.09	0.98	0.02
XLOC_022547	hnrnpd	5.09	11.72	-1.20	0.02
XLOC_029298	-	2.11	0.42	2.34	0.02
XLOC_008194	nt5dc3	5.05	2.95	0.78	0.02
XLOC_027736	-	25.50	42.84	-0.75	0.02
XLOC_000863	exosc4	8.08	3.82	1.08	0.02
XLOC_007178	-	1.25	2.71	-1.12	0.02
XLOC_012062	-	14.90	1.94	2.94	0.02
XLOC_023967	shpk	7.39	4.37	0.76	0.02
XLOC_000723	dpy19l3	3.00	1.65	0.87	0.02
XLOC_003459	polr2eb	28.65	17.81	0.69	0.02

XLOC_012070	plac8l1	3.40	9.02	-1.41	0.02
XLOC_016781	ENSONIG00000016824	3.02	1.37	1.14	0.02
XLOC_008182	edc3	1.29	0.62	1.05	0.02
XLOC_017124	SNORA13	86.21	52.84	0.71	0.02
XLOC_022755	cdk11b	6.99	4.31	0.70	0.02
XLOC_005456	cfap45	5.87	2.32	1.34	0.02
XLOC_007235	plbd1	12.77	21.48	-0.75	0.02
XLOC_011082	pabpc4	30.97	51.65	-0.74	0.02
XLOC_028280	mccc1	8.72	5.44	0.68	0.02
XLOC_006873	ganc	5.13	3.06	0.75	0.02
XLOC_007891	sox4b	0.32	1.22	-1.91	0.02
XLOC_013121	pank2	22.04	13.77	0.68	0.02
XLOC_018808	ENSONIG00000008953	1.18	0.28	2.08	0.02
XLOC_022187	-	0.53	1.75	-1.72	0.02
XLOC_016125	prcp	12.76	20.64	-0.69	0.02
XLOC_028646	atg10	11.87	6.68	0.83	0.02
XLOC_010764	-	6.25	0.98	2.68	0.02
XLOC_014984	-	2.80	12.26	-2.13	0.02
XLOC_017017	flad1	3.03	1.70	0.84	0.02
XLOC_020810	dgcr2	1.60	2.98	-0.90	0.02
XLOC_006464	ifi35	22.11	45.87	-1.05	0.02
XLOC_031060	-	0.79	4.49	-2.50	0.02
XLOC_001021	si:dkey-208k22.3	12.50	7.40	0.76	0.02
XLOC_009211	-	0.78	2.79	-1.84	0.02
XLOC_019138	si:ch211-71n6.4	11.00	17.65	-0.68	0.02
XLOC_027229	-	16.50	32.42	-0.97	0.02
XLOC_027942	fam117ab	0.57	1.24	-1.12	0.02
XLOC_029045	-	1.68	4.91	-1.54	0.02
XLOC_001672	LMF1	8.51	5.12	0.73	0.02
XLOC_003122	ENSONIG00000001832	5.80	10.73	-0.89	0.02
XLOC_008462	-	1.71	5.94	-1.80	0.02
XLOC_022429	golga1	2.38	1.32	0.85	0.02
XLOC_006541	atxn2l	2.16	3.74	-0.79	0.02
XLOC_007238	cbx7a	7.95	4.73	0.75	0.02
XLOC_013822	ENSONIG00000004397	47.66	78.07	-0.71	0.02
XLOC_005429	samsn1a	8.33	13.97	-0.75	0.02
XLOC_008512	adnp2b	67.81	32.78	1.05	0.02
XLOC_026509	ENSONIG00000017280	0.95	0.35	1.45	0.02
XLOC_017250	lifra,spef2	13.85	8.65	0.68	0.03
XLOC_020113	cox10	5.32	3.04	0.81	0.03
XLOC_021448	rxrbb	16.56	27.44	-0.73	0.03
XLOC_029201	KIF1C	2.79	1.70	0.72	0.03
XLOC_006609	-	0.54	3.14	-2.53	0.03
XLOC_009725	TIMP3	2.97	1.32	1.17	0.03
XLOC_018680	rnf24	2.67	0.92	1.54	0.03
XLOC_021253	-	188.22	101.14	0.90	0.03
XLOC_024356	-	0.51	1.54	-1.59	0.03
XLOC_024449	-	0	4.63	-inf	0.03

XLOC_028188	-	0	12.63	-inf	0.03
XLOC_011773	sod1	287.36	166.53	0.79	0.03
XLOC_013929	-	3.99	1.26	1.66	0.03
XLOC_015192	-	0.26	5.50	-4.42	0.03
XLOC_021503	-	1.14	3.31	-1.53	0.03
XLOC_028835	-	2.86	0.39	2.87	0.03
XLOC_003265	mettl13	1.25	0.39	1.68	0.03
XLOC_001471	ccdc172	0	0.91	-inf	0.03
XLOC_005049	usp49	2.58	4.60	-0.84	0.03
XLOC_023850	-	1.48	0.14	3.43	0.03
XLOC_028568	nup88	3.10	1.62	0.94	0.03
XLOC_029110	pdxdc1	4.39	2.69	0.71	0.03
XLOC_012821	ENSONIG00000004213	3.68	1.02	1.85	0.03
XLOC_016008	usp36	1.64	0.86	0.93	0.03
XLOC_021582	-	0.66	2.65	-2.01	0.03
XLOC_024794	ENSONIG00000006627	2.31	7.96	-1.79	0.03
XLOC_006846	kbtbd8	2.49	1.40	0.83	0.03
XLOC_009025	-	14.54	25.45	-0.81	0.03
XLOC_024283	-	0.95	3.32	-1.81	0.03
XLOC_011015	ENSONIG00000008144	3.19	6.86	-1.10	0.03
XLOC_011978	p4ha2	5.56	2.92	0.93	0.03
XLOC_021212	bdh2	94.58	153.48	-0.70	0.03
XLOC_025881	si:ch211-14a17.11	64.98	151.68	-1.22	0.03
XLOC_030372	-	0.46	1.66	-1.85	0.03
XLOC_008943	-	0.66	3.33	-2.34	0.03
XLOC_021047	mrpl21	14.61	7.48	0.97	0.03
XLOC_026542	pofut1	11.07	6.69	0.73	0.03
XLOC_027536	-	14.93	3.43	2.12	0.03
XLOC_024372	-	11.02	1.48	2.90	0.03
XLOC_006423	rnf25	15.57	9.35	0.74	0.03
XLOC_014352	stab2	1.76	1.04	0.76	0.03
XLOC_026102	-	2.53	1.49	0.76	0.03
XLOC_007604	srp72	33.02	20.63	0.68	0.03
XLOC_003242	cyp2n13	427.46	108.41	1.98	0.03
XLOC_006768	ENSONIG00000020254	8.63	23.61	-1.45	0.03
XLOC_024402	tp53inp1	0.65	1.75	-1.43	0.03
XLOC_015124	-	122.20	53.38	1.19	0.03
XLOC_026464	-	5.41	1.73	1.64	0.03
XLOC_028278	kng1	464.14	887.26	-0.93	0.03
XLOC_004751	zgc:162183	1.31	0.57	1.22	0.03
XLOC_010781	-	0.85	2.24	-1.39	0.03
XLOC_013556	larp4aa	10.02	5.06	0.99	0.03
XLOC_021338	-	3.46	0.58	2.59	0.03
XLOC_016671	-	3.19	1.30	1.30	0.03
XLOC_017141	ndufa3	27.97	16.80	0.73	0.03
XLOC_017282	ENSONIG00000007481	5.96	3.54	0.75	0.03
XLOC_019010	-	1.25	2.55	-1.03	0.03
XLOC_025207	-	3.56	6.83	-0.94	0.03

XLOC_000212	-	117.15	8.13	3.85	0.03
XLOC_020114	hs3st3b1b	8.99	5.57	0.69	0.03
XLOC_005572	mipep	5.48	3.14	0.80	0.03
XLOC_007693	-	12.07	6.39	0.92	0.03
XLOC_019787	kif26ab	0.26	0.99	-1.94	0.03
XLOC_021330	-	5.55	1.42	1.96	0.03
XLOC_003330	-	6.70	13.13	-0.97	0.03
XLOC_018655	psip1	3.33	1.79	0.90	0.03
XLOC_011196	abracl	0.37	1.81	-2.29	0.03
XLOC_016787	-	4.29	7.97	-0.89	0.03
XLOC_029999	-	15.23	2.71	2.49	0.03
XLOC_001310	ENSONIG00000010664	10.36	3.86	1.43	0.03
XLOC_015499	-	2.52	0.87	1.54	0.03
XLOC_022642	-	2.02	4.08	-1.01	0.03
XLOC_027984	khnyn	1.36	2.39	-0.82	0.03
XLOC_007344	triobpb	18.57	11.01	0.75	0.03
XLOC_014725	si:ch73-40i7.5	6.40	3.38	0.92	0.03
XLOC_018013	ap1b1	14.13	8.91	0.66	0.03
XLOC_019581	otc	2.37	0.90	1.39	0.03
XLOC_028761	-	73.28	26.01	1.49	0.03
XLOC_000140	ENSONIG00000017783	14.68	24.49	-0.74	0.03
XLOC_005753	ENSONIG00000016016	5.11	2.73	0.90	0.03
XLOC_024672	ENSONIG00000018303	1.13	3.19	-1.50	0.03
XLOC_011430	slc2a10	5.35	2.69	0.99	0.03
XLOC_015984	-	8.93	15.97	-0.84	0.03
XLOC_016010	socs3a	35.52	21.72	0.71	0.03
XLOC_021736	ak2	68.72	115.30	-0.75	0.03
XLOC_026776	-	0.39	3.96	-3.36	0.03
XLOC_019661	slc25a1b	100.73	58.52	0.78	0.03
XLOC_009428	znf362b	0.81	1.80	-1.16	0.03
XLOC_013519	-	182.97	57.31	1.67	0.03
XLOC_024595	vps37c	3.82	2.37	0.69	0.03
XLOC_027513	trit1	3.06	1.47	1.05	0.03
XLOC_007606	arl9	2.54	1.06	1.26	0.03
XLOC_012449	nup62l	8.17	4.94	0.73	0.03
XLOC_004879	-	4.39	2.03	1.12	0.03
XLOC_029387	-	0.64	3.64	-2.51	0.03
XLOC_012955	-	5.04	1.58	1.67	0.03
XLOC_024284	-	0.12	1.47	-3.63	0.03
XLOC_001875	-	23.61	116.06	-2.30	0.03
XLOC_000727	zgc:112160,zgc:136461	565.20	1980.05	-1.81	0.03
XLOC_007030	numb	7.31	4.49	0.70	0.03
XLOC_024983	zgc:158225	0.63	1.42	-1.18	0.03
XLOC_010581	trim105	21.83	41.07	-0.91	0.03
XLOC_012845	NFKB1	2.27	4.12	-0.86	0.03
XLOC_024601	-	0.38	5.19	-3.76	0.03
XLOC_001874	SSTR2 (1 of many)	21.04	35.99	-0.77	0.03
XLOC_019191	-	45.77	6.70	2.77	0.03

XLOC_025033	-	0.90	1.98	-1.13	0.03
XLOC_030707	rgn	27.24	45.40	-0.74	0.03
XLOC_004187	si:dkey-24l11.2	13.91	21.86	-0.65	0.03
XLOC_013539	-	1.62	3.42	-1.07	0.03
XLOC_019907	rcan3	0.84	0.11	3.00	0.03
XLOC_020902	-	0.40	1.73	-2.11	0.03
XLOC_028180	ENSONIG00000016596	0.49	1.29	-1.38	0.03
XLOC_004205	-	0.15	1.59	-3.39	0.03
XLOC_028747	-	3.40	1.32	1.37	0.03
XLOC_009366	-	8.39	5.09	0.72	0.03
XLOC_010833	ppdpfb	102.07	181.07	-0.83	0.03
XLOC_022634	ENSONIG00000013031	1.20	2.40	-1.00	0.03
XLOC_024604	lgals2b	2.01	1.04	0.95	0.03
XLOC_026997	klhl18	21.70	12.10	0.84	0.03
XLOC_029060	-	2.34	8.09	-1.79	0.03
XLOC_001489	sgms1	18.09	11.37	0.67	0.03
XLOC_008279	tspan18b	0.63	1.42	-1.18	0.03
XLOC_016984	galnt1	1.10	2.20	-1.00	0.03
XLOC_003699	srsf1a (1 of many)	31.50	50.44	-0.68	0.03
XLOC_013421	-	0.57	4.76	-3.06	0.03
XLOC_013514	-	0.70	9.43	-3.76	0.03
XLOC_020480	apc	11.56	7.21	0.68	0.03
XLOC_020644	pdcd11	3.01	1.46	1.05	0.03
XLOC_022375	-	1.46	2.60	-0.83	0.03
XLOC_026833	ints2	4.50	2.41	0.90	0.03
XLOC_031210	ENSONIG00000000412	0.25	8.38	-5.07	0.03
XLOC_021903	ENSONIG00000020644	1.36	0.53	1.36	0.03
XLOC_029823	-	2.85	14.15	-2.31	0.03
XLOC_031084	ENSONIG00000000040	0.29	1.25	-2.11	0.03
XLOC_001162	PHACTR1 (1 of many)	0.80	1.92	-1.26	0.03
XLOC_001671	ADAP1 (1 of many)	0.05	0.98	-4.15	0.03
XLOC_015761	-	7.39	3.66	1.01	0.03
XLOC_020832	nipblb	0.62	1.08	-0.79	0.03
XLOC_022133	-	23.00	4.12	2.48	0.03
XLOC_024212	ENSONIG00000013821	4.69	8.26	-0.81	0.03
XLOC_027065	drap1	12.47	5.83	1.10	0.03
XLOC_003809	phpt1	7.13	12.44	-0.80	0.03
XLOC_004106	si:ch211-245j22.3	0.47	2.94	-2.64	0.03
XLOC_009536	apaf1	0.89	1.58	-0.83	0.03
XLOC_012826	lman2	60.25	34.54	0.80	0.03
XLOC_022850	opa1	7.68	4.83	0.67	0.03
XLOC_011485	-	0.81	1.92	-1.24	0.03
XLOC_019254	ENSONIG00000006974	0	4.19	-inf	0.03
XLOC_020808	-	0.42	7.66	-4.19	0.03
XLOC_006333	sord	43.13	72.31	-0.75	0.03
XLOC_019262	tpi1a	0.87	0.22	1.99	0.03
XLOC_025994	ENSONIG00000011315	0.16	0.93	-2.53	0.03
XLOC_027191	ubl3a	25.20	15.31	0.72	0.03

XLOC_020472	ENSONIG00000012622	167.68	88.68	0.92	0.03
XLOC_030003	abcf1	12.70	7.09	0.84	0.03
XLOC_006700	hcfc1a	2.99	1.84	0.70	0.03
XLOC_010639	-	0.21	1.26	-2.61	0.03
XLOC_011603	ENSONIG00000012312	0.19	0.83	-2.10	0.03
XLOC_027861	-	0.28	2.05	-2.88	0.03
XLOC_018247	ddx18	11.47	3.28	1.81	0.03
XLOC_023209	batf	9.06	2.80	1.69	0.03
XLOC_004997	wdr11	3.56	1.98	0.85	0.03
XLOC_006200	tead1b	1.89	3.17	-0.74	0.03
XLOC_012329	SLC9A1 (1 of many)	0.27	0.79	-1.58	0.03
XLOC_013287	sept9b	3.43	6.25	-0.86	0.03
XLOC_016004	syngr2a	42.34	26.30	0.69	0.03
XLOC_001937	crym	0.21	5.14	-4.61	0.03
XLOC_003326	aldh7a1	22.98	51.06	-1.15	0.03
XLOC_017357	esr2b	2.96	5.11	-0.79	0.03
XLOC_018353	si:ch211-198a12.6	9.47	6.00	0.66	0.03
XLOC_020180	-	3.64	0.90	2.02	0.03
XLOC_012796	ENSONIG00000012951	1.54	6.87	-2.16	0.03
XLOC_024186	armc8	5.25	3.22	0.70	0.03
XLOC_030149	-	119.73	8.99	3.74	0.03
XLOC_030181	tbcela	11.08	17.98	-0.70	0.03
XLOC_003039	nfil3	5.71	3.41	0.74	0.03
XLOC_013008	AKT1	0.65	1.50	-1.21	0.03
XLOC_007610	-	1.95	8.49	-2.12	0.03
XLOC_009122	pola2	0.59	1.44	-1.29	0.03
XLOC_010658	0000017814,ENSONIG0	2.89	7.38	-1.35	0.03
XLOC_019713	mrpl35	28.17	16.92	0.74	0.03
XLOC_022757	MIB2	7.55	12.20	-0.69	0.03
XLOC_023459	mtmr7a	4.74	7.81	-0.72	0.03
XLOC_001214	gpsm2	1.45	2.57	-0.83	0.03
XLOC_003086	si:dkey-261o10.2	2.29	3.79	-0.72	0.03
XLOC_006625	-	21.00	33.45	-0.67	0.03
XLOC_008480	poc1bl	2.41	1.32	0.87	0.03
XLOC_005241	WDR77	2.41	1.16	1.06	0.03
XLOC_008173	sqrdl	47.91	27.30	0.81	0.03
XLOC_010535	sh2d1ab	0.71	8.40	-3.56	0.03
XLOC_010841	RAB29	1.26	0.40	1.65	0.03
XLOC_016902	mrpl28	16.71	8.89	0.91	0.03
XLOC_017748	-	1.02	2.04	-0.99	0.03
XLOC_003998	mef2aa	0.53	1.35	-1.35	0.03
XLOC_006953	-	4.06	9.19	-1.18	0.03
XLOC_009679	fcho1	3.27	5.86	-0.84	0.03
XLOC_015104	-	0.21	2.92	-3.80	0.03
XLOC_018566	klf3	1.28	0.62	1.06	0.03
XLOC_018780	socs5a	1.18	0.47	1.33	0.03
XLOC_027297	-	0.60	2.35	-1.96	0.03
XLOC_030992	ENSONIG00000012484	7.32	3.19	1.20	0.03

XLOC_022617	hnrnpaba	42.70	23.54	0.86	0.03
XLOC_007989	-	1.79	4.49	-1.32	0.03
XLOC_010467	ACSF3	6.48	3.76	0.79	0.03
XLOC_012375	lsm10	4.35	1.20	1.86	0.03
XLOC_014422	-	151.26	93.01	0.70	0.03
XLOC_014610	SNORD15	3.99	22.66	-2.51	0.03
XLOC_006122	-	42.62	68.21	-0.68	0.03
XLOC_027109	lysmd2	0.96	2.25	-1.23	0.03
XLOC_015147	-	0.09	6.17	-6.17	0.03
XLOC_015573	casp3a	4.84	7.88	-0.70	0.03
XLOC_027515	sh3bp5a	2.06	3.70	-0.85	0.03
XLOC_028545	AXL	0.54	1.28	-1.25	0.03
XLOC_002749	-	0.74	6.20	-3.07	0.03
XLOC_012647	sdr39u1	5.97	11.14	-0.90	0.03
XLOC_000446	-	2.86	0.73	1.96	0.03
XLOC_003812	-	0.75	1.88	-1.33	0.03
XLOC_008035	PHACTR4 (1 of many)	8.45	5.36	0.66	0.03
XLOC_023464	ENSONIG00000017631	5.33	3.15	0.76	0.03
XLOC_031436	-	27.16	12.08	1.17	0.03
XLOC_014955	fam65a	4.72	7.42	-0.65	0.03
XLOC_024358	hif1al	85.68	50.58	0.76	0.03
XLOC_001987	nlrc3	0.35	0.81	-1.23	0.03
XLOC_003445	h211-212d10.1 (1 of ma	0.90	2.60	-1.53	0.03
XLOC_020951	hspbp1	6.94	0.98	2.82	0.03
XLOC_030375	mllt3	1.06	0.43	1.29	0.03
XLOC_025008	rraga	3.33	5.35	-0.69	0.03
XLOC_001361	dis3	3.75	2.04	0.88	0.03
XLOC_015149	-	3.42	14.19	-2.05	0.03
XLOC_025878	ap1g2	90.63	50.53	0.84	0.03
XLOC_005657	pald1a	0.37	0.96	-1.37	0.03
XLOC_013776	-	60.22	114.60	-0.93	0.03
XLOC_005412	-	4.32	7.82	-0.86	0.03
XLOC_008598	aatf	1.47	0.52	1.50	0.03
XLOC_011297	-	2.74	6.87	-1.33	0.03
XLOC_001891	tefa	9.31	5.88	0.66	0.03
XLOC_018079	gpc4	5.29	8.39	-0.67	0.03
XLOC_018807	nxpe3 (1 of many)	1.24	2.94	-1.24	0.03
XLOC_027301	-	0.16	1.20	-2.90	0.03
XLOC_008075	SOX4 (1 of many)	0.18	1.22	-2.73	0.03
XLOC_025365	-	0.87	5.66	-2.71	0.03
XLOC_005188	trim107	3.09	5.44	-0.82	0.03
XLOC_019443	rhot1a	6.49	4.00	0.70	0.03
XLOC_021428	ENSONIG00000002742	0.26	1.16	-2.16	0.03
XLOC_022384	-	1.51	5.26	-1.80	0.03
XLOC_011007	tfb1m	3.13	1.40	1.16	0.03
XLOC_011130	bpnt1	2.41	6.55	-1.44	0.03
XLOC_021169	ENSONIG00000010731	32.06	15.59	1.04	0.03
XLOC_022916	plekha7a	9.53	15.39	-0.69	0.03

XLOC_023795	cgrrf1	6.46	4.01	0.69	0.03
XLOC_008100	-	1.50	0.19	3.00	0.03
XLOC_020309	-	0.97	3.09	-1.67	0.03
XLOC_024345	-	11.26	1.72	2.71	0.03
XLOC_026398	pin1	31.37	19.92	0.65	0.03
XLOC_029236	-	62.76	158.52	-1.34	0.03
XLOC_030429	-	1.59	0.24	2.72	0.03
XLOC_030906	-	94.92	239.22	-1.33	0.03
XLOC_005997	znf385c	1.18	2.05	-0.80	0.03
XLOC_010177	si:ch211-195e19.1	10.43	6.19	0.75	0.03
XLOC_012433	cycsb (1 of many)	46.06	21.67	1.09	0.03
XLOC_019457	-	9.67	4.00	1.27	0.03
XLOC_030113	-	14.67	3.24	2.18	0.03
XLOC_030623	-	66.16	7.70	3.10	0.03
XLOC_005316	METTL21B	4.56	1.84	1.31	0.03
XLOC_008942	tmem175	1.54	3.20	-1.06	0.03
XLOC_013465	-	2.50	5.28	-1.08	0.03
XLOC_019463	gtpbp1	3.84	2.24	0.78	0.03
XLOC_019544	irs2b	1.83	3.17	-0.80	0.03
XLOC_022630	-	6.93	2.92	1.25	0.03
XLOC_009692	-	0.40	3.09	-2.93	0.03
XLOC_001637	cbr1 (1 of many)	8.83	16.62	-0.91	0.03
XLOC_002596	rdh12l	7.25	3.65	0.99	0.03
XLOC_004915	ICK	0.32	0.85	-1.39	0.03
XLOC_009568	ccdc59	11.03	5.98	0.88	0.03
XLOC_011715	-	4.35	1.08	2.01	0.03
XLOC_018893	-	39.07	24.03	0.70	0.03
XLOC_015165	ponzr1 (1 of many)	4.60	8.15	-0.83	0.03
XLOC_017720	dolpp1	10.32	5.09	1.02	0.03
XLOC_018162	tarbp2	2.80	1.56	0.84	0.03
XLOC_028142	-	4.59	1.55	1.56	0.03
XLOC_030304	ENSONIG00000015501	0.70	1.30	-0.90	0.03
XLOC_024008	NOP9	2.20	1.12	0.98	0.03
XLOC_002971	-	72.34	14.42	2.33	0.03
XLOC_008806	galt	9.81	25.08	-1.35	0.03
XLOC_023972	pigl	2.91	6.54	-1.17	0.03
XLOC_017910	rfk	10.35	4.25	1.28	0.03
XLOC_003717	ENSONIG00000012526	0.14	0.98	-2.82	0.03
XLOC_020912	ENSONIG00000019363	0.35	1.02	-1.53	0.03
XLOC_029945	-	18.26	4.38	2.06	0.03
XLOC_001151	-	0.56	2.44	-2.11	0.03
XLOC_005979	phb	68.70	43.64	0.65	0.03
XLOC_013360	-	0.90	14.79	-4.04	0.03
XLOC_018018	atp6v0a2b	2.43	1.39	0.81	0.03
XLOC_030922	ENSONIG00000014850	1.74	0.28	2.65	0.03
XLOC_031248	-	1.01	4.93	-2.29	0.03
XLOC_002024	-	30.83	2.57	3.59	0.03
XLOC_006413	-	0.75	3.38	-2.18	0.03

XLOC_025039	-	12.91	45.00	-1.80	0.03
XLOC_006243	emc8	9.12	4.11	1.15	0.03
XLOC_008350	avpr1ab	1.04	0.39	1.42	0.03
XLOC_017626	EEF2 (1 of many)	1706.62	806.29	1.08	0.03
XLOC_017936	eif4ebp1	6.42	3.82	0.75	0.03
XLOC_020295	dpys	13.94	24.59	-0.82	0.03
XLOC_020546	-	5.67	2.49	1.19	0.03
XLOC_023846	-	2.73	8.52	-1.64	0.03
XLOC_015217	ENSONIG00000009368	0.25	1.25	-2.34	0.03
XLOC_025713	exoc6b	0.31	1.20	-1.94	0.03
XLOC_030305	-	0.76	10.16	-3.75	0.03
XLOC_017641	-	1.72	44.41	-4.69	0.03
XLOC_009422	ENSONIG00000002165	1.92	1.12	0.77	0.03
XLOC_011405	dnase1	2.64	27.39	-3.37	0.03
XLOC_016989	ENSONIG00000006042	2.88	0.85	1.77	0.03
XLOC_023394	-	8.31	0.80	3.37	0.03
XLOC_017154	-	13.63	6.56	1.05	0.03
XLOC_017226	dhx37	1.88	1.03	0.87	0.03
XLOC_018131	ENSONIG00000015804	12.26	7.79	0.65	0.03
XLOC_003925	ube2nb	16.22	10.03	0.69	0.03
XLOC_014071	-	0.97	3.14	-1.70	0.03
XLOC_021913	-	0.18	1.44	-3.02	0.03
XLOC_004325	slc25a47a	2.82	1.37	1.04	0.03
XLOC_008673	-	0.03	1.28	-5.29	0.03
XLOC_026179	ENSONIG00000019916	1.23	2.03	-0.73	0.03
XLOC_019538	-	0.71	5.03	-2.82	0.03
XLOC_022466	-	11.72	20.99	-0.84	0.03
XLOC_004354	trib2	2.44	0.73	1.75	0.03
XLOC_022715	ENSONIG00000017008	0.04	0.81	-4.50	0.03
XLOC_023213	-	4.21	2.26	0.90	0.03
XLOC_007405	nr2f5	3.49	5.94	-0.77	0.03
XLOC_012185	-	0.20	2.86	-3.81	0.03
XLOC_014724	pex11g	8.08	12.92	-0.68	0.03
XLOC_014813	-	0.30	2.59	-3.12	0.03
XLOC_018777	ttc7a	0.37	0.77	-1.04	0.03
XLOC_009803	pttg1	1.36	3.98	-1.55	0.03
XLOC_025442	-	2.01	9.95	-2.30	0.03
XLOC_019196	klf9	20.88	8.34	1.32	0.03
XLOC_000351	-	32.00	76.52	-1.26	0.03
XLOC_021366	setd7,zgc:113425	12.35	7.62	0.70	0.03
XLOC_023834	ENSONIG00000015918	3.19	1.76	0.86	0.03
XLOC_025793	-	0.32	3.81	-3.58	0.03
XLOC_028430	-	12.48	103.84	-3.06	0.03
XLOC_005624	cox7a3	773.09	485.98	0.67	0.03
XLOC_015228	-	4.47	0.66	2.76	0.03
XLOC_000829	usp14	12.95	7.00	0.89	0.03
XLOC_008059	-	1.51	0.48	1.66	0.03
XLOC_027426	ENSONIG00000009902	0.16	1.80	-3.51	0.03

XLOC_010548	lonrf1	3.34	2.02	0.72	0.03
XLOC_011009	prkd3	0.41	0.88	-1.09	0.03
XLOC_011679	ENSONIG00000018820	1.43	2.43	-0.77	0.03
XLOC_018986	RECQL4,cndp2	13.99	7.73	0.86	0.03
XLOC_031426	ENSONIG00000020667	6.62	2.84	1.22	0.03
XLOC_005215	NDUFAF3	6.17	3.05	1.02	0.03
XLOC_026839	-	1.64	5.35	-1.71	0.03
XLOC_010043	cxcr4a	5.28	8.88	-0.75	0.03
XLOC_018472	nr0b2	19.37	11.75	0.72	0.03
XLOC_021140	-	1.58	4.61	-1.55	0.03
XLOC_015320	-	4.63	2.21	1.07	0.03
XLOC_023179	ENSONIG00000015268	1.56	2.76	-0.82	0.03
XLOC_023498	-	0.59	2.52	-2.10	0.03
XLOC_007112	ENSONIG00000001141	4.46	7.08	-0.67	0.03
XLOC_030799	nxpe3 (1 of many)	2.01	3.52	-0.81	0.03
XLOC_000828	-	49.20	14.51	1.76	0.03
XLOC_001742	baiap2l1a	1.40	2.39	-0.77	0.03
XLOC_002937	il6st	25.49	16.12	0.66	0.03
XLOC_021566	cgref1	3.11	0.07	5.49	0.03
XLOC_021824	-	3.43	0.60	2.52	0.03
XLOC_001751	ENSONIG00000019855	0.59	1.81	-1.62	0.03
XLOC_010801	-	2.51	4.31	-0.78	0.03
XLOC_029292	ENSONIG00000013605	0	0.84	-inf	0.03
XLOC_008758	si:ch211-147j13.3	3.28	5.28	-0.69	0.03
XLOC_020389	-	2.92	1.24	1.23	0.03
XLOC_030119	-	0.79	2.67	-1.75	0.03
XLOC_011326	-	4.64	13.16	-1.50	0.03
XLOC_026087	aldh5a1	7.66	1.08	2.83	0.03
XLOC_029825	-	5.92	2.12	1.48	0.03
XLOC_000307	-	1.66	21.10	-3.67	0.03
XLOC_018030	alkbh2	2.15	5.28	-1.29	0.03
XLOC_028997	-	1.36	8.02	-2.56	0.03
XLOC_006473	-	0.11	1.51	-3.75	0.03
XLOC_010503	SNORA31	9.20	3.38	1.45	0.03
XLOC_015435	olfm1b	23.84	45.08	-0.92	0.03
XLOC_029273	-	3.79	2.06	0.88	0.03
XLOC_014014	zzz3	3.65	2.16	0.75	0.03
XLOC_014022	si:dkey-222f8.3	2.35	4.81	-1.03	0.03
XLOC_015154	-	18.00	50.13	-1.48	0.03
XLOC_019183	bmp1a	48.24	79.46	-0.72	0.03
XLOC_023113	-	12.24	0.62	4.31	0.03
XLOC_023695	arpc1b	237.30	135.48	0.81	0.03
XLOC_012992	DPF3 (1 of many)	2.53	1.42	0.83	0.03
XLOC_015675	VPS26B (1 of many)	7.01	4.40	0.67	0.03
XLOC_021646	eno1b	1.94	3.79	-0.96	0.03
XLOC_011144	eif2s1a	77.33	48.53	0.67	0.03
XLOC_011896	zgc:158234	27.96	17.23	0.70	0.03
XLOC_002912	mef2ca	0.74	1.35	-0.87	0.03

XLOC_025145	ENSONIG00000015553	1.69	0.74	1.19	0.03
XLOC_014371	-	150.16	95.22	0.66	0.03
XLOC_016124	rab30	0.83	3.01	-1.87	0.03
XLOC_020348	-	2.41	2.00	0.27	0.03
XLOC_008107	-	2.81	0.93	1.60	0.03
XLOC_026949	tmem129	16.79	10.56	0.67	0.03
XLOC_025810	-	5.78	14.18	-1.29	0.03
XLOC_004952	slc29a3	4.03	6.90	-0.77	0.03
XLOC_008995	si:ch211-251j10.3	15.71	9.95	0.66	0.03
XLOC_017970	ENSONIG00000013594	21.25	34.96	-0.72	0.03
XLOC_024181	-	1.23	2.49	-1.02	0.03
XLOC_007857	cntnap2a	0.76	2.10	-1.46	0.03
XLOC_012023	stc2b	1.98	0.51	1.96	0.03
XLOC_027642	-	0.43	2.01	-2.23	0.03
XLOC_002845	btbd10a	1.19	2.10	-0.83	0.03
XLOC_003544	ENSONIG00000003159	5.11	3.14	0.70	0.03
XLOC_005988	ppp1r1b	17.74	11.08	0.68	0.03
XLOC_013293	-	0.89	8.99	-3.34	0.03
XLOC_023489	khsrp	2.96	1.57	0.92	0.03
XLOC_000162	-	0.97	3.94	-2.02	0.03
XLOC_000320	ENSONIG00000004005	47.49	0.15	8.29	0.03
XLOC_000677	si:ch1073-459b3.2	0.04	1.31	-5.08	0.03
XLOC_005534	chordc1a (1 of many)	9.76	0.31	5.00	0.03
XLOC_010417	-	5.65	1.09	2.38	0.03
XLOC_015500	usp20	0.88	0.43	1.03	0.03
XLOC_017046	arnt	5.73	8.92	-0.64	0.03
XLOC_017300	-	0.94	4.52	-2.26	0.03
XLOC_019600	gsk3b	3.20	1.24	1.36	0.03
XLOC_021175	suds3	15.12	9.51	0.67	0.03
XLOC_022040	ENSONIG00000007900	0.16	0.76	-2.21	0.03
XLOC_026527	F7	76.84	126.26	-0.72	0.03
XLOC_026608	-	4.30	20.88	-2.28	0.03
XLOC_001614	zc3h15	18.97	12.17	0.64	0.03
XLOC_012531	scrn3	8.50	13.63	-0.68	0.03
XLOC_014719	slc35e1	3.15	1.88	0.74	0.03
XLOC_012262	-	1.69	5.30	-1.65	0.03
XLOC_002239	-	0.99	6.36	-2.69	0.03
XLOC_020308	-	0.25	1.30	-2.39	0.03
XLOC_002691	asap1b	1.26	0.53	1.24	0.03
XLOC_025172	BCL2L1 (1 of many)	11.83	6.83	0.79	0.03
XLOC_008484	sytl1	10.40	6.27	0.73	0.03
XLOC_031092	ehmt1a	3.58	5.67	-0.66	0.03
XLOC_003186	mier2	1.15	0.55	1.07	0.03
XLOC_007005	-	2.39	4.91	-1.04	0.03
XLOC_011723	cd109	1.10	2.19	-1.00	0.03
XLOC_013899	-	2.98	1.08	1.46	0.03
XLOC_024123	-	144.91	21.86	2.73	0.03
XLOC_025643	-	4.56	1.24	1.87	0.03

XLOC_031202	ENSONIG00000014741	4.56	2.21	1.04	0.03
XLOC_000057	tmem220	15.31	23.95	-0.65	0.03
XLOC_001888	cby1	7.82	3.85	1.02	0.03
XLOC_024021	-	2.08	3.61	-0.80	0.03
XLOC_013214	pkn1a	0.49	1.01	-1.04	0.03
XLOC_013402	-	0.36	1.10	-1.61	0.03
XLOC_021666	iars	8.81	5.62	0.65	0.03
XLOC_012697	zgc:66427	2.22	3.75	-0.76	0.03
XLOC_020482	ENSONIG00000012654	6.44	11.95	-0.89	0.03
XLOC_011132	ypel5	4.38	7.65	-0.81	0.03
XLOC_007550	calcrla	1.44	0.61	1.25	0.03
XLOC_010493	LAMP1	17.86	28.01	-0.65	0.03
XLOC_024649	ift88	0.55	1.18	-1.11	0.03
XLOC_004857	-	0.84	2.56	-1.61	0.03
XLOC_010075	rock2a	3.39	5.60	-0.72	0.03
XLOC_011751	si:ch73-212j7.3	1.24	0.68	0.87	0.03
XLOC_023994	ENSONIG00000012884	1.83	0.56	1.72	0.03
XLOC_002766	pdcd2l	1.59	0.65	1.29	0.03
XLOC_007333	RBFOX2 (1 of many)	3.22	8.08	-1.33	0.03
XLOC_004715	RDX (1 of many)	1.78	3.10	-0.80	0.03
XLOC_013808	ENSONIG00000004342	320.04	0.49	9.35	0.03
XLOC_025010	brwd3	1.81	1.12	0.70	0.03
XLOC_007139	manea	7.11	11.08	-0.64	0.03
XLOC_022265	-	23.90	40.45	-0.76	0.03
XLOC_019619	ufl1	11.93	7.60	0.65	0.03
XLOC_003959	sephs1	6.09	2.22	1.45	0.03
XLOC_019904	asap3	3.03	1.83	0.73	0.03
XLOC_020050	-	47.54	12.48	1.93	0.03
XLOC_025096	si:ch73-12o23.1	14.26	24.55	-0.78	0.03
XLOC_021501	kirrel3l	0.63	1.27	-1.01	0.03
XLOC_023420	-	8.49	2.92	1.54	0.03
XLOC_020367	ulk2	6.91	11.19	-0.70	0.03
XLOC_022884	nucb2b	459.81	179.79	1.35	0.03
XLOC_009839	dlg3	6.71	3.92	0.78	0.03
XLOC_010199	bnip3la	28.09	48.03	-0.77	0.03
XLOC_020831	nup155	5.47	3.15	0.80	0.03
XLOC_022944	trappc2	16.55	8.73	0.92	0.03
XLOC_015290	ENSONIG00000019076	4.49	2.05	1.13	0.03
XLOC_002960	dus2	4.69	2.73	0.78	0.03
XLOC_008595	akr1a1b	15.57	9.85	0.66	0.03
XLOC_014104	gucd1	3.73	1.89	0.98	0.03
XLOC_021719	-	14.98	48.17	-1.69	0.03
XLOC_003671	wdr5	10.33	6.37	0.70	0.03
XLOC_005556	ENSONIG00000005300	0.90	0.29	1.62	0.03
XLOC_009887	ENSONIG00000002525	1.42	2.59	-0.87	0.03
XLOC_016209	-	1.26	2.29	-0.86	0.03
XLOC_020065	TMEM69 (1 of many)	6.23	2.78	1.17	0.03
XLOC_019211	-	0.80	2.68	-1.75	0.03

XLOC_030855	-	3.27	1.11	1.55	0.03
XLOC_018357	lzts2a	0.82	1.53	-0.90	0.03
XLOC_021080	-	0.33	3.37	-3.36	0.03
XLOC_023952	ywhag1	5.07	1.94	1.38	0.03
XLOC_017634	-	68.51	25.10	1.45	0.03
XLOC_020256	tpbgb	0.30	1.39	-2.23	0.03
XLOC_029379	ITM2C (1 of many)	1.12	3.04	-1.43	0.03
XLOC_003044	-	1.65	4.67	-1.50	0.03
XLOC_010704	elk1	0.86	0.31	1.45	0.03
XLOC_029293	-	5.75	0.71	3.01	0.03
XLOC_001988	si:ch211-183d21.1	1.00	2.01	-1.00	0.03
XLOC_003653	man1b1a	0.76	0.26	1.54	0.03
XLOC_012899	-	3.76	0.49	2.94	0.03
XLOC_027144	-	3.21	0.05	5.91	0.03
XLOC_023609	GPR180	1.20	0.13	3.17	0.04
XLOC_025226	-	0.64	3.56	-2.47	0.04
XLOC_006098	-	4.09	1.40	1.55	0.04
XLOC_027939	dcaf7 (1 of many)	1.51	0.34	2.15	0.04
XLOC_018760	slc1a4	4.33	7.84	-0.86	0.04
XLOC_023845	-	57.24	267.29	-2.22	0.04
XLOC_025581	-	7.98	17.40	-1.12	0.04
XLOC_027722	phkg1b	1.42	0.07	4.34	0.04
XLOC_023471	hdx	1.76	3.12	-0.83	0.04
XLOC_025584	-	1.50	3.20	-1.09	0.04
XLOC_026563	tmed4	11.32	5.86	0.95	0.04
XLOC_029711	-	3.27	0.59	2.46	0.04
XLOC_001177	per2	3.25	6.29	-0.95	0.04
XLOC_003260	ENSONIG00000002753	0.37	1.26	-1.77	0.04
XLOC_006350	SAMD14	0.33	0.95	-1.53	0.04
XLOC_009445	nmnat1-rbp7a	15.07	9.45	0.67	0.04
XLOC_026254	ube2c	0.33	1.08	-1.73	0.04
XLOC_002520	tgif1	1.04	2.89	-1.47	0.04
XLOC_005396	aqp7	71.03	42.31	0.75	0.04
XLOC_010578	ENSONIG00000017769	1.94	3.44	-0.83	0.04
XLOC_021656	mrto4	17.71	10.31	0.78	0.04
XLOC_022280	nubp1	16.42	9.93	0.73	0.04
XLOC_003511	pigx	3.06	1.56	0.98	0.04
XLOC_012120	aifm5	0.92	0.41	1.15	0.04
XLOC_003760	-	2.00	5.72	-1.51	0.04
XLOC_009294	amt	17.46	27.72	-0.67	0.04
XLOC_024490	mtus1b	1.27	2.47	-0.96	0.04
XLOC_013397	SEC14L1 (1 of many)	0.77	1.33	-0.79	0.04
XLOC_003028	ranbp10	1.68	2.71	-0.69	0.04
XLOC_003769	nars	25.38	16.01	0.66	0.04
XLOC_020361	appbp2	3.17	1.72	0.88	0.04
XLOC_021748	-	5.55	3.42	0.70	0.04
XLOC_009221	-	0.70	8.54	-3.61	0.04
XLOC_010110	-	54.53	13.83	1.98	0.04

XLOC_019845	tpra	3.47	2.25	0.63	0.04
XLOC_030232	tac1	4.98	2.43	1.04	0.04
XLOC_030813	-	2.28	6.78	-1.57	0.04
XLOC_013466	ENSONIG00000017936	2.86	4.57	-0.68	0.04
XLOC_029005	cyb561d1	0.31	1.01	-1.73	0.04
XLOC_030428	-	1.81	0.26	2.80	0.04
XLOC_016802	DENND2A (1 of many)	1.93	3.16	-0.72	0.04
XLOC_028871	-	0.91	4.24	-2.22	0.04
XLOC_010713	-	2.26	0.73	1.64	0.04
XLOC_011315	-	6.55	1.18	2.47	0.04
XLOC_013377	pla2g12b	70.47	130.24	-0.89	0.04
XLOC_024034	-	2.80	0.89	1.65	0.04
XLOC_029173	-	2.77	0.23	3.58	0.04
XLOC_009421	-	4.72	8.73	-0.89	0.04
XLOC_007582	etfdh	22.42	35.64	-0.67	0.04
XLOC_007090	-	0.37	2.57	-2.81	0.04
XLOC_018598	clockb	2.74	4.32	-0.66	0.04
XLOC_030465	ENSONIG00000008309	0.11	1.92	-4.10	0.04
XLOC_008999	seta	23.50	14.51	0.70	0.04
XLOC_012744	-	0.71	3.19	-2.18	0.04
XLOC_022413	-	1.88	0.74	1.34	0.04
XLOC_007342	poldip3	8.92	5.40	0.72	0.04
XLOC_028563	opa3	12.90	5.94	1.12	0.04
XLOC_024988	-	4.67	0.78	2.59	0.04
XLOC_018537	chic2	5.87	2.71	1.11	0.04
XLOC_006304	-	2.17	4.47	-1.04	0.04
XLOC_015229	-	31.82	7.35	2.11	0.04
XLOC_007858	sec61g	1967.92	1185.26	0.73	0.04
XLOC_013219	MYH9 (1 of many)	0.86	0.48	0.83	0.04
XLOC_016499	taf12	6.44	10.98	-0.77	0.04
XLOC_000731	zdhhc1	0.43	0.95	-1.15	0.04
XLOC_000853	cebpd	132.22	56.13	1.24	0.04
XLOC_007668	pcgf1	4.90	2.30	1.09	0.04
XLOC_022771	-	2.13	4.93	-1.21	0.04
XLOC_027911	capgb	3.96	6.35	-0.68	0.04
XLOC_011520	-	128.62	22.59	2.51	0.04
XLOC_027626	-	4.81	1.11	2.11	0.04
XLOC_003266	dnm3a	0.45	0.99	-1.14	0.04
XLOC_013114	-	0.64	27.28	-5.42	0.04
XLOC_028661	-	0.51	1.52	-1.57	0.04
XLOC_002431	TMCC1 (1 of many)	7.81	4.98	0.65	0.04
XLOC_003345	timm13	5.10	2.14	1.26	0.04
XLOC_008870	RABGAP1	2.37	3.73	-0.65	0.04
XLOC_010049	-	1.31	0.44	1.56	0.04
XLOC_017405	-	0.24	1.33	-2.44	0.04
XLOC_021323	-	5.73	1.34	2.10	0.04
XLOC_002778	pdf	4.71	2.41	0.97	0.04
XLOC_019398	ENSONIG00000012374	5.60	9.19	-0.71	0.04

XLOC_022114	srprb	11.48	7.33	0.65	0.04
XLOC_014651	ENSONIG00000001118	0.21	0.71	-1.79	0.04
XLOC_017253	SEMA6A	0.53	0.96	-0.85	0.04
XLOC_028535	-	2.26	4.68	-1.05	0.04
XLOC_015933	add3a	1.42	2.58	-0.86	0.04
XLOC_012288	fhl3b	2.98	5.57	-0.90	0.04
XLOC_006208	dennd5a	3.16	5.09	-0.69	0.04
XLOC_013411	SLC47A1 (1 of many)	2.63	1.51	0.80	0.04
XLOC_020118	-	1.04	2.21	-1.09	0.04
XLOC_000775	txlng	6.85	11.52	-0.75	0.04
XLOC_003865	traf4a	1.50	2.55	-0.76	0.04
XLOC_007422	-	0.85	2.02	-1.25	0.04
XLOC_018111	-	0.27	2.47	-3.20	0.04
XLOC_006474	-	0.30	3.02	-3.35	0.04
XLOC_008950	grsf1	3.93	2.10	0.90	0.04
XLOC_010174	nol10	2.91	1.68	0.79	0.04
XLOC_010692	gnpda1	3.44	6.05	-0.82	0.04
XLOC_012372	ppt1	10.69	18.48	-0.79	0.04
XLOC_020000	prdx1	24.18	38.11	-0.66	0.04
XLOC_022568	-	0.81	2.48	-1.62	0.04
XLOC_029097	tspan4b	0.72	2.33	-1.70	0.04
XLOC_000947	si:dkeyp-94h10.5	0.91	0.46	0.98	0.04
XLOC_004138	cpt1b	1.99	1.03	0.96	0.04
XLOC_004280	naga	5.18	8.09	-0.64	0.04
XLOC_019484	-	0.92	2.00	-1.12	0.04
XLOC_021190	pmpca	17.37	9.79	0.83	0.04
XLOC_008668	-	0.47	1.92	-2.04	0.04
XLOC_011667	-	0.66	2.22	-1.75	0.04
XLOC_019023	s100a10a	18.00	31.03	-0.79	0.04
XLOC_028542	-	1.60	4.25	-1.41	0.04
XLOC_030739	0000012499,ENSONIG0	5.47	2.38	1.20	0.04
XLOC_009368	kcnc4	1.26	0.62	1.03	0.04
XLOC_024769	-	20.13	0.15	7.11	0.04
XLOC_009150	mbnl3	1.30	2.44	-0.91	0.04
XLOC_018206	ARHGAP9	2.07	6.27	-1.60	0.04
XLOC_029156	-	53.92	27.82	0.95	0.04
XLOC_013694	calcoco1a	3.61	5.66	-0.65	0.04
XLOC_029063	-	1.26	4.62	-1.87	0.04
XLOC_003296	scfd2	5.41	3.31	0.71	0.04
XLOC_004340	cipcb	3.02	1.87	0.69	0.04
XLOC_012641	pnp5b	7.33	3.26	1.17	0.04
XLOC_013691	FAIM2 (1 of many)	2.15	0.53	2.01	0.04
XLOC_001047	300000010631,atp13a3 (1	5.97	3.53	0.76	0.04
XLOC_004946	-	5.86	11.31	-0.95	0.04
XLOC_022511	ENSONIG00000015368	1.03	1.76	-0.78	0.04
XLOC_004476	zgc:103511	0.99	0.44	1.16	0.04
XLOC_003622	ENSONIG00000021051	3.09	1.77	0.80	0.04
XLOC_028707	-	0.08	1.08	-3.69	0.04

XLOC_013443	NTN4 (1 of many)	0.58	1.19	-1.03	0.04
XLOC_027972	-	3.22	10.93	-1.76	0.04
XLOC_029044	-	1.57	5.23	-1.73	0.04
XLOC_003646	ccnb1	0.31	1.18	-1.90	0.04
XLOC_020278	eif3ea	132.68	81.37	0.71	0.04
XLOC_025184	-	0.96	0.33	1.54	0.04
XLOC_026379	-	1.81	0.10	4.11	0.04
XLOC_017256	-	0.59	2.23	-1.91	0.04
XLOC_024574	pex6	27.34	13.73	0.99	0.04
XLOC_009716	-	17.81	30.97	-0.80	0.04
XLOC_017452	sh3bp5la	1.69	2.97	-0.82	0.04
XLOC_027255	nup160	6.28	3.87	0.70	0.04
XLOC_028017	-	5.78	11.28	-0.96	0.04
XLOC_016627	ncapd2	0.63	1.16	-0.88	0.04
XLOC_027465	-	1.46	5.98	-2.03	0.04
XLOC_001218	fam102ba	0.56	1.54	-1.46	0.04
XLOC_030606	-	0.20	2.22	-3.50	0.04
XLOC_012901	ippk	1.06	1.78	-0.75	0.04
XLOC_020965	SLC9A3R1 (1 of many)	18.97	11.72	0.69	0.04
XLOC_022708	gak	1.39	0.79	0.82	0.04
XLOC_005748	entpd1	2.85	5.91	-1.05	0.04
XLOC_006381	-	1.36	2.82	-1.05	0.04
XLOC_008472	-	0.22	1.33	-2.61	0.04
XLOC_001161	phlpp1	1.69	1.03	0.71	0.04
XLOC_013754	nprl2	6.86	16.34	-1.25	0.04
XLOC_015763	-	0.44	1.36	-1.64	0.04
XLOC_019117	prkacba	0.22	0.99	-2.18	0.04
XLOC_011401	ENSONIG00000006516	124.59	202.19	-0.70	0.04
XLOC_029418	NEMF	5.16	3.22	0.68	0.04
XLOC_012109	crcp	8.26	2.72	1.60	0.04
XLOC_014222	-	1.68	3.54	-1.07	0.04
XLOC_018374	ddit4	653.79	3.10	7.72	0.04
XLOC_026137	-	2.23	5.22	-1.23	0.04
XLOC_028751	-	3.56	0.98	1.86	0.04
XLOC_020138	-	3.51	1.75	1.01	0.04
XLOC_001187	ppp1r7	10.04	6.24	0.69	0.04
XLOC_013651	si:ch211-269m15.3	4.04	2.28	0.82	0.04
XLOC_024673	-	1.68	16.06	-3.25	0.04
XLOC_005094	tsfm	9.27	5.69	0.70	0.04
XLOC_023332	ENSONIG00000011795	1.73	5.46	-1.66	0.04
XLOC_025461	alkbh1	1.13	0.48	1.22	0.04
XLOC_030828	-	6.14	1.56	1.98	0.04
XLOC_022557	col4a3bpb	3.02	1.82	0.73	0.04
XLOC_010468	trappc2l	5.39	2.48	1.12	0.04
XLOC_030924	-	13.97	25.89	-0.89	0.04
XLOC_006175	-	1.11	0.27	2.02	0.04
XLOC_014186	-	23.14	8.32	1.48	0.04
XLOC_014970	SNORA71	70.32	0	inf	0.04

XLOC_012297	-	0.84	2.62	-1.64	0.04
XLOC_018713	npnt	0.54	1.07	-0.99	0.04
XLOC_022000	-	4.07	9.95	-1.29	0.04
XLOC_005973	arl5c	3.18	7.80	-1.30	0.04
XLOC_006419	eif1b	364.03	207.40	0.81	0.04
XLOC_014402	gorab	2.60	1.28	1.02	0.04
XLOC_015557	prmt9	5.49	9.17	-0.74	0.04
XLOC_019939	si:dkey-13p1.4	0.33	1.13	-1.76	0.04
XLOC_026109	aldh5a1	10.96	21.44	-0.97	0.04
XLOC_030329	-	1.59	0.14	3.53	0.04
XLOC_004172	nr2f1b (1 of many)	4.88	2.61	0.90	0.04
XLOC_010486	mrpl16	27.45	17.10	0.68	0.04
XLOC_015367	-	2.49	0.32	2.97	0.04
XLOC_007608	NCBP1	4.73	2.50	0.92	0.04
XLOC_004804	-	0.33	1.50	-2.19	0.04
XLOC_007790	gramd1c	5.69	3.45	0.72	0.04
XLOC_012733	f13a1b	2.64	1.09	1.28	0.04
XLOC_013387	QPRT	47.63	74.86	-0.65	0.04
XLOC_018564	pgm2	14.35	9.19	0.64	0.04
XLOC_022921	si:dkey-10o6.2	163.01	65.99	1.30	0.04
XLOC_028144	si:dkey-211g8.4	41.54	79.63	-0.94	0.04
XLOC_006651	-	0.58	1.51	-1.40	0.04
XLOC_010396	mphosph10	6.66	2.48	1.43	0.04
XLOC_023728	-	1.02	2.91	-1.51	0.04
XLOC_000717	-	1.17	2.64	-1.17	0.04
XLOC_023454	mospd1	12.01	7.46	0.69	0.04
XLOC_003066	-	32.10	11.71	1.45	0.04
XLOC_021539	setd2	1.66	1.04	0.68	0.04
XLOC_001370	ENSONIG00000000613	0.04	0.72	-4.09	0.04
XLOC_005413	-	0.80	2.09	-1.38	0.04
XLOC_003882	c2cd3	1.57	2.50	-0.67	0.04
XLOC_005535	-	3.32	7.63	-1.20	0.04
XLOC_019811	C14orf1	1.25	15.97	-3.67	0.04
XLOC_022798	5S_rRNA	1.18	0.21	2.48	0.04
XLOC_027443	PTPRB (1 of many)	6.50	4.18	0.64	0.04
XLOC_030346	-	22.84	128.68	-2.49	0.04
XLOC_012078	slc9a6b	3.28	2.01	0.71	0.04
XLOC_015512	arhgap10	2.97	4.72	-0.67	0.04
XLOC_005405	yif1b	9.10	5.77	0.66	0.04
XLOC_005713	-	3.32	1.04	1.68	0.04
XLOC_006070	-	4.76	10.36	-1.12	0.04
XLOC_006447	MPRIP (1 of many)	0.81	0.25	1.68	0.04
XLOC_010898	dock8	1.10	1.77	-0.69	0.04
XLOC_029046	-	0.51	2.17	-2.09	0.04
XLOC_031027	-	4.64	2.05	1.18	0.04
XLOC_018265	cops8	16.06	10.31	0.64	0.04
XLOC_030418	-	1.54	6.01	-1.97	0.04
XLOC_007436	-	1.28	2.55	-0.99	0.04

XLOC_007438	-	0.35	4.00	-3.52	0.04
XLOC_015562	sh3d19	0.85	0.37	1.19	0.04
XLOC_028877	-	1.11	4.44	-2.00	0.04
XLOC_016900	-	1.84	3.22	-0.81	0.04
XLOC_023516	-	135.47	61.89	1.13	0.04
XLOC_031274	ENSONIG00000020198	512.54	970.56	-0.92	0.04
XLOC_008729	pptc7b	1.83	3.10	-0.76	0.04
XLOC_017715	mybbp1a	3.79	2.42	0.65	0.04
XLOC_018613	grhprb	310.32	515.23	-0.73	0.04
XLOC_022561	wdr41	1.71	3.19	-0.90	0.04
XLOC_024440	tlr22 (1 of many)	3.37	10.39	-1.63	0.04
XLOC_001883	tmem184ba	9.62	6.08	0.66	0.04
XLOC_010109	actn1	2.81	4.79	-0.77	0.04
XLOC_022927	scamp2	12.89	8.31	0.63	0.04
XLOC_012426	-	0.38	25.42	-6.06	0.04
XLOC_013106	tmem39b	6.75	10.70	-0.67	0.04
XLOC_026101	-	5.70	3.21	0.83	0.04
XLOC_014027	acss2l	91.55	154.98	-0.76	0.04
XLOC_020958	spsb3a	0.50	1.28	-1.37	0.04
XLOC_030500	-	1.29	6.14	-2.26	0.04
XLOC_009066	ube2ka	11.69	7.38	0.66	0.04
XLOC_005259	rft1	4.56	2.61	0.81	0.04
XLOC_022482	-	1.31	6.52	-2.32	0.04
XLOC_008455	vps72	5.11	2.75	0.89	0.04
XLOC_029720	-	5.61	2.55	1.14	0.04
XLOC_008687	magoh	56.89	35.12	0.70	0.04
XLOC_015263	-	1.49	0.43	1.79	0.04
XLOC_000551	eprs	16.96	10.91	0.64	0.04
XLOC_011977	mrps31	4.82	2.70	0.83	0.04
XLOC_002744	emc9	10.79	2.71	1.99	0.04
XLOC_010655	ergic1	13.29	2.73	2.28	0.04
XLOC_021750	ENSONIG00000008962	0.16	1.60	-3.30	0.04
XLOC_030665	-	2.72	11.15	-2.04	0.04
XLOC_008104	-	3.94	0.87	2.17	0.04
XLOC_019464	luc7l3	23.83	15.43	0.63	0.04
XLOC_018299	MARCH8	2.86	4.94	-0.79	0.04
XLOC_020200	gtf2ird1	1.04	1.84	-0.82	0.04
XLOC_022896	ckmt1	2.57	4.20	-0.71	0.04
XLOC_027273	nol11	3.69	2.21	0.74	0.04
XLOC_029868	atad1b (1 of many)	0.15	1.35	-3.18	0.04
XLOC_019984	grhl1	0.85	0.32	1.41	0.04
XLOC_013092	-	0.97	0.17	2.48	0.04
XLOC_016598	ube2s	7.92	4.41	0.84	0.04
XLOC_008676	rfc4	1.01	2.43	-1.27	0.04
XLOC_012788	-	9.34	3.73	1.32	0.04
XLOC_029051	ENSONIG00000013102	0.35	2.98	-3.07	0.04
XLOC_006710	dido1	4.47	7.61	-0.77	0.04
XLOC_019401	-	2.09	3.76	-0.85	0.04

XLOC_024311	hagh	16.35	25.05	-0.62	0.04
XLOC_008752	GNAZ	0.70	1.52	-1.12	0.04
XLOC_008606	-	0.61	2.21	-1.86	0.04
XLOC_010738	-	0.47	2.29	-2.28	0.04
XLOC_010865	lmbrd2a	4.38	2.66	0.72	0.04
XLOC_016504	-	1.15	5.51	-2.26	0.04
XLOC_024151	-	1.17	0.25	2.20	0.04
XLOC_002519	uba5	65.30	18.88	1.79	0.04
XLOC_010157	pdss2	2.96	1.39	1.09	0.04
XLOC_029366	-	0.96	4.73	-2.30	0.04
XLOC_030569	-	1.26	6.56	-2.38	0.04
XLOC_002680	LPIN2 (1 of many)	0.45	0.98	-1.11	0.04
XLOC_017473	ENSONIG00000002025	3786.19	8790.64	-1.22	0.04
XLOC_022148	kpna5	7.25	4.55	0.67	0.04
XLOC_024465	-	13.01	0	inf	0.04
XLOC_008317	tmpob	4.65	2.92	0.67	0.04
XLOC_009749	gtpbp3	1.95	0.97	1.00	0.04
XLOC_010454	slc39a13	24.16	14.44	0.74	0.04
XLOC_012684	tesk2	1.18	0.57	1.05	0.04
XLOC_025565	fam133b	5.29	3.29	0.69	0.04
XLOC_011842	prdx4	309.03	192.63	0.68	0.04
XLOC_027278	helz	1.67	1.01	0.72	0.04
XLOC_003444	:h211-212d10.1 (1 of ma	0.62	2.62	-2.08	0.04
XLOC_014216	prr15lb	7.79	17.33	-1.15	0.04
XLOC_025196	zgc:110540	0.56	2.11	-1.91	0.04
XLOC_025637	-	0.24	2.76	-3.51	0.04
XLOC_009628	-	0.35	1.92	-2.44	0.04
XLOC_017482	s100v1	10.27	19.19	-0.90	0.04
XLOC_023870	-	1.55	10.86	-2.81	0.04
XLOC_016354	otub1b	11.79	18.27	-0.63	0.04
XLOC_025333	tegt (1 of many)	554.88	335.71	0.72	0.04
XLOC_025967	-	11.61	20.90	-0.85	0.04
XLOC_011229	tcte1	0.08	1.24	-3.96	0.04
XLOC_012596	zgc:158316	4.18	7.37	-0.82	0.04
XLOC_022054	p2ry12	1.04	1.96	-0.91	0.04
XLOC_026159	irak1	5.20	3.27	0.67	0.04
XLOC_001682	pkd1b	0.14	1.19	-3.06	0.04
XLOC_007412	-	3.62	1.65	1.14	0.04
XLOC_015718	nrip1b	1.42	2.33	-0.72	0.04
XLOC_021846	si:ch73-269m23.5	16.83	10.77	0.64	0.04
XLOC_024936	-	119.35	209.43	-0.81	0.04
XLOC_030545	-	0.74	4.53	-2.61	0.04
XLOC_000299	-	3.87	1.08	1.84	0.04
XLOC_002118	dvl1b	3.46	5.52	-0.67	0.04
XLOC_014981	-	2.19	4.66	-1.09	0.04
XLOC_015087	sars2	3.12	1.67	0.90	0.04
XLOC_030866	cinp	9.90	5.08	0.96	0.04
XLOC_025399	ENSONIG00000017326	0.66	1.73	-1.39	0.04

XLOC_008260	-	1.77	0.50	1.83	0.04
XLOC_011191	-	2.46	0.94	1.38	0.04
XLOC_021654	mrps16	22.39	12.59	0.83	0.04
XLOC_028861	-	138.37	6.81	4.34	0.04
XLOC_005460	tnfrsf19	0.43	1.10	-1.35	0.04
XLOC_006693	-	15.88	2.21	2.84	0.04
XLOC_007012	-	1.35	5.07	-1.91	0.04
XLOC_013398	dgke	1.86	3.29	-0.82	0.04
XLOC_015171	zgc:194930	9.66	5.82	0.73	0.04
XLOC_020067	aldh9a1b	22.29	14.05	0.67	0.04
XLOC_030422	ENSONIG00000009124	1.61	3.13	-0.96	0.04
XLOC_007107	GADD45B (1 of many)	0.27	0.89	-1.73	0.04
XLOC_017045	-	6.55	2.71	1.27	0.04
XLOC_028273	tmem69	3.62	1.66	1.13	0.04
XLOC_019263	cdc42l (1 of many)	3.77	5.87	-0.64	0.04
XLOC_014983	-	4.02	20.58	-2.36	0.04
XLOC_020833	ENSONIG00000014060	0.43	0.74	-0.80	0.04
XLOC_028123	-	1.55	11.56	-2.90	0.04
XLOC_005339	-	3.55	7.51	-1.08	0.04
XLOC_021499	-	0.14	0.86	-2.60	0.04
XLOC_030478	aars2	6.03	3.86	0.64	0.04
XLOC_004619	-	0.85	4.82	-2.51	0.04
XLOC_007923	trak1	1.64	0.75	1.13	0.04
XLOC_011783	ENSONIG00000004748	1.15	2.24	-0.96	0.04
XLOC_006762	bin2b	5.08	9.99	-0.97	0.04
XLOC_010161	-	5.33	0	inf	0.04
XLOC_014067	-	13.48	7.57	0.83	0.04
XLOC_017872	dcbld2	13.63	8.53	0.68	0.04
XLOC_007542	hdac4	3.57	5.55	-0.64	0.04
XLOC_015754	METTL24	0.28	0.84	-1.57	0.04
XLOC_017922	ENSONIG00000013299	1.19	0.66	0.85	0.04
XLOC_022965	-	5.13	3.10	0.72	0.04
XLOC_024875	cdh12a	0.24	0.80	-1.71	0.04
XLOC_027570	-	3.03	12.85	-2.08	0.04
XLOC_016738	tpi1b	95.75	167.85	-0.81	0.04
XLOC_015577	frg1	7.44	12.68	-0.77	0.04
XLOC_022427	-	1.78	0.51	1.81	0.04
XLOC_025592	-	2.86	0.41	2.82	0.04
XLOC_002076	NR4A1 (1 of many)	0.93	0.41	1.19	0.04
XLOC_002227	DAG1 (1 of many)	6.65	10.23	-0.62	0.04
XLOC_024157	-	2.64	0.39	2.75	0.04
XLOC_000598	rbb4l	35.68	19.97	0.84	0.04
XLOC_018550	-	0.53	2.58	-2.28	0.04
XLOC_021137	-	0.32	3.99	-3.63	0.04
XLOC_026781	-	26.44	1.32	4.32	0.04
XLOC_029174	-	5.57	1.42	1.97	0.04
XLOC_003522	exoc1	4.96	7.73	-0.64	0.04
XLOC_006646	-	0.39	1.44	-1.88	0.04

XLOC_025342	LIMA1 (1 of many)	8.85	5.38	0.72	0.04
XLOC_008716	zbtb11	2.64	1.59	0.74	0.04
XLOC_009892	upf3b	11.89	7.59	0.65	0.04
XLOC_024964	mpdu1a	3.03	4.73	-0.64	0.04
XLOC_006755	ubap2a	4.05	6.27	-0.63	0.04
XLOC_031196	-	10.98	2.20	2.32	0.04
XLOC_001575	-	5.45	10.96	-1.01	0.04
XLOC_012616	-	42.95	27.19	0.66	0.04
XLOC_018389	cry1aa	10.22	6.59	0.63	0.04
XLOC_025246	-	1.16	0.16	2.89	0.04
XLOC_000546	si:ch211-276a23.5	3.14	4.97	-0.66	0.04
XLOC_020821	gal3st1b	29.39	1.30	4.49	0.04
XLOC_023826	SNORA53	3.11	0	inf	0.04
XLOC_002593	cdk13	4.40	2.84	0.63	0.04
XLOC_002952	-	3.22	0.68	2.24	0.04
XLOC_012755	rbm14b	1.14	2.30	-1.01	0.04
XLOC_004310	-	0.79	3.60	-2.18	0.04
XLOC_020666	prdx2	217.05	457.36	-1.08	0.04
XLOC_025150	-	1.71	0.34	2.32	0.04
XLOC_005052	strip1	3.07	1.80	0.77	0.04
XLOC_021576	si:rp71-84d9.1	0.14	1.03	-2.86	0.04
XLOC_030038	-	0.89	2.58	-1.53	0.04
XLOC_029081	-	2.10	1.19	0.81	0.04
XLOC_000005	-	1.56	0.08	4.31	0.04
XLOC_005229	dstyk	1.83	3.35	-0.87	0.04
XLOC_028675	ENSONIG00000007819	0.08	0.96	-3.55	0.04
XLOC_027691	rnf139	4.43	2.77	0.68	0.04
XLOC_010120	-	7.73	18.31	-1.24	0.04
XLOC_009759	-	0.67	3.20	-2.26	0.04
XLOC_003009	arntl1a	1.59	2.52	-0.66	0.04
XLOC_004537	-	3.21	8.15	-1.34	0.04
XLOC_017227	-	6.85	11.30	-0.72	0.04
XLOC_004672	rnd3b	5.25	3.31	0.67	0.04
XLOC_020139	tmem101	4.33	2.20	0.98	0.04
XLOC_031437	-	2.56	0.26	3.30	0.04
XLOC_013621	phlda3	0.42	1.49	-1.84	0.04
XLOC_016747	znf526	2.06	1.21	0.77	0.04
XLOC_029889	-	0.67	9.23	-3.78	0.04
XLOC_014732	ctdspl3	10.93	6.76	0.69	0.04
XLOC_019124	ncbp3	19.01	12.36	0.62	0.04
XLOC_021458	fkbp14	6.85	3.67	0.90	0.04
XLOC_030651	-	192.66	337.13	-0.81	0.04
XLOC_000070	ENSONIG00000010892	0.25	1.04	-2.04	0.04
XLOC_009504	-	0.36	3.44	-3.27	0.04
XLOC_010983	-	1.40	13.32	-3.25	0.04
XLOC_012500	-	0.37	3.08	-3.05	0.04
XLOC_019171	inpp5l	0.16	1.09	-2.78	0.04
XLOC_012830	slc25a48	19.82	35.42	-0.84	0.04

XLOC_022116	-	0.13	1.09	-3.11	0.04
XLOC_029664	si:dkey-13e3.1	2.07	0.83	1.32	0.04
XLOC_012905	nol8	1.44	0.84	0.78	0.04
XLOC_025620	sirt5	7.82	5.02	0.64	0.04
XLOC_002072	-	34.56	21.30	0.70	0.04
XLOC_015676	arhgap32b	4.27	2.80	0.61	0.04
XLOC_022767	-	0.27	1.30	-2.26	0.04
XLOC_027759	crybb1l3	1.74	0.71	1.29	0.04
XLOC_003518	paics	33.53	21.28	0.66	0.04
XLOC_006570	-	3.95	1.09	1.86	0.04
XLOC_008445	ptpn2b	5.95	10	-0.75	0.04
XLOC_017941	-	1.79	0.59	1.59	0.04
XLOC_022707	-	9.75	2.07	2.23	0.04
XLOC_023479	ENSONIG00000017684	2.15	0.65	1.72	0.04
XLOC_005009	ret	12.51	19.19	-0.62	0.04
XLOC_024278	fahd1	10.54	17.62	-0.74	0.04
XLOC_017995	-	0.84	0.06	3.91	0.04
XLOC_025142	-	1.20	0.28	2.09	0.04
XLOC_026495	spint2	3.67	5.80	-0.66	0.04
XLOC_027897	YBX2	25.59	39.79	-0.64	0.04
XLOC_003802	alad	29.00	19.01	0.61	0.04
XLOC_013851	-	0.45	2.25	-2.34	0.04
XLOC_016967	-	221.66	123.47	0.84	0.04
XLOC_007227	-	0.39	1.42	-1.86	0.04
XLOC_012485	glb1l	11.37	18.77	-0.72	0.04
XLOC_020281	ebag9	17.76	11.12	0.68	0.04
XLOC_000951	alg3	8.08	4.78	0.76	0.04
XLOC_014559	-	2.48	1.07	1.21	0.04
XLOC_017474	-	0.77	3.27	-2.09	0.04
XLOC_004095	-	0.39	1.43	-1.85	0.04
XLOC_007785	-	9.20	18.77	-1.03	0.04
XLOC_021325	igbp1	13.92	8.73	0.67	0.04
XLOC_026904	-	1.22	2.64	-1.12	0.04
XLOC_000782	zgc:162297	6.30	10.72	-0.77	0.04
XLOC_009884	-	3.08	7.27	-1.24	0.04
XLOC_020686	-	1.98	4.91	-1.31	0.04
XLOC_027064	rela	26.64	17.27	0.63	0.04
XLOC_016656	ENSONIG00000013024	0.45	1.26	-1.48	0.04
XLOC_017286	-	1.70	0.17	3.31	0.04
XLOC_009846	-	1.78	3.49	-0.97	0.04
XLOC_025351	-	12.61	1.27	3.31	0.04
XLOC_031121	-	4.90	1.40	1.81	0.04
XLOC_003270	si:ch73-215a11.1	1.83	1.00	0.87	0.04
XLOC_020205	-	14.90	22.94	-0.62	0.04
XLOC_009117	pmt	78.28	141.11	-0.85	0.04
XLOC_003236	CYP2J2 (1 of many)	89.33	143.11	-0.68	0.05
XLOC_007553	-	55.90	5.00	3.48	0.05
XLOC_025463	c17h14orf159	27.86	16.99	0.71	0.05

XLOC_019142	rassf7b,rdh14a	2.50	1.27	0.97	0.05
XLOC_022128	WDR26 (1 of many)	2.06	3.99	-0.95	0.05
XLOC_025612	irf4b	0.03	0.85	-5.03	0.05
XLOC_002205	ENSONIG00000017255	51.34	1.97	4.70	0.05
XLOC_005117	-	0.78	3.93	-2.33	0.05
XLOC_012385	si:ch211-195b13.6	2.62	4.28	-0.71	0.05
XLOC_026706	-	3.73	1.73	1.11	0.05
XLOC_017420	-	57.19	87.42	-0.61	0.05
XLOC_025120	slc25a36b (1 of many)	9.44	14.88	-0.66	0.05
XLOC_029322	-	8.07	1.12	2.84	0.05
XLOC_003775	-	8.04	4.86	0.73	0.05
XLOC_005095	ENSONIG00000019103	0.24	1.51	-2.64	0.05
XLOC_005613	ENSONIG00000015852	0.52	1.00	-0.96	0.05
XLOC_007020	-	0.42	1.63	-1.96	0.05
XLOC_011212	grcc10	14.84	5.05	1.56	0.05
XLOC_011373	pex12	9.58	16.37	-0.77	0.05
XLOC_031424	-	12.07	20.75	-0.78	0.05
XLOC_021332	enpp6	0.32	0.97	-1.60	0.05
XLOC_011408	traf7	3.12	4.90	-0.65	0.05
XLOC_012801	ENSONIG00000012989	0.47	1.51	-1.69	0.05
XLOC_031165	ENSONIG00000004813	52.04	0.16	8.38	0.05
XLOC_014386	rtca	8.37	5.35	0.65	0.05
XLOC_022832	cthl	127.17	77.87	0.71	0.05
XLOC_000618	tspan3b	0.91	1.63	-0.83	0.05
XLOC_008152	sergef	2.68	1.48	0.85	0.05
XLOC_016249	-	5.89	0.69	3.10	0.05
XLOC_018813	-	0.53	3.40	-2.68	0.05
XLOC_021000	lrrc45	2.68	1.58	0.76	0.05
XLOC_026830	ENSONIG00000022110	39.08	22.34	0.81	0.05
XLOC_011582	-	1.14	3.27	-1.52	0.05
XLOC_017916	-	1.90	0.85	1.16	0.05
XLOC_027890	tmem254	46.58	30.61	0.61	0.05
XLOC_012240	srgap1a	2.52	4.29	-0.76	0.05
XLOC_022851	-	99.18	61.87	0.68	0.05
XLOC_009693	-	3.35	6.84	-1.03	0.05
XLOC_014924	-	2.28	0.15	3.92	0.05
XLOC_012753	-	427.35	7.82	5.77	0.05
XLOC_021405	gch1	33.25	56.42	-0.76	0.05
XLOC_001250	itga6b	0.77	0.33	1.22	0.05
XLOC_004658	-	1.18	4.75	-2.01	0.05
XLOC_013203	stx8	7.33	11.55	-0.66	0.05
XLOC_017545	notch2	1.49	2.32	-0.64	0.05
XLOC_029873	SOS1	10.00	6.50	0.62	0.05
XLOC_025658	ENSONIG00000004025	0.45	1.26	-1.47	0.05
XLOC_028848	-	14.31	6.03	1.25	0.05
XLOC_008409	azin1b	35.78	61.68	-0.79	0.05
XLOC_009792	-	1.69	0.71	1.25	0.05
XLOC_022875	rnf141	2.71	4.99	-0.88	0.05

XLOC_027668	ENSONIG00000007364	8.14	5.35	0.61	0.05
XLOC_002666	-	24.76	7.29	1.76	0.05
XLOC_008556	spag1a	1.34	0.43	1.65	0.05
XLOC_022843	rnf13	12.28	18.91	-0.62	0.05
XLOC_024379	-	0.28	1.94	-2.82	0.05
XLOC_014974	zc3h18	6.51	4.03	0.69	0.05
XLOC_019797	aldh6a1 (1 of many)	54.90	89.38	-0.70	0.05
XLOC_012088	ENSONIG00000008565	1.76	2.94	-0.74	0.05
XLOC_017938	-	0.89	3.79	-2.09	0.05
XLOC_013037	-	19.85	8.23	1.27	0.05
XLOC_007031	galnt16	43.15	68.52	-0.67	0.05
XLOC_017891	uchl3	16.17	10.51	0.62	0.05
XLOC_021335	hars	16.58	10.76	0.62	0.05
XLOC_029262	ENSONIG00000020466	3.24	6.75	-1.06	0.05
XLOC_013181	ENSONIG00000021228	15524.50	5478.55	1.50	0.05
XLOC_023275	cog5	55.14	21.72	1.34	0.05
XLOC_004515	prkrip1	9.11	4.56	1.00	0.05
XLOC_010149	si:dkeyp-33b5.4	3.97	1.95	1.03	0.05
XLOC_016315	CAAP1	2.10	1.14	0.88	0.05
XLOC_028159	ponzr1 (1 of many)	79.36	121.47	-0.61	0.05
XLOC_004956	ENSONIG00000009945	0.73	1.27	-0.80	0.05
XLOC_023066	ENSONIG00000000802	2.51	1.51	0.74	0.05
XLOC_011781	-	1.91	3.48	-0.87	0.05
XLOC_014111	cux2b	0.40	0.70	-0.80	0.05
XLOC_018531	cep76	0.39	0.94	-1.26	0.05
XLOC_021920	-	22.09	4.51	2.29	0.05
XLOC_026025	)0000005520,SNORD49	11.10	4.79	1.21	0.05
XLOC_003582	ENSONIG00000012618	1.91	0.97	0.98	0.05
XLOC_022938	klhl6	0.86	1.80	-1.07	0.05
XLOC_006094	slc27a1a	1.39	2.32	-0.74	0.05
XLOC_024012	-	0.39	3.19	-3.02	0.05
XLOC_018574	rhoh	0.32	1.28	-2.01	0.05
XLOC_027325	osgepl1	2.31	0.99	1.22	0.05
XLOC_006251	pdcd5,urah	67.01	38.27	0.81	0.05
XLOC_024263	-	5.62	2.43	1.21	0.05
XLOC_001117	gata6	25.80	15.92	0.70	0.05
XLOC_023321	-	4.73	8.10	-0.78	0.05
XLOC_028814	-	1.96	7.08	-1.85	0.05
XLOC_030896	-	5.56	2.67	1.06	0.05
XLOC_023052	kazald3	179.76	390.32	-1.12	0.05
XLOC_026565	-	14.40	8.38	0.78	0.05
XLOC_011892	thsd1	1.53	2.58	-0.76	0.05
XLOC_020318	brinp3a.1	1.14	2.02	-0.83	0.05
XLOC_007893	-	3.66	1.47	1.31	0.05
XLOC_020651	TMC7	6.60	10.12	-0.62	0.05
XLOC_003490	zyg11	1.36	2.73	-1.00	0.05
XLOC_005447	-	7.68	13.96	-0.86	0.05
XLOC_001053	zgc:55943	0.50	1.26	-1.33	0.05

XLOC_014583	-	2.57	7.14	-1.47	0.05
XLOC_021952	-	37.48	3.33	3.49	0.05
XLOC_011222	extl3	1.71	1.04	0.71	0.05
XLOC_015975	ENSONIG00000016464	35.83	13.94	1.36	0.05
XLOC_016216	GLCCI1	1.59	0.75	1.10	0.05
XLOC_020440	-	0.68	2.50	-1.87	0.05
XLOC_021674	POC1A	1.58	0.45	1.83	0.05
XLOC_028613	ENSONIG00000007122	0.04	1.17	-4.98	0.05
XLOC_000359	-	3.88	1.03	1.92	0.05
XLOC_011037	marcksl1a	9.35	14.52	-0.63	0.05
XLOC_003091	TPM2	1.15	0.37	1.64	0.05
XLOC_013825	-	7.54	2.11	1.84	0.05
XLOC_014712	-	0.25	2.35	-3.26	0.05
XLOC_018139	ENSONIG00000015842	1.06	1.93	-0.87	0.05
XLOC_023824	ldhba	26.64	40.93	-0.62	0.05
XLOC_019030	znf687b	1.19	0.71	0.76	0.05
XLOC_002259	TP53INP2	0.92	5.33	-2.53	0.05
XLOC_029694	-	0.26	1.01	-1.93	0.05
XLOC_028865	-	2.06	0.64	1.69	0.05
XLOC_011219	ENSONIG00000005882	4.69	7.31	-0.64	0.05
XLOC_028579	-	14.72	7.04	1.06	0.05
XLOC_010116	pcnx	2.01	3.08	-0.62	0.05
XLOC_015815	acp1	56.05	36.41	0.62	0.05
XLOC_022112	lxn	68.19	33.23	1.04	0.05
XLOC_022131	-	16.56	2.00	3.05	0.05
XLOC_029604	focad	3.82	2.03	0.91	0.05
XLOC_004603	ENSONIG00000012142	1.25	2.45	-0.97	0.05
XLOC_007810	armc1	13.99	9.23	0.60	0.05
XLOC_027336	gpr39	15.52	10.02	0.63	0.05
XLOC_000348	-	19.09	3.98	2.26	0.05
XLOC_008263	arpp19b	8.62	5.16	0.74	0.05
XLOC_006247	def8	4.18	6.68	-0.68	0.05
XLOC_009971	si:ch1073-55a19.2	3.87	2.53	0.61	0.05
XLOC_017829	ENSONIG00000020481	0.63	1.76	-1.49	0.05
XLOC_023356	SONIG00000011800,cep	4.48	7.15	-0.68	0.05
XLOC_002249	slc9a8	4.17	2.46	0.76	0.05
XLOC_004980	IRF2BP2 (1 of many)	23.31	35.53	-0.61	0.05
XLOC_005499	-	0.39	1.99	-2.36	0.05
XLOC_002565	ENSONIG00000006237	0.41	15.28	-5.23	0.05
XLOC_009242	stx5a	25.69	12.84	1.00	0.05
XLOC_009868	COPS6	8.76	5.54	0.66	0.05
XLOC_019933	-	1.83	5.37	-1.56	0.05
XLOC_025101	-	0.85	3.80	-2.16	0.05
XLOC_001995	spsb3b	0.47	1.11	-1.23	0.05
XLOC_001078	ENSONIG00000009173	42.43	27.84	0.61	0.05
XLOC_029787	-	4.80	1.69	1.51	0.05
XLOC_003415	fam32a	169.27	106.86	0.66	0.05
XLOC_017041	ADAMTSL4	9.98	15.29	-0.62	0.05

XLOC_031000	ENSONIG00000006401	0.19	0.76	-2.02	0.05
XLOC_009506	-	40.41	26.25	0.62	0.05
XLOC_000616	amfr	20.60	31.49	-0.61	0.05
XLOC_008551	-	3.97	0.47	3.09	0.05
XLOC_017155	agpat9l	1.40	2.35	-0.74	0.05
XLOC_021753	-	6.56	2.15	1.61	0.05
XLOC_024706	rnpc3	3.91	6.44	-0.72	0.05
XLOC_013315	-	1.73	0.81	1.09	0.05
XLOC_022289	-	0.52	1.32	-1.35	0.05
XLOC_027459	-	26.87	5.25	2.35	0.05
XLOC_031406	-	21.59	9.74	1.15	0.05
XLOC_021763	IKZF3	2.37	5.45	-1.20	0.05
XLOC_030497	-	1.29	3.89	-1.59	0.05
XLOC_005495	rnf7	57.37	34.81	0.72	0.05
XLOC_021197	-	0.43	1.60	-1.90	0.05
XLOC_022756	ubiad1	8.58	5.48	0.65	0.05
XLOC_023495	mri1	1.89	1.10	0.79	0.05
XLOC_025738	hps3	1.46	2.44	-0.74	0.05
XLOC_026602	-	2.03	0.57	1.82	0.05
XLOC_011461	-	0.49	4.51	-3.21	0.05
XLOC_022863	adgrl4	11.79	7.72	0.61	0.05
XLOC_024728	-	1.25	3.63	-1.54	0.05
XLOC_001306	gk5	16.39	24.94	-0.61	0.05
XLOC_011197	clu	1.19	0.51	1.22	0.05
XLOC_029306	-	3.37	5.41	-0.68	0.05
XLOC_008501	crtap	3.11	1.47	1.08	0.05
XLOC_011349	nfe2l1a	1.31	0.67	0.96	0.05
XLOC_031323	-	0.81	2.54	-1.65	0.05
XLOC_005352	si:ch211-160o17.6	3.49	0.94	1.90	0.05
XLOC_003243	-	0	8.91	-inf	0.05
XLOC_025290	-	0.20	1.14	-2.47	0.05
XLOC_014989	-	0.87	4.08	-2.23	0.05
XLOC_016286	-	4.26	7.39	-0.79	0.05
XLOC_031389	-	8.02	2.14	1.90	0.05
XLOC_006329	dagla	0.56	1.15	-1.05	0.05
XLOC_010518	-	0.42	2.19	-2.37	0.05
XLOC_014735	dpp9	7.64	5.02	0.61	0.05
XLOC_025094	-	1.54	3.53	-1.20	0.05
XLOC_003368	abl2	12.96	0.69	4.24	0.05
XLOC_015680	capns1b	6.53	9.88	-0.60	0.05
XLOC_020284	ube3d	2.62	1.34	0.97	0.05
XLOC_011023	srrm1	15.92	24.37	-0.61	0.05
XLOC_018219	ENSONIG00000005237	0.98	0.28	1.83	0.05
XLOC_028802	-	4.75	1.64	1.54	0.05
XLOC_029260	sbds	17.89	11.05	0.69	0.05
XLOC_012682	-	3.16	7.04	-1.15	0.05
XLOC_016721	-	1.50	0.48	1.65	0.05
XLOC_021804	-	20.13	3.35	2.59	0.05

XLOC_007865	mtmr6	7.19	10.99	-0.61	0.05
XLOC_009737	pla2g4ab	1.42	2.32	-0.71	0.05
XLOC_010016	heg1	2.42	3.77	-0.64	0.05
XLOC_022363	-	3.00	7.26	-1.27	0.05
XLOC_023899	-	0	11.67	-inf	0.05
XLOC_024742	pcca	15.77	10.42	0.60	0.05
XLOC_029426	-	0.16	2.96	-4.18	0.05
XLOC_030092	ENSONIG00000007648	2.54	8.47	-1.74	0.05
XLOC_030940	-	5.39	1.69	1.67	0.05
XLOC_031442	-	0.79	14.82	-4.23	0.05
XLOC_004827	ntpcr	5.41	2.95	0.88	0.05
XLOC_028711	-	0.41	2.25	-2.46	0.05
XLOC_017889	irg1	2.15	3.86	-0.84	0.05
XLOC_020805	-	0.16	2.28	-3.82	0.05
XLOC_025716	cyp26b1	0.03	0.92	-5.07	0.05

Table S9 List of miRNA-mRNA pairs with positive and negative correlation

miR_name	miR_seq	regulation	Accession	regulation
PC-3p-50929_43	TGGAAGTGTCAGAAATTCTGAGT	up	XLOC_000073	down
ola-miR-146a-5p_1ss24TA	TGAGAACTGAATTCCATAGATGGAA	up	XLOC_000073	down
ssa-miR-7a-5p	TGGAAGACTAGTGATTTTGTTGT	up	XLOC_000073	down
ssa-miR-7a-5p_R+1	TGGAAGACTAGTGATTTTGTTGTT	up	XLOC_000073	down
ola-miR-194-3p_1ss20CT	CCAGTGGAGGTGCTGTTACTTG	up	XLOC_000103	down
PC-5p-27517_164	TACATGCAGAGGTGGAGCAAGA	up	XLOC_000118	up
ssa-miR-16b-5p_R-1_1ss21TC	TAGCAGCACGTAAATATTGGC	down	XLOC_000135	up
aca-miR-338-3p_R+2	TCCAGCATCAGTGATTTTGTTAA	up	XLOC_000168	down
dre-miR-140-3p_L-1	ACCACAGGGTAGAACCACGGAC	up	XLOC_000168	down
ola-miR-194-3p_1ss20CT	CCAGTGGAGGTGCTGTTACTTG	up	XLOC_000168	down
ola-miR-146a-5p_1ss24TA	TGAGAACTGAATTCCATAGATGGAA	up	XLOC_000181	down
dre-miR-24_R+2_1	TGGCTCAGTTCAGCAGGAACAGAA	up	XLOC_000249	down
dre-miR-24_R+2_2	TGGCTCAGTTCAGCAGGAACAGTT	up	XLOC_000249	down
aca-miR-200b-3p_R+2	TAATACTGCCTGGTAATGATGAAT	up	XLOC_000305	up
PC-5p-45063_62	AAGGATAACTACAACTGTACTT	up	XLOC_000321	down
dre-let-7d-5p	TGAGGTAGTTGGTTGTATGGTT	up	XLOC_000321	down
dre-miR-133a-3p_L-1R+1	TTGGTCCCCTTCAACCAGCTGT	up	XLOC_000321	down
dre-miR-22a-3p	AAGCTGCCAGCTGAAGAACTGT	up	XLOC_000321	down
mmu-let-7j_1ss8TG	TGAGGTAGTAGTTTGTGCTGTTAT	up	XLOC_000321	down
ssa-miR-7132b-3p	TGAGGCGTTTAGAACAAGTTCA	down	XLOC_000321	down
tni-let-7j_1ss11TG	TGAGGTAGTTGTTTGTACAGTT	up	XLOC_000321	down
PC-5p-27517_164	TACATGCAGAGGTGGAGCAAGA	up	XLOC_000343	up
ola-miR-194-3p_1ss20CT	CCAGTGGAGGTGCTGTTACTTG	up	XLOC_000520	up
ola-miR-199a-3p_L+1	AACAGTAGTCTGCACATTGGTTA	up	XLOC_000520	up
ssa-miR-7a-5p	TGGAAGACTAGTGATTTTGTTGT	up	XLOC_000622	up
ssa-miR-7a-5p_R+1	TGGAAGACTAGTGATTTTGTTGTT	up	XLOC_000622	up
dre-miR-22a-3p	AAGCTGCCAGCTGAAGAACTGT	up	XLOC_000634	down
dre-miR-22a-3p	AAGCTGCCAGCTGAAGAACTGT	up	XLOC_000641	up
ssa-miR-199a-3p_R+2	ACAGTAGTCTGCACATTGGTTTT	up	XLOC_000719	down
dre-miR-122	TGGAGTGTGACAATGGTGTTTG	up	XLOC_000725	down
ssa-miR-7a-5p	TGGAAGACTAGTGATTTTGTTGT	up	XLOC_000794	down
ssa-miR-7a-5p_R+1	TGGAAGACTAGTGATTTTGTTGTT	up	XLOC_000794	down
ssa-miR-1-4-5p	ACATACTTCTTTATATGCCCATA	up	XLOC_000891	up
dre-miR-194a_R+2	TGTAACAGCAACTCCATGTGGAT	up	XLOC_000897	down
tni-miR-194_R+1	TGTAACAGCAACTCCATGTGGA	up	XLOC_000897	down
PC-3p-11630_419	ATGAGGAAAAGAAGTTAGGAGA	down	XLOC_000907	down
rno-miR-122-5p_L+3	ATCTGGAGTGTGACAATGGTGTTTG	up	XLOC_000907	down
dre-miR-125b-5p_R+1	TCCCTGAGACCCTAACTTGTGAT	up	XLOC_000921	up
sha-miR-125a_R+2	TCCCTGAGACCCTAACTTGTGAAA	up	XLOC_000921	up
ssa-miR-125b-5p_R-1	TCCCTGAGACCCTTAACCTGTG	up	XLOC_000921	up
ssa-miR-7a-5p	TGGAAGACTAGTGATTTTGTTGT	up	XLOC_000921	up
ssa-miR-7a-5p_R+1	TGGAAGACTAGTGATTTTGTTGTT	up	XLOC_000921	up
dre-miR-194a_R+2	TGTAACAGCAACTCCATGTGGAT	up	XLOC_000922	up
dre-miR-21_1ss23CA	TAGCTTATCAGACTGGTGTTGGA	up	XLOC_000922	up
ssa-miR-26a-4-3p	CCTATTCTTGATTACTTGTTTC	down	XLOC_000922	up
ssa-miR-7132a-5p_R+1	GACTTGGTCAAAGCTCCTCAGTT	down	XLOC_000922	up
ssa-miR-7132b-5p	GACTTGGTCAAAGCTCCTCAGC	down	XLOC_000922	up
tni-miR-194_R+1	TGTAACAGCAACTCCATGTGGA	up	XLOC_000922	up
aca-miR-338-3p_R+2	TCCAGCATCAGTGATTTTGTTAA	up	XLOC_001006	up

dre-miR-142a-5p	CATAAAGTAGAAAGCACTACT	down	XLOC_001006	up
ola-miR-194-3p_1ss20CT	CCAGTGGAGGTGCTGTTACTTG	up	XLOC_001006	up
ola-mir-100-2-p3	CAAGCTCGTATCTATAGGTATG	down	XLOC_001006	up
rno-miR-122-5p_L+3	ATCTGGAGTGTGACAATGGTGTTTG	up	XLOC_001006	up
ssa-miR-730a-5p_R-1	TCCTCATTGTGCATGCTGTGT	down	XLOC_001006	up
dre-miR-140-3p_L-1	ACCACAGGGTAGAACCACGGAC	up	XLOC_001052	up
dre-miR-194a_R+2	TGTAACAGCAACTCCATGTGGAT	up	XLOC_001052	up
tni-miR-194_R+1	TGTAACAGCAACTCCATGTGGA	up	XLOC_001052	up
PC-5p-27517_164	TACATGCAGAGGTGGAGCAAGA	up	XLOC_001058	up
PC-5p-27517_164	TACATGCAGAGGTGGAGCAAGA	up	XLOC_001090	up
dre-miR-24_R+2_1	TGGCTCAGTTCAGCAGGAACAGAA	up	XLOC_001090	up
dre-miR-24_R+2_2	TGGCTCAGTTCAGCAGGAACAGTT	up	XLOC_001090	up
rno-miR-122-5p_L+3	ATCTGGAGTGTGACAATGGTGTTTG	up	XLOC_001090	up
ola-miR-462_L-1R+4	TAACGGAACCCATAATGCAGCT	down	XLOC_001104	up
ssa-miR-1-4-5p	ACATACTTCTTTATATGCCCATA	up	XLOC_001104	up
PC-3p-11630_419	ATGAGGAAAAGAAGTTAGGAGA	down	XLOC_001106	down
PC-3p-50929_43	TGGAAGTGTCAGAAATTCTGAGT	up	XLOC_001106	down
PC-5p-27517_164	TACATGCAGAGGTGGAGCAAGA	up	XLOC_001106	down
ssa-miR-16b-5p_R-1_1ss21TC	TAGCAGCACGTAAATATTGGC	down	XLOC_001106	down
xtr-miR-122_L+1R-1	CTGGAGTGTGACAATGGTGTTTG	up	XLOC_001106	down
aca-miR-338-3p_R+2	TCCAGCATCAGTGATTTTGTTAA	up	XLOC_001120	down
rno-miR-122-5p_L+3	ATCTGGAGTGTGACAATGGTGTTTG	up	XLOC_001120	down
ssa-miR-730a-5p_R-1	TCCTCATTGTGCATGCTGTGT	down	XLOC_001120	down
PC-5p-27517_164	TACATGCAGAGGTGGAGCAAGA	up	XLOC_001134	down
dre-miR-122	TGGAGTGTGACAATGGTGTTTG	up	XLOC_001134	down
ola-miR-194-3p_1ss20CT	CCAGTGGAGGTGCTGTTACTTG	up	XLOC_001134	down
ssa-miR-16b-5p_R-1_1ss21TC	TAGCAGCACGTAAATATTGGC	down	XLOC_001134	down
ssa-miR-730a-5p_R-1	TCCTCATTGTGCATGCTGTGT	down	XLOC_001134	down
dre-miR-22a-3p	AAGCTGCCAGCTGAAGAACTGT	up	XLOC_001144	down
ssa-miR-7a-5p	TGGAAGACTAGTGATTTTGTTGT	up	XLOC_001144	down
ssa-miR-7a-5p_R+1	TGGAAGACTAGTGATTTTGTTGTT	up	XLOC_001144	down
ssa-miR-16b-5p_R-1_1ss21TC	TAGCAGCACGTAAATATTGGC	down	XLOC_001181	up
ssa-miR-199a-3p_R+2	ACAGTAGTCTGCACATTGGTTTT	up	XLOC_001190	up
ola-miR-199a-3p_L+1	AACAGTAGTCTGCACATTGGTTA	up	XLOC_001194	up
ssa-miR-199a-3p_R+2	ACAGTAGTCTGCACATTGGTTTT	up	XLOC_001194	up
ssa-miR-26d-5p_L+1_1ss13TC	CTTCAAGTAATCCAGGATAGGCT	up	XLOC_001194	up
ssa-miR-199a-3p_R+2	ACAGTAGTCTGCACATTGGTTTT	up	XLOC_001259	down
dre-miR-22a-3p	AAGCTGCCAGCTGAAGAACTGT	up	XLOC_001289	down
ssa-miR-7a-5p	TGGAAGACTAGTGATTTTGTTGT	up	XLOC_001289	down
ssa-miR-7a-5p_R+1	TGGAAGACTAGTGATTTTGTTGTT	up	XLOC_001289	down
ssa-miR-1-4-5p	ACATACTTCTTTATATGCCCATA	up	XLOC_001307	up
ola-miR-462_L-1R+4	TAACGGAACCCATAATGCAGCT	down	XLOC_001321	up
ssa-miR-1338-5p_R+1	AGGACTGTCCAACCTGAGAATG	down	XLOC_001348	down
PC-5p-27517_164	TACATGCAGAGGTGGAGCAAGA	up	XLOC_001360	down
PC-3p-41259_77	TGGCCATTAACTGCTAACCTTC	up	XLOC_001513	down
ssa-miR-206-3p	TGGAATGTAAGGAAGTGTGTGG	up	XLOC_001513	down
ssc-miR-206	TGGAATGTAAGGAAGTGTGTGA	up	XLOC_001513	down
ola-miR-199a-3p_L+1	AACAGTAGTCTGCACATTGGTTA	up	XLOC_001548	up
ssa-miR-16b-5p_R-1_1ss21TC	TAGCAGCACGTAAATATTGGC	down	XLOC_001548	up
ssa-miR-199a-3p_R+2	ACAGTAGTCTGCACATTGGTTTT	up	XLOC_001548	up
xtr-miR-122_L+1R-1	CTGGAGTGTGACAATGGTGTTTG	up	XLOC_001548	up
ola-miR-199a-3p_L+1	AACAGTAGTCTGCACATTGGTTA	up	XLOC_001641	up
ola-mir-100-2-p3	CAAGCTCGTATCTATAGGTATG	down	XLOC_001641	up
dre-miR-194a_R+2	TGTAACAGCAACTCCATGTGGAT	up	XLOC_001645	up

tni-miR-194_R+1	TGTAACAGCAACTCCATGTGGA	up	XLOC_001645	up
ssa-miR-730a-5p_R-1	TCCTCATTGTGCATGCTGTGT	down	XLOC_001656	down
PC-3p-50929_43	TGGAAGTGTCAGAAATTCTGAGT	up	XLOC_001665	up
ssa-miR-7132b-3p	TGAGGCGTTTAGAACAAGTTCA	down	XLOC_001665	up
dre-miR-21_1ss23CA	TAGCTTATCAGACTGGTGTTGGA	up	XLOC_001707	up
aca-miR-338-3p_R+2	TCCAGCATCAGTGATTTTGTTAA	up	XLOC_001755	up
dre-miR-125b-5p_R+1	TCCCTGAGACCCTAACTTGTGAT	up	XLOC_001755	up
ola-miR-194-3p_1ss20CT	CCAGTGGAGGTGCTGTTACTTG	up	XLOC_001755	up
sha-miR-125a_R+2	TCCCTGAGACCCTAACTTGTGAAA	up	XLOC_001755	up
ssa-miR-125b-5p_R-1	TCCCTGAGACCCTTAACCTGTG	up	XLOC_001755	up
ssa-miR-26d-5p_L+1_1ss13TC	CTTCAAGTAATCCAGGATAGGCT	up	XLOC_001755	up
PC-5p-27517_164	TACATGCAGAGGTGGAGCAAGA	up	XLOC_001761	up
aca-miR-338-3p_R+2	TCCAGCATCAGTGATTTTGTTAA	up	XLOC_001761	up
dre-miR-140-3p_L-1	ACCACAGGGTAGAACCACGGAC	up	XLOC_001761	up
ssa-miR-7a-5p	TGGAAGACTAGTGATTTTGTTGT	up	XLOC_001761	up
ssa-miR-7a-5p_R+1	TGGAAGACTAGTGATTTTGTTGTT	up	XLOC_001761	up
dre-miR-140-3p_L-1	ACCACAGGGTAGAACCACGGAC	up	XLOC_001771	up
PC-3p-11630_419	ATGAGGAAAAGAAGTTAGGAGA	down	XLOC_001821	down
ssa-miR-1338-5p_R+1	AGGACTGTCCAACCTGAGAATG	down	XLOC_001821	down
PC-3p-50929_43	TGGAAGTGTCAGAAATTCTGAGT	up	XLOC_001886	down
aca-miR-200b-3p_R+2	TAATACTGCCTGGTAATGATGAAT	up	XLOC_001886	down
dre-miR-142a-5p	CATAAAGTAGAAAGCACTACT	down	XLOC_001886	down
aca-miR-200b-3p_R+2	TAATACTGCCTGGTAATGATGAAT	up	XLOC_001887	up
dre-miR-142a-5p	CATAAAGTAGAAAGCACTACT	down	XLOC_001887	up
dre-miR-194a_R+2	TGTAACAGCAACTCCATGTGGAT	up	XLOC_001887	up
ssa-miR-199a-3p_R+2	ACAGTAGTCTGCACATTGGTTTT	up	XLOC_001887	up
ssa-miR-730a-5p_R-1	TCCTCATTGTGCATGCTGTGT	down	XLOC_001887	up
tni-miR-194_R+1	TGTAACAGCAACTCCATGTGGA	up	XLOC_001887	up
dre-miR-142a-3p_R-1	TGTAGTGTTTCCTACTTTATGG	down	XLOC_001890	up
ola-miR-199a-3p_L+1	AACAGTAGTCTGCACATTGGTTA	up	XLOC_001890	up
PC-5p-8690_526	GATGTTGAGTATCAAACTGTAT	down	XLOC_001909	down
dre-miR-142a-5p	CATAAAGTAGAAAGCACTACT	down	XLOC_001909	down
ola-miR-194-3p_1ss20CT	CCAGTGGAGGTGCTGTTACTTG	up	XLOC_001909	down
ssa-miR-7a-5p	TGGAAGACTAGTGATTTTGTTGT	up	XLOC_001909	down
ssa-miR-7a-5p_R+1	TGGAAGACTAGTGATTTTGTTGTT	up	XLOC_001909	down
dre-miR-122	TGGAGTGTGACAATGGTGTTTG	up	XLOC_001916	up
dre-miR-133b-3p_R-1	TTTGGTCCCCTTCAACCAGCT	up	XLOC_001916	up
dre-miR-194a_R+2	TGTAACAGCAACTCCATGTGGAT	up	XLOC_001916	up
oha-miR-133b-3p	TTTGGTCCCCTTCAACCAGCTAT	up	XLOC_001916	up
ssa-miR-16b-5p_R-1_1ss21TC	TAGCAGCACGTAAATATTGGC	down	XLOC_001916	up
tni-miR-194_R+1	TGTAACAGCAACTCCATGTGGA	up	XLOC_001916	up
dre-miR-1	TGGAATGTAAAGAAGTATGTAT	up	XLOC_001927	up
ssa-miR-1338-5p_R+1	AGGACTGTCCAACCTGAGAATG	down	XLOC_001927	up
ssa-miR-206-3p	TGGAATGTAAGGAAGTGTGTGG	up	XLOC_001927	up
ssc-miR-206	TGGAATGTAAGGAAGTGTGTGA	up	XLOC_001927	up
PC-5p-27517_164	TACATGCAGAGGTGGAGCAAGA	up	XLOC_001958	down
dre-miR-1	TGGAATGTAAAGAAGTATGTAT	up	XLOC_001958	down
ola-miR-462_L-1R+4	TAACGGAACCCATAATGCAGCT	down	XLOC_001958	down
tni-miR-10c	TACCCTGTAGATCCGGATTTGT	up	XLOC_001958	down
PC-3p-11630_419	ATGAGGAAAAGAAGTTAGGAGA	down	XLOC_002020	down
PC-5p-8690_526	GATGTTGAGTATCAAACTGTAT	down	XLOC_002020	down
dre-miR-133a-3p_L-1R+1	TTGGTCCCCTTCAACCAGCTGT	up	XLOC_002020	down
PC-5p-45063_62	AAGGATAACTACAACTGTACTT	up	XLOC_002035	up
aca-miR-338-3p_R+2	TCCAGCATCAGTGATTTTGTTAA	up	XLOC_002035	up

dre-miR-1	TGGAATGTAAAGAAGTATGTAT	up	XLOC_002035	up
dre-miR-133a-3p_L-1R+1	TTGGTCCCCTTCAACCAGCTGT	up	XLOC_002035	up
dre-miR-133b-3p_R-1	TTTGGTCCCCTTCAACCAGCT	up	XLOC_002035	up
dre-miR-24_R+2_1	TGGCTCAGTTCAGCAGGAACAGAA	up	XLOC_002035	up
dre-miR-24_R+2_2	TGGCTCAGTTCAGCAGGAACAGTT	up	XLOC_002035	up
oha-miR-133b-3p	TTTGGTCCCCTTCAACCAGCTAT	up	XLOC_002035	up
ola-miR-194-3p_1ss20CT	CCAGTGGAGGTGCTGTTACTTG	up	XLOC_002035	up
ssa-miR-1338-5p_R+1	AGGACTGTCCAACCTGAGAATG	down	XLOC_002035	up
ssa-miR-206-3p	TGGAATGTAAGGAAGTGTGTGG	up	XLOC_002035	up
ssa-miR-730a-5p_R-1	TCCTCATTGTGCATGCTGTGT	down	XLOC_002035	up
ssc-miR-206	TGGAATGTAAGGAAGTGTGTGA	up	XLOC_002035	up
PC-3p-11630_419	ATGAGGAAAAGAAGTTAGGAGA	down	XLOC_002037	down
ola-miR-194-3p_1ss20CT	CCAGTGGAGGTGCTGTTACTTG	up	XLOC_002037	down
dre-miR-24_R+2_1	TGGCTCAGTTCAGCAGGAACAGAA	up	XLOC_002061	down
dre-miR-24_R+2_2	TGGCTCAGTTCAGCAGGAACAGTT	up	XLOC_002061	down
ola-miR-199a-3p_L+1	AACAGTAGTCTGCACATTGGTTA	up	XLOC_002061	down
ssa-miR-16b-5p_R-1_1ss21TC	TAGCAGCACGTAAATATTGGC	down	XLOC_002061	down
ssa-miR-199a-3p_R+2	ACAGTAGTCTGCACATTGGTTTT	up	XLOC_002061	down
ssa-miR-7a-5p	TGGAAGACTAGTGATTTTGTTGT	up	XLOC_002061	down
ssa-miR-7a-5p_R+1	TGGAAGACTAGTGATTTTGTTGTT	up	XLOC_002061	down
PC-5p-8690_526	GATGTTGAGTATCAAACTGTAT	down	XLOC_002223	down
aca-miR-338-3p_R+2	TCCAGCATCAGTGATTTTGTTAA	up	XLOC_002223	down
dre-miR-194a_R+2	TGTAACAGCAACTCCATGTGGAT	up	XLOC_002223	down
dre-miR-24_R+2_1	TGGCTCAGTTCAGCAGGAACAGAA	up	XLOC_002223	down
dre-miR-24_R+2_2	TGGCTCAGTTCAGCAGGAACAGTT	up	XLOC_002223	down
tni-miR-194_R+1	TGTAACAGCAACTCCATGTGGA	up	XLOC_002223	down
dre-miR-140-3p_L-1	ACCACAGGGTAGAACCACGGAC	up	XLOC_002231	up
ola-miR-146a-5p_1ss24TA	TGAGAACTGAATTCCATAGATGGAA	up	XLOC_002231	up
rno-miR-122-5p_L+3	ATCTGGAGTGTGACAATGGTGTTTG	up	XLOC_002231	up
ssa-miR-1-4-5p	ACATACTTCTTTATATGCCCATA	up	XLOC_002231	up
ssa-miR-1338-5p_R+1	AGGACTGTCCAACCTGAGAATG	down	XLOC_002231	up
PC-5p-27517_164	TACATGCAGAGGTGGAGCAAGA	up	XLOC_002240	down
ola-miR-199a-3p_L+1	AACAGTAGTCTGCACATTGGTTA	up	XLOC_002240	down
dre-miR-22a-3p	AAGCTGCCAGCTGAAGAACTGT	up	XLOC_002244	down
ssa-miR-1338-5p_R+1	AGGACTGTCCAACCTGAGAATG	down	XLOC_002244	down
PC-3p-11630_419	ATGAGGAAAAGAAGTTAGGAGA	down	XLOC_002262	up
aca-miR-338-3p_R+2	TCCAGCATCAGTGATTTTGTTAA	up	XLOC_002262	up
dre-miR-140-3p_L-1	ACCACAGGGTAGAACCACGGAC	up	XLOC_002262	up
PC-3p-50929_43	TGGAAGTGTCAGAAATTCTGAGT	up	XLOC_002276	up
PC-5p-27517_164	TACATGCAGAGGTGGAGCAAGA	up	XLOC_002276	up
dre-miR-125b-5p_R+1	TCCCTGAGACCCTAACTTGTGAT	up	XLOC_002276	up
ola-miR-199a-3p_L+1	AACAGTAGTCTGCACATTGGTTA	up	XLOC_002276	up
sha-miR-125a_R+2	TCCCTGAGACCCTAACTTGTGAAA	up	XLOC_002276	up
ssa-miR-125b-5p_R-1	TCCCTGAGACCCTTAACCTGTG	up	XLOC_002276	up
PC-3p-11630_419	ATGAGGAAAAGAAGTTAGGAGA	down	XLOC_002283	up
aca-miR-338-3p_R+2	TCCAGCATCAGTGATTTTGTTAA	up	XLOC_002283	up
dre-let-7d-5p	TGAGGTAGTTGGTTGTATGGTT	up	XLOC_002283	up
mmu-let-7j_1ss8TG	TGAGGTAGTAGTTTGTGCTGTTAT	up	XLOC_002283	up
tni-let-7j_1ss11TG	TGAGGTAGTTGTTTGTACAGTT	up	XLOC_002283	up
xtr-miR-122_L+1R-1	CTGGAGTGTGACAATGGTGTTTG	up	XLOC_002283	up
dre-miR-24_R+2_1	TGGCTCAGTTCAGCAGGAACAGAA	up	XLOC_002291	down
dre-miR-24_R+2_2	TGGCTCAGTTCAGCAGGAACAGTT	up	XLOC_002291	down
ola-miR-194-3p_1ss20CT	CCAGTGGAGGTGCTGTTACTTG	up	XLOC_002291	down
ssa-miR-7a-5p	TGGAAGACTAGTGATTTTGTTGT	up	XLOC_002291	down

ssa-miR-7a-5p_R+1	TGGAAGACTAGTGATTTTGTTGTT	up	XLOC_002291	down
PC-3p-11630_419	ATGAGGAAAAGAAGTTAGGAGA	down	XLOC_002328	up
PC-3p-50929_43	TGGAAGTGTCAGAAATTCTGAGT	up	XLOC_002328	up
PC-5p-27517_164	TACATGCAGAGGTGGAGCAAGA	up	XLOC_002328	up
aca-miR-200b-3p_R+2	TAATACTGCCTGGTAATGATGAAT	up	XLOC_002328	up
aca-miR-338-3p_R+2	TCCAGCATCAGTGATTTTGTTAA	up	XLOC_002328	up
dre-miR-125b-5p_R+1	TCCCTGAGACCCTAACTTGTGAT	up	XLOC_002328	up
dre-miR-142a-5p	CATAAAGTAGAAAGCACTACT	down	XLOC_002328	up
dre-miR-194a_R+2	TGTAACAGCAACTCCATGTGGAT	up	XLOC_002328	up
ola-miR-194-3p_1ss20CT	CCAGTGGAGGTGCTGTTACTTG	up	XLOC_002328	up
sha-miR-125a_R+2	TCCCTGAGACCCTAACTTGTGAAA	up	XLOC_002328	up
ssa-miR-125b-5p_R-1	TCCCTGAGACCCTTAACCTGTG	up	XLOC_002328	up
ssa-miR-199a-3p_R+2	ACAGTAGTCTGCACATTGGTTTT	up	XLOC_002328	up
tni-miR-194_R+1	TGTAACAGCAACTCCATGTGGA	up	XLOC_002328	up
PC-5p-45063_62	AAGGATAACTACAACTGTACTT	up	XLOC_002385	down
ola-miR-146a-5p_1ss24TA	TGAGAACTGAATTCCATAGATGGAA	up	XLOC_002405	up
tni-miR-10c	TACCCTGTAGATCCGGATTTGT	up	XLOC_002405	up
PC-3p-41259_77	TGGCCATTAACTGCTAACCTTC	up	XLOC_002434	up
PC-5p-8690_526	GATGTTGAGTATCAAACTGTAT	down	XLOC_002434	up
ssa-miR-16b-5p_R-1_1ss21TC	TAGCAGCACGTAAATATTGGC	down	XLOC_002434	up
ssa-miR-26a-4-3p	CCTATTCTTGATTACTTGTTTC	down	XLOC_002434	up
PC-5p-8690_526	GATGTTGAGTATCAAACTGTAT	down	XLOC_002478	down
aca-miR-338-3p_R+2	TCCAGCATCAGTGATTTTGTTAA	up	XLOC_002478	down
dre-miR-21_1ss23CA	TAGCTTATCAGACTGGTGTTGGA	up	XLOC_002478	down
dre-miR-22a-3p	AAGCTGCCAGCTGAAGAACTGT	up	XLOC_002478	down
ssa-miR-16b-5p_R-1_1ss21TC	TAGCAGCACGTAAATATTGGC	down	XLOC_002478	down
ssa-miR-199a-3p_R+2	ACAGTAGTCTGCACATTGGTTTT	up	XLOC_002478	down
ssa-miR-26d-5p_L+1_1ss13TC	CTTCAAGTAATCCAGGATAGGCT	up	XLOC_002478	down
PC-5p-27517_164	TACATGCAGAGGTGGAGCAAGA	up	XLOC_002479	down
dre-miR-122	TGGAGTGTGACAATGGTGTTTG	up	XLOC_002479	down
dre-miR-140-3p_L-1	ACCACAGGGTAGAACCACGGAC	up	XLOC_002479	down
ola-miR-194-3p_1ss20CT	CCAGTGGAGGTGCTGTTACTTG	up	XLOC_002479	down
ssa-miR-199a-3p_R+2	ACAGTAGTCTGCACATTGGTTTT	up	XLOC_002479	down
ssa-miR-730a-5p_R-1	TCCTCATTGTGCATGCTGTGT	down	XLOC_002479	down
ssa-miR-26d-5p_L+1_1ss13TC	CTTCAAGTAATCCAGGATAGGCT	up	XLOC_002507	up
ssa-miR-7a-5p	TGGAAGACTAGTGATTTTGTTGT	up	XLOC_002507	up
ssa-miR-7a-5p_R+1	TGGAAGACTAGTGATTTTGTTGTT	up	XLOC_002507	up
tni-miR-10c	TACCCTGTAGATCCGGATTTGT	up	XLOC_002507	up
aca-miR-338-3p_R+2	TCCAGCATCAGTGATTTTGTTAA	up	XLOC_002524	up
dre-miR-122	TGGAGTGTGACAATGGTGTTTG	up	XLOC_002524	up
dre-miR-194a_R+2	TGTAACAGCAACTCCATGTGGAT	up	XLOC_002524	up
rno-miR-122-5p_L+3	ATCTGGAGTGTGACAATGGTGTTTG	up	XLOC_002524	up
ssa-miR-16b-5p_R-1_1ss21TC	TAGCAGCACGTAAATATTGGC	down	XLOC_002524	up
ssa-miR-730a-5p_R-1	TCCTCATTGTGCATGCTGTGT	down	XLOC_002524	up
tni-miR-194_R+1	TGTAACAGCAACTCCATGTGGA	up	XLOC_002524	up
xtr-miR-122_L+1R-1	CTGGAGTGTGACAATGGTGTTTG	up	XLOC_002524	up
aca-miR-200b-3p_R+2	TAATACTGCCTGGTAATGATGAAT	up	XLOC_002558	up
dre-miR-122	TGGAGTGTGACAATGGTGTTTG	up	XLOC_002558	up
dre-miR-21_1ss23CA	TAGCTTATCAGACTGGTGTTGGA	up	XLOC_002558	up
ola-miR-146a-5p_1ss24TA	TGAGAACTGAATTCCATAGATGGAA	up	XLOC_002558	up
rno-miR-122-5p_L+3	ATCTGGAGTGTGACAATGGTGTTTG	up	XLOC_002558	up
ssa-miR-16b-5p_R-1_1ss21TC	TAGCAGCACGTAAATATTGGC	down	XLOC_002558	up
ssa-miR-730a-5p_R-1	TCCTCATTGTGCATGCTGTGT	down	XLOC_002558	up
xtr-miR-122_L+1R-1	CTGGAGTGTGACAATGGTGTTTG	up	XLOC_002558	up

PC-5p-27517_164	TACATGCAGAGGTGGAGCAAGA	up	XLOC_002631	up
dre-miR-22a-3p	AAGCTGCCAGCTGAAGAACTGT	up	XLOC_002631	up
ssa-miR-26d-5p_L+1_1ss13TC	CTTCAAGTAATCCAGGATAGGCT	up	XLOC_002631	up
dre-miR-125b-5p_R+1	TCCCTGAGACCCTAACTTGTGAT	up	XLOC_002645	down
dre-miR-21_1ss23CA	TAGCTTATCAGACTGGTGTTGGA	up	XLOC_002645	down
sha-miR-125a_R+2	TCCCTGAGACCCTAACTTGTGAAA	up	XLOC_002645	down
ssa-miR-125b-5p_R-1	TCCCTGAGACCCTTAACCTGTG	up	XLOC_002645	down
ssa-miR-16b-5p_R-1_1ss21TC	TAGCAGCACGTAAATATTGGC	down	XLOC_002645	down
ssa-miR-730a-5p_R-1	TCCTCATTGTGCATGCTGTGT	down	XLOC_002645	down
tni-miR-10c	TACCCTGTAGATCCGGATTTGT	up	XLOC_002645	down
PC-3p-50929_43	TGGAAGTGTCAGAAATTCTGAGT	up	XLOC_002651	up
ssa-miR-16b-5p_R-1_1ss21TC	TAGCAGCACGTAAATATTGGC	down	XLOC_002651	up
PC-3p-11630_419	ATGAGGAAAAGAAGTTAGGAGA	down	XLOC_002702	up
dre-miR-22a-3p	AAGCTGCCAGCTGAAGAACTGT	up	XLOC_002702	up
ola-miR-194-3p_1ss20CT	CCAGTGGAGGTGCTGTTACTTG	up	XLOC_002702	up
PC-5p-27517_164	TACATGCAGAGGTGGAGCAAGA	up	XLOC_002781	down
dre-miR-122	TGGAGTGTGACAATGGTGTTTG	up	XLOC_002781	down
xtr-miR-122_L+1R-1	CTGGAGTGTGACAATGGTGTTTG	up	XLOC_002781	down
PC-3p-11630_419	ATGAGGAAAAGAAGTTAGGAGA	down	XLOC_002864	up
dre-let-7d-5p	TGAGGTAGTTGGTTGTATGGTT	up	XLOC_002867	up
dre-miR-133b-3p_R-1	TTTGGTCCCCTTCAACCAGCT	up	XLOC_002867	up
mmu-let-7j_1ss8TG	TGAGGTAGTAGTTTGTGCTGTTAT	up	XLOC_002867	up
oha-miR-133b-3p	TTTGGTCCCCTTCAACCAGCTAT	up	XLOC_002867	up
tni-let-7j_1ss11TG	TGAGGTAGTTGTTTGTACAGTT	up	XLOC_002867	up
ssa-miR-1338-5p_R+1	AGGACTGTCCAACCTGAGAATG	down	XLOC_002963	down
ssa-miR-16b-5p_R-1_1ss21TC	TAGCAGCACGTAAATATTGGC	down	XLOC_002963	down
dre-miR-133a-3p_L-1R+1	TTGGTCCCCTTCAACCAGCTGT	up	XLOC_002968	up
dre-miR-125b-5p_R+1	TCCCTGAGACCCTAACTTGTGAT	up	XLOC_002981	up
sha-miR-125a_R+2	TCCCTGAGACCCTAACTTGTGAAA	up	XLOC_002981	up
ssa-miR-125b-5p_R-1	TCCCTGAGACCCTTAACCTGTG	up	XLOC_002981	up
ssa-miR-1338-5p_R+1	AGGACTGTCCAACCTGAGAATG	down	XLOC_003020	down
PC-3p-11630_419	ATGAGGAAAAGAAGTTAGGAGA	down	XLOC_003081	down
ssa-miR-1-4-5p	ACATACTTCTTTATATGCCCATA	up	XLOC_003081	down
ola-miR-194-3p_1ss20CT	CCAGTGGAGGTGCTGTTACTTG	up	XLOC_003093	down
ola-miR-199a-3p_L+1	AACAGTAGTCTGCACATTGGTTA	up	XLOC_003093	down
ssa-miR-199a-3p_R+2	ACAGTAGTCTGCACATTGGTTTT	up	XLOC_003093	down
dre-miR-194a_R+2	TGTAACAGCAACTCCATGTGGAT	up	XLOC_003123	up
ssa-miR-26d-5p_L+1_1ss13TC	CTTCAAGTAATCCAGGATAGGCT	up	XLOC_003123	up
tni-miR-194_R+1	TGTAACAGCAACTCCATGTGGA	up	XLOC_003123	up
dre-miR-24_R+2_1	TGGCTCAGTTCAGCAGGAACAGAA	up	XLOC_003178	down
dre-miR-24_R+2_2	TGGCTCAGTTCAGCAGGAACAGTT	up	XLOC_003178	down
ssa-miR-16b-5p_R-1_1ss21TC	TAGCAGCACGTAAATATTGGC	down	XLOC_003178	down
PC-5p-27517_164	TACATGCAGAGGTGGAGCAAGA	up	XLOC_003194	up
dre-miR-194a_R+2	TGTAACAGCAACTCCATGTGGAT	up	XLOC_003195	up
ola-miR-199a-3p_L+1	AACAGTAGTCTGCACATTGGTTA	up	XLOC_003195	up
tni-miR-194_R+1	TGTAACAGCAACTCCATGTGGA	up	XLOC_003195	up
xtr-miR-122_L+1R-1	CTGGAGTGTGACAATGGTGTTTG	up	XLOC_003195	up
dre-miR-21_1ss23CA	TAGCTTATCAGACTGGTGTTGGA	up	XLOC_003202	up
tni-miR-10c	TACCCTGTAGATCCGGATTTGT	up	XLOC_003202	up
PC-3p-11630_419	ATGAGGAAAAGAAGTTAGGAGA	down	XLOC_003220	down
dre-miR-142a-5p	CATAAAGTAGAAAGCACTACT	down	XLOC_003220	down
ola-miR-194-3p_1ss20CT	CCAGTGGAGGTGCTGTTACTTG	up	XLOC_003220	down
ssa-miR-730a-5p_R-1	TCCTCATTGTGCATGCTGTGT	down	XLOC_003220	down
ssa-miR-7a-5p	TGGAAGACTAGTGATTTTGTTGT	up	XLOC_003220	down

ssa-miR-7a-5p_R+1	TGGAAGACTAGTGATTTTGTTGTT	up	XLOC_003220	down
ola-miR-146a-5p_1ss24TA	TGAGAACTGAATTCCATAGATGGAA	up	XLOC_003247	up
rno-miR-122-5p_L+3	ATCTGGAGTGTGACAATGGTGTTTG	up	XLOC_003247	up
PC-3p-41259_77	TGGCCATTAACTGCTAACCTTC	up	XLOC_003263	up
PC-5p-45063_62	AAGGATAACTACAACTGTACTT	up	XLOC_003263	up
tni-miR-10c	TACCCTGTAGATCCGGATTTGT	up	XLOC_003263	up
PC-5p-8690_526	GATGTTGAGTATCAAACTGTAT	down	XLOC_003290	down
ola-miR-194-3p_1ss20CT	CCAGTGGAGGTGCTGTTACTTG	up	XLOC_003290	down
ssa-miR-7132b-3p	TGAGGCGTTTAGAACAAGTTCA	down	XLOC_003290	down
dre-miR-142a-5p	CATAAAGTAGAAAGCACTACT	down	XLOC_003309	down
ssa-miR-16b-5p_R-1_1ss21TC	TAGCAGCACGTAAATATTGGC	down	XLOC_003309	down
ssa-miR-730a-5p_R-1	TCCTCATTGTGCATGCTGTGT	down	XLOC_003312	down
dre-miR-194a_R+2	TGTAACAGCAACTCCATGTGGAT	up	XLOC_003331	down
ola-miR-199a-3p_L+1	AACAGTAGTCTGCACATTGGTTA	up	XLOC_003331	down
tni-miR-194_R+1	TGTAACAGCAACTCCATGTGGA	up	XLOC_003331	down
dre-miR-140-3p_L-1	ACCACAGGGTAGAACCACGGAC	up	XLOC_003375	down
ssa-miR-16b-5p_R-1_1ss21TC	TAGCAGCACGTAAATATTGGC	down	XLOC_003405	up
aca-miR-338-3p_R+2	TCCAGCATCAGTGATTTTGTTAA	up	XLOC_003465	down
dre-miR-21_1ss23CA	TAGCTTATCAGACTGGTGTTGGA	up	XLOC_003502	up
PC-3p-50929_43	TGGAAGTGTCAGAAATTCTGAGT	up	XLOC_003513	up
aca-miR-200b-3p_R+2	TAATACTGCCTGGTAATGATGAAT	up	XLOC_003551	up
dre-miR-1	TGGAATGTAAAGAAGTATGTAT	up	XLOC_003551	up
ola-miR-194-3p_1ss20CT	CCAGTGGAGGTGCTGTTACTTG	up	XLOC_003551	up
ssa-miR-16b-5p_R-1_1ss21TC	TAGCAGCACGTAAATATTGGC	down	XLOC_003551	up
dre-miR-1	TGGAATGTAAAGAAGTATGTAT	up	XLOC_003559	down
ola-miR-462_L-1R+4	TAACGGAACCCATAATGCAGCT	down	XLOC_003559	down
ssa-miR-206-3p	TGGAATGTAAGGAAGTGTGTGG	up	XLOC_003559	down
ssc-miR-206	TGGAATGTAAGGAAGTGTGTGA	up	XLOC_003559	down
ssa-miR-26d-5p_L+1_1ss13TC	CTTCAAGTAATCCAGGATAGGCT	up	XLOC_003632	up
ssa-miR-730a-5p_R-1	TCCTCATTGTGCATGCTGTGT	down	XLOC_003632	up
rno-miR-122-5p_L+3	ATCTGGAGTGTGACAATGGTGTTTG	up	XLOC_003659	up
xtr-miR-122_L+1R-1	CTGGAGTGTGACAATGGTGTTTG	up	XLOC_003659	up
PC-5p-27517_164	TACATGCAGAGGTGGAGCAAGA	up	XLOC_003700	up
ssa-miR-1-4-5p	ACATACTTCTTTATATGCCCATA	up	XLOC_003700	up
ssa-miR-125b-5p_R-1	TCCCTGAGACCCTTAACCTGTG	up	XLOC_003700	up
PC-3p-41259_77	TGGCCATTAACTGCTAACCTTC	up	XLOC_003736	down
PC-5p-27517_164	TACATGCAGAGGTGGAGCAAGA	up	XLOC_003736	down
PC-5p-8690_526	GATGTTGAGTATCAAACTGTAT	down	XLOC_003736	down
PC-3p-11630_419	ATGAGGAAAAGAAGTTAGGAGA	down	XLOC_003795	up
ola-miR-199a-3p_L+1	AACAGTAGTCTGCACATTGGTTA	up	XLOC_003795	up
aca-miR-338-3p_R+2	TCCAGCATCAGTGATTTTGTTAA	up	XLOC_003806	down
ssa-miR-7132a-5p_R+1	GACTTGGTCAAAGCTCCTCAGTT	down	XLOC_003806	down
ssa-miR-7132b-5p	GACTTGGTCAAAGCTCCTCAGC	down	XLOC_003806	down
ssa-mir-15c-2-p3_1ss11CA	TGCGAACCATAATTTGCTGCTT	down	XLOC_003806	down
ola-mir-100-2-p3	CAAGCTCGTATCTATAGGTATG	down	XLOC_003808	down
ssa-miR-16b-5p_R-1_1ss21TC	TAGCAGCACGTAAATATTGGC	down	XLOC_003808	down
ssa-miR-26d-5p_L+1_1ss13TC	CTTCAAGTAATCCAGGATAGGCT	up	XLOC_003814	down
dre-miR-194a_R+2	TGTAACAGCAACTCCATGTGGAT	up	XLOC_003820	down
ssa-miR-16b-5p_R-1_1ss21TC	TAGCAGCACGTAAATATTGGC	down	XLOC_003820	down
tni-miR-194_R+1	TGTAACAGCAACTCCATGTGGA	up	XLOC_003820	down
ssa-miR-206-3p	TGGAATGTAAGGAAGTGTGTGG	up	XLOC_003822	down
ssa-miR-7132a-5p_R+1	GACTTGGTCAAAGCTCCTCAGTT	down	XLOC_003822	down
ssa-miR-7132b-5p	GACTTGGTCAAAGCTCCTCAGC	down	XLOC_003822	down
ssc-miR-206	TGGAATGTAAGGAAGTGTGTGA	up	XLOC_003822	down

aca-miR-200b-3p_R+2	TAATACTGCCTGGTAATGATGAAT	up	XLOC_003830	down
dre-miR-24_R+2_1	TGGCTCAGTTCAGCAGGAACAGAA	up	XLOC_003830	down
dre-miR-24_R+2_2	TGGCTCAGTTCAGCAGGAACAGTT	up	XLOC_003830	down
aca-miR-200b-3p_R+2	TAATACTGCCTGGTAATGATGAAT	up	XLOC_003836	down
dre-miR-125b-5p_R+1	TCCCTGAGACCCTAACTTGTGAT	up	XLOC_003836	down
dre-miR-194a_R+2	TGTAACAGCAACTCCATGTGGAT	up	XLOC_003836	down
sha-miR-125a_R+2	TCCCTGAGACCCTAACTTGTGAAA	up	XLOC_003836	down
ssa-miR-1-4-5p	ACATACTTCTTTATATGCCCATA	up	XLOC_003836	down
ssa-miR-125b-5p_R-1	TCCCTGAGACCCTTAACCTGTG	up	XLOC_003836	down
tni-miR-194_R+1	TGTAACAGCAACTCCATGTGGA	up	XLOC_003836	down
ola-miR-194-3p_1ss20CT	CCAGTGGAGGTGCTGTTACTTG	up	XLOC_003928	down
rno-miR-122-5p_L+3	ATCTGGAGTGTGACAATGGTGTTTG	up	XLOC_003928	down
ola-miR-194-3p_1ss20CT	CCAGTGGAGGTGCTGTTACTTG	up	XLOC_003987	down
PC-5p-8690_526	GATGTTGAGTATCAAACTGTAT	down	XLOC_004013	down
tni-miR-10c	TACCCTGTAGATCCGGATTTGT	up	XLOC_004013	down
aca-miR-200b-3p_R+2	TAATACTGCCTGGTAATGATGAAT	up	XLOC_004080	up
dre-miR-194a_R+2	TGTAACAGCAACTCCATGTGGAT	up	XLOC_004080	up
tni-miR-194_R+1	TGTAACAGCAACTCCATGTGGA	up	XLOC_004080	up
ola-miR-146a-5p_1ss24TA	TGAGAACTGAATTCCATAGATGGAA	up	XLOC_004112	up
aca-miR-338-3p_R+2	TCCAGCATCAGTGATTTTGTTAA	up	XLOC_004184	up
dre-miR-22a-3p	AAGCTGCCAGCTGAAGAACTGT	up	XLOC_004184	up
ssa-miR-16b-5p_R-1_1ss21TC	TAGCAGCACGTAAATATTGGC	down	XLOC_004184	up
ola-miR-146a-5p_1ss24TA	TGAGAACTGAATTCCATAGATGGAA	up	XLOC_004207	up
ola-miR-194-3p_1ss20CT	CCAGTGGAGGTGCTGTTACTTG	up	XLOC_004245	down
ssa-miR-730a-5p_R-1	TCCTCATTGTGCATGCTGTGT	down	XLOC_004245	down
aca-miR-338-3p_R+2	TCCAGCATCAGTGATTTTGTTAA	up	XLOC_004269	down
dre-miR-21_1ss23CA	TAGCTTATCAGACTGGTGTTGGA	up	XLOC_004269	down
ssa-miR-26d-5p_L+1_1ss13TC	CTTCAAGTAATCCAGGATAGGCT	up	XLOC_004269	down
mmu-let-7j_1ss8TG	TGAGGTAGTAGTTTGTGCTGTTAT	up	XLOC_004317	up
ola-miR-146a-5p_1ss24TA	TGAGAACTGAATTCCATAGATGGAA	up	XLOC_004317	up
ssa-miR-16b-5p_R-1_1ss21TC	TAGCAGCACGTAAATATTGGC	down	XLOC_004317	up
ssa-miR-206-3p	TGGAATGTAAGGAAGTGTGTGG	up	XLOC_004317	up
ssc-miR-206	TGGAATGTAAGGAAGTGTGTGA	up	XLOC_004317	up
PC-3p-41259_77	TGGCCATTAACTGCTAACCTTC	up	XLOC_004335	up
PC-3p-50929_43	TGGAAGTGTCAGAAATTCTGAGT	up	XLOC_004335	up
dre-miR-1	TGGAATGTAAAGAAGTATGTAT	up	XLOC_004335	up
dre-miR-140-3p_L-1	ACCACAGGGTAGAACCACGGAC	up	XLOC_004335	up
dre-miR-142a-3p_R-1	TGTAGTGTTTCCTACTTTATGG	down	XLOC_004335	up
ola-miR-194-3p_1ss20CT	CCAGTGGAGGTGCTGTTACTTG	up	XLOC_004335	up
ssa-miR-206-3p	TGGAATGTAAGGAAGTGTGTGG	up	XLOC_004335	up
ssa-miR-730a-5p_R-1	TCCTCATTGTGCATGCTGTGT	down	XLOC_004335	up
ssc-miR-206	TGGAATGTAAGGAAGTGTGTGA	up	XLOC_004335	up
PC-5p-8690_526	GATGTTGAGTATCAAACTGTAT	down	XLOC_004363	up
dre-miR-24_R+2_1	TGGCTCAGTTCAGCAGGAACAGAA	up	XLOC_004363	up
dre-miR-24_R+2_2	TGGCTCAGTTCAGCAGGAACAGTT	up	XLOC_004363	up
ssa-miR-7a-5p	TGGAAGACTAGTGATTTTGTTGT	up	XLOC_004363	up
ssa-miR-7a-5p_R+1	TGGAAGACTAGTGATTTTGTTGTT	up	XLOC_004363	up
PC-3p-50929_43	TGGAAGTGTCAGAAATTCTGAGT	up	XLOC_004367	up
dre-let-7d-5p	TGAGGTAGTTGGTTGTATGGTT	up	XLOC_004367	up
mmu-let-7j_1ss8TG	TGAGGTAGTAGTTTGTGCTGTTAT	up	XLOC_004367	up
ola-miR-194-3p_1ss20CT	CCAGTGGAGGTGCTGTTACTTG	up	XLOC_004367	up
tni-let-7j_1ss11TG	TGAGGTAGTTGTTTGTACAGTT	up	XLOC_004367	up
aca-miR-200b-3p_R+2	TAATACTGCCTGGTAATGATGAAT	up	XLOC_004402	up
dre-miR-194a_R+2	TGTAACAGCAACTCCATGTGGAT	up	XLOC_004402	up

tni-miR-194_R+1	TGTAACAGCAACTCCATGTGGA	up	XLOC_004402	up
ola-miR-194-3p_1ss20CT	CCAGTGGAGGTGCTGTTACTTG	up	XLOC_004448	up
ssa-miR-16b-5p_R-1_1ss21TC	TAGCAGCACGTAAATATTGGC	down	XLOC_004448	up
PC-5p-27517_164	TACATGCAGAGGTGGAGCAAGA	up	XLOC_004449	up
dre-miR-1	TGGAATGTAAAGAAGTATGTAT	up	XLOC_004497	up
dre-miR-22a-3p	AAGCTGCCAGCTGAAGAACTGT	up	XLOC_004497	up
ola-miR-462_L-1R+4	TAACGGAACCCATAATGCAGCT	down	XLOC_004497	up
ssa-miR-206-3p	TGGAATGTAAGGAAGTGTGTGG	up	XLOC_004497	up
ssc-miR-206	TGGAATGTAAGGAAGTGTGTGA	up	XLOC_004497	up
ola-miR-199a-3p_L+1	AACAGTAGTCTGCACATTGGTTA	up	XLOC_004507	down
ssa-miR-199a-3p_R+2	ACAGTAGTCTGCACATTGGTTTT	up	XLOC_004507	down
dre-miR-142a-5p	CATAAAGTAGAAAGCACTACT	down	XLOC_004512	down
dre-miR-194a_R+2	TGTAACAGCAACTCCATGTGGAT	up	XLOC_004512	down
tni-miR-10c	TACCCTGTAGATCCGGATTTGT	up	XLOC_004512	down
tni-miR-194_R+1	TGTAACAGCAACTCCATGTGGA	up	XLOC_004512	down
PC-3p-11630_419	ATGAGGAAAAGAAGTTAGGAGA	down	XLOC_004554	down
ssa-miR-1338-5p_R+1	AGGACTGTCCAACCTGAGAATG	down	XLOC_004554	down
dre-miR-140-3p_L-1	ACCACAGGGTAGAACCACGGAC	up	XLOC_004605	up
ola-miR-194-3p_1ss20CT	CCAGTGGAGGTGCTGTTACTTG	up	XLOC_004605	up
rno-miR-122-5p_L+3	ATCTGGAGTGTGACAATGGTGTTTG	up	XLOC_004605	up
ssa-miR-16b-5p_R-1_1ss21TC	TAGCAGCACGTAAATATTGGC	down	XLOC_004605	up
dre-miR-133a-3p_L-1R+1	TTGGTCCCCTTCAACCAGCTGT	up	XLOC_004628	down
ola-miR-194-3p_1ss20CT	CCAGTGGAGGTGCTGTTACTTG	up	XLOC_004628	down
dre-miR-122	TGGAGTGTGACAATGGTGTTTG	up	XLOC_004712	down
xtr-miR-122_L+1R-1	CTGGAGTGTGACAATGGTGTTTG	up	XLOC_004712	down
PC-3p-50929_43	TGGAAGTGTCAGAAATTCTGAGT	up	XLOC_004716	up
ola-miR-199a-3p_L+1	AACAGTAGTCTGCACATTGGTTA	up	XLOC_004716	up
xtr-miR-122_L+1R-1	CTGGAGTGTGACAATGGTGTTTG	up	XLOC_004716	up
ssa-miR-26d-5p_L+1_1ss13TC	CTTCAAGTAATCCAGGATAGGCT	up	XLOC_004721	up
PC-3p-41259_77	TGGCCATTAACTGCTAACCTTC	up	XLOC_004733	down
PC-5p-27517_164	TACATGCAGAGGTGGAGCAAGA	up	XLOC_004733	down
ssa-miR-7132a-5p_R+1	GACTTGGTCAAAGCTCCTCAGTT	down	XLOC_004733	down
ssa-miR-7132b-5p	GACTTGGTCAAAGCTCCTCAGC	down	XLOC_004733	down
ssa-miR-730a-5p_R-1	TCCTCATTGTGCATGCTGTGT	down	XLOC_004733	down
aca-miR-200b-3p_R+2	TAATACTGCCTGGTAATGATGAAT	up	XLOC_004745	down
ola-miR-199a-3p_L+1	AACAGTAGTCTGCACATTGGTTA	up	XLOC_004745	down
ssa-miR-199a-3p_R+2	ACAGTAGTCTGCACATTGGTTTT	up	XLOC_004745	down
ssa-miR-26a-4-3p	CCTATTCTTGATTACTTGTTTC	down	XLOC_004745	down
PC-3p-50929_43	TGGAAGTGTCAGAAATTCTGAGT	up	XLOC_004773	up
dre-miR-140-3p_L-1	ACCACAGGGTAGAACCACGGAC	up	XLOC_004773	up
PC-5p-27517_164	TACATGCAGAGGTGGAGCAAGA	up	XLOC_004775	down
ola-miR-146a-5p_1ss24TA	TGAGAACTGAATTCCATAGATGGAA	up	XLOC_004775	down
ola-miR-194-3p_1ss20CT	CCAGTGGAGGTGCTGTTACTTG	up	XLOC_004775	down
aca-miR-338-3p_R+2	TCCAGCATCAGTGATTTTGTTAA	up	XLOC_004836	down
dre-miR-194a_R+2	TGTAACAGCAACTCCATGTGGAT	up	XLOC_004836	down
rno-miR-122-5p_L+3	ATCTGGAGTGTGACAATGGTGTTTG	up	XLOC_004836	down
ssa-miR-7a-5p	TGGAAGACTAGTGATTTTGTTGT	up	XLOC_004836	down
ssa-miR-7a-5p_R+1	TGGAAGACTAGTGATTTTGTTGTT	up	XLOC_004836	down
tni-miR-194_R+1	TGTAACAGCAACTCCATGTGGA	up	XLOC_004836	down
aca-miR-338-3p_R+2	TCCAGCATCAGTGATTTTGTTAA	up	XLOC_004874	up
ssa-miR-1338-5p_R+1	AGGACTGTCCAACCTGAGAATG	down	XLOC_004874	up
ssa-miR-16b-5p_R-1_1ss21TC	TAGCAGCACGTAAATATTGGC	down	XLOC_004874	up
PC-5p-27517_164	TACATGCAGAGGTGGAGCAAGA	up	XLOC_004902	up
ssa-miR-16b-5p_R-1_1ss21TC	TAGCAGCACGTAAATATTGGC	down	XLOC_005014	up

PC-5p-8690_526	GATGTTGAGTATCAAACTGTAT	down	XLOC_005015	down
aca-miR-338-3p_R+2	TCCAGCATCAGTGATTTTGTTAA	up	XLOC_005015	down
ola-mir-100-2-p3	CAAGCTCGTATCTATAGGTATG	down	XLOC_005015	down
ssa-miR-199a-3p_R+2	ACAGTAGTCTGCACATTGGTTTT	up	XLOC_005015	down
ssa-miR-26d-5p_L+1_1ss13TC	CTTCAAGTAATCCAGGATAGGCT	up	XLOC_005015	down
dre-miR-140-3p_L-1	ACCACAGGGTAGAACCACGGAC	up	XLOC_005031	down
PC-5p-27517_164	TACATGCAGAGGTGGAGCAAGA	up	XLOC_005060	down
aca-miR-200b-3p_R+2	TAATACTGCCTGGTAATGATGAAT	up	XLOC_005060	down
ssa-miR-730a-5p_R-1	TCCTCATTGTGCATGCTGTGT	down	XLOC_005060	down
dre-miR-133b-3p_R-1	TTTGGTCCCCTTCAACCAGCT	up	XLOC_005065	up
oha-miR-133b-3p	TTTGGTCCCCTTCAACCAGCTAT	up	XLOC_005065	up
ssa-miR-16b-5p_R-1_1ss21TC	TAGCAGCACGTAAATATTGGC	down	XLOC_005065	up
PC-3p-50929_43	TGGAAGTGTCAGAAATTCTGAGT	up	XLOC_005075	up
dre-let-7d-5p	TGAGGTAGTTGGTTGTATGGTT	up	XLOC_005075	up
dre-miR-194a_R+2	TGTAACAGCAACTCCATGTGGAT	up	XLOC_005075	up
mmu-let-7j_1ss8TG	TGAGGTAGTAGTTTGTGCTGTTAT	up	XLOC_005075	up
ssa-miR-199a-3p_R+2	ACAGTAGTCTGCACATTGGTTTT	up	XLOC_005075	up
ssa-miR-730a-5p_R-1	TCCTCATTGTGCATGCTGTGT	down	XLOC_005075	up
tni-let-7j_1ss11TG	TGAGGTAGTTGTTTGTACAGTT	up	XLOC_005075	up
tni-miR-194_R+1	TGTAACAGCAACTCCATGTGGA	up	XLOC_005075	up
ssa-miR-199a-3p_R+2	ACAGTAGTCTGCACATTGGTTTT	up	XLOC_005125	up
PC-5p-8690_526	GATGTTGAGTATCAAACTGTAT	down	XLOC_005140	up
ssa-miR-730a-5p_R-1	TCCTCATTGTGCATGCTGTGT	down	XLOC_005140	up
ssa-miR-16b-5p_R-1_1ss21TC	TAGCAGCACGTAAATATTGGC	down	XLOC_005151	up
dre-miR-21_1ss23CA	TAGCTTATCAGACTGGTGTTGGA	up	XLOC_005174	up
ssa-miR-26d-5p_L+1_1ss13TC	CTTCAAGTAATCCAGGATAGGCT	up	XLOC_005174	up
PC-5p-8690_526	GATGTTGAGTATCAAACTGTAT	down	XLOC_005187	up
dre-miR-122	TGGAGTGTGACAATGGTGTTTG	up	XLOC_005187	up
dre-miR-140-3p_L-1	ACCACAGGGTAGAACCACGGAC	up	XLOC_005187	up
rno-miR-122-5p_L+3	ATCTGGAGTGTGACAATGGTGTTTG	up	XLOC_005187	up
tni-miR-10c	TACCCTGTAGATCCGGATTTGT	up	XLOC_005187	up
xtr-miR-122_L+1R-1	CTGGAGTGTGACAATGGTGTTTG	up	XLOC_005187	up
dre-miR-140-3p_L-1	ACCACAGGGTAGAACCACGGAC	up	XLOC_005284	down
PC-3p-11630_419	ATGAGGAAAAGAAGTTAGGAGA	down	XLOC_005330	up
PC-5p-27517_164	TACATGCAGAGGTGGAGCAAGA	up	XLOC_005330	up
dre-miR-1	TGGAATGTAAAGAAGTATGTAT	up	XLOC_005330	up
dre-miR-140-3p_L-1	ACCACAGGGTAGAACCACGGAC	up	XLOC_005330	up
dre-miR-24_R+2_1	TGGCTCAGTTCAGCAGGAACAGAA	up	XLOC_005330	up
dre-miR-24_R+2_2	TGGCTCAGTTCAGCAGGAACAGTT	up	XLOC_005330	up
ola-miR-194-3p_1ss20CT	CCAGTGGAGGTGCTGTTACTTG	up	XLOC_005330	up
ssa-miR-199a-3p_R+2	ACAGTAGTCTGCACATTGGTTTT	up	XLOC_005330	up
ssa-miR-206-3p	TGGAATGTAAGGAAGTGTGTGG	up	XLOC_005330	up
ssa-miR-26a-4-3p	CCTATTCTTGATTACTTGTTTC	down	XLOC_005330	up
ssa-miR-7132b-3p	TGAGGCGTTTAGAACAAGTTCA	down	XLOC_005330	up
ssa-miR-7a-5p	TGGAAGACTAGTGATTTTGTTGT	up	XLOC_005330	up
ssa-miR-7a-5p_R+1	TGGAAGACTAGTGATTTTGTTGTT	up	XLOC_005330	up
ssc-miR-206	TGGAATGTAAGGAAGTGTGTGA	up	XLOC_005330	up
ssa-miR-16b-5p_R-1_1ss21TC	TAGCAGCACGTAAATATTGGC	down	XLOC_005344	up
ssa-miR-730a-5p_R-1	TCCTCATTGTGCATGCTGTGT	down	XLOC_005344	up
dre-miR-140-3p_L-1	ACCACAGGGTAGAACCACGGAC	up	XLOC_005351	up
dre-miR-194a_R+2	TGTAACAGCAACTCCATGTGGAT	up	XLOC_005351	up
tni-miR-194_R+1	TGTAACAGCAACTCCATGTGGA	up	XLOC_005351	up
PC-5p-27517_164	TACATGCAGAGGTGGAGCAAGA	up	XLOC_005372	down
tni-miR-10c	TACCCTGTAGATCCGGATTTGT	up	XLOC_005372	down

dre-miR-140-3p_L-1	ACCACAGGGTAGAACCACGGAC	up	XLOC_005395	up
ssa-miR-16b-5p_R-1_1ss21TC	TAGCAGCACGTAAATATTGGC	down	XLOC_005432	up
PC-5p-27517_164	TACATGCAGAGGTGGAGCAAGA	up	XLOC_005472	up
ssa-miR-730a-5p_R-1	TCCTCATTGTGCATGCTGTGT	down	XLOC_005472	up
rno-miR-122-5p_L+3	ATCTGGAGTGTGACAATGGTGTTTG	up	XLOC_005478	up
PC-5p-45063_62	AAGGATAACTACAACTGTACTT	up	XLOC_005493	up
aca-miR-200b-3p_R+2	TAATACTGCCTGGTAATGATGAAT	up	XLOC_005493	up
dre-miR-142a-5p	CATAAAGTAGAAAGCACTACT	down	XLOC_005493	up
dre-miR-22a-3p	AAGCTGCCAGCTGAAGAACTGT	up	XLOC_005493	up
ssa-miR-16b-5p_R-1_1ss21TC	TAGCAGCACGTAAATATTGGC	down	XLOC_005493	up
ssa-miR-7a-5p	TGGAAGACTAGTGATTTTGTTGT	up	XLOC_005493	up
ssa-miR-7a-5p_R+1	TGGAAGACTAGTGATTTTGTTGTT	up	XLOC_005493	up
dre-miR-142a-3p_R-1	TGTAGTGTTTCCTACTTTATGG	down	XLOC_005494	up
ssa-miR-1338-5p_R+1	AGGACTGTCCAACCTGAGAATG	down	XLOC_005494	up
aca-miR-200b-3p_R+2	TAATACTGCCTGGTAATGATGAAT	up	XLOC_005515	down
dre-miR-1	TGGAATGTAAAGAAGTATGTAT	up	XLOC_005515	down
dre-miR-21_1ss23CA	TAGCTTATCAGACTGGTGTTGGA	up	XLOC_005515	down
ssa-miR-206-3p	TGGAATGTAAGGAAGTGTGTGG	up	XLOC_005515	down
ssc-miR-206	TGGAATGTAAGGAAGTGTGTGA	up	XLOC_005515	down
ssa-miR-730a-5p_R-1	TCCTCATTGTGCATGCTGTGT	down	XLOC_005533	up
PC-3p-50929_43	TGGAAGTGTCAGAAATTCTGAGT	up	XLOC_005609	up
ssa-miR-16b-5p_R-1_1ss21TC	TAGCAGCACGTAAATATTGGC	down	XLOC_005609	up
dre-miR-133a-3p_L-1R+1	TTGGTCCCCTTCAACCAGCTGT	up	XLOC_005628	up
dre-miR-133b-3p_R-1	TTTGGTCCCCTTCAACCAGCT	up	XLOC_005628	up
oha-miR-133b-3p	TTTGGTCCCCTTCAACCAGCTAT	up	XLOC_005628	up
ssa-miR-199a-3p_R+2	ACAGTAGTCTGCACATTGGTTTT	up	XLOC_005628	up
ssa-miR-26d-5p_L+1_1ss13TC	CTTCAAGTAATCCAGGATAGGCT	up	XLOC_005628	up
ola-miR-199a-3p_L+1	AACAGTAGTCTGCACATTGGTTA	up	XLOC_005741	down
dre-miR-140-3p_L-1	ACCACAGGGTAGAACCACGGAC	up	XLOC_005765	up
ssa-miR-1338-5p_R+1	AGGACTGTCCAACCTGAGAATG	down	XLOC_005765	up
PC-5p-27517_164	TACATGCAGAGGTGGAGCAAGA	up	XLOC_005770	up
dre-miR-1	TGGAATGTAAAGAAGTATGTAT	up	XLOC_005770	up
ssa-miR-1-4-5p	ACATACTTCTTTATATGCCCATA	up	XLOC_005770	up
ssa-miR-206-3p	TGGAATGTAAGGAAGTGTGTGG	up	XLOC_005770	up
ssc-miR-206	TGGAATGTAAGGAAGTGTGTGA	up	XLOC_005770	up
dre-miR-194a_R+2	TGTAACAGCAACTCCATGTGGAT	up	XLOC_005806	down
tni-miR-194_R+1	TGTAACAGCAACTCCATGTGGA	up	XLOC_005806	down
PC-3p-11630_419	ATGAGGAAAAGAAGTTAGGAGA	down	XLOC_005815	down
ssa-miR-199a-3p_R+2	ACAGTAGTCTGCACATTGGTTTT	up	XLOC_005815	down
dre-let-7d-5p	TGAGGTAGTTGGTTGTATGGTT	up	XLOC_005833	down
dre-miR-194a_R+2	TGTAACAGCAACTCCATGTGGAT	up	XLOC_005833	down
mmu-let-7j_1ss8TG	TGAGGTAGTAGTTTGTGCTGTTAT	up	XLOC_005833	down
ssa-miR-16b-5p_R-1_1ss21TC	TAGCAGCACGTAAATATTGGC	down	XLOC_005833	down
tni-let-7j_1ss11TG	TGAGGTAGTTGTTTGTACAGTT	up	XLOC_005833	down
tni-miR-194_R+1	TGTAACAGCAACTCCATGTGGA	up	XLOC_005833	down
dre-miR-24_R+2_1	TGGCTCAGTTCAGCAGGAACAGAA	up	XLOC_005858	down
dre-miR-24_R+2_2	TGGCTCAGTTCAGCAGGAACAGTT	up	XLOC_005858	down
ola-miR-199a-3p_L+1	AACAGTAGTCTGCACATTGGTTA	up	XLOC_005858	down
ssa-miR-16b-5p_R-1_1ss21TC	TAGCAGCACGTAAATATTGGC	down	XLOC_005858	down
dre-let-7d-5p	TGAGGTAGTTGGTTGTATGGTT	up	XLOC_005938	up
dre-miR-24_R+2_1	TGGCTCAGTTCAGCAGGAACAGAA	up	XLOC_005938	up
dre-miR-24_R+2_2	TGGCTCAGTTCAGCAGGAACAGTT	up	XLOC_005938	up
mmu-let-7j_1ss8TG	TGAGGTAGTAGTTTGTGCTGTTAT	up	XLOC_005938	up
ssa-miR-199a-3p_R+2	ACAGTAGTCTGCACATTGGTTTT	up	XLOC_005938	up

tni-let-7j_1ss11TG	TGAGGTAGTTGTTTGTACAGTT	up	XLOC_005938	up
ola-miR-146a-5p_1ss24TA	TGAGAACTGAATTCCATAGATGGAA	up	XLOC_005940	up
ola-miR-194-3p_1ss20CT	CCAGTGGAGGTGCTGTTACTTG	up	XLOC_005968	down
ola-mir-100-2-p3	CAAGCTCGTATCTATAGGTATG	down	XLOC_005968	down
PC-3p-50929_43	TGGAAGTGTCAGAAATTCTGAGT	up	XLOC_005994	down
dre-let-7d-5p	TGAGGTAGTTGGTTGTATGGTT	up	XLOC_005994	down
dre-miR-24_R+2_1	TGGCTCAGTTCAGCAGGAACAGAA	up	XLOC_005994	down
dre-miR-24_R+2_2	TGGCTCAGTTCAGCAGGAACAGTT	up	XLOC_005994	down
mmu-let-7j_1ss8TG	TGAGGTAGTAGTTTGTGCTGTTAT	up	XLOC_005994	down
ola-miR-199a-3p_L+1	AACAGTAGTCTGCACATTGGTTA	up	XLOC_005994	down
ssa-miR-1-4-5p	ACATACTTCTTTATATGCCCATA	up	XLOC_005994	down
tni-let-7j_1ss11TG	TGAGGTAGTTGTTTGTACAGTT	up	XLOC_005994	down
dre-miR-142a-3p_R-1	TGTAGTGTTTCCTACTTTATGG	down	XLOC_006006	up
PC-3p-41259_77	TGGCCATTAACTGCTAACCTTC	up	XLOC_006020	up
PC-5p-45063_62	AAGGATAACTACAACTGTACTT	up	XLOC_006020	up
PC-5p-27517_164	TACATGCAGAGGTGGAGCAAGA	up	XLOC_006060	up
dre-miR-122	TGGAGTGTGACAATGGTGTTTG	up	XLOC_006060	up
xtr-miR-122_L+1R-1	CTGGAGTGTGACAATGGTGTTTG	up	XLOC_006060	up
PC-5p-45063_62	AAGGATAACTACAACTGTACTT	up	XLOC_006089	up
dre-miR-142a-5p	CATAAAGTAGAAAGCACTACT	down	XLOC_006089	up
ola-miR-199a-3p_L+1	AACAGTAGTCTGCACATTGGTTA	up	XLOC_006089	up
PC-5p-8690_526	GATGTTGAGTATCAAACTGTAT	down	XLOC_006091	down
dre-miR-140-3p_L-1	ACCACAGGGTAGAACCACGGAC	up	XLOC_006091	down
ssa-miR-730a-5p_R-1	TCCTCATTGTGCATGCTGTGT	down	XLOC_006091	down
dre-miR-133a-3p_L-1R+1	TTGGTCCCCTTCAACCAGCTGT	up	XLOC_006096	down
dre-miR-22a-3p	AAGCTGCCAGCTGAAGAACTGT	up	XLOC_006120	up
PC-3p-11630_419	ATGAGGAAAAGAAGTTAGGAGA	down	XLOC_006125	up
dre-miR-21_1ss23CA	TAGCTTATCAGACTGGTGTTGGA	up	XLOC_006130	up
PC-3p-11630_419	ATGAGGAAAAGAAGTTAGGAGA	down	XLOC_006147	up
PC-5p-27517_164	TACATGCAGAGGTGGAGCAAGA	up	XLOC_006147	up
aca-miR-200b-3p_R+2	TAATACTGCCTGGTAATGATGAAT	up	XLOC_006147	up
dre-miR-194a_R+2	TGTAACAGCAACTCCATGTGGAT	up	XLOC_006147	up
tni-miR-194_R+1	TGTAACAGCAACTCCATGTGGA	up	XLOC_006147	up
dre-miR-194a_R+2	TGTAACAGCAACTCCATGTGGAT	up	XLOC_006178	up
ssa-miR-16b-5p_R-1_1ss21TC	TAGCAGCACGTAAATATTGGC	down	XLOC_006178	up
tni-miR-194_R+1	TGTAACAGCAACTCCATGTGGA	up	XLOC_006178	up
PC-3p-50929_43	TGGAAGTGTCAGAAATTCTGAGT	up	XLOC_006189	up
PC-5p-8690_526	GATGTTGAGTATCAAACTGTAT	down	XLOC_006204	down
dre-miR-21_1ss23CA	TAGCTTATCAGACTGGTGTTGGA	up	XLOC_006211	down
ola-miR-199a-3p_L+1	AACAGTAGTCTGCACATTGGTTA	up	XLOC_006211	down
dre-miR-22a-3p	AAGCTGCCAGCTGAAGAACTGT	up	XLOC_006222	up
ola-miR-199a-3p_L+1	AACAGTAGTCTGCACATTGGTTA	up	XLOC_006222	up
aca-miR-200b-3p_R+2	TAATACTGCCTGGTAATGATGAAT	up	XLOC_006238	down
dre-miR-1	TGGAATGTAAAGAAGTATGTAT	up	XLOC_006238	down
dre-miR-142a-3p_R-1	TGTAGTGTTTCCTACTTTATGG	down	XLOC_006238	down
ssa-miR-206-3p	TGGAATGTAAGGAAGTGTGTGG	up	XLOC_006238	down
ssc-miR-206	TGGAATGTAAGGAAGTGTGTGA	up	XLOC_006238	down
ola-miR-194-3p_1ss20CT	CCAGTGGAGGTGCTGTTACTTG	up	XLOC_006303	down
PC-5p-27517_164	TACATGCAGAGGTGGAGCAAGA	up	XLOC_006315	down
dre-miR-1	TGGAATGTAAAGAAGTATGTAT	up	XLOC_006315	down
dre-miR-133a-3p_L-1R+1	TTGGTCCCCTTCAACCAGCTGT	up	XLOC_006315	down
dre-miR-133b-3p_R-1	TTTGGTCCCCTTCAACCAGCT	up	XLOC_006315	down
oha-miR-133b-3p	TTTGGTCCCCTTCAACCAGCTAT	up	XLOC_006315	down
ssa-miR-206-3p	TGGAATGTAAGGAAGTGTGTGG	up	XLOC_006315	down

ssc-miR-206	TGGAATGTAAGGAAGTGTGTGA	up	XLOC_006315	down
PC-3p-11630_419	ATGAGGAAAAGAAGTTAGGAGA	down	XLOC_006319	up
aca-miR-200b-3p_R+2	TAATACTGCCTGGTAATGATGAAT	up	XLOC_006319	up
ola-miR-462_L-1R+4	TAACGGAACCCATAATGCAGCT	down	XLOC_006319	up
xtr-miR-122_L+1R-1	CTGGAGTGTGACAATGGTGTTTG	up	XLOC_006319	up
dre-miR-194a_R+2	TGTAACAGCAACTCCATGTGGAT	up	XLOC_006372	down
dre-miR-22a-3p	AAGCTGCCAGCTGAAGAACTGT	up	XLOC_006372	down
tni-miR-194_R+1	TGTAACAGCAACTCCATGTGGA	up	XLOC_006372	down
aca-miR-338-3p_R+2	TCCAGCATCAGTGATTTTGTTAA	up	XLOC_006384	up
dre-miR-22a-3p	AAGCTGCCAGCTGAAGAACTGT	up	XLOC_006384	up
ssa-miR-1338-5p_R+1	AGGACTGTCCAACCTGAGAATG	down	XLOC_006384	up
ssa-miR-26d-5p_L+1_1ss13TC	CTTCAAGTAATCCAGGATAGGCT	up	XLOC_006384	up
ssa-miR-730a-5p_R-1	TCCTCATTGTGCATGCTGTGT	down	XLOC_006384	up
ssa-miR-7a-5p	TGGAAGACTAGTGATTTTGTTGT	up	XLOC_006384	up
ssa-miR-7a-5p_R+1	TGGAAGACTAGTGATTTTGTTGTT	up	XLOC_006384	up
PC-5p-27517_164	TACATGCAGAGGTGGAGCAAGA	up	XLOC_006404	up
dre-miR-122	TGGAGTGTGACAATGGTGTTTG	up	XLOC_006450	down
dre-miR-142a-3p_R-1	TGTAGTGTTTCCTACTTTATGG	down	XLOC_006450	down
ola-miR-199a-3p_L+1	AACAGTAGTCTGCACATTGGTTA	up	XLOC_006450	down
ssa-miR-7a-5p	TGGAAGACTAGTGATTTTGTTGT	up	XLOC_006450	down
ssa-miR-7a-5p_R+1	TGGAAGACTAGTGATTTTGTTGTT	up	XLOC_006450	down
PC-3p-50929_43	TGGAAGTGTCAGAAATTCTGAGT	up	XLOC_006519	up
dre-miR-125b-5p_R+1	TCCCTGAGACCCTAACTTGTGAT	up	XLOC_006519	up
dre-miR-140-3p_L-1	ACCACAGGGTAGAACCACGGAC	up	XLOC_006519	up
sha-miR-125a_R+2	TCCCTGAGACCCTAACTTGTGAAA	up	XLOC_006519	up
ssa-miR-125b-5p_R-1	TCCCTGAGACCCTTAACCTGTG	up	XLOC_006519	up
ola-miR-146a-5p_1ss24TA	TGAGAACTGAATTCCATAGATGGAA	up	XLOC_006529	down
ola-mir-100-2-p3	CAAGCTCGTATCTATAGGTATG	down	XLOC_006529	down
ssa-miR-16b-5p_R-1_1ss21TC	TAGCAGCACGTAAATATTGGC	down	XLOC_006529	down
ssa-miR-199a-3p_R+2	ACAGTAGTCTGCACATTGGTTTT	up	XLOC_006529	down
rno-miR-122-5p_L+3	ATCTGGAGTGTGACAATGGTGTTTG	up	XLOC_006530	down
ssa-miR-16b-5p_R-1_1ss21TC	TAGCAGCACGTAAATATTGGC	down	XLOC_006539	up
dre-miR-133a-3p_L-1R+1	TTGGTCCCCTTCAACCAGCTGT	up	XLOC_006573	up
ola-miR-146a-5p_1ss24TA	TGAGAACTGAATTCCATAGATGGAA	up	XLOC_006573	up
dre-miR-125b-5p_R+1	TCCCTGAGACCCTAACTTGTGAT	up	XLOC_006577	up
ola-miR-194-3p_1ss20CT	CCAGTGGAGGTGCTGTTACTTG	up	XLOC_006577	up
sha-miR-125a_R+2	TCCCTGAGACCCTAACTTGTGAAA	up	XLOC_006577	up
ssa-miR-199a-3p_R+2	ACAGTAGTCTGCACATTGGTTTT	up	XLOC_006577	up
ola-miR-146a-5p_1ss24TA	TGAGAACTGAATTCCATAGATGGAA	up	XLOC_006580	down
ssa-miR-7a-5p	TGGAAGACTAGTGATTTTGTTGT	up	XLOC_006596	down
ssa-miR-7a-5p_R+1	TGGAAGACTAGTGATTTTGTTGTT	up	XLOC_006596	down
dre-miR-140-3p_L-1	ACCACAGGGTAGAACCACGGAC	up	XLOC_006601	up
PC-3p-50929_43	TGGAAGTGTCAGAAATTCTGAGT	up	XLOC_006665	up
PC-5p-27517_164	TACATGCAGAGGTGGAGCAAGA	up	XLOC_006665	up
dre-miR-122	TGGAGTGTGACAATGGTGTTTG	up	XLOC_006665	up
dre-miR-125b-5p_R+1	TCCCTGAGACCCTAACTTGTGAT	up	XLOC_006665	up
dre-miR-133b-3p_R-1	TTTGGTCCCCTTCAACCAGCT	up	XLOC_006665	up
dre-miR-22a-3p	AAGCTGCCAGCTGAAGAACTGT	up	XLOC_006665	up
oha-miR-133b-3p	TTTGGTCCCCTTCAACCAGCTAT	up	XLOC_006665	up
ola-miR-194-3p_1ss20CT	CCAGTGGAGGTGCTGTTACTTG	up	XLOC_006665	up
ola-miR-199a-3p_L+1	AACAGTAGTCTGCACATTGGTTA	up	XLOC_006665	up
sha-miR-125a_R+2	TCCCTGAGACCCTAACTTGTGAAA	up	XLOC_006665	up
ssa-miR-125b-5p_R-1	TCCCTGAGACCCTTAACCTGTG	up	XLOC_006665	up
ssa-miR-199a-3p_R+2	ACAGTAGTCTGCACATTGGTTTT	up	XLOC_006665	up

tni-miR-10c	TACCCTGTAGATCCGGATTTGT	up	XLOC_006665	up
xtr-miR-122_L+1R-1	CTGGAGTGTGACAATGGTGTTTG	up	XLOC_006665	up
ssa-miR-16b-5p_R-1_1ss21TC	TAGCAGCACGTAAATATTGGC	down	XLOC_006695	down
aca-miR-338-3p_R+2	TCCAGCATCAGTGATTTTGTTAA	up	XLOC_006751	down
dre-miR-22a-3p	AAGCTGCCAGCTGAAGAACTGT	up	XLOC_006751	down
ola-mir-100-2-p3	CAAGCTCGTATCTATAGGTATG	down	XLOC_006751	down
ssa-miR-1338-5p_R+1	AGGACTGTCCAACCTGAGAATG	down	XLOC_006751	down
ssa-miR-730a-5p_R-1	TCCTCATTGTGCATGCTGTGT	down	XLOC_006751	down
aca-miR-338-3p_R+2	TCCAGCATCAGTGATTTTGTTAA	up	XLOC_006801	up
ssa-miR-7a-5p	TGGAAGACTAGTGATTTTGTTGT	up	XLOC_006801	up
ssa-miR-7a-5p_R+1	TGGAAGACTAGTGATTTTGTTGTT	up	XLOC_006801	up
dre-miR-1	TGGAATGTAAAGAAGTATGTAT	up	XLOC_006808	up
ssa-miR-16b-5p_R-1_1ss21TC	TAGCAGCACGTAAATATTGGC	down	XLOC_006808	up
ssa-miR-206-3p	TGGAATGTAAGGAAGTGTGTGG	up	XLOC_006808	up
ssa-miR-26d-5p_L+1_1ss13TC	CTTCAAGTAATCCAGGATAGGCT	up	XLOC_006808	up
ssc-miR-206	TGGAATGTAAGGAAGTGTGTGA	up	XLOC_006808	up
PC-3p-11630_419	ATGAGGAAAAGAAGTTAGGAGA	down	XLOC_006828	up
PC-3p-50929_43	TGGAAGTGTCAGAAATTCTGAGT	up	XLOC_006828	up
ssa-miR-1338-5p_R+1	AGGACTGTCCAACCTGAGAATG	down	XLOC_006828	up
aca-miR-338-3p_R+2	TCCAGCATCAGTGATTTTGTTAA	up	XLOC_006842	up
PC-3p-41259_77	TGGCCATTAACTGCTAACCTTC	up	XLOC_006850	down
ssa-miR-26d-5p_L+1_1ss13TC	CTTCAAGTAATCCAGGATAGGCT	up	XLOC_006850	down
ssa-miR-7a-5p	TGGAAGACTAGTGATTTTGTTGT	up	XLOC_006850	down
ssa-miR-7a-5p_R+1	TGGAAGACTAGTGATTTTGTTGTT	up	XLOC_006850	down
PC-3p-11630_419	ATGAGGAAAAGAAGTTAGGAGA	down	XLOC_006883	up
ola-miR-194-3p_1ss20CT	CCAGTGGAGGTGCTGTTACTTG	up	XLOC_006883	up
PC-5p-45063_62	AAGGATAACTACAACTGTACTT	up	XLOC_006913	down
dre-miR-24_R+2_1	TGGCTCAGTTCAGCAGGAACAGAA	up	XLOC_006913	down
dre-miR-24_R+2_2	TGGCTCAGTTCAGCAGGAACAGTT	up	XLOC_006913	down
PC-3p-50929_43	TGGAAGTGTCAGAAATTCTGAGT	up	XLOC_006921	up
PC-5p-27517_164	TACATGCAGAGGTGGAGCAAGA	up	XLOC_006921	up
ssa-miR-16b-5p_R-1_1ss21TC	TAGCAGCACGTAAATATTGGC	down	XLOC_006921	up
ssa-miR-7a-5p	TGGAAGACTAGTGATTTTGTTGT	up	XLOC_006921	up
ssa-miR-7a-5p_R+1	TGGAAGACTAGTGATTTTGTTGTT	up	XLOC_006921	up
aca-miR-200b-3p_R+2	TAATACTGCCTGGTAATGATGAAT	up	XLOC_006991	up
aca-miR-338-3p_R+2	TCCAGCATCAGTGATTTTGTTAA	up	XLOC_006991	up
dre-miR-24_R+2_1	TGGCTCAGTTCAGCAGGAACAGAA	up	XLOC_006991	up
dre-miR-24_R+2_2	TGGCTCAGTTCAGCAGGAACAGTT	up	XLOC_006991	up
ola-miR-146a-5p_1ss24TA	TGAGAACTGAATTCCATAGATGGAA	up	XLOC_006991	up
ssa-miR-730a-5p_R-1	TCCTCATTGTGCATGCTGTGT	down	XLOC_006991	up
PC-3p-11630_419	ATGAGGAAAAGAAGTTAGGAGA	down	XLOC_007002	up
PC-3p-11630_419	ATGAGGAAAAGAAGTTAGGAGA	down	XLOC_007042	down
PC-5p-45063_62	AAGGATAACTACAACTGTACTT	up	XLOC_007042	down
dre-let-7d-5p	TGAGGTAGTTGGTTGTATGGTT	up	XLOC_007042	down
dre-miR-142a-5p	CATAAAGTAGAAAGCACTACT	down	XLOC_007042	down
mmu-let-7j_1ss8TG	TGAGGTAGTAGTTTGTGCTGTTAT	up	XLOC_007042	down
ssa-miR-16b-5p_R-1_1ss21TC	TAGCAGCACGTAAATATTGGC	down	XLOC_007042	down
tni-let-7j_1ss11TG	TGAGGTAGTTGTTTGTACAGTT	up	XLOC_007042	down
dre-miR-194a_R+2	TGTAACAGCAACTCCATGTGGAT	up	XLOC_007047	down
dre-miR-21_1ss23CA	TAGCTTATCAGACTGGTGTTGGA	up	XLOC_007047	down
rno-miR-122-5p_L+3	ATCTGGAGTGTGACAATGGTGTTTG	up	XLOC_007047	down
ssa-miR-26d-5p_L+1_1ss13TC	CTTCAAGTAATCCAGGATAGGCT	up	XLOC_007047	down
ssa-miR-7a-5p	TGGAAGACTAGTGATTTTGTTGT	up	XLOC_007047	down
ssa-miR-7a-5p_R+1	TGGAAGACTAGTGATTTTGTTGTT	up	XLOC_007047	down

tni-miR-10c	TACCCTGTAGATCCGGATTTGT	up	XLOC_007047	down
tni-miR-194_R+1	TGTAACAGCAACTCCATGTGGA	up	XLOC_007047	down
dre-miR-125b-5p_R+1	TCCCTGAGACCCTAACTTGTGAT	up	XLOC_007080	down
dre-miR-24_R+2_1	TGGCTCAGTTCAGCAGGAACAGAA	up	XLOC_007080	down
dre-miR-24_R+2_2	TGGCTCAGTTCAGCAGGAACAGTT	up	XLOC_007080	down
ola-miR-194-3p_1ss20CT	CCAGTGGAGGTGCTGTTACTTG	up	XLOC_007080	down
ola-mir-100-2-p3	CAAGCTCGTATCTATAGGTATG	down	XLOC_007080	down
sha-miR-125a_R+2	TCCCTGAGACCCTAACTTGTGAAA	up	XLOC_007080	down
ssa-miR-125b-5p_R-1	TCCCTGAGACCCTTAACCTGTG	up	XLOC_007080	down
ssa-miR-730a-5p_R-1	TCCTCATTGTGCATGCTGTGT	down	XLOC_007080	down
xtr-miR-122_L+1R-1	CTGGAGTGTGACAATGGTGTTTG	up	XLOC_007080	down
PC-3p-50929_43	TGGAAGTGTCAGAAATTCTGAGT	up	XLOC_007133	down
dre-miR-194a_R+2	TGTAACAGCAACTCCATGTGGAT	up	XLOC_007133	down
ola-miR-194-3p_1ss20CT	CCAGTGGAGGTGCTGTTACTTG	up	XLOC_007133	down
tni-miR-194_R+1	TGTAACAGCAACTCCATGTGGA	up	XLOC_007133	down
PC-3p-50929_43	TGGAAGTGTCAGAAATTCTGAGT	up	XLOC_007143	down
ssa-miR-16b-5p_R-1_1ss21TC	TAGCAGCACGTAAATATTGGC	down	XLOC_007143	down
PC-5p-27517_164	TACATGCAGAGGTGGAGCAAGA	up	XLOC_007180	down
rno-miR-122-5p_L+3	ATCTGGAGTGTGACAATGGTGTTTG	up	XLOC_007180	down
ssa-miR-730a-5p_R-1	TCCTCATTGTGCATGCTGTGT	down	XLOC_007180	down
PC-5p-45063_62	AAGGATAACTACAACTGTACTT	up	XLOC_007236	up
ola-miR-194-3p_1ss20CT	CCAGTGGAGGTGCTGTTACTTG	up	XLOC_007237	up
ssa-miR-730a-5p_R-1	TCCTCATTGTGCATGCTGTGT	down	XLOC_007242	up
ssa-miR-7132a-5p_R+1	GACTTGGTCAAAGCTCCTCAGTT	down	XLOC_007243	up
ssa-miR-7132b-5p	GACTTGGTCAAAGCTCCTCAGC	down	XLOC_007243	up
PC-3p-11630_419	ATGAGGAAAAGAAGTTAGGAGA	down	XLOC_007246	up
PC-3p-50929_43	TGGAAGTGTCAGAAATTCTGAGT	up	XLOC_007274	down
PC-5p-45063_62	AAGGATAACTACAACTGTACTT	up	XLOC_007274	down
aca-miR-338-3p_R+2	TCCAGCATCAGTGATTTTGTTAA	up	XLOC_007274	down
xtr-miR-122_L+1R-1	CTGGAGTGTGACAATGGTGTTTG	up	XLOC_007274	down
dre-miR-194a_R+2	TGTAACAGCAACTCCATGTGGAT	up	XLOC_007278	down
ssa-miR-1-4-5p	ACATACTTCTTTATATGCCCATA	up	XLOC_007278	down
tni-miR-194_R+1	TGTAACAGCAACTCCATGTGGA	up	XLOC_007278	down
dre-miR-194a_R+2	TGTAACAGCAACTCCATGTGGAT	up	XLOC_007291	down
dre-miR-21_1ss23CA	TAGCTTATCAGACTGGTGTTGGA	up	XLOC_007291	down
tni-miR-194_R+1	TGTAACAGCAACTCCATGTGGA	up	XLOC_007291	down
dre-miR-142a-3p_R-1	TGTAGTGTTTCCTACTTTATGG	down	XLOC_007319	up
ola-miR-199a-3p_L+1	AACAGTAGTCTGCACATTGGTTA	up	XLOC_007319	up
ssa-miR-199a-3p_R+2	ACAGTAGTCTGCACATTGGTTTT	up	XLOC_007319	up
PC-3p-11630_419	ATGAGGAAAAGAAGTTAGGAGA	down	XLOC_007346	down
ssa-miR-1338-5p_R+1	AGGACTGTCCAACCTGAGAATG	down	XLOC_007346	down
ola-mir-100-2-p3	CAAGCTCGTATCTATAGGTATG	down	XLOC_007371	up
ssa-miR-16b-5p_R-1_1ss21TC	TAGCAGCACGTAAATATTGGC	down	XLOC_007371	up
dre-miR-22a-3p	AAGCTGCCAGCTGAAGAACTGT	up	XLOC_007378	up
ssa-miR-16b-5p_R-1_1ss21TC	TAGCAGCACGTAAATATTGGC	down	XLOC_007378	up
ssa-miR-26a-4-3p	CCTATTCTTGATTACTTGTTTC	down	XLOC_007378	up
ola-miR-194-3p_1ss20CT	CCAGTGGAGGTGCTGTTACTTG	up	XLOC_007386	down
dre-miR-142a-3p_R-1	TGTAGTGTTTCCTACTTTATGG	down	XLOC_007391	down
ola-miR-199a-3p_L+1	AACAGTAGTCTGCACATTGGTTA	up	XLOC_007391	down
ssa-miR-26d-5p_L+1_1ss13TC	CTTCAAGTAATCCAGGATAGGCT	up	XLOC_007397	down
ssa-miR-7132a-5p_R+1	GACTTGGTCAAAGCTCCTCAGTT	down	XLOC_007397	down
ssa-miR-7132b-5p	GACTTGGTCAAAGCTCCTCAGC	down	XLOC_007397	down
rno-miR-122-5p_L+3	ATCTGGAGTGTGACAATGGTGTTTG	up	XLOC_007418	down
dre-miR-133b-3p_R-1	TTTGGTCCCCTTCAACCAGCT	up	XLOC_007437	down

oha-miR-133b-3p	TTTGGTCCCCTTCAACCAGCTAT	up	XLOC_007437	down
ssa-miR-7132a-5p_R+1	GACTTGGTCAAAGCTCCTCAGTT	down	XLOC_007437	down
ssa-miR-7132b-5p	GACTTGGTCAAAGCTCCTCAGC	down	XLOC_007437	down
PC-3p-11630_419	ATGAGGAAAAGAAGTTAGGAGA	down	XLOC_007480	down
PC-3p-41259_77	TGGCCATTAACTGCTAACCTTC	up	XLOC_007480	down
dre-miR-194a_R+2	TGTAACAGCAACTCCATGTGGAT	up	XLOC_007480	down
ola-miR-194-3p_1ss20CT	CCAGTGGAGGTGCTGTTACTTG	up	XLOC_007480	down
ssa-miR-1338-5p_R+1	AGGACTGTCCAACCTGAGAATG	down	XLOC_007480	down
ssa-miR-730a-5p_R-1	TCCTCATTGTGCATGCTGTGT	down	XLOC_007480	down
tni-miR-194_R+1	TGTAACAGCAACTCCATGTGGA	up	XLOC_007480	down
PC-5p-27517_164	TACATGCAGAGGTGGAGCAAGA	up	XLOC_007525	up
aca-miR-200b-3p_R+2	TAATACTGCCTGGTAATGATGAAT	up	XLOC_007525	up
PC-5p-27517_164	TACATGCAGAGGTGGAGCAAGA	up	XLOC_007650	down
mmu-let-7j_1ss8TG	TGAGGTAGTAGTTTGTGCTGTTAT	up	XLOC_007650	down
ola-miR-199a-3p_L+1	AACAGTAGTCTGCACATTGGTTA	up	XLOC_007650	down
PC-3p-11630_419	ATGAGGAAAAGAAGTTAGGAGA	down	XLOC_007716	up
dre-miR-21_1ss23CA	TAGCTTATCAGACTGGTGTTGGA	up	XLOC_007743	up
ola-miR-146a-5p_1ss24TA	TGAGAACTGAATTCCATAGATGGAA	up	XLOC_007743	up
ssa-miR-7a-5p	TGGAAGACTAGTGATTTTGTTGT	up	XLOC_007743	up
ssa-miR-7a-5p_R+1	TGGAAGACTAGTGATTTTGTTGTT	up	XLOC_007743	up
ola-miR-199a-3p_L+1	AACAGTAGTCTGCACATTGGTTA	up	XLOC_007745	down
ssa-miR-199a-3p_R+2	ACAGTAGTCTGCACATTGGTTTT	up	XLOC_007745	down
aca-miR-338-3p_R+2	TCCAGCATCAGTGATTTTGTTAA	up	XLOC_007764	up
dre-miR-140-3p_L-1	ACCACAGGGTAGAACCACGGAC	up	XLOC_007764	up
dre-miR-194a_R+2	TGTAACAGCAACTCCATGTGGAT	up	XLOC_007764	up
rno-miR-122-5p_L+3	ATCTGGAGTGTGACAATGGTGTTTG	up	XLOC_007764	up
tni-miR-194_R+1	TGTAACAGCAACTCCATGTGGA	up	XLOC_007764	up
xtr-miR-122_L+1R-1	CTGGAGTGTGACAATGGTGTTTG	up	XLOC_007764	up
PC-3p-41259_77	TGGCCATTAACTGCTAACCTTC	up	XLOC_007794	down
PC-5p-27517_164	TACATGCAGAGGTGGAGCAAGA	up	XLOC_007806	up
ssa-miR-16b-5p_R-1_1ss21TC	TAGCAGCACGTAAATATTGGC	down	XLOC_007806	up
xtr-miR-122_L+1R-1	CTGGAGTGTGACAATGGTGTTTG	up	XLOC_007806	up
PC-5p-27517_164	TACATGCAGAGGTGGAGCAAGA	up	XLOC_007953	up
dre-miR-133b-3p_R-1	TTTGGTCCCCTTCAACCAGCT	up	XLOC_007953	up
dre-miR-140-3p_L-1	ACCACAGGGTAGAACCACGGAC	up	XLOC_007953	up
oha-miR-133b-3p	TTTGGTCCCCTTCAACCAGCTAT	up	XLOC_007953	up
xtr-miR-122_L+1R-1	CTGGAGTGTGACAATGGTGTTTG	up	XLOC_007955	down
ssa-miR-7132b-3p	TGAGGCGTTTAGAACAAGTTCA	down	XLOC_007968	down
dre-miR-22a-3p	AAGCTGCCAGCTGAAGAACTGT	up	XLOC_007999	up
ssa-miR-26d-5p_L+1_1ss13TC	CTTCAAGTAATCCAGGATAGGCT	up	XLOC_007999	up
dre-miR-133b-3p_R-1	TTTGGTCCCCTTCAACCAGCT	up	XLOC_008038	up
dre-miR-194a_R+2	TGTAACAGCAACTCCATGTGGAT	up	XLOC_008038	up
oha-miR-133b-3p	TTTGGTCCCCTTCAACCAGCTAT	up	XLOC_008038	up
ssa-miR-7132a-5p_R+1	GACTTGGTCAAAGCTCCTCAGTT	down	XLOC_008038	up
ssa-miR-7132b-5p	GACTTGGTCAAAGCTCCTCAGC	down	XLOC_008038	up
tni-miR-194_R+1	TGTAACAGCAACTCCATGTGGA	up	XLOC_008038	up
ola-miR-199a-3p_L+1	AACAGTAGTCTGCACATTGGTTA	up	XLOC_008090	up
ssa-miR-199a-3p_R+2	ACAGTAGTCTGCACATTGGTTTT	up	XLOC_008091	up
ssa-miR-26d-5p_L+1_1ss13TC	CTTCAAGTAATCCAGGATAGGCT	up	XLOC_008091	up
dre-miR-122	TGGAGTGTGACAATGGTGTTTG	up	XLOC_008143	up
ola-miR-199a-3p_L+1	AACAGTAGTCTGCACATTGGTTA	up	XLOC_008143	up
xtr-miR-122_L+1R-1	CTGGAGTGTGACAATGGTGTTTG	up	XLOC_008143	up
dre-miR-194a_R+2	TGTAACAGCAACTCCATGTGGAT	up	XLOC_008160	down
ssa-miR-16b-5p_R-1_1ss21TC	TAGCAGCACGTAAATATTGGC	down	XLOC_008160	down

tni-miR-194_R+1	TGTAACAGCAACTCCATGTGGA	up	XLOC_008160	down
dre-miR-125b-5p_R+1	TCCCTGAGACCCTAACTTGTGAT	up	XLOC_008186	down
sha-miR-125a_R+2	TCCCTGAGACCCTAACTTGTGAAA	up	XLOC_008186	down
ssa-miR-125b-5p_R-1	TCCCTGAGACCCTTAACCTGTG	up	XLOC_008186	down
tni-miR-10c	TACCCTGTAGATCCGGATTTGT	up	XLOC_008186	down
dre-miR-22a-3p	AAGCTGCCAGCTGAAGAACTGT	up	XLOC_008191	down
aca-miR-338-3p_R+2	TCCAGCATCAGTGATTTTGTTAA	up	XLOC_008196	down
dre-miR-194a_R+2	TGTAACAGCAACTCCATGTGGAT	up	XLOC_008196	down
dre-miR-21_1ss23CA	TAGCTTATCAGACTGGTGTTGGA	up	XLOC_008196	down
ola-miR-146a-5p_1ss24TA	TGAGAACTGAATTCCATAGATGGAA	up	XLOC_008196	down
ssa-miR-16b-5p_R-1_1ss21TC	TAGCAGCACGTAAATATTGGC	down	XLOC_008196	down
tni-miR-194_R+1	TGTAACAGCAACTCCATGTGGA	up	XLOC_008196	down
PC-5p-45063_62	AAGGATAACTACAACTGTACTT	up	XLOC_008277	up
dre-miR-1	TGGAATGTAAAGAAGTATGTAT	up	XLOC_008277	up
ssa-miR-206-3p	TGGAATGTAAGGAAGTGTGTGG	up	XLOC_008277	up
ssc-miR-206	TGGAATGTAAGGAAGTGTGTGA	up	XLOC_008277	up
tni-miR-10c	TACCCTGTAGATCCGGATTTGT	up	XLOC_008277	up
dre-miR-1	TGGAATGTAAAGAAGTATGTAT	up	XLOC_008289	up
ssa-miR-206-3p	TGGAATGTAAGGAAGTGTGTGG	up	XLOC_008289	up
ssc-miR-206	TGGAATGTAAGGAAGTGTGTGA	up	XLOC_008289	up
xtr-miR-122_L+1R-1	CTGGAGTGTGACAATGGTGTTTG	up	XLOC_008289	up
PC-3p-11630_419	ATGAGGAAAAGAAGTTAGGAGA	down	XLOC_008299	down
ssa-miR-1-4-5p	ACATACTTCTTTATATGCCCATA	up	XLOC_008304	up
ssa-miR-730a-5p_R-1	TCCTCATTGTGCATGCTGTGT	down	XLOC_008304	up
dre-let-7d-5p	TGAGGTAGTTGGTTGTATGGTT	up	XLOC_008353	up
dre-miR-1	TGGAATGTAAAGAAGTATGTAT	up	XLOC_008353	up
dre-miR-22a-3p	AAGCTGCCAGCTGAAGAACTGT	up	XLOC_008353	up
mmu-let-7j_1ss8TG	TGAGGTAGTAGTTTGTGCTGTTAT	up	XLOC_008353	up
ssa-miR-16b-5p_R-1_1ss21TC	TAGCAGCACGTAAATATTGGC	down	XLOC_008353	up
ssa-miR-206-3p	TGGAATGTAAGGAAGTGTGTGG	up	XLOC_008353	up
ssa-miR-26a-4-3p	CCTATTCTTGATTACTTGTTTC	down	XLOC_008353	up
ssc-miR-206	TGGAATGTAAGGAAGTGTGTGA	up	XLOC_008353	up
tni-let-7j_1ss11TG	TGAGGTAGTTGTTTGTACAGTT	up	XLOC_008353	up
ola-mir-100-2-p3	CAAGCTCGTATCTATAGGTATG	down	XLOC_008375	down
ola-mir-100-2-p3	CAAGCTCGTATCTATAGGTATG	down	XLOC_008378	up
dre-miR-142a-5p	CATAAAGTAGAAAGCACTACT	down	XLOC_008388	down
ola-mir-100-2-p3	CAAGCTCGTATCTATAGGTATG	down	XLOC_008388	down
dre-miR-142a-3p_R-1	TGTAGTGTTTCCTACTTTATGG	down	XLOC_008400	up
dre-miR-142a-5p	CATAAAGTAGAAAGCACTACT	down	XLOC_008400	up
ola-miR-199a-3p_L+1	AACAGTAGTCTGCACATTGGTTA	up	XLOC_008400	up
ssa-miR-199a-3p_R+2	ACAGTAGTCTGCACATTGGTTTT	up	XLOC_008400	up
dre-miR-22a-3p	AAGCTGCCAGCTGAAGAACTGT	up	XLOC_008401	up
ssa-miR-26a-4-3p	CCTATTCTTGATTACTTGTTTC	down	XLOC_008487	down
ola-miR-194-3p_1ss20CT	CCAGTGGAGGTGCTGTTACTTG	up	XLOC_008521	down
PC-5p-27517_164	TACATGCAGAGGTGGAGCAAGA	up	XLOC_008552	up
ssa-miR-730a-5p_R-1	TCCTCATTGTGCATGCTGTGT	down	XLOC_008552	up
PC-3p-11630_419	ATGAGGAAAAGAAGTTAGGAGA	down	XLOC_008555	down
dre-miR-21_1ss23CA	TAGCTTATCAGACTGGTGTTGGA	up	XLOC_008555	down
ssa-miR-730a-5p_R-1	TCCTCATTGTGCATGCTGTGT	down	XLOC_008555	down
aca-miR-200b-3p_R+2	TAATACTGCCTGGTAATGATGAAT	up	XLOC_008599	up
ola-miR-194-3p_1ss20CT	CCAGTGGAGGTGCTGTTACTTG	up	XLOC_008599	up
dre-miR-1	TGGAATGTAAAGAAGTATGTAT	up	XLOC_008633	down
dre-miR-194a_R+2	TGTAACAGCAACTCCATGTGGAT	up	XLOC_008633	down
dre-miR-24_R+2_1	TGGCTCAGTTCAGCAGGAACAGAA	up	XLOC_008633	down

dre-miR-24_R+2_2	TGGCTCAGTTCAGCAGGAACAGTT	up	XLOC_008633	down
ssa-miR-16b-5p_R-1_1ss21TC	TAGCAGCACGTAAATATTGGC	down	XLOC_008633	down
ssa-miR-206-3p	TGGAATGTAAGGAAGTGTGTGG	up	XLOC_008633	down
ssa-miR-26d-5p_L+1_1ss13TC	CTTCAAGTAATCCAGGATAGGCT	up	XLOC_008633	down
ssc-miR-206	TGGAATGTAAGGAAGTGTGTGA	up	XLOC_008633	down
tni-miR-194_R+1	TGTAACAGCAACTCCATGTGGA	up	XLOC_008633	down
ola-miR-146a-5p_1ss24TA	TGAGAACTGAATTCCATAGATGGAA	up	XLOC_008696	down
ola-miR-199a-3p_L+1	AACAGTAGTCTGCACATTGGTTA	up	XLOC_008696	down
ssa-miR-16b-5p_R-1_1ss21TC	TAGCAGCACGTAAATATTGGC	down	XLOC_008696	down
ola-miR-146a-5p_1ss24TA	TGAGAACTGAATTCCATAGATGGAA	up	XLOC_008709	up
ssa-miR-1338-5p_R+1	AGGACTGTCCAACCTGAGAATG	down	XLOC_008709	up
PC-5p-8690_526	GATGTTGAGTATCAAACTGTAT	down	XLOC_008738	up
aca-miR-338-3p_R+2	TCCAGCATCAGTGATTTTGTTAA	up	XLOC_008738	up
dre-miR-194a_R+2	TGTAACAGCAACTCCATGTGGAT	up	XLOC_008738	up
ola-miR-462_L-1R+4	TAACGGAACCCATAATGCAGCT	down	XLOC_008738	up
ssa-miR-16b-5p_R-1_1ss21TC	TAGCAGCACGTAAATATTGGC	down	XLOC_008738	up
tni-miR-194_R+1	TGTAACAGCAACTCCATGTGGA	up	XLOC_008738	up
dre-miR-133a-3p_L-1R+1	TTGGTCCCCTTCAACCAGCTGT	up	XLOC_008770	up
dre-miR-21_1ss23CA	TAGCTTATCAGACTGGTGTTGGA	up	XLOC_008770	up
ola-miR-199a-3p_L+1	AACAGTAGTCTGCACATTGGTTA	up	XLOC_008770	up
ssa-miR-199a-3p_R+2	ACAGTAGTCTGCACATTGGTTTT	up	XLOC_008770	up
ssa-miR-7132a-5p_R+1	GACTTGGTCAAAGCTCCTCAGTT	down	XLOC_008770	up
ssa-miR-7132b-5p	GACTTGGTCAAAGCTCCTCAGC	down	XLOC_008770	up
aca-miR-338-3p_R+2	TCCAGCATCAGTGATTTTGTTAA	up	XLOC_008788	down
dre-miR-140-3p_L-1	ACCACAGGGTAGAACCACGGAC	up	XLOC_008814	up
ssa-miR-7a-5p	TGGAAGACTAGTGATTTTGTTGT	up	XLOC_008814	up
ssa-miR-7a-5p_R+1	TGGAAGACTAGTGATTTTGTTGTT	up	XLOC_008814	up
dre-miR-142a-3p_R-1	TGTAGTGTTTCCTACTTTATGG	down	XLOC_008822	down
dre-miR-142a-5p	CATAAAGTAGAAAGCACTACT	down	XLOC_008822	down
ssa-miR-7a-5p	TGGAAGACTAGTGATTTTGTTGT	up	XLOC_008822	down
ssa-miR-7a-5p_R+1	TGGAAGACTAGTGATTTTGTTGTT	up	XLOC_008822	down
dre-miR-24_R+2_1	TGGCTCAGTTCAGCAGGAACAGAA	up	XLOC_008840	up
dre-miR-24_R+2_2	TGGCTCAGTTCAGCAGGAACAGTT	up	XLOC_008840	up
dre-miR-133b-3p_R-1	TTTGGTCCCCTTCAACCAGCT	up	XLOC_008917	down
dre-miR-142a-3p_R-1	TGTAGTGTTTCCTACTTTATGG	down	XLOC_008917	down
oha-miR-133b-3p	TTTGGTCCCCTTCAACCAGCTAT	up	XLOC_008917	down
ssa-miR-1338-5p_R+1	AGGACTGTCCAACCTGAGAATG	down	XLOC_008917	down
PC-5p-27517_164	TACATGCAGAGGTGGAGCAAGA	up	XLOC_008937	up
dre-miR-21_1ss23CA	TAGCTTATCAGACTGGTGTTGGA	up	XLOC_008937	up
ssa-miR-1-4-5p	ACATACTTCTTTATATGCCCATA	up	XLOC_008937	up
dre-miR-122	TGGAGTGTGACAATGGTGTTTG	up	XLOC_009011	down
dre-miR-125b-5p_R+1	TCCCTGAGACCCTAACTTGTGAT	up	XLOC_009011	down
dre-miR-22a-3p	AAGCTGCCAGCTGAAGAACTGT	up	XLOC_009011	down
ola-miR-199a-3p_L+1	AACAGTAGTCTGCACATTGGTTA	up	XLOC_009011	down
sha-miR-125a_R+2	TCCCTGAGACCCTAACTTGTGAAA	up	XLOC_009011	down
ssa-miR-125b-5p_R-1	TCCCTGAGACCCTTAACCTGTG	up	XLOC_009011	down
ssa-miR-26d-5p_L+1_1ss13TC	CTTCAAGTAATCCAGGATAGGCT	up	XLOC_009011	down
dre-miR-125b-5p_R+1	TCCCTGAGACCCTAACTTGTGAT	up	XLOC_009033	up
sha-miR-125a_R+2	TCCCTGAGACCCTAACTTGTGAAA	up	XLOC_009033	up
ssa-miR-125b-5p_R-1	TCCCTGAGACCCTTAACCTGTG	up	XLOC_009033	up
dre-miR-194a_R+2	TGTAACAGCAACTCCATGTGGAT	up	XLOC_009056	down
ssa-mir-15c-2-p3_1ss11CA	TGCGAACCATAATTTGCTGCTT	down	XLOC_009056	down
tni-miR-194_R+1	TGTAACAGCAACTCCATGTGGA	up	XLOC_009056	down
dre-miR-22a-3p	AAGCTGCCAGCTGAAGAACTGT	up	XLOC_009105	down

ola-miR-199a-3p_L+1	AACAGTAGTCTGCACATTGGTTA	up	XLOC_009105	down
ssa-miR-26d-5p_L+1_1ss13TC	CTTCAAGTAATCCAGGATAGGCT	up	XLOC_009105	down
PC-3p-50929_43	TGGAAGTGTCAGAAATTCTGAGT	up	XLOC_009111	down
PC-5p-27517_164	TACATGCAGAGGTGGAGCAAGA	up	XLOC_009111	down
rno-miR-122-5p_L+3	ATCTGGAGTGTGACAATGGTGTTTG	up	XLOC_009111	down
ssa-miR-1338-5p_R+1	AGGACTGTCCAACCTGAGAATG	down	XLOC_009111	down
ssa-miR-7132a-5p_R+1	GACTTGGTCAAAGCTCCTCAGTT	down	XLOC_009111	down
ssa-miR-7132b-5p	GACTTGGTCAAAGCTCCTCAGC	down	XLOC_009111	down
PC-3p-11630_419	ATGAGGAAAAGAAGTTAGGAGA	down	XLOC_009119	down
PC-5p-8690_526	GATGTTGAGTATCAAACTGTAT	down	XLOC_009119	down
ssa-miR-730a-5p_R-1	TCCTCATTGTGCATGCTGTGT	down	XLOC_009119	down
aca-miR-338-3p_R+2	TCCAGCATCAGTGATTTTGTTAA	up	XLOC_009126	up
dre-let-7d-5p	TGAGGTAGTTGGTTGTATGGTT	up	XLOC_009126	up
dre-miR-122	TGGAGTGTGACAATGGTGTTTG	up	XLOC_009126	up
mmu-let-7j_1ss8TG	TGAGGTAGTAGTTTGTGCTGTTAT	up	XLOC_009126	up
ssa-miR-16b-5p_R-1_1ss21TC	TAGCAGCACGTAAATATTGGC	down	XLOC_009126	up
tni-let-7j_1ss11TG	TGAGGTAGTTGTTTGTACAGTT	up	XLOC_009126	up
PC-3p-50929_43	TGGAAGTGTCAGAAATTCTGAGT	up	XLOC_009175	down
dre-miR-125b-5p_R+1	TCCCTGAGACCCTAACTTGTGAT	up	XLOC_009175	down
sha-miR-125a_R+2	TCCCTGAGACCCTAACTTGTGAAA	up	XLOC_009175	down
ssa-miR-125b-5p_R-1	TCCCTGAGACCCTTAACCTGTG	up	XLOC_009175	down
ssa-miR-7a-5p	TGGAAGACTAGTGATTTTGTTGT	up	XLOC_009196	down
ssa-miR-7a-5p_R+1	TGGAAGACTAGTGATTTTGTTGTT	up	XLOC_009196	down
ssa-mir-15c-2-p3_1ss11CA	TGCGAACCATAATTTGCTGCTT	down	XLOC_009196	down
dre-miR-125b-5p_R+1	TCCCTGAGACCCTAACTTGTGAT	up	XLOC_009229	up
ola-mir-100-2-p3	CAAGCTCGTATCTATAGGTATG	down	XLOC_009229	up
sha-miR-125a_R+2	TCCCTGAGACCCTAACTTGTGAAA	up	XLOC_009229	up
ssa-miR-125b-5p_R-1	TCCCTGAGACCCTTAACCTGTG	up	XLOC_009229	up
dre-miR-140-3p_L-1	ACCACAGGGTAGAACCACGGAC	up	XLOC_009259	up
dre-miR-1	TGGAATGTAAAGAAGTATGTAT	up	XLOC_009274	up
dre-miR-142a-5p	CATAAAGTAGAAAGCACTACT	down	XLOC_009274	up
ola-miR-146a-5p_1ss24TA	TGAGAACTGAATTCCATAGATGGAA	up	XLOC_009274	up
ola-miR-199a-3p_L+1	AACAGTAGTCTGCACATTGGTTA	up	XLOC_009274	up
ssa-miR-16b-5p_R-1_1ss21TC	TAGCAGCACGTAAATATTGGC	down	XLOC_009274	up
ssa-miR-199a-3p_R+2	ACAGTAGTCTGCACATTGGTTTT	up	XLOC_009274	up
ssa-miR-206-3p	TGGAATGTAAGGAAGTGTGTGG	up	XLOC_009274	up
ssc-miR-206	TGGAATGTAAGGAAGTGTGTGA	up	XLOC_009274	up
dre-miR-24_R+2_1	TGGCTCAGTTCAGCAGGAACAGAA	up	XLOC_009296	down
dre-miR-24_R+2_2	TGGCTCAGTTCAGCAGGAACAGTT	up	XLOC_009296	down
ssa-miR-199a-3p_R+2	ACAGTAGTCTGCACATTGGTTTT	up	XLOC_009296	down
PC-3p-50929_43	TGGAAGTGTCAGAAATTCTGAGT	up	XLOC_009307	up
PC-5p-27517_164	TACATGCAGAGGTGGAGCAAGA	up	XLOC_009307	up
ssa-miR-26d-5p_L+1_1ss13TC	CTTCAAGTAATCCAGGATAGGCT	up	XLOC_009307	up
dre-let-7d-5p	TGAGGTAGTTGGTTGTATGGTT	up	XLOC_009381	down
ola-miR-146a-5p_1ss24TA	TGAGAACTGAATTCCATAGATGGAA	up	XLOC_009381	down
tni-let-7j_1ss11TG	TGAGGTAGTTGTTTGTACAGTT	up	XLOC_009381	down
aca-miR-200b-3p_R+2	TAATACTGCCTGGTAATGATGAAT	up	XLOC_009391	up
dre-miR-140-3p_L-1	ACCACAGGGTAGAACCACGGAC	up	XLOC_009391	up
ssa-miR-16b-5p_R-1_1ss21TC	TAGCAGCACGTAAATATTGGC	down	XLOC_009391	up
ola-miR-146a-5p_1ss24TA	TGAGAACTGAATTCCATAGATGGAA	up	XLOC_009393	up
ssa-miR-730a-5p_R-1	TCCTCATTGTGCATGCTGTGT	down	XLOC_009408	up
dre-miR-1	TGGAATGTAAAGAAGTATGTAT	up	XLOC_009415	up
dre-miR-142a-5p	CATAAAGTAGAAAGCACTACT	down	XLOC_009415	up
dre-miR-194a_R+2	TGTAACAGCAACTCCATGTGGAT	up	XLOC_009415	up

ssa-miR-206-3p	TGGAATGTAAGGAAGTGTGTGG	up	XLOC_009415	up
ssc-miR-206	TGGAATGTAAGGAAGTGTGTGA	up	XLOC_009415	up
tni-miR-194_R+1	TGTAACAGCAACTCCATGTGGA	up	XLOC_009415	up
xtr-miR-122_L+1R-1	CTGGAGTGTGACAATGGTGTTTG	up	XLOC_009415	up
dre-miR-142a-3p_R-1	TGTAGTGTTTCCTACTTTATGG	down	XLOC_009418	down
ola-miR-199a-3p_L+1	AACAGTAGTCTGCACATTGGTTA	up	XLOC_009418	down
ssa-miR-199a-3p_R+2	ACAGTAGTCTGCACATTGGTTTT	up	XLOC_009418	down
PC-5p-27517_164	TACATGCAGAGGTGGAGCAAGA	up	XLOC_009429	down
dre-miR-125b-5p_R+1	TCCCTGAGACCCTAACTTGTGAT	up	XLOC_009429	down
dre-miR-194a_R+2	TGTAACAGCAACTCCATGTGGAT	up	XLOC_009429	down
ola-miR-194-3p_1ss20CT	CCAGTGGAGGTGCTGTTACTTG	up	XLOC_009429	down
sha-miR-125a_R+2	TCCCTGAGACCCTAACTTGTGAAA	up	XLOC_009429	down
ssa-miR-125b-5p_R-1	TCCCTGAGACCCTTAACCTGTG	up	XLOC_009429	down
tni-miR-194_R+1	TGTAACAGCAACTCCATGTGGA	up	XLOC_009429	down
PC-3p-41259_77	TGGCCATTAACTGCTAACCTTC	up	XLOC_009449	down
dre-miR-1	TGGAATGTAAAGAAGTATGTAT	up	XLOC_009449	down
ola-miR-194-3p_1ss20CT	CCAGTGGAGGTGCTGTTACTTG	up	XLOC_009449	down
ssa-miR-125b-5p_R-1	TCCCTGAGACCCTTAACCTGTG	up	XLOC_009449	down
ssa-miR-16b-5p_R-1_1ss21TC	TAGCAGCACGTAAATATTGGC	down	XLOC_009449	down
ssa-miR-206-3p	TGGAATGTAAGGAAGTGTGTGG	up	XLOC_009449	down
ssa-miR-7132b-3p	TGAGGCGTTTAGAACAAGTTCA	down	XLOC_009449	down
ssa-miR-730a-5p_R-1	TCCTCATTGTGCATGCTGTGT	down	XLOC_009449	down
ssc-miR-206	TGGAATGTAAGGAAGTGTGTGA	up	XLOC_009449	down
PC-3p-50929_43	TGGAAGTGTCAGAAATTCTGAGT	up	XLOC_009463	down
dre-miR-142a-3p_R-1	TGTAGTGTTTCCTACTTTATGG	down	XLOC_009463	down
ola-miR-146a-5p_1ss24TA	TGAGAACTGAATTCCATAGATGGAA	up	XLOC_009463	down
PC-5p-45063_62	AAGGATAACTACAACTGTACTT	up	XLOC_009492	down
ola-miR-199a-3p_L+1	AACAGTAGTCTGCACATTGGTTA	up	XLOC_009509	down
dre-miR-22a-3p	AAGCTGCCAGCTGAAGAACTGT	up	XLOC_009549	up
PC-3p-11630_419	ATGAGGAAAAGAAGTTAGGAGA	down	XLOC_009640	up
aca-miR-200b-3p_R+2	TAATACTGCCTGGTAATGATGAAT	up	XLOC_009640	up
ssa-miR-1338-5p_R+1	AGGACTGTCCAACCTGAGAATG	down	XLOC_009640	up
ssa-miR-7132a-5p_R+1	GACTTGGTCAAAGCTCCTCAGTT	down	XLOC_009640	up
ssa-miR-7132b-5p	GACTTGGTCAAAGCTCCTCAGC	down	XLOC_009640	up
ssa-miR-730a-5p_R-1	TCCTCATTGTGCATGCTGTGT	down	XLOC_009640	up
xtr-miR-122_L+1R-1	CTGGAGTGTGACAATGGTGTTTG	up	XLOC_009640	up
dre-miR-21_1ss23CA	TAGCTTATCAGACTGGTGTTGGA	up	XLOC_009657	up
ola-miR-194-3p_1ss20CT	CCAGTGGAGGTGCTGTTACTTG	up	XLOC_009657	up
PC-3p-11630_419	ATGAGGAAAAGAAGTTAGGAGA	down	XLOC_009669	down
dre-miR-142a-5p	CATAAAGTAGAAAGCACTACT	down	XLOC_009669	down
ssa-miR-16b-5p_R-1_1ss21TC	TAGCAGCACGTAAATATTGGC	down	XLOC_009669	down
ola-miR-194-3p_1ss20CT	CCAGTGGAGGTGCTGTTACTTG	up	XLOC_009743	down
ssa-miR-1338-5p_R+1	AGGACTGTCCAACCTGAGAATG	down	XLOC_009743	down
ssa-miR-16b-5p_R-1_1ss21TC	TAGCAGCACGTAAATATTGGC	down	XLOC_009743	down
aca-miR-200b-3p_R+2	TAATACTGCCTGGTAATGATGAAT	up	XLOC_009762	down
dre-miR-122	TGGAGTGTGACAATGGTGTTTG	up	XLOC_009780	down
dre-miR-133b-3p_R-1	TTTGGTCCCCTTCAACCAGCT	up	XLOC_009780	down
dre-miR-140-3p_L-1	ACCACAGGGTAGAACCACGGAC	up	XLOC_009780	down
dre-miR-142a-3p_R-1	TGTAGTGTTTCCTACTTTATGG	down	XLOC_009780	down
oha-miR-133b-3p	TTTGGTCCCCTTCAACCAGCTAT	up	XLOC_009780	down
xtr-miR-122_L+1R-1	CTGGAGTGTGACAATGGTGTTTG	up	XLOC_009780	down
ola-miR-194-3p_1ss20CT	CCAGTGGAGGTGCTGTTACTTG	up	XLOC_009805	up
rno-miR-122-5p_L+3	ATCTGGAGTGTGACAATGGTGTTTG	up	XLOC_009805	up
PC-5p-27517_164	TACATGCAGAGGTGGAGCAAGA	up	XLOC_009814	down

dre-miR-133b-3p_R-1	TTTGGTCCCCTTCAACCAGCT	up	XLOC_009814	down
oha-miR-133b-3p	TTTGGTCCCCTTCAACCAGCTAT	up	XLOC_009814	down
PC-3p-11630_419	ATGAGGAAAAGAAGTTAGGAGA	down	XLOC_009825	down
ola-miR-194-3p_1ss20CT	CCAGTGGAGGTGCTGTTACTTG	up	XLOC_009860	up
dre-miR-21_1ss23CA	TAGCTTATCAGACTGGTGTTGGA	up	XLOC_009879	down
dre-miR-22a-3p	AAGCTGCCAGCTGAAGAACTGT	up	XLOC_009879	down
dre-miR-24_R+2_1	TGGCTCAGTTCAGCAGGAACAGAA	up	XLOC_009879	down
dre-miR-24_R+2_2	TGGCTCAGTTCAGCAGGAACAGTT	up	XLOC_009879	down
ssa-miR-730a-5p_R-1	TCCTCATTGTGCATGCTGTGT	down	XLOC_009996	down
ssa-miR-730a-5p_R-1	TCCTCATTGTGCATGCTGTGT	down	XLOC_009999	down
PC-5p-27517_164	TACATGCAGAGGTGGAGCAAGA	up	XLOC_010014	down
dre-miR-133b-3p_R-1	TTTGGTCCCCTTCAACCAGCT	up	XLOC_010014	down
oha-miR-133b-3p	TTTGGTCCCCTTCAACCAGCTAT	up	XLOC_010014	down
PC-3p-11630_419	ATGAGGAAAAGAAGTTAGGAGA	down	XLOC_010068	up
xtr-miR-122_L+1R-1	CTGGAGTGTGACAATGGTGTTTG	up	XLOC_010131	down
PC-3p-41259_77	TGGCCATTAACTGCTAACCTTC	up	XLOC_010137	up
aca-miR-200b-3p_R+2	TAATACTGCCTGGTAATGATGAAT	up	XLOC_010137	up
dre-miR-1	TGGAATGTAAAGAAGTATGTAT	up	XLOC_010137	up
ssa-miR-206-3p	TGGAATGTAAGGAAGTGTGTGG	up	XLOC_010137	up
ssc-miR-206	TGGAATGTAAGGAAGTGTGTGA	up	XLOC_010137	up
dre-miR-142a-3p_R-1	TGTAGTGTTTCCTACTTTATGG	down	XLOC_010140	down
dre-miR-1	TGGAATGTAAAGAAGTATGTAT	up	XLOC_010148	down
dre-miR-194a_R+2	TGTAACAGCAACTCCATGTGGAT	up	XLOC_010148	down
ssa-miR-206-3p	TGGAATGTAAGGAAGTGTGTGG	up	XLOC_010148	down
ssc-miR-206	TGGAATGTAAGGAAGTGTGTGA	up	XLOC_010148	down
tni-miR-194_R+1	TGTAACAGCAACTCCATGTGGA	up	XLOC_010148	down
ssa-miR-16b-5p_R-1_1ss21TC	TAGCAGCACGTAAATATTGGC	down	XLOC_010155	up
ssa-miR-206-3p	TGGAATGTAAGGAAGTGTGTGG	up	XLOC_010155	up
ssc-miR-206	TGGAATGTAAGGAAGTGTGTGA	up	XLOC_010155	up
dre-miR-1	TGGAATGTAAAGAAGTATGTAT	up	XLOC_010187	down
dre-miR-22a-3p	AAGCTGCCAGCTGAAGAACTGT	up	XLOC_010187	down
ola-miR-194-3p_1ss20CT	CCAGTGGAGGTGCTGTTACTTG	up	XLOC_010187	down
ssa-miR-206-3p	TGGAATGTAAGGAAGTGTGTGG	up	XLOC_010187	down
ssc-miR-206	TGGAATGTAAGGAAGTGTGTGA	up	XLOC_010187	down
PC-3p-41259_77	TGGCCATTAACTGCTAACCTTC	up	XLOC_010202	down
PC-3p-50929_43	TGGAAGTGTCAGAAATTCTGAGT	up	XLOC_010202	down
aca-miR-200b-3p_R+2	TAATACTGCCTGGTAATGATGAAT	up	XLOC_010202	down
dre-miR-140-3p_L-1	ACCACAGGGTAGAACCACGGAC	up	XLOC_010202	down
ola-mir-100-2-p3	CAAGCTCGTATCTATAGGTATG	down	XLOC_010202	down
ssa-miR-730a-5p_R-1	TCCTCATTGTGCATGCTGTGT	down	XLOC_010202	down
aca-miR-200b-3p_R+2	TAATACTGCCTGGTAATGATGAAT	up	XLOC_010218	down
xtr-miR-122_L+1R-1	CTGGAGTGTGACAATGGTGTTTG	up	XLOC_010218	down
ssa-miR-16b-5p_R-1_1ss21TC	TAGCAGCACGTAAATATTGGC	down	XLOC_010241	up
PC-5p-27517_164	TACATGCAGAGGTGGAGCAAGA	up	XLOC_010248	down
dre-let-7d-5p	TGAGGTAGTTGGTTGTATGGTT	up	XLOC_010248	down
mmu-let-7j_1ss8TG	TGAGGTAGTAGTTTGTGCTGTTAT	up	XLOC_010248	down
tni-let-7j_1ss11TG	TGAGGTAGTTGTTTGTACAGTT	up	XLOC_010248	down
PC-3p-41259_77	TGGCCATTAACTGCTAACCTTC	up	XLOC_010281	up
PC-5p-8690_526	GATGTTGAGTATCAAACTGTAT	down	XLOC_010284	down
ola-miR-194-3p_1ss20CT	CCAGTGGAGGTGCTGTTACTTG	up	XLOC_010284	down
ssa-miR-26d-5p_L+1_1ss13TC	CTTCAAGTAATCCAGGATAGGCT	up	XLOC_010284	down
ssa-miR-730a-5p_R-1	TCCTCATTGTGCATGCTGTGT	down	XLOC_010284	down
ssa-miR-26d-5p_L+1_1ss13TC	CTTCAAGTAATCCAGGATAGGCT	up	XLOC_010309	up
dre-miR-125b-5p_R+1	TCCCTGAGACCCTAACTTGTGAT	up	XLOC_010315	down

sha-miR-125a_R+2	TCCCTGAGACCCTAACTTGTGAAA	up	XLOC_010315	down
ssa-miR-125b-5p_R-1	TCCCTGAGACCCTTAACCTGTG	up	XLOC_010315	down
dre-miR-122	TGGAGTGTGACAATGGTGTTTG	up	XLOC_010321	down
PC-5p-27517_164	TACATGCAGAGGTGGAGCAAGA	up	XLOC_010323	down
dre-miR-125b-5p_R+1	TCCCTGAGACCCTAACTTGTGAT	up	XLOC_010323	down
sha-miR-125a_R+2	TCCCTGAGACCCTAACTTGTGAAA	up	XLOC_010323	down
ssa-miR-125b-5p_R-1	TCCCTGAGACCCTTAACCTGTG	up	XLOC_010323	down
ssa-miR-730a-5p_R-1	TCCTCATTGTGCATGCTGTGT	down	XLOC_010323	down
dre-miR-1	TGGAATGTAAAGAAGTATGTAT	up	XLOC_010354	up
ssa-miR-1338-5p_R+1	AGGACTGTCCAACCTGAGAATG	down	XLOC_010354	up
ssa-miR-206-3p	TGGAATGTAAGGAAGTGTGTGG	up	XLOC_010354	up
ssc-miR-206	TGGAATGTAAGGAAGTGTGTGA	up	XLOC_010354	up
ola-miR-194-3p_1ss20CT	CCAGTGGAGGTGCTGTTACTTG	up	XLOC_010407	down
ssa-miR-16b-5p_R-1_1ss21TC	TAGCAGCACGTAAATATTGGC	down	XLOC_010407	down
PC-5p-8690_526	GATGTTGAGTATCAAACTGTAT	down	XLOC_010429	down
PC-5p-8690_526	GATGTTGAGTATCAAACTGTAT	down	XLOC_010449	down
dre-miR-142a-3p_R-1	TGTAGTGTTTCCTACTTTATGG	down	XLOC_010449	down
dre-miR-194a_R+2	TGTAACAGCAACTCCATGTGGAT	up	XLOC_010449	down
ola-miR-199a-3p_L+1	AACAGTAGTCTGCACATTGGTTA	up	XLOC_010449	down
ssa-miR-730a-5p_R-1	TCCTCATTGTGCATGCTGTGT	down	XLOC_010449	down
tni-miR-194_R+1	TGTAACAGCAACTCCATGTGGA	up	XLOC_010449	down
PC-3p-41259_77	TGGCCATTAACTGCTAACCTTC	up	XLOC_010475	down
PC-3p-50929_43	TGGAAGTGTCAGAAATTCTGAGT	up	XLOC_010475	down
dre-miR-133b-3p_R-1	TTTGGTCCCCTTCAACCAGCT	up	XLOC_010475	down
dre-miR-24_R+2_1	TGGCTCAGTTCAGCAGGAACAGAA	up	XLOC_010475	down
dre-miR-24_R+2_2	TGGCTCAGTTCAGCAGGAACAGTT	up	XLOC_010475	down
oha-miR-133b-3p	TTTGGTCCCCTTCAACCAGCTAT	up	XLOC_010475	down
ola-miR-199a-3p_L+1	AACAGTAGTCTGCACATTGGTTA	up	XLOC_010475	down
ssa-miR-199a-3p_R+2	ACAGTAGTCTGCACATTGGTTTT	up	XLOC_010475	down
ssa-miR-26d-5p_L+1_1ss13TC	CTTCAAGTAATCCAGGATAGGCT	up	XLOC_010475	down
ssa-miR-7132a-5p_R+1	GACTTGGTCAAAGCTCCTCAGTT	down	XLOC_010475	down
ssa-miR-7132b-5p	GACTTGGTCAAAGCTCCTCAGC	down	XLOC_010475	down
PC-5p-27517_164	TACATGCAGAGGTGGAGCAAGA	up	XLOC_010501	down
aca-miR-200b-3p_R+2	TAATACTGCCTGGTAATGATGAAT	up	XLOC_010501	down
dre-miR-122	TGGAGTGTGACAATGGTGTTTG	up	XLOC_010501	down
dre-miR-140-3p_L-1	ACCACAGGGTAGAACCACGGAC	up	XLOC_010501	down
dre-miR-142a-5p	CATAAAGTAGAAAGCACTACT	down	XLOC_010501	down
xtr-miR-122_L+1R-1	CTGGAGTGTGACAATGGTGTTTG	up	XLOC_010501	down
dre-miR-24_R+2_1	TGGCTCAGTTCAGCAGGAACAGAA	up	XLOC_010523	up
dre-miR-24_R+2_2	TGGCTCAGTTCAGCAGGAACAGTT	up	XLOC_010523	up
ssa-miR-26a-4-3p	CCTATTCTTGATTACTTGTTTC	down	XLOC_010523	up
dre-miR-140-3p_L-1	ACCACAGGGTAGAACCACGGAC	up	XLOC_010541	up
dre-miR-22a-3p	AAGCTGCCAGCTGAAGAACTGT	up	XLOC_010541	up
ssa-miR-206-3p	TGGAATGTAAGGAAGTGTGTGG	up	XLOC_010541	up
ssc-miR-206	TGGAATGTAAGGAAGTGTGTGA	up	XLOC_010541	up
ssa-miR-1338-5p_R+1	AGGACTGTCCAACCTGAGAATG	down	XLOC_010559	down
PC-5p-27517_164	TACATGCAGAGGTGGAGCAAGA	up	XLOC_010561	up
dre-let-7d-5p	TGAGGTAGTTGGTTGTATGGTT	up	XLOC_010561	up
ssa-miR-16b-5p_R-1_1ss21TC	TAGCAGCACGTAAATATTGGC	down	XLOC_010561	up
ssa-miR-26a-4-3p	CCTATTCTTGATTACTTGTTTC	down	XLOC_010561	up
tni-let-7j_1ss11TG	TGAGGTAGTTGTTTGTACAGTT	up	XLOC_010561	up
PC-3p-41259_77	TGGCCATTAACTGCTAACCTTC	up	XLOC_010583	up
dre-miR-142a-3p_R-1	TGTAGTGTTTCCTACTTTATGG	down	XLOC_010583	up
ssa-miR-16b-5p_R-1_1ss21TC	TAGCAGCACGTAAATATTGGC	down	XLOC_010583	up

aca-miR-338-3p_R+2	TCCAGCATCAGTGATTTTGTTAA	up	XLOC_010621	down
ssa-miR-16b-5p_R-1_1ss21TC	TAGCAGCACGTAAATATTGGC	down	XLOC_010621	down
rno-miR-122-5p_L+3	ATCTGGAGTGTGACAATGGTGTTTG	up	XLOC_010628	up
dre-let-7d-5p	TGAGGTAGTTGGTTGTATGGTT	up	XLOC_010643	down
dre-miR-142a-3p_R-1	TGTAGTGTTTCCTACTTTATGG	down	XLOC_010643	down
mmu-let-7j_1ss8TG	TGAGGTAGTAGTTTGTGCTGTTAT	up	XLOC_010643	down
tni-let-7j_1ss11TG	TGAGGTAGTTGTTTGTACAGTT	up	XLOC_010643	down
dre-miR-21_1ss23CA	TAGCTTATCAGACTGGTGTTGGA	up	XLOC_010694	down
aca-miR-338-3p_R+2	TCCAGCATCAGTGATTTTGTTAA	up	XLOC_010710	down
dre-miR-125b-5p_R+1	TCCCTGAGACCCTAACTTGTGAT	up	XLOC_010710	down
dre-miR-194a_R+2	TGTAACAGCAACTCCATGTGGAT	up	XLOC_010710	down
sha-miR-125a_R+2	TCCCTGAGACCCTAACTTGTGAAA	up	XLOC_010710	down
ssa-miR-125b-5p_R-1	TCCCTGAGACCCTTAACCTGTG	up	XLOC_010710	down
ssa-miR-16b-5p_R-1_1ss21TC	TAGCAGCACGTAAATATTGGC	down	XLOC_010710	down
ssa-miR-730a-5p_R-1	TCCTCATTGTGCATGCTGTGT	down	XLOC_010710	down
tni-miR-194_R+1	TGTAACAGCAACTCCATGTGGA	up	XLOC_010710	down
dre-let-7d-5p	TGAGGTAGTTGGTTGTATGGTT	up	XLOC_010717	up
dre-miR-122	TGGAGTGTGACAATGGTGTTTG	up	XLOC_010717	up
PC-3p-50929_43	TGGAAGTGTCAGAAATTCTGAGT	up	XLOC_010718	up
PC-5p-27517_164	TACATGCAGAGGTGGAGCAAGA	up	XLOC_010718	up
dre-miR-142a-5p	CATAAAGTAGAAAGCACTACT	down	XLOC_010718	up
dre-miR-22a-3p	AAGCTGCCAGCTGAAGAACTGT	up	XLOC_010718	up
PC-3p-50929_43	TGGAAGTGTCAGAAATTCTGAGT	up	XLOC_010807	up
dre-miR-24_R+2_1	TGGCTCAGTTCAGCAGGAACAGAA	up	XLOC_010807	up
dre-miR-24_R+2_2	TGGCTCAGTTCAGCAGGAACAGTT	up	XLOC_010807	up
dre-miR-122	TGGAGTGTGACAATGGTGTTTG	up	XLOC_010820	down
dre-miR-133a-3p_L-1R+1	TTGGTCCCCTTCAACCAGCTGT	up	XLOC_010820	down
ola-miR-194-3p_1ss20CT	CCAGTGGAGGTGCTGTTACTTG	up	XLOC_010820	down
ssa-miR-7a-5p	TGGAAGACTAGTGATTTTGTTGT	up	XLOC_010820	down
ssa-miR-7a-5p_R+1	TGGAAGACTAGTGATTTTGTTGTT	up	XLOC_010820	down
xtr-miR-122_L+1R-1	CTGGAGTGTGACAATGGTGTTTG	up	XLOC_010820	down
PC-3p-11630_419	ATGAGGAAAAGAAGTTAGGAGA	down	XLOC_010881	up
PC-5p-27517_164	TACATGCAGAGGTGGAGCAAGA	up	XLOC_010881	up
dre-miR-194a_R+2	TGTAACAGCAACTCCATGTGGAT	up	XLOC_010881	up
dre-miR-21_1ss23CA	TAGCTTATCAGACTGGTGTTGGA	up	XLOC_010881	up
dre-miR-24_R+2_1	TGGCTCAGTTCAGCAGGAACAGAA	up	XLOC_010881	up
dre-miR-24_R+2_2	TGGCTCAGTTCAGCAGGAACAGTT	up	XLOC_010881	up
tni-miR-194_R+1	TGTAACAGCAACTCCATGTGGA	up	XLOC_010881	up
dre-miR-142a-5p	CATAAAGTAGAAAGCACTACT	down	XLOC_010890	down
ssa-miR-16b-5p_R-1_1ss21TC	TAGCAGCACGTAAATATTGGC	down	XLOC_010890	down
dre-miR-21_1ss23CA	TAGCTTATCAGACTGGTGTTGGA	up	XLOC_010936	up
ssa-miR-206-3p	TGGAATGTAAGGAAGTGTGTGG	up	XLOC_010936	up
ssc-miR-206	TGGAATGTAAGGAAGTGTGTGA	up	XLOC_010936	up
ssa-miR-730a-5p_R-1	TCCTCATTGTGCATGCTGTGT	down	XLOC_010940	up
dre-miR-22a-3p	AAGCTGCCAGCTGAAGAACTGT	up	XLOC_010972	up
ola-miR-199a-3p_L+1	AACAGTAGTCTGCACATTGGTTA	up	XLOC_010979	up
ssa-miR-1338-5p_R+1	AGGACTGTCCAACCTGAGAATG	down	XLOC_010979	up
ssa-miR-26a-4-3p	CCTATTCTTGATTACTTGTTTC	down	XLOC_010979	up
PC-3p-11630_419	ATGAGGAAAAGAAGTTAGGAGA	down	XLOC_011010	down
ola-miR-146a-5p_1ss24TA	TGAGAACTGAATTCCATAGATGGAA	up	XLOC_011010	down
ssa-miR-7a-5p	TGGAAGACTAGTGATTTTGTTGT	up	XLOC_011010	down
ssa-miR-7a-5p_R+1	TGGAAGACTAGTGATTTTGTTGTT	up	XLOC_011010	down
ola-miR-199a-3p_L+1	AACAGTAGTCTGCACATTGGTTA	up	XLOC_011068	up
ssa-miR-199a-3p_R+2	ACAGTAGTCTGCACATTGGTTTT	up	XLOC_011068	up

ssa-miR-7132b-3p	TGAGGCGTTTAGAACAAGTTCA	down	XLOC_011068	up
aca-miR-200b-3p_R+2	TAATACTGCCTGGTAATGATGAAT	up	XLOC_011088	down
dre-miR-21_1ss23CA	TAGCTTATCAGACTGGTGTTGGA	up	XLOC_011134	up
dre-miR-22a-3p	AAGCTGCCAGCTGAAGAACTGT	up	XLOC_011235	down
ssa-miR-199a-3p_R+2	ACAGTAGTCTGCACATTGGTTTT	up	XLOC_011242	down
ssa-miR-7132b-3p	TGAGGCGTTTAGAACAAGTTCA	down	XLOC_011242	down
PC-5p-8690_526	GATGTTGAGTATCAAACTGTAT	down	XLOC_011350	up
dre-miR-194a_R+2	TGTAACAGCAACTCCATGTGGAT	up	XLOC_011350	up
ssa-miR-16b-5p_R-1_1ss21TC	TAGCAGCACGTAAATATTGGC	down	XLOC_011350	up
ssa-miR-199a-3p_R+2	ACAGTAGTCTGCACATTGGTTTT	up	XLOC_011350	up
tni-miR-194_R+1	TGTAACAGCAACTCCATGTGGA	up	XLOC_011350	up
dre-miR-122	TGGAGTGTGACAATGGTGTTTG	up	XLOC_011392	up
ola-miR-194-3p_1ss20CT	CCAGTGGAGGTGCTGTTACTTG	up	XLOC_011392	up
dre-miR-122	TGGAGTGTGACAATGGTGTTTG	up	XLOC_011438	up
dre-miR-194a_R+2	TGTAACAGCAACTCCATGTGGAT	up	XLOC_011438	up
rno-miR-122-5p_L+3	ATCTGGAGTGTGACAATGGTGTTTG	up	XLOC_011438	up
ssa-miR-26a-4-3p	CCTATTCTTGATTACTTGTTTC	down	XLOC_011438	up
tni-miR-194_R+1	TGTAACAGCAACTCCATGTGGA	up	XLOC_011438	up
xtr-miR-122_L+1R-1	CTGGAGTGTGACAATGGTGTTTG	up	XLOC_011438	up
PC-3p-11630_419	ATGAGGAAAAGAAGTTAGGAGA	down	XLOC_011454	up
ola-mir-100-2-p3	CAAGCTCGTATCTATAGGTATG	down	XLOC_011454	up
ssa-miR-7a-5p	TGGAAGACTAGTGATTTTGTTGT	up	XLOC_011454	up
ssa-miR-7a-5p_R+1	TGGAAGACTAGTGATTTTGTTGTT	up	XLOC_011454	up
PC-5p-27517_164	TACATGCAGAGGTGGAGCAAGA	up	XLOC_011516	up
PC-5p-8690_526	GATGTTGAGTATCAAACTGTAT	down	XLOC_011516	up
ssa-miR-7a-5p	TGGAAGACTAGTGATTTTGTTGT	up	XLOC_011516	up
ssa-miR-7a-5p_R+1	TGGAAGACTAGTGATTTTGTTGTT	up	XLOC_011516	up
PC-3p-11630_419	ATGAGGAAAAGAAGTTAGGAGA	down	XLOC_011569	down
tni-miR-10c	TACCCTGTAGATCCGGATTTGT	up	XLOC_011569	down
PC-3p-41259_77	TGGCCATTAACTGCTAACCTTC	up	XLOC_011576	up
ssa-miR-16b-5p_R-1_1ss21TC	TAGCAGCACGTAAATATTGGC	down	XLOC_011576	up
dre-miR-21_1ss23CA	TAGCTTATCAGACTGGTGTTGGA	up	XLOC_011588	down
PC-5p-27517_164	TACATGCAGAGGTGGAGCAAGA	up	XLOC_011644	down
ssa-miR-199a-3p_R+2	ACAGTAGTCTGCACATTGGTTTT	up	XLOC_011665	up
ssa-miR-26d-5p_L+1_1ss13TC	CTTCAAGTAATCCAGGATAGGCT	up	XLOC_011665	up
dre-miR-21_1ss23CA	TAGCTTATCAGACTGGTGTTGGA	up	XLOC_011666	up
dre-miR-24_R+2_1	TGGCTCAGTTCAGCAGGAACAGAA	up	XLOC_011666	up
dre-miR-24_R+2_2	TGGCTCAGTTCAGCAGGAACAGTT	up	XLOC_011666	up
aca-miR-200b-3p_R+2	TAATACTGCCTGGTAATGATGAAT	up	XLOC_011672	down
dre-miR-140-3p_L-1	ACCACAGGGTAGAACCACGGAC	up	XLOC_011672	down
PC-3p-50929_43	TGGAAGTGTCAGAAATTCTGAGT	up	XLOC_011688	down
aca-miR-200b-3p_R+2	TAATACTGCCTGGTAATGATGAAT	up	XLOC_011749	up
ssa-miR-7132b-3p	TGAGGCGTTTAGAACAAGTTCA	down	XLOC_011749	up
ssa-miR-730a-5p_R-1	TCCTCATTGTGCATGCTGTGT	down	XLOC_011769	down
PC-3p-11630_419	ATGAGGAAAAGAAGTTAGGAGA	down	XLOC_011774	down
PC-5p-27517_164	TACATGCAGAGGTGGAGCAAGA	up	XLOC_011774	down
aca-miR-200b-3p_R+2	TAATACTGCCTGGTAATGATGAAT	up	XLOC_011796	down
ssa-miR-7132b-3p	TGAGGCGTTTAGAACAAGTTCA	down	XLOC_011796	down
PC-3p-11630_419	ATGAGGAAAAGAAGTTAGGAGA	down	XLOC_011839	down
rno-miR-122-5p_L+3	ATCTGGAGTGTGACAATGGTGTTTG	up	XLOC_011839	down
aca-miR-200b-3p_R+2	TAATACTGCCTGGTAATGATGAAT	up	XLOC_011840	down
dre-miR-21_1ss23CA	TAGCTTATCAGACTGGTGTTGGA	up	XLOC_011840	down
ola-miR-199a-3p_L+1	AACAGTAGTCTGCACATTGGTTA	up	XLOC_011840	down
ssa-miR-199a-3p_R+2	ACAGTAGTCTGCACATTGGTTTT	up	XLOC_011840	down

PC-5p-27517_164	TACATGCAGAGGTGGAGCAAGA	up	XLOC_011853	down
aca-miR-200b-3p_R+2	TAATACTGCCTGGTAATGATGAAT	up	XLOC_011853	down
dre-miR-22a-3p	AAGCTGCCAGCTGAAGAACTGT	up	XLOC_011853	down
ssa-miR-16b-5p_R-1_1ss21TC	TAGCAGCACGTAAATATTGGC	down	XLOC_011853	down
ssa-miR-26a-4-3p	CCTATTCTTGATTACTTGTTTC	down	XLOC_011862	down
ssa-miR-7a-5p	TGGAAGACTAGTGATTTTGTTGT	up	XLOC_011890	up
ssa-miR-7a-5p_R+1	TGGAAGACTAGTGATTTTGTTGTT	up	XLOC_011890	up
dre-miR-1	TGGAATGTAAAGAAGTATGTAT	up	XLOC_011901	down
dre-miR-140-3p_L-1	ACCACAGGGTAGAACCACGGAC	up	XLOC_011901	down
ssa-miR-1-4-5p	ACATACTTCTTTATATGCCCATA	up	XLOC_011901	down
ssa-miR-206-3p	TGGAATGTAAGGAAGTGTGTGG	up	XLOC_011901	down
ssc-miR-206	TGGAATGTAAGGAAGTGTGTGA	up	XLOC_011901	down
tni-miR-10c	TACCCTGTAGATCCGGATTTGT	up	XLOC_011901	down
dre-miR-125b-5p_R+1	TCCCTGAGACCCTAACTTGTGAT	up	XLOC_011910	up
sha-miR-125a_R+2	TCCCTGAGACCCTAACTTGTGAAA	up	XLOC_011910	up
ssa-miR-125b-5p_R-1	TCCCTGAGACCCTTAACCTGTG	up	XLOC_011910	up
ssa-miR-730a-5p_R-1	TCCTCATTGTGCATGCTGTGT	down	XLOC_011910	up
PC-3p-11630_419	ATGAGGAAAAGAAGTTAGGAGA	down	XLOC_011922	down
aca-miR-200b-3p_R+2	TAATACTGCCTGGTAATGATGAAT	up	XLOC_011922	down
dre-miR-1	TGGAATGTAAAGAAGTATGTAT	up	XLOC_011962	down
ssa-miR-206-3p	TGGAATGTAAGGAAGTGTGTGG	up	XLOC_011962	down
ssc-miR-206	TGGAATGTAAGGAAGTGTGTGA	up	XLOC_011962	down
PC-3p-50929_43	TGGAAGTGTCAGAAATTCTGAGT	up	XLOC_011993	down
PC-5p-8690_526	GATGTTGAGTATCAAACTGTAT	down	XLOC_011993	down
dre-miR-194a_R+2	TGTAACAGCAACTCCATGTGGAT	up	XLOC_011993	down
ssa-miR-730a-5p_R-1	TCCTCATTGTGCATGCTGTGT	down	XLOC_011993	down
tni-miR-194_R+1	TGTAACAGCAACTCCATGTGGA	up	XLOC_011993	down
ola-miR-194-3p_1ss20CT	CCAGTGGAGGTGCTGTTACTTG	up	XLOC_012011	up
ssa-miR-7132b-3p	TGAGGCGTTTAGAACAAGTTCA	down	XLOC_012011	up
ssa-miR-1-4-5p	ACATACTTCTTTATATGCCCATA	up	XLOC_012016	up
dre-miR-142a-3p_R-1	TGTAGTGTTTCCTACTTTATGG	down	XLOC_012115	down
PC-3p-41259_77	TGGCCATTAACTGCTAACCTTC	up	XLOC_012117	down
aca-miR-338-3p_R+2	TCCAGCATCAGTGATTTTGTTAA	up	XLOC_012117	down
ola-miR-146a-5p_1ss24TA	TGAGAACTGAATTCCATAGATGGAA	up	XLOC_012117	down
ssa-miR-730a-5p_R-1	TCCTCATTGTGCATGCTGTGT	down	XLOC_012117	down
ssa-miR-1338-5p_R+1	AGGACTGTCCAACCTGAGAATG	down	XLOC_012125	up
PC-5p-27517_164	TACATGCAGAGGTGGAGCAAGA	up	XLOC_012130	down
dre-miR-194a_R+2	TGTAACAGCAACTCCATGTGGAT	up	XLOC_012130	down
tni-miR-194_R+1	TGTAACAGCAACTCCATGTGGA	up	XLOC_012130	down
PC-3p-41259_77	TGGCCATTAACTGCTAACCTTC	up	XLOC_012134	up
PC-5p-27517_164	TACATGCAGAGGTGGAGCAAGA	up	XLOC_012134	up
PC-5p-45063_62	AAGGATAACTACAACTGTACTT	up	XLOC_012134	up
aca-miR-338-3p_R+2	TCCAGCATCAGTGATTTTGTTAA	up	XLOC_012134	up
dre-miR-194a_R+2	TGTAACAGCAACTCCATGTGGAT	up	XLOC_012134	up
dre-miR-22a-3p	AAGCTGCCAGCTGAAGAACTGT	up	XLOC_012134	up
dre-miR-24_R+2_1	TGGCTCAGTTCAGCAGGAACAGAA	up	XLOC_012134	up
dre-miR-24_R+2_2	TGGCTCAGTTCAGCAGGAACAGTT	up	XLOC_012134	up
ssa-miR-7a-5p	TGGAAGACTAGTGATTTTGTTGT	up	XLOC_012134	up
ssa-miR-7a-5p_R+1	TGGAAGACTAGTGATTTTGTTGTT	up	XLOC_012134	up
tni-miR-194_R+1	TGTAACAGCAACTCCATGTGGA	up	XLOC_012134	up
xtr-miR-122_L+1R-1	CTGGAGTGTGACAATGGTGTTTG	up	XLOC_012134	up
dre-miR-24_R+2_1	TGGCTCAGTTCAGCAGGAACAGAA	up	XLOC_012136	down
dre-miR-24_R+2_2	TGGCTCAGTTCAGCAGGAACAGTT	up	XLOC_012136	down
PC-5p-27517_164	TACATGCAGAGGTGGAGCAAGA	up	XLOC_012201	down

dre-miR-142a-3p_R-1	TGTAGTGTTTCCTACTTTATGG	down	XLOC_012214	up
rno-miR-122-5p_L+3	ATCTGGAGTGTGACAATGGTGTTTG	up	XLOC_012214	up
ssa-miR-7132a-5p_R+1	GACTTGGTCAAAGCTCCTCAGTT	down	XLOC_012214	up
ssa-miR-7132b-5p	GACTTGGTCAAAGCTCCTCAGC	down	XLOC_012214	up
xtr-miR-122_L+1R-1	CTGGAGTGTGACAATGGTGTTTG	up	XLOC_012214	up
PC-5p-8690_526	GATGTTGAGTATCAAACTGTAT	down	XLOC_012232	down
rno-miR-122-5p_L+3	ATCTGGAGTGTGACAATGGTGTTTG	up	XLOC_012232	down
ssa-miR-7a-5p	TGGAAGACTAGTGATTTTGTTGT	up	XLOC_012232	down
ssa-miR-7a-5p_R+1	TGGAAGACTAGTGATTTTGTTGTT	up	XLOC_012232	down
PC-5p-8690_526	GATGTTGAGTATCAAACTGTAT	down	XLOC_012278	down
dre-miR-142a-3p_R-1	TGTAGTGTTTCCTACTTTATGG	down	XLOC_012278	down
ssa-miR-16b-5p_R-1_1ss21TC	TAGCAGCACGTAAATATTGGC	down	XLOC_012278	down
PC-5p-27517_164	TACATGCAGAGGTGGAGCAAGA	up	XLOC_012279	up
ola-miR-194-3p_1ss20CT	CCAGTGGAGGTGCTGTTACTTG	up	XLOC_012279	up
ola-miR-199a-3p_L+1	AACAGTAGTCTGCACATTGGTTA	up	XLOC_012279	up
ssa-miR-26d-5p_L+1_1ss13TC	CTTCAAGTAATCCAGGATAGGCT	up	XLOC_012279	up
PC-3p-50929_43	TGGAAGTGTCAGAAATTCTGAGT	up	XLOC_012298	down
dre-miR-1	TGGAATGTAAAGAAGTATGTAT	up	XLOC_012298	down
dre-miR-140-3p_L-1	ACCACAGGGTAGAACCACGGAC	up	XLOC_012298	down
ola-miR-199a-3p_L+1	AACAGTAGTCTGCACATTGGTTA	up	XLOC_012298	down
ssa-miR-206-3p	TGGAATGTAAGGAAGTGTGTGG	up	XLOC_012298	down
ssc-miR-206	TGGAATGTAAGGAAGTGTGTGA	up	XLOC_012298	down
dre-miR-122	TGGAGTGTGACAATGGTGTTTG	up	XLOC_012317	down
dre-miR-142a-3p_R-1	TGTAGTGTTTCCTACTTTATGG	down	XLOC_012317	down
ola-miR-462_L-1R+4	TAACGGAACCCATAATGCAGCT	down	XLOC_012317	down
tni-miR-10c	TACCCTGTAGATCCGGATTTGT	up	XLOC_012317	down
PC-3p-11630_419	ATGAGGAAAAGAAGTTAGGAGA	down	XLOC_012371	up
ssa-miR-7132b-3p	TGAGGCGTTTAGAACAAGTTCA	down	XLOC_012371	up
ssa-miR-7a-5p	TGGAAGACTAGTGATTTTGTTGT	up	XLOC_012371	up
ssa-miR-7a-5p_R+1	TGGAAGACTAGTGATTTTGTTGTT	up	XLOC_012371	up
dre-miR-24_R+2_1	TGGCTCAGTTCAGCAGGAACAGAA	up	XLOC_012379	down
dre-miR-24_R+2_2	TGGCTCAGTTCAGCAGGAACAGTT	up	XLOC_012379	down
ssa-miR-730a-5p_R-1	TCCTCATTGTGCATGCTGTGT	down	XLOC_012379	down
PC-3p-11630_419	ATGAGGAAAAGAAGTTAGGAGA	down	XLOC_012380	down
dre-miR-140-3p_L-1	ACCACAGGGTAGAACCACGGAC	up	XLOC_012380	down
dre-miR-194a_R+2	TGTAACAGCAACTCCATGTGGAT	up	XLOC_012380	down
ola-miR-194-3p_1ss20CT	CCAGTGGAGGTGCTGTTACTTG	up	XLOC_012380	down
tni-miR-194_R+1	TGTAACAGCAACTCCATGTGGA	up	XLOC_012380	down
PC-5p-27517_164	TACATGCAGAGGTGGAGCAAGA	up	XLOC_012393	down
PC-5p-45063_62	AAGGATAACTACAACTGTACTT	up	XLOC_012393	down
mmu-let-7j_1ss8TG	TGAGGTAGTAGTTTGTGCTGTTAT	up	XLOC_012393	down
ola-miR-146a-5p_1ss24TA	TGAGAACTGAATTCCATAGATGGAA	up	XLOC_012393	down
ssa-miR-16b-5p_R-1_1ss21TC	TAGCAGCACGTAAATATTGGC	down	XLOC_012393	down
ssa-miR-26d-5p_L+1_1ss13TC	CTTCAAGTAATCCAGGATAGGCT	up	XLOC_012400	up
dre-miR-22a-3p	AAGCTGCCAGCTGAAGAACTGT	up	XLOC_012456	up
ssa-miR-1-4-5p	ACATACTTCTTTATATGCCCATA	up	XLOC_012473	up
aca-miR-200b-3p_R+2	TAATACTGCCTGGTAATGATGAAT	up	XLOC_012510	up
ssa-miR-730a-5p_R-1	TCCTCATTGTGCATGCTGTGT	down	XLOC_012510	up
tni-miR-10c	TACCCTGTAGATCCGGATTTGT	up	XLOC_012510	up
ssa-miR-199a-3p_R+2	ACAGTAGTCTGCACATTGGTTTT	up	XLOC_012605	up
dre-miR-122	TGGAGTGTGACAATGGTGTTTG	up	XLOC_012632	down
ssa-miR-199a-3p_R+2	ACAGTAGTCTGCACATTGGTTTT	up	XLOC_012632	down
dre-miR-122	TGGAGTGTGACAATGGTGTTTG	up	XLOC_012654	up
PC-5p-27517_164	TACATGCAGAGGTGGAGCAAGA	up	XLOC_012683	down

ola-mir-100-2-p3	CAAGCTCGTATCTATAGGTATG	down	XLOC_012683	down
ssa-miR-730a-5p_R-1	TCCTCATTGTGCATGCTGTGT	down	XLOC_012683	down
PC-3p-11630_419	ATGAGGAAAAGAAGTTAGGAGA	down	XLOC_012689	up
PC-3p-41259_77	TGGCCATTAACTGCTAACCTTC	up	XLOC_012689	up
PC-5p-27517_164	TACATGCAGAGGTGGAGCAAGA	up	XLOC_012689	up
dre-miR-21_1ss23CA	TAGCTTATCAGACTGGTGTTGGA	up	XLOC_012689	up
ssa-miR-1-4-5p	ACATACTTCTTTATATGCCCATA	up	XLOC_012689	up
ssa-miR-26a-4-3p	CCTATTCTTGATTACTTGTTTC	down	XLOC_012689	up
PC-3p-50929_43	TGGAAGTGTCAGAAATTCTGAGT	up	XLOC_012728	down
dre-miR-140-3p_L-1	ACCACAGGGTAGAACCACGGAC	up	XLOC_012728	down
ssa-miR-26d-5p_L+1_1ss13TC	CTTCAAGTAATCCAGGATAGGCT	up	XLOC_012728	down
dre-miR-140-3p_L-1	ACCACAGGGTAGAACCACGGAC	up	XLOC_012823	up
dre-let-7d-5p	TGAGGTAGTTGGTTGTATGGTT	up	XLOC_012824	up
mmu-let-7j_1ss8TG	TGAGGTAGTAGTTTGTGCTGTTAT	up	XLOC_012824	up
tni-let-7j_1ss11TG	TGAGGTAGTTGTTTGTACAGTT	up	XLOC_012824	up
PC-5p-27517_164	TACATGCAGAGGTGGAGCAAGA	up	XLOC_012833	down
PC-3p-50929_43	TGGAAGTGTCAGAAATTCTGAGT	up	XLOC_012871	up
ola-miR-199a-3p_L+1	AACAGTAGTCTGCACATTGGTTA	up	XLOC_012871	up
ola-mir-100-2-p3	CAAGCTCGTATCTATAGGTATG	down	XLOC_012871	up
dre-miR-194a_R+2	TGTAACAGCAACTCCATGTGGAT	up	XLOC_012886	down
ssa-miR-1338-5p_R+1	AGGACTGTCCAACCTGAGAATG	down	XLOC_012886	down
ssa-miR-199a-3p_R+2	ACAGTAGTCTGCACATTGGTTTT	up	XLOC_012886	down
tni-miR-194_R+1	TGTAACAGCAACTCCATGTGGA	up	XLOC_012886	down
PC-5p-8690_526	GATGTTGAGTATCAAACTGTAT	down	XLOC_012890	down
dre-miR-125b-5p_R+1	TCCCTGAGACCCTAACTTGTGAT	up	XLOC_012890	down
dre-miR-140-3p_L-1	ACCACAGGGTAGAACCACGGAC	up	XLOC_012890	down
dre-miR-194a_R+2	TGTAACAGCAACTCCATGTGGAT	up	XLOC_012890	down
dre-miR-21_1ss23CA	TAGCTTATCAGACTGGTGTTGGA	up	XLOC_012890	down
sha-miR-125a_R+2	TCCCTGAGACCCTAACTTGTGAAA	up	XLOC_012890	down
ssa-miR-125b-5p_R-1	TCCCTGAGACCCTTAACCTGTG	up	XLOC_012890	down
ssa-miR-730a-5p_R-1	TCCTCATTGTGCATGCTGTGT	down	XLOC_012890	down
ssa-miR-7a-5p	TGGAAGACTAGTGATTTTGTTGT	up	XLOC_012890	down
ssa-miR-7a-5p_R+1	TGGAAGACTAGTGATTTTGTTGTT	up	XLOC_012890	down
tni-miR-194_R+1	TGTAACAGCAACTCCATGTGGA	up	XLOC_012890	down
PC-5p-45063_62	AAGGATAACTACAACTGTACTT	up	XLOC_012988	up
ola-miR-146a-5p_1ss24TA	TGAGAACTGAATTCCATAGATGGAA	up	XLOC_013074	down
ola-miR-199a-3p_L+1	AACAGTAGTCTGCACATTGGTTA	up	XLOC_013074	down
dre-miR-1	TGGAATGTAAAGAAGTATGTAT	up	XLOC_013076	down
dre-miR-133a-3p_L-1R+1	TTGGTCCCCTTCAACCAGCTGT	up	XLOC_013076	down
dre-miR-133b-3p_R-1	TTTGGTCCCCTTCAACCAGCT	up	XLOC_013076	down
oha-miR-133b-3p	TTTGGTCCCCTTCAACCAGCTAT	up	XLOC_013076	down
ola-miR-194-3p_1ss20CT	CCAGTGGAGGTGCTGTTACTTG	up	XLOC_013076	down
ola-miR-199a-3p_L+1	AACAGTAGTCTGCACATTGGTTA	up	XLOC_013076	down
ssa-miR-1-4-5p	ACATACTTCTTTATATGCCCATA	up	XLOC_013076	down
ssa-miR-206-3p	TGGAATGTAAGGAAGTGTGTGG	up	XLOC_013076	down
ssa-miR-730a-5p_R-1	TCCTCATTGTGCATGCTGTGT	down	XLOC_013076	down
ssc-miR-206	TGGAATGTAAGGAAGTGTGTGA	up	XLOC_013076	down
aca-miR-200b-3p_R+2	TAATACTGCCTGGTAATGATGAAT	up	XLOC_013081	down
dre-miR-122	TGGAGTGTGACAATGGTGTTTG	up	XLOC_013081	down
ola-miR-199a-3p_L+1	AACAGTAGTCTGCACATTGGTTA	up	XLOC_013081	down
ssa-miR-16b-5p_R-1_1ss21TC	TAGCAGCACGTAAATATTGGC	down	XLOC_013082	down
ssa-miR-26a-4-3p	CCTATTCTTGATTACTTGTTTC	down	XLOC_013129	up
PC-5p-8690_526	GATGTTGAGTATCAAACTGTAT	down	XLOC_013135	down
dre-miR-142a-3p_R-1	TGTAGTGTTTCCTACTTTATGG	down	XLOC_013135	down

ssa-miR-26d-5p_L+1_1ss13TC	CTTCAAGTAATCCAGGATAGGCT	up	XLOC_013135	down
tni-miR-10c	TACCCTGTAGATCCGGATTTGT	up	XLOC_013135	down
dre-miR-194a_R+2	TGTAACAGCAACTCCATGTGGAT	up	XLOC_013142	down
tni-miR-194_R+1	TGTAACAGCAACTCCATGTGGA	up	XLOC_013142	down
aca-miR-338-3p_R+2	TCCAGCATCAGTGATTTTGTTAA	up	XLOC_013154	down
dre-miR-122	TGGAGTGTGACAATGGTGTTTG	up	XLOC_013154	down
ola-miR-199a-3p_L+1	AACAGTAGTCTGCACATTGGTTA	up	XLOC_013154	down
rno-miR-122-5p_L+3	ATCTGGAGTGTGACAATGGTGTTTG	up	XLOC_013154	down
aca-miR-338-3p_R+2	TCCAGCATCAGTGATTTTGTTAA	up	XLOC_013175	down
ccr-miR-99_R+3	AACCCGTAGATCCGATCTTGTGAA	up	XLOC_013175	down
dre-miR-22a-3p	AAGCTGCCAGCTGAAGAACTGT	up	XLOC_013175	down
ssa-miR-7a-5p	TGGAAGACTAGTGATTTTGTTGT	up	XLOC_013175	down
ssa-miR-7a-5p_R+1	TGGAAGACTAGTGATTTTGTTGTT	up	XLOC_013175	down
PC-3p-11630_419	ATGAGGAAAAGAAGTTAGGAGA	down	XLOC_013187	down
PC-5p-27517_164	TACATGCAGAGGTGGAGCAAGA	up	XLOC_013187	down
aca-miR-200b-3p_R+2	TAATACTGCCTGGTAATGATGAAT	up	XLOC_013187	down
ssa-miR-1338-5p_R+1	AGGACTGTCCAACCTGAGAATG	down	XLOC_013205	down
ssa-miR-730a-5p_R-1	TCCTCATTGTGCATGCTGTGT	down	XLOC_013205	down
PC-5p-45063_62	AAGGATAACTACAACTGTACTT	up	XLOC_013246	down
aca-miR-200b-3p_R+2	TAATACTGCCTGGTAATGATGAAT	up	XLOC_013246	down
dre-miR-133b-3p_R-1	TTTGGTCCCCTTCAACCAGCT	up	XLOC_013246	down
oha-miR-133b-3p	TTTGGTCCCCTTCAACCAGCTAT	up	XLOC_013246	down
dre-miR-133b-3p_R-1	TTTGGTCCCCTTCAACCAGCT	up	XLOC_013252	up
dre-miR-140-3p_L-1	ACCACAGGGTAGAACCACGGAC	up	XLOC_013252	up
dre-miR-142a-3p_R-1	TGTAGTGTTTCCTACTTTATGG	down	XLOC_013252	up
oha-miR-133b-3p	TTTGGTCCCCTTCAACCAGCTAT	up	XLOC_013252	up
ssa-miR-1-4-5p	ACATACTTCTTTATATGCCCATA	up	XLOC_013252	up
ssa-miR-7132b-5p	GACTTGGTCAAAGCTCCTCAGC	down	XLOC_013252	up
PC-5p-27517_164	TACATGCAGAGGTGGAGCAAGA	up	XLOC_013304	up
aca-miR-200b-3p_R+2	TAATACTGCCTGGTAATGATGAAT	up	XLOC_013304	up
dre-miR-133a-3p_L-1R+1	TTGGTCCCCTTCAACCAGCTGT	up	XLOC_013304	up
ssa-miR-7a-5p	TGGAAGACTAGTGATTTTGTTGT	up	XLOC_013304	up
ssa-miR-7a-5p_R+1	TGGAAGACTAGTGATTTTGTTGTT	up	XLOC_013304	up
PC-3p-11630_419	ATGAGGAAAAGAAGTTAGGAGA	down	XLOC_013312	down
ssa-miR-16b-5p_R-1_1ss21TC	TAGCAGCACGTAAATATTGGC	down	XLOC_013312	down
aca-miR-338-3p_R+2	TCCAGCATCAGTGATTTTGTTAA	up	XLOC_013314	up
dre-miR-142a-3p_R-1	TGTAGTGTTTCCTACTTTATGG	down	XLOC_013314	up
dre-miR-194a_R+2	TGTAACAGCAACTCCATGTGGAT	up	XLOC_013314	up
ola-miR-194-3p_1ss20CT	CCAGTGGAGGTGCTGTTACTTG	up	XLOC_013314	up
rno-miR-122-5p_L+3	ATCTGGAGTGTGACAATGGTGTTTG	up	XLOC_013314	up
ssa-miR-1-4-5p	ACATACTTCTTTATATGCCCATA	up	XLOC_013314	up
ssa-miR-1338-5p_R+1	AGGACTGTCCAACCTGAGAATG	down	XLOC_013314	up
ssa-miR-26d-5p_L+1_1ss13TC	CTTCAAGTAATCCAGGATAGGCT	up	XLOC_013314	up
ssa-miR-730a-5p_R-1	TCCTCATTGTGCATGCTGTGT	down	XLOC_013314	up
ssa-miR-7a-5p	TGGAAGACTAGTGATTTTGTTGT	up	XLOC_013314	up
ssa-miR-7a-5p_R+1	TGGAAGACTAGTGATTTTGTTGTT	up	XLOC_013314	up
tni-miR-194_R+1	TGTAACAGCAACTCCATGTGGA	up	XLOC_013314	up
dre-miR-21_1ss23CA	TAGCTTATCAGACTGGTGTTGGA	up	XLOC_013370	down
ssa-miR-16b-5p_R-1_1ss21TC	TAGCAGCACGTAAATATTGGC	down	XLOC_013370	down
ola-miR-194-3p_1ss20CT	CCAGTGGAGGTGCTGTTACTTG	up	XLOC_013381	down
dre-miR-22a-3p	AAGCTGCCAGCTGAAGAACTGT	up	XLOC_013468	up
dre-miR-140-3p_L-1	ACCACAGGGTAGAACCACGGAC	up	XLOC_013504	down
PC-3p-50929_43	TGGAAGTGTCAGAAATTCTGAGT	up	XLOC_013512	down
dre-miR-22a-3p	AAGCTGCCAGCTGAAGAACTGT	up	XLOC_013512	down

ola-miR-462_L-1R+4	TAACGGAACCCATAATGCAGCT	down	XLOC_013512	down
ola-mir-100-2-p3	CAAGCTCGTATCTATAGGTATG	down	XLOC_013512	down
ssa-miR-7a-5p	TGGAAGACTAGTGATTTTGTTGT	up	XLOC_013512	down
ssa-miR-7a-5p_R+1	TGGAAGACTAGTGATTTTGTTGTT	up	XLOC_013512	down
PC-3p-50929_43	TGGAAGTGTCAGAAATTCTGAGT	up	XLOC_013544	down
aca-miR-338-3p_R+2	TCCAGCATCAGTGATTTTGTTAA	up	XLOC_013544	down
rno-miR-122-5p_L+3	ATCTGGAGTGTGACAATGGTGTTTG	up	XLOC_013544	down
ola-miR-199a-3p_L+1	AACAGTAGTCTGCACATTGGTTA	up	XLOC_013555	down
dre-miR-133a-3p_L-1R+1	TTGGTCCCCTTCAACCAGCTGT	up	XLOC_013572	up
ola-miR-462_L-1R+4	TAACGGAACCCATAATGCAGCT	down	XLOC_013572	up
dre-miR-125b-5p_R+1	TCCCTGAGACCCTAACTTGTGAT	up	XLOC_013583	down
dre-miR-142a-3p_R-1	TGTAGTGTTTCCTACTTTATGG	down	XLOC_013583	down
ola-mir-100-2-p3	CAAGCTCGTATCTATAGGTATG	down	XLOC_013583	down
sha-miR-125a_R+2	TCCCTGAGACCCTAACTTGTGAAA	up	XLOC_013583	down
ssa-miR-1-4-5p	ACATACTTCTTTATATGCCCATA	up	XLOC_013583	down
ssa-miR-125b-5p_R-1	TCCCTGAGACCCTTAACCTGTG	up	XLOC_013583	down
dre-miR-122	TGGAGTGTGACAATGGTGTTTG	up	XLOC_013584	down
ola-miR-146a-5p_1ss24TA	TGAGAACTGAATTCCATAGATGGAA	up	XLOC_013584	down
ola-miR-194-3p_1ss20CT	CCAGTGGAGGTGCTGTTACTTG	up	XLOC_013584	down
ssa-miR-26d-5p_L+1_1ss13TC	CTTCAAGTAATCCAGGATAGGCT	up	XLOC_013584	down
xtr-miR-122_L+1R-1	CTGGAGTGTGACAATGGTGTTTG	up	XLOC_013584	down
dre-miR-140-3p_L-1	ACCACAGGGTAGAACCACGGAC	up	XLOC_013657	up
PC-3p-50929_43	TGGAAGTGTCAGAAATTCTGAGT	up	XLOC_013673	down
aca-miR-338-3p_R+2	TCCAGCATCAGTGATTTTGTTAA	up	XLOC_013673	down
dre-miR-142a-5p	CATAAAGTAGAAAGCACTACT	down	XLOC_013673	down
ssa-miR-26d-5p_L+1_1ss13TC	CTTCAAGTAATCCAGGATAGGCT	up	XLOC_013673	down
ola-miR-146a-5p_1ss24TA	TGAGAACTGAATTCCATAGATGGAA	up	XLOC_013688	up
tni-miR-10c	TACCCTGTAGATCCGGATTTGT	up	XLOC_013688	up
PC-5p-27517_164	TACATGCAGAGGTGGAGCAAGA	up	XLOC_013758	up
ssa-miR-26d-5p_L+1_1ss13TC	CTTCAAGTAATCCAGGATAGGCT	up	XLOC_013758	up
ssa-miR-7132a-5p_R+1	GACTTGGTCAAAGCTCCTCAGTT	down	XLOC_013766	up
ssa-miR-7132b-5p	GACTTGGTCAAAGCTCCTCAGC	down	XLOC_013766	up
dre-miR-140-3p_L-1	ACCACAGGGTAGAACCACGGAC	up	XLOC_013770	up
dre-miR-142a-3p_R-1	TGTAGTGTTTCCTACTTTATGG	down	XLOC_013770	up
PC-3p-11630_419	ATGAGGAAAAGAAGTTAGGAGA	down	XLOC_013780	down
PC-5p-27517_164	TACATGCAGAGGTGGAGCAAGA	up	XLOC_013780	down
ola-miR-194-3p_1ss20CT	CCAGTGGAGGTGCTGTTACTTG	up	XLOC_013780	down
ssa-miR-199a-3p_R+2	ACAGTAGTCTGCACATTGGTTTT	up	XLOC_013839	up
PC-5p-27517_164	TACATGCAGAGGTGGAGCAAGA	up	XLOC_013844	up
dre-miR-142a-5p	CATAAAGTAGAAAGCACTACT	down	XLOC_013869	down
PC-5p-8690_526	GATGTTGAGTATCAAACTGTAT	down	XLOC_013910	down
dre-miR-22a-3p	AAGCTGCCAGCTGAAGAACTGT	up	XLOC_013910	down
ssa-miR-26a-4-3p	CCTATTCTTGATTACTTGTTTC	down	XLOC_013910	down
dre-miR-133b-3p_R-1	TTTGGTCCCCTTCAACCAGCT	up	XLOC_013948	up
dre-miR-142a-3p_R-1	TGTAGTGTTTCCTACTTTATGG	down	XLOC_013948	up
oha-miR-133b-3p	TTTGGTCCCCTTCAACCAGCTAT	up	XLOC_013948	up
ssa-miR-16b-5p_R-1_1ss21TC	TAGCAGCACGTAAATATTGGC	down	XLOC_013948	up
PC-3p-41259_77	TGGCCATTAACTGCTAACCTTC	up	XLOC_013988	up
dre-miR-122	TGGAGTGTGACAATGGTGTTTG	up	XLOC_013988	up
dre-miR-142a-3p_R-1	TGTAGTGTTTCCTACTTTATGG	down	XLOC_013988	up
ola-miR-146a-5p_1ss24TA	TGAGAACTGAATTCCATAGATGGAA	up	XLOC_013988	up
ola-miR-199a-3p_L+1	AACAGTAGTCTGCACATTGGTTA	up	XLOC_013988	up
ola-miR-462_L-1R+4	TAACGGAACCCATAATGCAGCT	down	XLOC_013988	up
ssa-miR-1338-5p_R+1	AGGACTGTCCAACCTGAGAATG	down	XLOC_013988	up

xtr-miR-122_L+1R-1	CTGGAGTGTGACAATGGTGTTTG	up	XLOC_013988	up
ssa-miR-26a-4-3p	CCTATTCTTGATTACTTGTTTC	down	XLOC_013995	up
ssa-miR-7132a-5p_R+1	GACTTGGTCAAAGCTCCTCAGTT	down	XLOC_013995	up
ssa-miR-7132b-3p	TGAGGCGTTTAGAACAAGTTCA	down	XLOC_013995	up
ssa-miR-7132b-5p	GACTTGGTCAAAGCTCCTCAGC	down	XLOC_013995	up
ssa-miR-730a-5p_R-1	TCCTCATTGTGCATGCTGTGT	down	XLOC_013995	up
dre-miR-1	TGGAATGTAAAGAAGTATGTAT	up	XLOC_014000	up
dre-miR-21_1ss23CA	TAGCTTATCAGACTGGTGTTGGA	up	XLOC_014000	up
ssa-miR-206-3p	TGGAATGTAAGGAAGTGTGTGG	up	XLOC_014000	up
ssc-miR-206	TGGAATGTAAGGAAGTGTGTGA	up	XLOC_014000	up
dre-miR-1	TGGAATGTAAAGAAGTATGTAT	up	XLOC_014039	down
ssa-miR-206-3p	TGGAATGTAAGGAAGTGTGTGG	up	XLOC_014039	down
ssa-miR-7132a-5p_R+1	GACTTGGTCAAAGCTCCTCAGTT	down	XLOC_014039	down
ssa-miR-7132b-5p	GACTTGGTCAAAGCTCCTCAGC	down	XLOC_014039	down
ssc-miR-206	TGGAATGTAAGGAAGTGTGTGA	up	XLOC_014039	down
PC-3p-50929_43	TGGAAGTGTCAGAAATTCTGAGT	up	XLOC_014079	down
PC-5p-27517_164	TACATGCAGAGGTGGAGCAAGA	up	XLOC_014079	down
dre-miR-142a-5p	CATAAAGTAGAAAGCACTACT	down	XLOC_014079	down
dre-miR-24_R+2_1	TGGCTCAGTTCAGCAGGAACAGAA	up	XLOC_014081	up
dre-miR-24_R+2_2	TGGCTCAGTTCAGCAGGAACAGTT	up	XLOC_014081	up
aca-miR-338-3p_R+2	TCCAGCATCAGTGATTTTGTTAA	up	XLOC_014082	up
ssa-miR-7132a-5p_R+1	GACTTGGTCAAAGCTCCTCAGTT	down	XLOC_014082	up
ssa-miR-7132b-5p	GACTTGGTCAAAGCTCCTCAGC	down	XLOC_014082	up
ssa-miR-7132a-5p_R+1	GACTTGGTCAAAGCTCCTCAGTT	down	XLOC_014086	up
ssa-miR-7132b-5p	GACTTGGTCAAAGCTCCTCAGC	down	XLOC_014086	up
dre-miR-21_1ss23CA	TAGCTTATCAGACTGGTGTTGGA	up	XLOC_014095	down
dre-miR-1	TGGAATGTAAAGAAGTATGTAT	up	XLOC_014096	down
dre-miR-22a-3p	AAGCTGCCAGCTGAAGAACTGT	up	XLOC_014096	down
ola-miR-199a-3p_L+1	AACAGTAGTCTGCACATTGGTTA	up	XLOC_014096	down
rno-miR-122-5p_L+3	ATCTGGAGTGTGACAATGGTGTTTG	up	XLOC_014096	down
ssa-miR-206-3p	TGGAATGTAAGGAAGTGTGTGG	up	XLOC_014096	down
ssc-miR-206	TGGAATGTAAGGAAGTGTGTGA	up	XLOC_014096	down
dre-miR-133a-3p_L-1R+1	TTGGTCCCCTTCAACCAGCTGT	up	XLOC_014106	up
ssa-miR-1338-5p_R+1	AGGACTGTCCAACCTGAGAATG	down	XLOC_014106	up
ssa-miR-730a-5p_R-1	TCCTCATTGTGCATGCTGTGT	down	XLOC_014106	up
PC-3p-11630_419	ATGAGGAAAAGAAGTTAGGAGA	down	XLOC_014224	up
dre-miR-1	TGGAATGTAAAGAAGTATGTAT	up	XLOC_014224	up
ssa-miR-206-3p	TGGAATGTAAGGAAGTGTGTGG	up	XLOC_014224	up
ssa-miR-730a-5p_R-1	TCCTCATTGTGCATGCTGTGT	down	XLOC_014224	up
ssc-miR-206	TGGAATGTAAGGAAGTGTGTGA	up	XLOC_014224	up
aca-miR-338-3p_R+2	TCCAGCATCAGTGATTTTGTTAA	up	XLOC_014235	down
dre-miR-1	TGGAATGTAAAGAAGTATGTAT	up	XLOC_014235	down
dre-miR-194a_R+2	TGTAACAGCAACTCCATGTGGAT	up	XLOC_014235	down
ssa-miR-1-4-5p	ACATACTTCTTTATATGCCCATA	up	XLOC_014235	down
ssa-miR-206-3p	TGGAATGTAAGGAAGTGTGTGG	up	XLOC_014235	down
ssc-miR-206	TGGAATGTAAGGAAGTGTGTGA	up	XLOC_014235	down
tni-miR-10c	TACCCTGTAGATCCGGATTTGT	up	XLOC_014235	down
tni-miR-194_R+1	TGTAACAGCAACTCCATGTGGA	up	XLOC_014235	down
aca-miR-200b-3p_R+2	TAATACTGCCTGGTAATGATGAAT	up	XLOC_014268	down
dre-miR-194a_R+2	TGTAACAGCAACTCCATGTGGAT	up	XLOC_014268	down
ola-miR-146a-5p_1ss24TA	TGAGAACTGAATTCCATAGATGGAA	up	XLOC_014268	down
ola-miR-194-3p_1ss20CT	CCAGTGGAGGTGCTGTTACTTG	up	XLOC_014268	down
tni-miR-194_R+1	TGTAACAGCAACTCCATGTGGA	up	XLOC_014268	down
xtr-miR-122_L+1R-1	CTGGAGTGTGACAATGGTGTTTG	up	XLOC_014268	down

PC-5p-27517_164	TACATGCAGAGGTGGAGCAAGA	up	XLOC_014300	up
dre-miR-125b-5p_R+1	TCCCTGAGACCCTAACTTGTGAT	up	XLOC_014300	up
ola-miR-199a-3p_L+1	AACAGTAGTCTGCACATTGGTTA	up	XLOC_014300	up
sha-miR-125a_R+2	TCCCTGAGACCCTAACTTGTGAAA	up	XLOC_014300	up
ssa-miR-125b-5p_R-1	TCCCTGAGACCCTTAACCTGTG	up	XLOC_014300	up
ssa-miR-199a-3p_R+2	ACAGTAGTCTGCACATTGGTTTT	up	XLOC_014300	up
ssa-miR-7132b-3p	TGAGGCGTTTAGAACAAGTTCA	down	XLOC_014301	up
PC-3p-50929_43	TGGAAGTGTCAGAAATTCTGAGT	up	XLOC_014319	down
dre-miR-125b-5p_R+1	TCCCTGAGACCCTAACTTGTGAT	up	XLOC_014319	down
dre-miR-140-3p_L-1	ACCACAGGGTAGAACCACGGAC	up	XLOC_014319	down
dre-miR-22a-3p	AAGCTGCCAGCTGAAGAACTGT	up	XLOC_014319	down
sha-miR-125a_R+2	TCCCTGAGACCCTAACTTGTGAAA	up	XLOC_014319	down
ssa-miR-125b-5p_R-1	TCCCTGAGACCCTTAACCTGTG	up	XLOC_014319	down
ssa-miR-1338-5p_R+1	AGGACTGTCCAACCTGAGAATG	down	XLOC_014319	down
ssa-miR-16b-5p_R-1_1ss21TC	TAGCAGCACGTAAATATTGGC	down	XLOC_014319	down
PC-3p-41259_77	TGGCCATTAACTGCTAACCTTC	up	XLOC_014344	down
dre-miR-122	TGGAGTGTGACAATGGTGTTTG	up	XLOC_014353	down
dre-miR-140-3p_L-1	ACCACAGGGTAGAACCACGGAC	up	XLOC_014360	up
ola-miR-199a-3p_L+1	AACAGTAGTCTGCACATTGGTTA	up	XLOC_014360	up
PC-5p-27517_164	TACATGCAGAGGTGGAGCAAGA	up	XLOC_014376	down
aca-miR-200b-3p_R+2	TAATACTGCCTGGTAATGATGAAT	up	XLOC_014376	down
ola-miR-194-3p_1ss20CT	CCAGTGGAGGTGCTGTTACTTG	up	XLOC_014376	down
ola-miR-199a-3p_L+1	AACAGTAGTCTGCACATTGGTTA	up	XLOC_014376	down
ssa-miR-26d-5p_L+1_1ss13TC	CTTCAAGTAATCCAGGATAGGCT	up	XLOC_014376	down
ssa-miR-7132a-5p_R+1	GACTTGGTCAAAGCTCCTCAGTT	down	XLOC_014379	up
ssa-miR-7132b-5p	GACTTGGTCAAAGCTCCTCAGC	down	XLOC_014379	up
PC-3p-11630_419	ATGAGGAAAAGAAGTTAGGAGA	down	XLOC_014412	down
PC-3p-41259_77	TGGCCATTAACTGCTAACCTTC	up	XLOC_014412	down
ola-miR-194-3p_1ss20CT	CCAGTGGAGGTGCTGTTACTTG	up	XLOC_014412	down
xtr-miR-122_L+1R-1	CTGGAGTGTGACAATGGTGTTTG	up	XLOC_014412	down
ola-miR-146a-5p_1ss24TA	TGAGAACTGAATTCCATAGATGGAA	up	XLOC_014419	up
aca-miR-338-3p_R+2	TCCAGCATCAGTGATTTTGTTAA	up	XLOC_014436	up
ssa-miR-7132b-3p	TGAGGCGTTTAGAACAAGTTCA	down	XLOC_014440	up
dre-miR-122	TGGAGTGTGACAATGGTGTTTG	up	XLOC_014525	up
dre-miR-125b-5p_R+1	TCCCTGAGACCCTAACTTGTGAT	up	XLOC_014525	up
rno-miR-122-5p_L+3	ATCTGGAGTGTGACAATGGTGTTTG	up	XLOC_014525	up
sha-miR-125a_R+2	TCCCTGAGACCCTAACTTGTGAAA	up	XLOC_014525	up
ssa-miR-125b-5p_R-1	TCCCTGAGACCCTTAACCTGTG	up	XLOC_014525	up
ssa-miR-7132a-5p_R+1	GACTTGGTCAAAGCTCCTCAGTT	down	XLOC_014525	up
ssa-miR-7132b-5p	GACTTGGTCAAAGCTCCTCAGC	down	XLOC_014525	up
tni-miR-10c	TACCCTGTAGATCCGGATTTGT	up	XLOC_014525	up
xtr-miR-122_L+1R-1	CTGGAGTGTGACAATGGTGTTTG	up	XLOC_014525	up
dre-miR-140-3p_L-1	ACCACAGGGTAGAACCACGGAC	up	XLOC_014570	up
dre-miR-21_1ss23CA	TAGCTTATCAGACTGGTGTTGGA	up	XLOC_014584	down
ola-miR-194-3p_1ss20CT	CCAGTGGAGGTGCTGTTACTTG	up	XLOC_014584	down
PC-5p-27517_164	TACATGCAGAGGTGGAGCAAGA	up	XLOC_014607	down
ssa-miR-730a-5p_R-1	TCCTCATTGTGCATGCTGTGT	down	XLOC_014607	down
ssa-miR-1-4-5p	ACATACTTCTTTATATGCCCATA	up	XLOC_014708	down
tni-miR-10c	TACCCTGTAGATCCGGATTTGT	up	XLOC_014708	down
PC-5p-27517_164	TACATGCAGAGGTGGAGCAAGA	up	XLOC_014713	up
aca-miR-338-3p_R+2	TCCAGCATCAGTGATTTTGTTAA	up	XLOC_014713	up
dre-miR-142a-3p_R-1	TGTAGTGTTTCCTACTTTATGG	down	XLOC_014713	up
ssa-miR-26d-5p_L+1_1ss13TC	CTTCAAGTAATCCAGGATAGGCT	up	XLOC_014713	up
PC-5p-8690_526	GATGTTGAGTATCAAACTGTAT	down	XLOC_014741	down

PC-3p-11630_419	ATGAGGAAAAGAAGTTAGGAGA	down	XLOC_014820	up
dre-miR-122	TGGAGTGTGACAATGGTGTTTG	up	XLOC_014820	up
dre-miR-22a-3p	AAGCTGCCAGCTGAAGAACTGT	up	XLOC_014820	up
xtr-miR-122_L+1R-1	CTGGAGTGTGACAATGGTGTTTG	up	XLOC_014820	up
dre-miR-122	TGGAGTGTGACAATGGTGTTTG	up	XLOC_014853	down
dre-miR-21_1ss23CA	TAGCTTATCAGACTGGTGTTGGA	up	XLOC_014853	down
ola-miR-146a-5p_1ss24TA	TGAGAACTGAATTCCATAGATGGAA	up	XLOC_014853	down
ssa-miR-16b-5p_R-1_1ss21TC	TAGCAGCACGTAAATATTGGC	down	XLOC_014853	down
tni-miR-10c	TACCCTGTAGATCCGGATTTGT	up	XLOC_014853	down
dre-miR-125b-5p_R+1	TCCCTGAGACCCTAACTTGTGAT	up	XLOC_014884	down
dre-miR-24_R+2_1	TGGCTCAGTTCAGCAGGAACAGAA	up	XLOC_014884	down
dre-miR-24_R+2_2	TGGCTCAGTTCAGCAGGAACAGTT	up	XLOC_014884	down
sha-miR-125a_R+2	TCCCTGAGACCCTAACTTGTGAAA	up	XLOC_014884	down
ssa-miR-125b-5p_R-1	TCCCTGAGACCCTTAACCTGTG	up	XLOC_014884	down
PC-5p-27517_164	TACATGCAGAGGTGGAGCAAGA	up	XLOC_014905	up
dre-miR-140-3p_L-1	ACCACAGGGTAGAACCACGGAC	up	XLOC_014905	up
rno-miR-122-5p_L+3	ATCTGGAGTGTGACAATGGTGTTTG	up	XLOC_014905	up
ssa-miR-730a-5p_R-1	TCCTCATTGTGCATGCTGTGT	down	XLOC_014905	up
PC-3p-50929_43	TGGAAGTGTCAGAAATTCTGAGT	up	XLOC_014923	up
dre-miR-21_1ss23CA	TAGCTTATCAGACTGGTGTTGGA	up	XLOC_014923	up
dre-miR-24_R+2_1	TGGCTCAGTTCAGCAGGAACAGAA	up	XLOC_014923	up
dre-miR-24_R+2_2	TGGCTCAGTTCAGCAGGAACAGTT	up	XLOC_014923	up
ssa-miR-26d-5p_L+1_1ss13TC	CTTCAAGTAATCCAGGATAGGCT	up	XLOC_014923	up
dre-miR-142a-3p_R-1	TGTAGTGTTTCCTACTTTATGG	down	XLOC_014997	down
dre-miR-22a-3p	AAGCTGCCAGCTGAAGAACTGT	up	XLOC_014997	down
ola-miR-146a-5p_1ss24TA	TGAGAACTGAATTCCATAGATGGAA	up	XLOC_014997	down
ssa-miR-199a-3p_R+2	ACAGTAGTCTGCACATTGGTTTT	up	XLOC_014997	down
PC-5p-45063_62	AAGGATAACTACAACTGTACTT	up	XLOC_015097	down
aca-miR-200b-3p_R+2	TAATACTGCCTGGTAATGATGAAT	up	XLOC_015097	down
aca-miR-338-3p_R+2	TCCAGCATCAGTGATTTTGTTAA	up	XLOC_015097	down
dre-miR-142a-3p_R-1	TGTAGTGTTTCCTACTTTATGG	down	XLOC_015097	down
dre-miR-194a_R+2	TGTAACAGCAACTCCATGTGGAT	up	XLOC_015097	down
ola-miR-194-3p_1ss20CT	CCAGTGGAGGTGCTGTTACTTG	up	XLOC_015097	down
rno-miR-122-5p_L+3	ATCTGGAGTGTGACAATGGTGTTTG	up	XLOC_015097	down
ssa-miR-16b-5p_R-1_1ss21TC	TAGCAGCACGTAAATATTGGC	down	XLOC_015097	down
tni-miR-194_R+1	TGTAACAGCAACTCCATGTGGA	up	XLOC_015097	down
aca-miR-200b-3p_R+2	TAATACTGCCTGGTAATGATGAAT	up	XLOC_015139	down
dre-miR-142a-3p_R-1	TGTAGTGTTTCCTACTTTATGG	down	XLOC_015140	up
PC-3p-50929_43	TGGAAGTGTCAGAAATTCTGAGT	up	XLOC_015158	up
dre-miR-22a-3p	AAGCTGCCAGCTGAAGAACTGT	up	XLOC_015158	up
xtr-miR-122_L+1R-1	CTGGAGTGTGACAATGGTGTTTG	up	XLOC_015160	down
aca-miR-338-3p_R+2	TCCAGCATCAGTGATTTTGTTAA	up	XLOC_015180	up
dre-miR-122	TGGAGTGTGACAATGGTGTTTG	up	XLOC_015180	up
dre-miR-133b-3p_R-1	TTTGGTCCCCTTCAACCAGCT	up	XLOC_015180	up
oha-miR-133b-3p	TTTGGTCCCCTTCAACCAGCTAT	up	XLOC_015180	up
ola-miR-199a-3p_L+1	AACAGTAGTCTGCACATTGGTTA	up	XLOC_015180	up
dre-miR-194a_R+2	TGTAACAGCAACTCCATGTGGAT	up	XLOC_015208	down
tni-miR-194_R+1	TGTAACAGCAACTCCATGTGGA	up	XLOC_015208	down
aca-miR-200b-3p_R+2	TAATACTGCCTGGTAATGATGAAT	up	XLOC_015224	up
dre-miR-140-3p_L-1	ACCACAGGGTAGAACCACGGAC	up	XLOC_015224	up
ola-miR-194-3p_1ss20CT	CCAGTGGAGGTGCTGTTACTTG	up	XLOC_015224	up
dre-miR-133a-3p_L-1R+1	TTGGTCCCCTTCAACCAGCTGT	up	XLOC_015248	down
ola-miR-194-3p_1ss20CT	CCAGTGGAGGTGCTGTTACTTG	up	XLOC_015248	down
PC-5p-45063_62	AAGGATAACTACAACTGTACTT	up	XLOC_015299	up

PC-5p-27517_164	TACATGCAGAGGTGGAGCAAGA	up	XLOC_015336	down
dre-miR-194a_R+2	TGTAACAGCAACTCCATGTGGAT	up	XLOC_015336	down
tni-miR-194_R+1	TGTAACAGCAACTCCATGTGGA	up	XLOC_015336	down
dre-miR-22a-3p	AAGCTGCCAGCTGAAGAACTGT	up	XLOC_015371	down
dre-miR-24_R+2_1	TGGCTCAGTTCAGCAGGAACAGAA	up	XLOC_015371	down
dre-miR-24_R+2_2	TGGCTCAGTTCAGCAGGAACAGTT	up	XLOC_015371	down
rno-miR-122-5p_L+3	ATCTGGAGTGTGACAATGGTGTTTG	up	XLOC_015390	down
ssa-miR-7132b-3p	TGAGGCGTTTAGAACAAGTTCA	down	XLOC_015390	down
tni-miR-10c	TACCCTGTAGATCCGGATTTGT	up	XLOC_015390	down
aca-miR-200b-3p_R+2	TAATACTGCCTGGTAATGATGAAT	up	XLOC_015415	down
dre-miR-1	TGGAATGTAAAGAAGTATGTAT	up	XLOC_015415	down
ssa-miR-206-3p	TGGAATGTAAGGAAGTGTGTGG	up	XLOC_015415	down
ssc-miR-206	TGGAATGTAAGGAAGTGTGTGA	up	XLOC_015415	down
PC-3p-11630_419	ATGAGGAAAAGAAGTTAGGAGA	down	XLOC_015507	up
ola-miR-146a-5p_1ss24TA	TGAGAACTGAATTCCATAGATGGAA	up	XLOC_015507	up
ola-miR-194-3p_1ss20CT	CCAGTGGAGGTGCTGTTACTTG	up	XLOC_015507	up
ssa-miR-1338-5p_R+1	AGGACTGTCCAACCTGAGAATG	down	XLOC_015507	up
ssa-miR-7a-5p	TGGAAGACTAGTGATTTTGTTGT	up	XLOC_015526	down
ssa-miR-7a-5p_R+1	TGGAAGACTAGTGATTTTGTTGTT	up	XLOC_015526	down
PC-5p-8690_526	GATGTTGAGTATCAAACTGTAT	down	XLOC_015536	up
ola-miR-146a-5p_1ss24TA	TGAGAACTGAATTCCATAGATGGAA	up	XLOC_015536	up
rno-miR-122-5p_L+3	ATCTGGAGTGTGACAATGGTGTTTG	up	XLOC_015536	up
ssa-miR-730a-5p_R-1	TCCTCATTGTGCATGCTGTGT	down	XLOC_015536	up
ola-miR-194-3p_1ss20CT	CCAGTGGAGGTGCTGTTACTTG	up	XLOC_015546	up
dre-miR-194a_R+2	TGTAACAGCAACTCCATGTGGAT	up	XLOC_015569	up
ssa-miR-16b-5p_R-1_1ss21TC	TAGCAGCACGTAAATATTGGC	down	XLOC_015569	up
tni-miR-194_R+1	TGTAACAGCAACTCCATGTGGA	up	XLOC_015569	up
ola-miR-199a-3p_L+1	AACAGTAGTCTGCACATTGGTTA	up	XLOC_015637	down
aca-miR-200b-3p_R+2	TAATACTGCCTGGTAATGATGAAT	up	XLOC_015639	up
dre-let-7d-5p	TGAGGTAGTTGGTTGTATGGTT	up	XLOC_015639	up
dre-miR-21_1ss23CA	TAGCTTATCAGACTGGTGTTGGA	up	XLOC_015639	up
mmu-let-7j_1ss8TG	TGAGGTAGTAGTTTGTGCTGTTAT	up	XLOC_015639	up
ola-miR-194-3p_1ss20CT	CCAGTGGAGGTGCTGTTACTTG	up	XLOC_015639	up
ola-miR-199a-3p_L+1	AACAGTAGTCTGCACATTGGTTA	up	XLOC_015639	up
ssa-miR-16b-5p_R-1_1ss21TC	TAGCAGCACGTAAATATTGGC	down	XLOC_015639	up
ssa-miR-199a-3p_R+2	ACAGTAGTCTGCACATTGGTTTT	up	XLOC_015639	up
tni-let-7j_1ss11TG	TGAGGTAGTTGTTTGTACAGTT	up	XLOC_015639	up
tni-miR-10c	TACCCTGTAGATCCGGATTTGT	up	XLOC_015639	up
ola-miR-194-3p_1ss20CT	CCAGTGGAGGTGCTGTTACTTG	up	XLOC_015645	up
dre-miR-122	TGGAGTGTGACAATGGTGTTTG	up	XLOC_015696	down
xtr-miR-122_L+1R-1	CTGGAGTGTGACAATGGTGTTTG	up	XLOC_015696	down
aca-miR-338-3p_R+2	TCCAGCATCAGTGATTTTGTTAA	up	XLOC_015721	up
ssa-miR-16b-5p_R-1_1ss21TC	TAGCAGCACGTAAATATTGGC	down	XLOC_015721	up
ssa-miR-7a-5p	TGGAAGACTAGTGATTTTGTTGT	up	XLOC_015752	down
ssa-miR-7a-5p_R+1	TGGAAGACTAGTGATTTTGTTGTT	up	XLOC_015752	down
PC-3p-11630_419	ATGAGGAAAAGAAGTTAGGAGA	down	XLOC_015753	up
PC-3p-41259_77	TGGCCATTAACTGCTAACCTTC	up	XLOC_015753	up
aca-miR-338-3p_R+2	TCCAGCATCAGTGATTTTGTTAA	up	XLOC_015753	up
dre-miR-122	TGGAGTGTGACAATGGTGTTTG	up	XLOC_015753	up
ola-miR-146a-5p_1ss24TA	TGAGAACTGAATTCCATAGATGGAA	up	XLOC_015753	up
ssa-miR-16b-5p_R-1_1ss21TC	TAGCAGCACGTAAATATTGGC	down	XLOC_015753	up
xtr-miR-122_L+1R-1	CTGGAGTGTGACAATGGTGTTTG	up	XLOC_015753	up
PC-3p-11630_419	ATGAGGAAAAGAAGTTAGGAGA	down	XLOC_015804	down
PC-3p-50929_43	TGGAAGTGTCAGAAATTCTGAGT	up	XLOC_015804	down

ssa-miR-16b-5p_R-1_1ss21TC	TAGCAGCACGTAAATATTGGC	down	XLOC_015881	up
ola-miR-194-3p_1ss20CT	CCAGTGGAGGTGCTGTTACTTG	up	XLOC_015887	up
ssa-miR-16b-5p_R-1_1ss21TC	TAGCAGCACGTAAATATTGGC	down	XLOC_015887	up
ola-miR-194-3p_1ss20CT	CCAGTGGAGGTGCTGTTACTTG	up	XLOC_015888	up
aca-miR-338-3p_R+2	TCCAGCATCAGTGATTTTGTTAA	up	XLOC_015893	up
dre-let-7d-5p	TGAGGTAGTTGGTTGTATGGTT	up	XLOC_015893	up
dre-miR-140-3p_L-1	ACCACAGGGTAGAACCACGGAC	up	XLOC_015893	up
dre-miR-142a-3p_R-1	TGTAGTGTTTCCTACTTTATGG	down	XLOC_015893	up
mmu-let-7j_1ss8TG	TGAGGTAGTAGTTTGTGCTGTTAT	up	XLOC_015893	up
ssa-miR-16b-5p_R-1_1ss21TC	TAGCAGCACGTAAATATTGGC	down	XLOC_015893	up
tni-let-7j_1ss11TG	TGAGGTAGTTGTTTGTACAGTT	up	XLOC_015893	up
dre-miR-142a-3p_R-1	TGTAGTGTTTCCTACTTTATGG	down	XLOC_015947	down
PC-3p-11630_419	ATGAGGAAAAGAAGTTAGGAGA	down	XLOC_015955	down
PC-3p-41259_77	TGGCCATTAACTGCTAACCTTC	up	XLOC_015955	down
aca-miR-338-3p_R+2	TCCAGCATCAGTGATTTTGTTAA	up	XLOC_015955	down
dre-miR-122	TGGAGTGTGACAATGGTGTTTG	up	XLOC_015955	down
dre-miR-133a-3p_L-1R+1	TTGGTCCCCTTCAACCAGCTGT	up	XLOC_015955	down
ola-mir-100-2-p3	CAAGCTCGTATCTATAGGTATG	down	XLOC_015955	down
dre-miR-24_R+2_1	TGGCTCAGTTCAGCAGGAACAGAA	up	XLOC_016042	up
dre-miR-24_R+2_2	TGGCTCAGTTCAGCAGGAACAGTT	up	XLOC_016042	up
dre-miR-1	TGGAATGTAAAGAAGTATGTAT	up	XLOC_016046	down
ssa-miR-206-3p	TGGAATGTAAGGAAGTGTGTGG	up	XLOC_016046	down
ssc-miR-206	TGGAATGTAAGGAAGTGTGTGA	up	XLOC_016046	down
dre-miR-125b-5p_R+1	TCCCTGAGACCCTAACTTGTGAT	up	XLOC_016049	down
dre-miR-22a-3p	AAGCTGCCAGCTGAAGAACTGT	up	XLOC_016049	down
sha-miR-125a_R+2	TCCCTGAGACCCTAACTTGTGAAA	up	XLOC_016049	down
ssa-miR-125b-5p_R-1	TCCCTGAGACCCTTAACCTGTG	up	XLOC_016049	down
PC-3p-50929_43	TGGAAGTGTCAGAAATTCTGAGT	up	XLOC_016055	down
aca-miR-338-3p_R+2	TCCAGCATCAGTGATTTTGTTAA	up	XLOC_016055	down
aca-miR-200b-3p_R+2	TAATACTGCCTGGTAATGATGAAT	up	XLOC_016093	down
aca-miR-338-3p_R+2	TCCAGCATCAGTGATTTTGTTAA	up	XLOC_016093	down
PC-3p-50929_43	TGGAAGTGTCAGAAATTCTGAGT	up	XLOC_016109	down
PC-5p-27517_164	TACATGCAGAGGTGGAGCAAGA	up	XLOC_016109	down
dre-miR-1	TGGAATGTAAAGAAGTATGTAT	up	XLOC_016109	down
dre-miR-122	TGGAGTGTGACAATGGTGTTTG	up	XLOC_016109	down
ssa-miR-16b-5p_R-1_1ss21TC	TAGCAGCACGTAAATATTGGC	down	XLOC_016109	down
ssa-miR-206-3p	TGGAATGTAAGGAAGTGTGTGG	up	XLOC_016109	down
ssa-miR-26d-5p_L+1_1ss13TC	CTTCAAGTAATCCAGGATAGGCT	up	XLOC_016109	down
ssc-miR-206	TGGAATGTAAGGAAGTGTGTGA	up	XLOC_016109	down
aca-miR-200b-3p_R+2	TAATACTGCCTGGTAATGATGAAT	up	XLOC_016118	up
dre-miR-142a-5p	CATAAAGTAGAAAGCACTACT	down	XLOC_016118	up
tni-miR-10c	TACCCTGTAGATCCGGATTTGT	up	XLOC_016118	up
dre-miR-24_R+2_1	TGGCTCAGTTCAGCAGGAACAGAA	up	XLOC_016147	down
dre-miR-24_R+2_2	TGGCTCAGTTCAGCAGGAACAGTT	up	XLOC_016147	down
ssa-mir-15c-2-p3_1ss11CA	TGCGAACCATAATTTGCTGCTT	down	XLOC_016147	down
PC-3p-11630_419	ATGAGGAAAAGAAGTTAGGAGA	down	XLOC_016152	up
dre-miR-194a_R+2	TGTAACAGCAACTCCATGTGGAT	up	XLOC_016152	up
ola-miR-194-3p_1ss20CT	CCAGTGGAGGTGCTGTTACTTG	up	XLOC_016152	up
tni-miR-194_R+1	TGTAACAGCAACTCCATGTGGA	up	XLOC_016152	up
PC-5p-27517_164	TACATGCAGAGGTGGAGCAAGA	up	XLOC_016185	down
ola-miR-199a-3p_L+1	AACAGTAGTCTGCACATTGGTTA	up	XLOC_016185	down
ssa-miR-16b-5p_R-1_1ss21TC	TAGCAGCACGTAAATATTGGC	down	XLOC_016185	down
tni-let-7j_1ss11TG	TGAGGTAGTTGTTTGTACAGTT	up	XLOC_016185	down
aca-miR-338-3p_R+2	TCCAGCATCAGTGATTTTGTTAA	up	XLOC_016187	down

dre-miR-140-3p_L-1	ACCACAGGGTAGAACCACGGAC	up	XLOC_016187	down
dre-miR-21_1ss23CA	TAGCTTATCAGACTGGTGTTGGA	up	XLOC_016187	down
dre-miR-22a-3p	AAGCTGCCAGCTGAAGAACTGT	up	XLOC_016187	down
ola-mir-100-2-p3	CAAGCTCGTATCTATAGGTATG	down	XLOC_016187	down
ssa-miR-730a-5p_R-1	TCCTCATTGTGCATGCTGTGT	down	XLOC_016187	down
tni-miR-10c	TACCCTGTAGATCCGGATTTGT	up	XLOC_016187	down
PC-5p-27517_164	TACATGCAGAGGTGGAGCAAGA	up	XLOC_016188	up
dre-miR-24_R+2_1	TGGCTCAGTTCAGCAGGAACAGAA	up	XLOC_016188	up
dre-miR-24_R+2_2	TGGCTCAGTTCAGCAGGAACAGTT	up	XLOC_016188	up
PC-5p-8690_526	GATGTTGAGTATCAAACTGTAT	down	XLOC_016203	down
dre-miR-1	TGGAATGTAAAGAAGTATGTAT	up	XLOC_016203	down
ola-miR-146a-5p_1ss24TA	TGAGAACTGAATTCCATAGATGGAA	up	XLOC_016203	down
rno-miR-122-5p_L+3	ATCTGGAGTGTGACAATGGTGTTTG	up	XLOC_016203	down
ssa-miR-206-3p	TGGAATGTAAGGAAGTGTGTGG	up	XLOC_016203	down
ssa-miR-730a-5p_R-1	TCCTCATTGTGCATGCTGTGT	down	XLOC_016203	down
ssc-miR-206	TGGAATGTAAGGAAGTGTGTGA	up	XLOC_016203	down
aca-miR-200b-3p_R+2	TAATACTGCCTGGTAATGATGAAT	up	XLOC_016211	down
dre-miR-125b-5p_R+1	TCCCTGAGACCCTAACTTGTGAT	up	XLOC_016211	down
ola-miR-199a-3p_L+1	AACAGTAGTCTGCACATTGGTTA	up	XLOC_016211	down
sha-miR-125a_R+2	TCCCTGAGACCCTAACTTGTGAAA	up	XLOC_016211	down
ssa-miR-125b-5p_R-1	TCCCTGAGACCCTTAACCTGTG	up	XLOC_016211	down
ssa-miR-7132a-5p_R+1	GACTTGGTCAAAGCTCCTCAGTT	down	XLOC_016211	down
ssa-miR-7132b-5p	GACTTGGTCAAAGCTCCTCAGC	down	XLOC_016211	down
dre-miR-1	TGGAATGTAAAGAAGTATGTAT	up	XLOC_016262	up
ssa-miR-206-3p	TGGAATGTAAGGAAGTGTGTGG	up	XLOC_016262	up
ssc-miR-206	TGGAATGTAAGGAAGTGTGTGA	up	XLOC_016262	up
ssa-miR-730a-5p_R-1	TCCTCATTGTGCATGCTGTGT	down	XLOC_016270	up
dre-miR-21_1ss23CA	TAGCTTATCAGACTGGTGTTGGA	up	XLOC_016271	up
ssa-miR-26a-4-3p	CCTATTCTTGATTACTTGTTTC	down	XLOC_016271	up
dre-let-7d-5p	TGAGGTAGTTGGTTGTATGGTT	up	XLOC_016272	up
dre-miR-142a-5p	CATAAAGTAGAAAGCACTACT	down	XLOC_016272	up
dre-miR-21_1ss23CA	TAGCTTATCAGACTGGTGTTGGA	up	XLOC_016272	up
mmu-let-7j_1ss8TG	TGAGGTAGTAGTTTGTGCTGTTAT	up	XLOC_016272	up
tni-let-7j_1ss11TG	TGAGGTAGTTGTTTGTACAGTT	up	XLOC_016272	up
dre-miR-21_1ss23CA	TAGCTTATCAGACTGGTGTTGGA	up	XLOC_016282	down
ssa-miR-7132a-5p_R+1	GACTTGGTCAAAGCTCCTCAGTT	down	XLOC_016282	down
ssa-miR-7132b-5p	GACTTGGTCAAAGCTCCTCAGC	down	XLOC_016282	down
ola-miR-194-3p_1ss20CT	CCAGTGGAGGTGCTGTTACTTG	up	XLOC_016288	down
ssa-miR-7a-5p	TGGAAGACTAGTGATTTTGTTGT	up	XLOC_016319	down
ssa-miR-7a-5p_R+1	TGGAAGACTAGTGATTTTGTTGTT	up	XLOC_016319	down
ola-mir-100-2-p3	CAAGCTCGTATCTATAGGTATG	down	XLOC_016324	up
ssa-miR-7132b-3p	TGAGGCGTTTAGAACAAGTTCA	down	XLOC_016324	up
tni-miR-10c	TACCCTGTAGATCCGGATTTGT	up	XLOC_016324	up
PC-5p-45063_62	AAGGATAACTACAACTGTACTT	up	XLOC_016416	down
PC-3p-41259_77	TGGCCATTAACTGCTAACCTTC	up	XLOC_016425	down
PC-5p-45063_62	AAGGATAACTACAACTGTACTT	up	XLOC_016425	down
rno-miR-122-5p_L+3	ATCTGGAGTGTGACAATGGTGTTTG	up	XLOC_016445	down
ssa-miR-199a-3p_R+2	ACAGTAGTCTGCACATTGGTTTT	up	XLOC_016445	down
ola-miR-194-3p_1ss20CT	CCAGTGGAGGTGCTGTTACTTG	up	XLOC_016447	down
dre-let-7d-5p	TGAGGTAGTTGGTTGTATGGTT	up	XLOC_016514	down
dre-miR-194a_R+2	TGTAACAGCAACTCCATGTGGAT	up	XLOC_016514	down
mmu-let-7j_1ss8TG	TGAGGTAGTAGTTTGTGCTGTTAT	up	XLOC_016514	down
tni-let-7j_1ss11TG	TGAGGTAGTTGTTTGTACAGTT	up	XLOC_016514	down
tni-miR-194_R+1	TGTAACAGCAACTCCATGTGGA	up	XLOC_016514	down

ola-miR-194-3p_1ss20CT	CCAGTGGAGGTGCTGTTACTTG	up	XLOC_016555	down
ola-miR-199a-3p_L+1	AACAGTAGTCTGCACATTGGTTA	up	XLOC_016557	down
ssa-miR-199a-3p_R+2	ACAGTAGTCTGCACATTGGTTTT	up	XLOC_016557	down
ssa-miR-7132b-3p	TGAGGCGTTTAGAACAAGTTCA	down	XLOC_016601	up
tni-miR-10c	TACCCTGTAGATCCGGATTTGT	up	XLOC_016601	up
dre-miR-22a-3p	AAGCTGCCAGCTGAAGAACTGT	up	XLOC_016625	up
rno-miR-122-5p_L+3	ATCTGGAGTGTGACAATGGTGTTTG	up	XLOC_016625	up
dre-miR-194a_R+2	TGTAACAGCAACTCCATGTGGAT	up	XLOC_016638	down
ssa-miR-1-4-5p	ACATACTTCTTTATATGCCCATA	up	XLOC_016638	down
tni-miR-194_R+1	TGTAACAGCAACTCCATGTGGA	up	XLOC_016638	down
PC-3p-11630_419	ATGAGGAAAAGAAGTTAGGAGA	down	XLOC_016673	down
ssa-miR-26d-5p_L+1_1ss13TC	CTTCAAGTAATCCAGGATAGGCT	up	XLOC_016673	down
ola-miR-199a-3p_L+1	AACAGTAGTCTGCACATTGGTTA	up	XLOC_016706	down
rno-miR-122-5p_L+3	ATCTGGAGTGTGACAATGGTGTTTG	up	XLOC_016706	down
ssa-miR-199a-3p_R+2	ACAGTAGTCTGCACATTGGTTTT	up	XLOC_016706	down
dre-miR-140-3p_L-1	ACCACAGGGTAGAACCACGGAC	up	XLOC_016719	up
ssa-miR-16b-5p_R-1_1ss21TC	TAGCAGCACGTAAATATTGGC	down	XLOC_016719	up
dre-miR-122	TGGAGTGTGACAATGGTGTTTG	up	XLOC_016724	down
dre-miR-133b-3p_R-1	TTTGGTCCCCTTCAACCAGCT	up	XLOC_016724	down
oha-miR-133b-3p	TTTGGTCCCCTTCAACCAGCTAT	up	XLOC_016724	down
ola-miR-199a-3p_L+1	AACAGTAGTCTGCACATTGGTTA	up	XLOC_016724	down
rno-miR-122-5p_L+3	ATCTGGAGTGTGACAATGGTGTTTG	up	XLOC_016724	down
xtr-miR-122_L+1R-1	CTGGAGTGTGACAATGGTGTTTG	up	XLOC_016724	down
PC-5p-8690_526	GATGTTGAGTATCAAACTGTAT	down	XLOC_016810	down
aca-miR-200b-3p_R+2	TAATACTGCCTGGTAATGATGAAT	up	XLOC_016810	down
dre-miR-1	TGGAATGTAAAGAAGTATGTAT	up	XLOC_016810	down
ssa-miR-206-3p	TGGAATGTAAGGAAGTGTGTGG	up	XLOC_016810	down
ssc-miR-206	TGGAATGTAAGGAAGTGTGTGA	up	XLOC_016810	down
dre-miR-194a_R+2	TGTAACAGCAACTCCATGTGGAT	up	XLOC_016822	down
ssa-miR-26d-5p_L+1_1ss13TC	CTTCAAGTAATCCAGGATAGGCT	up	XLOC_016822	down
ssa-miR-7a-5p	TGGAAGACTAGTGATTTTGTTGT	up	XLOC_016822	down
ssa-miR-7a-5p_R+1	TGGAAGACTAGTGATTTTGTTGTT	up	XLOC_016822	down
tni-miR-194_R+1	TGTAACAGCAACTCCATGTGGA	up	XLOC_016822	down
dre-miR-22a-3p	AAGCTGCCAGCTGAAGAACTGT	up	XLOC_016826	down
ola-miR-146a-5p_1ss24TA	TGAGAACTGAATTCCATAGATGGAA	up	XLOC_016826	down
ssa-miR-730a-5p_R-1	TCCTCATTGTGCATGCTGTGT	down	XLOC_016826	down
dre-miR-24_R+2_1	TGGCTCAGTTCAGCAGGAACAGAA	up	XLOC_016870	down
dre-miR-24_R+2_2	TGGCTCAGTTCAGCAGGAACAGTT	up	XLOC_016870	down
ssa-miR-16b-5p_R-1_1ss21TC	TAGCAGCACGTAAATATTGGC	down	XLOC_016870	down
PC-5p-27517_164	TACATGCAGAGGTGGAGCAAGA	up	XLOC_016918	up
dre-miR-1	TGGAATGTAAAGAAGTATGTAT	up	XLOC_016918	up
ssa-miR-16b-5p_R-1_1ss21TC	TAGCAGCACGTAAATATTGGC	down	XLOC_016918	up
ssa-miR-206-3p	TGGAATGTAAGGAAGTGTGTGG	up	XLOC_016918	up
ssa-miR-26d-5p_L+1_1ss13TC	CTTCAAGTAATCCAGGATAGGCT	up	XLOC_016918	up
ssa-miR-730a-5p_R-1	TCCTCATTGTGCATGCTGTGT	down	XLOC_016918	up
ssc-miR-206	TGGAATGTAAGGAAGTGTGTGA	up	XLOC_016918	up
PC-3p-41259_77	TGGCCATTAACTGCTAACCTTC	up	XLOC_016923	down
PC-5p-27517_164	TACATGCAGAGGTGGAGCAAGA	up	XLOC_016923	down
dre-miR-125b-5p_R+1	TCCCTGAGACCCTAACTTGTGAT	up	XLOC_016923	down
dre-miR-142a-3p_R-1	TGTAGTGTTTCCTACTTTATGG	down	XLOC_016923	down
sha-miR-125a_R+2	TCCCTGAGACCCTAACTTGTGAAA	up	XLOC_016923	down
ssa-miR-125b-5p_R-1	TCCCTGAGACCCTTAACCTGTG	up	XLOC_016923	down
ssa-miR-7132a-5p_R+1	GACTTGGTCAAAGCTCCTCAGTT	down	XLOC_016923	down
ssa-miR-7132b-5p	GACTTGGTCAAAGCTCCTCAGC	down	XLOC_016923	down

ssa-miR-7132a-5p_R+1	GACTTGGTCAAAGCTCCTCAGTT	down	XLOC_016964	down
ssa-miR-7132b-5p	GACTTGGTCAAAGCTCCTCAGC	down	XLOC_016964	down
ssa-miR-16b-5p_R-1_1ss21TC	TAGCAGCACGTAAATATTGGC	down	XLOC_016986	up
PC-5p-27517_164	TACATGCAGAGGTGGAGCAAGA	up	XLOC_016988	up
PC-5p-8690_526	GATGTTGAGTATCAAACTGTAT	down	XLOC_016988	up
ssa-miR-16b-5p_R-1_1ss21TC	TAGCAGCACGTAAATATTGGC	down	XLOC_016988	up
PC-5p-27517_164	TACATGCAGAGGTGGAGCAAGA	up	XLOC_017023	up
ssa-miR-7a-5p	TGGAAGACTAGTGATTTTGTTGT	up	XLOC_017023	up
ssa-miR-7a-5p_R+1	TGGAAGACTAGTGATTTTGTTGTT	up	XLOC_017023	up
aca-miR-200b-3p_R+2	TAATACTGCCTGGTAATGATGAAT	up	XLOC_017030	down
rno-miR-122-5p_L+3	ATCTGGAGTGTGACAATGGTGTTTG	up	XLOC_017030	down
ssa-miR-16b-5p_R-1_1ss21TC	TAGCAGCACGTAAATATTGGC	down	XLOC_017030	down
dre-miR-22a-3p	AAGCTGCCAGCTGAAGAACTGT	up	XLOC_017060	down
dre-miR-24_R+2_1	TGGCTCAGTTCAGCAGGAACAGAA	up	XLOC_017060	down
dre-miR-24_R+2_2	TGGCTCAGTTCAGCAGGAACAGTT	up	XLOC_017060	down
PC-3p-50929_43	TGGAAGTGTCAGAAATTCTGAGT	up	XLOC_017111	down
PC-5p-27517_164	TACATGCAGAGGTGGAGCAAGA	up	XLOC_017111	down
PC-5p-8690_526	GATGTTGAGTATCAAACTGTAT	down	XLOC_017111	down
dre-miR-140-3p_L-1	ACCACAGGGTAGAACCACGGAC	up	XLOC_017111	down
tni-miR-10c	TACCCTGTAGATCCGGATTTGT	up	XLOC_017111	down
rno-miR-122-5p_L+3	ATCTGGAGTGTGACAATGGTGTTTG	up	XLOC_017163	up
ssa-miR-26d-5p_L+1_1ss13TC	CTTCAAGTAATCCAGGATAGGCT	up	XLOC_017163	up
ssa-miR-730a-5p_R-1	TCCTCATTGTGCATGCTGTGT	down	XLOC_017163	up
dre-miR-133b-3p_R-1	TTTGGTCCCCTTCAACCAGCT	up	XLOC_017174	down
dre-miR-140-3p_L-1	ACCACAGGGTAGAACCACGGAC	up	XLOC_017174	down
dre-miR-24_R+2_1	TGGCTCAGTTCAGCAGGAACAGAA	up	XLOC_017174	down
dre-miR-24_R+2_2	TGGCTCAGTTCAGCAGGAACAGTT	up	XLOC_017174	down
oha-miR-133b-3p	TTTGGTCCCCTTCAACCAGCTAT	up	XLOC_017174	down
ssa-miR-16b-5p_R-1_1ss21TC	TAGCAGCACGTAAATATTGGC	down	XLOC_017174	down
ola-miR-199a-3p_L+1	AACAGTAGTCTGCACATTGGTTA	up	XLOC_017185	down
rno-miR-122-5p_L+3	ATCTGGAGTGTGACAATGGTGTTTG	up	XLOC_017185	down
PC-5p-27517_164	TACATGCAGAGGTGGAGCAAGA	up	XLOC_017188	up
rno-miR-122-5p_L+3	ATCTGGAGTGTGACAATGGTGTTTG	up	XLOC_017188	up
ssa-miR-26a-4-3p	CCTATTCTTGATTACTTGTTTC	down	XLOC_017188	up
aca-miR-338-3p_R+2	TCCAGCATCAGTGATTTTGTTAA	up	XLOC_017211	down
PC-3p-50929_43	TGGAAGTGTCAGAAATTCTGAGT	up	XLOC_017213	down
PC-5p-45063_62	AAGGATAACTACAACTGTACTT	up	XLOC_017213	down
aca-miR-338-3p_R+2	TCCAGCATCAGTGATTTTGTTAA	up	XLOC_017213	down
dre-miR-194a_R+2	TGTAACAGCAACTCCATGTGGAT	up	XLOC_017213	down
ssa-miR-7a-5p	TGGAAGACTAGTGATTTTGTTGT	up	XLOC_017213	down
ssa-miR-7a-5p_R+1	TGGAAGACTAGTGATTTTGTTGTT	up	XLOC_017213	down
tni-miR-194_R+1	TGTAACAGCAACTCCATGTGGA	up	XLOC_017213	down
PC-5p-45063_62	AAGGATAACTACAACTGTACTT	up	XLOC_017214	up
aca-miR-200b-3p_R+2	TAATACTGCCTGGTAATGATGAAT	up	XLOC_017214	up
dre-miR-21_1ss23CA	TAGCTTATCAGACTGGTGTTGGA	up	XLOC_017214	up
dre-miR-24_R+2_1	TGGCTCAGTTCAGCAGGAACAGAA	up	XLOC_017214	up
dre-miR-24_R+2_2	TGGCTCAGTTCAGCAGGAACAGTT	up	XLOC_017214	up
ssa-miR-26a-4-3p	CCTATTCTTGATTACTTGTTTC	down	XLOC_017214	up
PC-5p-8690_526	GATGTTGAGTATCAAACTGTAT	down	XLOC_017221	down
dre-miR-24_R+2_1	TGGCTCAGTTCAGCAGGAACAGAA	up	XLOC_017221	down
dre-miR-24_R+2_2	TGGCTCAGTTCAGCAGGAACAGTT	up	XLOC_017221	down
ssa-miR-26d-5p_L+1_1ss13TC	CTTCAAGTAATCCAGGATAGGCT	up	XLOC_017238	down
ssa-miR-730a-5p_R-1	TCCTCATTGTGCATGCTGTGT	down	XLOC_017238	down
tni-miR-10c	TACCCTGTAGATCCGGATTTGT	up	XLOC_017238	down

ola-miR-199a-3p_L+1	AACAGTAGTCTGCACATTGGTTA	up	XLOC_017264	down
ssa-miR-16b-5p_R-1_1ss21TC	TAGCAGCACGTAAATATTGGC	down	XLOC_017264	down
PC-5p-27517_164	TACATGCAGAGGTGGAGCAAGA	up	XLOC_017327	down
PC-3p-11630_419	ATGAGGAAAAGAAGTTAGGAGA	down	XLOC_017359	up
aca-miR-338-3p_R+2	TCCAGCATCAGTGATTTTGTTAA	up	XLOC_017359	up
dre-miR-24_R+2_1	TGGCTCAGTTCAGCAGGAACAGAA	up	XLOC_017359	up
dre-miR-24_R+2_2	TGGCTCAGTTCAGCAGGAACAGTT	up	XLOC_017359	up
ssa-miR-1338-5p_R+1	AGGACTGTCCAACCTGAGAATG	down	XLOC_017359	up
ssa-miR-16b-5p_R-1_1ss21TC	TAGCAGCACGTAAATATTGGC	down	XLOC_017359	up
xtr-miR-122_L+1R-1	CTGGAGTGTGACAATGGTGTTTG	up	XLOC_017359	up
dre-miR-24_R+2_1	TGGCTCAGTTCAGCAGGAACAGAA	up	XLOC_017360	up
dre-miR-24_R+2_2	TGGCTCAGTTCAGCAGGAACAGTT	up	XLOC_017360	up
dre-miR-142a-5p	CATAAAGTAGAAAGCACTACT	down	XLOC_017376	down
dre-miR-21_1ss23CA	TAGCTTATCAGACTGGTGTTGGA	up	XLOC_017384	up
PC-5p-27517_164	TACATGCAGAGGTGGAGCAAGA	up	XLOC_017411	down
aca-miR-200b-3p_R+2	TAATACTGCCTGGTAATGATGAAT	up	XLOC_017506	up
ssa-miR-199a-3p_R+2	ACAGTAGTCTGCACATTGGTTTT	up	XLOC_017506	up
PC-3p-11630_419	ATGAGGAAAAGAAGTTAGGAGA	down	XLOC_017573	down
PC-3p-41259_77	TGGCCATTAACTGCTAACCTTC	up	XLOC_017573	down
aca-miR-338-3p_R+2	TCCAGCATCAGTGATTTTGTTAA	up	XLOC_017573	down
dre-miR-1	TGGAATGTAAAGAAGTATGTAT	up	XLOC_017573	down
dre-miR-140-3p_L-1	ACCACAGGGTAGAACCACGGAC	up	XLOC_017573	down
dre-miR-142a-5p	CATAAAGTAGAAAGCACTACT	down	XLOC_017573	down
dre-miR-194a_R+2	TGTAACAGCAACTCCATGTGGAT	up	XLOC_017573	down
ola-miR-199a-3p_L+1	AACAGTAGTCTGCACATTGGTTA	up	XLOC_017573	down
ssa-miR-16b-5p_R-1_1ss21TC	TAGCAGCACGTAAATATTGGC	down	XLOC_017573	down
ssa-miR-206-3p	TGGAATGTAAGGAAGTGTGTGG	up	XLOC_017573	down
ssc-miR-206	TGGAATGTAAGGAAGTGTGTGA	up	XLOC_017573	down
tni-miR-10c	TACCCTGTAGATCCGGATTTGT	up	XLOC_017573	down
tni-miR-194_R+1	TGTAACAGCAACTCCATGTGGA	up	XLOC_017573	down
ssa-miR-16b-5p_R-1_1ss21TC	TAGCAGCACGTAAATATTGGC	down	XLOC_017576	up
ssa-miR-7a-5p	TGGAAGACTAGTGATTTTGTTGT	up	XLOC_017576	up
ssa-miR-7a-5p_R+1	TGGAAGACTAGTGATTTTGTTGTT	up	XLOC_017576	up
tni-miR-10c	TACCCTGTAGATCCGGATTTGT	up	XLOC_017576	up
dre-miR-142a-5p	CATAAAGTAGAAAGCACTACT	down	XLOC_017601	up
ssa-miR-730a-5p_R-1	TCCTCATTGTGCATGCTGTGT	down	XLOC_017601	up
ssa-miR-1-4-5p	ACATACTTCTTTATATGCCCATA	up	XLOC_017660	down
ssa-miR-16b-5p_R-1_1ss21TC	TAGCAGCACGTAAATATTGGC	down	XLOC_017660	down
PC-5p-27517_164	TACATGCAGAGGTGGAGCAAGA	up	XLOC_017678	down
ssa-miR-16b-5p_R-1_1ss21TC	TAGCAGCACGTAAATATTGGC	down	XLOC_017718	up
PC-5p-45063_62	AAGGATAACTACAACTGTACTT	up	XLOC_017737	up
ola-miR-199a-3p_L+1	AACAGTAGTCTGCACATTGGTTA	up	XLOC_017737	up
xtr-miR-122_L+1R-1	CTGGAGTGTGACAATGGTGTTTG	up	XLOC_017761	up
PC-3p-11630_419	ATGAGGAAAAGAAGTTAGGAGA	down	XLOC_017878	up
dre-miR-21_1ss23CA	TAGCTTATCAGACTGGTGTTGGA	up	XLOC_017893	down
dre-miR-140-3p_L-1	ACCACAGGGTAGAACCACGGAC	up	XLOC_017918	up
dre-miR-133b-3p_R-1	TTTGGTCCCCTTCAACCAGCT	up	XLOC_017929	down
oha-miR-133b-3p	TTTGGTCCCCTTCAACCAGCTAT	up	XLOC_017929	down
aca-miR-200b-3p_R+2	TAATACTGCCTGGTAATGATGAAT	up	XLOC_017933	down
dre-miR-140-3p_L-1	ACCACAGGGTAGAACCACGGAC	up	XLOC_017933	down
rno-miR-122-5p_L+3	ATCTGGAGTGTGACAATGGTGTTTG	up	XLOC_017933	down
PC-3p-50929_43	TGGAAGTGTCAGAAATTCTGAGT	up	XLOC_017937	up
dre-miR-22a-3p	AAGCTGCCAGCTGAAGAACTGT	up	XLOC_017937	up
dre-miR-125b-5p_R+1	TCCCTGAGACCCTAACTTGTGAT	up	XLOC_017962	up

sha-miR-125a_R+2	TCCCTGAGACCCTAACTTGTGAAA	up	XLOC_017962	up
ssa-miR-125b-5p_R-1	TCCCTGAGACCCTTAACCTGTG	up	XLOC_017962	up
ssa-miR-730a-5p_R-1	TCCTCATTGTGCATGCTGTGT	down	XLOC_017962	up
aca-miR-338-3p_R+2	TCCAGCATCAGTGATTTTGTTAA	up	XLOC_017979	down
dre-miR-22a-3p	AAGCTGCCAGCTGAAGAACTGT	up	XLOC_017979	down
PC-3p-50929_43	TGGAAGTGTCAGAAATTCTGAGT	up	XLOC_017986	up
aca-miR-338-3p_R+2	TCCAGCATCAGTGATTTTGTTAA	up	XLOC_017986	up
dre-miR-21_1ss23CA	TAGCTTATCAGACTGGTGTTGGA	up	XLOC_017986	up
ola-miR-194-3p_1ss20CT	CCAGTGGAGGTGCTGTTACTTG	up	XLOC_017986	up
ssa-miR-26a-4-3p	CCTATTCTTGATTACTTGTTTC	down	XLOC_017986	up
aca-miR-338-3p_R+2	TCCAGCATCAGTGATTTTGTTAA	up	XLOC_017992	up
ssa-miR-16b-5p_R-1_1ss21TC	TAGCAGCACGTAAATATTGGC	down	XLOC_017992	up
PC-5p-8690_526	GATGTTGAGTATCAAACTGTAT	down	XLOC_018011	down
ssa-miR-1-4-5p	ACATACTTCTTTATATGCCCATA	up	XLOC_018011	down
PC-3p-50929_43	TGGAAGTGTCAGAAATTCTGAGT	up	XLOC_018028	up
PC-5p-45063_62	AAGGATAACTACAACTGTACTT	up	XLOC_018028	up
dre-miR-122	TGGAGTGTGACAATGGTGTTTG	up	XLOC_018028	up
dre-miR-194a_R+2	TGTAACAGCAACTCCATGTGGAT	up	XLOC_018028	up
ssa-miR-7a-5p	TGGAAGACTAGTGATTTTGTTGT	up	XLOC_018028	up
ssa-miR-7a-5p_R+1	TGGAAGACTAGTGATTTTGTTGTT	up	XLOC_018028	up
tni-miR-194_R+1	TGTAACAGCAACTCCATGTGGA	up	XLOC_018028	up
dre-miR-142a-3p_R-1	TGTAGTGTTTCCTACTTTATGG	down	XLOC_018046	down
ola-miR-194-3p_1ss20CT	CCAGTGGAGGTGCTGTTACTTG	up	XLOC_018046	down
ola-miR-199a-3p_L+1	AACAGTAGTCTGCACATTGGTTA	up	XLOC_018046	down
ssa-miR-199a-3p_R+2	ACAGTAGTCTGCACATTGGTTTT	up	XLOC_018046	down
ssa-miR-730a-5p_R-1	TCCTCATTGTGCATGCTGTGT	down	XLOC_018046	down
aca-miR-200b-3p_R+2	TAATACTGCCTGGTAATGATGAAT	up	XLOC_018063	up
ssa-miR-16b-5p_R-1_1ss21TC	TAGCAGCACGTAAATATTGGC	down	XLOC_018063	up
ssa-miR-16b-5p_R-1_1ss21TC	TAGCAGCACGTAAATATTGGC	down	XLOC_018095	up
dre-miR-22a-3p	AAGCTGCCAGCTGAAGAACTGT	up	XLOC_018118	down
ssa-miR-730a-5p_R-1	TCCTCATTGTGCATGCTGTGT	down	XLOC_018118	down
PC-3p-41259_77	TGGCCATTAACTGCTAACCTTC	up	XLOC_018152	down
dre-miR-24_R+2_1	TGGCTCAGTTCAGCAGGAACAGAA	up	XLOC_018172	down
dre-miR-24_R+2_2	TGGCTCAGTTCAGCAGGAACAGTT	up	XLOC_018172	down
dre-miR-24_R+2_1	TGGCTCAGTTCAGCAGGAACAGAA	up	XLOC_018178	down
dre-miR-24_R+2_2	TGGCTCAGTTCAGCAGGAACAGTT	up	XLOC_018178	down
ssa-miR-199a-3p_R+2	ACAGTAGTCTGCACATTGGTTTT	up	XLOC_018178	down
PC-3p-50929_43	TGGAAGTGTCAGAAATTCTGAGT	up	XLOC_018222	up
PC-5p-45063_62	AAGGATAACTACAACTGTACTT	up	XLOC_018222	up
PC-5p-8690_526	GATGTTGAGTATCAAACTGTAT	down	XLOC_018222	up
ola-miR-146a-5p_1ss24TA	TGAGAACTGAATTCCATAGATGGAA	up	XLOC_018222	up
ssa-miR-7a-5p	TGGAAGACTAGTGATTTTGTTGT	up	XLOC_018222	up
ssa-miR-7a-5p_R+1	TGGAAGACTAGTGATTTTGTTGTT	up	XLOC_018222	up
xtr-miR-122_L+1R-1	CTGGAGTGTGACAATGGTGTTTG	up	XLOC_018222	up
PC-5p-27517_164	TACATGCAGAGGTGGAGCAAGA	up	XLOC_018268	up
PC-5p-27517_164	TACATGCAGAGGTGGAGCAAGA	up	XLOC_018298	down
PC-3p-50929_43	TGGAAGTGTCAGAAATTCTGAGT	up	XLOC_018312	up
xtr-miR-122_L+1R-1	CTGGAGTGTGACAATGGTGTTTG	up	XLOC_018369	up
dre-miR-1	TGGAATGTAAAGAAGTATGTAT	up	XLOC_018413	down
dre-miR-122	TGGAGTGTGACAATGGTGTTTG	up	XLOC_018413	down
ssa-miR-206-3p	TGGAATGTAAGGAAGTGTGTGG	up	XLOC_018413	down
ssa-miR-730a-5p_R-1	TCCTCATTGTGCATGCTGTGT	down	XLOC_018413	down
ssc-miR-206	TGGAATGTAAGGAAGTGTGTGA	up	XLOC_018413	down
dre-miR-125b-5p_R+1	TCCCTGAGACCCTAACTTGTGAT	up	XLOC_018422	down

dre-miR-133a-3p_L-1R+1	TTGGTCCCCTTCAACCAGCTGT	up	XLOC_018422	down
sha-miR-125a_R+2	TCCCTGAGACCCTAACTTGTGAAA	up	XLOC_018422	down
ssa-miR-730a-5p_R-1	TCCTCATTGTGCATGCTGTGT	down	XLOC_018422	down
dre-miR-22a-3p	AAGCTGCCAGCTGAAGAACTGT	up	XLOC_018423	up
dre-miR-125b-5p_R+1	TCCCTGAGACCCTAACTTGTGAT	up	XLOC_018470	up
ola-miR-194-3p_1ss20CT	CCAGTGGAGGTGCTGTTACTTG	up	XLOC_018470	up
sha-miR-125a_R+2	TCCCTGAGACCCTAACTTGTGAAA	up	XLOC_018470	up
ssa-miR-125b-5p_R-1	TCCCTGAGACCCTTAACCTGTG	up	XLOC_018470	up
tni-miR-10c	TACCCTGTAGATCCGGATTTGT	up	XLOC_018481	down
PC-3p-41259_77	TGGCCATTAACTGCTAACCTTC	up	XLOC_018488	up
PC-5p-8690_526	GATGTTGAGTATCAAACTGTAT	down	XLOC_018488	up
ola-miR-194-3p_1ss20CT	CCAGTGGAGGTGCTGTTACTTG	up	XLOC_018488	up
ssa-miR-16b-5p_R-1_1ss21TC	TAGCAGCACGTAAATATTGGC	down	XLOC_018488	up
xtr-miR-122_L+1R-1	CTGGAGTGTGACAATGGTGTTTG	up	XLOC_018488	up
PC-3p-11630_419	ATGAGGAAAAGAAGTTAGGAGA	down	XLOC_018493	up
PC-5p-27517_164	TACATGCAGAGGTGGAGCAAGA	up	XLOC_018493	up
dre-let-7d-5p	TGAGGTAGTTGGTTGTATGGTT	up	XLOC_018493	up
dre-miR-140-3p_L-1	ACCACAGGGTAGAACCACGGAC	up	XLOC_018493	up
dre-miR-194a_R+2	TGTAACAGCAACTCCATGTGGAT	up	XLOC_018493	up
mmu-let-7j_1ss8TG	TGAGGTAGTAGTTTGTGCTGTTAT	up	XLOC_018493	up
tni-let-7j_1ss11TG	TGAGGTAGTTGTTTGTACAGTT	up	XLOC_018493	up
tni-miR-194_R+1	TGTAACAGCAACTCCATGTGGA	up	XLOC_018493	up
PC-5p-27517_164	TACATGCAGAGGTGGAGCAAGA	up	XLOC_018523	down
PC-3p-50929_43	TGGAAGTGTCAGAAATTCTGAGT	up	XLOC_018676	up
ssa-miR-16b-5p_R-1_1ss21TC	TAGCAGCACGTAAATATTGGC	down	XLOC_018676	up
ssa-miR-7a-5p	TGGAAGACTAGTGATTTTGTTGT	up	XLOC_018676	up
ssa-miR-7a-5p_R+1	TGGAAGACTAGTGATTTTGTTGTT	up	XLOC_018676	up
PC-3p-50929_43	TGGAAGTGTCAGAAATTCTGAGT	up	XLOC_018678	down
PC-5p-27517_164	TACATGCAGAGGTGGAGCAAGA	up	XLOC_018678	down
aca-miR-338-3p_R+2	TCCAGCATCAGTGATTTTGTTAA	up	XLOC_018678	down
ssa-miR-1338-5p_R+1	AGGACTGTCCAACCTGAGAATG	down	XLOC_018678	down
ssa-miR-26a-4-3p	CCTATTCTTGATTACTTGTTTC	down	XLOC_018678	down
ssa-miR-7132b-3p	TGAGGCGTTTAGAACAAGTTCA	down	XLOC_018678	down
PC-3p-11630_419	ATGAGGAAAAGAAGTTAGGAGA	down	XLOC_018735	down
dre-let-7d-5p	TGAGGTAGTTGGTTGTATGGTT	up	XLOC_018735	down
dre-miR-1	TGGAATGTAAAGAAGTATGTAT	up	XLOC_018735	down
dre-miR-122	TGGAGTGTGACAATGGTGTTTG	up	XLOC_018735	down
mmu-let-7j_1ss8TG	TGAGGTAGTAGTTTGTGCTGTTAT	up	XLOC_018735	down
ola-miR-194-3p_1ss20CT	CCAGTGGAGGTGCTGTTACTTG	up	XLOC_018735	down
ssa-miR-1338-5p_R+1	AGGACTGTCCAACCTGAGAATG	down	XLOC_018735	down
ssa-miR-206-3p	TGGAATGTAAGGAAGTGTGTGG	up	XLOC_018735	down
ssc-miR-206	TGGAATGTAAGGAAGTGTGTGA	up	XLOC_018735	down
tni-let-7j_1ss11TG	TGAGGTAGTTGTTTGTACAGTT	up	XLOC_018735	down
xtr-miR-122_L+1R-1	CTGGAGTGTGACAATGGTGTTTG	up	XLOC_018735	down
PC-5p-8690_526	GATGTTGAGTATCAAACTGTAT	down	XLOC_018745	down
PC-3p-41259_77	TGGCCATTAACTGCTAACCTTC	up	XLOC_018757	up
PC-3p-50929_43	TGGAAGTGTCAGAAATTCTGAGT	up	XLOC_018757	up
ssa-miR-26d-5p_L+1_1ss13TC	CTTCAAGTAATCCAGGATAGGCT	up	XLOC_018757	up
aca-miR-200b-3p_R+2	TAATACTGCCTGGTAATGATGAAT	up	XLOC_018775	up
dre-miR-133b-3p_R-1	TTTGGTCCCCTTCAACCAGCT	up	XLOC_018775	up
oha-miR-133b-3p	TTTGGTCCCCTTCAACCAGCTAT	up	XLOC_018775	up
aca-miR-338-3p_R+2	TCCAGCATCAGTGATTTTGTTAA	up	XLOC_018779	up
ola-miR-146a-5p_1ss24TA	TGAGAACTGAATTCCATAGATGGAA	up	XLOC_018779	up
ssa-miR-199a-3p_R+2	ACAGTAGTCTGCACATTGGTTTT	up	XLOC_018779	up

PC-3p-41259_77	TGGCCATTAACTGCTAACCTTC	up	XLOC_018799	up
dre-miR-133b-3p_R-1	TTTGGTCCCCTTCAACCAGCT	up	XLOC_018799	up
dre-miR-194a_R+2	TGTAACAGCAACTCCATGTGGAT	up	XLOC_018799	up
oha-miR-133b-3p	TTTGGTCCCCTTCAACCAGCTAT	up	XLOC_018799	up
tni-miR-194_R+1	TGTAACAGCAACTCCATGTGGA	up	XLOC_018799	up
PC-3p-50929_43	TGGAAGTGTCAGAAATTCTGAGT	up	XLOC_018819	up
ssa-miR-26a-4-3p	CCTATTCTTGATTACTTGTTTC	down	XLOC_018819	up
ssa-miR-16b-5p_R-1_1ss21TC	TAGCAGCACGTAAATATTGGC	down	XLOC_018822	up
PC-5p-8690_526	GATGTTGAGTATCAAACTGTAT	down	XLOC_018867	down
dre-miR-24_R+2_1	TGGCTCAGTTCAGCAGGAACAGAA	up	XLOC_018904	up
dre-miR-24_R+2_2	TGGCTCAGTTCAGCAGGAACAGTT	up	XLOC_018904	up
dre-let-7d-5p	TGAGGTAGTTGGTTGTATGGTT	up	XLOC_018933	down
mmu-let-7j_1ss8TG	TGAGGTAGTAGTTTGTGCTGTTAT	up	XLOC_018933	down
tni-let-7j_1ss11TG	TGAGGTAGTTGTTTGTACAGTT	up	XLOC_018933	down
aca-miR-200b-3p_R+2	TAATACTGCCTGGTAATGATGAAT	up	XLOC_018971	down
PC-5p-27517_164	TACATGCAGAGGTGGAGCAAGA	up	XLOC_018983	down
dre-miR-24_R+2_1	TGGCTCAGTTCAGCAGGAACAGAA	up	XLOC_018983	down
dre-miR-24_R+2_2	TGGCTCAGTTCAGCAGGAACAGTT	up	XLOC_018983	down
rno-miR-122-5p_L+3	ATCTGGAGTGTGACAATGGTGTTTG	up	XLOC_018983	down
ssa-miR-16b-5p_R-1_1ss21TC	TAGCAGCACGTAAATATTGGC	down	XLOC_018983	down
ssa-miR-7132a-5p_R+1	GACTTGGTCAAAGCTCCTCAGTT	down	XLOC_018983	down
ssa-miR-7132b-5p	GACTTGGTCAAAGCTCCTCAGC	down	XLOC_018983	down
ssa-miR-730a-5p_R-1	TCCTCATTGTGCATGCTGTGT	down	XLOC_018983	down
ssa-miR-16b-5p_R-1_1ss21TC	TAGCAGCACGTAAATATTGGC	down	XLOC_019044	up
dre-miR-140-3p_L-1	ACCACAGGGTAGAACCACGGAC	up	XLOC_019071	up
ssa-miR-16b-5p_R-1_1ss21TC	TAGCAGCACGTAAATATTGGC	down	XLOC_019071	up
aca-miR-200b-3p_R+2	TAATACTGCCTGGTAATGATGAAT	up	XLOC_019090	up
dre-miR-122	TGGAGTGTGACAATGGTGTTTG	up	XLOC_019090	up
xtr-miR-122_L+1R-1	CTGGAGTGTGACAATGGTGTTTG	up	XLOC_019090	up
aca-miR-338-3p_R+2	TCCAGCATCAGTGATTTTGTTAA	up	XLOC_019099	down
aca-miR-338-3p_R+2	TCCAGCATCAGTGATTTTGTTAA	up	XLOC_019110	down
PC-5p-8690_526	GATGTTGAGTATCAAACTGTAT	down	XLOC_019112	up
dre-miR-140-3p_L-1	ACCACAGGGTAGAACCACGGAC	up	XLOC_019112	up
ssa-miR-1-4-5p	ACATACTTCTTTATATGCCCATA	up	XLOC_019112	up
dre-miR-133a-3p_L-1R+1	TTGGTCCCCTTCAACCAGCTGT	up	XLOC_019123	up
ola-mir-100-2-p3	CAAGCTCGTATCTATAGGTATG	down	XLOC_019123	up
dre-miR-125b-5p_R+1	TCCCTGAGACCCTAACTTGTGAT	up	XLOC_019130	up
ola-miR-194-3p_1ss20CT	CCAGTGGAGGTGCTGTTACTTG	up	XLOC_019130	up
sha-miR-125a_R+2	TCCCTGAGACCCTAACTTGTGAAA	up	XLOC_019130	up
ssa-miR-125b-5p_R-1	TCCCTGAGACCCTTAACCTGTG	up	XLOC_019130	up
ssa-miR-730a-5p_R-1	TCCTCATTGTGCATGCTGTGT	down	XLOC_019130	up
PC-5p-27517_164	TACATGCAGAGGTGGAGCAAGA	up	XLOC_019198	up
ssa-miR-26d-5p_L+1_1ss13TC	CTTCAAGTAATCCAGGATAGGCT	up	XLOC_019198	up
ssa-miR-730a-5p_R-1	TCCTCATTGTGCATGCTGTGT	down	XLOC_019198	up
tni-miR-10c	TACCCTGTAGATCCGGATTTGT	up	XLOC_019198	up
PC-3p-50929_43	TGGAAGTGTCAGAAATTCTGAGT	up	XLOC_019371	up
rno-miR-122-5p_L+3	ATCTGGAGTGTGACAATGGTGTTTG	up	XLOC_019371	up
ssa-miR-16b-5p_R-1_1ss21TC	TAGCAGCACGTAAATATTGGC	down	XLOC_019371	up
dre-miR-194a_R+2	TGTAACAGCAACTCCATGTGGAT	up	XLOC_019410	down
ssa-miR-16b-5p_R-1_1ss21TC	TAGCAGCACGTAAATATTGGC	down	XLOC_019410	down
tni-miR-194_R+1	TGTAACAGCAACTCCATGTGGA	up	XLOC_019410	down
ssa-miR-7a-5p	TGGAAGACTAGTGATTTTGTTGT	up	XLOC_019423	up
ssa-miR-7a-5p_R+1	TGGAAGACTAGTGATTTTGTTGTT	up	XLOC_019423	up
dre-miR-125b-5p_R+1	TCCCTGAGACCCTAACTTGTGAT	up	XLOC_019470	down

ola-miR-199a-3p_L+1	AACAGTAGTCTGCACATTGGTTA	up	XLOC_019470	down
sha-miR-125a_R+2	TCCCTGAGACCCTAACTTGTGAAA	up	XLOC_019470	down
ssa-miR-125b-5p_R-1	TCCCTGAGACCCTTAACCTGTG	up	XLOC_019470	down
tni-miR-10c	TACCCTGTAGATCCGGATTTGT	up	XLOC_019470	down
ssa-miR-1-4-5p	ACATACTTCTTTATATGCCCATA	up	XLOC_019474	down
tni-miR-10c	TACCCTGTAGATCCGGATTTGT	up	XLOC_019474	down
aca-miR-338-3p_R+2	TCCAGCATCAGTGATTTTGTTAA	up	XLOC_019580	up
ssa-miR-16b-5p_R-1_1ss21TC	TAGCAGCACGTAAATATTGGC	down	XLOC_019580	up
ssa-miR-730a-5p_R-1	TCCTCATTGTGCATGCTGTGT	down	XLOC_019580	up
PC-3p-11630_419	ATGAGGAAAAGAAGTTAGGAGA	down	XLOC_019659	down
PC-5p-27517_164	TACATGCAGAGGTGGAGCAAGA	up	XLOC_019659	down
ssa-miR-730a-5p_R-1	TCCTCATTGTGCATGCTGTGT	down	XLOC_019659	down
PC-3p-11630_419	ATGAGGAAAAGAAGTTAGGAGA	down	XLOC_019664	up
dre-miR-125b-5p_R+1	TCCCTGAGACCCTAACTTGTGAT	up	XLOC_019670	up
dre-miR-22a-3p	AAGCTGCCAGCTGAAGAACTGT	up	XLOC_019670	up
sha-miR-125a_R+2	TCCCTGAGACCCTAACTTGTGAAA	up	XLOC_019670	up
ssa-miR-125b-5p_R-1	TCCCTGAGACCCTTAACCTGTG	up	XLOC_019670	up
dre-miR-22a-3p	AAGCTGCCAGCTGAAGAACTGT	up	XLOC_019708	down
PC-3p-11630_419	ATGAGGAAAAGAAGTTAGGAGA	down	XLOC_019710	down
ssa-miR-16b-5p_R-1_1ss21TC	TAGCAGCACGTAAATATTGGC	down	XLOC_019714	up
dre-miR-194a_R+2	TGTAACAGCAACTCCATGTGGAT	up	XLOC_019745	down
tni-miR-194_R+1	TGTAACAGCAACTCCATGTGGA	up	XLOC_019745	down
dre-miR-194a_R+2	TGTAACAGCAACTCCATGTGGAT	up	XLOC_019760	down
tni-miR-194_R+1	TGTAACAGCAACTCCATGTGGA	up	XLOC_019760	down
ola-miR-462_L-1R+4	TAACGGAACCCATAATGCAGCT	down	XLOC_019782	up
ola-mir-100-2-p3	CAAGCTCGTATCTATAGGTATG	down	XLOC_019782	up
ssa-miR-1338-5p_R+1	AGGACTGTCCAACCTGAGAATG	down	XLOC_019782	up
ssa-miR-1-4-5p	ACATACTTCTTTATATGCCCATA	up	XLOC_019786	down
dre-miR-125b-5p_R+1	TCCCTGAGACCCTAACTTGTGAT	up	XLOC_019789	down
dre-miR-21_1ss23CA	TAGCTTATCAGACTGGTGTTGGA	up	XLOC_019789	down
sha-miR-125a_R+2	TCCCTGAGACCCTAACTTGTGAAA	up	XLOC_019789	down
ssa-miR-125b-5p_R-1	TCCCTGAGACCCTTAACCTGTG	up	XLOC_019789	down
aca-miR-338-3p_R+2	TCCAGCATCAGTGATTTTGTTAA	up	XLOC_019822	down
dre-miR-142a-3p_R-1	TGTAGTGTTTCCTACTTTATGG	down	XLOC_019822	down
ssa-miR-26a-4-3p	CCTATTCTTGATTACTTGTTTC	down	XLOC_019843	down
ssa-miR-16b-5p_R-1_1ss21TC	TAGCAGCACGTAAATATTGGC	down	XLOC_019873	down
ssa-miR-730a-5p_R-1	TCCTCATTGTGCATGCTGTGT	down	XLOC_019873	down
dre-let-7d-5p	TGAGGTAGTTGGTTGTATGGTT	up	XLOC_019902	down
mmu-let-7j_1ss8TG	TGAGGTAGTAGTTTGTGCTGTTAT	up	XLOC_019902	down
tni-let-7j_1ss11TG	TGAGGTAGTTGTTTGTACAGTT	up	XLOC_019902	down
PC-5p-8690_526	GATGTTGAGTATCAAACTGTAT	down	XLOC_019924	up
ssa-miR-26d-5p_L+1_1ss13TC	CTTCAAGTAATCCAGGATAGGCT	up	XLOC_020010	down
PC-3p-50929_43	TGGAAGTGTCAGAAATTCTGAGT	up	XLOC_020027	up
ola-miR-194-3p_1ss20CT	CCAGTGGAGGTGCTGTTACTTG	up	XLOC_020036	down
dre-miR-122	TGGAGTGTGACAATGGTGTTTG	up	XLOC_020044	up
mmu-let-7j_1ss8TG	TGAGGTAGTAGTTTGTGCTGTTAT	up	XLOC_020044	up
xtr-miR-122_L+1R-1	CTGGAGTGTGACAATGGTGTTTG	up	XLOC_020044	up
dre-miR-122	TGGAGTGTGACAATGGTGTTTG	up	XLOC_020060	down
ola-miR-194-3p_1ss20CT	CCAGTGGAGGTGCTGTTACTTG	up	XLOC_020060	down
ssa-miR-16b-5p_R-1_1ss21TC	TAGCAGCACGTAAATATTGGC	down	XLOC_020060	down
dre-miR-133b-3p_R-1	TTTGGTCCCCTTCAACCAGCT	up	XLOC_020088	up
dre-miR-21_1ss23CA	TAGCTTATCAGACTGGTGTTGGA	up	XLOC_020088	up
oha-miR-133b-3p	TTTGGTCCCCTTCAACCAGCTAT	up	XLOC_020088	up
PC-3p-11630_419	ATGAGGAAAAGAAGTTAGGAGA	down	XLOC_020110	down

dre-miR-1	TGGAATGTAAAGAAGTATGTAT	up	XLOC_020110	down
ssa-miR-206-3p	TGGAATGTAAGGAAGTGTGTGG	up	XLOC_020110	down
ssc-miR-206	TGGAATGTAAGGAAGTGTGTGA	up	XLOC_020110	down
tni-miR-10c	TACCCTGTAGATCCGGATTTGT	up	XLOC_020110	down
aca-miR-200b-3p_R+2	TAATACTGCCTGGTAATGATGAAT	up	XLOC_020111	down
ssa-miR-16b-5p_R-1_1ss21TC	TAGCAGCACGTAAATATTGGC	down	XLOC_020111	down
ssa-miR-730a-5p_R-1	TCCTCATTGTGCATGCTGTGT	down	XLOC_020127	up
aca-miR-338-3p_R+2	TCCAGCATCAGTGATTTTGTTAA	up	XLOC_020137	up
dre-miR-194a_R+2	TGTAACAGCAACTCCATGTGGAT	up	XLOC_020204	down
ssa-miR-26d-5p_L+1_1ss13TC	CTTCAAGTAATCCAGGATAGGCT	up	XLOC_020204	down
ssa-miR-730a-5p_R-1	TCCTCATTGTGCATGCTGTGT	down	XLOC_020204	down
tni-miR-194_R+1	TGTAACAGCAACTCCATGTGGA	up	XLOC_020204	down
aca-miR-200b-3p_R+2	TAATACTGCCTGGTAATGATGAAT	up	XLOC_020210	up
dre-miR-21_1ss23CA	TAGCTTATCAGACTGGTGTTGGA	up	XLOC_020210	up
ssa-miR-16b-5p_R-1_1ss21TC	TAGCAGCACGTAAATATTGGC	down	XLOC_020210	up
dre-miR-140-3p_L-1	ACCACAGGGTAGAACCACGGAC	up	XLOC_020249	down
dre-miR-22a-3p	AAGCTGCCAGCTGAAGAACTGT	up	XLOC_020283	down
ssa-miR-26a-4-3p	CCTATTCTTGATTACTTGTTTC	down	XLOC_020283	down
ola-miR-146a-5p_1ss24TA	TGAGAACTGAATTCCATAGATGGAA	up	XLOC_020299	down
dre-miR-125b-5p_R+1	TCCCTGAGACCCTAACTTGTGAT	up	XLOC_020364	up
dre-miR-140-3p_L-1	ACCACAGGGTAGAACCACGGAC	up	XLOC_020364	up
sha-miR-125a_R+2	TCCCTGAGACCCTAACTTGTGAAA	up	XLOC_020364	up
ssa-miR-125b-5p_R-1	TCCCTGAGACCCTTAACCTGTG	up	XLOC_020364	up
PC-3p-11630_419	ATGAGGAAAAGAAGTTAGGAGA	down	XLOC_020433	up
ola-miR-199a-3p_L+1	AACAGTAGTCTGCACATTGGTTA	up	XLOC_020504	down
ssa-miR-1338-5p_R+1	AGGACTGTCCAACCTGAGAATG	down	XLOC_020504	down
ssa-miR-199a-3p_R+2	ACAGTAGTCTGCACATTGGTTTT	up	XLOC_020504	down
ola-miR-194-3p_1ss20CT	CCAGTGGAGGTGCTGTTACTTG	up	XLOC_020525	down
ssa-miR-16b-5p_R-1_1ss21TC	TAGCAGCACGTAAATATTGGC	down	XLOC_020556	down
ssa-miR-7132a-5p_R+1	GACTTGGTCAAAGCTCCTCAGTT	down	XLOC_020625	down
ssa-miR-7132b-5p	GACTTGGTCAAAGCTCCTCAGC	down	XLOC_020625	down
ssa-miR-1338-5p_R+1	AGGACTGTCCAACCTGAGAATG	down	XLOC_020627	down
dre-miR-1	TGGAATGTAAAGAAGTATGTAT	up	XLOC_020634	up
dre-miR-125b-5p_R+1	TCCCTGAGACCCTAACTTGTGAT	up	XLOC_020634	up
sha-miR-125a_R+2	TCCCTGAGACCCTAACTTGTGAAA	up	XLOC_020634	up
ssa-miR-125b-5p_R-1	TCCCTGAGACCCTTAACCTGTG	up	XLOC_020634	up
ssa-miR-206-3p	TGGAATGTAAGGAAGTGTGTGG	up	XLOC_020634	up
ssa-miR-26d-5p_L+1_1ss13TC	CTTCAAGTAATCCAGGATAGGCT	up	XLOC_020634	up
ssc-miR-206	TGGAATGTAAGGAAGTGTGTGA	up	XLOC_020634	up
PC-5p-27517_164	TACATGCAGAGGTGGAGCAAGA	up	XLOC_020649	up
dre-miR-194a_R+2	TGTAACAGCAACTCCATGTGGAT	up	XLOC_020649	up
dre-miR-24_R+2_1	TGGCTCAGTTCAGCAGGAACAGAA	up	XLOC_020649	up
dre-miR-24_R+2_2	TGGCTCAGTTCAGCAGGAACAGTT	up	XLOC_020649	up
ola-miR-199a-3p_L+1	AACAGTAGTCTGCACATTGGTTA	up	XLOC_020649	up
ssa-miR-16b-5p_R-1_1ss21TC	TAGCAGCACGTAAATATTGGC	down	XLOC_020649	up
tni-miR-194_R+1	TGTAACAGCAACTCCATGTGGA	up	XLOC_020649	up
ccr-miR-99_R+3	AACCCGTAGATCCGATCTTGTGAA	up	XLOC_020653	down
tni-miR-10c	TACCCTGTAGATCCGGATTTGT	up	XLOC_020680	down
ola-miR-194-3p_1ss20CT	CCAGTGGAGGTGCTGTTACTTG	up	XLOC_020758	down
ssa-miR-730a-5p_R-1	TCCTCATTGTGCATGCTGTGT	down	XLOC_020796	up
ssa-miR-7a-5p	TGGAAGACTAGTGATTTTGTTGT	up	XLOC_020796	up
ssa-miR-7a-5p_R+1	TGGAAGACTAGTGATTTTGTTGTT	up	XLOC_020796	up
ola-miR-199a-3p_L+1	AACAGTAGTCTGCACATTGGTTA	up	XLOC_020919	up
ssa-miR-7a-5p	TGGAAGACTAGTGATTTTGTTGT	up	XLOC_020919	up

ssa-miR-7a-5p_R+1	TGGAAGACTAGTGATTTTGTTGTT	up	XLOC_020919	up
ssa-mir-15c-2-p3_1ss11CA	TGCGAACCATAATTTGCTGCTT	down	XLOC_020919	up
xtr-miR-122_L+1R-1	CTGGAGTGTGACAATGGTGTTTG	up	XLOC_020919	up
aca-miR-200b-3p_R+2	TAATACTGCCTGGTAATGATGAAT	up	XLOC_020928	up
aca-miR-338-3p_R+2	TCCAGCATCAGTGATTTTGTTAA	up	XLOC_020928	up
PC-3p-50929_43	TGGAAGTGTCAGAAATTCTGAGT	up	XLOC_020931	down
PC-5p-27517_164	TACATGCAGAGGTGGAGCAAGA	up	XLOC_020931	down
aca-miR-200b-3p_R+2	TAATACTGCCTGGTAATGATGAAT	up	XLOC_020931	down
ola-miR-194-3p_1ss20CT	CCAGTGGAGGTGCTGTTACTTG	up	XLOC_020931	down
xtr-miR-122_L+1R-1	CTGGAGTGTGACAATGGTGTTTG	up	XLOC_020931	down
dre-miR-21_1ss23CA	TAGCTTATCAGACTGGTGTTGGA	up	XLOC_020973	down
aca-miR-200b-3p_R+2	TAATACTGCCTGGTAATGATGAAT	up	XLOC_020992	up
PC-3p-11630_419	ATGAGGAAAAGAAGTTAGGAGA	down	XLOC_021020	up
PC-3p-50929_43	TGGAAGTGTCAGAAATTCTGAGT	up	XLOC_021020	up
aca-miR-200b-3p_R+2	TAATACTGCCTGGTAATGATGAAT	up	XLOC_021020	up
ssa-miR-730a-5p_R-1	TCCTCATTGTGCATGCTGTGT	down	XLOC_021020	up
ssa-miR-26d-5p_L+1_1ss13TC	CTTCAAGTAATCCAGGATAGGCT	up	XLOC_021221	down
ola-miR-146a-5p_1ss24TA	TGAGAACTGAATTCCATAGATGGAA	up	XLOC_021229	up
dre-miR-142a-5p	CATAAAGTAGAAAGCACTACT	down	XLOC_021240	up
ola-miR-199a-3p_L+1	AACAGTAGTCTGCACATTGGTTA	up	XLOC_021240	up
ssa-miR-16b-5p_R-1_1ss21TC	TAGCAGCACGTAAATATTGGC	down	XLOC_021240	up
dre-miR-125b-5p_R+1	TCCCTGAGACCCTAACTTGTGAT	up	XLOC_021305	up
sha-miR-125a_R+2	TCCCTGAGACCCTAACTTGTGAAA	up	XLOC_021305	up
ssa-miR-125b-5p_R-1	TCCCTGAGACCCTTAACCTGTG	up	XLOC_021305	up
PC-5p-27517_164	TACATGCAGAGGTGGAGCAAGA	up	XLOC_021312	down
ola-miR-199a-3p_L+1	AACAGTAGTCTGCACATTGGTTA	up	XLOC_021312	down
PC-5p-8690_526	GATGTTGAGTATCAAACTGTAT	down	XLOC_021319	up
PC-3p-50929_43	TGGAAGTGTCAGAAATTCTGAGT	up	XLOC_021347	up
rno-miR-122-5p_L+3	ATCTGGAGTGTGACAATGGTGTTTG	up	XLOC_021347	up
aca-miR-200b-3p_R+2	TAATACTGCCTGGTAATGATGAAT	up	XLOC_021403	up
dre-miR-142a-3p_R-1	TGTAGTGTTTCCTACTTTATGG	down	XLOC_021403	up
ssa-miR-16b-5p_R-1_1ss21TC	TAGCAGCACGTAAATATTGGC	down	XLOC_021403	up
ssa-miR-7132a-5p_R+1	GACTTGGTCAAAGCTCCTCAGTT	down	XLOC_021403	up
ssa-miR-7132b-5p	GACTTGGTCAAAGCTCCTCAGC	down	XLOC_021403	up
ssa-miR-7a-5p	TGGAAGACTAGTGATTTTGTTGT	up	XLOC_021426	down
ssa-miR-7a-5p_R+1	TGGAAGACTAGTGATTTTGTTGTT	up	XLOC_021426	down
dre-miR-125b-5p_R+1	TCCCTGAGACCCTAACTTGTGAT	up	XLOC_021480	up
sha-miR-125a_R+2	TCCCTGAGACCCTAACTTGTGAAA	up	XLOC_021480	up
ssa-miR-125b-5p_R-1	TCCCTGAGACCCTTAACCTGTG	up	XLOC_021480	up
ssa-miR-730a-5p_R-1	TCCTCATTGTGCATGCTGTGT	down	XLOC_021480	up
ssa-miR-7a-5p	TGGAAGACTAGTGATTTTGTTGT	up	XLOC_021480	up
ssa-miR-7a-5p_R+1	TGGAAGACTAGTGATTTTGTTGTT	up	XLOC_021480	up
dre-miR-194a_R+2	TGTAACAGCAACTCCATGTGGAT	up	XLOC_021524	down
ola-miR-146a-5p_1ss24TA	TGAGAACTGAATTCCATAGATGGAA	up	XLOC_021524	down
tni-miR-194_R+1	TGTAACAGCAACTCCATGTGGA	up	XLOC_021524	down
dre-miR-194a_R+2	TGTAACAGCAACTCCATGTGGAT	up	XLOC_021550	down
tni-miR-194_R+1	TGTAACAGCAACTCCATGTGGA	up	XLOC_021550	down
PC-5p-27517_164	TACATGCAGAGGTGGAGCAAGA	up	XLOC_021608	down
aca-miR-200b-3p_R+2	TAATACTGCCTGGTAATGATGAAT	up	XLOC_021608	down
dre-let-7d-5p	TGAGGTAGTTGGTTGTATGGTT	up	XLOC_021608	down
dre-miR-22a-3p	AAGCTGCCAGCTGAAGAACTGT	up	XLOC_021608	down
dre-miR-24_R+2_1	TGGCTCAGTTCAGCAGGAACAGAA	up	XLOC_021608	down
dre-miR-24_R+2_2	TGGCTCAGTTCAGCAGGAACAGTT	up	XLOC_021608	down
mmu-let-7j_1ss8TG	TGAGGTAGTAGTTTGTGCTGTTAT	up	XLOC_021608	down

ssa-miR-199a-3p_R+2	ACAGTAGTCTGCACATTGGTTTT	up	XLOC_021608	down
tni-let-7j_1ss11TG	TGAGGTAGTTGTTTGTACAGTT	up	XLOC_021608	down
ola-miR-194-3p_1ss20CT	CCAGTGGAGGTGCTGTTACTTG	up	XLOC_021626	down
ola-miR-199a-3p_L+1	AACAGTAGTCTGCACATTGGTTA	up	XLOC_021626	down
ssa-miR-199a-3p_R+2	ACAGTAGTCTGCACATTGGTTTT	up	XLOC_021626	down
ssa-miR-7132a-5p_R+1	GACTTGGTCAAAGCTCCTCAGTT	down	XLOC_021679	down
ssa-miR-7132b-5p	GACTTGGTCAAAGCTCCTCAGC	down	XLOC_021679	down
ssa-miR-16b-5p_R-1_1ss21TC	TAGCAGCACGTAAATATTGGC	down	XLOC_021716	down
ssa-miR-1-4-5p	ACATACTTCTTTATATGCCCATA	up	XLOC_021735	down
PC-3p-50929_43	TGGAAGTGTCAGAAATTCTGAGT	up	XLOC_021777	down
ssa-miR-16b-5p_R-1_1ss21TC	TAGCAGCACGTAAATATTGGC	down	XLOC_021777	down
ssa-miR-730a-5p_R-1	TCCTCATTGTGCATGCTGTGT	down	XLOC_021777	down
ssa-miR-7132b-3p	TGAGGCGTTTAGAACAAGTTCA	down	XLOC_021823	up
ssa-miR-16b-5p_R-1_1ss21TC	TAGCAGCACGTAAATATTGGC	down	XLOC_021827	up
ssa-miR-26d-5p_L+1_1ss13TC	CTTCAAGTAATCCAGGATAGGCT	up	XLOC_021827	up
xtr-miR-122_L+1R-1	CTGGAGTGTGACAATGGTGTTTG	up	XLOC_021827	up
ola-miR-194-3p_1ss20CT	CCAGTGGAGGTGCTGTTACTTG	up	XLOC_021840	up
ccr-miR-99_R+3	AACCCGTAGATCCGATCTTGTGAA	up	XLOC_021912	down
ssa-miR-7a-5p	TGGAAGACTAGTGATTTTGTTGT	up	XLOC_021946	up
ssa-miR-7a-5p_R+1	TGGAAGACTAGTGATTTTGTTGTT	up	XLOC_021946	up
ola-miR-146a-5p_1ss24TA	TGAGAACTGAATTCCATAGATGGAA	up	XLOC_022011	down
ola-miR-199a-3p_L+1	AACAGTAGTCTGCACATTGGTTA	up	XLOC_022031	down
ssa-miR-199a-3p_R+2	ACAGTAGTCTGCACATTGGTTTT	up	XLOC_022031	down
dre-miR-133b-3p_R-1	TTTGGTCCCCTTCAACCAGCT	up	XLOC_022097	down
oha-miR-133b-3p	TTTGGTCCCCTTCAACCAGCTAT	up	XLOC_022097	down
ssa-miR-7132a-5p_R+1	GACTTGGTCAAAGCTCCTCAGTT	down	XLOC_022097	down
ssa-miR-7132b-5p	GACTTGGTCAAAGCTCCTCAGC	down	XLOC_022097	down
PC-5p-45063_62	AAGGATAACTACAACTGTACTT	up	XLOC_022189	up
aca-miR-200b-3p_R+2	TAATACTGCCTGGTAATGATGAAT	up	XLOC_022189	up
aca-miR-338-3p_R+2	TCCAGCATCAGTGATTTTGTTAA	up	XLOC_022189	up
dre-miR-140-3p_L-1	ACCACAGGGTAGAACCACGGAC	up	XLOC_022189	up
ssa-miR-16b-5p_R-1_1ss21TC	TAGCAGCACGTAAATATTGGC	down	XLOC_022189	up
PC-3p-50929_43	TGGAAGTGTCAGAAATTCTGAGT	up	XLOC_022200	up
PC-3p-50929_43	TGGAAGTGTCAGAAATTCTGAGT	up	XLOC_022202	up
dre-miR-133a-3p_L-1R+1	TTGGTCCCCTTCAACCAGCTGT	up	XLOC_022202	up
dre-miR-194a_R+2	TGTAACAGCAACTCCATGTGGAT	up	XLOC_022273	up
ola-miR-146a-5p_1ss24TA	TGAGAACTGAATTCCATAGATGGAA	up	XLOC_022273	up
ssa-miR-1338-5p_R+1	AGGACTGTCCAACCTGAGAATG	down	XLOC_022273	up
tni-miR-194_R+1	TGTAACAGCAACTCCATGTGGA	up	XLOC_022273	up
PC-3p-50929_43	TGGAAGTGTCAGAAATTCTGAGT	up	XLOC_022279	down
ssa-miR-199a-3p_R+2	ACAGTAGTCTGCACATTGGTTTT	up	XLOC_022279	down
dre-miR-125b-5p_R+1	TCCCTGAGACCCTAACTTGTGAT	up	XLOC_022286	up
sha-miR-125a_R+2	TCCCTGAGACCCTAACTTGTGAAA	up	XLOC_022286	up
ssa-miR-125b-5p_R-1	TCCCTGAGACCCTTAACCTGTG	up	XLOC_022286	up
PC-5p-27517_164	TACATGCAGAGGTGGAGCAAGA	up	XLOC_022322	up
ssa-miR-7a-5p	TGGAAGACTAGTGATTTTGTTGT	up	XLOC_022339	down
ssa-miR-7a-5p_R+1	TGGAAGACTAGTGATTTTGTTGTT	up	XLOC_022339	down
PC-3p-41259_77	TGGCCATTAACTGCTAACCTTC	up	XLOC_022351	up
PC-5p-27517_164	TACATGCAGAGGTGGAGCAAGA	up	XLOC_022351	up
aca-miR-338-3p_R+2	TCCAGCATCAGTGATTTTGTTAA	up	XLOC_022351	up
dre-miR-194a_R+2	TGTAACAGCAACTCCATGTGGAT	up	XLOC_022351	up
dre-miR-24_R+2_1	TGGCTCAGTTCAGCAGGAACAGAA	up	XLOC_022351	up
dre-miR-24_R+2_2	TGGCTCAGTTCAGCAGGAACAGTT	up	XLOC_022351	up
ssa-miR-26a-4-3p	CCTATTCTTGATTACTTGTTTC	down	XLOC_022351	up

tni-miR-194_R+1	TGTAACAGCAACTCCATGTGGA	up	XLOC_022351	up
PC-5p-8690_526	GATGTTGAGTATCAAACTGTAT	down	XLOC_022404	down
dre-miR-194a_R+2	TGTAACAGCAACTCCATGTGGAT	up	XLOC_022404	down
ssa-miR-1338-5p_R+1	AGGACTGTCCAACCTGAGAATG	down	XLOC_022404	down
tni-miR-194_R+1	TGTAACAGCAACTCCATGTGGA	up	XLOC_022404	down
ola-miR-194-3p_1ss20CT	CCAGTGGAGGTGCTGTTACTTG	up	XLOC_022417	up
ssa-miR-7a-5p	TGGAAGACTAGTGATTTTGTTGT	up	XLOC_022417	up
ssa-miR-7a-5p_R+1	TGGAAGACTAGTGATTTTGTTGTT	up	XLOC_022417	up
dre-miR-125b-5p_R+1	TCCCTGAGACCCTAACTTGTGAT	up	XLOC_022459	up
sha-miR-125a_R+2	TCCCTGAGACCCTAACTTGTGAAA	up	XLOC_022459	up
ssa-miR-125b-5p_R-1	TCCCTGAGACCCTTAACCTGTG	up	XLOC_022459	up
ssa-miR-7132b-3p	TGAGGCGTTTAGAACAAGTTCA	down	XLOC_022459	up
aca-miR-200b-3p_R+2	TAATACTGCCTGGTAATGATGAAT	up	XLOC_022484	up
dre-miR-125b-5p_R+1	TCCCTGAGACCCTAACTTGTGAT	up	XLOC_022484	up
sha-miR-125a_R+2	TCCCTGAGACCCTAACTTGTGAAA	up	XLOC_022484	up
ssa-miR-125b-5p_R-1	TCCCTGAGACCCTTAACCTGTG	up	XLOC_022484	up
PC-5p-27517_164	TACATGCAGAGGTGGAGCAAGA	up	XLOC_022533	down
PC-5p-45063_62	AAGGATAACTACAACTGTACTT	up	XLOC_022533	down
PC-5p-8690_526	GATGTTGAGTATCAAACTGTAT	down	XLOC_022551	up
dre-miR-24_R+2_1	TGGCTCAGTTCAGCAGGAACAGAA	up	XLOC_022551	up
dre-miR-24_R+2_2	TGGCTCAGTTCAGCAGGAACAGTT	up	XLOC_022551	up
rno-miR-122-5p_L+3	ATCTGGAGTGTGACAATGGTGTTTG	up	XLOC_022551	up
ola-miR-194-3p_1ss20CT	CCAGTGGAGGTGCTGTTACTTG	up	XLOC_022566	down
PC-5p-45063_62	AAGGATAACTACAACTGTACTT	up	XLOC_022706	up
aca-miR-200b-3p_R+2	TAATACTGCCTGGTAATGATGAAT	up	XLOC_022706	up
dre-miR-140-3p_L-1	ACCACAGGGTAGAACCACGGAC	up	XLOC_022706	up
ola-mir-100-2-p3	CAAGCTCGTATCTATAGGTATG	down	XLOC_022706	up
ssa-miR-7132a-5p_R+1	GACTTGGTCAAAGCTCCTCAGTT	down	XLOC_022706	up
ssa-miR-7132b-3p	TGAGGCGTTTAGAACAAGTTCA	down	XLOC_022706	up
ssa-miR-7132b-5p	GACTTGGTCAAAGCTCCTCAGC	down	XLOC_022706	up
PC-5p-45063_62	AAGGATAACTACAACTGTACTT	up	XLOC_022709	up
dre-miR-194a_R+2	TGTAACAGCAACTCCATGTGGAT	up	XLOC_022709	up
dre-miR-21_1ss23CA	TAGCTTATCAGACTGGTGTTGGA	up	XLOC_022709	up
ola-miR-194-3p_1ss20CT	CCAGTGGAGGTGCTGTTACTTG	up	XLOC_022709	up
tni-miR-194_R+1	TGTAACAGCAACTCCATGTGGA	up	XLOC_022709	up
dre-miR-133a-3p_L-1R+1	TTGGTCCCCTTCAACCAGCTGT	up	XLOC_022740	down
dre-miR-133b-3p_R-1	TTTGGTCCCCTTCAACCAGCT	up	XLOC_022740	down
dre-miR-194a_R+2	TGTAACAGCAACTCCATGTGGAT	up	XLOC_022740	down
oha-miR-133b-3p	TTTGGTCCCCTTCAACCAGCTAT	up	XLOC_022740	down
ssa-miR-1-4-5p	ACATACTTCTTTATATGCCCATA	up	XLOC_022740	down
tni-miR-194_R+1	TGTAACAGCAACTCCATGTGGA	up	XLOC_022740	down
PC-3p-41259_77	TGGCCATTAACTGCTAACCTTC	up	XLOC_022858	up
dre-miR-122	TGGAGTGTGACAATGGTGTTTG	up	XLOC_022858	up
dre-miR-22a-3p	AAGCTGCCAGCTGAAGAACTGT	up	XLOC_022858	up
ssa-miR-7a-5p	TGGAAGACTAGTGATTTTGTTGT	up	XLOC_022858	up
ssa-miR-7a-5p_R+1	TGGAAGACTAGTGATTTTGTTGTT	up	XLOC_022858	up
dre-miR-194a_R+2	TGTAACAGCAACTCCATGTGGAT	up	XLOC_022868	down
tni-miR-194_R+1	TGTAACAGCAACTCCATGTGGA	up	XLOC_022868	down
PC-3p-50929_43	TGGAAGTGTCAGAAATTCTGAGT	up	XLOC_022897	up
aca-miR-200b-3p_R+2	TAATACTGCCTGGTAATGATGAAT	up	XLOC_022897	up
ola-miR-194-3p_1ss20CT	CCAGTGGAGGTGCTGTTACTTG	up	XLOC_022897	up
ssa-miR-26d-5p_L+1_1ss13TC	CTTCAAGTAATCCAGGATAGGCT	up	XLOC_022903	up
ssa-miR-730a-5p_R-1	TCCTCATTGTGCATGCTGTGT	down	XLOC_022903	up
PC-3p-11630_419	ATGAGGAAAAGAAGTTAGGAGA	down	XLOC_022905	down

PC-5p-8690_526	GATGTTGAGTATCAAACTGTAT	down	XLOC_022920	down
ola-miR-146a-5p_1ss24TA	TGAGAACTGAATTCCATAGATGGAA	up	XLOC_022920	down
ssa-miR-199a-3p_R+2	ACAGTAGTCTGCACATTGGTTTT	up	XLOC_022920	down
ssa-miR-1338-5p_R+1	AGGACTGTCCAACCTGAGAATG	down	XLOC_022924	up
dre-miR-24_R+2_1	TGGCTCAGTTCAGCAGGAACAGAA	up	XLOC_022980	down
dre-miR-24_R+2_2	TGGCTCAGTTCAGCAGGAACAGTT	up	XLOC_022980	down
dre-miR-24_R+2_1	TGGCTCAGTTCAGCAGGAACAGAA	up	XLOC_023108	down
dre-miR-24_R+2_2	TGGCTCAGTTCAGCAGGAACAGTT	up	XLOC_023108	down
ssa-miR-16b-5p_R-1_1ss21TC	TAGCAGCACGTAAATATTGGC	down	XLOC_023108	down
PC-3p-11630_419	ATGAGGAAAAGAAGTTAGGAGA	down	XLOC_023109	down
PC-3p-50929_43	TGGAAGTGTCAGAAATTCTGAGT	up	XLOC_023109	down
dre-miR-22a-3p	AAGCTGCCAGCTGAAGAACTGT	up	XLOC_023109	down
ola-miR-194-3p_1ss20CT	CCAGTGGAGGTGCTGTTACTTG	up	XLOC_023109	down
ola-miR-194-3p_1ss20CT	CCAGTGGAGGTGCTGTTACTTG	up	XLOC_023237	down
ssa-miR-16b-5p_R-1_1ss21TC	TAGCAGCACGTAAATATTGGC	down	XLOC_023237	down
dre-miR-1	TGGAATGTAAAGAAGTATGTAT	up	XLOC_023324	down
ssa-miR-206-3p	TGGAATGTAAGGAAGTGTGTGG	up	XLOC_023324	down
ssc-miR-206	TGGAATGTAAGGAAGTGTGTGA	up	XLOC_023324	down
ssa-miR-16b-5p_R-1_1ss21TC	TAGCAGCACGTAAATATTGGC	down	XLOC_023376	up
aca-miR-200b-3p_R+2	TAATACTGCCTGGTAATGATGAAT	up	XLOC_023412	up
ssa-miR-1338-5p_R+1	AGGACTGTCCAACCTGAGAATG	down	XLOC_023412	up
dre-miR-22a-3p	AAGCTGCCAGCTGAAGAACTGT	up	XLOC_023438	up
aca-miR-338-3p_R+2	TCCAGCATCAGTGATTTTGTTAA	up	XLOC_023458	up
dre-miR-125b-5p_R+1	TCCCTGAGACCCTAACTTGTGAT	up	XLOC_023458	up
sha-miR-125a_R+2	TCCCTGAGACCCTAACTTGTGAAA	up	XLOC_023458	up
ssa-miR-125b-5p_R-1	TCCCTGAGACCCTTAACCTGTG	up	XLOC_023458	up
ssa-miR-16b-5p_R-1_1ss21TC	TAGCAGCACGTAAATATTGGC	down	XLOC_023458	up
dre-miR-133a-3p_L-1R+1	TTGGTCCCCTTCAACCAGCTGT	up	XLOC_023507	up
dre-miR-21_1ss23CA	TAGCTTATCAGACTGGTGTTGGA	up	XLOC_023507	up
PC-3p-11630_419	ATGAGGAAAAGAAGTTAGGAGA	down	XLOC_023602	up
PC-5p-27517_164	TACATGCAGAGGTGGAGCAAGA	up	XLOC_023602	up
dre-miR-133b-3p_R-1	TTTGGTCCCCTTCAACCAGCT	up	XLOC_023602	up
dre-miR-140-3p_L-1	ACCACAGGGTAGAACCACGGAC	up	XLOC_023602	up
dre-miR-21_1ss23CA	TAGCTTATCAGACTGGTGTTGGA	up	XLOC_023602	up
oha-miR-133b-3p	TTTGGTCCCCTTCAACCAGCTAT	up	XLOC_023602	up
ola-miR-194-3p_1ss20CT	CCAGTGGAGGTGCTGTTACTTG	up	XLOC_023602	up
aca-miR-200b-3p_R+2	TAATACTGCCTGGTAATGATGAAT	up	XLOC_023619	down
dre-miR-142a-3p_R-1	TGTAGTGTTTCCTACTTTATGG	down	XLOC_023619	down
dre-miR-22a-3p	AAGCTGCCAGCTGAAGAACTGT	up	XLOC_023619	down
ola-miR-199a-3p_L+1	AACAGTAGTCTGCACATTGGTTA	up	XLOC_023619	down
PC-5p-8690_526	GATGTTGAGTATCAAACTGTAT	down	XLOC_023622	down
PC-3p-11630_419	ATGAGGAAAAGAAGTTAGGAGA	down	XLOC_023719	up
dre-miR-142a-5p	CATAAAGTAGAAAGCACTACT	down	XLOC_023719	up
aca-miR-200b-3p_R+2	TAATACTGCCTGGTAATGATGAAT	up	XLOC_023764	up
dre-let-7d-5p	TGAGGTAGTTGGTTGTATGGTT	up	XLOC_023764	up
dre-miR-133b-3p_R-1	TTTGGTCCCCTTCAACCAGCT	up	XLOC_023764	up
dre-miR-22a-3p	AAGCTGCCAGCTGAAGAACTGT	up	XLOC_023764	up
mmu-let-7j_1ss8TG	TGAGGTAGTAGTTTGTGCTGTTAT	up	XLOC_023764	up
oha-miR-133b-3p	TTTGGTCCCCTTCAACCAGCTAT	up	XLOC_023764	up
ssa-miR-16b-5p_R-1_1ss21TC	TAGCAGCACGTAAATATTGGC	down	XLOC_023764	up
ssa-miR-26a-4-3p	CCTATTCTTGATTACTTGTTTC	down	XLOC_023764	up
tni-let-7j_1ss11TG	TGAGGTAGTTGTTTGTACAGTT	up	XLOC_023764	up
dre-miR-194a_R+2	TGTAACAGCAACTCCATGTGGAT	up	XLOC_023785	up
ssa-miR-26a-4-3p	CCTATTCTTGATTACTTGTTTC	down	XLOC_023785	up

tni-miR-194_R+1	TGTAACAGCAACTCCATGTGGA	up	XLOC_023785	up
ssa-miR-1-4-5p	ACATACTTCTTTATATGCCCATA	up	XLOC_023840	up
ssa-miR-1338-5p_R+1	AGGACTGTCCAACCTGAGAATG	down	XLOC_023840	up
ssa-miR-26d-5p_L+1_1ss13TC	CTTCAAGTAATCCAGGATAGGCT	up	XLOC_023840	up
ssa-miR-26d-5p_L+1_1ss13TC	CTTCAAGTAATCCAGGATAGGCT	up	XLOC_023874	down
PC-3p-50929_43	TGGAAGTGTCAGAAATTCTGAGT	up	XLOC_023882	down
dre-miR-142a-3p_R-1	TGTAGTGTTTCCTACTTTATGG	down	XLOC_023882	down
dre-miR-194a_R+2	TGTAACAGCAACTCCATGTGGAT	up	XLOC_023884	up
dre-miR-21_1ss23CA	TAGCTTATCAGACTGGTGTTGGA	up	XLOC_023884	up
ssa-miR-16b-5p_R-1_1ss21TC	TAGCAGCACGTAAATATTGGC	down	XLOC_023884	up
ssa-miR-7132a-5p_R+1	GACTTGGTCAAAGCTCCTCAGTT	down	XLOC_023884	up
ssa-miR-7132b-5p	GACTTGGTCAAAGCTCCTCAGC	down	XLOC_023884	up
tni-miR-194_R+1	TGTAACAGCAACTCCATGTGGA	up	XLOC_023884	up
PC-3p-11630_419	ATGAGGAAAAGAAGTTAGGAGA	down	XLOC_023929	up
aca-miR-338-3p_R+2	TCCAGCATCAGTGATTTTGTTAA	up	XLOC_023929	up
dre-miR-1	TGGAATGTAAAGAAGTATGTAT	up	XLOC_023961	up
ssa-miR-206-3p	TGGAATGTAAGGAAGTGTGTGG	up	XLOC_023961	up
ssc-miR-206	TGGAATGTAAGGAAGTGTGTGA	up	XLOC_023961	up
dre-miR-140-3p_L-1	ACCACAGGGTAGAACCACGGAC	up	XLOC_023981	down
ssa-miR-7a-5p	TGGAAGACTAGTGATTTTGTTGT	up	XLOC_023981	down
ssa-miR-7a-5p_R+1	TGGAAGACTAGTGATTTTGTTGTT	up	XLOC_023981	down
dre-miR-125b-5p_R+1	TCCCTGAGACCCTAACTTGTGAT	up	XLOC_024063	up
dre-miR-140-3p_L-1	ACCACAGGGTAGAACCACGGAC	up	XLOC_024063	up
dre-miR-142a-3p_R-1	TGTAGTGTTTCCTACTTTATGG	down	XLOC_024063	up
dre-miR-21_1ss23CA	TAGCTTATCAGACTGGTGTTGGA	up	XLOC_024063	up
ola-miR-146a-5p_1ss24TA	TGAGAACTGAATTCCATAGATGGAA	up	XLOC_024063	up
sha-miR-125a_R+2	TCCCTGAGACCCTAACTTGTGAAA	up	XLOC_024063	up
ssa-miR-125b-5p_R-1	TCCCTGAGACCCTTAACCTGTG	up	XLOC_024063	up
dre-let-7d-5p	TGAGGTAGTTGGTTGTATGGTT	up	XLOC_024204	up
mmu-let-7j_1ss8TG	TGAGGTAGTAGTTTGTGCTGTTAT	up	XLOC_024204	up
tni-let-7j_1ss11TG	TGAGGTAGTTGTTTGTACAGTT	up	XLOC_024204	up
dre-miR-125b-5p_R+1	TCCCTGAGACCCTAACTTGTGAT	up	XLOC_024206	up
sha-miR-125a_R+2	TCCCTGAGACCCTAACTTGTGAAA	up	XLOC_024206	up
ssa-miR-125b-5p_R-1	TCCCTGAGACCCTTAACCTGTG	up	XLOC_024206	up
ola-miR-462_L-1R+4	TAACGGAACCCATAATGCAGCT	down	XLOC_024207	up
tni-miR-10c	TACCCTGTAGATCCGGATTTGT	up	XLOC_024207	up
ssa-miR-1-4-5p	ACATACTTCTTTATATGCCCATA	up	XLOC_024226	down
ssa-miR-16b-5p_R-1_1ss21TC	TAGCAGCACGTAAATATTGGC	down	XLOC_024226	down
ssa-miR-206-3p	TGGAATGTAAGGAAGTGTGTGG	up	XLOC_024226	down
ssa-miR-7132a-5p_R+1	GACTTGGTCAAAGCTCCTCAGTT	down	XLOC_024226	down
ssa-miR-7132b-5p	GACTTGGTCAAAGCTCCTCAGC	down	XLOC_024226	down
ssc-miR-206	TGGAATGTAAGGAAGTGTGTGA	up	XLOC_024226	down
PC-3p-41259_77	TGGCCATTAACTGCTAACCTTC	up	XLOC_024238	down
dre-miR-125b-5p_R+1	TCCCTGAGACCCTAACTTGTGAT	up	XLOC_024238	down
dre-miR-194a_R+2	TGTAACAGCAACTCCATGTGGAT	up	XLOC_024238	down
dre-miR-22a-3p	AAGCTGCCAGCTGAAGAACTGT	up	XLOC_024238	down
sha-miR-125a_R+2	TCCCTGAGACCCTAACTTGTGAAA	up	XLOC_024238	down
ssa-miR-125b-5p_R-1	TCCCTGAGACCCTTAACCTGTG	up	XLOC_024238	down
tni-miR-194_R+1	TGTAACAGCAACTCCATGTGGA	up	XLOC_024238	down
ssa-miR-16b-5p_R-1_1ss21TC	TAGCAGCACGTAAATATTGGC	down	XLOC_024239	down
PC-5p-45063_62	AAGGATAACTACAACTGTACTT	up	XLOC_024241	down
dre-miR-24_R+2_1	TGGCTCAGTTCAGCAGGAACAGAA	up	XLOC_024241	down
dre-miR-24_R+2_2	TGGCTCAGTTCAGCAGGAACAGTT	up	XLOC_024241	down
ola-miR-199a-3p_L+1	AACAGTAGTCTGCACATTGGTTA	up	XLOC_024241	down

dre-miR-1	TGGAATGTAAAGAAGTATGTAT	up	XLOC_024250	up
ola-miR-194-3p_1ss20CT	CCAGTGGAGGTGCTGTTACTTG	up	XLOC_024250	up
ssa-miR-1338-5p_R+1	AGGACTGTCCAACCTGAGAATG	down	XLOC_024250	up
ssa-miR-206-3p	TGGAATGTAAGGAAGTGTGTGG	up	XLOC_024250	up
ssc-miR-206	TGGAATGTAAGGAAGTGTGTGA	up	XLOC_024250	up
PC-3p-50929_43	TGGAAGTGTCAGAAATTCTGAGT	up	XLOC_024271	down
ssa-miR-7a-5p	TGGAAGACTAGTGATTTTGTTGT	up	XLOC_024271	down
ssa-miR-7a-5p_R+1	TGGAAGACTAGTGATTTTGTTGTT	up	XLOC_024271	down
dre-miR-1	TGGAATGTAAAGAAGTATGTAT	up	XLOC_024276	down
dre-miR-22a-3p	AAGCTGCCAGCTGAAGAACTGT	up	XLOC_024276	down
ola-miR-146a-5p_1ss24TA	TGAGAACTGAATTCCATAGATGGAA	up	XLOC_024276	down
ola-miR-199a-3p_L+1	AACAGTAGTCTGCACATTGGTTA	up	XLOC_024276	down
ssa-miR-199a-3p_R+2	ACAGTAGTCTGCACATTGGTTTT	up	XLOC_024276	down
ssa-miR-206-3p	TGGAATGTAAGGAAGTGTGTGG	up	XLOC_024276	down
ssc-miR-206	TGGAATGTAAGGAAGTGTGTGA	up	XLOC_024276	down
ola-miR-194-3p_1ss20CT	CCAGTGGAGGTGCTGTTACTTG	up	XLOC_024289	down
ssa-miR-1338-5p_R+1	AGGACTGTCCAACCTGAGAATG	down	XLOC_024289	down
ssa-miR-7a-5p	TGGAAGACTAGTGATTTTGTTGT	up	XLOC_024290	up
ssa-miR-7a-5p_R+1	TGGAAGACTAGTGATTTTGTTGTT	up	XLOC_024290	up
PC-5p-27517_164	TACATGCAGAGGTGGAGCAAGA	up	XLOC_024324	up
dre-miR-194a_R+2	TGTAACAGCAACTCCATGTGGAT	up	XLOC_024324	up
ola-miR-146a-5p_1ss24TA	TGAGAACTGAATTCCATAGATGGAA	up	XLOC_024324	up
ssa-miR-7132a-5p_R+1	GACTTGGTCAAAGCTCCTCAGTT	down	XLOC_024324	up
ssa-miR-7132b-5p	GACTTGGTCAAAGCTCCTCAGC	down	XLOC_024324	up
ssa-miR-7a-5p	TGGAAGACTAGTGATTTTGTTGT	up	XLOC_024324	up
ssa-miR-7a-5p_R+1	TGGAAGACTAGTGATTTTGTTGTT	up	XLOC_024324	up
tni-miR-194_R+1	TGTAACAGCAACTCCATGTGGA	up	XLOC_024324	up
dre-miR-133a-3p_L-1R+1	TTGGTCCCCTTCAACCAGCTGT	up	XLOC_024325	down
dre-miR-24_R+2_1	TGGCTCAGTTCAGCAGGAACAGAA	up	XLOC_024341	down
dre-miR-24_R+2_2	TGGCTCAGTTCAGCAGGAACAGTT	up	XLOC_024341	down
ssa-miR-7132b-3p	TGAGGCGTTTAGAACAAGTTCA	down	XLOC_024341	down
PC-5p-27517_164	TACATGCAGAGGTGGAGCAAGA	up	XLOC_024360	down
PC-5p-45063_62	AAGGATAACTACAACTGTACTT	up	XLOC_024360	down
dre-miR-194a_R+2	TGTAACAGCAACTCCATGTGGAT	up	XLOC_024360	down
ssa-miR-7a-5p	TGGAAGACTAGTGATTTTGTTGT	up	XLOC_024360	down
ssa-miR-7a-5p_R+1	TGGAAGACTAGTGATTTTGTTGTT	up	XLOC_024360	down
tni-miR-194_R+1	TGTAACAGCAACTCCATGTGGA	up	XLOC_024360	down
PC-3p-41259_77	TGGCCATTAACTGCTAACCTTC	up	XLOC_024363	down
PC-5p-8690_526	GATGTTGAGTATCAAACTGTAT	down	XLOC_024363	down
dre-miR-1	TGGAATGTAAAGAAGTATGTAT	up	XLOC_024363	down
ola-miR-199a-3p_L+1	AACAGTAGTCTGCACATTGGTTA	up	XLOC_024363	down
ssa-miR-16b-5p_R-1_1ss21TC	TAGCAGCACGTAAATATTGGC	down	XLOC_024363	down
ssa-miR-206-3p	TGGAATGTAAGGAAGTGTGTGG	up	XLOC_024363	down
ssa-miR-7132a-5p_R+1	GACTTGGTCAAAGCTCCTCAGTT	down	XLOC_024363	down
ssa-miR-7132b-5p	GACTTGGTCAAAGCTCCTCAGC	down	XLOC_024363	down
ssc-miR-206	TGGAATGTAAGGAAGTGTGTGA	up	XLOC_024363	down
ssa-miR-26d-5p_L+1_1ss13TC	CTTCAAGTAATCCAGGATAGGCT	up	XLOC_024369	down
aca-miR-200b-3p_R+2	TAATACTGCCTGGTAATGATGAAT	up	XLOC_024436	down
dre-miR-194a_R+2	TGTAACAGCAACTCCATGTGGAT	up	XLOC_024437	down
dre-miR-24_R+2_1	TGGCTCAGTTCAGCAGGAACAGAA	up	XLOC_024437	down
dre-miR-24_R+2_2	TGGCTCAGTTCAGCAGGAACAGTT	up	XLOC_024437	down
tni-miR-194_R+1	TGTAACAGCAACTCCATGTGGA	up	XLOC_024437	down
dre-miR-125b-5p_R+1	TCCCTGAGACCCTAACTTGTGAT	up	XLOC_024518	up
dre-miR-140-3p_L-1	ACCACAGGGTAGAACCACGGAC	up	XLOC_024518	up

dre-miR-24_R+2_1	TGGCTCAGTTCAGCAGGAACAGAA	up	XLOC_024518	up
dre-miR-24_R+2_2	TGGCTCAGTTCAGCAGGAACAGTT	up	XLOC_024518	up
sha-miR-125a_R+2	TCCCTGAGACCCTAACTTGTGAAA	up	XLOC_024518	up
ssa-miR-125b-5p_R-1	TCCCTGAGACCCTTAACCTGTG	up	XLOC_024518	up
PC-5p-27517_164	TACATGCAGAGGTGGAGCAAGA	up	XLOC_024548	up
dre-miR-142a-3p_R-1	TGTAGTGTTTCCTACTTTATGG	down	XLOC_024622	up
mmu-let-7j_1ss8TG	TGAGGTAGTAGTTTGTGCTGTTAT	up	XLOC_024627	down
ssa-miR-1338-5p_R+1	AGGACTGTCCAACCTGAGAATG	down	XLOC_024627	down
ssa-miR-26a-4-3p	CCTATTCTTGATTACTTGTTTC	down	XLOC_024627	down
tni-let-7j_1ss11TG	TGAGGTAGTTGTTTGTACAGTT	up	XLOC_024627	down
ssa-miR-26a-4-3p	CCTATTCTTGATTACTTGTTTC	down	XLOC_024681	up
ssa-miR-7a-5p	TGGAAGACTAGTGATTTTGTTGT	up	XLOC_024681	up
ssa-miR-7a-5p_R+1	TGGAAGACTAGTGATTTTGTTGTT	up	XLOC_024681	up
ola-mir-100-2-p3	CAAGCTCGTATCTATAGGTATG	down	XLOC_024703	down
ssa-miR-199a-3p_R+2	ACAGTAGTCTGCACATTGGTTTT	up	XLOC_024703	down
PC-3p-11630_419	ATGAGGAAAAGAAGTTAGGAGA	down	XLOC_024708	up
ssa-miR-7132b-3p	TGAGGCGTTTAGAACAAGTTCA	down	XLOC_024708	up
PC-3p-11630_419	ATGAGGAAAAGAAGTTAGGAGA	down	XLOC_024853	up
PC-3p-50929_43	TGGAAGTGTCAGAAATTCTGAGT	up	XLOC_024853	up
PC-5p-27517_164	TACATGCAGAGGTGGAGCAAGA	up	XLOC_024853	up
aca-miR-338-3p_R+2	TCCAGCATCAGTGATTTTGTTAA	up	XLOC_024853	up
dre-miR-125b-5p_R+1	TCCCTGAGACCCTAACTTGTGAT	up	XLOC_024853	up
dre-miR-133a-3p_L-1R+1	TTGGTCCCCTTCAACCAGCTGT	up	XLOC_024853	up
dre-miR-22a-3p	AAGCTGCCAGCTGAAGAACTGT	up	XLOC_024853	up
dre-miR-24_R+2_1	TGGCTCAGTTCAGCAGGAACAGAA	up	XLOC_024853	up
dre-miR-24_R+2_2	TGGCTCAGTTCAGCAGGAACAGTT	up	XLOC_024853	up
sha-miR-125a_R+2	TCCCTGAGACCCTAACTTGTGAAA	up	XLOC_024853	up
ssa-miR-125b-5p_R-1	TCCCTGAGACCCTTAACCTGTG	up	XLOC_024853	up
ssa-miR-199a-3p_R+2	ACAGTAGTCTGCACATTGGTTTT	up	XLOC_024853	up
ssa-miR-7a-5p	TGGAAGACTAGTGATTTTGTTGT	up	XLOC_024853	up
ssa-miR-7a-5p_R+1	TGGAAGACTAGTGATTTTGTTGTT	up	XLOC_024853	up
tni-miR-10c	TACCCTGTAGATCCGGATTTGT	up	XLOC_024853	up
dre-miR-194a_R+2	TGTAACAGCAACTCCATGTGGAT	up	XLOC_024886	down
ssa-miR-730a-5p_R-1	TCCTCATTGTGCATGCTGTGT	down	XLOC_024886	down
tni-miR-194_R+1	TGTAACAGCAACTCCATGTGGA	up	XLOC_024886	down
ola-miR-462_L-1R+4	TAACGGAACCCATAATGCAGCT	down	XLOC_024986	up
ssa-miR-26d-5p_L+1_1ss13TC	CTTCAAGTAATCCAGGATAGGCT	up	XLOC_025037	up
dre-miR-1	TGGAATGTAAAGAAGTATGTAT	up	XLOC_025081	up
dre-miR-125b-5p_R+1	TCCCTGAGACCCTAACTTGTGAT	up	XLOC_025081	up
ola-miR-194-3p_1ss20CT	CCAGTGGAGGTGCTGTTACTTG	up	XLOC_025081	up
sha-miR-125a_R+2	TCCCTGAGACCCTAACTTGTGAAA	up	XLOC_025081	up
ssa-miR-1-4-5p	ACATACTTCTTTATATGCCCATA	up	XLOC_025081	up
ssa-miR-125b-5p_R-1	TCCCTGAGACCCTTAACCTGTG	up	XLOC_025081	up
ssa-miR-206-3p	TGGAATGTAAGGAAGTGTGTGG	up	XLOC_025081	up
ssc-miR-206	TGGAATGTAAGGAAGTGTGTGA	up	XLOC_025081	up
tni-miR-10c	TACCCTGTAGATCCGGATTTGT	up	XLOC_025081	up
aca-miR-338-3p_R+2	TCCAGCATCAGTGATTTTGTTAA	up	XLOC_025100	up
dre-miR-194a_R+2	TGTAACAGCAACTCCATGTGGAT	up	XLOC_025100	up
ssa-miR-7132a-5p_R+1	GACTTGGTCAAAGCTCCTCAGTT	down	XLOC_025100	up
ssa-miR-7132b-5p	GACTTGGTCAAAGCTCCTCAGC	down	XLOC_025100	up
tni-miR-194_R+1	TGTAACAGCAACTCCATGTGGA	up	XLOC_025100	up
PC-3p-50929_43	TGGAAGTGTCAGAAATTCTGAGT	up	XLOC_025113	down
PC-5p-27517_164	TACATGCAGAGGTGGAGCAAGA	up	XLOC_025113	down
PC-5p-8690_526	GATGTTGAGTATCAAACTGTAT	down	XLOC_025113	down

dre-miR-142a-3p_R-1	TGTAGTGTTTCCTACTTTATGG	down	XLOC_025113	down
rno-miR-122-5p_L+3	ATCTGGAGTGTGACAATGGTGTTTG	up	XLOC_025168	up
dre-miR-142a-3p_R-1	TGTAGTGTTTCCTACTTTATGG	down	XLOC_025206	down
dre-miR-122	TGGAGTGTGACAATGGTGTTTG	up	XLOC_025234	up
dre-miR-24_R+2_1	TGGCTCAGTTCAGCAGGAACAGAA	up	XLOC_025234	up
dre-miR-24_R+2_2	TGGCTCAGTTCAGCAGGAACAGTT	up	XLOC_025234	up
ssa-miR-1-4-5p	ACATACTTCTTTATATGCCCATA	up	XLOC_025234	up
ssa-miR-16b-5p_R-1_1ss21TC	TAGCAGCACGTAAATATTGGC	down	XLOC_025234	up
ssa-miR-7132a-5p_R+1	GACTTGGTCAAAGCTCCTCAGTT	down	XLOC_025234	up
ssa-miR-7132b-5p	GACTTGGTCAAAGCTCCTCAGC	down	XLOC_025234	up
PC-3p-41259_77	TGGCCATTAACTGCTAACCTTC	up	XLOC_025245	up
aca-miR-338-3p_R+2	TCCAGCATCAGTGATTTTGTTAA	up	XLOC_025247	up
dre-miR-1	TGGAATGTAAAGAAGTATGTAT	up	XLOC_025247	up
dre-miR-133a-3p_L-1R+1	TTGGTCCCCTTCAACCAGCTGT	up	XLOC_025247	up
dre-miR-133b-3p_R-1	TTTGGTCCCCTTCAACCAGCT	up	XLOC_025247	up
dre-miR-140-3p_L-1	ACCACAGGGTAGAACCACGGAC	up	XLOC_025247	up
oha-miR-133b-3p	TTTGGTCCCCTTCAACCAGCTAT	up	XLOC_025247	up
PC-3p-11630_419	ATGAGGAAAAGAAGTTAGGAGA	down	XLOC_025440	up
ssa-miR-7a-5p	TGGAAGACTAGTGATTTTGTTGT	up	XLOC_025440	up
ssa-miR-7a-5p_R+1	TGGAAGACTAGTGATTTTGTTGTT	up	XLOC_025440	up
ssa-miR-7132a-5p_R+1	GACTTGGTCAAAGCTCCTCAGTT	down	XLOC_025542	up
ssa-miR-7132b-5p	GACTTGGTCAAAGCTCCTCAGC	down	XLOC_025542	up
ola-miR-146a-5p_1ss24TA	TGAGAACTGAATTCCATAGATGGAA	up	XLOC_025564	down
dre-miR-125b-5p_R+1	TCCCTGAGACCCTAACTTGTGAT	up	XLOC_025586	down
dre-miR-133a-3p_L-1R+1	TTGGTCCCCTTCAACCAGCTGT	up	XLOC_025586	down
ola-miR-146a-5p_1ss24TA	TGAGAACTGAATTCCATAGATGGAA	up	XLOC_025586	down
sha-miR-125a_R+2	TCCCTGAGACCCTAACTTGTGAAA	up	XLOC_025586	down
ssa-miR-125b-5p_R-1	TCCCTGAGACCCTTAACCTGTG	up	XLOC_025586	down
ssa-miR-7132b-3p	TGAGGCGTTTAGAACAAGTTCA	down	XLOC_025616	up
PC-3p-50929_43	TGGAAGTGTCAGAAATTCTGAGT	up	XLOC_025704	down
dre-miR-133b-3p_R-1	TTTGGTCCCCTTCAACCAGCT	up	XLOC_025704	down
oha-miR-133b-3p	TTTGGTCCCCTTCAACCAGCTAT	up	XLOC_025704	down
xtr-miR-122_L+1R-1	CTGGAGTGTGACAATGGTGTTTG	up	XLOC_025704	down
ola-mir-100-2-p3	CAAGCTCGTATCTATAGGTATG	down	XLOC_025841	up
dre-miR-140-3p_L-1	ACCACAGGGTAGAACCACGGAC	up	XLOC_025873	down
dre-miR-22a-3p	AAGCTGCCAGCTGAAGAACTGT	up	XLOC_025873	down
ola-miR-194-3p_1ss20CT	CCAGTGGAGGTGCTGTTACTTG	up	XLOC_025873	down
rno-miR-122-5p_L+3	ATCTGGAGTGTGACAATGGTGTTTG	up	XLOC_025873	down
ssa-miR-7a-5p	TGGAAGACTAGTGATTTTGTTGT	up	XLOC_025873	down
ssa-miR-7a-5p_R+1	TGGAAGACTAGTGATTTTGTTGTT	up	XLOC_025873	down
ola-miR-146a-5p_1ss24TA	TGAGAACTGAATTCCATAGATGGAA	up	XLOC_026130	up
dre-miR-1	TGGAATGTAAAGAAGTATGTAT	up	XLOC_026195	up
ola-miR-462_L-1R+4	TAACGGAACCCATAATGCAGCT	down	XLOC_026195	up
dre-let-7d-5p	TGAGGTAGTTGGTTGTATGGTT	up	XLOC_026204	down
dre-miR-194a_R+2	TGTAACAGCAACTCCATGTGGAT	up	XLOC_026204	down
tni-let-7j_1ss11TG	TGAGGTAGTTGTTTGTACAGTT	up	XLOC_026204	down
tni-miR-10c	TACCCTGTAGATCCGGATTTGT	up	XLOC_026204	down
tni-miR-194_R+1	TGTAACAGCAACTCCATGTGGA	up	XLOC_026204	down
PC-3p-11630_419	ATGAGGAAAAGAAGTTAGGAGA	down	XLOC_026248	up
dre-miR-140-3p_L-1	ACCACAGGGTAGAACCACGGAC	up	XLOC_026248	up
dre-miR-194a_R+2	TGTAACAGCAACTCCATGTGGAT	up	XLOC_026397	down
dre-miR-21_1ss23CA	TAGCTTATCAGACTGGTGTTGGA	up	XLOC_026397	down
tni-miR-194_R+1	TGTAACAGCAACTCCATGTGGA	up	XLOC_026397	down
PC-5p-27517_164	TACATGCAGAGGTGGAGCAAGA	up	XLOC_026429	down

PC-5p-8690_526	GATGTTGAGTATCAAACTGTAT	down	XLOC_026429	down
dre-miR-142a-3p_R-1	TGTAGTGTTTCCTACTTTATGG	down	XLOC_026429	down
ola-mir-100-2-p3	CAAGCTCGTATCTATAGGTATG	down	XLOC_026429	down
PC-3p-41259_77	TGGCCATTAACTGCTAACCTTC	up	XLOC_026500	up
ola-miR-146a-5p_1ss24TA	TGAGAACTGAATTCCATAGATGGAA	up	XLOC_026500	up
ola-miR-194-3p_1ss20CT	CCAGTGGAGGTGCTGTTACTTG	up	XLOC_026503	down
dre-miR-194a_R+2	TGTAACAGCAACTCCATGTGGAT	up	XLOC_026540	down
tni-miR-194_R+1	TGTAACAGCAACTCCATGTGGA	up	XLOC_026540	down
dre-miR-122	TGGAGTGTGACAATGGTGTTTG	up	XLOC_026541	up
dre-miR-133a-3p_L-1R+1	TTGGTCCCCTTCAACCAGCTGT	up	XLOC_026607	up
rno-miR-122-5p_L+3	ATCTGGAGTGTGACAATGGTGTTTG	up	XLOC_026607	up
ssa-miR-7132b-3p	TGAGGCGTTTAGAACAAGTTCA	down	XLOC_026607	up
xtr-miR-122_L+1R-1	CTGGAGTGTGACAATGGTGTTTG	up	XLOC_026668	up
aca-miR-200b-3p_R+2	TAATACTGCCTGGTAATGATGAAT	up	XLOC_026724	down
dre-miR-140-3p_L-1	ACCACAGGGTAGAACCACGGAC	up	XLOC_026826	down
ola-mir-100-2-p3	CAAGCTCGTATCTATAGGTATG	down	XLOC_026826	down
aca-miR-200b-3p_R+2	TAATACTGCCTGGTAATGATGAAT	up	XLOC_026841	down
dre-miR-24_R+2_1	TGGCTCAGTTCAGCAGGAACAGAA	up	XLOC_026862	up
dre-miR-24_R+2_2	TGGCTCAGTTCAGCAGGAACAGTT	up	XLOC_026862	up
dre-miR-140-3p_L-1	ACCACAGGGTAGAACCACGGAC	up	XLOC_026893	up
dre-miR-22a-3p	AAGCTGCCAGCTGAAGAACTGT	up	XLOC_026893	up
ola-miR-146a-5p_1ss24TA	TGAGAACTGAATTCCATAGATGGAA	up	XLOC_026893	up
PC-5p-27517_164	TACATGCAGAGGTGGAGCAAGA	up	XLOC_027063	up
ssa-miR-16b-5p_R-1_1ss21TC	TAGCAGCACGTAAATATTGGC	down	XLOC_027063	up
dre-miR-1	TGGAATGTAAAGAAGTATGTAT	up	XLOC_027078	up
dre-miR-142a-5p	CATAAAGTAGAAAGCACTACT	down	XLOC_027078	up
ssa-miR-16b-5p_R-1_1ss21TC	TAGCAGCACGTAAATATTGGC	down	XLOC_027078	up
ssa-miR-206-3p	TGGAATGTAAGGAAGTGTGTGG	up	XLOC_027078	up
ssc-miR-206	TGGAATGTAAGGAAGTGTGTGA	up	XLOC_027078	up
dre-miR-194a_R+2	TGTAACAGCAACTCCATGTGGAT	up	XLOC_027117	down
tni-miR-194_R+1	TGTAACAGCAACTCCATGTGGA	up	XLOC_027117	down
PC-3p-50929_43	TGGAAGTGTCAGAAATTCTGAGT	up	XLOC_027166	up
dre-miR-142a-3p_R-1	TGTAGTGTTTCCTACTTTATGG	down	XLOC_027166	up
dre-miR-142a-5p	CATAAAGTAGAAAGCACTACT	down	XLOC_027166	up
ssa-miR-730a-5p_R-1	TCCTCATTGTGCATGCTGTGT	down	XLOC_027166	up
ola-miR-194-3p_1ss20CT	CCAGTGGAGGTGCTGTTACTTG	up	XLOC_027193	up
dre-miR-194a_R+2	TGTAACAGCAACTCCATGTGGAT	up	XLOC_027204	down
ssa-miR-16b-5p_R-1_1ss21TC	TAGCAGCACGTAAATATTGGC	down	XLOC_027204	down
tni-miR-194_R+1	TGTAACAGCAACTCCATGTGGA	up	XLOC_027204	down
ola-miR-146a-5p_1ss24TA	TGAGAACTGAATTCCATAGATGGAA	up	XLOC_027215	down
xtr-miR-122_L+1R-1	CTGGAGTGTGACAATGGTGTTTG	up	XLOC_027240	down
aca-miR-338-3p_R+2	TCCAGCATCAGTGATTTTGTTAA	up	XLOC_027387	up
dre-miR-22a-3p	AAGCTGCCAGCTGAAGAACTGT	up	XLOC_027387	up
ssa-miR-7132a-5p_R+1	GACTTGGTCAAAGCTCCTCAGTT	down	XLOC_027387	up
ssa-miR-7132b-5p	GACTTGGTCAAAGCTCCTCAGC	down	XLOC_027387	up
dre-miR-140-3p_L-1	ACCACAGGGTAGAACCACGGAC	up	XLOC_027428	down
ola-miR-462_L-1R+4	TAACGGAACCCATAATGCAGCT	down	XLOC_027428	down
PC-5p-27517_164	TACATGCAGAGGTGGAGCAAGA	up	XLOC_027484	down
dre-miR-22a-3p	AAGCTGCCAGCTGAAGAACTGT	up	XLOC_027484	down
ola-miR-199a-3p_L+1	AACAGTAGTCTGCACATTGGTTA	up	XLOC_027484	down
rno-miR-122-5p_L+3	ATCTGGAGTGTGACAATGGTGTTTG	up	XLOC_027484	down
ssa-miR-199a-3p_R+2	ACAGTAGTCTGCACATTGGTTTT	up	XLOC_027484	down
tni-miR-10c	TACCCTGTAGATCCGGATTTGT	up	XLOC_027484	down
PC-5p-45063_62	AAGGATAACTACAACTGTACTT	up	XLOC_027505	down

dre-miR-142a-5p	CATAAAGTAGAAAGCACTACT	down	XLOC_027505	down
ola-miR-146a-5p_1ss24TA	TGAGAACTGAATTCCATAGATGGAA	up	XLOC_027505	down
xtr-miR-122_L+1R-1	CTGGAGTGTGACAATGGTGTTTG	up	XLOC_027505	down
PC-5p-8690_526	GATGTTGAGTATCAAACTGTAT	down	XLOC_027518	down
dre-miR-125b-5p_R+1	TCCCTGAGACCCTAACTTGTGAT	up	XLOC_027524	down
sha-miR-125a_R+2	TCCCTGAGACCCTAACTTGTGAAA	up	XLOC_027524	down
ssa-miR-125b-5p_R-1	TCCCTGAGACCCTTAACCTGTG	up	XLOC_027524	down
dre-miR-140-3p_L-1	ACCACAGGGTAGAACCACGGAC	up	XLOC_027525	up
PC-3p-11630_419	ATGAGGAAAAGAAGTTAGGAGA	down	XLOC_027531	up
PC-5p-8690_526	GATGTTGAGTATCAAACTGTAT	down	XLOC_027531	up
dre-miR-125b-5p_R+1	TCCCTGAGACCCTAACTTGTGAT	up	XLOC_027531	up
ola-miR-194-3p_1ss20CT	CCAGTGGAGGTGCTGTTACTTG	up	XLOC_027531	up
sha-miR-125a_R+2	TCCCTGAGACCCTAACTTGTGAAA	up	XLOC_027531	up
ssa-miR-125b-5p_R-1	TCCCTGAGACCCTTAACCTGTG	up	XLOC_027531	up
PC-5p-8690_526	GATGTTGAGTATCAAACTGTAT	down	XLOC_027534	down
dre-miR-133a-3p_L-1R+1	TTGGTCCCCTTCAACCAGCTGT	up	XLOC_027534	down
dre-miR-194a_R+2	TGTAACAGCAACTCCATGTGGAT	up	XLOC_027534	down
ssa-miR-1338-5p_R+1	AGGACTGTCCAACCTGAGAATG	down	XLOC_027534	down
ssa-miR-16b-5p_R-1_1ss21TC	TAGCAGCACGTAAATATTGGC	down	XLOC_027534	down
ssa-miR-26d-5p_L+1_1ss13TC	CTTCAAGTAATCCAGGATAGGCT	up	XLOC_027534	down
tni-miR-194_R+1	TGTAACAGCAACTCCATGTGGA	up	XLOC_027534	down
ssa-miR-1338-5p_R+1	AGGACTGTCCAACCTGAGAATG	down	XLOC_027546	up
dre-miR-122	TGGAGTGTGACAATGGTGTTTG	up	XLOC_027555	up
dre-miR-125b-5p_R+1	TCCCTGAGACCCTAACTTGTGAT	up	XLOC_027728	up
sha-miR-125a_R+2	TCCCTGAGACCCTAACTTGTGAAA	up	XLOC_027728	up
ssa-miR-125b-5p_R-1	TCCCTGAGACCCTTAACCTGTG	up	XLOC_027728	up
PC-3p-50929_43	TGGAAGTGTCAGAAATTCTGAGT	up	XLOC_027753	down
dre-miR-122	TGGAGTGTGACAATGGTGTTTG	up	XLOC_027753	down
ssa-miR-16b-5p_R-1_1ss21TC	TAGCAGCACGTAAATATTGGC	down	XLOC_027753	down
dre-miR-22a-3p	AAGCTGCCAGCTGAAGAACTGT	up	XLOC_027771	up
dre-miR-194a_R+2	TGTAACAGCAACTCCATGTGGAT	up	XLOC_027786	up
ssa-miR-199a-3p_R+2	ACAGTAGTCTGCACATTGGTTTT	up	XLOC_027786	up
ssa-miR-7a-5p	TGGAAGACTAGTGATTTTGTTGT	up	XLOC_027786	up
ssa-miR-7a-5p_R+1	TGGAAGACTAGTGATTTTGTTGTT	up	XLOC_027786	up
tni-miR-194_R+1	TGTAACAGCAACTCCATGTGGA	up	XLOC_027786	up
dre-miR-122	TGGAGTGTGACAATGGTGTTTG	up	XLOC_027859	up
dre-miR-142a-5p	CATAAAGTAGAAAGCACTACT	down	XLOC_027914	down
dre-miR-22a-3p	AAGCTGCCAGCTGAAGAACTGT	up	XLOC_027914	down
ssa-miR-1338-5p_R+1	AGGACTGTCCAACCTGAGAATG	down	XLOC_027914	down
ssa-miR-16b-5p_R-1_1ss21TC	TAGCAGCACGTAAATATTGGC	down	XLOC_027914	down
ssa-miR-16b-5p_R-1_1ss21TC	TAGCAGCACGTAAATATTGGC	down	XLOC_027931	up
ssa-miR-1-4-5p	ACATACTTCTTTATATGCCCATA	up	XLOC_027935	down
ssa-miR-730a-5p_R-1	TCCTCATTGTGCATGCTGTGT	down	XLOC_027935	down
PC-5p-27517_164	TACATGCAGAGGTGGAGCAAGA	up	XLOC_027947	down
dre-miR-194a_R+2	TGTAACAGCAACTCCATGTGGAT	up	XLOC_027947	down
tni-miR-194_R+1	TGTAACAGCAACTCCATGTGGA	up	XLOC_027947	down
dre-let-7d-5p	TGAGGTAGTTGGTTGTATGGTT	up	XLOC_027953	up
PC-3p-50929_43	TGGAAGTGTCAGAAATTCTGAGT	up	XLOC_027954	down
dre-let-7d-5p	TGAGGTAGTTGGTTGTATGGTT	up	XLOC_027991	down
mmu-let-7j_1ss8TG	TGAGGTAGTAGTTTGTGCTGTTAT	up	XLOC_027991	down
ola-miR-199a-3p_L+1	AACAGTAGTCTGCACATTGGTTA	up	XLOC_027991	down
tni-let-7j_1ss11TG	TGAGGTAGTTGTTTGTACAGTT	up	XLOC_027991	down
ola-miR-194-3p_1ss20CT	CCAGTGGAGGTGCTGTTACTTG	up	XLOC_028287	down
ola-miR-194-3p_1ss20CT	CCAGTGGAGGTGCTGTTACTTG	up	XLOC_028290	down

ola-mir-100-2-p3	CAAGCTCGTATCTATAGGTATG	down	XLOC_028290	down
aca-miR-200b-3p_R+2	TAATACTGCCTGGTAATGATGAAT	up	XLOC_028314	down
tni-miR-10c	TACCCTGTAGATCCGGATTTGT	up	XLOC_028314	down
PC-5p-27517_164	TACATGCAGAGGTGGAGCAAGA	up	XLOC_028334	up
rno-miR-122-5p_L+3	ATCTGGAGTGTGACAATGGTGTTTG	up	XLOC_028334	up
ssa-miR-16b-5p_R-1_1ss21TC	TAGCAGCACGTAAATATTGGC	down	XLOC_028334	up
xtr-miR-122_L+1R-1	CTGGAGTGTGACAATGGTGTTTG	up	XLOC_028334	up
ssa-miR-26d-5p_L+1_1ss13TC	CTTCAAGTAATCCAGGATAGGCT	up	XLOC_028367	down
ssa-miR-730a-5p_R-1	TCCTCATTGTGCATGCTGTGT	down	XLOC_028367	down
PC-5p-8690_526	GATGTTGAGTATCAAACTGTAT	down	XLOC_028382	down
PC-5p-27517_164	TACATGCAGAGGTGGAGCAAGA	up	XLOC_028496	down
dre-let-7d-5p	TGAGGTAGTTGGTTGTATGGTT	up	XLOC_028496	down
dre-miR-194a_R+2	TGTAACAGCAACTCCATGTGGAT	up	XLOC_028496	down
mmu-let-7j_1ss8TG	TGAGGTAGTAGTTTGTGCTGTTAT	up	XLOC_028496	down
ola-miR-199a-3p_L+1	AACAGTAGTCTGCACATTGGTTA	up	XLOC_028496	down
tni-let-7j_1ss11TG	TGAGGTAGTTGTTTGTACAGTT	up	XLOC_028496	down
tni-miR-194_R+1	TGTAACAGCAACTCCATGTGGA	up	XLOC_028496	down
dre-miR-194a_R+2	TGTAACAGCAACTCCATGTGGAT	up	XLOC_028538	up
ssa-miR-26a-4-3p	CCTATTCTTGATTACTTGTTTC	down	XLOC_028538	up
tni-miR-194_R+1	TGTAACAGCAACTCCATGTGGA	up	XLOC_028538	up
dre-miR-194a_R+2	TGTAACAGCAACTCCATGTGGAT	up	XLOC_028540	down
ola-miR-199a-3p_L+1	AACAGTAGTCTGCACATTGGTTA	up	XLOC_028540	down
ola-mir-100-2-p3	CAAGCTCGTATCTATAGGTATG	down	XLOC_028540	down
tni-miR-194_R+1	TGTAACAGCAACTCCATGTGGA	up	XLOC_028540	down
dre-miR-24_R+2_1	TGGCTCAGTTCAGCAGGAACAGAA	up	XLOC_028660	down
dre-miR-24_R+2_2	TGGCTCAGTTCAGCAGGAACAGTT	up	XLOC_028660	down
ssa-miR-730a-5p_R-1	TCCTCATTGTGCATGCTGTGT	down	XLOC_028660	down
dre-let-7d-5p	TGAGGTAGTTGGTTGTATGGTT	up	XLOC_028699	down
dre-miR-142a-5p	CATAAAGTAGAAAGCACTACT	down	XLOC_028699	down
mmu-let-7j_1ss8TG	TGAGGTAGTAGTTTGTGCTGTTAT	up	XLOC_028699	down
tni-let-7j_1ss11TG	TGAGGTAGTTGTTTGTACAGTT	up	XLOC_028699	down
dre-miR-1	TGGAATGTAAAGAAGTATGTAT	up	XLOC_028701	down
ssa-miR-206-3p	TGGAATGTAAGGAAGTGTGTGG	up	XLOC_028701	down
ssc-miR-206	TGGAATGTAAGGAAGTGTGTGA	up	XLOC_028701	down
ola-miR-146a-5p_1ss24TA	TGAGAACTGAATTCCATAGATGGAA	up	XLOC_028774	up
ola-miR-194-3p_1ss20CT	CCAGTGGAGGTGCTGTTACTTG	up	XLOC_028774	up
PC-3p-41259_77	TGGCCATTAACTGCTAACCTTC	up	XLOC_028813	down
dre-miR-194a_R+2	TGTAACAGCAACTCCATGTGGAT	up	XLOC_028813	down
ssa-miR-16b-5p_R-1_1ss21TC	TAGCAGCACGTAAATATTGGC	down	XLOC_028813	down
ssa-miR-26a-4-3p	CCTATTCTTGATTACTTGTTTC	down	XLOC_028813	down
ssa-miR-7a-5p	TGGAAGACTAGTGATTTTGTTGT	up	XLOC_028813	down
ssa-miR-7a-5p_R+1	TGGAAGACTAGTGATTTTGTTGTT	up	XLOC_028813	down
tni-miR-194_R+1	TGTAACAGCAACTCCATGTGGA	up	XLOC_028813	down
dre-miR-24_R+2_1	TGGCTCAGTTCAGCAGGAACAGAA	up	XLOC_028832	down
dre-miR-24_R+2_2	TGGCTCAGTTCAGCAGGAACAGTT	up	XLOC_028832	down
PC-5p-8690_526	GATGTTGAGTATCAAACTGTAT	down	XLOC_028838	down
tni-miR-10c	TACCCTGTAGATCCGGATTTGT	up	XLOC_028838	down
ssa-miR-7a-5p	TGGAAGACTAGTGATTTTGTTGT	up	XLOC_028863	up
ssa-miR-7a-5p_R+1	TGGAAGACTAGTGATTTTGTTGTT	up	XLOC_028863	up
dre-miR-194a_R+2	TGTAACAGCAACTCCATGTGGAT	up	XLOC_028897	down
tni-miR-194_R+1	TGTAACAGCAACTCCATGTGGA	up	XLOC_028897	down
dre-miR-133b-3p_R-1	TTTGGTCCCCTTCAACCAGCT	up	XLOC_029035	up
oha-miR-133b-3p	TTTGGTCCCCTTCAACCAGCTAT	up	XLOC_029035	up
PC-5p-27517_164	TACATGCAGAGGTGGAGCAAGA	up	XLOC_029043	down

ola-miR-194-3p_1ss20CT	CCAGTGGAGGTGCTGTTACTTG	up	XLOC_029043	down
ola-miR-199a-3p_L+1	AACAGTAGTCTGCACATTGGTTA	up	XLOC_029043	down
aca-miR-200b-3p_R+2	TAATACTGCCTGGTAATGATGAAT	up	XLOC_029074	up
ola-miR-194-3p_1ss20CT	CCAGTGGAGGTGCTGTTACTTG	up	XLOC_029160	down
rno-miR-122-5p_L+3	ATCTGGAGTGTGACAATGGTGTTTG	up	XLOC_029215	down
dre-miR-133a-3p_L-1R+1	TTGGTCCCCTTCAACCAGCTGT	up	XLOC_029244	up
PC-5p-45063_62	AAGGATAACTACAACTGTACTT	up	XLOC_029274	up
ssa-miR-199a-3p_R+2	ACAGTAGTCTGCACATTGGTTTT	up	XLOC_029274	up
aca-miR-338-3p_R+2	TCCAGCATCAGTGATTTTGTTAA	up	XLOC_029276	down
ola-miR-146a-5p_1ss24TA	TGAGAACTGAATTCCATAGATGGAA	up	XLOC_029276	down
ssa-miR-1338-5p_R+1	AGGACTGTCCAACCTGAGAATG	down	XLOC_029276	down
ssa-miR-7132a-5p_R+1	GACTTGGTCAAAGCTCCTCAGTT	down	XLOC_029276	down
ssa-miR-7132b-5p	GACTTGGTCAAAGCTCCTCAGC	down	XLOC_029276	down
PC-3p-11630_419	ATGAGGAAAAGAAGTTAGGAGA	down	XLOC_029349	up
PC-3p-50929_43	TGGAAGTGTCAGAAATTCTGAGT	up	XLOC_029349	up
dre-miR-140-3p_L-1	ACCACAGGGTAGAACCACGGAC	up	XLOC_029349	up
dre-miR-194a_R+2	TGTAACAGCAACTCCATGTGGAT	up	XLOC_029349	up
ssa-miR-16b-5p_R-1_1ss21TC	TAGCAGCACGTAAATATTGGC	down	XLOC_029349	up
PC-3p-41259_77	TGGCCATTAACTGCTAACCTTC	up	XLOC_029513	down
ccr-miR-99_R+3	AACCCGTAGATCCGATCTTGTGAA	up	XLOC_029513	down
dre-miR-133b-3p_R-1	TTTGGTCCCCTTCAACCAGCT	up	XLOC_029513	down
dre-miR-142a-3p_R-1	TGTAGTGTTTCCTACTTTATGG	down	XLOC_029513	down
oha-miR-133b-3p	TTTGGTCCCCTTCAACCAGCTAT	up	XLOC_029513	down
ssa-miR-16b-5p_R-1_1ss21TC	TAGCAGCACGTAAATATTGGC	down	XLOC_029513	down
ssa-miR-199a-3p_R+2	ACAGTAGTCTGCACATTGGTTTT	up	XLOC_029513	down
dre-miR-142a-5p	CATAAAGTAGAAAGCACTACT	down	XLOC_029605	up
ssa-miR-26a-4-3p	CCTATTCTTGATTACTTGTTTC	down	XLOC_029628	down
PC-5p-27517_164	TACATGCAGAGGTGGAGCAAGA	up	XLOC_029635	down
dre-miR-133a-3p_L-1R+1	TTGGTCCCCTTCAACCAGCTGT	up	XLOC_029635	down
dre-miR-133b-3p_R-1	TTTGGTCCCCTTCAACCAGCT	up	XLOC_029635	down
oha-miR-133b-3p	TTTGGTCCCCTTCAACCAGCTAT	up	XLOC_029635	down
ola-miR-146a-5p_1ss24TA	TGAGAACTGAATTCCATAGATGGAA	up	XLOC_029635	down
PC-5p-8690_526	GATGTTGAGTATCAAACTGTAT	down	XLOC_029845	up
ssa-miR-199a-3p_R+2	ACAGTAGTCTGCACATTGGTTTT	up	XLOC_029845	up
ssa-miR-1338-5p_R+1	AGGACTGTCCAACCTGAGAATG	down	XLOC_029857	down
dre-miR-140-3p_L-1	ACCACAGGGTAGAACCACGGAC	up	XLOC_030040	up
ssa-miR-730a-5p_R-1	TCCTCATTGTGCATGCTGTGT	down	XLOC_030040	up
tni-miR-10c	TACCCTGTAGATCCGGATTTGT	up	XLOC_030040	up
ssa-miR-7132a-5p_R+1	GACTTGGTCAAAGCTCCTCAGTT	down	XLOC_030042	up
ssa-miR-7132b-5p	GACTTGGTCAAAGCTCCTCAGC	down	XLOC_030042	up
ola-miR-199a-3p_L+1	AACAGTAGTCTGCACATTGGTTA	up	XLOC_030107	down
PC-5p-45063_62	AAGGATAACTACAACTGTACTT	up	XLOC_030108	up
dre-miR-142a-5p	CATAAAGTAGAAAGCACTACT	down	XLOC_030108	up
PC-3p-11630_419	ATGAGGAAAAGAAGTTAGGAGA	down	XLOC_030163	up
dre-miR-194a_R+2	TGTAACAGCAACTCCATGTGGAT	up	XLOC_030178	up
ssa-miR-16b-5p_R-1_1ss21TC	TAGCAGCACGTAAATATTGGC	down	XLOC_030178	up
tni-miR-194_R+1	TGTAACAGCAACTCCATGTGGA	up	XLOC_030178	up
PC-5p-8690_526	GATGTTGAGTATCAAACTGTAT	down	XLOC_030187	up
PC-5p-27517_164	TACATGCAGAGGTGGAGCAAGA	up	XLOC_030204	up
dre-miR-142a-5p	CATAAAGTAGAAAGCACTACT	down	XLOC_030204	up
ssa-miR-730a-5p_R-1	TCCTCATTGTGCATGCTGTGT	down	XLOC_030253	up
dre-miR-1	TGGAATGTAAAGAAGTATGTAT	up	XLOC_030266	up
ssa-miR-206-3p	TGGAATGTAAGGAAGTGTGTGG	up	XLOC_030266	up
ssc-miR-206	TGGAATGTAAGGAAGTGTGTGA	up	XLOC_030266	up

PC-3p-11630_419	ATGAGGAAAAGAAGTTAGGAGA	down	XLOC_030306	down
dre-miR-1	TGGAATGTAAAGAAGTATGTAT	up	XLOC_030306	down
dre-miR-21_1ss23CA	TAGCTTATCAGACTGGTGTTGGA	up	XLOC_030306	down
ssa-miR-1338-5p_R+1	AGGACTGTCCAACCTGAGAATG	down	XLOC_030306	down
ssa-miR-206-3p	TGGAATGTAAGGAAGTGTGTGG	up	XLOC_030306	down
ssc-miR-206	TGGAATGTAAGGAAGTGTGTGA	up	XLOC_030306	down
ssa-miR-1-4-5p	ACATACTTCTTTATATGCCCATA	up	XLOC_030356	up
dre-miR-125b-5p_R+1	TCCCTGAGACCCTAACTTGTGAT	up	XLOC_030390	down
sha-miR-125a_R+2	TCCCTGAGACCCTAACTTGTGAAA	up	XLOC_030390	down
ssa-miR-125b-5p_R-1	TCCCTGAGACCCTTAACCTGTG	up	XLOC_030390	down
ssa-miR-1338-5p_R+1	AGGACTGTCCAACCTGAGAATG	down	XLOC_030390	down
dre-miR-24_R+2_1	TGGCTCAGTTCAGCAGGAACAGAA	up	XLOC_030482	down
dre-miR-24_R+2_1	TGGCTCAGTTCAGCAGGAACAGAA	up	XLOC_030807	up
dre-miR-24_R+2_2	TGGCTCAGTTCAGCAGGAACAGTT	up	XLOC_030807	up
rno-miR-122-5p_L+3	ATCTGGAGTGTGACAATGGTGTTTG	up	XLOC_030807	up
ssa-miR-16b-5p_R-1_1ss21TC	TAGCAGCACGTAAATATTGGC	down	XLOC_030807	up
dre-miR-125b-5p_R+1	TCCCTGAGACCCTAACTTGTGAT	up	XLOC_030867	up
sha-miR-125a_R+2	TCCCTGAGACCCTAACTTGTGAAA	up	XLOC_030867	up
ssa-miR-125b-5p_R-1	TCCCTGAGACCCTTAACCTGTG	up	XLOC_030867	up
aca-miR-200b-3p_R+2	TAATACTGCCTGGTAATGATGAAT	up	XLOC_031236	up
ola-miR-199a-3p_L+1	AACAGTAGTCTGCACATTGGTTA	up	XLOC_031236	up
ssa-miR-16b-5p_R-1_1ss21TC	TAGCAGCACGTAAATATTGGC	down	XLOC_031236	up
dre-miR-21_1ss23CA	TAGCTTATCAGACTGGTGTTGGA	up	XLOC_031318	down
dre-miR-142a-5p	CATAAAGTAGAAAGCACTACT	down	XLOC_031322	down
ola-miR-199a-3p_L+1	AACAGTAGTCTGCACATTGGTTA	up	XLOC_031322	down
ssa-miR-199a-3p_R+2	ACAGTAGTCTGCACATTGGTTTT	up	XLOC_031322	down
dre-miR-22a-3p	AAGCTGCCAGCTGAAGAACTGT	up	XLOC_031328	down
dre-miR-125b-5p_R+1	TCCCTGAGACCCTAACTTGTGAT	up	XLOC_031335	down
sha-miR-125a_R+2	TCCCTGAGACCCTAACTTGTGAAA	up	XLOC_031335	down
ssa-miR-125b-5p_R-1	TCCCTGAGACCCTTAACCTGTG	up	XLOC_031335	down

Table S10 List of miRNA-mRNA pairs with negative correlation

miR_name	miR_seq	regulation	Accession	regulation
dre-miR-1	TGGAATGTAAAGAAGTATGTAT	up	XLOC_001958	down
dre-miR-1	TGGAATGTAAAGAAGTATGTAT	up	XLOC_003559	down
dre-miR-1	TGGAATGTAAAGAAGTATGTAT	up	XLOC_005515	down
dre-miR-1	TGGAATGTAAAGAAGTATGTAT	up	XLOC_006238	down
dre-miR-1	TGGAATGTAAAGAAGTATGTAT	up	XLOC_006315	down
dre-miR-1	TGGAATGTAAAGAAGTATGTAT	up	XLOC_008633	down
dre-miR-1	TGGAATGTAAAGAAGTATGTAT	up	XLOC_009449	down
dre-miR-1	TGGAATGTAAAGAAGTATGTAT	up	XLOC_010148	down
dre-miR-1	TGGAATGTAAAGAAGTATGTAT	up	XLOC_010187	down
dre-miR-1	TGGAATGTAAAGAAGTATGTAT	up	XLOC_011901	down
dre-miR-1	TGGAATGTAAAGAAGTATGTAT	up	XLOC_011962	down
dre-miR-1	TGGAATGTAAAGAAGTATGTAT	up	XLOC_012298	down
dre-miR-1	TGGAATGTAAAGAAGTATGTAT	up	XLOC_013076	down
dre-miR-1	TGGAATGTAAAGAAGTATGTAT	up	XLOC_014039	down
dre-miR-1	TGGAATGTAAAGAAGTATGTAT	up	XLOC_014096	down
dre-miR-1	TGGAATGTAAAGAAGTATGTAT	up	XLOC_014235	down
dre-miR-1	TGGAATGTAAAGAAGTATGTAT	up	XLOC_015415	down
dre-miR-1	TGGAATGTAAAGAAGTATGTAT	up	XLOC_016046	down
dre-miR-1	TGGAATGTAAAGAAGTATGTAT	up	XLOC_016109	down
dre-miR-1	TGGAATGTAAAGAAGTATGTAT	up	XLOC_016203	down
dre-miR-1	TGGAATGTAAAGAAGTATGTAT	up	XLOC_016810	down
dre-miR-1	TGGAATGTAAAGAAGTATGTAT	up	XLOC_017573	down
dre-miR-1	TGGAATGTAAAGAAGTATGTAT	up	XLOC_018413	down
dre-miR-1	TGGAATGTAAAGAAGTATGTAT	up	XLOC_018735	down
dre-miR-1	TGGAATGTAAAGAAGTATGTAT	up	XLOC_020110	down
dre-miR-1	TGGAATGTAAAGAAGTATGTAT	up	XLOC_023324	down
dre-miR-1	TGGAATGTAAAGAAGTATGTAT	up	XLOC_024276	down
dre-miR-1	TGGAATGTAAAGAAGTATGTAT	up	XLOC_024363	down
dre-miR-1	TGGAATGTAAAGAAGTATGTAT	up	XLOC_028701	down
dre-miR-1	TGGAATGTAAAGAAGTATGTAT	up	XLOC_030306	down
ssa-miR-1338-5p_R+1	AGGACTGTCCAACCTGAGAATG	down	XLOC_001927	up
ssa-miR-1338-5p_R+1	AGGACTGTCCAACCTGAGAATG	down	XLOC_002035	up
ssa-miR-1338-5p_R+1	AGGACTGTCCAACCTGAGAATG	down	XLOC_002231	up
ssa-miR-1338-5p_R+1	AGGACTGTCCAACCTGAGAATG	down	XLOC_004874	up
ssa-miR-1338-5p_R+1	AGGACTGTCCAACCTGAGAATG	down	XLOC_005494	up
ssa-miR-1338-5p_R+1	AGGACTGTCCAACCTGAGAATG	down	XLOC_005765	up
ssa-miR-1338-5p_R+1	AGGACTGTCCAACCTGAGAATG	down	XLOC_006384	up
ssa-miR-1338-5p_R+1	AGGACTGTCCAACCTGAGAATG	down	XLOC_006828	up
ssa-miR-1338-5p_R+1	AGGACTGTCCAACCTGAGAATG	down	XLOC_008709	up
ssa-miR-1338-5p_R+1	AGGACTGTCCAACCTGAGAATG	down	XLOC_009640	up
ssa-miR-1338-5p_R+1	AGGACTGTCCAACCTGAGAATG	down	XLOC_010354	up
ssa-miR-1338-5p_R+1	AGGACTGTCCAACCTGAGAATG	down	XLOC_010979	up
ssa-miR-1338-5p_R+1	AGGACTGTCCAACCTGAGAATG	down	XLOC_012125	up
ssa-miR-1338-5p_R+1	AGGACTGTCCAACCTGAGAATG	down	XLOC_013314	up
ssa-miR-1338-5p_R+1	AGGACTGTCCAACCTGAGAATG	down	XLOC_013988	up
ssa-miR-1338-5p_R+1	AGGACTGTCCAACCTGAGAATG	down	XLOC_014106	up
ssa-miR-1338-5p_R+1	AGGACTGTCCAACCTGAGAATG	down	XLOC_015507	up
ssa-miR-1338-5p_R+1	AGGACTGTCCAACCTGAGAATG	down	XLOC_017359	up
ssa-miR-1338-5p_R+1	AGGACTGTCCAACCTGAGAATG	down	XLOC_019782	up

ssa-miR-1338-5p_R+1	AGGACTGTCCAACCTGAGAATG	down	XLOC_022273	up
ssa-miR-1338-5p_R+1	AGGACTGTCCAACCTGAGAATG	down	XLOC_022924	up
ssa-miR-1338-5p_R+1	AGGACTGTCCAACCTGAGAATG	down	XLOC_023412	up
ssa-miR-1338-5p_R+1	AGGACTGTCCAACCTGAGAATG	down	XLOC_023840	up
ssa-miR-1338-5p_R+1	AGGACTGTCCAACCTGAGAATG	down	XLOC_024250	up
ssa-miR-1338-5p_R+1	AGGACTGTCCAACCTGAGAATG	down	XLOC_027546	up
ssa-miR-16b-5p_R-1_1ss21TC	TAGCAGCACGTAAATATTGGC	down	XLOC_000135	up
ssa-miR-16b-5p_R-1_1ss21TC	TAGCAGCACGTAAATATTGGC	down	XLOC_001181	up
ssa-miR-16b-5p_R-1_1ss21TC	TAGCAGCACGTAAATATTGGC	down	XLOC_001548	up
ssa-miR-16b-5p_R-1_1ss21TC	TAGCAGCACGTAAATATTGGC	down	XLOC_001916	up
ssa-miR-16b-5p_R-1_1ss21TC	TAGCAGCACGTAAATATTGGC	down	XLOC_002434	up
ssa-miR-16b-5p_R-1_1ss21TC	TAGCAGCACGTAAATATTGGC	down	XLOC_002524	up
ssa-miR-16b-5p_R-1_1ss21TC	TAGCAGCACGTAAATATTGGC	down	XLOC_002558	up
ssa-miR-16b-5p_R-1_1ss21TC	TAGCAGCACGTAAATATTGGC	down	XLOC_002651	up
ssa-miR-16b-5p_R-1_1ss21TC	TAGCAGCACGTAAATATTGGC	down	XLOC_003405	up
ssa-miR-16b-5p_R-1_1ss21TC	TAGCAGCACGTAAATATTGGC	down	XLOC_003551	up
ssa-miR-16b-5p_R-1_1ss21TC	TAGCAGCACGTAAATATTGGC	down	XLOC_004184	up
ssa-miR-16b-5p_R-1_1ss21TC	TAGCAGCACGTAAATATTGGC	down	XLOC_004317	up
ssa-miR-16b-5p_R-1_1ss21TC	TAGCAGCACGTAAATATTGGC	down	XLOC_004448	up
ssa-miR-16b-5p_R-1_1ss21TC	TAGCAGCACGTAAATATTGGC	down	XLOC_004605	up
ssa-miR-16b-5p_R-1_1ss21TC	TAGCAGCACGTAAATATTGGC	down	XLOC_004874	up
ssa-miR-16b-5p_R-1_1ss21TC	TAGCAGCACGTAAATATTGGC	down	XLOC_005014	up
ssa-miR-16b-5p_R-1_1ss21TC	TAGCAGCACGTAAATATTGGC	down	XLOC_005065	up
ssa-miR-16b-5p_R-1_1ss21TC	TAGCAGCACGTAAATATTGGC	down	XLOC_005151	up
ssa-miR-16b-5p_R-1_1ss21TC	TAGCAGCACGTAAATATTGGC	down	XLOC_005344	up
ssa-miR-16b-5p_R-1_1ss21TC	TAGCAGCACGTAAATATTGGC	down	XLOC_005432	up
ssa-miR-16b-5p_R-1_1ss21TC	TAGCAGCACGTAAATATTGGC	down	XLOC_005493	up
ssa-miR-16b-5p_R-1_1ss21TC	TAGCAGCACGTAAATATTGGC	down	XLOC_005609	up
ssa-miR-16b-5p_R-1_1ss21TC	TAGCAGCACGTAAATATTGGC	down	XLOC_006178	up
ssa-miR-16b-5p_R-1_1ss21TC	TAGCAGCACGTAAATATTGGC	down	XLOC_006539	up
ssa-miR-16b-5p_R-1_1ss21TC	TAGCAGCACGTAAATATTGGC	down	XLOC_006808	up
ssa-miR-16b-5p_R-1_1ss21TC	TAGCAGCACGTAAATATTGGC	down	XLOC_006921	up
ssa-miR-16b-5p_R-1_1ss21TC	TAGCAGCACGTAAATATTGGC	down	XLOC_007371	up
ssa-miR-16b-5p_R-1_1ss21TC	TAGCAGCACGTAAATATTGGC	down	XLOC_007378	up
ssa-miR-16b-5p_R-1_1ss21TC	TAGCAGCACGTAAATATTGGC	down	XLOC_007806	up
ssa-miR-16b-5p_R-1_1ss21TC	TAGCAGCACGTAAATATTGGC	down	XLOC_008353	up
ssa-miR-16b-5p_R-1_1ss21TC	TAGCAGCACGTAAATATTGGC	down	XLOC_008738	up
ssa-miR-16b-5p_R-1_1ss21TC	TAGCAGCACGTAAATATTGGC	down	XLOC_009126	up
ssa-miR-16b-5p_R-1_1ss21TC	TAGCAGCACGTAAATATTGGC	down	XLOC_009274	up
ssa-miR-16b-5p_R-1_1ss21TC	TAGCAGCACGTAAATATTGGC	down	XLOC_009391	up
ssa-miR-16b-5p_R-1_1ss21TC	TAGCAGCACGTAAATATTGGC	down	XLOC_010155	up
ssa-miR-16b-5p_R-1_1ss21TC	TAGCAGCACGTAAATATTGGC	down	XLOC_010241	up
ssa-miR-16b-5p_R-1_1ss21TC	TAGCAGCACGTAAATATTGGC	down	XLOC_010561	up
ssa-miR-16b-5p_R-1_1ss21TC	TAGCAGCACGTAAATATTGGC	down	XLOC_010583	up
ssa-miR-16b-5p_R-1_1ss21TC	TAGCAGCACGTAAATATTGGC	down	XLOC_011350	up
ssa-miR-16b-5p_R-1_1ss21TC	TAGCAGCACGTAAATATTGGC	down	XLOC_011576	up
ssa-miR-16b-5p_R-1_1ss21TC	TAGCAGCACGTAAATATTGGC	down	XLOC_013948	up
ssa-miR-16b-5p_R-1_1ss21TC	TAGCAGCACGTAAATATTGGC	down	XLOC_015569	up
ssa-miR-16b-5p_R-1_1ss21TC	TAGCAGCACGTAAATATTGGC	down	XLOC_015639	up
ssa-miR-16b-5p_R-1_1ss21TC	TAGCAGCACGTAAATATTGGC	down	XLOC_015721	up
ssa-miR-16b-5p_R-1_1ss21TC	TAGCAGCACGTAAATATTGGC	down	XLOC_015753	up
ssa-miR-16b-5p_R-1_1ss21TC	TAGCAGCACGTAAATATTGGC	down	XLOC_015881	up
ssa-miR-16b-5p_R-1_1ss21TC	TAGCAGCACGTAAATATTGGC	down	XLOC_015887	up
ssa-miR-16b-5p_R-1_1ss21TC	TAGCAGCACGTAAATATTGGC	down	XLOC_015893	up

ssa-miR-16b-5p_R-1_1ss21TC	TAGCAGCACGTAAATATTGGC	down	XLOC_016719	up
ssa-miR-16b-5p_R-1_1ss21TC	TAGCAGCACGTAAATATTGGC	down	XLOC_016918	up
ssa-miR-16b-5p_R-1_1ss21TC	TAGCAGCACGTAAATATTGGC	down	XLOC_016986	up
ssa-miR-16b-5p_R-1_1ss21TC	TAGCAGCACGTAAATATTGGC	down	XLOC_016988	up
ssa-miR-16b-5p_R-1_1ss21TC	TAGCAGCACGTAAATATTGGC	down	XLOC_017359	up
ssa-miR-16b-5p_R-1_1ss21TC	TAGCAGCACGTAAATATTGGC	down	XLOC_017576	up
ssa-miR-16b-5p_R-1_1ss21TC	TAGCAGCACGTAAATATTGGC	down	XLOC_017718	up
ssa-miR-16b-5p_R-1_1ss21TC	TAGCAGCACGTAAATATTGGC	down	XLOC_017992	up
ssa-miR-16b-5p_R-1_1ss21TC	TAGCAGCACGTAAATATTGGC	down	XLOC_018063	up
ssa-miR-16b-5p_R-1_1ss21TC	TAGCAGCACGTAAATATTGGC	down	XLOC_018095	up
ssa-miR-16b-5p_R-1_1ss21TC	TAGCAGCACGTAAATATTGGC	down	XLOC_018488	up
ssa-miR-16b-5p_R-1_1ss21TC	TAGCAGCACGTAAATATTGGC	down	XLOC_018676	up
ssa-miR-16b-5p_R-1_1ss21TC	TAGCAGCACGTAAATATTGGC	down	XLOC_018822	up
ssa-miR-16b-5p_R-1_1ss21TC	TAGCAGCACGTAAATATTGGC	down	XLOC_019044	up
ssa-miR-16b-5p_R-1_1ss21TC	TAGCAGCACGTAAATATTGGC	down	XLOC_019071	up
ssa-miR-16b-5p_R-1_1ss21TC	TAGCAGCACGTAAATATTGGC	down	XLOC_019371	up
ssa-miR-16b-5p_R-1_1ss21TC	TAGCAGCACGTAAATATTGGC	down	XLOC_019580	up
ssa-miR-16b-5p_R-1_1ss21TC	TAGCAGCACGTAAATATTGGC	down	XLOC_019714	up
ssa-miR-16b-5p_R-1_1ss21TC	TAGCAGCACGTAAATATTGGC	down	XLOC_020210	up
ssa-miR-16b-5p_R-1_1ss21TC	TAGCAGCACGTAAATATTGGC	down	XLOC_020649	up
ssa-miR-16b-5p_R-1_1ss21TC	TAGCAGCACGTAAATATTGGC	down	XLOC_021240	up
ssa-miR-16b-5p_R-1_1ss21TC	TAGCAGCACGTAAATATTGGC	down	XLOC_021403	up
ssa-miR-16b-5p_R-1_1ss21TC	TAGCAGCACGTAAATATTGGC	down	XLOC_021827	up
ssa-miR-16b-5p_R-1_1ss21TC	TAGCAGCACGTAAATATTGGC	down	XLOC_022189	up
ssa-miR-16b-5p_R-1_1ss21TC	TAGCAGCACGTAAATATTGGC	down	XLOC_023376	up
ssa-miR-16b-5p_R-1_1ss21TC	TAGCAGCACGTAAATATTGGC	down	XLOC_023458	up
ssa-miR-16b-5p_R-1_1ss21TC	TAGCAGCACGTAAATATTGGC	down	XLOC_023764	up
ssa-miR-16b-5p_R-1_1ss21TC	TAGCAGCACGTAAATATTGGC	down	XLOC_023884	up
ssa-miR-16b-5p_R-1_1ss21TC	TAGCAGCACGTAAATATTGGC	down	XLOC_025234	up
ssa-miR-16b-5p_R-1_1ss21TC	TAGCAGCACGTAAATATTGGC	down	XLOC_027063	up
ssa-miR-16b-5p_R-1_1ss21TC	TAGCAGCACGTAAATATTGGC	down	XLOC_027078	up
ssa-miR-16b-5p_R-1_1ss21TC	TAGCAGCACGTAAATATTGGC	down	XLOC_027931	up
ssa-miR-16b-5p_R-1_1ss21TC	TAGCAGCACGTAAATATTGGC	down	XLOC_028334	up
ssa-miR-16b-5p_R-1_1ss21TC	TAGCAGCACGTAAATATTGGC	down	XLOC_029349	up
ssa-miR-16b-5p_R-1_1ss21TC	TAGCAGCACGTAAATATTGGC	down	XLOC_030178	up
ssa-miR-16b-5p_R-1_1ss21TC	TAGCAGCACGTAAATATTGGC	down	XLOC_030807	up
ssa-miR-16b-5p_R-1_1ss21TC	TAGCAGCACGTAAATATTGGC	down	XLOC_031236	up
dre-miR-142a-5p	CATAAAGTAGAAAGCACTACT	down	XLOC_001006	up
dre-miR-142a-5p	CATAAAGTAGAAAGCACTACT	down	XLOC_001887	up
dre-miR-142a-5p	CATAAAGTAGAAAGCACTACT	down	XLOC_002328	up
dre-miR-142a-5p	CATAAAGTAGAAAGCACTACT	down	XLOC_005493	up
dre-miR-142a-5p	CATAAAGTAGAAAGCACTACT	down	XLOC_006089	up
dre-miR-142a-5p	CATAAAGTAGAAAGCACTACT	down	XLOC_008400	up
dre-miR-142a-5p	CATAAAGTAGAAAGCACTACT	down	XLOC_009274	up
dre-miR-142a-5p	CATAAAGTAGAAAGCACTACT	down	XLOC_009415	up
dre-miR-142a-5p	CATAAAGTAGAAAGCACTACT	down	XLOC_010718	up
dre-miR-142a-5p	CATAAAGTAGAAAGCACTACT	down	XLOC_016118	up
dre-miR-142a-5p	CATAAAGTAGAAAGCACTACT	down	XLOC_016272	up
dre-miR-142a-5p	CATAAAGTAGAAAGCACTACT	down	XLOC_017601	up
dre-miR-142a-5p	CATAAAGTAGAAAGCACTACT	down	XLOC_021240	up
dre-miR-142a-5p	CATAAAGTAGAAAGCACTACT	down	XLOC_023719	up
dre-miR-142a-5p	CATAAAGTAGAAAGCACTACT	down	XLOC_027078	up
dre-miR-142a-5p	CATAAAGTAGAAAGCACTACT	down	XLOC_027166	up
dre-miR-142a-5p	CATAAAGTAGAAAGCACTACT	down	XLOC_029605	up

dre-miR-142a-5p	CATAAAGTAGAAAGCACTACT	down	XLOC_030108	up
dre-miR-142a-5p	CATAAAGTAGAAAGCACTACT	down	XLOC_030204	up
ssa-miR-7132b-5p	GACTTGGTCAAAGCTCCTCAGC	down	XLOC_000922	
ssa-miR-7132b-3p	TGAGGCGTTTAGAACAAGTTCA	down	XLOC_001665	
ssa-miR-7132b-3p	TGAGGCGTTTAGAACAAGTTCA	down	XLOC_005330	
ssa-miR-7132b-5p	GACTTGGTCAAAGCTCCTCAGC	down	XLOC_007243	
ssa-miR-7132b-5p	GACTTGGTCAAAGCTCCTCAGC	down	XLOC_008038	
ssa-miR-7132b-5p	GACTTGGTCAAAGCTCCTCAGC	down	XLOC_008770	
ssa-miR-7132b-5p	GACTTGGTCAAAGCTCCTCAGC	down	XLOC_009640	
ssa-miR-7132b-3p	TGAGGCGTTTAGAACAAGTTCA	down	XLOC_011068	
ssa-miR-7132b-3p	TGAGGCGTTTAGAACAAGTTCA	down	XLOC_011749	
ssa-miR-7132b-3p	TGAGGCGTTTAGAACAAGTTCA	down	XLOC_012011	
ssa-miR-7132b-5p	GACTTGGTCAAAGCTCCTCAGC	down	XLOC_012214	
ssa-miR-7132b-3p	TGAGGCGTTTAGAACAAGTTCA	down	XLOC_012371	
ssa-miR-7132b-5p	GACTTGGTCAAAGCTCCTCAGC	down	XLOC_013252	
ssa-miR-7132b-5p	GACTTGGTCAAAGCTCCTCAGC	down	XLOC_013766	
ssa-miR-7132b-3p	TGAGGCGTTTAGAACAAGTTCA	down	XLOC_013995	
ssa-miR-7132b-5p	GACTTGGTCAAAGCTCCTCAGC	down	XLOC_013995	
ssa-miR-7132b-5p	GACTTGGTCAAAGCTCCTCAGC	down	XLOC_014082	
ssa-miR-7132b-5p	GACTTGGTCAAAGCTCCTCAGC	down	XLOC_014086	
ssa-miR-7132b-3p	TGAGGCGTTTAGAACAAGTTCA	down	XLOC_014301	
ssa-miR-7132b-5p	GACTTGGTCAAAGCTCCTCAGC	down	XLOC_014379	
ssa-miR-7132b-3p	TGAGGCGTTTAGAACAAGTTCA	down	XLOC_014440	
ssa-miR-7132b-5p	GACTTGGTCAAAGCTCCTCAGC	down	XLOC_014525	
ssa-miR-7132b-3p	TGAGGCGTTTAGAACAAGTTCA	down	XLOC_016324	
ssa-miR-7132b-3p	TGAGGCGTTTAGAACAAGTTCA	down	XLOC_016601	
ssa-miR-7132b-5p	GACTTGGTCAAAGCTCCTCAGC	down	XLOC_021403	
ssa-miR-7132b-3p	TGAGGCGTTTAGAACAAGTTCA	down	XLOC_021823	
ssa-miR-7132b-3p	TGAGGCGTTTAGAACAAGTTCA	down	XLOC_022459	
ssa-miR-7132b-3p	TGAGGCGTTTAGAACAAGTTCA	down	XLOC_022706	
ssa-miR-7132b-5p	GACTTGGTCAAAGCTCCTCAGC	down	XLOC_022706	
ssa-miR-7132b-5p	GACTTGGTCAAAGCTCCTCAGC	down	XLOC_023884	
ssa-miR-7132b-5p	GACTTGGTCAAAGCTCCTCAGC	down	XLOC_024324	
ssa-miR-7132b-3p	TGAGGCGTTTAGAACAAGTTCA	down	XLOC_024708	
ssa-miR-7132b-5p	GACTTGGTCAAAGCTCCTCAGC	down	XLOC_025100	
ssa-miR-7132b-5p	GACTTGGTCAAAGCTCCTCAGC	down	XLOC_025234	
ssa-miR-7132b-5p	GACTTGGTCAAAGCTCCTCAGC	down	XLOC_025542	
ssa-miR-7132b-3p	TGAGGCGTTTAGAACAAGTTCA	down	XLOC_025616	
ssa-miR-7132b-3p	TGAGGCGTTTAGAACAAGTTCA	down	XLOC_026607	
ssa-miR-7132b-5p	GACTTGGTCAAAGCTCCTCAGC	down	XLOC_027387	
ssa-miR-7132b-5p	GACTTGGTCAAAGCTCCTCAGC	down	XLOC_030042	
ssa-miR-730a-5p_R-1	TCCTCATTGTGCATGCTGTGT	down	XLOC_001006	up
ssa-miR-730a-5p_R-1	TCCTCATTGTGCATGCTGTGT	down	XLOC_001887	up
ssa-miR-730a-5p_R-1	TCCTCATTGTGCATGCTGTGT	down	XLOC_002035	up
ssa-miR-730a-5p_R-1	TCCTCATTGTGCATGCTGTGT	down	XLOC_002524	up
ssa-miR-730a-5p_R-1	TCCTCATTGTGCATGCTGTGT	down	XLOC_002558	up
ssa-miR-730a-5p_R-1	TCCTCATTGTGCATGCTGTGT	down	XLOC_003632	up
ssa-miR-730a-5p_R-1	TCCTCATTGTGCATGCTGTGT	down	XLOC_004335	up
ssa-miR-730a-5p_R-1	TCCTCATTGTGCATGCTGTGT	down	XLOC_005075	up
ssa-miR-730a-5p_R-1	TCCTCATTGTGCATGCTGTGT	down	XLOC_005140	up
ssa-miR-730a-5p_R-1	TCCTCATTGTGCATGCTGTGT	down	XLOC_005344	up
ssa-miR-730a-5p_R-1	TCCTCATTGTGCATGCTGTGT	down	XLOC_005472	up
ssa-miR-730a-5p_R-1	TCCTCATTGTGCATGCTGTGT	down	XLOC_005533	up
ssa-miR-730a-5p_R-1	TCCTCATTGTGCATGCTGTGT	down	XLOC_006384	up

ssa-miR-730a-5p_R-1	TCCTCATTGTGCATGCTGTGT	down	XLOC_006991	up
ssa-miR-730a-5p_R-1	TCCTCATTGTGCATGCTGTGT	down	XLOC_007242	up
ssa-miR-730a-5p_R-1	TCCTCATTGTGCATGCTGTGT	down	XLOC_008304	up
ssa-miR-730a-5p_R-1	TCCTCATTGTGCATGCTGTGT	down	XLOC_008552	up
ssa-miR-730a-5p_R-1	TCCTCATTGTGCATGCTGTGT	down	XLOC_009408	up
ssa-miR-730a-5p_R-1	TCCTCATTGTGCATGCTGTGT	down	XLOC_009640	up
ssa-miR-730a-5p_R-1	TCCTCATTGTGCATGCTGTGT	down	XLOC_010940	up
ssa-miR-730a-5p_R-1	TCCTCATTGTGCATGCTGTGT	down	XLOC_011910	up
ssa-miR-730a-5p_R-1	TCCTCATTGTGCATGCTGTGT	down	XLOC_012510	up
ssa-miR-730a-5p_R-1	TCCTCATTGTGCATGCTGTGT	down	XLOC_013314	up
ssa-miR-730a-5p_R-1	TCCTCATTGTGCATGCTGTGT	down	XLOC_013995	up
ssa-miR-730a-5p_R-1	TCCTCATTGTGCATGCTGTGT	down	XLOC_014106	up
ssa-miR-730a-5p_R-1	TCCTCATTGTGCATGCTGTGT	down	XLOC_014224	up
ssa-miR-730a-5p_R-1	TCCTCATTGTGCATGCTGTGT	down	XLOC_014905	up
ssa-miR-730a-5p_R-1	TCCTCATTGTGCATGCTGTGT	down	XLOC_015536	up
ssa-miR-730a-5p_R-1	TCCTCATTGTGCATGCTGTGT	down	XLOC_016270	up
ssa-miR-730a-5p_R-1	TCCTCATTGTGCATGCTGTGT	down	XLOC_016918	up
ssa-miR-730a-5p_R-1	TCCTCATTGTGCATGCTGTGT	down	XLOC_017163	up
ssa-miR-730a-5p_R-1	TCCTCATTGTGCATGCTGTGT	down	XLOC_017601	up
ssa-miR-730a-5p_R-1	TCCTCATTGTGCATGCTGTGT	down	XLOC_017962	up
ssa-miR-730a-5p_R-1	TCCTCATTGTGCATGCTGTGT	down	XLOC_019130	up
ssa-miR-730a-5p_R-1	TCCTCATTGTGCATGCTGTGT	down	XLOC_019198	up
ssa-miR-730a-5p_R-1	TCCTCATTGTGCATGCTGTGT	down	XLOC_019580	up
ssa-miR-730a-5p_R-1	TCCTCATTGTGCATGCTGTGT	down	XLOC_020127	up
ssa-miR-730a-5p_R-1	TCCTCATTGTGCATGCTGTGT	down	XLOC_020796	up
ssa-miR-730a-5p_R-1	TCCTCATTGTGCATGCTGTGT	down	XLOC_021020	up
ssa-miR-730a-5p_R-1	TCCTCATTGTGCATGCTGTGT	down	XLOC_021480	up
ssa-miR-730a-5p_R-1	TCCTCATTGTGCATGCTGTGT	down	XLOC_022903	up
ssa-miR-730a-5p_R-1	TCCTCATTGTGCATGCTGTGT	down	XLOC_027166	up
ssa-miR-730a-5p_R-1	TCCTCATTGTGCATGCTGTGT	down	XLOC_030040	up
ssa-miR-730a-5p_R-1	TCCTCATTGTGCATGCTGTGT	down	XLOC_030253	up
ssa-miR-26a-4-3p	CCTATTCTTGATTACTTGTTTC	down	XLOC_000922	up
ssa-miR-26a-4-3p	CCTATTCTTGATTACTTGTTTC	down	XLOC_002434	up
ssa-miR-26a-4-3p	CCTATTCTTGATTACTTGTTTC	down	XLOC_005330	up
ssa-miR-26a-4-3p	CCTATTCTTGATTACTTGTTTC	down	XLOC_007378	up
ssa-miR-26a-4-3p	CCTATTCTTGATTACTTGTTTC	down	XLOC_008353	up
ssa-miR-26a-4-3p	CCTATTCTTGATTACTTGTTTC	down	XLOC_010523	up
ssa-miR-26a-4-3p	CCTATTCTTGATTACTTGTTTC	down	XLOC_010561	up
ssa-miR-26a-4-3p	CCTATTCTTGATTACTTGTTTC	down	XLOC_010979	up
ssa-miR-26a-4-3p	CCTATTCTTGATTACTTGTTTC	down	XLOC_011438	up
ssa-miR-26a-4-3p	CCTATTCTTGATTACTTGTTTC	down	XLOC_012689	up
ssa-miR-26a-4-3p	CCTATTCTTGATTACTTGTTTC	down	XLOC_013129	up
ssa-miR-26a-4-3p	CCTATTCTTGATTACTTGTTTC	down	XLOC_013995	up
ssa-miR-26a-4-3p	CCTATTCTTGATTACTTGTTTC	down	XLOC_016271	up
ssa-miR-26a-4-3p	CCTATTCTTGATTACTTGTTTC	down	XLOC_017188	up
ssa-miR-26a-4-3p	CCTATTCTTGATTACTTGTTTC	down	XLOC_017214	up
ssa-miR-26a-4-3p	CCTATTCTTGATTACTTGTTTC	down	XLOC_017986	up
ssa-miR-26a-4-3p	CCTATTCTTGATTACTTGTTTC	down	XLOC_018819	up
ssa-miR-26a-4-3p	CCTATTCTTGATTACTTGTTTC	down	XLOC_022351	up
ssa-miR-26a-4-3p	CCTATTCTTGATTACTTGTTTC	down	XLOC_023764	up
ssa-miR-26a-4-3p	CCTATTCTTGATTACTTGTTTC	down	XLOC_023785	up
ssa-miR-26a-4-3p	CCTATTCTTGATTACTTGTTTC	down	XLOC_024681	up
ssa-miR-26a-4-3p	CCTATTCTTGATTACTTGTTTC	down	XLOC_028538	up
ssa-mir-15c-2-p3_1ss11CA	TGCGAACCATAATTTGCTGCTT	down	XLOC_020919	up

PC-3p-11630_419	ATGAGGAAAAGAAGTTAGGAGA	down	XLOC_002262	up
PC-3p-11630_419	ATGAGGAAAAGAAGTTAGGAGA	down	XLOC_002283	up
PC-3p-11630_419	ATGAGGAAAAGAAGTTAGGAGA	down	XLOC_002328	up
PC-3p-11630_419	ATGAGGAAAAGAAGTTAGGAGA	down	XLOC_002702	up
PC-3p-11630_419	ATGAGGAAAAGAAGTTAGGAGA	down	XLOC_002864	up
PC-3p-11630_419	ATGAGGAAAAGAAGTTAGGAGA	down	XLOC_003795	up
PC-3p-11630_419	ATGAGGAAAAGAAGTTAGGAGA	down	XLOC_005330	up
PC-3p-11630_419	ATGAGGAAAAGAAGTTAGGAGA	down	XLOC_006125	up
PC-3p-11630_419	ATGAGGAAAAGAAGTTAGGAGA	down	XLOC_006147	up
PC-3p-11630_419	ATGAGGAAAAGAAGTTAGGAGA	down	XLOC_006319	up
PC-3p-11630_419	ATGAGGAAAAGAAGTTAGGAGA	down	XLOC_006828	up
PC-3p-11630_419	ATGAGGAAAAGAAGTTAGGAGA	down	XLOC_006883	up
PC-3p-11630_419	ATGAGGAAAAGAAGTTAGGAGA	down	XLOC_007002	up
PC-3p-11630_419	ATGAGGAAAAGAAGTTAGGAGA	down	XLOC_007246	up
PC-3p-11630_419	ATGAGGAAAAGAAGTTAGGAGA	down	XLOC_007716	up
PC-3p-11630_419	ATGAGGAAAAGAAGTTAGGAGA	down	XLOC_009640	up
PC-3p-11630_419	ATGAGGAAAAGAAGTTAGGAGA	down	XLOC_010068	up
PC-3p-11630_419	ATGAGGAAAAGAAGTTAGGAGA	down	XLOC_010881	up
PC-3p-11630_419	ATGAGGAAAAGAAGTTAGGAGA	down	XLOC_011454	up
PC-3p-11630_419	ATGAGGAAAAGAAGTTAGGAGA	down	XLOC_012371	up
PC-3p-11630_419	ATGAGGAAAAGAAGTTAGGAGA	down	XLOC_012689	up
PC-3p-11630_419	ATGAGGAAAAGAAGTTAGGAGA	down	XLOC_014224	up
PC-3p-11630_419	ATGAGGAAAAGAAGTTAGGAGA	down	XLOC_014820	up
PC-3p-11630_419	ATGAGGAAAAGAAGTTAGGAGA	down	XLOC_015507	up
PC-3p-11630_419	ATGAGGAAAAGAAGTTAGGAGA	down	XLOC_015753	up
PC-3p-11630_419	ATGAGGAAAAGAAGTTAGGAGA	down	XLOC_016152	up
PC-3p-11630_419	ATGAGGAAAAGAAGTTAGGAGA	down	XLOC_017359	up
PC-3p-11630_419	ATGAGGAAAAGAAGTTAGGAGA	down	XLOC_017878	up
PC-3p-11630_419	ATGAGGAAAAGAAGTTAGGAGA	down	XLOC_018493	up
PC-3p-11630_419	ATGAGGAAAAGAAGTTAGGAGA	down	XLOC_019664	up
PC-3p-11630_419	ATGAGGAAAAGAAGTTAGGAGA	down	XLOC_020433	up
PC-3p-11630_419	ATGAGGAAAAGAAGTTAGGAGA	down	XLOC_021020	up
PC-3p-11630_419	ATGAGGAAAAGAAGTTAGGAGA	down	XLOC_023602	up
PC-3p-11630_419	ATGAGGAAAAGAAGTTAGGAGA	down	XLOC_023719	up
PC-3p-11630_419	ATGAGGAAAAGAAGTTAGGAGA	down	XLOC_023929	up
PC-3p-11630_419	ATGAGGAAAAGAAGTTAGGAGA	down	XLOC_024708	up
PC-3p-11630_419	ATGAGGAAAAGAAGTTAGGAGA	down	XLOC_024853	up
PC-3p-11630_419	ATGAGGAAAAGAAGTTAGGAGA	down	XLOC_025440	up
PC-3p-11630_419	ATGAGGAAAAGAAGTTAGGAGA	down	XLOC_026248	up
PC-3p-11630_419	ATGAGGAAAAGAAGTTAGGAGA	down	XLOC_027531	up
PC-3p-11630_419	ATGAGGAAAAGAAGTTAGGAGA	down	XLOC_029349	up
PC-3p-11630_419	ATGAGGAAAAGAAGTTAGGAGA	down	XLOC_030163	up
ola-miR-462_L-1R+4	TAACGGAACCCATAATGCAGCT	down	XLOC_001104	up
ola-miR-462_L-1R+4	TAACGGAACCCATAATGCAGCT	down	XLOC_001321	up
ola-miR-462_L-1R+4	TAACGGAACCCATAATGCAGCT	down	XLOC_004497	up
ola-miR-462_L-1R+4	TAACGGAACCCATAATGCAGCT	down	XLOC_006319	up
ola-miR-462_L-1R+4	TAACGGAACCCATAATGCAGCT	down	XLOC_008738	up
ola-miR-462_L-1R+4	TAACGGAACCCATAATGCAGCT	down	XLOC_013572	up
ola-miR-462_L-1R+4	TAACGGAACCCATAATGCAGCT	down	XLOC_013988	up
ola-miR-462_L-1R+4	TAACGGAACCCATAATGCAGCT	down	XLOC_019782	up
ola-miR-462_L-1R+4	TAACGGAACCCATAATGCAGCT	down	XLOC_024207	up
ola-miR-462_L-1R+4	TAACGGAACCCATAATGCAGCT	down	XLOC_024986	up
ola-miR-462_L-1R+4	TAACGGAACCCATAATGCAGCT	down	XLOC_026195	up
dre-miR-22a-3p	AAGCTGCCAGCTGAAGAACTGT	up	XLOC_000321	down

dre-miR-22a-3p	AAGCTGCCAGCTGAAGAACTGT	up	XLOC_000634	down
dre-miR-22a-3p	AAGCTGCCAGCTGAAGAACTGT	up	XLOC_001144	down
dre-miR-22a-3p	AAGCTGCCAGCTGAAGAACTGT	up	XLOC_001289	down
dre-miR-22a-3p	AAGCTGCCAGCTGAAGAACTGT	up	XLOC_002244	down
dre-miR-22a-3p	AAGCTGCCAGCTGAAGAACTGT	up	XLOC_002478	down
dre-miR-22a-3p	AAGCTGCCAGCTGAAGAACTGT	up	XLOC_006372	down
dre-miR-22a-3p	AAGCTGCCAGCTGAAGAACTGT	up	XLOC_006751	down
dre-miR-22a-3p	AAGCTGCCAGCTGAAGAACTGT	up	XLOC_008191	down
dre-miR-22a-3p	AAGCTGCCAGCTGAAGAACTGT	up	XLOC_009011	down
dre-miR-22a-3p	AAGCTGCCAGCTGAAGAACTGT	up	XLOC_009105	down
dre-miR-22a-3p	AAGCTGCCAGCTGAAGAACTGT	up	XLOC_009879	down
dre-miR-22a-3p	AAGCTGCCAGCTGAAGAACTGT	up	XLOC_010187	down
dre-miR-22a-3p	AAGCTGCCAGCTGAAGAACTGT	up	XLOC_011235	down
dre-miR-22a-3p	AAGCTGCCAGCTGAAGAACTGT	up	XLOC_011853	down
dre-miR-22a-3p	AAGCTGCCAGCTGAAGAACTGT	up	XLOC_013175	down
dre-miR-22a-3p	AAGCTGCCAGCTGAAGAACTGT	up	XLOC_013512	down
dre-miR-22a-3p	AAGCTGCCAGCTGAAGAACTGT	up	XLOC_013910	down
dre-miR-22a-3p	AAGCTGCCAGCTGAAGAACTGT	up	XLOC_014096	down
dre-miR-22a-3p	AAGCTGCCAGCTGAAGAACTGT	up	XLOC_014319	down
dre-miR-22a-3p	AAGCTGCCAGCTGAAGAACTGT	up	XLOC_014997	down
dre-miR-22a-3p	AAGCTGCCAGCTGAAGAACTGT	up	XLOC_015371	down
dre-miR-22a-3p	AAGCTGCCAGCTGAAGAACTGT	up	XLOC_016049	down
dre-miR-22a-3p	AAGCTGCCAGCTGAAGAACTGT	up	XLOC_016187	down
dre-miR-22a-3p	AAGCTGCCAGCTGAAGAACTGT	up	XLOC_016826	down
dre-miR-22a-3p	AAGCTGCCAGCTGAAGAACTGT	up	XLOC_017060	down
dre-miR-22a-3p	AAGCTGCCAGCTGAAGAACTGT	up	XLOC_017979	down
dre-miR-22a-3p	AAGCTGCCAGCTGAAGAACTGT	up	XLOC_018118	down
dre-miR-22a-3p	AAGCTGCCAGCTGAAGAACTGT	up	XLOC_019708	down
dre-miR-22a-3p	AAGCTGCCAGCTGAAGAACTGT	up	XLOC_020283	down
dre-miR-22a-3p	AAGCTGCCAGCTGAAGAACTGT	up	XLOC_021608	down
dre-miR-22a-3p	AAGCTGCCAGCTGAAGAACTGT	up	XLOC_023109	down
dre-miR-22a-3p	AAGCTGCCAGCTGAAGAACTGT	up	XLOC_023619	down
dre-miR-22a-3p	AAGCTGCCAGCTGAAGAACTGT	up	XLOC_024238	down
dre-miR-22a-3p	AAGCTGCCAGCTGAAGAACTGT	up	XLOC_024276	down
dre-miR-22a-3p	AAGCTGCCAGCTGAAGAACTGT	up	XLOC_025873	down
dre-miR-22a-3p	AAGCTGCCAGCTGAAGAACTGT	up	XLOC_027484	down
dre-miR-22a-3p	AAGCTGCCAGCTGAAGAACTGT	up	XLOC_027914	down
dre-miR-22a-3p	AAGCTGCCAGCTGAAGAACTGT	up	XLOC_031328	down
dre-miR-133a-3p_L-1R+1	TTGGTCCCCTTCAACCAGCTGT	up	XLOC_000321	down
dre-miR-133a-3p_L-1R+1	TTGGTCCCCTTCAACCAGCTGT	up	XLOC_002020	down
dre-miR-133a-3p_L-1R+1	TTGGTCCCCTTCAACCAGCTGT	up	XLOC_004628	down
dre-miR-133a-3p_L-1R+1	TTGGTCCCCTTCAACCAGCTGT	up	XLOC_006096	down
dre-miR-133a-3p_L-1R+1	TTGGTCCCCTTCAACCAGCTGT	up	XLOC_006315	down
dre-miR-133a-3p_L-1R+1	TTGGTCCCCTTCAACCAGCTGT	up	XLOC_010820	down
dre-miR-133a-3p_L-1R+1	TTGGTCCCCTTCAACCAGCTGT	up	XLOC_013076	down
dre-miR-133a-3p_L-1R+1	TTGGTCCCCTTCAACCAGCTGT	up	XLOC_015248	down
dre-miR-133a-3p_L-1R+1	TTGGTCCCCTTCAACCAGCTGT	up	XLOC_015955	down
dre-miR-133a-3p_L-1R+1	TTGGTCCCCTTCAACCAGCTGT	up	XLOC_018422	down
dre-miR-133a-3p_L-1R+1	TTGGTCCCCTTCAACCAGCTGT	up	XLOC_022740	down
dre-miR-133a-3p_L-1R+1	TTGGTCCCCTTCAACCAGCTGT	up	XLOC_024325	down
dre-miR-133a-3p_L-1R+1	TTGGTCCCCTTCAACCAGCTGT	up	XLOC_025586	down
dre-miR-133a-3p_L-1R+1	TTGGTCCCCTTCAACCAGCTGT	up	XLOC_027534	down
dre-miR-133a-3p_L-1R+1	TTGGTCCCCTTCAACCAGCTGT	up	XLOC_029635	down
mmu-let-7j_1ss8TG	TGAGGTAGTAGTTTGTGCTGTTAT	up	XLOC_000321	down

mmu-let-7j_1ss8TG	TGAGGTAGTAGTTTGTGCTGTTAT	up	XLOC_005833	down
mmu-let-7j_1ss8TG	TGAGGTAGTAGTTTGTGCTGTTAT	up	XLOC_005994	down
mmu-let-7j_1ss8TG	TGAGGTAGTAGTTTGTGCTGTTAT	up	XLOC_007042	down
mmu-let-7j_1ss8TG	TGAGGTAGTAGTTTGTGCTGTTAT	up	XLOC_007650	down
mmu-let-7j_1ss8TG	TGAGGTAGTAGTTTGTGCTGTTAT	up	XLOC_010248	down
mmu-let-7j_1ss8TG	TGAGGTAGTAGTTTGTGCTGTTAT	up	XLOC_010643	down
mmu-let-7j_1ss8TG	TGAGGTAGTAGTTTGTGCTGTTAT	up	XLOC_012393	down
mmu-let-7j_1ss8TG	TGAGGTAGTAGTTTGTGCTGTTAT	up	XLOC_016514	down
mmu-let-7j_1ss8TG	TGAGGTAGTAGTTTGTGCTGTTAT	up	XLOC_018735	down
mmu-let-7j_1ss8TG	TGAGGTAGTAGTTTGTGCTGTTAT	up	XLOC_018933	down
mmu-let-7j_1ss8TG	TGAGGTAGTAGTTTGTGCTGTTAT	up	XLOC_019902	down
mmu-let-7j_1ss8TG	TGAGGTAGTAGTTTGTGCTGTTAT	up	XLOC_021608	down
mmu-let-7j_1ss8TG	TGAGGTAGTAGTTTGTGCTGTTAT	up	XLOC_024627	down
mmu-let-7j_1ss8TG	TGAGGTAGTAGTTTGTGCTGTTAT	up	XLOC_027991	down
mmu-let-7j_1ss8TG	TGAGGTAGTAGTTTGTGCTGTTAT	up	XLOC_028496	down
mmu-let-7j_1ss8TG	TGAGGTAGTAGTTTGTGCTGTTAT	up	XLOC_028699	down
ola-mir-100-2-p3	CAAGCTCGTATCTATAGGTATG	down	XLOC_001006	up
ola-mir-100-2-p3	CAAGCTCGTATCTATAGGTATG	down	XLOC_001641	up
ola-mir-100-2-p3	CAAGCTCGTATCTATAGGTATG	down	XLOC_007371	up
ola-mir-100-2-p3	CAAGCTCGTATCTATAGGTATG	down	XLOC_008378	up
ola-mir-100-2-p3	CAAGCTCGTATCTATAGGTATG	down	XLOC_009229	up
ola-mir-100-2-p3	CAAGCTCGTATCTATAGGTATG	down	XLOC_011454	up
ola-mir-100-2-p3	CAAGCTCGTATCTATAGGTATG	down	XLOC_012871	up
ola-mir-100-2-p3	CAAGCTCGTATCTATAGGTATG	down	XLOC_016324	up
ola-mir-100-2-p3	CAAGCTCGTATCTATAGGTATG	down	XLOC_019123	up
ola-mir-100-2-p3	CAAGCTCGTATCTATAGGTATG	down	XLOC_019782	up
ola-mir-100-2-p3	CAAGCTCGTATCTATAGGTATG	down	XLOC_022706	up
ola-mir-100-2-p3	CAAGCTCGTATCTATAGGTATG	down	XLOC_025841	up
dre-miR-125b-5p_R+1	TCCCTGAGACCCTAACTTGTGAT	up	XLOC_002645	down
dre-miR-125b-5p_R+1	TCCCTGAGACCCTAACTTGTGAT	up	XLOC_003836	down
dre-miR-125b-5p_R+1	TCCCTGAGACCCTAACTTGTGAT	up	XLOC_007080	down
dre-miR-125b-5p_R+1	TCCCTGAGACCCTAACTTGTGAT	up	XLOC_008186	down
dre-miR-125b-5p_R+1	TCCCTGAGACCCTAACTTGTGAT	up	XLOC_009011	down
dre-miR-125b-5p_R+1	TCCCTGAGACCCTAACTTGTGAT	up	XLOC_009175	down
dre-miR-125b-5p_R+1	TCCCTGAGACCCTAACTTGTGAT	up	XLOC_009429	down
dre-miR-125b-5p_R+1	TCCCTGAGACCCTAACTTGTGAT	up	XLOC_010315	down
dre-miR-125b-5p_R+1	TCCCTGAGACCCTAACTTGTGAT	up	XLOC_010323	down
dre-miR-125b-5p_R+1	TCCCTGAGACCCTAACTTGTGAT	up	XLOC_010710	down
dre-miR-125b-5p_R+1	TCCCTGAGACCCTAACTTGTGAT	up	XLOC_012890	down
dre-miR-125b-5p_R+1	TCCCTGAGACCCTAACTTGTGAT	up	XLOC_013583	down
dre-miR-125b-5p_R+1	TCCCTGAGACCCTAACTTGTGAT	up	XLOC_014319	down
dre-miR-125b-5p_R+1	TCCCTGAGACCCTAACTTGTGAT	up	XLOC_014884	down
dre-miR-125b-5p_R+1	TCCCTGAGACCCTAACTTGTGAT	up	XLOC_016049	down
dre-miR-125b-5p_R+1	TCCCTGAGACCCTAACTTGTGAT	up	XLOC_016211	down
dre-miR-125b-5p_R+1	TCCCTGAGACCCTAACTTGTGAT	up	XLOC_016923	down
dre-miR-125b-5p_R+1	TCCCTGAGACCCTAACTTGTGAT	up	XLOC_018422	down
dre-miR-125b-5p_R+1	TCCCTGAGACCCTAACTTGTGAT	up	XLOC_019470	down
dre-miR-125b-5p_R+1	TCCCTGAGACCCTAACTTGTGAT	up	XLOC_019789	down
dre-miR-125b-5p_R+1	TCCCTGAGACCCTAACTTGTGAT	up	XLOC_024238	down
dre-miR-125b-5p_R+1	TCCCTGAGACCCTAACTTGTGAT	up	XLOC_025586	down
dre-miR-125b-5p_R+1	TCCCTGAGACCCTAACTTGTGAT	up	XLOC_027524	down
dre-miR-125b-5p_R+1	TCCCTGAGACCCTAACTTGTGAT	up	XLOC_030390	down
dre-miR-125b-5p_R+1	TCCCTGAGACCCTAACTTGTGAT	up	XLOC_031335	down
sha-miR-125a_R+2	TCCCTGAGACCCTAACTTGTGAAA	up	XLOC_002645	down

sha-miR-125a_R+2	TCCCTGAGACCCTAACTTGTGAAA	up	XLOC_003836	down
sha-miR-125a_R+2	TCCCTGAGACCCTAACTTGTGAAA	up	XLOC_007080	down
sha-miR-125a_R+2	TCCCTGAGACCCTAACTTGTGAAA	up	XLOC_008186	down
sha-miR-125a_R+2	TCCCTGAGACCCTAACTTGTGAAA	up	XLOC_009011	down
sha-miR-125a_R+2	TCCCTGAGACCCTAACTTGTGAAA	up	XLOC_009175	down
sha-miR-125a_R+2	TCCCTGAGACCCTAACTTGTGAAA	up	XLOC_009429	down
sha-miR-125a_R+2	TCCCTGAGACCCTAACTTGTGAAA	up	XLOC_010315	down
sha-miR-125a_R+2	TCCCTGAGACCCTAACTTGTGAAA	up	XLOC_010323	down
sha-miR-125a_R+2	TCCCTGAGACCCTAACTTGTGAAA	up	XLOC_010710	down
sha-miR-125a_R+2	TCCCTGAGACCCTAACTTGTGAAA	up	XLOC_012890	down
sha-miR-125a_R+2	TCCCTGAGACCCTAACTTGTGAAA	up	XLOC_013583	down
sha-miR-125a_R+2	TCCCTGAGACCCTAACTTGTGAAA	up	XLOC_014319	down
sha-miR-125a_R+2	TCCCTGAGACCCTAACTTGTGAAA	up	XLOC_014884	down
sha-miR-125a_R+2	TCCCTGAGACCCTAACTTGTGAAA	up	XLOC_016049	down
sha-miR-125a_R+2	TCCCTGAGACCCTAACTTGTGAAA	up	XLOC_016211	down
sha-miR-125a_R+2	TCCCTGAGACCCTAACTTGTGAAA	up	XLOC_016923	down
sha-miR-125a_R+2	TCCCTGAGACCCTAACTTGTGAAA	up	XLOC_018422	down
sha-miR-125a_R+2	TCCCTGAGACCCTAACTTGTGAAA	up	XLOC_019470	down
sha-miR-125a_R+2	TCCCTGAGACCCTAACTTGTGAAA	up	XLOC_019789	down
sha-miR-125a_R+2	TCCCTGAGACCCTAACTTGTGAAA	up	XLOC_024238	down
sha-miR-125a_R+2	TCCCTGAGACCCTAACTTGTGAAA	up	XLOC_025586	down
sha-miR-125a_R+2	TCCCTGAGACCCTAACTTGTGAAA	up	XLOC_027524	down
sha-miR-125a_R+2	TCCCTGAGACCCTAACTTGTGAAA	up	XLOC_030390	down
sha-miR-125a_R+2	TCCCTGAGACCCTAACTTGTGAAA	up	XLOC_031335	down
dre-let-7d-5p	TGAGGTAGTTGGTTGTATGGTT	up	XLOC_000321	down
dre-let-7d-5p	TGAGGTAGTTGGTTGTATGGTT	up	XLOC_005833	down
dre-let-7d-5p	TGAGGTAGTTGGTTGTATGGTT	up	XLOC_005994	down
dre-let-7d-5p	TGAGGTAGTTGGTTGTATGGTT	up	XLOC_007042	down
dre-let-7d-5p	TGAGGTAGTTGGTTGTATGGTT	up	XLOC_009381	down
dre-let-7d-5p	TGAGGTAGTTGGTTGTATGGTT	up	XLOC_010248	down
dre-let-7d-5p	TGAGGTAGTTGGTTGTATGGTT	up	XLOC_010643	down
dre-let-7d-5p	TGAGGTAGTTGGTTGTATGGTT	up	XLOC_016514	down
dre-let-7d-5p	TGAGGTAGTTGGTTGTATGGTT	up	XLOC_018735	down
dre-let-7d-5p	TGAGGTAGTTGGTTGTATGGTT	up	XLOC_018933	down
dre-let-7d-5p	TGAGGTAGTTGGTTGTATGGTT	up	XLOC_019902	down
dre-let-7d-5p	TGAGGTAGTTGGTTGTATGGTT	up	XLOC_021608	down
dre-let-7d-5p	TGAGGTAGTTGGTTGTATGGTT	up	XLOC_026204	down
dre-let-7d-5p	TGAGGTAGTTGGTTGTATGGTT	up	XLOC_027991	down
dre-let-7d-5p	TGAGGTAGTTGGTTGTATGGTT	up	XLOC_028496	down
dre-let-7d-5p	TGAGGTAGTTGGTTGTATGGTT	up	XLOC_028699	down
PC-5p-27517_164	TACATGCAGAGGTGGAGCAAGA	up	XLOC_001106	down
PC-5p-27517_164	TACATGCAGAGGTGGAGCAAGA	up	XLOC_001134	down
PC-5p-27517_164	TACATGCAGAGGTGGAGCAAGA	up	XLOC_001360	down
PC-5p-27517_164	TACATGCAGAGGTGGAGCAAGA	up	XLOC_001958	down
PC-5p-27517_164	TACATGCAGAGGTGGAGCAAGA	up	XLOC_002240	down
PC-5p-27517_164	TACATGCAGAGGTGGAGCAAGA	up	XLOC_002479	down
PC-5p-27517_164	TACATGCAGAGGTGGAGCAAGA	up	XLOC_002781	down
PC-5p-27517_164	TACATGCAGAGGTGGAGCAAGA	up	XLOC_003736	down
PC-5p-27517_164	TACATGCAGAGGTGGAGCAAGA	up	XLOC_004733	down
PC-5p-27517_164	TACATGCAGAGGTGGAGCAAGA	up	XLOC_004775	down
PC-5p-27517_164	TACATGCAGAGGTGGAGCAAGA	up	XLOC_005060	down
PC-5p-27517_164	TACATGCAGAGGTGGAGCAAGA	up	XLOC_005372	down
PC-5p-27517_164	TACATGCAGAGGTGGAGCAAGA	up	XLOC_006315	down
PC-5p-27517_164	TACATGCAGAGGTGGAGCAAGA	up	XLOC_007180	down

PC-5p-27517_164	TACATGCAGAGGTGGAGCAAGA	up	XLOC_007650	down
PC-5p-27517_164	TACATGCAGAGGTGGAGCAAGA	up	XLOC_009111	down
PC-5p-27517_164	TACATGCAGAGGTGGAGCAAGA	up	XLOC_009429	down
PC-5p-27517_164	TACATGCAGAGGTGGAGCAAGA	up	XLOC_009814	down
PC-5p-27517_164	TACATGCAGAGGTGGAGCAAGA	up	XLOC_010014	down
PC-5p-27517_164	TACATGCAGAGGTGGAGCAAGA	up	XLOC_010248	down
PC-5p-27517_164	TACATGCAGAGGTGGAGCAAGA	up	XLOC_010323	down
PC-5p-27517_164	TACATGCAGAGGTGGAGCAAGA	up	XLOC_010501	down
PC-5p-27517_164	TACATGCAGAGGTGGAGCAAGA	up	XLOC_011644	down
PC-5p-27517_164	TACATGCAGAGGTGGAGCAAGA	up	XLOC_011774	down
PC-5p-27517_164	TACATGCAGAGGTGGAGCAAGA	up	XLOC_011853	down
PC-5p-27517_164	TACATGCAGAGGTGGAGCAAGA	up	XLOC_012130	down
PC-5p-27517_164	TACATGCAGAGGTGGAGCAAGA	up	XLOC_012201	down
PC-5p-27517_164	TACATGCAGAGGTGGAGCAAGA	up	XLOC_012393	down
PC-5p-27517_164	TACATGCAGAGGTGGAGCAAGA	up	XLOC_012683	down
PC-5p-27517_164	TACATGCAGAGGTGGAGCAAGA	up	XLOC_012833	down
PC-5p-27517_164	TACATGCAGAGGTGGAGCAAGA	up	XLOC_013187	down
PC-5p-27517_164	TACATGCAGAGGTGGAGCAAGA	up	XLOC_013780	down
PC-5p-27517_164	TACATGCAGAGGTGGAGCAAGA	up	XLOC_014079	down
PC-5p-27517_164	TACATGCAGAGGTGGAGCAAGA	up	XLOC_014376	down
PC-5p-27517_164	TACATGCAGAGGTGGAGCAAGA	up	XLOC_014607	down
PC-5p-27517_164	TACATGCAGAGGTGGAGCAAGA	up	XLOC_015336	down
PC-5p-27517_164	TACATGCAGAGGTGGAGCAAGA	up	XLOC_016109	down
PC-5p-27517_164	TACATGCAGAGGTGGAGCAAGA	up	XLOC_016185	down
PC-5p-27517_164	TACATGCAGAGGTGGAGCAAGA	up	XLOC_016923	down
PC-5p-27517_164	TACATGCAGAGGTGGAGCAAGA	up	XLOC_017111	down
PC-5p-27517_164	TACATGCAGAGGTGGAGCAAGA	up	XLOC_017327	down
PC-5p-27517_164	TACATGCAGAGGTGGAGCAAGA	up	XLOC_017411	down
PC-5p-27517_164	TACATGCAGAGGTGGAGCAAGA	up	XLOC_017678	down
PC-5p-27517_164	TACATGCAGAGGTGGAGCAAGA	up	XLOC_018298	down
PC-5p-27517_164	TACATGCAGAGGTGGAGCAAGA	up	XLOC_018523	down
PC-5p-27517_164	TACATGCAGAGGTGGAGCAAGA	up	XLOC_018678	down
PC-5p-27517_164	TACATGCAGAGGTGGAGCAAGA	up	XLOC_018983	down
PC-5p-27517_164	TACATGCAGAGGTGGAGCAAGA	up	XLOC_019659	down
PC-5p-27517_164	TACATGCAGAGGTGGAGCAAGA	up	XLOC_020931	down
PC-5p-27517_164	TACATGCAGAGGTGGAGCAAGA	up	XLOC_021312	down
PC-5p-27517_164	TACATGCAGAGGTGGAGCAAGA	up	XLOC_021608	down
PC-5p-27517_164	TACATGCAGAGGTGGAGCAAGA	up	XLOC_022533	down
PC-5p-27517_164	TACATGCAGAGGTGGAGCAAGA	up	XLOC_024360	down
PC-5p-27517_164	TACATGCAGAGGTGGAGCAAGA	up	XLOC_025113	down
PC-5p-27517_164	TACATGCAGAGGTGGAGCAAGA	up	XLOC_026429	down
PC-5p-27517_164	TACATGCAGAGGTGGAGCAAGA	up	XLOC_027484	down
PC-5p-27517_164	TACATGCAGAGGTGGAGCAAGA	up	XLOC_027947	down
PC-5p-27517_164	TACATGCAGAGGTGGAGCAAGA	up	XLOC_028496	down
PC-5p-27517_164	TACATGCAGAGGTGGAGCAAGA	up	XLOC_029043	down
PC-5p-27517_164	TACATGCAGAGGTGGAGCAAGA	up	XLOC_029635	down
tni-miR-194_R+1	TGTAACAGCAACTCCATGTGGA	up	XLOC_002223	down
tni-miR-194_R+1	TGTAACAGCAACTCCATGTGGA	up	XLOC_003331	down
tni-miR-194_R+1	TGTAACAGCAACTCCATGTGGA	up	XLOC_003820	down
tni-miR-194_R+1	TGTAACAGCAACTCCATGTGGA	up	XLOC_003836	down
tni-miR-194_R+1	TGTAACAGCAACTCCATGTGGA	up	XLOC_004512	down
tni-miR-194_R+1	TGTAACAGCAACTCCATGTGGA	up	XLOC_004836	down
tni-miR-194_R+1	TGTAACAGCAACTCCATGTGGA	up	XLOC_005806	down
tni-miR-194_R+1	TGTAACAGCAACTCCATGTGGA	up	XLOC_005833	down

tni-miR-194_R+1	TGTAACAGCAACTCCATGTGGA	up	XLOC_006372	down
tni-miR-194_R+1	TGTAACAGCAACTCCATGTGGA	up	XLOC_007047	down
tni-miR-194_R+1	TGTAACAGCAACTCCATGTGGA	up	XLOC_007133	down
tni-miR-194_R+1	TGTAACAGCAACTCCATGTGGA	up	XLOC_007278	down
tni-miR-194_R+1	TGTAACAGCAACTCCATGTGGA	up	XLOC_007291	down
tni-miR-194_R+1	TGTAACAGCAACTCCATGTGGA	up	XLOC_007480	down
tni-miR-194_R+1	TGTAACAGCAACTCCATGTGGA	up	XLOC_008160	down
tni-miR-194_R+1	TGTAACAGCAACTCCATGTGGA	up	XLOC_008196	down
tni-miR-194_R+1	TGTAACAGCAACTCCATGTGGA	up	XLOC_008633	down
tni-miR-194_R+1	TGTAACAGCAACTCCATGTGGA	up	XLOC_009056	down
tni-miR-194_R+1	TGTAACAGCAACTCCATGTGGA	up	XLOC_009429	down
tni-miR-194_R+1	TGTAACAGCAACTCCATGTGGA	up	XLOC_010148	down
tni-miR-194_R+1	TGTAACAGCAACTCCATGTGGA	up	XLOC_010449	down
tni-miR-194_R+1	TGTAACAGCAACTCCATGTGGA	up	XLOC_010710	down
tni-miR-194_R+1	TGTAACAGCAACTCCATGTGGA	up	XLOC_011993	down
tni-miR-194_R+1	TGTAACAGCAACTCCATGTGGA	up	XLOC_012130	down
tni-miR-194_R+1	TGTAACAGCAACTCCATGTGGA	up	XLOC_012380	down
tni-miR-194_R+1	TGTAACAGCAACTCCATGTGGA	up	XLOC_012886	down
tni-miR-194_R+1	TGTAACAGCAACTCCATGTGGA	up	XLOC_012890	down
tni-miR-194_R+1	TGTAACAGCAACTCCATGTGGA	up	XLOC_013142	down
tni-miR-194_R+1	TGTAACAGCAACTCCATGTGGA	up	XLOC_014235	down
tni-miR-194_R+1	TGTAACAGCAACTCCATGTGGA	up	XLOC_014268	down
tni-miR-194_R+1	TGTAACAGCAACTCCATGTGGA	up	XLOC_015097	down
tni-miR-194_R+1	TGTAACAGCAACTCCATGTGGA	up	XLOC_015208	down
tni-miR-194_R+1	TGTAACAGCAACTCCATGTGGA	up	XLOC_015336	down
tni-miR-194_R+1	TGTAACAGCAACTCCATGTGGA	up	XLOC_016514	down
tni-miR-194_R+1	TGTAACAGCAACTCCATGTGGA	up	XLOC_016638	down
tni-miR-194_R+1	TGTAACAGCAACTCCATGTGGA	up	XLOC_016822	down
tni-miR-194_R+1	TGTAACAGCAACTCCATGTGGA	up	XLOC_017213	down
tni-miR-194_R+1	TGTAACAGCAACTCCATGTGGA	up	XLOC_017573	down
tni-miR-194_R+1	TGTAACAGCAACTCCATGTGGA	up	XLOC_019410	down
tni-miR-194_R+1	TGTAACAGCAACTCCATGTGGA	up	XLOC_019745	down
tni-miR-194_R+1	TGTAACAGCAACTCCATGTGGA	up	XLOC_019760	down
tni-miR-194_R+1	TGTAACAGCAACTCCATGTGGA	up	XLOC_020204	down
tni-miR-194_R+1	TGTAACAGCAACTCCATGTGGA	up	XLOC_021524	down
tni-miR-194_R+1	TGTAACAGCAACTCCATGTGGA	up	XLOC_021550	down
tni-miR-194_R+1	TGTAACAGCAACTCCATGTGGA	up	XLOC_022404	down
tni-miR-194_R+1	TGTAACAGCAACTCCATGTGGA	up	XLOC_022740	down
tni-miR-194_R+1	TGTAACAGCAACTCCATGTGGA	up	XLOC_022868	down
tni-miR-194_R+1	TGTAACAGCAACTCCATGTGGA	up	XLOC_024238	down
tni-miR-194_R+1	TGTAACAGCAACTCCATGTGGA	up	XLOC_024360	down
tni-miR-194_R+1	TGTAACAGCAACTCCATGTGGA	up	XLOC_024437	down
tni-miR-194_R+1	TGTAACAGCAACTCCATGTGGA	up	XLOC_024886	down
tni-miR-194_R+1	TGTAACAGCAACTCCATGTGGA	up	XLOC_026204	down
tni-miR-194_R+1	TGTAACAGCAACTCCATGTGGA	up	XLOC_026397	down
tni-miR-194_R+1	TGTAACAGCAACTCCATGTGGA	up	XLOC_026540	down
tni-miR-194_R+1	TGTAACAGCAACTCCATGTGGA	up	XLOC_027117	down
tni-miR-194_R+1	TGTAACAGCAACTCCATGTGGA	up	XLOC_027204	down
tni-miR-194_R+1	TGTAACAGCAACTCCATGTGGA	up	XLOC_027534	down
tni-miR-194_R+1	TGTAACAGCAACTCCATGTGGA	up	XLOC_027947	down
tni-miR-194_R+1	TGTAACAGCAACTCCATGTGGA	up	XLOC_028496	down
tni-miR-194_R+1	TGTAACAGCAACTCCATGTGGA	up	XLOC_028540	down
tni-miR-194_R+1	TGTAACAGCAACTCCATGTGGA	up	XLOC_028813	down
tni-miR-194_R+1	TGTAACAGCAACTCCATGTGGA	up	XLOC_028897	down

PC-5p-8690_526	GATGTTGAGTATCAAACTGTAT	down	XLOC_002434	up
PC-5p-8690_526	GATGTTGAGTATCAAACTGTAT	down	XLOC_004363	up
PC-5p-8690_526	GATGTTGAGTATCAAACTGTAT	down	XLOC_005140	up
PC-5p-8690_526	GATGTTGAGTATCAAACTGTAT	down	XLOC_005187	up
PC-5p-8690_526	GATGTTGAGTATCAAACTGTAT	down	XLOC_008738	up
PC-5p-8690_526	GATGTTGAGTATCAAACTGTAT	down	XLOC_011350	up
PC-5p-8690_526	GATGTTGAGTATCAAACTGTAT	down	XLOC_011516	up
PC-5p-8690_526	GATGTTGAGTATCAAACTGTAT	down	XLOC_015536	up
PC-5p-8690_526	GATGTTGAGTATCAAACTGTAT	down	XLOC_016988	up
PC-5p-8690_526	GATGTTGAGTATCAAACTGTAT	down	XLOC_018222	up
PC-5p-8690_526	GATGTTGAGTATCAAACTGTAT	down	XLOC_018488	up
PC-5p-8690_526	GATGTTGAGTATCAAACTGTAT	down	XLOC_019112	up
PC-5p-8690_526	GATGTTGAGTATCAAACTGTAT	down	XLOC_019924	up
PC-5p-8690_526	GATGTTGAGTATCAAACTGTAT	down	XLOC_021319	up
PC-5p-8690_526	GATGTTGAGTATCAAACTGTAT	down	XLOC_022551	up
PC-5p-8690_526	GATGTTGAGTATCAAACTGTAT	down	XLOC_027531	up
PC-5p-8690_526	GATGTTGAGTATCAAACTGTAT	down	XLOC_029845	up
PC-5p-8690_526	GATGTTGAGTATCAAACTGTAT	down	XLOC_030187	up
tni-miR-10c	TACCCTGTAGATCCGGATTTGT	up	XLOC_001958	down
tni-miR-10c	TACCCTGTAGATCCGGATTTGT	up	XLOC_002645	down
tni-miR-10c	TACCCTGTAGATCCGGATTTGT	up	XLOC_004013	down
tni-miR-10c	TACCCTGTAGATCCGGATTTGT	up	XLOC_004512	down
tni-miR-10c	TACCCTGTAGATCCGGATTTGT	up	XLOC_005372	down
tni-miR-10c	TACCCTGTAGATCCGGATTTGT	up	XLOC_007047	down
tni-miR-10c	TACCCTGTAGATCCGGATTTGT	up	XLOC_008186	down
tni-miR-10c	TACCCTGTAGATCCGGATTTGT	up	XLOC_011569	down
tni-miR-10c	TACCCTGTAGATCCGGATTTGT	up	XLOC_011901	down
tni-miR-10c	TACCCTGTAGATCCGGATTTGT	up	XLOC_012317	down
tni-miR-10c	TACCCTGTAGATCCGGATTTGT	up	XLOC_013135	down
tni-miR-10c	TACCCTGTAGATCCGGATTTGT	up	XLOC_014235	down
tni-miR-10c	TACCCTGTAGATCCGGATTTGT	up	XLOC_014708	down
tni-miR-10c	TACCCTGTAGATCCGGATTTGT	up	XLOC_014853	down
tni-miR-10c	TACCCTGTAGATCCGGATTTGT	up	XLOC_015390	down
tni-miR-10c	TACCCTGTAGATCCGGATTTGT	up	XLOC_016187	down
tni-miR-10c	TACCCTGTAGATCCGGATTTGT	up	XLOC_017111	down
tni-miR-10c	TACCCTGTAGATCCGGATTTGT	up	XLOC_017238	down
tni-miR-10c	TACCCTGTAGATCCGGATTTGT	up	XLOC_017573	down
tni-miR-10c	TACCCTGTAGATCCGGATTTGT	up	XLOC_018481	down
tni-miR-10c	TACCCTGTAGATCCGGATTTGT	up	XLOC_019470	down
tni-miR-10c	TACCCTGTAGATCCGGATTTGT	up	XLOC_019474	down
tni-miR-10c	TACCCTGTAGATCCGGATTTGT	up	XLOC_020110	down
tni-miR-10c	TACCCTGTAGATCCGGATTTGT	up	XLOC_020680	down
tni-miR-10c	TACCCTGTAGATCCGGATTTGT	up	XLOC_026204	down
tni-miR-10c	TACCCTGTAGATCCGGATTTGT	up	XLOC_027484	down
tni-miR-10c	TACCCTGTAGATCCGGATTTGT	up	XLOC_028314	down
tni-miR-10c	TACCCTGTAGATCCGGATTTGT	up	XLOC_028838	down
ccr-miR-99_R+3	AACCCGTAGATCCGATCTTGTGAA	up	XLOC_013175	down
ccr-miR-99_R+3	AACCCGTAGATCCGATCTTGTGAA	up	XLOC_020653	down
ccr-miR-99_R+3	AACCCGTAGATCCGATCTTGTGAA	up	XLOC_021912	down
ccr-miR-99_R+3	AACCCGTAGATCCGATCTTGTGAA	up	XLOC_029513	down
PC-3p-50929_43	TGGAAGTGTCAGAAATTCTGAGT	up	XLOC_000073	down
PC-3p-50929_43	TGGAAGTGTCAGAAATTCTGAGT	up	XLOC_001106	down
PC-3p-50929_43	TGGAAGTGTCAGAAATTCTGAGT	up	XLOC_001886	down
PC-3p-50929_43	TGGAAGTGTCAGAAATTCTGAGT	up	XLOC_005994	down

PC-3p-50929_43	TGGAAGTGTCAGAAATTCTGAGT	up	XLOC_007133	down
PC-3p-50929_43	TGGAAGTGTCAGAAATTCTGAGT	up	XLOC_007143	down
PC-3p-50929_43	TGGAAGTGTCAGAAATTCTGAGT	up	XLOC_007274	down
PC-3p-50929_43	TGGAAGTGTCAGAAATTCTGAGT	up	XLOC_009111	down
PC-3p-50929_43	TGGAAGTGTCAGAAATTCTGAGT	up	XLOC_009175	down
PC-3p-50929_43	TGGAAGTGTCAGAAATTCTGAGT	up	XLOC_009463	down
PC-3p-50929_43	TGGAAGTGTCAGAAATTCTGAGT	up	XLOC_010202	down
PC-3p-50929_43	TGGAAGTGTCAGAAATTCTGAGT	up	XLOC_010475	down
PC-3p-50929_43	TGGAAGTGTCAGAAATTCTGAGT	up	XLOC_011688	down
PC-3p-50929_43	TGGAAGTGTCAGAAATTCTGAGT	up	XLOC_011993	down
PC-3p-50929_43	TGGAAGTGTCAGAAATTCTGAGT	up	XLOC_012298	down
PC-3p-50929_43	TGGAAGTGTCAGAAATTCTGAGT	up	XLOC_012728	down
PC-3p-50929_43	TGGAAGTGTCAGAAATTCTGAGT	up	XLOC_013512	down
PC-3p-50929_43	TGGAAGTGTCAGAAATTCTGAGT	up	XLOC_013544	down
PC-3p-50929_43	TGGAAGTGTCAGAAATTCTGAGT	up	XLOC_013673	down
PC-3p-50929_43	TGGAAGTGTCAGAAATTCTGAGT	up	XLOC_014079	down
PC-3p-50929_43	TGGAAGTGTCAGAAATTCTGAGT	up	XLOC_014319	down
PC-3p-50929_43	TGGAAGTGTCAGAAATTCTGAGT	up	XLOC_015804	down
PC-3p-50929_43	TGGAAGTGTCAGAAATTCTGAGT	up	XLOC_016055	down
PC-3p-50929_43	TGGAAGTGTCAGAAATTCTGAGT	up	XLOC_016109	down
PC-3p-50929_43	TGGAAGTGTCAGAAATTCTGAGT	up	XLOC_017111	down
PC-3p-50929_43	TGGAAGTGTCAGAAATTCTGAGT	up	XLOC_017213	down
PC-3p-50929_43	TGGAAGTGTCAGAAATTCTGAGT	up	XLOC_018678	down
PC-3p-50929_43	TGGAAGTGTCAGAAATTCTGAGT	up	XLOC_020931	down
PC-3p-50929_43	TGGAAGTGTCAGAAATTCTGAGT	up	XLOC_021777	down
PC-3p-50929_43	TGGAAGTGTCAGAAATTCTGAGT	up	XLOC_022279	down
PC-3p-50929_43	TGGAAGTGTCAGAAATTCTGAGT	up	XLOC_023109	down
PC-3p-50929_43	TGGAAGTGTCAGAAATTCTGAGT	up	XLOC_023882	down
PC-3p-50929_43	TGGAAGTGTCAGAAATTCTGAGT	up	XLOC_024271	down
PC-3p-50929_43	TGGAAGTGTCAGAAATTCTGAGT	up	XLOC_025113	down
PC-3p-50929_43	TGGAAGTGTCAGAAATTCTGAGT	up	XLOC_025704	down
PC-3p-50929_43	TGGAAGTGTCAGAAATTCTGAGT	up	XLOC_027753	down
PC-3p-50929_43	TGGAAGTGTCAGAAATTCTGAGT	up	XLOC_027954	down
ola-miR-194-3p_1ss20CT	CCAGTGGAGGTGCTGTTACTTG	up	XLOC_000103	down
ola-miR-194-3p_1ss20CT	CCAGTGGAGGTGCTGTTACTTG	up	XLOC_000168	down
ola-miR-194-3p_1ss20CT	CCAGTGGAGGTGCTGTTACTTG	up	XLOC_001134	down
ola-miR-194-3p_1ss20CT	CCAGTGGAGGTGCTGTTACTTG	up	XLOC_001909	down
ola-miR-194-3p_1ss20CT	CCAGTGGAGGTGCTGTTACTTG	up	XLOC_002037	down
ola-miR-194-3p_1ss20CT	CCAGTGGAGGTGCTGTTACTTG	up	XLOC_002291	down
ola-miR-194-3p_1ss20CT	CCAGTGGAGGTGCTGTTACTTG	up	XLOC_002479	down
ola-miR-194-3p_1ss20CT	CCAGTGGAGGTGCTGTTACTTG	up	XLOC_003093	down
ola-miR-194-3p_1ss20CT	CCAGTGGAGGTGCTGTTACTTG	up	XLOC_003220	down
ola-miR-194-3p_1ss20CT	CCAGTGGAGGTGCTGTTACTTG	up	XLOC_003290	down
ola-miR-194-3p_1ss20CT	CCAGTGGAGGTGCTGTTACTTG	up	XLOC_003928	down
ola-miR-194-3p_1ss20CT	CCAGTGGAGGTGCTGTTACTTG	up	XLOC_003987	down
ola-miR-194-3p_1ss20CT	CCAGTGGAGGTGCTGTTACTTG	up	XLOC_004245	down
ola-miR-194-3p_1ss20CT	CCAGTGGAGGTGCTGTTACTTG	up	XLOC_004628	down
ola-miR-194-3p_1ss20CT	CCAGTGGAGGTGCTGTTACTTG	up	XLOC_004775	down
ola-miR-194-3p_1ss20CT	CCAGTGGAGGTGCTGTTACTTG	up	XLOC_005968	down
ola-miR-194-3p_1ss20CT	CCAGTGGAGGTGCTGTTACTTG	up	XLOC_006303	down
ola-miR-194-3p_1ss20CT	CCAGTGGAGGTGCTGTTACTTG	up	XLOC_007080	down
ola-miR-194-3p_1ss20CT	CCAGTGGAGGTGCTGTTACTTG	up	XLOC_007133	down
ola-miR-194-3p_1ss20CT	CCAGTGGAGGTGCTGTTACTTG	up	XLOC_007386	down
ola-miR-194-3p_1ss20CT	CCAGTGGAGGTGCTGTTACTTG	up	XLOC_007480	down

ola-miR-194-3p_1ss20CT	CCAGTGGAGGTGCTGTTACTTG	up	XLOC_008521	down
ola-miR-194-3p_1ss20CT	CCAGTGGAGGTGCTGTTACTTG	up	XLOC_009429	down
ola-miR-194-3p_1ss20CT	CCAGTGGAGGTGCTGTTACTTG	up	XLOC_009449	down
ola-miR-194-3p_1ss20CT	CCAGTGGAGGTGCTGTTACTTG	up	XLOC_009743	down
ola-miR-194-3p_1ss20CT	CCAGTGGAGGTGCTGTTACTTG	up	XLOC_010187	down
ola-miR-194-3p_1ss20CT	CCAGTGGAGGTGCTGTTACTTG	up	XLOC_010284	down
ola-miR-194-3p_1ss20CT	CCAGTGGAGGTGCTGTTACTTG	up	XLOC_010407	down
ola-miR-194-3p_1ss20CT	CCAGTGGAGGTGCTGTTACTTG	up	XLOC_010820	down
ola-miR-194-3p_1ss20CT	CCAGTGGAGGTGCTGTTACTTG	up	XLOC_012380	down
ola-miR-194-3p_1ss20CT	CCAGTGGAGGTGCTGTTACTTG	up	XLOC_013076	down
ola-miR-194-3p_1ss20CT	CCAGTGGAGGTGCTGTTACTTG	up	XLOC_013381	down
ola-miR-194-3p_1ss20CT	CCAGTGGAGGTGCTGTTACTTG	up	XLOC_013584	down
ola-miR-194-3p_1ss20CT	CCAGTGGAGGTGCTGTTACTTG	up	XLOC_013780	down
ola-miR-194-3p_1ss20CT	CCAGTGGAGGTGCTGTTACTTG	up	XLOC_014268	down
ola-miR-194-3p_1ss20CT	CCAGTGGAGGTGCTGTTACTTG	up	XLOC_014376	down
ola-miR-194-3p_1ss20CT	CCAGTGGAGGTGCTGTTACTTG	up	XLOC_014412	down
ola-miR-194-3p_1ss20CT	CCAGTGGAGGTGCTGTTACTTG	up	XLOC_014584	down
ola-miR-194-3p_1ss20CT	CCAGTGGAGGTGCTGTTACTTG	up	XLOC_015097	down
ola-miR-194-3p_1ss20CT	CCAGTGGAGGTGCTGTTACTTG	up	XLOC_015248	down
ola-miR-194-3p_1ss20CT	CCAGTGGAGGTGCTGTTACTTG	up	XLOC_016288	down
ola-miR-194-3p_1ss20CT	CCAGTGGAGGTGCTGTTACTTG	up	XLOC_016447	down
ola-miR-194-3p_1ss20CT	CCAGTGGAGGTGCTGTTACTTG	up	XLOC_016555	down
ola-miR-194-3p_1ss20CT	CCAGTGGAGGTGCTGTTACTTG	up	XLOC_018046	down
ola-miR-194-3p_1ss20CT	CCAGTGGAGGTGCTGTTACTTG	up	XLOC_018735	down
ola-miR-194-3p_1ss20CT	CCAGTGGAGGTGCTGTTACTTG	up	XLOC_020036	down
ola-miR-194-3p_1ss20CT	CCAGTGGAGGTGCTGTTACTTG	up	XLOC_020060	down
ola-miR-194-3p_1ss20CT	CCAGTGGAGGTGCTGTTACTTG	up	XLOC_020525	down
ola-miR-194-3p_1ss20CT	CCAGTGGAGGTGCTGTTACTTG	up	XLOC_020758	down
ola-miR-194-3p_1ss20CT	CCAGTGGAGGTGCTGTTACTTG	up	XLOC_020931	down
ola-miR-194-3p_1ss20CT	CCAGTGGAGGTGCTGTTACTTG	up	XLOC_021626	down
ola-miR-194-3p_1ss20CT	CCAGTGGAGGTGCTGTTACTTG	up	XLOC_022566	down
ola-miR-194-3p_1ss20CT	CCAGTGGAGGTGCTGTTACTTG	up	XLOC_023109	down
ola-miR-194-3p_1ss20CT	CCAGTGGAGGTGCTGTTACTTG	up	XLOC_023237	down
ola-miR-194-3p_1ss20CT	CCAGTGGAGGTGCTGTTACTTG	up	XLOC_024289	down
ola-miR-194-3p_1ss20CT	CCAGTGGAGGTGCTGTTACTTG	up	XLOC_025873	down
ola-miR-194-3p_1ss20CT	CCAGTGGAGGTGCTGTTACTTG	up	XLOC_026503	down
ola-miR-194-3p_1ss20CT	CCAGTGGAGGTGCTGTTACTTG	up	XLOC_028287	down
ola-miR-194-3p_1ss20CT	CCAGTGGAGGTGCTGTTACTTG	up	XLOC_028290	down
ola-miR-194-3p_1ss20CT	CCAGTGGAGGTGCTGTTACTTG	up	XLOC_029043	down
ola-miR-194-3p_1ss20CT	CCAGTGGAGGTGCTGTTACTTG	up	XLOC_029160	down
ssa-miR-206-3p	TGGAATGTAAGGAAGTGTGTGG	up	XLOC_001513	down
ssa-miR-206-3p	TGGAATGTAAGGAAGTGTGTGG	up	XLOC_003559	down
ssa-miR-206-3p	TGGAATGTAAGGAAGTGTGTGG	up	XLOC_003822	down
ssa-miR-206-3p	TGGAATGTAAGGAAGTGTGTGG	up	XLOC_005515	down
ssa-miR-206-3p	TGGAATGTAAGGAAGTGTGTGG	up	XLOC_006238	down
ssa-miR-206-3p	TGGAATGTAAGGAAGTGTGTGG	up	XLOC_006315	down
ssa-miR-206-3p	TGGAATGTAAGGAAGTGTGTGG	up	XLOC_008633	down
ssa-miR-206-3p	TGGAATGTAAGGAAGTGTGTGG	up	XLOC_009449	down
ssa-miR-206-3p	TGGAATGTAAGGAAGTGTGTGG	up	XLOC_010148	down
ssa-miR-206-3p	TGGAATGTAAGGAAGTGTGTGG	up	XLOC_010187	down
ssa-miR-206-3p	TGGAATGTAAGGAAGTGTGTGG	up	XLOC_011901	down
ssa-miR-206-3p	TGGAATGTAAGGAAGTGTGTGG	up	XLOC_011962	down
ssa-miR-206-3p	TGGAATGTAAGGAAGTGTGTGG	up	XLOC_012298	down
ssa-miR-206-3p	TGGAATGTAAGGAAGTGTGTGG	up	XLOC_013076	down

ssa-miR-206-3p	TGGAATGTAAGGAAGTGTGTGG	up	XLOC_014039	down
ssa-miR-206-3p	TGGAATGTAAGGAAGTGTGTGG	up	XLOC_014096	down
ssa-miR-206-3p	TGGAATGTAAGGAAGTGTGTGG	up	XLOC_014235	down
ssa-miR-206-3p	TGGAATGTAAGGAAGTGTGTGG	up	XLOC_015415	down
ssa-miR-206-3p	TGGAATGTAAGGAAGTGTGTGG	up	XLOC_016046	down
ssa-miR-206-3p	TGGAATGTAAGGAAGTGTGTGG	up	XLOC_016109	down
ssa-miR-206-3p	TGGAATGTAAGGAAGTGTGTGG	up	XLOC_016203	down
ssa-miR-206-3p	TGGAATGTAAGGAAGTGTGTGG	up	XLOC_016810	down
ssa-miR-206-3p	TGGAATGTAAGGAAGTGTGTGG	up	XLOC_017573	down
ssa-miR-206-3p	TGGAATGTAAGGAAGTGTGTGG	up	XLOC_018413	down
ssa-miR-206-3p	TGGAATGTAAGGAAGTGTGTGG	up	XLOC_018735	down
ssa-miR-206-3p	TGGAATGTAAGGAAGTGTGTGG	up	XLOC_020110	down
ssa-miR-206-3p	TGGAATGTAAGGAAGTGTGTGG	up	XLOC_023324	down
ssa-miR-206-3p	TGGAATGTAAGGAAGTGTGTGG	up	XLOC_024226	down
ssa-miR-206-3p	TGGAATGTAAGGAAGTGTGTGG	up	XLOC_024276	down
ssa-miR-206-3p	TGGAATGTAAGGAAGTGTGTGG	up	XLOC_024363	down
ssa-miR-206-3p	TGGAATGTAAGGAAGTGTGTGG	up	XLOC_028701	down
ssa-miR-206-3p	TGGAATGTAAGGAAGTGTGTGG	up	XLOC_030306	down
dre-miR-122	TGGAGTGTGACAATGGTGTTTG	up	XLOC_000725	down
dre-miR-122	TGGAGTGTGACAATGGTGTTTG	up	XLOC_001134	down
dre-miR-122	TGGAGTGTGACAATGGTGTTTG	up	XLOC_002479	down
dre-miR-122	TGGAGTGTGACAATGGTGTTTG	up	XLOC_002781	down
dre-miR-122	TGGAGTGTGACAATGGTGTTTG	up	XLOC_004712	down
dre-miR-122	TGGAGTGTGACAATGGTGTTTG	up	XLOC_006450	down
dre-miR-122	TGGAGTGTGACAATGGTGTTTG	up	XLOC_009011	down
dre-miR-122	TGGAGTGTGACAATGGTGTTTG	up	XLOC_009780	down
dre-miR-122	TGGAGTGTGACAATGGTGTTTG	up	XLOC_010321	down
dre-miR-122	TGGAGTGTGACAATGGTGTTTG	up	XLOC_010501	down
dre-miR-122	TGGAGTGTGACAATGGTGTTTG	up	XLOC_010820	down
dre-miR-122	TGGAGTGTGACAATGGTGTTTG	up	XLOC_012317	down
dre-miR-122	TGGAGTGTGACAATGGTGTTTG	up	XLOC_012632	down
dre-miR-122	TGGAGTGTGACAATGGTGTTTG	up	XLOC_013081	down
dre-miR-122	TGGAGTGTGACAATGGTGTTTG	up	XLOC_013154	down
dre-miR-122	TGGAGTGTGACAATGGTGTTTG	up	XLOC_013584	down
dre-miR-122	TGGAGTGTGACAATGGTGTTTG	up	XLOC_014353	down
dre-miR-122	TGGAGTGTGACAATGGTGTTTG	up	XLOC_014853	down
dre-miR-122	TGGAGTGTGACAATGGTGTTTG	up	XLOC_015696	down
dre-miR-122	TGGAGTGTGACAATGGTGTTTG	up	XLOC_015955	down
dre-miR-122	TGGAGTGTGACAATGGTGTTTG	up	XLOC_016109	down
dre-miR-122	TGGAGTGTGACAATGGTGTTTG	up	XLOC_016724	down
dre-miR-122	TGGAGTGTGACAATGGTGTTTG	up	XLOC_018413	down
dre-miR-122	TGGAGTGTGACAATGGTGTTTG	up	XLOC_018735	down
dre-miR-122	TGGAGTGTGACAATGGTGTTTG	up	XLOC_020060	down
dre-miR-122	TGGAGTGTGACAATGGTGTTTG	up	XLOC_027753	down

Table S11 Primer design of DE miRNA

Name	Primer sequence (5'-3')
**miR-1：**	CCGCGTGGAATGTAAAGAAGT
**miR-1338-5p：**	CCGCAAGGACTGTCCAACC
**miR-16b-5p：**	CCAAGGCAGTAGCAGCACG
**miR-122：**	CTGGAGTGTGACAATGGTGTTT
**miR-22a-3p：**	CAAAGAAGCTGCCAGCTGAAG
**Let-7j：**	CCGCGTGAGGTAGTAGTTTGTG
**Let-7d-5p：**	TCGCGTGAGGTAGTTGGTTGT
**PC-5p-27517：**	CAGCGTACATGCAGAGGTGG
**PC-3p-50929：**	CCAGCGTGTGGAAGTGTCAGA
**miR-194：**	CCGCGTGTAACAGCAACTCC
**miR-10c：**	TCGCGTACCCTGTAGATCCG
**miR-99：**	ACGAGAACCCGTAGATCCGAT
**miR-194-3p：**	TCGCCAGTGGAGGTGCTG
**miR-206-3p：**	CCGCGTGGAATGTAAGGAAG
**miR-462：**	CCGCGTAACGGAACCCAT

Table S12 Primer design of DE mRNA

Name	Primer sequence (5'-3')
**Complement C3**	F: 5'-CAGGCAGGAGGATGTATCGG-3'<br>R: 5'- TGCCAGCGTCAAGTCTTTTCT-3'
**GHITM**	F: 5'- GTGGGAGGTCTGTCTACTGTTGC-3'<br>R: 5'- TCCGAATGCTGAGGTGGG-3'
**TNIP1**	F: 5'- CTCCCTACTGTGGTCCCTTTG-3'<br>R: 5'- TCTGGTGGTGGTCGTGAACT-3'
**HMOX1**	F: 5'- GGCAGCGATTTATCAGAGCAGA-3'<br>R: 5'- CGTAGAGGGAGCACAGCAGGA-3'
**FADS2**	F: 5'- AACTCAACCAGAGGAATACGGG-3'<br>R: 5'-GAAGCGAAGGTAGAATGAGATGAAC-3'
**GLRX**	F: 5'-ATGACAAGGCAGCGGGACT-3'<br>R: 5'- TTACAGGGCAACAGCAAGATTA-3'
**MSMO1**	F: 5'- GGTAGACGGGACATACAACGG-3'<br>R: 5'- TCAGGGAGGGCTGGAACG-3'
**DGAT2**	F: 5'- CCTTGCAACCCTAGCAGGAA-3'<br>R: 5'- GACGAGGCGAGGAGTTTGAA-3'
**SC5d**	F: 5'- CCTCCCTCCATCGTTGTTCC-3'<br>R: 5'- TTCTCGCCATACCTGATTCTCC-3'
**EPHX2**	F: 5'- CTGCTGCCGTCCTTTACCA-3'<br>R: 5'- GGGAGCCACAGTACCACCTT-3'
**SHMT1**	F: 5'- CTGCTGCCGTCCTTTACCA-3'<br>R: 5'- GGGAGCCACAGTACCACCTT-3'
**ACOX1**	F: 5'- CAGATGTGCGGAGGGTGC-3'<br>R: 5'- CCTGACTCTTCCTGCGTTGC-3'
**HSP90b1**	F: 5'- GCCTCGGATGCTCTGGATA-3'<br>R: 5'- GCCTGACTTGGCAATGGTT-3'

**MFAP4**	F: 5'- TTGATGGTTGAGGGTAATTTGG-3'<br>R: 5'- GAAGCAGCTTAGTCGTCGTTTG-3'
**EGLN2**	F: 5'- GAACAGACGGACCTTGTAAACCC-3'<br>R: 5'- CGCCGCACATAAGCCATT-3'
**AQP10a**	F: 5'- TAAGTTGGCTTTTCACGGTCTG-3'<br>R: 5'- GAAGTGGCTGGTGGTGGGT-3'
**MASP1**	F: 5'- AGTGCTGAGTTCGGTCCGTT-3'<br>R: 5'- GATTGGAAAGGGTTCATCAGG-3'
**MGST3b**	F: 5'- TGTACTTCTTTCTGGCTCCCC-3'<br>R: 5'- TTCCTCCTCTTTCCTGCGTTA-3'
**CYP3a65**	F: 5'- CGTCCAGGTGGTTGGTGAA-3'<br>R: 5'- CAGGCTCCCAGCTCGTTT-3'
**AGXTB**	F: 5'- GCAAACAATCAGATGACAACCTC-3'<br>R: 5'- CCTCCAGCTCTTAAAACAACTAAAC-3'
**PCK2**	F: 5'- GATGCTCCCGTGCGTCTG-3'<br>R: 5'- CGGTTTCTCCCCTCCACTATG-3'
**ADH5**	F: 5'- ACAGCGTTCACATTCCCCACA-3'<br>R: 5'- TGAGACTGGAAAAGGGATGAAA-3'
**CLDN2**	F: 5'- CAGCGTGCCCAGATAATGC-3'<br>R: 5'- CCAGTCACTTCCCCTCCAAA-3'
**CALR3a**	F: 5'- GTGTCTGCTTTCCTGCGGTT-3'<br>R: 5'- CGGTCTCGTGCCTGCTAATC-3'
**ACACA**	F: 5'- TTCGGATGCCGTGTTCAA-3'<br>R: 5'- AGCTTCCAGGAGTTCACAAAGA-3'
**18s rRNA**	F: 5'-GGCCGTTCTTAGTTGGTGGA-3'<br>R: 5'-TTGCTCAATCTCGTGTGGCT-3'
